# In-depth proteomic analyses of *Haliotis laevigata* (greenlip abalone) nacre and prismatic organic shell matrix

**DOI:** 10.1186/s12953-018-0139-3

**Published:** 2018-06-15

**Authors:** Karlheinz Mann, Nicolas Cerveau, Meike Gummich, Monika Fritz, Matthias Mann, Daniel J. Jackson

**Affiliations:** 1 Abteilung Proteomics und Signaltransduktion, Max-Planck-Institut für Biochemie, Am Klopferspitz 18, D-82152 Martinsried, Germany; 2 Department of Geobiology, Georg-August University of Göttingen, Goldschmidstr. 3, 37077 Göttingen, Germany; 3 Universität Bremen, Institut für Biophysik, Otto Hahn Allee NW1, D-28334 Bremen, Germany

**Keywords:** Biomineralization, Mantle transcriptome, Shell organic matrix, Nacre, Prismatic layer, Proteome

## Abstract

**Background:**

The shells of various *Haliotis* species have served as models of invertebrate biomineralization and physical shell properties for more than 20 years. A focus of this research has been the nacreous inner layer of the shell with its conspicuous arrangement of aragonite platelets, resembling in cross-section a brick-and-mortar wall. In comparison, the outer, less stable, calcitic prismatic layer has received much less attention. One of the first molluscan shell proteins to be characterized at the molecular level was Lustrin A, a component of the nacreous organic matrix of *Haliotis rufescens*. This was soon followed by the C-type lectin perlucin and the growth factor-binding perlustrin, both isolated from *H. laevigata* nacre, and the crystal growth-modulating AP7 and AP24, isolated from *H. rufescens* nacre. Mass spectrometry-based proteomics was subsequently applied to to *Haliotis* biomineralization research with the analysis of the *H. asinina* shell matrix and yielded 14 different shell-associated proteins. That study was the most comprehensive for a *Haliotis* species to date.

**Methods:**

The shell proteomes of nacre and prismatic layer of the marine gastropod *Haliotis laevigata* were analyzed combining mass spectrometry-based proteomics and next generation sequencing.

**Results:**

We identified 297 proteins from the nacreous shell layer and 350 proteins from the prismatic shell layer from the green lip abalone *H. laevigata*. Considering the overlap between the two sets we identified a total of 448 proteins. Fifty-one nacre proteins and 43 prismatic layer proteins were defined as major proteins based on their abundance at more than 0.2% of the total. The remaining proteins occurred at low abundance and may not play any significant role in shell fabrication. The overlap of major proteins between the two shell layers was 17, amounting to a total of 77 major proteins.

**Conclusions:**

The *H. laevigata* shell proteome shares moderate sequence similarity at the protein level with other gastropod, bivalve and more distantly related invertebrate biomineralising proteomes. Features conserved in *H. laevigata* and other molluscan shell proteomes include short repetitive sequences of low complexity predicted to lack intrinsic three-dimensional structure, and domains such as tyrosinase, chitin-binding, and carbonic anhydrase. This catalogue of *H. laevigata* shell proteins represents the most comprehensive for a haliotid and should support future efforts to elucidate the molecular mechanisms of shell assembly.

**Electronic supplementary material:**

The online version of this article (10.1186/s12953-018-0139-3) contains supplementary material, which is available to authorized users.

## Background

Species of the gastropod genus *Haliotis* construct a shell with two clearly distinguishable major layers, prismatic and nacreous, both of which are a composite of mineralized CaCO_3_ and organic molecules [1, 2]. The outer, relatively soft and chalky prismatic layer is comprised of prism-shaped crystals. The inner mother-of-pearl layer, or nacre, is characterized by thin intercalated plates and has attracted much more interest as a model in biomaterials and biomineralization research than the prismatic layer. This is due to its extraordinary toughness and fracture resistance conferred by the arrangement of individual aragonite crystals which are connected by mineral bridges and enclosed by a thin layer of organic matrix [3–6].

In both layers the crystals are enveloped and pervaded by an organic matrix that constitutes approximately 2% of the total bio-composite weight, and which is composed predominantly of protein and polysaccharide. The mineral and organic precursors of the shell are secreted by the mantle epithelium that lines the extrapallial space between mantle tissue and the shell [7]. The secreted organic matrix is thought to assemble extracellularly and to provide a mold that templates and guides the growth of the mineral [4]. In fact isolated *H. rufescens* organic shell matrix was shown to control nucleation, crystal orientation, the nature of the calcium carbonate polymorph deposited [8–11], and to act as an adhesive between the aragonitic plates [12].

The search for individual proteins responsible for these effects by molecular biological and biochemical methods lead to the discovery in *H. rufescens* nacre of lustrin A, a large multi-domain protein [13] that is localised immunohistochemically to the extra-crystalline matrix between nacre plates [12]. Other *Haliotis* proteins isolated and characterized include the mineral-binding C-type lectin perlucin [14, 15], the IGF-binding protein perlustrin [16], the mineral-binding proteins AP7 and AP24 [17], the crystal morphology-modifying AP8 proteins [18], the crystal growth-inhibitor perlwapin [19], and perlinhibins, low-abundance Cys-, His- and Arg-rich mini-proteins that inhibit calcium carbonate crystallization [20]. More recently increased application of mass spectrometry-based proteomic techniques to biomineral matrices has enabled the identification of comparatively large numbers of proteins in a short time without the need to resort to complicated protein separation protocols. However, for these proteomic methods one still requires sequence databases as comprehensive as possible to obtain meaningful results. Examples of the application of such proteomic methods to haliotids with relatively limited EST databases created by Sanger sequencing include analyses of the shell organic matrix in *H. asinina* [21] and *H. tuberculata* [22]. Altogether 21 proteins were identified by searching mass spectra against translated EST sequences of *H. asinina*. Perlwapin was the only protein among these 21 that had been previously identified. The study on *H. asinina* [21] compared the proteomes of the entire shell and nacre alone. Five proteins were identified in the whole shell but not in nacre, indicating that they were restricted to the prismatic layer and may induce the formation of prisms or the inhibition of nacre. Similarly, differences in protein composition were also found in more comprehensive studies of separate shell layers of the pearl oysters *Pinctada margaritifera* and *P. maxima* [23] and various *Mytilus* species [24, 25].

Next generation sequencing (NGS) techniques have developed rapidly and allow for the rapid sequencing of entire genomes and transcriptomes that can be used to study not only the expression of biomineralization-related genes, but also as sequence databases for more comprehensive proteomic studies. In the present report we have conducted an in-depth proteomic analysis of the separated prismatic and nacreous layer organic matrices of *H. laevigata* coupled with transcriptomic sequencing of *Haliotis* mantle tissue. The resulting shell-associated proteome included almost all previously identified *Haliotis* proteins as well as many new proteins that were annotated with respect to abundance, similarity to other proteins, predicted domain structure, predicted secretion signal peptide and transmembrane segments, isoelectric point, amino acid composition, and predicted intrinsic disorder. We have also compared these proteins with similarly derived datasets from a range of other molluscs and more distantly related invertebrates in order to determine what broad level of sequence similarity exists between these biomineralising proteomes.

## Methods

### Preparation of matrix and peptides

*Haliotis laevigata* shells of lengths of 15-18 cm and weights of 150-200 g were treated with a final concentration of 4% sodium hypochlorite solution (Carl Roth, Karlsruhe, Germany) for 2 h without (method A) or with (method B) a 5 min ultrasound treatment at the start of each hour. Shells were then washed extensively with deionized water and dried. Alternatively, the nacreous layer of a shell not washed with hypochlorite before was sand-blasted from each side to remove possible contaminants (method C). Nacre matrix was prepared as described previously [26]. For prismatic shell layer preparation the surface of shells was cleaned mechanically to remove mineralized worm tubes and other material not belonging to the shell. Shells were then washed with hypochlorite as before (methods A and B) and the prismatic shell layer was filed off and collected for demineralization. Calcite powder and nacre pieces were dissolved in 12% acetic acid and the suspension was dialyzed and stored in 3% acetic acid at 4 °C for 13 days until centrifugation. Acid-soluble and acid-insoluble matrix components were separated by ultracentrifugation (Optima LE 80 K, 45Ti rotor, Beckman Coulter, Krefeld, Germany) at 4 °C and 146,900 x g for 60 min. The fractions were then lyophilized for concentration and storage. Matrix proteins were separated by SDS-PAGE in pre-cast 4–12% Novex Bis-Tris gels using the MES buffer system with reagents and protocols supplied by the manufacturer (Invitrogen, Carlsbad, CA) except for the reducing agent, which was β-mercaptoethanol added to a final concentration of 2%. The sample buffer contained lithium dodecyl sulphate (LDS, final concentration 1%) while pre-cast gels and running buffer contained SDS (0.1%). Samples were suspended in 30 μl sample buffer/200 μg of organic matrix, boiled for 5 min, and centrifuged at 13000 rpm for 5 min in an Eppendorf bench-top centrifuge before SDS-PAGE analysis. Separated proteins were stained with colloidal Coomassie blue (Invitrogen). Gels containing acid-soluble nacre matrix and acid-insoluble prismatic layer matrix were cut into 12 slices and identical slices of three lanes were used for in-gel digestion with trypsin [27]. All slices were treated equally irrespective of staining intensity or presence of visible bands. The eluted peptides were cleaned with C18 Stage Tips before MS analysis [28]. The acid-soluble fraction of the prismatic layer and the acid-insoluble matrix of nacre, PAGE analysis of which showed no or only few and week protein bands, respectively, were cleaved using a filter-aided sample preparation (FASP) method [29, 30] modified as follows. Matrix components were dissolved in 0.1 M Tris buffer, pH 8, containing 6 M guanidine and 0.01 M dithiothreitol (DTT) and heated to 56 °C for 60 min. Aliquots containing 200, 400 and 800 μg of matrix were then loaded onto Microcon YM-30 centrifugal filter devices (Millipore) and DTT was removed by centrifugation at 13000 rpm in a Eppendorf bench top centrifuge model 5415D for 10 min and washing with 2 x 1vol of the same buffer. Carbamidomethylation was performed in the device using Tris-guanidine buffer containing 0.05 mM iodoacetamide and incubation for 45 min in the dark. Carbamidomethylated proteins were washed with 0.05 M ammonium hydrogen carbonate buffer, pH 8, containing 2 M urea, and centrifugation as before. Trypsin (2 μg, Sequencing grade, modified; Promega, Madison, USA) was added in 40 μl of the same buffer and the devices were incubated at 37 °C for 16 h. Peptides were collected by centrifugation and the filters were washed twice with 40 μl of buffer. The peptide solution was acidified to pH 1–2 with trifluoroacetic acid and peptides were cleaned and concentrated using C18 Stage Tips [28].

### LC-MS and MS data analysis and transcriptomics

Peptide mixtures were fractionated by on-line nanoflow liquid chromatography using the EASY-nLC 1000 system (Thermo Fisher Scientific, Germany) with 20 cm capillary columns of an internal diameter of 75 μm and filled with 1.8 μm Reprosil-Pur C18-AQ resin (Dr. Maisch GmbH, Ammerbuch-Entringen, Germany). Column temperature was 30 °C. The gradient consisted of 5–30% buffer B (80% acetonitrile in 0.1% formic acid) for 85 min, 30–60% buffer B for 12 min and 60–80% buffer B for 7 min at a flow rate of 250 nl/min. The eluate was electrosprayed into an LTQ Orbitrap Velos or Orbitrap Elite (Thermo Fisher Scientific, Germany) through a Proxeon nanoelectrospray ion source. The Orbitrap Velos and Orbitrap Elite were operated in a HCD top 10 mode essentially as described ([31](Velos),[32] (Elite)). Survey full scan spectra (from m/z 300–1750) were acquired at a resolution of 30,000 (Velos) and 120,000 (Elite) at m/z 400. Dynamic exclusion time was 90s. Raw files were processed using version 1.5.1.6 of MaxQuant [33–36] a computational proteomics platform based on the Andromeda search engine (http://www.coxdocs.org/doku.php?id=maxquant:start) [37]. The protein databases used for protein identification were derived from *H. laevigata* hemolymph and epipodial tentacle tissue [38] and mantle tissue (see below). The hemolymph and tentatacle database was kindly provided by Dr. Shiel (Department of Genetics, La Trobe Institute for Molecular Science, La Trobe University, Melbourne) in the form of a nucleotide database that we translated into protein sequences using the EMBOSS Transeq program (http://www.ebi.ac.uk/Tools/st/emboss_transeq/) [39] with six reading frame translation, trim option and the standard code. Because this transcriptomic database has not yet been deposited in a publicly accessible database, we have compiled accessions confirmed by peptide MS/MS sequences in Additional file 1. This file contains the sequence with the most peptide matches occurring in the respective MaxQuant output table protein group. In addition we generated a new mantle tissue transcriptome for what turned out in retrospect to apparently be a hybrid species between *H. laevigata* and *H. rubra*. Briefly, the mantle tissue from an animal collected from Ocean Wave Seafoods (Lara, Victoria, Australia) was dissected and total RNA extracted using TriReagent according to the manufacturer’s instructions. Total RNA was used for Illumina library preparation and 100 bp paired end stranded sequencing on the HiSeq2000 platform. We collected more than 137 million reads which have been deposited in GenBank under SRP126753. Trimmomatic [40] was used to remove low quality reads and adapter sequences. Reads were assembled de novo using our recently developed assembly pipeline [41]. Briefly, we employed three assembly packages with unique assembly strategies: Trinity V2.0.3 [42], the commercial CLC Genomics Workbench and IDBA-tran V1.1.1 [43]. This transcriptome assembly was complemented by a *Haliotis* sequence subset of the UniProt protein sequence database (release 1015–06; 1404 entries with *Haliotis* as organism). Databases were combined with the reversed sequences and sequences of widespread contaminants, such as human keratins. Carbamidomethylation was set as fixed modification. Variable modifications were methionine oxidation, N-acetyl (protein), pyro-Glu/Gln (N-term) and phosphorylation (S,T,Y). Maximal peptide mass tolerance was set to 20 ppm and 6 ppm for first search and main search, respectively. MS/MS mass tolerance was set to a maximal value of 20 ppm. Two missed cleavages were allowed and the minimal length required for a peptide was seven amino acids. Maximal FDR for peptide spectral match, proteins and site was set to 0.01. The minimal score for modified and unmodified peptides was 60. Identifications with only two sequence-unique peptides were routinely validated with the help of the MaxQuant Expert System software of MaxQuant [44] considering the assignment of major peaks, occurrence of uninterrupted y- or b-ion series of at least four consecutive amino acids, preferred cleavages N-terminal to proline bonds, the possible presence of a2/b2 ion pairs, immonium ions and mass accuracy. Only identifications with at least two peptides in a preparation and occurring in at least two preparations of the same shell layer were accepted. Identifications with only one sequence-unique peptide or only in one fraction were exceptionally accepted if only one measurable peptide was predicted under regular cleavage conditions or if it shared peptides with other proteins. The iBAQ (intensity-based absolute quantification) [36] option of MaxQuant was used to calculate, based on the sum of peak intensities, the approximate share of each protein in the total proteome, including identifications that were not accepted finally. The mass spectrometry proteomics data have been deposited to the ProteomeXchange Consortium via the PRIDE [45] partner repository (https://www.ebi.ac.uk/pride/archive/) with the dataset identifier PXD009567.

### Other bioinformatics analyses

Protein similarity searches were performed using FASTA (http://www.ebi.ac.uk/Tools/sss/fasta/) [37] against the UniProt Knowledgebase. Some published sequences not in public protein databases were searched against *H. laevigata* sequences using the Local Blast function [46] of BioEdit Sequence Alignment Editor v.7.2.5 (http://www.mbio.ncsu.edu/bioedit/bioedit.html). Domain prediction including prediction of signal peptides and transmembrane segments was done with InterProScan (http://www.ebi.ac.uk/interpro/search/sequence-search) [47]. Signal peptide prediction was confirmed using SignalP 4.1 (http://www.cbs.dtu.dk/services/SignalP/) [48]. Intrinsically disordered proteins (IDP) and intrinsically disordered regions (IDR) were predicted with MFDp2 [49–51] (http://biomine.cs.vcu.edu/servers/MFDp2/). Sequence alignments were done with the help of Clustal Omega (http://www.ebi.ac.uk/Tools/msa/clustalo/) [39]. Amino acid composition and isoelectric point of protein sequences were calculated using the ExPasy tool ProtParam (http://web.expasy.org/protparam/) after removal of predicted signal peptide sequences [52]. Venn diagrams were drawn using Venn Diagram Plotter (https://omics.pnl.gov/software/venn-diagram-plotter). Some sequences were analysed for tandem repeats using XSTREAM (http://jimcooperlab.mcdb.ucsb.edu/ xstream/) [53]. In some cases the results were checked with PrDOS [54] (http://prdos.hgc.jp/cgi-bin/top.cgi) and IUPred [55] (http://iupred.enzim.hu/pred.php). BLASTp sequence similarity comparisons of the 77 major *H. laevigata* shell proteins described in Table 1 (and in addition 3 contigs encoding UP6 and UP7 as described in [21]) were performed against a variety of calcifying proteome datasets derived from a wide phylogenetic range of metazoans as described in [56]. These included: 42 proteins from the oyster *Pinctada maxima* reported in [23]; 78 proteins from the oyster *Pinctada margaritifera* reported in [23]; 94 proteins from the abalone *Haliotis asinina* reported in [21, 57]; 63 protein from the limpet *Lottia gigantea* reported in [58]; 53 proteins from the oyster *Crassostrea gigas* reported in [59]; 71 proteins from the mussel *Mya truncata* reported in [60]; 59 proteins from the grove snail *Cepaea nemoralis* reported in [56]; 44 proteins from the oyster *Pinctada fucata* reported in [61]; 53 proteins from the mussel *Mytilus coruscus* reported in [24]; 66 proteins from the brachiopod *Magellania venosa* reported in [62]; 139 proteins from the sea urchin *Strongylocentrotus purpuratus* reported in [63]; 37 proteins from the coral *Acropora millepora* reported in [64]. A consensus phylogenetic tree was manually constructed for all of these species based on a selection of previous studies [65–68].

**Table 1:** Major proteins (≥ 0.2% of total in at least two fractions) of the *Haliotis laevigata* shell

Protein	Accession	Abundance (% of total)^a^										Predicted domains^b^ and other features	Ref^c^
		N_AS_	N_BS_	N_CS_	N_AI_	N_BI_	N_CI_	P_AS_	P_BS_	P_AI_	P_BI_		
Actin(s)	Tri_131427, Comp103470_c1_ seq20_6	0.05	0.14	0.03	0.1	0.26	0.34	0.01	0.10	0.17	0.32		
Similar to tyramine beta-hydroxylase/temptin	idb_10968, Comp112534_c0_seq1_2	0.26	0.17	0.22	0.09	0.06	0.05	–	–	–	–	SSP, IDR (C-term); R/G	
Uncharacterized	Comp128817_c0_seq1_3, idb_42198	–	0.01	0.02	–	–	–	0.01	–	0.48	0.30	hirudin_antistatin, IDR; P	
Uncharacterized	Comp49273_c0_seq1_2, idb_46434	0.01	0.02	0.11	–	0.01	0.05	0.45	0.09	**1.03**	0.49	SSP, IDR; N/Q/S	
**Uncharacterized/similar to putative ferric-chelate reductase 1-like**	**Tri_28544,** **Comp59223_c0_seq1_2**	0.29	0.22	0.01	**2.42**	**1.63**	**1.25**	**–**	**–**	0.03	0.02	SSP, reelin, DOMON, IDR	
**Uncharacterized**	**Tri_111928,** **Comp64272_c0_seq1_3**	0.02	0.03	0.01	0.01	0.05	0.13	0.04	–	**3.84**	**4.43**	SSP	
**Similar to perlustrin**	**Comp70759_c0_seq1_2**	0.31	0.40	**1.39**	–	–	0.30	**1.18**	0.53	**6.04**	**8.71**	SSP, Growth_fac _rcpt/IGFBP; IDR	
**BPTI/Kunitz domain-containing protein (KCP)**	**CLC_148, CLC_77,** **Comp84928_c0_seq1_4**	0.41	0.54	0.45	**2.05**	**2.82**	**3.65**	**–**	**–**	0.72	0.60	SSP, Kunitz_ BPTI; R/C/G/L	[21]
Similar to aragonite protein AP24	CLC_1642, Comp85674_c0_seq1_1, Comp85674_c0_ seq2_1	0.23	0.17	0.19	0.41	0.25	0.34	–	–	0.09	0.10	TM; IDR	[17]
**Similar to endochitinase**	**CLC_4146,** **Comp87152_c0_seq1_4**	0.03	0.06	0.03	–	0.05	0.06	0.01	–	**1.96**	**1.45**	SSP, VWA, chitin-bd_II (2×)	
**Uncharacterized**	**Comp88250_c0_seq2_2**	0.06	0.11	0.04	**6.10**	**2.35**	**1.68**	0.01	–	0.03	0.02	TM; IDR	
**Uncharacterized**	**CLC_12027, idb_54497**	**5.30**	**6.70**	**11.7**	0.21	0.59	0.75	**–**	**–**	0.40	0.09	SSP,TM; IDR, G/M/P; repeats (Additional file 27: Figure S2A**)**	
Similar to tyrosinase	CLC_123, idb_32947	0.05	0.02	0.02	0.81	0.79	0.68	0.03	–	0.14	0.13	SSP; tyrosinase_ Cu-bd, IDR; G; repeats (Additional file 27: Figure S2B)	
Lustrin A (in several fragments)	CLC_1320 etc	0.15	0.41	0.26	0.61	**1.19**	0.77	0.09	0.01	0.04	0.01	SSP, Cys_repeats, IDR; C/P; repeats (PPA)_7_	[13]
Similar to ependymin-related protein 1	CLC_160	–	–	–	–	–	–	–	–	0.73	**3.13**	SSP, ependymin	[21]
Similar to ependymin-related protein 1	CLC_1876	–	–	–	–	–	–	–	–	0.33	**0.58**	Ependymin; L/S	[21]
Similar to glycine-, alanine- and asparagine-rich protein (GAAP)	Tri_107535, CLC_21	0.08	0.07	0.03	0.78	0.47	**2.72**	0.74	0.26	0.53	0.51	IDR; A/G/S; repeats (Additional file 27: Figure S2C)	[21]
Similar to glutamine-rich protein (QRP)	CLC_253	–	–	–	0.80	0.46	0.28	–	–	–	–	IDR; Q; repeats (Additional file 27: Figure S2D)	[21]
Uncharacterized/hasina P0014F12_631	CLC_303	0.09	0.07	0.04	0.64	0.62	**1.14**	0.01	–	0.09	0.08	Chitin-bd_II (3×), ConA-like; IDR, repeats (Additional file 27: Figure S2E)	[22]
**Uncharacterized protein 3 (UP3)**	**CLC_39**	**21.98**	**17.96**	**20.86**	**10.19**	**17.96**	**4.78**	0.13	0.06	0.66	0.75	SSP, IDR; A/L/P; repeats: aa26–52 (GPPPGA[A,V]LR)_3_	[21]
**Similar to cartilage matrix protein/ML7A11**	**CLC_4, Tri_11338**	**5.54**	**6.25**	**8.68**	**5.38**	**6.25**	**10.33**	**–**	**–**	**1.06**	**1.03**	SSP; IDR, N/D/G; repeats (Additional file 27: Figure S2F)	[22]
**Uncharacterized**	**Tri_33510, CLC_62**	0.85	0.87	**1.24**	**4.51**	**5.63**	**6.13**	0.16	0.23	0.04	0.02	SSP; IDP, Q/G/P; repeats (Additional file 27: Figure S2G)	
**Uncharacterized**	**CLC_73, idb_17035,** **Tri_121458**	**6.21**	**7.00**	**6.33**	0.51	0.69	0.66	0.06	0.02	0.41	0.38	IDR; G/P/S; repeats (Additional file 27: Figure S2H)	
Uncharacterized	idb_16318	0.45	0.09	0.28	0.17	0.04	0.08	–	–	–	–	P/V	
**Uncharacterized/similar to mucin**	**idb_18725**	0.03	0.07	0.12	–	–	0.03	**2.26**	0.67	0.84	**1.38**	IDP; A/Q/S/T; repeats (Additional file 27: Figure S2I)	
**Uncharacterized protein 5 (UP5)**	**idb_50884, idb_18,771, idb_18,767**	0.57	0.61	0.12	0.86	**3.79**	**2.69**	0.05	**–**	0.63	0.34	SSP, methyltransf_FA	[21]
**Ependymin-related protein (1)**	**idb_19681**	0.01	0.01	–	–	**–**	**–**	0.02	**–**	**5.30**	**4.89**	SSP, ependymin,	[21]
**Uncharacterized**	**idb_20008**	0.16	0.17	0.45	0.04	0.05	0.15	0.63	0.21	**1.57**	**1.18**	SSP; IDP; Q/G/P; repeats (Additional file 27: Figure S2J)	
Similar to shell protein 4/aplysianin-A	idb_20988	0.01	0.01	0.01	0.22	0.32	0.42	–	–	0.81	0.71	amine_oxidase	[21]
Similar to ependymin-related protein 1	idb_22001	0.10	0.06	0.10	0.02	0.01	0.16	–	–	0.48	**2.80**	Ependymin; T	[21]
**Uncharacterized**	**idb_22086, idb_ 22,087, idb_42421**	0.06	–	0.04	**1.12**	**1.14**	**1.68**	0.01	0.01	0.09	0.06	pI 3.3, IDP, D; repeats (Additional file 27: Figure S2K)	
**Uncharacterized**	**Tri_117880, idb_23862**	0.01	0.03	0.14	–	0.03	0.06	**10.15**	**9.94**	0.48	**1.19**	pI 3.9; IDP, A/S/T; repeats (Additional file 27: Figure S2L)	
**Ependymin-related protein (1)**	**Tri_31898, idb_24481**	0.05	0.06	0.05	–	–	–	0.20	0.03	**7.76**	**3.13**	SSP, ependymin, V	[21]
Uncharacterized	idb_25730	0.05	0.10	0.02	0.21	0.41	0.13	–	–	–	–	VWA, TSP1, chitin-bd_II (2×), ConA_like; G/T; repeats (Additional file 27: Figure S2M)	
**Similar to peroxidase-like**	**idb_25746**	0.11	0.19	0.14	0.84	**1.60**	**2.07**	0.02	0.02	**2.81**	**2.93**	SSP, peroxidase_ 3; IDR	
Uncharacterized/similar to zinc transporter	idb_26030	0.35	0.25	0.05	**1.10**	0.60	0.32	–	–	–	–	SSP, TM, zinc/iron_per-mease, IDR	
Uncharacterized	idb_26568, idb_26567	0.01	0.03	0.07	–	–	0.01	0.44	0.07	0.39	0.25	SSP, IDP, N/Q/P/S; repeats (Additional file 27: Figure S2N)	
Uncharacterized	idb_26836	–	–	0.10	–	–	–	0.94	0.34	0.23	0.33	IDP; S/T; repeats (Additional file 27: Figure S2O)	
**Uncharacterized**	**idb_27355**	0.05	0.11	0.39	0.01	0.01	0.07	**1.93**	**1.81**	**2.91**	**5.54**	SSP, IDP; A/Q/S/T; repeats (Additional file 27: Figure S2P)	
**Uncharacterized**	**idb_27864**	–	–	–	–	–	–	**9.47**	**16.53**	0.57	0.26	pI 4.1, IDP, A/Q/S/T; repeats (Additional file 27: Figure S2Q)	
**Uncharacterized**	**idb_27866**	0.02	0.10	0.11	–	–	–	**19.70**	**22.06**	0.80	0.84	pI 4.5, IDP,A/G/S/T; repeats (Additional file 27: Figure S2R)	
**Uncharacterized**	**idb_32603, idb_32602**	0.02	0.08	0.14	–	–	0.06	**6.05**	**3.21**	**1.81**	**2.05**	SSP; IDP; Q/P/S; repeats (Additional file 27: Figure S2S)	
Uncharacterized protein 2 (UP2)	idb_34528	0.74	0.82	**1.18**	0.09	0.21	0.40	–	–	0.07	0.4	SSP/TM; IDP, A/L/P; repeats	[21]
Uncharacterized	idb_3591	0.59	0.62	0.90	0.23	0.32	0.35	0.01	0.01	0.03	0.01	SSP; IDP, G/S	
**Uncharacterized**	**idb_36583**	0.01	0.04	0.17	–	0.01	0.05	**4.32**	**4.03**	0.99	0.74	IDP, G/S/T; repeats (Additional file 27: Figure S2T)	
Similar to ependymin-1/2	idb_40080	–	–	–	–	–	–	–	–	0.56	0.59	Ependymin; T/V	[21]
Uncharacterized/similar to basic proline-rich protein/methionine-rich protein (MRP; aa162–270)	idb_4071	0.60	0.44	0.50	0.09	0.17	0.18	0.01	–	0.09	0.13	pI 5.1; IDP; Q/P; repeats (Additional file 27: Figure S2U)	
Uncharacterized	idb_43368	0.01	0.02	–	0.40	0.44	**1.53**	0.51	0.23	0.05	0.05	pI 3.3, IDP, A/Q/G/S; repeats (Additional file 27: Figure S2V)	
**Uncharacterized**	**idb_44689**	0.01	0.03	0.12	0.01	0.03	0.09	**7.57**	**5.40**	0.99	**1.36**	IDP, G/S/T; repeats (Additional file 27: Figure S2W)	
**Uncharacterized**	**idb_47306**	0.71	0.86	**3.25**	0.71	0.86	**1.22**	0.24	0.12	0.24	0.18	SSP; IDP, 46 AQ-repeats in C-term, A/Q/G; pI 4.8 (Additional file 27: Figure S2X)	
**Uncharacterized**	**idb_51205**	–	–	–	–	–	–	**17.70**	**21.70**	0.77	0.49	pI 4.0, IDP, repeats; A/Q/S/T (Additional file 27: Figure S2Y)	
Uncharacterized	idb_5218	0.98	**1.38**	0.97	0.33	0.46	0.32	–	–	0.02	0.01		
**Similar to ependymin-related protein (2)**	**idb_52687**	0.01	0.02	–	–	0.02	–	0.05	0.02	**3.06**	**3.94**	Ependymin; T	[21]
Uncharacterized	idb_66139	0.14	0.03	–	**1.06**	0.82	0.34	–	–	0.02	0.06	Repeats: [HQVXL]_2_ in aa56–65	
**Uncharacterized**	**Tri_108584**	**8.64**	**7.60**	**8.24**	**3.32**	**2.35**	**2.00**	0.09	0.07	0.48	0.45	SSP; IDP, P/S; repeats (Additional file 27: Figure S2Z)	
**Uncharacterized protein 4 (UP4)**	**Tri_119193**	**5.84**	**5.43**	**5.58**	**9.74**	**4.28**	**3.58**	**1.62**	0.52	0.22	0.19	TM; A/L	[21]
Uncharacterized	Tri_127820	0.45	0.12	0.16	**2.00**	0.82	0.87	**1.26**	0.32	–	–	SSP; IDR, G/L/P	
Similar to carbonic anhydrase	Tri_130845, idb_813	–	–	–	0.02	0.02	0.01	–	–	0.40	0.50	SSP, αCA_2; Q/G	
**Uncharacterized protein 1 (UP1)**	**Tri_1743**	0.13	0.68	0.92	0.03	0.13	0.33	**1.02**	**1.78**	**12.27**	**16.68**	SSP, IDR; A/Q/L	[21]
**Glycin-rich boundary protein**	**Tri_17455, Tri_2746**	**3.43**	**3.42**	**1.03**	0.83	**3.51**	**2.69**	0.01	**–**	0.25	0.18	SSP/TM; IDR, A/Q; aa176–203 similar to [G-MGA] _7_, aa96–123 [QQQA]_7_	
**Uncharacterized/similar to AP7**	**Tri_24151**	**4.02**	**2.23**	0.24	**1.51**	**1.33**	0.87	**–**	0.02	0.04	0.20	SSP	[17]
**Uncharacterized/similar to shell protein 4/** **ML3D4**	**Tri_25106**	0.07	0.07	0.04	**1.19**	**3.16**	**2.44**	0.02	0.01	**3.35**	**1.71**	SSP	[21]
**Uncharacterized**	**Tri_29101**	0.07	0.05	0.05	0.70	**1.08**	**1.59**	**–**	**–**	0.04	0.02	RmlC-like_jelly_ roll_fold, IDR, A/S/T; repeats (Additional file 27: Figure S2Za)	
**Ependymin-related protein (1)**	**Tri_31892** **Comp22593_c0_seq1_3**	0.50	**1.10**	**2.11**	0.04	0.11	0.42	0.41	0.10	**13.92**	**9.71**	SSP, ependymin,	[21, 103]
**Similar to ependymin-related protein (1)**	**Tri_31897**	0.03	0.01	**–**	–	–	–	–	–	**2.76**	**1.57**	SSP, ependymin	[21]
Uncharacterized	Tri_35519	0.18	0.15	0.09	0.13	0.14	0.17	–	–	0.61	0.48	ConA_like, TM	
Uncharacterized	Tri_45070	0.34	0.40	0.58	0.08	0.12	0.17	0.01	0.01	0.01	0.01	IDP; P/S/T	
**Uncharacterized/similar to molluscan shell protein 1/MSI60-related protein/DGRP/P008C13_381**	**Tri_57798, CLC_5**	0.05	0.14	0.13	**8.38**	**4.38**	**8.64**	**–**	0.83	**1.60**	**1.55**	pI 3.5, IDP; A/D/G; repeats (Additional file 27: Figure S2Zb)	[21, 22]
Uncharacterized/similar to ferric-chelate reductase 1	Tri_61496	0.12	0.12	0.09	0.46	0.29	0.63	–	–	0.17	0.12	Reeler, TM, IDR; S	
Uncharacterized/ similar to putative ferric-chelate reductase 1-like /ML7B12	Tri_63049	0.43	0.58	0.24	0.35	0.34	0.55	–	–	0.02	0.02	Reeler, IDR; T	[22]
Uncharacterized	Tri_64952	0.20	0.01	0.22	**1.47**	0.61	0.68	–	–	0.01	–	IDR; R/G/S	
Carbonic anhydrase	Tri_72839	0.01	–	0.01	0.40	0.21	0.63	–	–	–	0.01	SSP, carbonic_ anhydrase_a; IDR	
**Uncharacterized**	**Tri_73035**	**1.38**	**1.55**	**1.42**	0.77	**1.02**	**1.15**	0.02	–	0.06	0.04	TM; IDP, A/G/P	
Uncharacterized	Tri_81308	0.26	0.32	0.17	0.13	0.16	0.12	–	–	0.02	0.02		
Perlustrin	PLS_HALLA	0.14	0.49	–	–	**2.00**	–	–	–	–	–	IGFBP_N; C	[16]
**Perlucin(s)**	**PLC_HALLA**	**7.44**	**8.12**	0.05	**4.36**	**28.50**	**8.57**	0.03	0.04	**2.40**	0.14	CLECT	[14]
**Perlwapin**	**PWAP_HALLA** **Comp36269_c0_seq1_4**	**3.77**	**4.00**	**2.85**	**1.76**	**2.23**	**1.84**	0.02	**–**	0.80	0.23	WAP; C/G/P	[19, 21]

For more detailed annotations see Additional file 4: Table S2 and Additional file 5: Table S3. *SSP* predicted signal sequence peptide, *TM* predicted transmembrane segment, *IDR* predicted intrinsically disordered sequence regions, *IDP* predicted intrinsically disordered protein (predicted disorder < 90%), *N* nacre, *P* prismatic layer, *S* acid-soluble, *I* acid-insoluble: A, B, C, shell cleaning protocols as detailed in Methods. Amino acids constituting > 10% of the overall amino acid composition are indicated by their standard one-letter abbreviation

^a^calculated from MaxQuant iBAQ intensities; the values are rounded to the second decimal

^b^domain abbreviations are those of InterProScan (http://www.ebi.ac.uk/interpro/)

^c^similar protein previously identified in *Haliotis* shell proteome. A complete list of accepted identifications is contained in tables S2 and S3 (Additional files 4 and 5). The quantitatively most important major proteins (abundance > 1.0 in at least two fractions) and abundance percentages > 1.0 are in bold. Figure S2 is contained in Additional file 27

## Results and discussion

### Isolation of biomineralized organic matrices

**Fig. 1:**
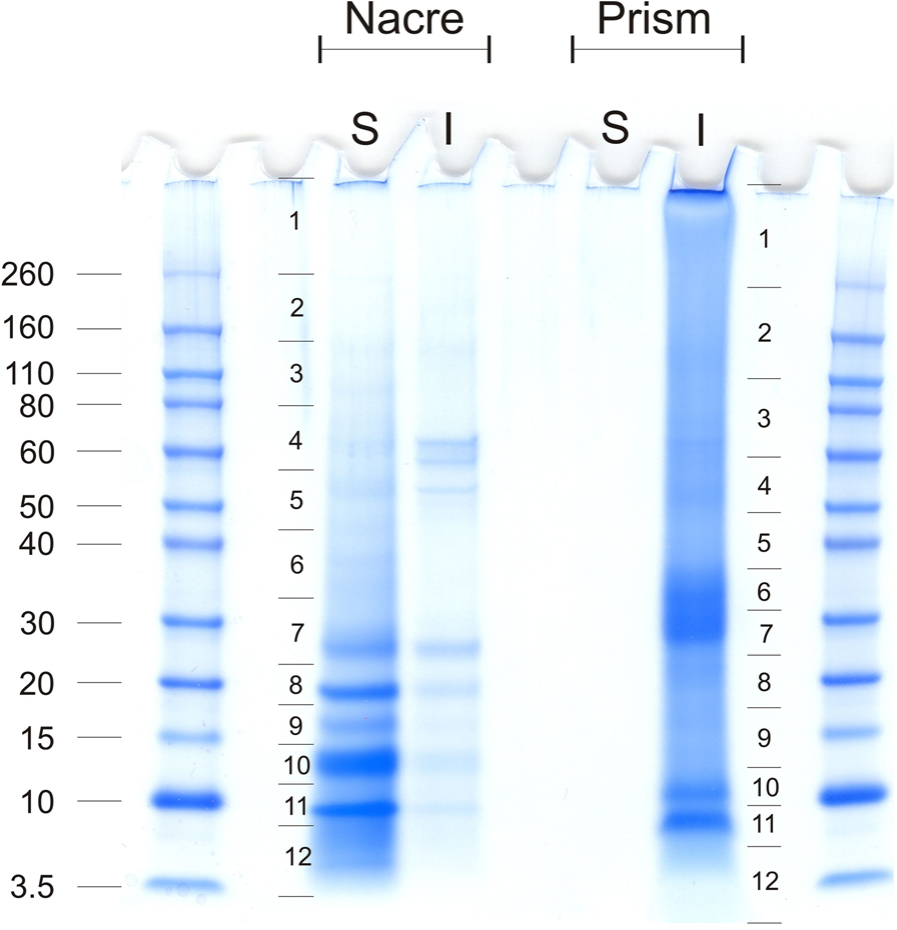
SDS-PAGE separation of nacre and prismatic layer organic matrix proteins. S, acid-soluble; I, acid-insoluble. 200 μg of matrix were applied to each lane. Nacre acid-soluble matrix and prismatic layer acid-insoluble matrix were cut into sections for in-gel digestion as indicated. At the left the masses of marker proteins are shown in kDa (Novex Sharp pre-stained, Invitrogen)

In the literature different protocols can be found that differ in the length of hypochlorite treatment used to clean the biomineral prior to extraction of organic molecules. Here we cleaned most *H. laevigata* shells with sodium hypochlorite prior demineralization to destroy and remove contaminating organic material adhering to the shell surface. However, a treatment lasting 24 h as previously reported [69] visibly damages the nacreous part of the *Haliotis laevigata* shell. Apparently some nacre tablets were detached from the shell and the shell lost some of its lustre and took on a whitish, opaque appearance at the rim. We ascribed this to the partial destruction of the extra-crystalline matrix encasing the typical aragonite tablets of nacre. Therefore the hypochlorite treatment was limited to 2 h (method A) and was combined with short periods of ultrasound treatment with one shell (method B). Possibly different shell types respond differently to hypochlorite treatment, because we did not observe visible damage after 24 h treatment of complete *Lottia gigantea* shells and also did not find major differences in the proteomes extracted after 2 h or 24 h washing [69]. One shell was not treated with hypochlorite at all, but the nacreous layer was sand-blasted from both sides to remove the prismatic layer and upper nacreous layers to obtain pure nacre without any chemical treatment (method C). However, this was not possible with the prismatic layer because it is much thinner and softer than nacre. Therefore only methods A and B were used for the preparation of the prismatic layer. Traditionally the organic shell matrix is separated into acid-soluble and acid-insoluble fractions by centrifugation, and we followed this protocol. Acid-soluble matrix yields were 2-3 mg/g of shell for nacre and 3-7 mg/g for the prismatic layer (Additional file 2: Table S1). Minor, mostly quantitative, differences observed between the SDS-PAGE protein band patterns (Additional file 3: Figure S1) of shell matrices extracted from shells after different cleaning protocols may be due to unintentional technical variations. In contrast, matrices isolated from the different shell layers, nacreous or prismatic, showed very different protein band patterns (Fig. 1 and Additional file 3: Figure S1). Furthermore, we also observed differences between acid-soluble and acid–insoluble fractions (Fig. 1). Differences between nacre acid-soluble and acid-insoluble fractions seemed to be mostly quantitative, while differences between respective prismatic layer fractions were quite dramatic (Fig. 1). Although the yield of matrix in the prismatic layer acid soluble-fraction was much higher than in the acid-insoluble fraction, almost no protein was observed in this fraction indicating that most of the matrix was either not protein or not visible with Coomassie Blue stain (Fig. 1) nor additional silver staining (not shown), or alternatively was not soluble in the denaturing PAGE sample loading buffer. In fact centrifugation of samples after solubilization in PAGE sample buffer produced large insoluble pellets. The recalcitrant nature of many biomineral-associated proteins to standard chromatographic and electrophoretic techniques is well recognized [70] and likely also contributes to the discrepancy we see between acid-soluble and acid-insoluble fractions of the prismatic layer. Acid-soluble fractions of nacre matrix and acid-insoluble fractions of the prismatic layer were separated by SDS-PAGE and in-gel digested. The nacre acid-insoluble matrix and the prismatic layer acid-soluble matrix, which seemed to be less important (as to protein content) were digested in solution using a filter-aided sample preparation (FASP, [29, 30]) technique. All in-gel digested samples were analysed with three technical replicates resulting in a total of 36 raw-files per fraction that were run together in MaxQuant. The in-solution samples were run with five replicates resulting in five raw-files per fraction.

### Comparison of nacre and prismatic layer proteomes

**Fig. 2:**
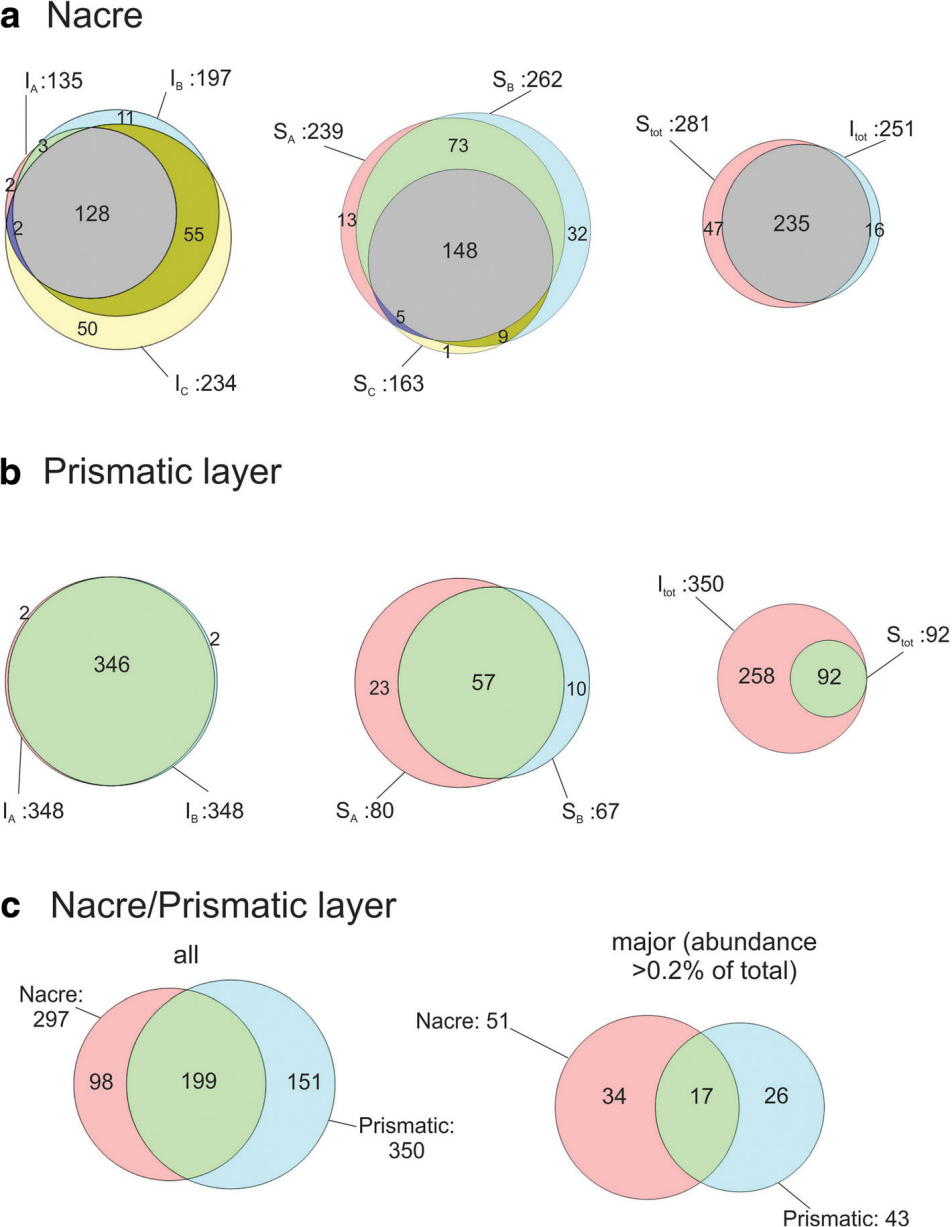
Distribution of *Haliotis laevigata* shell protein between different fractions. Venn diagrams showing the distribution of accepted identifications between the different fractions obtained by extraction of different shell layers with different shell washing protocols. I, acetic acid-insoluble fraction; S, acetic acid-soluble fraction. **a, b** and **c**, shell washing protocols applied before matrix extraction as described in the Material and Methods section. Correctly proportioned two and three circle Venn diagrams were drawn using Venn Diagram Plotter (https://omics.pnl.gov/software/venn-diagram-plotter)

Applying the criteria for acceptance of identifications detailed above in Materials and methods almost 450 proteins were identified (Additional file 4: Table S2; Additional file 5: Table S3). The distribution of proteins between the different fractions obtained with different shell purification methods is shown in Fig. 2. All identifications including those not accepted, for instance single peptide identifications, were retained in the respective MaxQuant output files shown in Additional files 6, 7, 8, 9, 10, 11, 12, 13, 14, and 15 for protein groups. Additional file 16 shows the distribution of nacre and prismatic layer proteins among gel slices. Additional files 17, 18, 19, 20, 21, 22, 23, 24, 25, and 26 show the corresponding identified peptide data f. The numbers of proteins in Fig. 2 and Additional file 4: Tables S2 and Additional file 5: Table S3 should be considered tentative. Thus, some database entries may contain the sequences of several distinct proteins while others may contain only partial sequences of the same protein. We have tentatively combined such fragments into one group as indicated by the differential shading in Additional file 4: Tables S2 and Additional file 5: Table S3. Other proteins have very similar sequences and share most of their peptides. One example of this is the perlucin splice variants detected by cDNA cloning [71]. Because of the sequence similarity and therefore high number of shared peptides we were not able to disentangle and properly quantify the different peptide sets and therefore chose to count these variants as one group. Finally, some proteins may have been missed because of their low abundance, such as perlinhibin and perlinhibin-related protein [20]. These mini-proteins were observed to occur at a very low concentration and their abundance may be too low to be detectable in such a proteomic survey without prior enrichment. Other reasons for missing proteins may be an absence of the respective sequence from the nucleotide databases, or a lack of trypsin cleavage sites. In general, the nacre samples extracted after sodium hypochlorite treatment yielded more proteins and peptides than the samples from shells that were mechanically cleaned, indicating that hypochlorite washing in some way facilitated protein extraction. However, the differences in proteomic results from shells cleaned with different methods were not considered to be meaningful enough to be explored further. Instead we aimed at obtaining a representative shell proteome of *H. laevigata*. As expected from SDS-PAGE results, most of the proteins isolated from nacre without chemical cleaning were found in acid-insoluble fractions (Fig. 2a). With prismatic layer samples, most proteins were identified in the acid-insoluble samples. In fact no protein was identified exclusively in the acid-soluble fractions (Fig.2b).

As previously described [58], we used MaxQuant iBAQ [35, 72] to discern minor and major proteins. The peptide yield of the previously localized extra-crystalline matrix protein lustrin A [12, 13], predominantly identified in the acid-insoluble fractions of nacre, was not particularly affected when the shell was treated with sodium hypochlorite (Additional file 4: Table S2), indicating that our relatively mild washing most probably not did destroy the extra-crystalline matrix to an appreciable extent. We assume that the major proteins are likely to play an important role in shell assembly and shell structure, although minor proteins may of course be important for shell assembly by virtue of enzymatic activities or as part of a signaling network. In the following section we will focus our discussion on the quantitatively major proteins from both *H. laevigata* shell layers (Table 1).

### Major proteins of the *H. laevigata* shell

For the present report we defined major proteins as those that occur in at least two different preparations with an abundance of ≥ 0.2% of the total. However, we did not count those proteins with ≥ 0.2% occurring exclusively in prismatic layer acid-soluble samples, because these samples yielded only very few proteins and apparently did not contain much protein at all (see above) and therefore most likely do not matter quantitatively. In this way we obtained a total of 77 major proteins. This group contained almost all of the proteins previously identified in *Haliotis* shells (Table 1) with the obvious exceptions of UP6_HALAI and UP7_HALAI previously identified in *H. asinina* [21], the *H. laevigata* homologs of which were identified as minor proteins in the prismatic layer and in nacre, respectively (Additional file 4: Table S2; Additional file 5: Table S3). Of the 77 major proteins 34 were categorized as major exclusively in nacre samples and 26 exclusively in prismatic layer samples. Seventeen proteins occurred with the required abundance of ≥ 0.2% in samples from both layers (Fig.2c, right). However, most of the major proteins (86%) could be identified as occurring in both nacre and prismatic layers, but frequently as a minor protein in one of the layers. Only 11 major proteins were identified exclusively in either nacre or prismatic layer (Table 1). One unexpected shell-associated protein was actin with abundances in three fractions just above our threshold for definition as major protein (> 0.2%; Table 1). Cytoskeletal and intracellular housekeeping proteins are frequently identified in biomineral proteomes, and many others than actin also occurred as minor proteins in the proteome of *H. laevigata* (Additional file 4: Tables S2 and Additional file 5: Table S3). When such proteins are identified in shells they are commonly considered to be contaminants as their presence in the shell matrix is difficult to reconcile with current models of shell matrix assembly. We took great care in cleaning the surface of the shells before extraction of the matrix, indicating that these proteins were an integral part of the shell structure and are difficult or impossible to remove without causing damage. This was also shown to be the case with the shell of the brachiopod *Magellania venosa* [62], where we succeeded in significantly reducing the level of intracellular proteins when we treated powdered shell particles for 24 h with hypochlorite, but also lost some interesting proteins with some features characteristic of shell proteins, probably by removal of a large part of the extra-crystalline matrix. In mammals, intracellular proteins like the cytoskeletal component actin have been found at the cell surface, in extracellular matrices, and in body fluids (reviewed in [73]). The source of these proteins remains essentially unknown, but one suspected origin is from damaged or stressed cells. Currently it remains unknown whether such proteins are inadvertently occluded into the growing edge of the biomineral, or whether they genuinely play a functional role in biomineralisation. One piece of information that links the cytoskeleton to the process of shell formation comes from an unusual chitin synthase gene isolated from the marine bivalve *Atrina rigida* [74] that contains a myosin head domain that may interact with the actin cytoskeleton, thus providing a link between a component of the shell-forming machinery and the cytoskeleton [75].

Nacreous and prismatic layers have been separately analyzed in species other than *Haliotis* previously. The shell of the pearl oyster *Pinctada* [23] yielded a total of 80 identified proteins. Forty-seven of these were apparently prism specific and 30 were nacre specific. Only three were identified in both compartments. More overlap was found in the different compartments of *Mytilus* shells. Nacre, fibrous prism and myostracum layers of *M. coruscus* [24] yielded a total of 63 proteins with 16 nacre specific proteins, 14 fibrous prism specific proteins, and eight myostracum specific proteins. Twelve proteins were shared by all three compartments, eight by nacre and myostracum, and five by nacre and fibrous prism layers. *Mytilus galloprovincialis* provided similar distributions with a total of 113 identified proteins [25]. The total numbers of identified proteins were similar to the number of major proteins we identify in the present report. However, no abundance estimates were provided for *Pinctada* or *Mytilus* proteins.

The list of major proteins contains some very acidic proteins and many proteins predicted to contain intrinsically disordered regions (IDR) or to be intrinsically disordered proteins (IDP) altogether. Both properties are thought to play an important functional role and have attracted much attention. Some of the first characterized proteins of biomineral organic matrices were unusually acidic with calculated isoelectric points close to four due to a high proportion of aspartic acid in their sequences. Early examples include MSP-1 from the shell of the scallop *Patinopecten yessoensis* [76, 77], prismalin-14 from the prismatic layer of the oyster *Pinctada fucata* [78], and aspein also from *Pinctada fucata* [79]. Because of their possible ability to bind calcium ions in solution and on crystal surfaces by electrostatic interaction, acidic proteins were suggested to control crystal nucleation or crystal growth regulation [80] and became a major topic of biomineralization research [81]. However, it soon became apparent that biomineral matrices did not only contain acidic proteins but also neutral and basic ones [82]. Furthermore, many of these proteins displayed biased amino acid compositions with high percentages of certain amino acids, most often Ala, Gly, Gln, Ser and Pro. Frequently these amino acids occurred in uninterrupted blocks or in short repetitive sequence stretches [83]. An early example of such a shell protein was MSI60 from the oyster *Pinctada fucata* that consisted to 26% of Ala and 37% of Gly [84]. Biomineral matrix proteins with large stretches of simple repeats comprise, for instance, nacrein of the gastropod *Turbo marmoratus* [85], or pearlin from *Pinctada margeritifera* [86] that contain extended blocks of Gly-Asn repeats. Proteins or protein regions with such characteristics frequently do not have a three-dimensional structure under native conditions and belong to the widespread group of intrinsically disordered proteins [87–89]. IDPs and IDRs apparently also occur frequently in biomineral matrix proteins [90–92] therefore prediction of disorder was added to the annotations in Additional file 4: Tables S2 and Additional file 5: Table S3.

### Major proteins previously known as *Haliotis* shell components

**Fig. 3:**
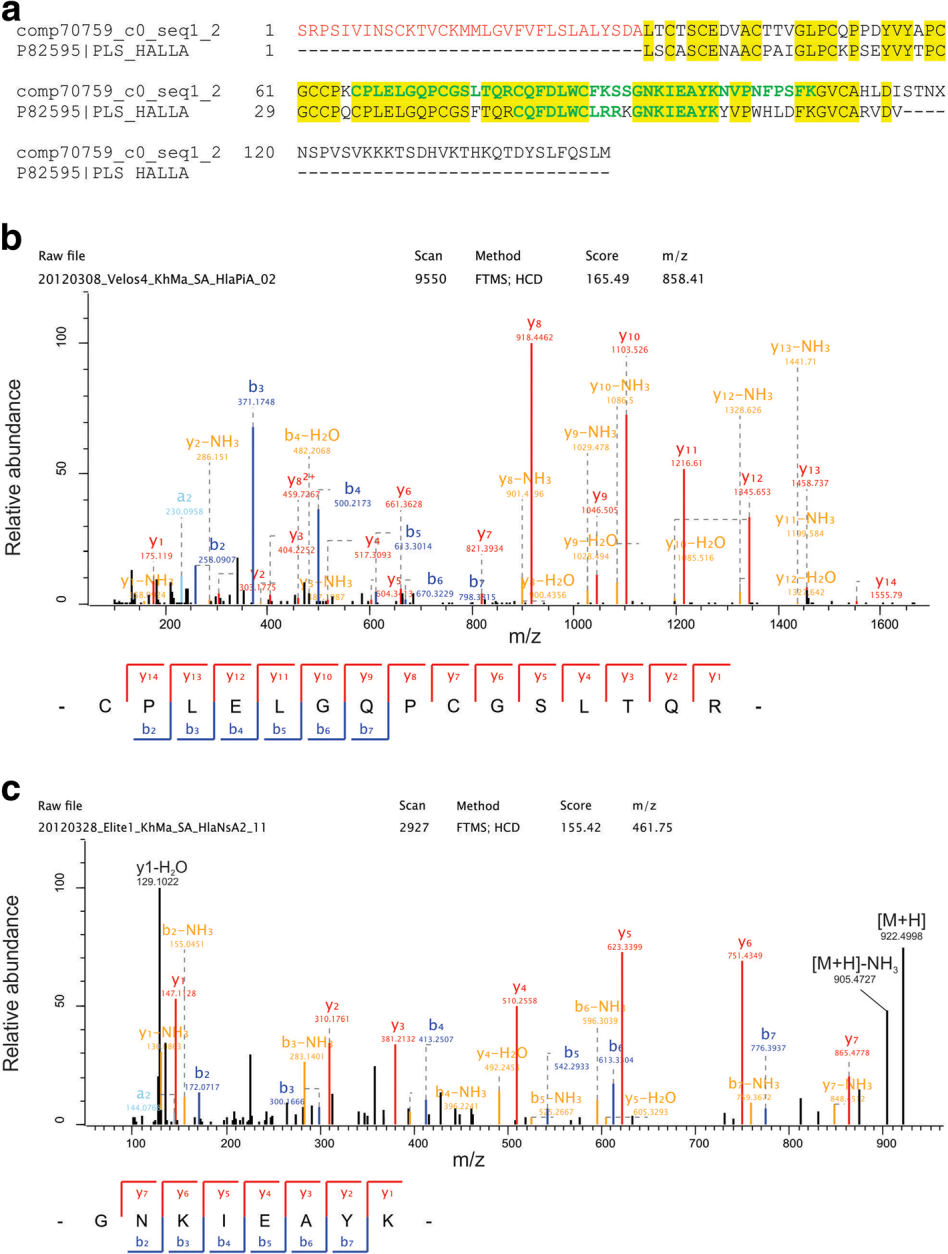
Perlustrin alignment and spectra. **a** Alignment of a nacre protein 70% identical to mature *H. laevigata* perlustrin isolated from nacre matrix and sequenced on the protein level using automated Edman chemistry [16]. A predicted signal sequence peptide is in red. Sequence regions confirmed by MS/MS-derived peptide sequences are in green. **b** MS/MS spectrum of a selected sequence-unique peptide of comp70759_c0_seq1_2. This peptide of a mass of 1714.8131 Da was identified with a Posterior Error Probability (PEP) of 5.2e-19 and a mass error of 0.3 ppm. **c** MS/MS spectrum of a selected sequence-unique peptide of P82595. This peptide showing one miss-cleavage was identified with a PEP of 0.019 and a mass error of 0.3 ppm. Y-ions are shown in red, b-ions are in blue, and fragments with neutral loss are in orange. A few fragment non-standard but advanced annotations with the help of the MaxQuant Expert system [44] are shown in black. For the sake of clarity most advanced annotations are not shown. The mass spectrometer model used, Velos or Elite, is contained in the raw-file name on top of the y-axis of the spectra

Most of the proteins previously identified in *Haliotis* shells were identified in this proteomic survey above the threshold set for major proteins. The *H. laevigata* C-type lectin perlucin [14] and the WAP domain–containing perlwapin [19] were among the most abundant proteins of the nacreous layer (Table 1). Both proteins were shown to modulate calcium carbonate nucleation and crystal growth in vitro [15, 19, 93]. Perlucin was recently shown to occur in several splice variants [71]. In this survey we indeed found evidence for several perlucins (Additional file 4: Table S2; more than 60 when we perform a tBLASTn search against the assembled transcriptome). However, the sequences of these variants were so similar that most of them shared most of their peptides and we were not able to quantify them properly. Consequently they were treated as one protein in this report. The small *H. laevigata* nacre IGF-binding protein perlustrin [16] was found with lower and variable abundance (Table 1). Interestingly, the *H. laevigata* shell contained another very similar protein (about 70% identical to PLS_HALLA in overlapping sequence regions; Additional file 4: Table S2; Fig. 3), which was found in the tentacle/hemolymph-derived database [38] and was identified as a major protein in both, nacre and prismatic layer. These perlustrins did not share peptides, but each amino acid sequence was validated by proper MS/MS-sequences (Fig. 3). Interestingly, hemocytes were previously shown to contribute to shell mineralization and repair in *Crassostrea virginica* and *Pinctada fucata* [94, 95] and to be present in the extrapallial fluid [95].

Fragments of the long extra-crystalline matrix protein lustrin A were identified in several transcriptome database entries (Additional file 4: Table S2). The leading entries CLC_1320, Tri_116352, idb_288 and CLC_608 were on average 74% identical to *H. rufescens* (O44341_HALRU; [13]) and *H. tuberculata* (A0A088CBA1_HALTU, F6KD05_HALTU; [96]) sequences and covered approximately 70% of the sequence of O44341_HALRU, apparently the most complete lustrin A. In agreement with this we previously reported great difficulty in assembling a complete lustrin from NGS data, most probably due to the repetitive architecture of this protein [6].

**Fig. 4:**
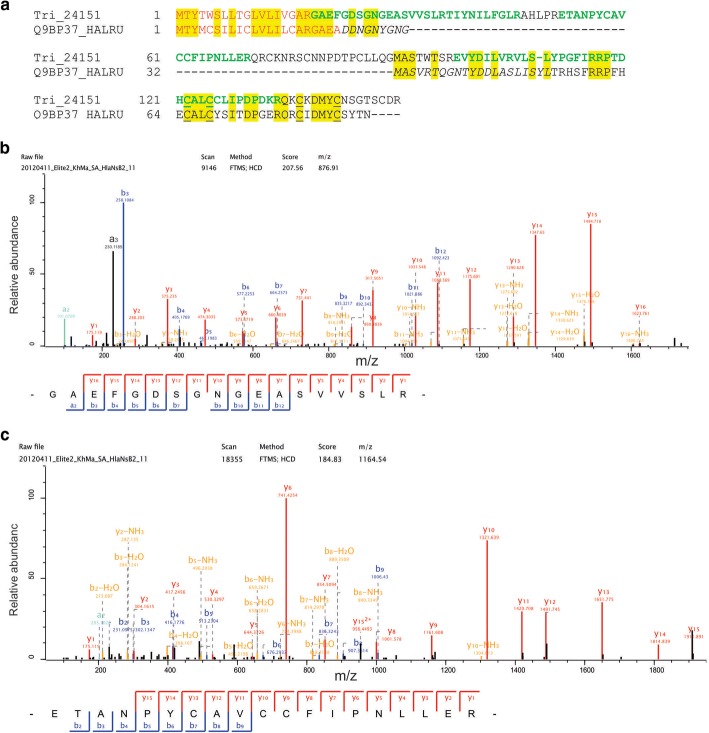
AP7 alignment and spectra, **a** Alignment of *H. laevigata* Tri_24151 to *H. rufescens* AP7 (Q9BP37_HALRU; [17]). Predicted signal sequence peptides are in red. Sequence regions confirmed by MS/MS-derived peptide sequences are in green. Cysteines proposed to be part of the metal binding site [98] are underlined. The N-terminal mineral-interacting domain [97] is shown in italics. **b** MS/MS spectrum of a selected sequence-unique peptide most probably representing the N-terminus of this protein and confirming the secretion signal peptide prediction. This doubly charged peptide was identified with a mass error of 0.5 ppm and a Posterior Error Probability (PEP) of 1.7e-42. Y-ions are shown in red, b-ions are in blue, and fragments with neutral loss are in orange. Ion a3 was identified using the advanced annotation option of the MaxQuant viewer (Expert system [44]). **c** MS/MS spectrum of a selected sequence-unique peptide from the insert sequence region not present in *H. rufescens* AP7. The doubly charged peptide was identified with a mass error of 0.01 ppm and a PEP of 2.7e-36. The mass spectrometer model used, Velos or Elite, is contained in the raw-file name on top of the y-axis of the spectra

Entry Tri_24151 possibly contained the sequence of a *H. laevigata* counterpart of *H. rufescens* AP7 [17] (Fig. 4). *H. rufescens* AP7 (Q9BP37_HALRU) was shown to consist of two small domains, a calcium-binding N-terminus [17, 97] following the secretion signal peptide, and a C-terminal C-RING-like domain [98], which was found to participate in in vitro protein-protein interactions, self-assembly, and mineral nucleation [99, 100]. The sequence identity of Tri_24151 to Q9BP37_HALRU was only 43.5% and the e-value (0.0005) was relatively high (Additional file 4: Table S2). The four cysteines probably taking part in multivalent metal ion binding [98] were preserved in the *H. laevigata* sequence (Fig. 4). However the N-terminal domain of AP7 was disrupted in Tri_24151 by a 58aa-long insertion. The predicted N-terminus of the mature protein, the insert, the N-terminal domain and the C-terminal domain of the presumptive translation product were confirmed by MS/MS-derived peptide sequences (Fig. 4). AP7 was shown to be at least partially disordered [101], a feature that was not predicted for Tri_24151 by neither MFDp2 nor two other disorder prediction programs (IUPred and PrDos). Protein Tri_24151 was identified with very high abundance (> 1.0%) in samples of hypochlorite-treated nacre, with lower abundance in untreated nacre and even less in prismatic layer samples (Table 1). In the original report the column fraction containing AP7 contained another nacre protein, AP24 [17]. A very similar protein (77.8% identity; Additional file 4: Table S2) was contained in entry CLC_1642. As for the N-terminal 30aa of AP7, the N-terminus of AP24 was shown by NMR to be disordered [102], but again this feature was not predicted by the prediction software programs we used. Instead, the region between aa152–176 was predicted to be disordered. AP24 was identified in nacre, but at a much lower abundance than AP7 (Table 1).

The first proteomic analyses of *Haliotis* shell matrices [21, 22] yielded 17 and 7 proteins, respectively, including perlwapin and three tentatively identified proteins. The *H. asinina* shell [21] contained seven proteins essentially without predicted domain structure, the recommended name of which in the UniProtKB database is uncharacterized protein (UP) 1 to 7. In the present report we will also use this name (Tables 1, Additional file 4: Table S2 and Additional file 5: Table S3). UP3 (CLC_39) and UP4 (Tri_119193) were among the most abundant proteins in nacre with abundances of > 1.0% in all nacre samples (Table 1). UP1 (Tri_1743) was a major protein in all prismatic layer samples (Table 1). UP2 (idb_34528) and UP5 (idb_50885/18771/18767) were less abundant, but still major proteins predominantly identified in nacre. UP6 (idb_59441 and idb_27788) and UP7 (Tri_100716) did not comply with our thresholds for major proteins but were identified and classified as minor proteins (see below). The average sequence identitity between *H. asinina* UPs and their *H. lavigata* equivalents was 80–81%.

Another important group of *H. asinina* prismatic layer matrix components were two ependymin-related proteins, EDPR 1 (ML1E6) and EDPR 2 (6G3) [21]. In *H. laevigata* we identified many entries containing predicted ependidym domains (Additional file 4: Table S2, Additional file 5: Table S3), many of them sharing peptides. The table of major proteins (Table 1) contains nine ependymin-related entries. Besides shared peptides many of them contained sequence unique peptides often located at identical positions of alignments to EDPR 1 and 2. From our data it was difficult to decide whether these were independent but related gene products or variants of a particular protein. The entries most similar to the *H. asinina* [21] proteins were Tri_31892 for EDPR1 with 84.3% identity and CLC_1876 for EDPR2 with 64.0% identity. However, in FASTA searches against the UniProtKB database EDPR1 was the highest scoring match in both cases. The best match for EDPR2 in FASTA searches was idb_52687 with 57.6% identity (Additional file 5: Table S3). As reported for EDPR1 and 2 in *H. asinina* [21], these proteins were most abundant in prismatic layer samples and were either not identified not at all or only in negligible amounts in nacre. The only exception was Tri_31892/comp22593_c0_seq1_3 that was also a major protein in nacre (Table 1). Tri_31892 was also similar to an ependymin-like protein extracted from the nacre organic matrix of *H. diversicolor* (AEP 25 kDa; [103]).

**Fig. 5:**
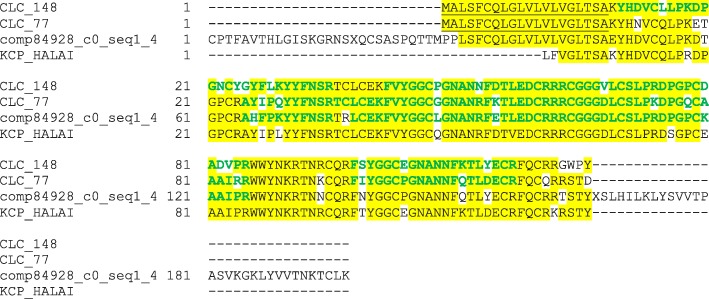
Sequence alignment of KCP_HALAI to related major *H. laevigata* sequences. Predicted signal sequence peptides are underlined. Sequence regions confirmed by identified peptides are shown in green

Other major proteins previously identified in the shell of *H.asinina* [21] were KCP_HALAI (P0012N13_463), GAAP_HALAI (HasCL10contig2), QRP_HALAI (ML8B1), and DGRP_HALAI (P0025F23_658), which were also among the major proteins of the *H. laevigata* shell matrix (Table 1). Sequences with approximately 83% sequence identity to the BPTI/Kunitz domain-containing protein KCP were identified in CLC_148, CLC_77 and Comp84928_c0_seq1_4. Despite some sequence differences confirmed in part by MS/MS-sequenced peptides (Additional file 4: Table S2) these proteins were so similar to each other and KCP that we chose to treat them as variants of one protein (Fig. 5), and were identified as major proteins in both shell layers. However, as in all such cases encountered, these entries could of course also represent different gene products. Glycine-, alanine- and asparagine-rich protein (GAAP) was contained in *H. laevigata* sequence database entries Tri_107535/CLC_21 with 77.5% sequence identity. This protein was also identified as a major protein in both shell layers (Table 1). A sequence with 57.1% identity to glutamine-rich protein (QRP_HALAI) was identified in the C-terminal half of entry CLC_253 (Additional file 27: Figure S2D). In contrast to [21] we identified this protein only in the acid-insoluble fractions of nacre. Possibly this difference was due to different centrifugation procedures. While we used ultracentrifugation, Marie et al. [21] used centrifugation at 3900 g to sediment acid-insoluble matrix. However, sedimentation by ultracentrifugation would require some kind of aggregation with itself or other matrix components. A protein with 73.9% identity to aspartate- and glycine-rich protein of *H. asinina* (DGRP_HALAI) was detected in the C-terminal half of entry Tri_57798 (Additional file 4: Table S2). The N-terminal half of this entry was most similar to part of the MSI60-related protein of *Pinctada fucata* (46.4% identity to G9MD31_PINFU; [84]) and the entire entry was 32.2% identical to molluscan shell protein 1 (MSP-1) of *Mizuhopecten yessoensis* (Q95YF6_MIZYE; [76, 77]). Of the tentatively (with a single unique peptide) identified proteins of Marie et al. [21], ML3D4 was similar to Tri_25106 and idb_20988 (Table 1, Additional file 4: Table S2). These in turn were similar to a putative amine oxidase identified in the shell proteome of *Mytilus coruscus* (A0A0G2YN89_MYTCO; [24]).

A pilot study of the *H. tuberculata* shell proteome [22] contained four new proteins not identified in *Haliotis* shells before. The protein similar to hasinaP0014F12_631 was similar to aa615–745 of entry CLC_303 (Table 1, Additional file 4: Table S2), similar to ML7B12 was similar to Tri_63049 (Table 1, Additional file 4: Table S2), similar to hasinaP008C13_381 was similar to aa94–216 of Tri_57798 (Table 1, Additional file 4: Table S2), and similar to ML7A11 was similar to aa24–244 of Tri_11338 (Table 1, Additional file 4: Table S2).

### Major proteins previously detected in transcriptomic studies of *Haliotis* mantle tissue

Carbonic anhydrases (CA) catalyze the formation of hydrogen carbonate from CO_2_ and H_2_O. This is an extremely important reaction for calcium carbonate biomineral-forming organisms and the enzyme(s) are therefore almost ubiquitous [104]. Many molluscs produce α-carbonic anhydrases in the mantle tissue and often these enzymes are recovered from biomineral matrices, for instance in *Lottia gigantea* [69, 105]. To date no carbonic anhydrase protein has been identified in a haliotid although mRNAs coding for two predicted CAs were identified in the mantle transcriptome of *Haliotis tuberculata* [106]. However, proteomic analysis and enzyme activity assays failed to reveal the presence of carbonic anhydrase in the matrix [106]. One of the putative α-CA proteins was predicted to be a secreted protein (htCA1), the second one was predicted to be a transmembrane protein (htCA2) [106]. We have identified two CAs among the major proteins of *Haliotis laevigata* shell matrix (Table 1). One of them, Tri_72839, was present predominantly in acid-insoluble nacre samples while the second one, Tri_130845/idb_813, was present almost exclusively in acid-insoluble prismatic layer samples. Both were predicted to be secreted (Table 1, Additional file 4: Table S2, Additional file 5: Table S3). The nacre enzyme, Tri_72839, was 78.5% identical to htCA2/G0YY03_HALTU of [106]. The prismatic layer enzyme, Tri_130845/idb_813, was most similar to the *Patella vulgaris* putative CA (J7QJT8_PATVU; [107]), however only with 31.2% identity (Additional file 5: Table S3). No sequence similar to htCA1 was identified in the present study.

A glycine-rich putative secreted shell protein derived from the mantle transcriptome of *H. asinina* and termed glycine-rich boundary protein (A0A0B4VCR4_HALAI; submitted by McDougall C, Woodcroft B, Degnan B; 2014), was similar to Tri_17455 (Additional file 4: Table S2) and was not only rich in glycine but also in alanine, glutamine and methionine. About 64.6% of the sequence was predicted to be disordered. This protein was found to be one of the major *H. laevigata* nacre matrix proteins with an abundance > 1.0% in five out of six fractions (Table 1).

### Major proteins not previously identified in *Haliotis* shell proteomes

The *H. laevigata* shell proteome also contained proteins not previously identified in *Haliotis* shells. However, these were predicted to contain domains or other features encountered previously in other mollusc shell proteins. Entries CLC_123/idb_32947 contained the sequence of a predicted tyrosinase. Messages coding for tyrosinase-like proteins have been detected in molluscan mantle transcriptomes and shells [108–110] and may be involved in shell protein cross-linking, especially in the periostracum. Tyrosinases may also play a role in shell coloration [111]. In addition to the predicted tyrosinase domain in aa18–271, this entry also contained a short stretch of collagen triple-helical repeats in aa336–354 and the predicted disordered structure of the C-terminus consisted essentially of G-rich tandem repeats (Additional file 27: Figure S2B). Participation in cross-linking of matrix proteins has also been suggested for peroxidase-like proteins [112] identified in mollusc shell proteomes [56, 105]. The putative peroxidase contained in entry idb_25746 was a very abundant component of the acid-insoluble fractions of both the nacreous and prismatic layers (Table 1). The uncharacterized proteins with similarity to ferric-chelate reductase-like proteins in entries Tri_28544/Comp59223_c0_seq1_2 and Tri_61496 (Table 1) may also be involved in some kind of redox reaction important for shell protein cross-linking as suggested previously [113]. The former contained a predicted DOMON domain typically found in dopamine β-monooxygenase/hydroxylase and a reelin domain. This protein was very abundant in acid-insoluble fractions of nacre while Tri_61496 was much less abundant and contained only a predicted reelin domain. Both proteins were predicted to contain disordered sequence regions (Additional file 4: Table S2). Mollusc shells are known to contain chitin, which contributes to the insoluble fraction of the shell matrix [114]. Consequently most mollusc shell proteomes also contain proteins with chitin-binding and/or chitin-modifying domains. These proteins are likely to participate in chitin metabolism or to mediate between an insoluble chitin scaffold and functionally important soluble matrix proteins. The major proteins predicted to contain chitin-functionality (Table 1) were only a fraction of the total number of *H. laevigata* predicted chitin-binding shell matrix proteins identified (Additional file 4: Table S2 and Additional file 5: Table S3). CLC_4146/Comp87152_c0_seq1_4 was identified with very high abundance in the acid-insoluble prismatic layer samples while idb_25730/Comp68740_c0_seq1_1 was identified at a much lower abundance in nacre only. Both proteins contained in addition to the chitin-binding domain a von Willebrand A domain, a combination that is also known from shell matrix proteins Pif and BMSP [115–117].

More than half of the entries in the list of major proteins did not contain predicted domains. Frequently the respective protein sequences displayed biased amino acid compositions (Table 1) and the respective amino acids (frequently D, Q, A, S or P) were often organized in repetitive short motifs or longer sequence blocks of a few particular amino acids. Most of these proteins were predicted to be disordered and frequently they were very acidic. Repeats, together with their corresponding complete sequences are presented in Figure S2 (Additional file 27) and reference to sequences and their repeats is included into the second last column of Table 1. These kinds of distinctive features have also been observed in bivalve shell matrix proteins and other invertebrate biomineral matrix proteins [81, 82, 116, 118–120]. However, database searches with these uncharacterized *H. laevigata* proteins resulted either in no convincing match or matches based on particular amino acid composition features, such as extremely high aparagine or glycine content. This raises the question whether such proteins share true evolutionary homology. Previous comparisons between the mantle transcriptomes of the nacre-forming gastropod *H. asinina* and the nacre-forming bivalve *Pinctada maxima* indicated that proteins with such features, frequently called repetitive, low-complexity domains (RLCDs) are not related and are likely to be the result of convergent evolution [121]. However, between species of one genus such proteins are thought to have evolved rapidly [120, 121]. The independent evolution of these proteins in different invertebrate classes implies that these sequences possibly embody common principles required for shell building. Table 1 contains several entries with very acidic isoelectric point (3.3–4.5). In all cases these sequences were predicted to be intrinsically disordered and contained tandem repeats of various lengths. However, only in two cases strongly acidic isoelectric point coincided with high concentration of aspartic acid (idb_22086 and Tri_57798, 36 and 25% D, respectively; Additional file 4: Table S2). Both proteins were still far away from such extreme aspartic acid accumulations as observed in bivalve aspein [79, 122] or asprich [123] with up to 75% aspartic acid. Entry Tri_57798 contained in the N-terminus an almost uninterrupted stretch of 55 aspartic acid residues, very much similar to the more extended D blocks in some bivalve proteins, in addition to short D-rich repeats (Additional file 27: Figure S2Zb). In idb_22086 and some related sequences aspartic acids were much more evenly distributed along the sequence and its repeats (Additional file 27: Figure S2K). Idb_22086 and 22,087 were identical up to aa309 and shared many peptides. The C-terminal sequences however were not related. In contrast, the N-terminal half of the much shorter sequence of idb_42421 aligned to a region in the C-terminus of idb_22086 (Additional file 27: Figure 2SK). The exact relationship between these three entries is not clear at present. The sequences could be those of distinct, but related proteins, or fragments of one or two proteins. All three contain many tandem repeats. For the time being we have preferred to put them into one group. A Q-rich protein other than the previously identified QRP (CLC_253) was contained in Tri_33510/CLC_62 (Additional file 27: Figure S2G). This very abundant nacre protein was predicted to be intrinsically disordered. The glutamines occurred in blocks of up to 10 Q in the C-terminal half of the sequence. The glutamine-, glycine- and proline-rich secreted intrinsically disordered prismatic layer protein of idb_20008 (Table 1) contained an almost uninterrupted sequence of 24 glutamines in aa80–104. In addition the sequence was full of short tandem sequence repeats of between 5 and 16 amino acids, the most numerous being 13 repeats of the type GMGNPM/TX in aa287–377 and some Q/P-rich repeats in aa470–573 (Additional file 27: Figure S2J). Other proteins contained stretches of very simple short repeats in tandem, such as [GN]_n_ or [AQ]_n_. GN (or NG) tandem repeats as in CLC_4/Tri_11338 (Additional file 27: Figure S2E) and CLC_5/Tri_57798 (Additional file 27: Figure S2Zb), or related repeats, such as [GNN]_n_, were also found in the bivalve shell proteins nacrein [85], pearlin [86], N66 and N14 [124]. Extended stretches of [AQ] and [AA] were found in CLC_303 (Additional file 27: Figure S2E) and idb_47306 (Additional file 27: Figure S2X). Proteins idb_54497/CLC_12027 contained in their predicted disordered region following the secretion signal peptide several G/M-rich repeats built around the motif [GMPG/MX_n_] (Additional file 27: Figure S2A). Overlapping sequences of entries CLC_73, idb_17035 and Tri_121458 (Additional file 27: Figure S2H) may be variants of one protein and were treated as such (Additional file 4: Table S2) although they also contained confirmed sequence-unique peptides at conflicting locations. However, all three entries also shared peptides and had very similar features as, for instance basic pI, high concentrations of serine, and predicted disordered structures. A distinctive feature of entry CLC_73 was a long N-terminal collagen triple-helical domain that was lacking in the shorter entries. This protein also contained in its sequence Ser-rich and tandem repeats. More sequences with tandem repeat structures are contained in Additional file 27: Figure S2 as cross-referenced in Table 1. All of these features are not new but occur identically or similarly in many other biomineralising proteins [91, 92, 125–127].

### Minor proteins of potential importance

Although we assume that the most abundant proteins represent those of greatest functional significance, less abundant proteins can of course also have an impact if enzymatically active or form part of a signaling cascade. For this reason we focus on a few minor proteins of potential interest.

In addition to the major peroxidase-like idb_25746 we identified several other possible peroxidase/peroxidasin-like proteins which were contained in entries Comp51700_c0_seq3_3, idb_19812/idb_19814, and Tri_4200 (Additional file 4: Table S2 and Additional file 5: Table S3). Furthermore, entry idb_40380/Comp89520_c0_seq1_4 contained the sequence of a predicted superoxide dismutase. Superoxide dismutases are a family of enzymes with widespread subcellular distribution that remove superoxide, a normal aerobic metabolite that is also a substrate of peroxidases. Peroxidases have been implicated previously in mollusc shell formation [112]. Possibly they are responsible for the sclerotization of the periostracum [128–130], the proteinaceous layer confining the mantle cavity before the start of mineralization. As discussed previously [21, 56] one may hypothesize that peroxidases function in stabilization of the newly secreted matrix by cross-linking some of its components. Although the highest scoring match in FASTA database searches for idb_19812 and idb_25746 was a *Lottia gigantea* sequence (Additional file 4: Table S2), this was not one of the peroxidases identified as major proteins in the *L. gigantea* shell. In addition to the major carbonic anhydrases in Tri_130845/idb_813 and Tri_72839 the *H. laevigata* shell prismatic layer contained several minor proteins predicted to be carbonic anhydrases because of their sequence similarity to other molluscan CAs and predicted CA domains. However, these proteins (Comp97413_c0_seq7_1/ idb_58049, Tri_119238, Tri_6552) were all of very low abundance (Additional file 5: Table S3). Metalloproteases, enzymes that were abundant in sea urchin biomineralized structures [131] were found predominantly in the insoluble fraction of the *H. laevigata* prismatic shell layer at low abundance (Additional file 5: Table S3; CLC_3466, idb_18707, idb_20328).

As briefly discussed above, chitin is a key component of mollusc shells. Thus all proteins and enzymes binding to chitin may be of potential importance for shell assembly. In addition to the major chitin-binding proteins in Table 1 we have identified many minor proteins predicted to bind chitin or related domains (summarized in Table 2). For most of these minor proteins the best matches, that is, the highest scoring hits appearing in the first line of the FASTP output, were molluscan proteins (Additional file 4: Table S2, Additional file 5: Table S3), the sequences of which were from genome sequencing projects of the limpet *Lottia gigantea* [132] and the oyster *Crassostrea gigas* [133]. Rarely the sequences were from single gene cloning experiments, as for instance, the chitin metabolic enzyme genes of the freshwater mussel *Hyriopsis cumingii* ([134]; J7FHX7 and J7F1C1, Additional file 4: Tables S2 and Additional file 5: Table S3), and even more rarely a protein was identified in a shell proteomic study, as for instance, PSM_MYTCA [135]. The percentage of conserved residues between the species was rarely more than 40%.

**Table 2:** Low-abundance proteins predicted to be related to chitin binding and modification

Protein	Accession no.	Predicted domains	Shell layer
**Similar to chitinase-3**	**Comp79626_c0_seq1_4, idb_43266**	**SSP; chitinase_II, chitin-bd_II**	**N, P**
**Similar to chitin-binding protein**	**CLC_1125**	**SSP/TM; Cellulose/chitin-bd_N**	**N, P**
Uncharacterized	CLC_18633	Chitin-bd_N; TM	N, P
Similar to chitinase-3	CLC_2296	SSP; chitinase_II, chitin-bd_II (2×)	N, P
Uncharacterized	CLC_2347, idb_28940	ARM_like, chitin-bd_II (2×); ConA_like	N
Uncharacterized/IgGFc-binding protein	CLC_3878, idb_2768, idb_2772, Tri_120377, Tri_120379	SSP; chitin-bd_II (4×), Sushi, galectin_CRP, FA58C_3	N, P
Similar to shell matrix protein (PSM_MYTCA**)**	idb_13357 (aa561–780), idb_13358	chitin-binding_II (2×); IDP	N, P
Similar to IgGFc-binding protein	idb_1745	SSP; chitin-bd_II (23×)	N, P
Uncharacterized	idb_2023, CLC_2607, idb_2021	IG, chitin-bd_II	N, P
Similar to chitinase-3	idb_32310	SSP; chitinase_II, chitin-bd_II (2×)	N, P
Uncharacterized	idb_44571	chitin-bd_II (4×); TM	N, P
Similar to endochitinase	idb_53451	glyco_hydro_18, chitin-bd_II	N
Similar to chitin deacetylase	idb_6290	SSP; glyco_hydro/deAcase_b/a-brl/NodB (2×)	N, P
Uncharacterized	idb_982	SSP**;** multiple Sushi_SCR_CCP, galactose_bd, chitin-bd_II (6×), fucolectin/tachylectin-4/pentraxin-1, galectin	N; P
Uncharacterized	Tri_109450	SSP; chitin-bd_II (2×)	N, P
Uncharacterized	Tri_7902	chitin-bd_II (3×)	N, P
Uncharacterized	idb_54309, Comp22563_c0_seq1_3, idb_57746	SSP, chitin-bd_II (3×)	P
Uncharacterized	Comp99505_c0_seq1_5	TM; chitinase_II	P
Uncharacterized	CLC_413	chitinase_II	P
Uncharacterized	idb_32090	TM; chitin-bd_II (3×)	P
Uncharacterized	idb_5844	TM; SEA, chitin-bd_II (3×), Ig-like_fold	P
Uncharacterized	Tri_50040	SSP; ConA-like, chitin-bd	P
Uncharacterized	Tri_95672	SSP; ConA-like,, chitin-bd_II (3×)	P

For more detailed annotations see additional Additional file 4: Table S2 and Additional file 5: Table S3. *SSP* predicted signal sequence peptide, *TM* predicted transmembrane segment, *IDP* predicted intrinsically disordered protein (predicted disorder > 90%), *N* nacre, *P* prismatic layer. Domain abbreviations are those of InterProScan (http://www.ebi.ac.uk/interpro/). The two first entries were close to the threshold for major proteins (bold print)

Other minor proteins potentially important for shell assembly were the relatively abundant proteins similar to KCP in CLC_1047/Comp51373_c0_seq1_3 and the protein similar to shell matrix protein G9MBW9_PINMA (Tri_138845/ CLC_25186). The former was only 53.7% identical to KCP_HALAI, in contrast to the major KCPs with > 80% identity. The latter was about only 30% identical to *Pinctada maxima* aspein [122]. With only 24% aspartic acid it contained much less than aspein (75%).

### Broad sequence similarity comparisons of the major *H. laevigata* proteins to other biomineralising proteomes

**Fig. 6:**
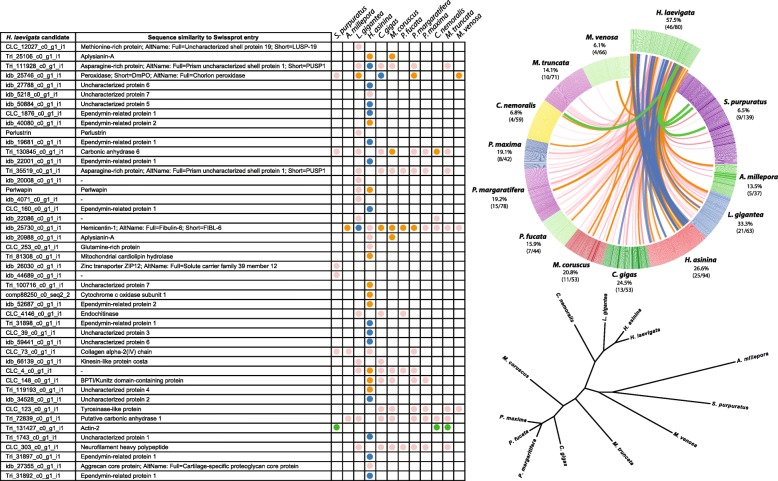
BLASTp comparisons of the *Haliotis laevigata* shell proteome against 799 biocalcifying proteins derived from 6 bivalves, 3 gastropods, 1 brachiopod, 1 sea urchin and 1 coral. Individual lines spanning the ideogram connect proteins that share significant similarity (e values <10e^− 6^). Transparent red lines connect proteins with the lowest quartile of similarity (with a threshold of 10e^− 6^), orange lines with the next highest quartile of similarity, blue lines with the next highest quartile of similarity and green lines with the highest quartile of similarity. The percentage of each biomineralizing proteome that shared similarity with the *H. laevigata* proteome is indicated. The table provides further information for those candidates that share sequence similarity. The tree is a consensus that was manually constructed based on previous phylogenetic studies (see Material and methods section)

Of the 80 *H. laevigata* proteins (collected in Additional file 28) included in our invertebrate-focused biomineralizing proteome comparison 46 (57.5%) returned some degree of sequence similarity below the arbitrary e-value threshold of 10e-6 (Fig. 6). With some exceptions we observed a general trend of phylogenetic proximity to *H. laevigata* yielding higher frequencies and higher levels of sequence similarity (Fig. 6). This was apparent with *L. gigantea* and *H. asinina* returning the highest overall frequencies of sequence similarity (33.3 and 26.6% respectively) although *H. asinina* is the more closely related to *H. laevigata*. *H. asinina* also possessed some of the most similar proteins to *H. laevigata* (primarily uncharacterised proteins) represented by the blue and green links in Fig. 6. Interestingly only 6.8% of the *C. nemoralis* (the only terrestrial pulmonate gastropod included in this analysis) biomineralising proteome shared any sequence similarity with that of *H. laevigata*. Also of note is the significant proportion of the *C. gigas* (a marine bivalve) shell-forming proteome shared with *H. laevigata* (24.5%). The proportions of all other bivalve proteomes that shared sequence similarity with *H. laevigata* ranged between 14.1 and 20.8%. The brachiopod *M. venosa*, the sea urchin *S. purpuratus* and the coral *A. millepora* shared the lowest proportions of similarity with *H. laevigata* (6.1, 6.5 and 13.5% respectively). Of the 46 *H. laevigata* proteins included in this comparison that shared some degree of similarity with another invertebrate biomineralising protein, 41 returned a significant match against proteins deposited in Swissprot (Fig. 6). Some of these (for example hemicentin) shared weak similarity with sequences in almost all species included in the analysis, while others (most noticeably the uncharacterized proteins 1, 2, 3, 5 and 6 and the ependymin-related proteins 1 and 2) were only found in the *H. asinina* dataset.

We also searched all 448 identified proteins against the complete UniProtKB/TrEMBL protein database. When we consider only the highest scoring matches of the FASTA search output (Additional file 4: Table S2, Additional file 5: Table S3), 78 *Haliotis* entries were returned (17% of the total). As discussed above, this number included almost all of the previously identified *Haliotis* shell proteins. The relatively small number of this group is also likely due to the low number of *Haliotis* proteins in the database. Another 21% (96 proteins) of the identified proteins were most similar to *L. gigantea* proteins, the sequences of which are derived from the *L. gigantea* genome sequencing project [132]. However, only 28 of these 96 proteins were identified in the *Lottia* shell proteome (Additional file 4: Table S2, Additional file 5: Table S3). All of these were minor, or even trace, components of the shell matrix of both shells, with the exception of the major protein idb_4071, a short sequence stretch (aa162–270) of which did match to the major *Lottia* shell protein MRP_LOTGI (Additional file 4: Table S2) [56, 105]. The third large group of highest scoring matches was to *Crassostrea gigas* proteins (39 proteins, 9% of the total). However, only two of the oyster proteins were previously identified in the shell proteome of this bivalve. These were the minor proteins Tri_111928/K1QJ54_CRAGI and Comp52297_c0_seq1_2/K1R3V2 (Additional file 4: Table S2). Another 43 identified proteins were most similar in FASTA searches to various molluscs, the largest single fraction (22 minor proteins) originating from a combined transcriptomic and proteomic study of the shell-less terrestrial gastropod *Arion vulgaris* [136].

## Conclusions

The shell matrix proteome presented in this study is the most comprehensive for a *Haliotis* species to date and with almost 450 identified proteins is also one of the most comprehensive published molluscan shell proteomes. It comprises almost all of the previously published *Haliotis* shell matrix proteins which, in most cases, were among the set of 77 major proteins (Table 1). A comparison of the proteomes of the nacreous and the prismatic shell layers indicated that most major proteins could be detected in both layers, but often with very different abundances (ie not always as major proteins). This was not the case in a comprehensive comparison of oyster nacreous and prismatic layers [23] and we interpret this difference to be due to the significant evolutionary distance between gastropods and bivalves. Furthermore, a previous comparison of oyster and abalone nacre forming transcriptomes also found surprisingly little in common [121], supporting the results reported here. It has been suggested that layer specific proteins may control the mineral polymorph and the crystal structure. However, the differences in mineral polymorph and microscopic structure of the two shell layers may depend not only on the presence or absence of certain proteins, but rather on their quantity.

Recent comparisons between mollusc shell proteomes [121, 126, 137, 138] and an increasing number of in-depth transcriptomic and proteomic studies are contributing to an ever-increasing list of novel proteins. The data that can support the concept of an ancestral “biomineralization toolkit” at least for the Mollusca increasingly appears to include a core group of enzymes such as carbonic anhydrases, peroxidases and tyrosinases, and proteins with repetitive low complexity domains and specifically biased amino acid composition. All of these features were also identified or predicted in many *H. laevigata* shell proteins (Table 1, Additional file 4: Table S2, Additional file 5: Table S3).

Unfortunately the determination of protein function is seriously lagging behind the rapid rate at which new shell matrix proteins are being identified. For many proteins the presence of a function, or at least an activity, is predicted by the presence of a conserved domain, as in the case of tyrosinase, carbonic anhydrase, chitin-binding and other domains. However, in very few cases experimental evidence for the respective activity has been obtained. Revealing the specific function of shell matrix proteins at the molecular level is clearly a major challenge for the coming years.

## Additional files

Additional file 1: Confirmed reading frames of the hemolymph and tentacle *H. laevigata* database. This docx-file contains a compilation of all reading frames translated from the nucleic acid sequence database of [38] confirmed by MS/MS-derived peptide sequences. Only majority proteins (shortest sequence containing most peptides) of MaxQuant ProteinGroups output tables are shown. Identifications not accepted, for instance most single-peptide identifications, are also included. Identified peptides are in blue. (DOCX 697 kb)

Additional file 2: **Table S1.** Organic matrix yields. This docx-file shows the organic matrix yields of individual shell fractions as determined by weighing after lyophilisation of acidic extracts. (DOCX 14 kb)

Additional file 3: **Figure S1.** SDS-PAGE of shell organic matrix. This figure in jpg format shows a SDS-PAGE comparison between the nacre acid-soluble fraction obtained with different protocols A, B and C, and comparison of prismatic layer acid-insoluble fractions A and B. Similar amounts of matrix (ca. 200 μg) were applied to each lane. (JPG 1284 kb)

Additional file 4: **Table S2.** Nacre proteins. docx-file listing all accepted identifications of *Haliotis laevigata* nacre proteins including most similar database matches, number of identified peptides and abundance in different shell fractions. (DOCX 306 kb)

Additional file 5: **Table S3.** Prismatic layer proteins. docx-file listing all accepted identifications of *Haliotis laevigata* prismatic layer proteins including most similar database matches, number of identified peptides and abundance in different shell fractions. (DOCX 309 kb)

Additional file 6. ProteinGroups, nacre acid-insoluble, protocol A. Slightly modified MaxQuant output table in xlsx format showing identified protein groups/proteins including those not finally accepted for various reasons. The table contains all accession numbers and various parameters such as iBAQ intensity, peptide count, sequence coverage, protein score and molecular weight. Contaminant and reversed sequence hits were removed. Identified vertebrate contaminating proteins were removed. (XLSX 103 kb)

Additional file 7: ProteinGroups, nacre acid-soluble, protocol A. See legend to Additional file 6. (XLSX 167 kb)

Additional file 8: ProteinGroups, nacre acid-insoluble, protocol B. See legend to Additional file 6. (XLSX 141 kb)

Additional file 9: ProteinGroups, nacre acid-soluble, protocol B. See legend to Additional file 6. (XLSX 174 kb)

Additional file 10: ProteinGroups, nacre acid-insoluble, protocol C. See legend to Additional file 6. (XLSX 179 kb)

Additional file 11: ProteinGroups, nacre acid-soluble, protocol C. See legend to Additional file 6. (XLSX 112 kb)

Additional file 12: ProteinGroups, prismatic layer acid-insoluble, protocol A. See legend to Additional file 6. (XLSX 245 kb)

Additional file 13: ProteinGroups, prismatic layer acid-soluble, protocol A. See legend to Additional file 6. (XLSX 58 kb)

Additional file 14: ProteinGroups, prismatic layer acid-insoluble, protocol B. See legend to Additional file 6. (XLSX 271 kb)

Additional file 15: ProteinGroups, prismatic layer acid-soluble, protocol B. See legend to Additional file 6. (XLSX 53 kb)

Additional file 16: Distribution of nacre and prismatic layer proteins showing a summary of the distribution of the peptides of each identified protein among gel slices (fraction 1 to fraction 12). Fraction 111 shows the number of peptides in in-solution (FASP)-digested samples. Nacre proteins are contained in lines 3 to 641, prismatic layer proteins in lines 646 to 1285. The peptide distribution was derived from MaxQuant output files obtained by analysis of combined nacre sample raw-files and combined prismatic layer raw-files. (XLSX 303 kb)

Additional file 17: Peptides, nacre acid-insoluble, protocol A. Slightly modified MaxQuant output table in xlsx format showing peptides to corresponding ProteinGroups files. The table contains the peptide sequences and various parameters such as peptide length, peptide mass, number of missed cleavages, charges, posterior error probabilities (PEP), peptide scores and peak intensities. Contaminant and reversed sequence hits were removed. (XLSX 287 kb)

Additional file 18: Peptides, nacre acid-soluble, protocol A. See legend to Additional file 17. (XLSX 432 kb)

Additional file 19: Peptides, nacre acid-insoluble, protocol B. See legend to Additional file 17. (XLSX 547 kb)

Additional file 20: Peptides, nacre acid-soluble, protocol B. See legend to Additional file 17. (XLSX 587 kb)

Additional file 21: Peptides, nacre acid-insoluble, protocol C. See legend to Additional file 17. (XLSX 555 kb)

Additional file 22: Peptides, nacre acid-soluble, protocol C. See legend to Additional file 17. (XLSX 339 kb)

Additional file 23: Peptides, prismatic layer acid-insoluble, protocol A. See legend to Additional file 17. (XLSX 902 kb)

Additional file 24: Peptides, prismatic layer acid-soluble, protocol A. See legend to Additional file 17. (XLSX 884 kb)

Additional file 25: Peptides, prismatic layer acid-insoluble, protocol B. See legend to Additional file 17. (XLSX 134 kb)

Additional file 26: Peptides, prismatic layer acid-soluble, protocol B. See legend to Additional file 17. (XLSX 116 kb)

Additional file 27: **Figure S2.** Sequences and repeat structure of uncharacterized major proteins. Sequence regions covered by identified peptides are shown in bold green. Predicted signal sequence peptides are underlined. Collagen triple-helical sequences are in italics. In sequence alignments identical amino acids are shaded yellow. (DOCX 75 kb)

Additional file 28: Sequences of proteins used in proteome comparison. Conceptually derived protein sequences of 80 *H. laevigata* shell-forming proteins used in the generation of the Circoletto figure (Fig. 6). These sequences represent the 77 most abundant sequences from the shell described in Table 1 (77 proteins), and the minor proteins UP6 and UP7 (reported by Marie et al. [21]) which are encoded by three contigs. (TXT 32 kb)

## Abbreviations

aa: Amino acid
CA: Carbonic anhydrase
FDR: False discovery rate
HCD: Higher-energy collision-induced dissociation
iBAQ: Intensity-based absolute quantification
IDP: Intrinsically disordered protein
IDR: Intrinsically disordered region
MS/MS: Tandem mass spectrometry
NGS: Next generation sequencing
PAGE: Polyacrylamide gel electrophoresis
