# Supplementary material for: In-depth proteomic analyses of Haliotis laevigata (greenlip abalone) nacre and prismatic organic shell matrix
Source: Proteome Sci. 2018 Jun 15;16:11. doi: 10.1186/s12953-018-0139-3 (PMC6003135; doi:10.1186/s12953-018-0139-3)
Supplement: Supplementary file 1 — Confirmed reading frames of the hemolymph and tentacle H. laevigata database. This docx-file contains a compilation of all reading frames translated from the nucleic acid sequence database of [38] confirmed by MS/MS-derived peptide sequences. Only majority proteins (shortest sequence containing most peptides) of MaxQuant ProteinGroups output tables are shown. Identifications not accepted, for instance most single-peptide identifications, are also included. Identified peptides are in blue. (DOCX 697 kb) [file 12953_2018_139_MOESM1_ESM.docx]

>**comp100168_c0_seq2_1** len=2206 path=[2299:0-165 2465:166-190 2490:191-893 3193:894-911 3211:912-1191 3491:1192-1207 6450:1208-1318 6482:1319-1331 3631:1332-1336 6516:1337-1358 6538:1359-1360 3660:1361-1363 3663:1364-1462 3762:1463-1702 6609:1703-1709 6616:1710-1720 4020:1721-1902 4202:1903-2056 4370:2057-2101 6720:2102-2149 4469:2150-2205]

PRINTLDQTVRLACCSSPPVHSITSR*DFLTVSDQSR*ALDTHSV*DGVSRRHVDTKTSS

LSHTDPLSARDLFKRYKTTRAVCCFMWEG*FTSGDI*FGFIGTTTKSAAMKVYNLCFCVG

VVSLCLVTYSSSSSTNETVSNSTVDSEVKSDYDDVVVEAGDVAGGTGDLTPEEVNELTPE

DEDMETLKIEELGGLIDAKDMVDNEEETIEDMEVEDIPEDKEDLRFEPIPMPTDFDSYDR

DGDGFIVMKELIEVTGAFENVAEAFKACDTNGDERIDRAEFRDAPWDLSGKRPEVVIEYI

N*PWHCVLLSK*CHVTISLSDTV*LAGFATTLHVHTCSGECVISVLWTI*RFSSLRFLTS

LMQTFCRTKPAIRTDTGTKQVLDCVGTCLSIQAIPWIYIKLD*LSDVL**PVFVCSRSGY

E*VMCY*K*ID*TRPVYCQFAIIDFF*RIKMFVNIGVFEHEKCTQYEFYRTIGNLR*DTK

LNQNENL*TSFLFLFETHQREE*RLPSWIPRAVSQLLPDSQRWP*SNGVCTLCVSDSITV

QGKVIILDPSLGDMCAFPPCHVTLFR*N*AYCTFPFILHSHYHRIVITLRAILVSMTKLQ

SVFRDCIFG*ICESLVLV*AVKQRFSVYPAIHVIKVSSVY*YSIYRTL*RLLDSR*FGQY

HWGTIPSGLTGFHCKLKDLLV*TVERVHSPVKLS**AGPKVKDTLSEVIFV*IDVQKRQQ

SVVS*FNEHVDAIS*X

>**comp319327_c0_seq1_4** len=271 path=[249:0-270]

EFRAILFIPKRAPFDMFEGGSNKKKYNQIKLYVRRVFIMDNCEDLMPEWLSFVKGVVDSE

DLPLNISRESLQQNKILRVIKKNLIKKSLD

>**comp101252_c0_seq3_2** len=1445 path=[12575:0-38 12614:39-47 12623:48-159 12735:160-162 12738:163-166 12742:167-571 13147:572-810 13386:811-817 13393:818-863 13439:864-947 13523:948-949 13525:950-1444]

GYTRNCILQEFKSTELSYNC*RTSGELVSLNFYHIMAFRIVLACLLVAMATATRSVGSCV

VKSNPDFRPDPDDCSVFYVCANDILYKFNCSNSVFDPVTRTCVAKGSIYDRCTKKDTPAQ

TVICLPGSRALIPHPENCAQYYNCSGPKQGFRWPEHLRECPYPQLYNVISKQCEHYSMVK

CGDREQPISHCDYRANQCEAAHCIPCIVRYPSCKGLPDGLNQWVGREDSPYYVVCDGGRM

VYSGLCNQAYGPQTFDKVRKMCVEVTL*RHEDQEPFRFLAVGIVSELPLS*FVISFLAVI

FTFIFISYFPGPFQRNSPII*YMVGAK*KSNLDIQVSFMCSRN*M*PIRLLIRISLVFVF

AHE*IMKTAMTIPSLA*IILQFERTVLFVMIQSWY*KLKY*PVSY*MCTQVPLLLVSTSV

VFIFESLILVIIMKNML*LCVV*AVLHKLVVNLNLIRQP*PVVFMLC*TYSLIRV*CLLN

MX

>**comp101550_c1_seq1_3** len=2684 path=[2888:0-1014 3903:1015-1703 15176:1704-1706 4595:1707-1802 4691:1803-1898 15270:1899-1899 4788:1900-1901 4790:1902-2034 4923:2035-2062 10588:2063-2111 5000:2112-2247 5136:2248-2262 5151:2263-2395 5284:2396-2405 5294:2406-2560 5449:2561-2590 5479:2591-2683]

LDSGVESIWLPVCGYLRLQPIIAKMKSLIRRQSANFLCILFAWLVSVVKTEVQPLNDGNV

NSILGTHEIVFVNFYADWCRFSQMLTPVFEEASNKVREAFPMPGRVVFGKVDCDRETNIA

QTYRINKYPTLKLFRNGEVVKKEYRGQRSADSLAQFIKDQLTDPAEEHTDIESLEELNNK

KRHVVGYFEAKNSDTYKTFARVASSFRDDCKFHALFGPASEAERTAGDRVVFKPPGTGKQ

DMMFMGSLTDFDNLRVWIQDKCVPLVREITFENAEELTEEGLPFLILFHNPDDHGVVEKF

THEVSTQLLHEKANVNFLTADGSKFTHPLHHIGKTLSDLPVLAIDSFRHMYIFPHDVTKD

LDKPGLLKKFIEDLHSGKLHREFHHGPDPTEATPAQIVNEQPGDAKHVPKDTVSGGVPPI

KKPTSPPESTFRKLAPSSNRYTILKDEL*IIKISFKMKS*FYMSV*KWSEPVWVYIFF*M

CMYELGIHI**FMCPSVQGSLCFCLSC*CNSIRKNQILLFQLQM*RT*RDLSKVQ*RVAG

YPEG*SVCQSENPGLNPHMETLLKPILLVPGILKIV*NQTHVYLSYPSH*QIVFYSSNCN

STFEVCLIWS*LYSSDRIQKLNPARNC*QL*LLFFTQRLVPFSQSLLTLHSPISVRNVCE

INMVSIILF*NSICQS*C*LPQFMVTSVL*FHHKSFAFFEVKLQYKTNKCASRGNNCRNP

ADLYCLLFKDIF*VLQMTLPVL*NDFHLTL*GILFVVMIWISSVYCFTFCCMNNEHGIIL

LRCLLFR*DNVAALLAVLMQLKHVFVISYVQEIQRD*DNLPLFPSISLVIIKKVMFQQWI

FALNLLSFLLAKHQACLLQGTKHISLFAAAHLSFLKKSCSVLERHC*VRTD*CL

>**comp101638_c0_seq5_5** len=1626 path=[2732:0-74 10580:75-88 10594:89-96 2829:97-107 2840:108-110 2843:111-137 7690:138-156 2889:157-160 2893:161-208 8024:209-223 10658:224-253 10688:254-275 3004:276-354 9007:355-414 3142:415-524 3252:525-1187 3915:1188-1601 10991:1602-1602 10992:1603-1625]

KKTCAEGAINTTVGPTAPSSGTVERKDIECTAATMDIHIPRTLLPTHKVTLRDPTCVVPS

NGTHYIISISFNQCGTTVSFSNDTVVFNNELVATVIQGSNPSTIIDYGLSKDDQRVSLEC

VYKRHTSVDLNYRPLMKAAHFYEKRYGMLDFEIKQYSDEQFNTEIAQTDVPTGVVLNSEI

FIDLIVDRRQTSDIGIDVPSCFATPTPSPTDPLYYPLIKSSCPAQTDVRTFTSPNINEFR

FSFLAFKFTRAPLQKPDASLPAVPMVYIHCQVSACPRGNCSSSCAQGHRAKRAAIQDSTH

LISSGPFYMKTDESTTGYSSIVVAVTMSVAVAATVVAMAAIVAWRRSAAALRAYRSLPMD

ITS*KFFFFFSNILLSSLHTNQNILTRELFIISLSKY*FIVFTAYFINILYYFLQFVS*H

FSSIRHYFIHLATIFCNHICKIALSASVLILDVFVNC*KRL*TESN**LLNVLDRFSICV

RLLA*NICGDLLLKTQSINECHAKCC**LTLVGCFLVVFSLILCIVCEQRFVLSFNKQHS

QX

>**comp101858_c3_seq18_4** len=2097 path=[10064:0-47 10112:48-189 10254:190-207 10272:208-1130 729:1131-1154 753:1155-1893 1492:1894-2096]

GTAFIQTQQLHAAMADTFLEHMCRLDIDSAPITARNTGIICTIGPASRSVEMLKEMIKSG

MNVARLNFSHGTHEYHAETIKNVREATESFASDPILYRPVAVALDTKGPEIRTGLIKGSG

TAEVELKKGATLKITLDNAYMEKCDENILWLDYKNICKVVEVGSKIYVDDGLISLQVKEK

GADFLVTEVENGGSLGSKKGVNLPGAAVDLPAVSEKDIQDLKFGVEQDVDMVFASFIRKA

ADVHEVRKVLGEKGKNIKIISKIENHEGVRRFDEILEASDGIMVARGDLGIEIPAEKVFL

AQKMMIGRCNRAGKPVICATQMLESMIKKPRPTRAEGSDVANAVLDGADCIMLSGETAKG

DYPLEAVRMQHLIAREAEAAIYHLQLFEELRRLAPITSDPTEAAAVGAVEASFKCCSGAI

IVLTKSGRSAHQVARYRPRAPIIAVTRNPQTARQAHLYRGIFPVLCKDAVLNAWAEDVDL

RVNLAMDVGKARGFFKKGDVVIVLTGWRPGSGFTNTMRVVPVP*WPSGAPLLAPVPSPPL

SFPLGQQRL*CSLWAIVWRWWAGTPGKINASKTCNRDQLLFRALPEPGVEEECRTGNPDF

ITEGRQHLWALLL*KVVRIPSPSLESGDSKRVGAEGVGPRVPV*MTTSGPGPDLLSQQLW

PPHFLCTPLLSLQTLHSPPCILQSLQACCYSAHLNVNKQ

>**comp102281_c0_seq4_1** len=4840 path=[11773:0-133 5565:134-134 5566:135-785 6217:786-1212 6644:1213-2728 8160:2729-2746 8178:2747-3403 8835:3404-3917 15131:3918-3922 15136:3923-3927 15141:3928-3940 9372:3941-4640 10072:4641-4838 14334:4839-4839]

TSQAVVRLQCVLVMLLLMT*TVCVESGMSAPFHV*LLWNLGTASWVIL*CAEQD*VKDTH

DSASRICCND*HNLWITTMTKHRCECRSIVIKYMCGSAAVSKMAVVFVLVSLMLASVGHC

SISVVDSHDIWGISSHGSRTGDDSSVSLMWGVADTTASVGHFFNYTIPQDAFKGNDLQYK

VMEAGKDTLPRWLVFSPKKQLFQGLPTFSDLGQYNIEVVAVGLNGEQAKDIFSIFVNEDT

GPAAGSTLRFKEGGPKTVRCKRSEPETAVSIVVDVDVDNLGAQERLDILTKLVSHLNLAE

DMVKLSQVGQKPLTDASALVSGAGDAKTLQTPGSFVSWLVGCGRVQQDHMQALQQVETDS

SNGVMADAIGYPIHVWHVTNSRFQEHKRRKRQAIRATATPAVTPTLPIRGPTDTIRATGT

EQDAMTRPVPTLDSPSFGIQPTKTMAPMEKTASPVMPTKVPGGMKPSVVMPEEKPMTKTQ

VLPTKTYVMTDVMPSKSVPMPTDAPTRKPASGACRDLVPVVKEPMKNMMYSVGEVIRYKI

PENTFYDCRVGNTRKLRLLLMANTTTALPSDYWIKFDMKKQLIQGLPLEGNIGKHLFNLI

GYNADQADTVAKTAFNITVIPDPRSASGQLNHEVSMTLDYDFEKFSSSTENKISFLNKVA

KLNGERNAKNFKMNSVTKGSTVVSWTNTDLASPDCPVDQIKAMMSKFVQEDGSLNKEAIR

GMKPFNLKGVGVVPLGACENDPNFPVLPGTKDDTDDPSSPDGTDTTDDTSVDENPPVTPE

PEPTTTEGVRAGSAGGAQSDDIWITTVVPAVVIVAILLLALLIACILYRKKRKGKMNLED

QNTFVNKGVPVIFQDELDEKPTDSSKPLLMEGQPPCPPPEYQRGDSQGSTPPVNHKQPQF

DHDDHIEDTNVTSPLYQPPPPVTASSGNKQPRPHVPQPAYRSQPPSIPP*TLPHLREESS

ED*GTTQGCGFVEDCYCLRTMHCLEQTLGSWSSFFILLIRILILTNFNCTALFEILHIPV

*QLKEILEMKLYPSNLKYQCFTCGGTLNLHALLIMPFMPYEETVTSWFLYVKAGCR*TEE

KLWLPSCCISAWRKPCGYMQGLMTVDCST*LRSAMCNVSVVLVISGDCYNTQPVAGGLTN

WMAELDYRVTQWSFLTVGFSFKRGF*HCFFRMFSNHICCYDIRLF**N*YLILLKLNRK*

SSLS*SVVFCLRQVNKQEVQSKSWFS*SL*AQSQCVQQSDGV*NPVVG*VMFTNASI*SA

IVCIIYCQHRKLAAL*NVFCCRRSLDLYGQ*NMTFDHIVTIC*KELKKLFHVKYVLI**I

HIVFGGIYAILL*WSRFACIIR*HIMSGTE**HVTSR*GCH*FQLVQVYVHRIINHCECT

LFKLI*YTDCIDPTCCIPCLKGGLITYFSPA*LIGSLHCLLSDKAQC*V*VLKSCLVY*S

MSSVFVCSCIYFITDDVAILCQYID*LHYLLVIVSFSCL*LSFFSHVLILKSSLLQGHCT

WWKVRGENQF*DFVHSGMLTCPSNTSIMCSCGGIHSNADISAK*QVRFFSQNILSNLGHL

F*FISCVECKVAPAFVSFELINPAFMSWQSYFILLRPVPSVYRQVSALRTVRAX

>**comp102499_c1_seq3_6** len=4766 path=[13092:0-102 6066:103-745 6709:746-754 6718:755-2267 8231:2268-2292 8256:2293-2414 8378:2415-2428 8392:2429-2642 8607:2643-3196 9162:3197-3240 15203:3241-3459 9425:3460-3485 9451:3486-4033 9999:4034-4476 10443:4477-4740 10707:4741-4765]

SKLRILRATNVLRIRVLVIKV*T*DC*YFQSRTLHILQCQ*IVALLTAVLKMHRSASTNI

QYESGNVMSRGIYQVINQNNAKQP*TNKQINISNMKNLQNKSD*LKKQKNKNNYQF*YKT

QKNISKKSFALFRMLNVCSRFCFQMQGIYKH*VLYMTSEIRPILILC*AHDIMNIFIVAS

RWAAPPLTMTRHSHLEIGDCCCSTCYSSWVPLKKYPSSIQKQNQGQNQNTGTYPRCDCVT

LETVRSVC*TGS*QTKCAEPWYNQCKC*RNVFSNRKNLEVNTTNIF**GLLASHKLAYNN

LNSDSHVLINLLSHFYSRLSSARVGF*I*F*LRYIYL*LH*ML*PPYSVTTRPDAAIFKL

NTVHINLYSFIQLCLIQNYFYPIMIHLGRLEGKSNNQHKRMCCCWYKTYLCHVTQSL*DV

MVQMAVLETMTMKVYNTGHGYIQTSIFRGKNC*YYLLGKLSVPRRQ*NLFPWYTAT*PIP

KHNHVIRGRQLFHTQEFKFTSPLWLDHSYVLTNLC*LQYHIII*W*KY*LKTENLRCKKL

C*LCLLYT*YVPAP*STLRTHESWVTRFFILKKCFTSNNS*IFKRLCLRTNYIYILFFLT

CIILVPSSIHTQPEK*NTYFPLISKCSP*RTASGQVYSSNSV*QYLSFLQYL*QKRYDFS

NFVPESLN*PN*WSCCSEMA*HLMLITNTIKVCIIYNQQSYISKLHKNTYIFI**HSTFN

IC*TEGILFELHIKDMNLFCIHN*TNIRGTMNAPQIDRNVCRNKQIF*NDRQLH*GRKLT

YYMVSTTNTNMEINIFNDRTNNKTLKLNAF*MLTMLKEGNIS*P*RNDNEHFRDNTHWVI

VILLVPKYMYM*SLTNINGYHQTITVVNPH*A*CVLLRQ*FVFFHLELCDVIATAKEVVI

TKAIGRCPFKVMVSTTL*TVPGLNMYTFHRSVLLEIQDFDDTECSLESEVHVSL*GFTTI

INRIQSCAFQQSSFLFVSNSATTVLRTEYYTCFSTRLCSNKAWTPSDAIFFVSNTQ*WTS

LFLHSS*STEAAGRLVMASSNVML*RWLKNISVKHQVQLA*N*WHYVAVNILKPRLPCMF

ITIQKLNTLKSGPCFISRNMP*KYQTFDSKC*IYFAKNVKIAVQILDFCQIRIMELDGQT

T*NVEFRMITVRTKKARSSTSEDSIFFCRYCVAAGQGTPTL*FTGGSPGWKAWTGAVGYE

GPTMYTT*P*PWEGVPGMGAKPVEGEDG*T**QEPPPPFV*LLAREAGGGGGGPHAAELG

RG*ELTRTFGMLDRGAVLGGGAVNSGGW**GPTKVDDSEGGILCIVGGWEVGACKDVQGK

NVRPEFISMGRF*PGTKELALTFRLLALSL*LTKPSDC*M*MGWPSHRALAALVSASNTW

NLGGISLIGFTSL*LPYM*YFRLPFPLPSLLISIHSVLPRGFSSFSQTL*LPSISPSSVE

LSSLEGLKLPEHSLTQPLSVDRQPSSSASFSR*GSDTELPPFFRSGFGARLKSDGF*LIC

VLTGNLFLMLD*P*S*CDIIG*ADMSTGPIMGGGGGGGNIGCWDCWKLPITLEYLNLFIL

MQLDQRICTLTHTVIRLSHTTDLNLSVVT

>**comp102930_c0_seq1_5** len=2482 path=[1:0-245 247:246-278 280:279-294 296:295-442 444:443-462 8038:463-516 521:517-619 8120:620-636 5253:637-652 8145:653-741 5172:742-760 764:761-1008 8270:1009-1029 1033:1030-1105 1109:1106-1125 8321:1126-1152 1156:1153-1341 1345:1342-1358 1362:1359-1441 5058:1442-1490 1494:1491-1514 8477:1515-1535 1539:1536-1645 5572:1646-1649 1653:1650-1656 1660:1657-1712 8619:1713-1734 8642:1735-1737 8645:1738-1754 3493:1755-1908 3647:1909-1910 3649:1911-2481]

LGWP*RIASDELGDHFESCFPPFPPLKMAKVLGVAVDGSDISNAAFEYALESLYKAGDTV

VVIHVAEYHISIPAVGSSDVDKLCEEMKKRDQEIGALTATYTNALRQKSISGRAVRPTGG

KPGEVIVKTCQEEGISHIVMGTRGLGAFQRAILGSVSQYVLNHSSIPVTIIPKKA*AIGL

MWNINENISIWRFS*DLKDF*QIFRGEIEL*AKVKIAFKADC*REN*IIISEICGEAFIV

RGYRLK**NLDSVLLHLDHG*CVSCLLLVTSTSTIVHTD**FM*YNLSLDELSLMDQCQC

LSMYMRQDIPRLENQTLPSILYKHKCNWVFDVPAYLFTVHMTHCDGISNWTESCYWE*IP

AIFQVFLFNLALANADFCF*FELPTLLQLHLLLEENAQRCVTWVSTTQISFIVIGYISNA

TWQVLEQYSRFLFVYQLPW*HEKDCFKTPDLLMELFEETIQDQKYLI*FWKHYQCTSREC

CFSSFLVLHWKFFKHCYRGNWNYLLSLYGIFALILMFSSSCIYSLRRVFLFVCMQFIQLF

TPLSEFYREET**KCEIYK**HETFTFCITMNTNLVLQVLIREFLFITKLLTFRLLF*KC

SIISLFLANSSSKCLFFSEYSMFINRVHCKRYFHSVVYTLVSRLITDMFLNVL**YYCIS

RRSHVNLGYI*CDLTFLVMMLHMGVCTEHQKLYFTIKGTITAKPLVPLL*GTGLSTLCKL

QDSPQPQSC*TSMTVLRRHNPVVNITYSTI*ISE*I*HQIL*PTTSVLSKLHDFLS*QLY

LIKAIDLMDCIISSLPNDACYAYLLTPCTNSYK*LSHLYSCSI**IFX

>**comp103017_c0_seq2_6** len=3012 path=[1:0-111 113:112-463 465:464-1959 27157:1960-2163 35207:2164-2164 13002:2165-2840 35333:2841-2843 25906:2844-2980 26043:2981-3011]

KGQLPQRW*GKYISLQCDFGVLIFFCQQGPVRSRIVRRYSCFTANKPRYLRLHITMVLSS

SQAMRAYTLLLVLAVLAVSYSSAIPKPDGDKKDRTKEKTLSEQEHDDHGEHNNDYDHDAF

LGKDDAKTFDQLTPEESQDRLGLIVDKIDKDGNGFVTEEELKDWIQYVQKRYIITDTDRM

WKDHEPDSDDKLLWTSYKKRTYGYPDEPEEGSPTYEYRDMIQRDRRRWEKADKDGDNKLS

KEEFMDFLHPEDAEHMREIVVHETLEDIDKDKDGFISIEEYIGDMWPDKDKGEEEPEWVK

SEREQFNTYRDKNKDGKMDGEEVREWILPTDYDHSRAETKHLIHEADADKDGKLTKEEIL

EKYDLFVGSQATDFGEALTRHDEF*TQII*VFQFGKNPSS*TELQQDCVTKPSLITWRRD

NCF*SLKHCDKYETISSTQ*T*ETWTYRIIRDLVC*SRFSKYRSCRDIFVLHLESSTRLD

SCSGIVTFDFLHCPIGFQDSIM*PG*AVGCVVAVALNLNLYLWNVHDVRVRF*QYCAQSL

ICLNKKSNLNIESNVVITRSQLCSEIEQIFFINLSLLSVVTDLTLFIKVLC*HSHRS*LW

NLFTC**IFNENPQSMLIFIRTYYFLVTHKCWSLLFKIQETLILIKRKYIVPFFNECYHG

NYGYKEALLCLCVWSL*NGIVPNHVLLSVFFQMVPWFNLVLLIVKVPLN*ILERLM*NLS

RLPKM*DDLYRSFS*NLCSVKSNKNVLF*QQFQTFGYL*IFVCLDILSYNKVHNDT*LIF

IPH*TGVVSKVLESYDFPNN*LRIPQLILKCTNIDKV*HPQKGHLSQHHSLDVKII*KIR

GLRRHSTFFKMNHVFDMSDVCTNKRTVIIFN*SQPPE*LFPGFFISGTMLSRSS*LQTIF

*KVK*KCFEFWLSMNLVVLC*NCCLN*ISNISSQMQLGVQEYSDEITCSVVLSL*IEK*S

GKLVVFSVLPYSLNIPTGTVG*QCG*SFRSSHQRPNFDSPHGYX

>**comp103470_c1_seq40_4** len=1311 path=[1358:0-204 1563:205-210 1569:211-219 1578:220-228 25904:229-231 25907:232-234 3619:235-270 25943:271-275 25948:276-279 25952:280-323 1682:324-350 1709:351-354 1713:355-365 26024:366-383 26042:384-389 2744:390-408 26060:409-419 2774:420-426 2781:427-438 26090:439-442 26094:443-446 2801:447-459 2814:460-465 26103:466-483 26121:484-489 26127:490-495 26133:496-503 3212:504-560 3269:561-584 3293:585-596 26207:597-613 26224:614-620 1979:621-636 1995:637-669 2028:670-681 2040:682-686 2045:687-690 2049:691-705 2064:706-729 26301:730-734 26306:735-752 26324:753-758 26330:759-789 26407:790-797 2156:798-831 2190:832-863 26452:864-864 26453:865-876 26465:877-918 2277:919-984 26494:985-989 26499:990-992 2351:993-1011 26514:1012-1041 26534:1042-1044 26537:1045-1106 23610:1107-1119 16350:1120-1122 2481:1123-1175 26617:1176-1184 26626:1185-1199 26641:1200-1202 25307:1203-1230 24062:1231-1263 2622:1264-1310]

DVAALVIDNGSGMCKAGFAGDDAPRAVFPSIVGRPRHQGVMVGMGQKDSYVGDEAQSKRG

ILTLKYPIEHGIVTNWDDMEKIWHHTFYNELRVAPEEHPVLLTEAPLNPKANREKMTQIM

FETFNSPAMYVAIQAVLSLYASGRTTGIVLDSGDGVTHTVPIYEGYALPHAIMRLDLAGR

DLTDYLMKILTERGYSFTTTAEREIVRDIKEKLDYVALDFEQEMATAASSSSLEKSYELP

DGQVITIGNERFRCPEALFQPSFLGMESAGIHETTYNSIMKCDVDIRKDLYANTVLSGGT

TMFPGIADRMQKEITALAPATMKIKIIAPPERKYSVWIGGSILASLSTFQQMWISKQEYD

ESGPSIVHRKCF*RYLSYCCNIVLYMVTWKSFLDTIWQYRQCSCNLRSYVMLEAASVNIH

VHFCCFHCVIF*INMYN

>**comp103479_c0_seq1_1** len=4049 path=[4032:0-334 4367:335-357 4390:358-739 4772:740-755 4788:756-859 4892:860-869 4902:870-907 8346:908-924 4957:925-1088 5121:1089-1096 5129:1097-1135 5168:1136-1136 5169:1137-1895 5928:1896-2089 6122:2090-2127 6160:2128-2428 11177:2429-2554 11247:2555-2570 6603:2571-2625 11296:2626-2630 11301:2631-2645 6681:2646-2694 11327:2695-2700 11333:2701-2703 6739:2704-2704 6740:2705-2705 6741:2706-2710 6746:2711-3086 7122:3087-3302 7338:3303-3308 7344:3309-3334 7370:3335-3344 7380:3345-3833 7869:3834-3841 7877:3842-3874 8132:3875-3897 7935:3898-4048]

*AGRSGTEIACVGC*AGNMSRIIIAAVCVLFAGISHAQDNPSFDDAGPNYIKEGDEINLS

CTTTADVQFMASKQLAIVHKNLATEAITVISEQESLLVANPDNYVIKLEMIPIVRVTLKM

KTASAADDGEYICRTQKTGTTDALESATKQVVFVAPVSEVKLTVNGEVKAEGDTIESKPA

TLTVTCEAKGSNPEPLIQVMVGDQMVETGTVNSIMDKEGNTERRSFKSAVDFELDFVHSM

FDKALKCQAKPMLPDGVTPPSPAEMTVSVNLKGIVDKPVISCHNNTAGINDRRVTLTCYV

SKVPKVKSVMFNVGNRHQLYPGNQTESLTEVVQKGFNDTHDEVILKFYEMKEWHFSANIY

LDVKTFDNQVFQYPVYLLKVNGATSLSGAITLLAVCLLAGLLQKVL*TSEIMQQI*DSTK

SRE*EAAGAEEAKARPDQLDLPTTHSLQTADDINWLYDISKVC*ENLRQFGQMIVIKCLV

*KFALEMFQFEEDESFRTSSLFGEYFLPVSYYRLLYYNSDHCCFIDFDSRQSKIIIIFPS

QQ*YLSCDGL*VLNVVA*NPDIC*V*LIQGRQHFDKQDIFHFLHQPF*V*RPMFLYVHVL

KLLPVST*EVLLPVLC*MHYVIFQFIHLFGGMFILMFLSMCCVGMLPS*LNLSPLVYCLA

AFLLLL*LFLPVPLIQVFTA*VLPCKY*LTYEFTASDVHTRFSLFITIFEPSNQHLRVVL

RLGVGCL*AYVMKCLMHSNNIQYVCRYIVYQLSYNILYV*PNCAAIRVYIPQ*FI*TTW*

CPSHASIPTCRRTR*YSIA*AQFA*FNHNFDSNFVETPCACADSFLPKR*GYSLPATLQF

PPQCFGCPSPILHPDAPPPSTSYHKCVFLSHYPCPEPIKMLRVVNLFFYLSLPNTQPFST

PHPPCPVLTPPCPNLNIYSTKGTLEGHFIVVLCVRLRRMLYLCTLFCLKGNCFV*SCTAS

ALDKCYYRTRE*RATHLQSTSNSMGKCGTGYNSGIPCNICVHVGLV*CSLYR*IVIVTVV

YNKSYLPFTGVHIRPILICNLFLYAKFIHSIPLQD*CSSLSIVGLQWKGDNSGCSNIG*L

V*NYFERSQLNTPGSQWATHFWEFIVCFL*FYESFVYSTTMYTSCDSRITTNNILCTWLC

SHFLENKTLVLQFLSGFIISCVLKGKC*TTSFPYQAVWSCKIY*NTQKKRLWPRMTLTII

LLFSIWEKNVEIQNGGTKSFNCLMEDNILYLFCKRAGPTGLRS*LYNDGSLIIGEIGVFT

TAKPKLV*VYKVT*VHSDVAWTPPMLECTLGTCLQEWM*CLEVVAGLNHPHHCKVYNDCD

WPNPNL*IHLDYCCLLLS*THHVVIKVFTA

>**comp104004_c0_seq4_5** len=1737 path=[4425:0-35 18479:36-51 4424:52-458 4883:459-461 4886:462-1736]

KMATLKLWRTSVMTRFSVLVFSAVLSVCLAGQELVITHAPSYVSFQQQADQLHTSEVPKV

LAHTLGLKTESDLGWKGMVQGSLFKRPKANVLMTIVTHPDQPLPLKKLAKFPVNDDVAFP

DVEGVMNSLQGMFLEQSPLMLDLHADNNMFDLKTEEQLFRKLPNSIRKLSDRLLDSDSII

HKLSSGSLNMTLNSDLSLMSEMQMIADVMKTMSENPKLVKNKTPDLFSFTIYGLRNVADK

HGPKSEQAKDATKMISDFVDHVTDHFKSLYKDNVVVEVLSAAPEKGHVRKVRSLMATTPA

PSKPTSNNLNLADDYSQEYPAIFNIVLWLMLVLAIAVYAIVYGMWYMDPGVDSIIYRMTS

QRLKKD*ISS*STVFLLCP*T*PWSYIYLIPSKTFSDIKRIEIFLKMKIRNEVYLLFVI*

G*SCQREVTLTTLHG*TG*PKSL*M*S*L*KFSF*TGYF*LLGH*GNVNVKTHHTYGTDM

HILSLKPFQRIILSFM*QSTRCTSAFQFCNEICTRHSGPFRVYIMLQEIRNWG*FHKAIL

TLRRS*LRCVKIGLRSS*H*DCLRSMLEYWSYDHRSTKX

>**comp104092_c0_seq1_6** len=3885 path=[1:0-41 43:42-242 244:243-569 11294:570-584 8268:585-585 10817:586-609 616:610-894 901:895-942 949:943-973 980:974-998 1005:999-1044 1051:1045-1069 1076:1070-1143 1150:1144-1167 1174:1168-1345 1352:1346-1667 1674:1668-1691 1698:1692-1712 1719:1713-1736 1743:1737-1760 1767:1761-1784 1791:1785-1847 8269:1848-1871 1878:1872-1997 2004:1998-2009 8849:2010-2033 2040:2034-2135 2142:2136-2159 2166:2160-2222 2229:2223-2261 2268:2262-2510 2517:2511-2513 2520:2514-2534 2541:2535-2537 2544:2538-2699 8083:2700-2741 2748:2742-2744 7735:2745-2801 2808:2802-2837 2844:2838-2858 7993:2859-2882 2889:2883-2894 7945:2895-2918 2925:2919-3011 3018:3012-3023 3030:3024-3047 3054:3048-3152 3159:3153-3176 3183:3177-3281 3288:3282-3305 3312:3306-3339 8149:3340-3363 3370:3364-3372 3379:3373-3413 3420:3414-3436 3443:3437-3460 3467:3461-3526 3533:3527-3555 3562:3556-3572 3579:3573-3620 3627:3621-3646 3653:3647-3670 3677:3671-3693 3700:3694-3719 3726:3720-3746 3753:3747-3770 3777:3771-3884]

SDQSEVI*TPEFVTRLKRVLVQSNRRILKVYITT*LAKFTGCQSGVHFMNVTS*LCCKVA

DTARIIEK*FSLLHHSPLQSGMHPWKVKQLLSSC*FLDFKSWTYINT*TLEKVSSGCVID

CE*FLYQICYSKNAELTKGFTQN*ISWKCSEFHTNQNLLKSQKNICDSVQKTYILKMASF

LRRRRGLLIKAMLMVPVLWLLSVMLYSGPGRWASVDGEDKVPRDRRDGSVDKPLHPPPDF

HPNEVNKDHIDGERLGHDRKIHESDKDSLKFPGNKEDSNVKPPVDVGVVRMDKQKVTPHS

FNPGDPGEQGKPVTINKDNLAPAERKKYDDGWQKNAYNQYASDMISLHRSLPDVRDKECK

DLTYRDNLPDSSVVICFHNEAWTVLLRTVHSVIDRSPAHLLKEIVLVDDFSDMDHLKTPL

KDYMNKLKIVKIVRTKEREGLIRARLLGFAAATGDVVTFLDSHCECTEGWLEPMLDRIAE

NKSIVVCPVIDVIEDDSFKYQYGSAKATSIGGFDWNLQFTWHSIPEYERTRRANDILPVR

SPTMAGGLFAISREYFEHLGTYDPGMDIWGGENLELSFRVWMCGGSLEIIPCSHVGHIFR

KRSPYKWKTGVNVVKKNSVRLAEVWMDEYKNYYYERFNFDLGDFGDVTERRALRKRLNCK

SFDWFVKNIYPDLFVPGEAVASGEVRSKAKPMCIDSPVDHHNYHKAVNMWPCHNQGGNQY

WMMSKGGEVRRDDGCLDYSGGDDVIIYPCHGQKGNQEWQYKEDNTILHVNTQKCAEVSID

GKKLQMRPCSGIDRQLWQWKRKPPTGIIRDKA*FIIRSRVSSVTMQLNILSRL*LVRTFK

LTSSPHR*TGADNSCLMCISKHHEDCLSVGYILFTTSCGDICISVNGWYNLCDWMTSCGH

*GWMKT*KRRKCLKNFCIQILKHNY*WISLLYGIWKS*CSHIWAKFDI*LCTH*PSHWHM

TL**FEGISLLVQLSSICP*PYQLP*PSYGCDLDL*PHLLWAYMK*FTDLLSRLKMLCQH

LSSGETTDNIYYVKEVTMT*LSGTISQTAQVLLTH*YNAAFHLVRFA*LIIKIWSSAI*Q

KTSCILLTVV*EIN*YFLHYNHIPLKIQKNT*FIVYV*NIYVNIKFGMRAGTSILFITGR

ISKQ*HIYILQSMFRATAP*LKPLNMDSYQNVIFEGMSLFKHVLPIFLCTLS*ICGIKSI

LSFF*IQFEIFPFFMIECFSNLGSFFHVKVFYMLPLL*R*WTSKCI**I*GTSVVVCNVI

*LRNFILSRLYVHHGLQII*LHNFQIHGKARPILX

>**comp104530_c1_seq1_2** len=2811 path=[3257:0-1085 8372:1086-1095 4353:1096-1272 4530:1273-1275 4533:1276-1310 4568:1311-1329 4587:1330-1565 4823:1566-1575 4833:1576-1582 4840:1583-1879 5137:1880-1913 5171:1914-2004 5262:2005-2034 5292:2035-2127 8898:2128-2137 8908:2138-2139 5397:2140-2188 11357:2189-2239 11395:2240-2252 5510:2253-2409 5667:2410-2520 5778:2521-2557 5815:2558-2567 5825:2568-2617 5875:2618-2634 5892:2635-2712 5970:2713-2718 5976:2719-2791 11570:2792-2810]

GDRSDSHARSVG*FRMMANLLVSFVILVSIVHGNTQDVDITALNIFDALSSTLNCGQFKN

LIIASGLQVQDFFKLSQSLTLFAPNDTAFEQMLPDKRQKLFSMTATEKSDYVKSYTLTKV

LQTSSMNNNLIEKSQGALGNTNIFFYKRERRRDNIVSGLAGRTEYYANGALIYKPDVRAQ

NGIVHIIDRVLDPIVPYGVYGYIQRPEILQPTLQMKFFTEMTEYIRGDYYNQAISPLTTS

VRFTMFVPTDEAIDKIPKEKLDELRGKQPQLAAVIKQHAIPNLAVYTSFVNHNEGFQCLD

GRAVFKKNIRESVYVSAGGVNAQITKGNLTLSNGVIHFVDSLLGYVYNTAREQIQLDPSI

SQFRQVLDRSRKELQDFLVTTSGVTVFVPMNDAFTNLQNLHGVDFFNNQTLINYVVELNM

LEPGLEFAITNVNGDYQARIETPSQYRGWNIKIYSQGNDTWAEGGYVKTRVIRPDVGVTN

GFVHYVDGIFGVPYRDVPGIIYCEDWLIKSSYIFSITGLNEYLKDVNMNRMPACSYNTKR

SDYNYADPSLFNNNQNVYGTNPNSDNQPSASPPIKRGGCGESNTLCQFTVFVPNGTTIDN

FGNSYYGRQLFRDNNRLRYVLRRHIALNRKIYVDQLSRGTHVYTADTGEEVRFNKIDDKN

TEVYFMNARARVIHSDLGATNGVIHIVDDFLFVNEDMTRDISGSSKMAASRTTTFISLIV

LLYSVCL*TVICFVNTLLVPLFMTEDSRCYRISSNLFTIKTGVVKLEKSQALLLYAYAPN

VHVCFGNIDI*CCNKDWFCDLEDRAFARERKVEG*LLVLFCEKMILNVALSVVNRIQ*LV

GSGSLYSV*VFMLKWRLVSQ*FLF*NWNDCVLLWN*KRDACWSGSAQSRCALL*N*SHSA

RMEG*SLIPGSGCCKNNTQNDLSATLIVSLYLRRGVX

>**comp104658_c0_seq8_6** len=621 path=[12117:0-7 3943:8-19 3955:20-120 16071:121-141 4077:142-275 4211:276-278 4214:279-422 4358:423-461 16249:462-508 4444:509-567 4503:568-620]

WGLF*STIVRDCSFGFEF*NGGKCLTGQNMLNRRHSLTI*LAFHLFRMDGIRLSGIQPGV

SEKKIKTFLERHHLRVKKIYYPLLNNDAVITFIDSTSTSMALFERLEGLSKFTISPLPHR

IFSSVTVELEDQVAALFQTSECKKHIEQTTDGRLQIFTAGDGNVTLKGNQFDIELAEKVL

HEFLESQHKIHQDLQNGHDQSAADYEA

>**comp104776_c0_seq5_3** len=5123 path=[1:0-165 167:166-324 326:325-340 342:341-1126 1128:1127-1384 1386:1385-1403 1405:1404-2168 2173:2169-2455 2460:2456-2459 2464:2460-2601 20113:2602-2602 2619:2603-2613 2630:2614-4268 13324:4269-4529 20368:4530-4533 13589:4534-4535 13591:4536-4542 13598:4543-4640 13696:4641-4646 13702:4647-5049 14105:5050-5122]

SCVHLFPKFISSDTQHRKSSYHEDIFHFGMRRRPRKLSKLLIICVTGALVLFFGPFYFQV

PSPQTQGIDRRVLHERYQKNKVIVPCDEEVKLCGKDSIKEGAEERGGGGDEGGRIDSRPL

AVFKDGVIGNYETFDPTNRTGPGEYGEPVITKDEEKIEAEMSAREFGFNLVNSDKIALDR

ALPDIRLKECRYWHYPDNLPTASVIIVFHNEGWSTLLRTVHSVINTSPTRLLKEVILVDD

DSDKEHLKQQLDLYVRRFEGLVKLYRNDEREGLIRSRTKGALLATGDVILFLDAHCECNK

NWLPPLLTRIAHDRTIMAVPIIDGIDWTKYTYRSNYYGVHYRGIFEWSLFYKESLLPGQE

EGRRDYKSEPYRSPTHAGGLFAMDRKYFFEIGAYDPGMQIWGGENFELSFKIWQCGGSIE

WVPCARVGHVYRNHMPYSFGKISHKIPVINLNYMRVVEVWLDPEFREYFYTREPTIRGYP

VGDLTKQLEFKKEKKCKSFKWFMDNVAYEVYDRFPPPPPNKAWGEFKEKQGNRCWDTVGQ

GVNGGPIGVYYCHHGGGNQLYRLNTKGQIGYGERCIESPNGDTLHIHYCDVQPTGPWDYD

ENSYAIKNILLSKCVEIGKDERLHLQPCDPASKNQQFIVNEIFPWKDKR*RTQAEDFPAV

YSHQ*CQSSINFHLLRERGISTINSCNTVA*KSRLYVIIIIMDISNAPVHAYGAIIKIRR

YFTKVHCGFEKLVQCNRCS*RDRFLGEI*NLRQNQKERELYRHGEFM*ILIFNVTEMKV*

P*KNSVSVTDIQDEA*RYSQHITNTFVMHIFLLDWQLFVMFFIISVIDVSEDPFDKLGFQ

*AGSFKDFKLSVSGWQLGKKAILF*NDILFLKIQTQ*SLSSCQSGFKDQVSA*GL*CQRE

*TKDTFLNIFYIVVVIVVCSRVGITPCIPDNMTIVPRTIHITLGLFRII*HIYL*NT*SF

KFCLKINCLTDALLLSD*HQ*HSEVILGVQIFRSNIEKLYKP*TCNFLFLNLFKFLLFLL

KFCW*IHICDITLENMPIAVFSSSIVLCLCIYVLQSIAMGNVYN*GCTSLKSKCKYFICN

CEVVVRIHTKYCT*PLTTNPSSVFISSQLSSHDIFIPCTFC*FKTLS*THLKPINVNNNV

LIILFIWQEYLPH*YM*LVHSSKDQSLFLSHCVGLFIFSTEL**IIIR*KYGTVVLVLQS

VTLEILLWYAWK*LILCPFIYMFIMVHMIYHVVC*NTCDIYRLPVMSRCFMLRGLNPVTC

NLMTERTMVPGAV*CDSIVEILIYVETLSKMCKFMFSLTLVLRVPR*NLSQ*NGIDATSV

CLVKIFRGATWWILVFKAPFVNLNTIFVKILLKA*NHCKSFT*RP*NLSVKWGMSAASVC

LVVWDGCSRVDEDVYRPLCQLDHLIFKCLCRLLRNIVKSSIKSKVNHLCKDVEIYYTEMG

YDCYICLPSGNGRM*LGGPLRLRPHIFKSLCSLL*NIV*SSIK*KPVIYVGIHGNDQLFN

FLGIAFPGCVEFYHDCVHYTIICNCRPTLPCVHIYLGKV*FLC*DDDIDQ*PCR*LPLMF

PFSL*NHM*DVSCTKWEIFSHNM*GQFCFCRYSFWHLHDISMGV*LMIV*CMIVESMCLF

LRSHLHQVVLCFIVFMVAQEIWHDIL**FCWSSHLNNMKRMRSYL*ILR*CLRNFHHLDQ

LGTEVIQHHPDHS*HFSLVNGAVG*PS

>**comp105397_c0_seq5_4** len=2029 path=[6606:0-396 23423:397-408 23435:409-428 1219:429-695 23626:696-720 23651:721-746 23677:747-758 5923:759-789 23748:790-794 23753:795-803 23762:804-827 23786:828-879 23861:880-891 1682:892-993 1784:994-1044 23918:1045-1073 23942:1074-1103 1894:1104-1119 24002:1120-1143 1934:1144-1404 2195:1405-1455 24055:1456-1465 24065:1466-1472 24072:1473-1473 14432:1474-1596 2387:1597-2028]

WDAVAVYSRNAFVKVDGGKLVCFS*EETVPRNMREIVHLQAGQCGNQIGSKFWEVIADEH

GIDPTGSYQGDADNQLERISVYFTEAQGAKYVPRAVLVDLEPGTMDAVRSGAFGQLFRPD

NFVYGQTGAGNNWAKGHYTEGAELVDAVLDVVRKEAESCDCLQGFQLAHSLGGGTGSGMG

TLLINKIREEYPDRIMNTFSVVPSPKVSDTVVEPYNATLSVHQLVENTDETYCIDNEALY

DICFRTLKLTTPTYGDLNHLVSATMSGVTTCLRFPGQLNADLRKLAVNMVPFPRLHFFMP

GFAPLTSRGSQQYRALTVPELTQQMFDAKNMMAACDPRHGRYLTVAAIFRGRMSMKEVDE

QMLNVQNKNSSYFVEWIPSNVKTAVCDIPPRGLKMSATFIGNSTAIQELFKRISEQFTAM

FRRKAFLHWYTGEGMDEMEFTEAESNMNDLVSEYQQYQDATAEEEGEFDEEEAEEEG*IP

YKLTS*QGYLTNC*PKSY*KNCILKVLVHSFKSIP*VYPDQ*FWLSF*IFLKTTQIAFRR

WPNVRMLSGKFHQVNTLPPLAYRVRCLFWYLSLKIRSLFHCKIMPISINPILQFLFYLLK

AVSTHLTI*HCHLI*SETDILNYHSRTWKGYILLYLSQL*LFQLLFHIKHQYLKYFSTFT

ISCTKTLYGNKRTNLK

>**comp105495_c0_seq9_4** len=2286 path=[9783:0-21 1946:22-1210 3135:1211-1564 19827:1565-1565 3490:1566-1729 3654:1730-2285]

VERSVVDFS*FRDPIPREGINMDVLWLLSLLYLTHAVVCEDVLFESGKRLAVVDPATITG

GDSEYADPKVTGSSLFDSSVGNTDKLRMSLLVMVHNFRSKSSNYFRAHPVFISCLQQILN

DLQNENTRAQISSGYKTKSEVGGSTSLQDLYAQAGTGAYIEFQSGVSGDVKNIAKAALKH

CPVIFERIQRDIGIIMTATGMYIHMTGDVDTQSHFSAEPGFEISPANFPAWAQSLINEGL

DPVQSPDCSRFLGLESGQIHPSDVTTPEELVGVVDQPITRDTPQDFIKLVQYQGTNVGFS

NPERSAAWCGKEGNACVDCRNSPAGNHVSKRCASRLMSARMYNFLRVLQKLTRDNIRGDK

LRVTKAFVEPYSGQSDYSASSLHVEGRQLTLELENGGSGLKTVTKLAQCIGVDFIKHNGD

HVVLAVRKMKGYAAQKMAFQQTVLLAVEPPHSKADDYRLPVEFGESEYDDYPLFDSNGQE

DDLLAEDTTLSQFTSKDPEFRYLRLNSLIPKCYSQLLHYHNKRNADGDPATGIDVVRGFI

STKEQILTIDPFDQRYNTFILGLAMEVSYNSSHNPNSHTLSHLAQVAVDKCAPVFAEGRQ

EMGVGLYADRVFVDMRDDFDVWTDNVNLIPHEFGSIGVYKAELKIRFDAAIEKRLVDPDD

IEEAKKSANVPYPQSPLFSHTPPPHVLRRRRAAPDPDDCVPKTDTPFCEKTKQFRDKEIE

SIWTEVNRKYHYHNQEEVRQALELCMGLCGTCMDGSVYESKL

>**comp106756_c0_seq2_6** len=1159 path=[1838:0-82 1937:83-146 10103:147-148 2003:149-157 2012:158-191 9913:192-229 15787:230-335 10742:336-384 2261:385-456 2333:457-494 2371:495-514 15870:515-549 15906:550-697 2574:698-909 16033:910-917 11271:918-927 2804:928-1078 13772:1079-1158]

LPDSLNMKNPLL*QMTHMNF*FIGAHR*CPAWH*SC*ELVYACPCGSSVHGWCSTAGLLP

RTAPAVPQSYWHHGTPYPPGVAQ*SLMSIIWHIVAYDLSHSTHSGRICRRCVWLYVISSI

M*LVT*MSLGHVKQTKQAPHADYGNRIRRRG*LLIPG*VLFVVASDKTKMSVCGGASEVK

TATESQQKLCDEVKSDLESQASRTFTTYKAISYRSQVVAGTNYFIKVDVGDEHFHLRIFV

PLPHTNSPTSLAAYQAGHTAETELSYFSE*SLSERKICKQKYCRWEELLMITGIDKKHFS

LIKYLA*TIMTK*SFVFPKLCGTSWADVSLCSEHC*QLVINALM*NSHQ*HEICGFLLKL

FL**FIDIKLKIINLCNLHRQEP*IX

**>comp106862_c0_seq3_3** len=3884 path=[6962:0-1296 20050:1297-1621 8584:1622-1642 8605:1643-1701 8664:1702-1730 8693:1731-1872 8835:1873-1882 8845:1883-1992 8955:1993-2052 9015:2053-2092 9055:2093-2112 9075:2113-2419 9382:2420-2431 9394:2432-2457 9420:2458-2477 9440:2478-3169 10132:3170-3184 10147:3185-3666 20548:3667-3716 10679:3717-3856 18534:3857-3883]

KQNRTVPCGPRTDNSPHDVI*QSTRKRNMLPSGLVSVLVVATFTTVVQGNYFIVSPSVFR

PNEDFKVEITYTGSGTASAKATIGIMGGSDVAASTEQTLTNGQGLLVVPISPNVEKGSYK

LTVVGKVNGQEVFRNSTEVRLVMENSFVFIQLDKSMYKPLQTVRFRVLSLDQELKPVAQS

IRTDISIVDGNNNKLEEWLGVESMAGVIENSYQLSDQPPLGKWKITAKHKFGEDTKEFTV

EKYVLPKFEVTAEALPDYIVNGETDAINIKVTSKYTYGKPVKGKAKFIFGKGEVVKETML

NTGEDGVHTEKIMMKDVSTAKTWNGLKVAVNVTEEPAQVTETSNQLNIPVYSQRVKLEFL

PTSSQVYRSGLPVQLRMKVSNLVDKTPAHTGKQLTVTTYLGSNQEKTLTIDSQGMASTDF

DIPPSYSNDIHFTARVVGEDGISGSHQVKEYRTKLNTVLSLEVSTTQAIEVGNSFDIAIK

STATLRHVAYMVLARGVIVEGGKLNMPGTGLTHTLTATRPMSPMAKVIVYGVVDNGTMSE

VIADSEDIEVSSVFDNQVGLRFSADTSRAGTTVDLTVTSAPNSKYYLLAVDKSVLLLGTG

NDITQGDVISGFHKLGMEDTPSQNEGQPEMLGDVIMPRRGGGSESDLPTSAGEVLKRLGL

VFMTDIIVEKEARQWEFERVMDADIMFKGGVPEIQHSPVAGVGAGAAAKPKYAEVGRTRK

FFPEAWLWKDGTVSGDGTVTVSETVPDTITTWVATAFAISAEKGLGITAQSSKIKAFQPF

FVSVALPYAIKKGESFEMRVTIFNYMNQSTPVRVTLKKSDDFGVEQTVNGVKSVVSKEVV

TEVQVESNVPKGVTFWISAKTLGPIKISLVAQPDESTGEKGDSLERVVEVKPPGIVQRMV

VGETVELDAETTLYNKTLMLDFPNDVVEGSRRVEVMATGDVMGPALEGLEKLIRMPTGCG

EQNMITLVPNIVVYKYLNAVNRITDEIGRKAIQNMKSGYQRELNYKRDDNSFSAFGQSDD

SGSTWLTAFVMKCFSQARNQITVDRDIMTKAFTWMKSSQKEDGSFRDKGRVIHQEMLGGS

TGERALTAFIYIALKESESFLAETDKSSLTRAVTYLEGLVDSNQLTGTYEMAIVAYALKL

AGSTRATVLLDKLEGRTKPWEKTVEPVNSGGKVMGRQFIYHPPQAAASDIETWSYVLLAY

VHGQQLAEGRPYMKWLLTQQNGNGGFRSTQDTVIGLQALAEYAALVYASPQTDISLTVKA

NAVSGTESKVFMVQRNKLLLLQSYVVCITLFLCM

>**comp107313_c0_seq1_3** len=23161 path=[1:0-25 27:26-687 689:688-1040 51643:1041-1124 47524:1125-1126 1128:1127-2114 2116:2115-2116 2118:2117-3775 3777:3776-4372 4374:4373-4408 4410:4409-4576 4578:4577-4644 4646:4645-4716 4718:4717-6321 6323:6322-6329 6331:6330-6457 6459:6458-7628 7630:7629-8470 8472:8471-10786 52568:10787-10788 52570:10789-10866 52600:10867-11006 52667:11007-11015 11017:11016-11017 11019:11018-11018 11020:11019-11134 11136:11135-11399 11401:11400-12037 12039:12038-12612 12614:12613-12624 12626:12625-12864 12866:12865-12889 12891:12890-13836 13838:13837-14486 14488:14487-14510 14512:14511-14520 14522:14521-14719 14721:14720-14721 14723:14722-15158 15160:15159-15160 15162:15161-15290 15292:15291-15736 15738:15737-15745 15747:15746-15948 15950:15949-15953 15955:15954-17582 17584:17583-17588 17590:17589-18070 18072:18071-18108 18110:18109-19724 19726:19725-20189 20191:20190-22500 22502:22501-22817 22819:22818-22832 22834:22833-22931 22933:22932-23160]

YGD**DYTSRNDSSEHLSH*PRFCAPIQHSGVCPDSQWCDRSHVFQSGG*YW*CSSEETS

NRKPRFSV*VPGSCT*QRCTIPGAVTTSNCENYCPPQQQCPLLCW*PLLHYNQ*RSSCGV

TDPTCCCTGL*CQ*SI*PSDTSCDW**ERTNILWFGQQSNQCH*GPYFGRRK*SPLYTAH

*SKRWRVPCPFCHSVGLHNCQQEP*SSGVHYPSLQCQDTRDTTACSLCCTGSGNRWRQSC

PI*HIAV*DQRCSIQYSGTPVLYGRFGNWCCECEA*SGDRHQ*HICIQVYC*CHRWKWSE

GTTACQC*YHCGQESLSSSLYYHQLHSCHQQHHTTGLTSLHCFGH*WGFYESLQCGAIQH

HWG*WESELLQC*C*QWCGVTCTASV*HPEYTVQGATPGCGRLRSYRCGSTHCVHSEKPV

CSCLHSDFIRACHSGNNSCVHQYSQRVRPGQRPNSPLQYSQLFCKWLIRQVSCKCQQWSC

NC*TLTYW*IWPDFHIPGVCPRWWYPSTA*SVCHSSHQCNSQQFSSCVCQRAIQPVTAGN

SKCRRWCSPSYSH*CRC*VSLQ*CEV*YHW**QCPSHLPN**EQWSDISPKHL*W*WC*A

LQDPSTSTRWWQPTSV*YHCCVR*CDPKSSCTGFQHPNLQCKDFGDHKLW*SFYLSHSSR

LGHFGPQQCTDLPADW*YRMSTILWHQQQWGTVCTS*PCHRSYKGHILHLPSISTGPRQS

ATGLHQPGDSEHQCDQEHSPRVH*SAIQCCIAEKYHHWHSSRNIHSY*Q*YRCTFQ*TEL

QDTWRWQRPCIIQPKWKQCGSYQFD*PCCRYHHFIYGSS*SERWWPLCSIRNSCDGHSSD

PEPCNTSL**FIH*AQY**KLSSGWTHYYPQCN***PAGSE*RADISGPEPE*YRLLICQ

SHQWRSSYTKVFYWVNRPTV*FFGEGSRWW*PPQDSCSYCAC*CGS**PDTGIPAGQLCV

YHGGEHPCHKQYHQC*GNTRA*CDIPAYWLQ*LRRLLPCQPQ*WADNSHQGPHNRCFKAA

NFLFTS*SREAVHSQPAEGYCQCHHHSAKEYK*SYLQ*N**HL*GDN**NSCTWVQYPSV

VCSRC*WGYSSL*SDKLPGWRNRFPLPRTFQWPYSCCQTFDIHQHQEV*VHS*G*GWNES

RKILNCQCYCQCPR*QPATSIHQ*TIQFSAQLQ*PSWNPCVYCTGNRS*PHWTNELRADW

TILISTSLFPCGQNNRRSIPVFLNIN*EYINIHSWPVNF*LCSSYEKRLYQPDHHCKPES

QCSSFHNILL*QDYLGDI*SRCVCHPD*G**PRWTNPELHSRGGESYRL*FLLPARSLRT

HSGLTCSHNFLISPVHNDCENPRQWCSCKTECWSCHSNHQD**KQQCSCIWRWSIHKNHS

*DTKYWLHSFPVPSH*C*PTEH*ILTFRVQFDWR*QYWQFL*NQ*PDWCVHSPKSTGQE*

YRQICGTSGCKGWWISKQERYSHWNC*YTEEPQWPCV*PNLL*CDYI*DSRPP*QYCACC

SNRCRQIWSSEYIHLFTARLVWELCQHIFPN*CN*WQHLCPFASYPGPDRHFFC*VLSNS

H*PRCTSAIFSATCTCACQHYPQPEPPCLPE*TLQCKN*LHCSRKLPCHECAGYRC*HCG

SI*SGYLFPDW*WFGNQLVYSYA*HWCHQDSKYP*F**Q*TICASSCCHGWRNSKTHRHI

NSASNCKQKFECTTTAASKLQCWDFGHTGSLHTHPHY*CC*C*CCGTI*HSSVQANIKSI

V**VLWCGSFFWGRVCEERSSPPVRYFNLYWHCVSV*LSL*STLF*CAILCDRPGDT*PE

HTLLHQ*AIQKRPPAHSWCRYRSVDSSSSRQG*S*HNLWADSLHSHW**PSPQLLQYQPH

QWTH*DQQCTGSNLRQSLSASCPGM*PWWSV*PYHSHCDYHYKLSSSHHHPSYLYSDCAG

DHLTRNTHH*CQCYRC*SHGSKQPDYIRHI*RP*GPGMFHDQPGHWCHHCKTLITLQSMH

SISLLYASGCS*PWFPIAGKHCCFCDHQCTAQRQPSYFHQ*TIYNQSAGRCQYK*YCLHS

KCCRHGYCCSVQHHSV*SHW**HSSSLLRYPSN**QQCTDFRESTSLE**QDNLYYESPG

D*WRYS*QVRHDHCSHHSQQESVCSSL*SN*LQHHHFRDSVIGIVHLPSDCY*W*QQGNK

MATMF*FVS*CYVSIVVKLFAK*ISLILKHLV*WNGYGFVITVKFPFFC*AFSAFGYMVL

SLM*MLLIAVIWRI*SKRILMHLCHDLCVIQSPHRDVRYEATGDGTALTYFNILSNSGTL

YVQRDLRTDTQKRTTYNFVISSRDLGVPSLPGSNIATVTINVLRNLNCPAYTTLPRSITI

NQTATGVIFNVDATDADTANEYNTASIRYQIVGDDNANNFFSINPSSGQITALASISTDT

SFVYRVRVNAADGGSPSCTAVAVLTVNVRRNLNPPVFTQLNVVATILETHSVEALVSNLL

ATDFDTGNGAVISYGFETSSQWQNLFRIDANTGNVFLRRRLIGVTGNQFVMQFLATDNGN

PRLTSTPATLTVNVLRNNFPPEFLLEPYNENIRRDVGPNTLVTRVVARDNDTVSPFNRTS

FTIIGDENANAYFRMDPTTGQIFTTSAIDSVATPVFNVRVVVSDGGEPALTDTSVVRVFV

NRNLASPAFNQVQYTVRILEDQLVGLPLTPVTCTDADNFAPYNQVRYALTGNSTVLQFFQ

VNTVSNVGTVSLKRAIYDYPDNTVTQLQFQVTCEDLGNPSQAASNSATVFVTIVRNTPPF

FDRIPYSTIIPATQAADTSVFQVAARDNDVDAPFNEISLSLLGDSNTPSYFTLSQTGLIS

VRSGVNLTTDTRTNYVARVQVRDGGTPSLTSTALVAISVTRNFFAPVFSNSRVQETIPET

TPVGTTVTTLTASDRDTVAPNGNFQYRLVSGTGVGFFYVDPNNGQVVLLRSVEGLSTQRF

FTLNVEARDEGTPSLFATAIVEVSISFDPDQLVFGAGEYNITISENTPVNTIITQVQAQP

QAGVVYTVTGLSSGPDFFLVGGSTGQVRVQTLLTSDAAELTGYRLRVRATRTGVSSQTAF

TTVNIIVNRNINAPVFDRAQYLITIPDTTPLGSVLDTVRATDADGDVIEYTLLTGQPASD

FFLVGPRDGSITLKTTLIGTTQNVYQFTIQADDQKVPRQKTSTVQVTVNITRDAGAPRFI

RIPYSFNIREDVAESSSIFTVTATDDDLRGQIEFVTIGQGVAEAFFTLNTQPAQNPRESF

VIVRDTSALRNDVTQQYTLRVRAYDTAYPNNPATTDVTINIERNLNSPQFSSQRYQVTIY

ATQVLGVPIIQVNASDADGDNLMFEITGSSQAQAFYNINPLTGEIYVKRSLLEGNQLNDQ

ISLRVRDQRTPEKYGTAEVDVTIKRDRFTPEFQNQPYIRNNFPYRSNVNDNVLTVLATDR

DLEGTIVYEVTGVYPAPSFFRVNNETGLVDLSQQLTTISGDYRLRVIAYDSFYPNNAATS

TVTILVDTNPNTPICNPPVISQTLDTGSLLTPVGFVIGTVNATDADGDQISYSIVNGDPE

DYRYFIVDPTDGTLRLREPIDNSFTGFNFQIQASDRGVPTAKVCTTFVSITVQRDDAPFF

VNEPYSNTIDETVTTGTPVFRVEAQDNNLKGRIVYGLVGDLRAPYFFTINDTNGIITLRN

SIRTDTFMSYKVRVTAYDSGSTTRTATATVDLTVNRNAGQPAFGLPSYDTTIEELTAVGT

NIINVTATDSDQDVLRYTLEGNPVSVNGLQYFGINSNTGKISVIRPLTADVARTSQYVMT

VRVTDQRQPVEKSSTAQVTVNVFRNLNKPYFRNNPYSRQISELTTVSTSIITVTAVDDDL

KGDLILDVVGEGSAPSYFSLTPATRVATTRNAQVDLRNSVRSDRGQFQYVLVVRAYDSFY

PQDFVEERIVIDVTRNEFAPEYINTPYRVTIDEDTPVGRAVIDVNATDRNGDRIYYAAYG

EADTMQLFQLDPNSGVVTLIRPLVPGTQMQYTMRVNATDGGLPERWTPVEVIITIRRDQF

APIFIGNYQTNVFETQAVPSDVPIVVQATDQDGVVGQIVYKATGIYPALSFFEVDGTSGQ

IRLIRDLKMDFSKPNPYLLEVTAEDSARPRKKGTATVTINIIRNPNTPQWGLRSYSANIP

EDYPVPGPVVNVTATDLDAGDKIRYSLNSEVIQRDVSIGPIGYFYIDTETGLISLRRSVL

GSGIRQFTLGITACDNGYPVRCVNTTAVITINRDGQSPVWQNSPYNVRIQETHAAGTFVL

SVRAVDPDLKPGSQVVYEFVNRPAFFALDELSGNITLENSVLFDSNTQYNFQVRAYDRLD

KPRSVTADVTVFVIRNANPPSFLRQPYQTTISEYYTVGSEVLNTTAVDLDGDVPLYSFVN

PTNEFASKFYIDPRDGIIYLRSSMERTPITLYNVTISATDQRLVSPRSSSTYAEIRVDLD

DTPQFTQNPYNQPIQESFTVGTSVITVQAVDPDLQGDIRYGIDGLYPAESFFGINSTSGI

ISLRQDIKNEPLQLSSYTLRVFAYDTAHPTRRGSSSVLLSLVRDPNAPRFIPTDQYETTV

DVNTPIGDRIENTTAIDLDGDTVTYRVVTAFTNTLKYFYLDPDTGDIHVRAPLTSAPENF

YSMVVQASDGRGGTANATVTIEINRPRNRDPTFTGQPYNANILVTQPANSYVTRVTATDA

DLQGSIEYEAVGVYPARDFFRLNKTTGEIYVSRNLTTDPLRTPRYTLRVQAYDTAFPTMR

ASEDVVIFVNLNPNGPVFNDSEYRISIPENYPLNSMLLGVGAFDPDGDSIQYVILQDTEG

QTASRYFTVGTTSGIIFTRSSLQAAGTPNFFRFTVQAVDNGSPRRTSQIFVYVTIERDAG

APVFSQVEYQTNIPENTAVNDTQPVLTVFANDADRKGSVKYVITGDGLGMAFFWMDENNG

RLYVRRPLTSTTTSRFDMTVAAFDSEFPLKRSTARVTVYVDRNVGVPQFTAPQYSAQVFE

FTNTGTSVLRTNASDSDGDTVRYSMVLPNDEAQMYFFIDDRTGDIYVRRSLSLNTQSQYV

FQVRASDQRDPPKESDTQVVIQVLRDTQPRFIGTPYFASASESNTVDTFIFDVNAVDDNQ

RGQLVYEIIGDIPAPSYFKINSTTGVISVRSNLTTVQSRQYTLRVSVYDSDIPSVRSTQT

VIIQVDVNSNAPKFTLNPYVINISEDKQIGTLVTTVTAVDPDGDRVTYNLEATTNAGSYF

FLNGATGDIYLIRSVQNIPNTQFSLPVRAYDRPILTQEDRSQVIINIQKDQFPPEFLGTP

YRVTGVPETIPVGTVISSAVSARDPDLKGSLVFEATGIYPAQTFFDVNRNDGRINVTQDL

KNDGLGRDSYTLQITVYDSQVPDARVTENVEIGVVRNANSPQFSQTAYTRTILETHQLAV

PVININATDQDRDAVTYQLTSDLNGADGLSYFYIVRETGIIYPRRQLIGLGGRTYTMNVR

ATDNGRPTKTSDVTVNIIITGIQRPTFSSSSFATTVREDRTVTASIYQFSASKPNLGGSM

VYEVQGEGLAPYFFAVNQTTGIVTVRNDLRSGRDTQYTLRVRAYDSEFPTFWAESTLVIT

VTRNPSSPLFIGKPYSTTIQDNAGLGTTVTNVISAQDADQRDKLTFTITGDTRCQETFYM

NPDTGVVLLGKNLRVATTPSFTCSIQVTDNGFPTANVDTTTLVVNVGSIQFPQFTQNEYS

ATVDEKNPLGTFVVDVQATKNPPGNMVYEVVGEYPAPTFFGINNRTGRVTIINDLRPDGN

REYKLLVAGYDSLYPDLRSTSTVRITVNRNGFGPEFSPTSVSLNLPETTSTESWSRPINV

TDRDSSMTTCEISGTAKAQEFFAVDSRTCLLTLKKNLTTDTDRTTRYDITIIAKDNGNPA

RTGTTVVTVNVPRDTAIPIIRNLPASVTVEESAQPTDLVYTIDPFDADLDGSIRCRMIGD

WPSSTFFTLDSATGQVRINNSPKLDTMSRDEYKVRIQCYDTAWPDNRAEGLLTVNVRRNP

AFPAFTNTPYSRSIPDTFPLGSTILQLSGQDPDGDSVTFSNQGTDTDKSFYYVNPTTGVV

VLLKDLRSTDRTSFEIPVQVSDGRGKTGDAIVRITVTKNSGPPVFSNTPYETRIPVTQPV

GTRVLSLSVNDPDRVGTIVCQLEGYEPGQTYFDLNNVTHAITVRANLTLNPQPDVSYVLL

AKCFDSGNPSEVSYTTAKINVERNLASPQFSPTQYTATINDYDPKGTSVVAVTATDSDPL

APENQIMFSLSDGSRAANDYFTVHPFTGLITVSRRVSEDPTTQNRYILYVVAQDTSLTPR

SSTATVTVLVARNQAPVFLREDTYSAVVDEEAPVLNYTILTVTATDSDDSTTLNGQVRYS

ILSSGANRVFQMNPATGAISPRIDLRRVTQPIWRFDVQATDAGIPPLSDTAAVFFTISKL

GLPTFTQSEVEININENRPVNDSIYTITATDPITGSVLQYELRGDGLGPNFFSINRQTGQ

ITVSRPLIEDTQKVTVYTLRVVAFRLSNTAETTEAIVRVNVLRNPNAPVFPDQTLVFNLP

ENQPLGVEFGRINATDSDRGDNGVLDFSIDTTGVTPSYSYGYFFVNPTTGALSVITRLSE

DSNTQQYLFNVMAIDRGSPRRTASVTVTVNVARNPGAPVFAPNLYERTVPEVTPEGTSIV

DVNAADADGDTVTYTILQEPPDSYYFNIVPTTGLITLARSFTQELQDRYIIRVVASDGRS

SPRSDTATVTVNIVRNPNAPKFNPHNGTVDVSEYRPIQTVITTIRATDNDTASTLGGQLR

YSITGVSPISATDYFIISPTSGDILLAKPLANAISPNRTELTIQVTDRAARPKTDVTTLV

INTIRNMYAPEFTAASISANASERDPIGRVITNVTATDADVNVPKNQFTPNAQIEYRIIG

NAVDVRSFGIASDGSIFITQPLSNDNAEGNYVRSFKVLATDRSWNPKFSEIQVNVSVTPL

NVVLGDLGFIQPVYYKEIPENVQNKTVLFLDVVNQQNNEVTCSIISGDETETGLFTTTPD

TDRKNCYLMLMRNLDREQKDIYKLRVRVSAAQSRNKRAIGTDNAAVQTYNSWRIADIVLI

VTDVNDNNPIFKYQDIPANGYNKDQYYGAISIQAKPDEQVLRINATDLDIGPNGLVTYGR

DISQETVPPFGVTSNEGLIFTTTEFDQNPTARRQIRFGVQAQDNPTQEAPRVSTTTVVVD

LIEDYNRFILVMDKTRANEIIPNIEVVRKSIQDAVGFLTLIERVEGKRTRQSQNNIVTEM

ESTDIVFVLVGNEADYSYALMNNTNKAIQDSILSDDTLSRLNVRIGSDTSFNVERVRLPY

PQASLIRTMVTKSYIWWMDDPWAALIALAAIIILLSIVGIIVIVFSYSRYMKFIQQYRLY

QHNYGDAPEFTEPPSFLREYETQSLNMYVPPDEQIQSVGEINMTFEGDTMKGAMHTNHGE

DVAAVVNPIFQGEGHGQQQLSYNESTTIL*HVTPVLPPPLNLTFDTMTFQKK*H*SMMFT

KNPWT*NSWIYLWCYFNQITYTQKCNNMEERSLKNSYCKFCI*FYMYMYEFKSAVSSVKH

*SLPVTIIHELYLMNHAAASCLDDITDLC*TKHVVLTSL*GFFTFT*YIFL*KCRYSVV*

NLKF*NEK**NGYKQNLKHAEKRSMFVMIVIVPQLLMFV*L*MRQVSTGRMYIR*SVVYR

YIDFVHLRLYHFLSIRFVQLLYKYDILYIHE*RFYFYKKX

>**comp107337_c0_seq3_6** len=9597 path=[34099:0-11 14939:12-705 36323:706-717 15645:718-721 15649:722-814 15742:815-828 15756:829-1136 16064:1137-1166 16094:1167-1279 16207:1280-1437 16365:1438-1554 16482:1555-2008 16936:2009-2070 16998:2071-2728 17656:2729-3048 17976:3049-4764 19692:4765-4965 19893:4966-5176 20104:5177-5246 37072:5247-5254 37080:5255-5495 20423:5496-5743 20671:5744-5784 20712:5785-6893 21821:6894-6941 21869:6942-6984 21912:6985-6991 21919:6992-7180 22108:7181-7707 22635:7708-7756 22684:7757-7826 22754:7827-8851 23779:8852-9254 24182:9255-9315 37579:9316-9405 24333:9406-9410 24338:9411-9553 24481:9554-9596]

KHHVDNGSN*FFNHPSVMFCFETLLISSFKYCHKRAIHSCEGKSVFCCHGIKDLHANFW*

TRMRGSIDANVPICIAIREDDCVICSI*RSAISG*SATSGIDVSKSVKPVTISGSLLLNT

ATFLEMVIANGRPQISEANESALTMNPVRSFKVATDLYGVSSTILGNVPSVHIS*LTSAY

DFKNWRVFSGVTQLTLSDSFVFLN*P*I*SGASMTFMLRSRKTSLSFCVFLLAVARAGTC

DDDA*EWNCDCSIW**DPKLLK*PANPTSLVTDTQFTLAIMSHVLPSG*SENFPCTGLMT

PNKSHFFCTMSV*KLMAMDFQKLSVAFEISGKNR**PLRFC*STFT*NSCVQFILVFIIS

IADRYAGMTIASTMCLFQSDLWSCDPCHKFLLASWSRAKWPFTCTGRPVNTGRGRIQNAF

PSIPDPEWADLVGIGTAMPPLGSRPMSPLSQAEETEVGISLSLVNPLL*TTNHCCLLPIE

SCTSSDSFSSKGVNAGRCFLATAWLLKFSINFFCSSASPSVIFDSSEYSFDNSPPADILA

ITIFFTL*INRRESNISFLNSLRRSVIMSVVISLSEIMESSAIATVLRGNRKSVPFCCSG

THPSR*PSNASLYFTLNLSRFCIFTPGFLHVAHGTYLTCGSRAQIVLFVVGPTVMKEGTS

VRW*TALKPTPYCPVFNGSSPLLP*PRFLMPAKSASVKKLSL*THRAGPCREDQFSPNT*

VVGRDLKSLINISTVAPASSAFCSNSFIIETSQG*PDRILLILEVSFSLCPKDSIDEIWS

QRSASLFPFTPNHLAATGWILG*RNTMVSSSSTQSQSSKQSDDASCRKPCLSGSIISSIS

LLPYGSGKMSIKVFISLSMHERFSLVSFSRPVSSEID*TTAKMS*ISNLSLTPKTSNSCL

NILVWLVVT*YR*GDRSWDNRKATC*GRTIRSPTNMTHSFPSTSSVVVLCSILSSLSVSP

YQCFK*EYISKHMSLNCFSNDGFVVQLIFSATYKSCLAAVSISISSLQCSPIGVNTSSSF

LE*PG*LESMTSFEQPTKFKTLLLNISLALAKTWCGISSAFHDSSHVAGCCKIGKKGIFF

RCCMTSSVYSAIRCFFTSCLLE*SVSEM*PTIFQHLFTASCGFLW*RLATD*ALSSRDSP

GRSSLTIPSLQSISSRRGSVKPCKLVPVGKHSSSSKNIAAHFPCGCTSCPGCFSTP*GHA

GTHAVALIR*WISESRKFKAYIVSLSLSSVVQYSSQIFGKKTVEKNSKVDIHLDSNSFER

SAALKLSKILLGTSTISLSVLFQKCTAALNAMFPTTGSE*RV*LRNLIL*KLVLLTVPS*

GKRNTCASS*ATT**KLFTMGPSD*HVCGHS**KEDISD*RSTDISTSADT*DLQTASCF

NTLFQSASLSSLSSSV*VHSCLLSDVRANVALM*TGNGTCTGNGKKQK*CPSL*NPVGHS

FIS*TPFRPSSILSGRATAANGSNPFSFPEQAKFPDSL*PVAHIMSQRIVFTPTTSFSFL

VIPALNIWRWISVLSLILRPSLG*FLHAPAAYDKTLCPVNDDPTGYRIVWF*IVFFTWRS

SSG*SGSPLGGR**NLTHEIFCVKRRRFPELSANFLKNSPISYAV*DKSFISLSDA*SCV

LKGNLKRVPSHIGSLGACLLNRQPNIPSKGLNWPLIFRNILLFLTLFKLALNPGFPKCFS

CGSKITILFKLMKLGTSVRL*TELNPNPNLPTLVVSSSFVAPLSFFMLLKSLSLKLELL*

AQNAGPWSAAIFSFISDVLAS*FCLSSYMKQTSVAPASSAFCISSFGTENPSVYSRSKFL

SLRVKGSD**YSISKSSVSSITRSVKEQTPSCCAVFSDMFEWTNHISSSLPSASSEV*SL

SHSALQ*VHCSAGSRIRNSSFF*WTGR*TLSGRGWKSFSSLRICRISFRSFCTSIGLFVF

EVLKPSCFSFISSRTPVNM*GV*LW*HPQSWSNELQLLSRDGRGNLYGFRSKSLDFGM*T

DFGYWNPSSTPTQTHCVHANRSLSVSDKHWDCRKL*MFSKKSM*RLCSLLL*TLPIFFRC

CIT*CGFGCLLSPKIGSTLSGMALATGQTDPITDEYLEL*TSSEHKRGPLECPFHVKEGG

YVAGFSCKHGMHFFTLSSESNGREYLFQFLSRHLVSADAFTSASELLPV*TNSSIFLAEV

TMSPSVTPSSVLRPISFRPFFSVSVLVCLWAGNLSSIQNSFLRKSLSGLKRSHALKPSLP

FLDDKYSICETTSFVCSMIC*SLSIRNSTKSVI*SFVYNL*SIRLRIC*QTLRKDPCGTP

SFRASFLTVRDEHCMYCSGYGTGIFLSGASVN*DSTLQSLPSVTVNIANFLKTTMVLSDI

*QRLM*STKAFLSCF*AMELNFCTAVFRPVCSESFSKALITPACVGYWITFPKTCGF*RT

KLGTYFRWIISNFCSALPTFGGSDSMFVTDWAMNTLLKLCSLPFPELLTSGKIFSSLNVD

WAFIF*SLM*VHTCSSQLRFKY*EGSSALPPGKCMSRQLRSSLGNMAGVITDSPRTPSGA

RFLRRLCKSLGRPVSAN**LRPGKS*TSAGLSQMTTSHFPN*AKLPSATGSRYRSFRSSY

*SDPRRCCTRVSFVCLGSDVYLSPDLSVSQIAFAVTMFTALLN*ICNVFNSVDGTGTMFL

LYKVNISIINDRSSLVSSTRTPSSSLIQILLAGVNRNGMLWYSWS*NSERTGLQFTSTSA

TAGIIL*TCCFILSELACSREY*MIYLRMLQ*TSGSTSLTRHLFHSIALSVCD*FCS*SA

VGHLTCLRF*LKAKKPLT*TCRPVGL*LCRGSGRKQNT*P*HC*PTGTATPTYGKRDISL

QRLRKL*DISLILYTLLIIHVWLMFLEFIKSSVSITT*SLSSMDGDINSKSMSCTFEAKS

LR*CLTDVKQLLESCTQKARIFSSVAA*VWNNWTL*IELRKSSVTGASDLKA*NRSMTFS

FVYKFPERFDA*ILRGNQKRVPL*PSVKDLSENPNVPHRTRVKILFTLSDRFRNFLEKHT

LCATCSGLMKRI*SPLMMHG*SVIWNTDLKPSPNCPIF*GSSLRLVYNILTPVQSFFVNK

ASL*THNAGACKDFRNAFCFSSLASISG*IVLPSYKMSTTSAPASSAFCISSLSICPSSG

YWSSTDLSCLMRVGSCMQV*PLSSESESRLMVLPPSHLEHSLLLTTSVHAIVSERY**LH

A*YRLRLERVLSYVHLQQS

>**comp85993_c0_seq3_1** len=269 path=[1:0-25 2598:26-29 1290:30-56 1317:57-133 3005:134-185 3038:186-199 2517:200-215 1475:216-235 3070:236-245 3080:246-268]

KQLEDGRTLSDYNIQKESTLHLVLRLRGGMQIFVKTLTGKTITLEVEPSDTIENVKAKIQ

DKEGIPPDQQRLIFAGKQLEDGRTLADYNX

>**comp109254_c0_seq1_3** len=1231 path=[1209:0-1230]

RPHLLVQCQPRPVDKMVKVGVNGFGRIGRLVTRAAICSGKVEIVAINDPFIDLNYMVYMF

QYDSTHGKFNGTVKAENGKLVINGKPITIFQERDPTNIKWGEAGAEYVVESTGVFTTMEK

AGAHLKGGAKRVIISAPSADAPMFVMGVNHEKYDNSLKIVSNASCTTNCLAPLAKVIHDN

FGIVEGLMTTVHAITATQKTVDGPSGKLWRDGRGAAQNIIPASTGAAKAVGKVIPELNGK

LTGMAFRVPTPNVSVVDLTCRLEKPAKYDDIKKVVKQASEGPLKGILGYTEDQVVSCDFN

SNSHSSTFDAGAGIALNDNFVKLISWYDNEYGYSNRVVDLMAYMASKE*ETLDHPPQQGH

*AREALSQLGPQH*ASPSQFPSQTPIITGGA*GALPTLLNTINKVRCTHX

>**comp111239_c0_seq1_1** len=1999 path=[1977:0-1998]

TRSEKLRESETLSASIRRADPAGAAAAMDDREDLVYQAKLAEQAERYDEMVESMKKVAGM

DVELTVEERNLLSVAYKNVIGARRASWRIISSIEQKEENKGGEDKLKMIREYRQMVETEL

KLICCDILDVLDKHLIPAANTGESKVFYYKMKGDYHRYLAEFATGNDRKEAAENSLVAYK

AASDIAMTELPPTHPIRLGLALNFSVFYYEILNSPDRACRLAKAAFDDAIAELDTLSEES

YKDSTLIMQLLRDNLTLWTSDMQGDGEEQNKEALQDVEDENQ*DVIKANKRNHL*LPLPP

LPLEVPHCH*EPPNLTFTFGLRI*VPALLFSFFFFFFSPLPFFKTNKQTNSFQKFLRQE*

ISVDFTGPSFRFFTTLTGLHRGFFSITVLSPATLARSSLEMEMTFESH*TSR*CI*ERLI

TQ*RHMRCHFSFF*LLN*ILYQCLNLNWVLA*GVLGEFVVMVLL*TVFGTLLKCC*KAWC

W*QFNNPWLLILADSSPSEAG*H*RWYGSLHACSTLFLLPPPRPPSSPPSLAQPLLFSTC

NLKLICTTGYLTGAAGTDLYCSY*NTAWN*H*T*IKQT*IKNAKYIMPPSFILLK*NL*S

RHV*QYCLSECEISPILSHKSFVFQ*CVFGGKGGLLVIWC*TTKSVIVLSFKPVSNTIFM

FKFIIKX

>**comp111388_c0_seq1_4** len=1244 path=[1222:0-1243]

QFQHLFLIPKLDNTQGKKKVRQNSK*MYFACHSQSTGCGSLDSVF*RNLRLQPHSGKTIA

NDEQYLTIYSTQKKPMAPKLAAKLAVAVPLGPMMGPVKAWGNLERAFWWYLAQKVKGILD

QGSPKSPSSSQVSSSNSMSSQLQGPDTASSPIPMRFLASARASASAAAASRASKASSAAS

MNCAVQSRGSLF*TSAMNLSTFILLVSW*ERRPQRNSYSGGLLLGTRL*SRYFCFTNS*V

RSFLRSPRRGCLTPGRSPILRSASQRTASVARLPFMKIMPRITKRRPSFGVSLVVPSMPA

RDSGVLMRIYRCSSLSISFSWIPNFFSRTKHARSMISG*TSVYSRMISLSISERLMGTFV

*SLSIRYLTNLFALS*RRATSRGCGGPWFCEARELEEDLRLGEGRKFCQSGGIG

>**comp112534_c0_seq1_2** len=422 path=[400:0-421]

SMFMMILACVLCLCAAGVQAIPKYLDRIPNGYLVPDPCNPRLSWRTVGHSNLMGGKRSLN

AFGKDFRAEGYIWTVALCRKDSDGDGRNNGDELGDPNCTWRFNQSTASRNISHPGIVCSG

PRTRHRRN*RVYLIW*INLRX

>**comp114698_c0_seq1_3** len=444 path=[1:0-443]

PPKVSSKGAKKAGKAKAARVGDKKKKRRRKESYSIYIYKVLKQVHPDTGISSKAMSIMNS

FVNDIFERIAAEASRLAHYNKRSTITSREIQTAVRLLLPGELAKHAVSEGTKAVTKYTSS

K*TVH*NLYLHPTVLFRTTQISQKNT*X

>**comp118163_c0_seq1_4** len=717 path=[1:0-716]

PRCSPLPHLRAFRPHGVAKMSLSNKLTLDKLDVKGKRVVMRVDFNVPMKNNQITNNQRIK

AAVPSIKFCLDNGAKSVVLMSHLGRPDGVPMPDKYSLEPVAAELKSLLGKDVLFLKDCVG

PEVENACANPAAGTVILLENLRFHVEEEGKGKDASGNKVKAEPAKIDAFRASLSKLGDVY

VNDAFGTAHRAHSSMVGVNLPQKAGGFLMKKELNYFAKALESPERPFLAILGGAKVADK

>**comp121020_c0_seq1_1** len=949 path=[1:0-948]

GMAFTFLKVLNNMEIGTSLYDEEGAKIVKDLMSKAEKNGVKITLPVDFVTADKFDENAKT

GQATVASGIPAGWMGLDCGTESSKKYAEAVGRAKQIVWNGPVGVFEWEAFARGTKSLMDE

VVKATSRGCITIIGGGDTATCCAKWNTEDKVSHVSTGGGASLELLEGKVLPGVDALSNV*

YFLSCLWFLCS*VNLVFSTSPFGVSARFS*WLRCGTRNP*TVAQHLSSSLLHQDLSVFFK

IPFKFLSD*NHCAL*RASIYILPEKGSEL*RLSSLSDVCSLWLASSLTVLDSAWQSDEIP

AVSLQKLMIY*TIKMSX

>**comp133740_c0_seq1_1** len=1438 path=[1416:0-1437]

ATRAIPELTKLLNDEDQVVVNKAAVMVHQLSKKEASRHAIMRSPQMVSAIVRTMQNTNDV

ETARCTAGTLHNLSHHREGLLAIFKSGGIPALVKMLGSPVDSVLFYAITTLHNLLLHQEG

AKMAVRLAGGLQKMVALLNKTNVKFLAITTDCLQILAYGNQESKLIILASGGPQALVNIM

RTYTYEKLLWTTSRVLKVLSVCSSNKPAIVEAGGMQALGLHLTDPSQRLVQNCLWTLRNL

SDAATKQEGMEGLLGTLVQLLGSDDINVVTCAAGILSNLTCNNYKNKMMVCQVGGIEALV

RTVLRAGDREDITEPAICALRHLTSRHQEAEMAQNAVRLHYGLPVVVKLLHPPSHWPLIK

ATVGLIRNLALCPANHAPLREQGAIPRLVQLLVRAHQDTQRRTSMGGTQQQFVEGVRMEE

IVEGCTGALHILARDVHNRIVIRGLNTIPLFVQLLYSPIENIQRVAAGVLCELAQDKEAX

>**comp135434_c0_seq1_4** len=334 path=[312:0-333]

AIMSGRGKGGKGLGKGGAKRHRKVLRDNIQGITKPAIRRLARRGGVKRISGLIYEETRGV

LKVFLENVIRDAVTYTEHAKRKTVTAMDVVYALKRQGRTLYGFGG*KLCFH

>**comp22593_c0_seq1_3** len=735 path=[1:0-734]

LAEDLVQVHLQRNPSIMILQAALFLAGLTVVSGSICCPPKQFNAFQYVTFVNSTTTLRAL

YVIIYDGVNQRYLITGDRSNNKLVGTTKVIYDYKKRIAYSIDAGARTCTKFPVEGNFEDQ

QYVCVPSGAESVGPLFYGYDQSRLNSQAYVYNSTAPDGSFQNVVTTVSQDDCVPIVICAT

TTGGPGGNSVYTVGYNDFYPGIRDITVFDIPPYC*A*YQHC*KETQGSELQLYNVYLARY

SCYDX

>**comp23247_c0_seq1_2** len=1455 path=[1433:0-1210 2644:1211-1454]

GANAARAPQPS*IGCVLLERAAERGSGDPSRVRSLVLSPTHRRTRQTHPVLCQESTATGT

MPHPYPALTPEQKKELSDIAHRIVAPGKGILAADESTGSIAKRLQSIGTENTEENRRFYR

QLLLTADDRVNPCIGGVILFHETLYQKADDGRPFPQVIKSKGGVVGIKVDKGVVPLAGTN

GETTTQGLDGLSERCAQYKKDGADFAKWRCVLKIGEHTPSALAIMENANVLARYASICQQ

NGIVPIVEPEILPDGDHDLKRCQYVTEKVLAAVYKALSDHHVYLEGTLLKPNMVTPGHAC

TQKFSNEEIAMATVTALRRTVPPAVTGVTFLSGGQSEEEASINLNAINKCPLLKPWALTF

SYGRALQASALKAWGGKKENLKAAQEEYIKRALANSLACQGKYTPSGQSGAAASESLFIS

NHAY*PELN*GCSINTPGPCLPTCY*RGVFRLFPITLAAALVCGVVCEC*ICHPFQPTAN

KQLFX

>**comp241021_c0_seq1_1** len=240 path=[218:0-239]

GDDDDDNGGYAFLRRALARASARARAAASAAGRSRGGSGRSGGSGGSGGSGGSGGSGGSG

GSARARASASASARASSGSG

>**comp25997_c0_seq1_1** len=264 path=[242:0-96 339:97-263]

NRDEDSFLKGYALRHGRASRYRLGGTDLNIEGRWLWEGQRRMDYTRWQPGNPSNSKGREH

CLELLNEHGKEIWNDNQCDIPLHFICEK

>**comp26600_c0_seq1_4** len=660 path=[638:0-659]

RLCCFCALPPAMASLKDLEGKWRLMESHGFEEYMKELGVGLALRKMAAMAKPDCIITCDG

NNITVKTESTVKTTVFSCNLGEKFDETTADGRKTETVCTFQDGALVQHQQWDGKESTITR

KLKDGKMIVECVMNNATCTRVYEKVQ*GLPRHPGQELAARVNMLNSVSGQMQQTASLLWF

YFSWLLSSLYHKHFTWTFMSNLVYPGSFLWLVNKRVSANL

>**comp48128_c0_seq1_4** len=1730 path=[1:0-117 119:118-1729]

GLPSERRCCENHR*FKAKMGKEKTHINIVVIGHVDSGKSTTTGHLIYKCGGIDKRTIEKF

EKEAAEMGKGSFKYAWVLDKLKAERERGITIDISLWKFETSKYYVTIIDAPGHRDFIKNM

ITGTSQADCAVLIVAAGVGEFEAGISKNGQTREHALLAYTLGVKQLIVGVNKMDSTEPPY

SQKRYEEIVKEVSTYIKKIGYNPDTVAFVPISGWNGDNMLEPSANMPWFKGWKVTRKDGS

ASGTTLLEALDCILPPTRPTDKPLRLPLQDVYKIGGIGTVPVGRVETGVLKPGMVVTFAP

VNVTTEVKSVEMHHEALSEALPGDNVGFNVKNVSVKDVRRGNVAGDSKNDPPMEAAGFTA

QVIILNHPGQISAGYAPVLDCHTAHIACKFAELKEKIDRRSGKKLEDGPKFLKSGDAAIV

DMVPGKPMCVESFSDYPPLGRFAVRDMRQTVAVGVIKAVDKKAAGAGKVTKSAQKAQKAK

*ILPLTPATPVLISGGRTVSELFVSIGHLSLIVKDWLMITMHRKTFRRKECCGPFFLCVA

VLSY*FSKSVLFNGNNLTKNLSQNFETIKTSLMRKK

>**comp51700_c0_seq3_3** len=645 path=[1:0-572 574:573-644]

GSFCL*DLWLDPADSKMSSGNAKIGYPAPNFKATAVMPDGQFKDISLSEYKGKYVVFFFY

PLDFTFVCPTEIIAFSDRADEFKKLNCQVIGASVDSHFCHLAWINTPKKQGGLGPMNIPL

ISDPKRTIAQDYGVLKADEGISFRGLFIIDDKGILRQITINDLPVGRSVDEIIRLVQAFQ

FTDKHGEVCPAGWKPGSDTIKPDVNKSKEYFSKQX

>**comp52010_c0_seq1_4** len=919 path=[1:0-918]

LGLSRF*DLFPVSTRVWTSVCPALAHAVMASGNAQIGKSAPDFTATAVVDGAFKEIKLSD

YRGKYVVLFFYPLDFTFVCPTEIIAFSDHAEDFRKLGCEVLGVSVDSQFTHLAWINTPRK

EGGLGPLNIPLLADVTKSLSQNYGVLKNDEGIAYRGLFIIDAKGVLRQITVNDLPVGRSV

DEALRLVQAFQYTDEHGEVCPAGWKPGSDTIKPNVDDSKEYFSKHN*DG*TSVSLKLGFH

LCPNLDVLCWPRKC*IFLHSLKGLESRLRLSHYPPGIW*IVILP*AHLAGPRSIGNQ*SI

RDSVKK

>**comp52213_c0_seq1_3** len=2417 path=[2395:0-1708 4104:1709-1900 4296:1901-2416]

DGSARGVAV*GLGYIETYGTVAVIQCPGVRKMNRIHAVVLLLVVWTARSSSLLWTLEAEG

TQGQHTRNYRSEASGHQSVLLYQGESILFSICFMRRTNLTVKDVVFSNDGHSDNVGVGLD

NVAIGSFRTHRHDTGGRSWNKFYHSGELGSIGVGAGINRLSIYANETDRWGLEIDRVVIE

IADDHLNADILHCRLPCVQDPPVLHNPQRESVSSGYITQKSYPTSCAEEDNVHIPVFHDS

VSEYTISATLPMYHSFRNDVYQDFDNCSFAQTVFWNYTDVKLSSLLVQTDPNSLSVLASR

VNSQGIATIHLGLLFYLETPEEGVYNSEMGGIISLSLSGIMDVAMVTLLCRGKFNRYTTL

QTYQFTNTSDTFEWEVPDYVLGANANHVILVIVTSHPETITVNHFYIKRRTFEEDSMFEI

FKGDVIIEGLHVDFWWQQNTTMEVTLSTTSQSWTAHYIRFYVPVPWSNNGWCQIMVIYQD

GNVRLLPMTPRGASWIPFGSSVLIGQSDKSMRPSAPISKVTIDPIHLSLDVVYADGGTAT

MTLYPLVERTELRITKIKMAGDTTTNPFATFRSMYVAGGNADTDSVATDTHDVVHVMDDW

GNITGTVFGLFRRCQSSHLTQSPDLNLRIVKT*NDFTPSIVDTCL*RLYTLGLNLA*YFY

VNMLIWPL*LLYFSTF*INKY*YQVLPFESRRENL*NNIQKWTTINFTLIRVYMMLFIRK

SRMTSVLS*CFLDASMLYSDVVKLMLHECVLNMCTDMRSCHL*YEYKVYNVSCMVLRPRY

VSTLGTNNAHHINHGSVRSVRHLLI

>**comp52297_c0_seq1_2** len=813 path=[791:0-812]

VTANGNVFNMNCAPGTLYNATSCKCDITTTPPDPPACTEELYIPCNVDISDHSGNRLWVA

NTNVTLATVPGRSYKACSFDGSADLSVPFFKNNDLGDNWTIRFNFRSQTGSTGVRVLVSN

SVCNSISPTLEVIHAQDRVCVNIKTNSNHFTLCSANTGIPPPNLPYLPVTVKVNKGKLIL

VNGLHANSIPINGKVKLTKCPLTIGKGYNRLNYVGLLWNLQVWKCNPDSFTNVQQLTPAT

PLP*QCIRPDPRSSRKPHLHRAVHHSNQITX

>**comp52564_c0_seq2_5** len=1810 path=[1:0-283 285:284-1750 1752:1751-1809]

PQRSYDSQPPRPHSLVAVFPAAASQPTFEAMSTRSVSSSSYRRMFGGSGTSSRPSSNRSY

VTTSTRTYSLGSALRPSTSRSLYSSSPGGAYVTRSSAVRLRSSVPGVRLLQDSVDFSLAD

AINTEFKNTRTNEKVELQELNDRFANYIDKVRFLEQQNKILLAELEQLKGQGKSRLGDLY

EEEMRELRRQVDQLTNDKARVEVERDNLAEDIMRLREKLQEEMLQREEAESTLQSFRQDV

DNASLARLDLERKVESLQEEIAFLKKLHDEEIQELQAQIQEQHVQIDVDVSKPDLTAALR

DVRQQYESVAAKNLQEAEEWYKSKFADLSEAANRNNDALRQAKQESNEYRRQVQSLTCEV

DALKGTNESLERQMREMEENFALEAANYQDTIGRLQDEIQNMKEEMARHLREYQDLLNVK

MALDIEIATYRKLLEGEESRISLPLPTFSSLNLRETNLESLPLVDTHSKRTLLIKTVETR

DGQVINETSQHHDDLE*KLHTLGATVQYQQEGKKNRILGKQLSSAFTAVFQERKIDLE*K

EAQHLTTDTPKDVEKVYKII*FTKKSCARILFKVFLNTIKTAFFQ*ISDQLVTASINLQK

YKKX

>**comp59223_c0_seq1_2** len=530 path=[1:0-47 49:48-413 415:414-471 1077:472-529]

WD*LPHPMMTSFLKIRGWDSVTECVEVGTGIFFYPSYNIGHSNIRVSQYPVGLTPVSGSD

KDGIFQCSFRRKKVLSGDDRFYDLNKDFYLLVASGRATPEGNKIQHTWRPWISSVTIDLQ

KHTSTHVNTLPAQPPRPQSNLKTKQQHSHHTLPL*PSVQP*NKAATLTPHPSSLAQX

>**comp64272_c0_seq1_3** len=1172 path=[1150:0-1147 2298:1148-1171]

GGVVQRQARPSYQSE*FLEAELCITLTDKSLGSKDCGLHSTVYRMEKSIWRGCLACLAFA

VTLSLTSAQGTCYFAEVPGNNTVYLDTYSGKIMPCANGTVFNVKTCTCVHGENTFKWCRP

RFAMDFNSKRIRNKVIRYNIGGGETLDLYEGFAHFTPSSSAIFWTFEDRPFDPNLQVQMR

FKDDPTSKAKKQVLLTNCDAKDPPGQPSLLILLDKTQRNHGKKDLVRVRVRTYNMSQGVE

VVVPFKTGEFNTLVVLKAMDALSVRIDYEFGGRTINSNRLIGDLVKSKSALRLGSCAGQH

NGFVGVMDWFDYYPCWPKV*RRETTAVLSSQPNLY*SHFKTQTCRVRDDFTVPFRFL*SR

SVLPT*RNIFSAETFFIRCPLHTNEIKQYW

>**comp64599_c0_seq2_6** len=1464 path=[3017:0-1301 2878:1302-1463]

PDQGQSLSLAATAAAAAAQRLPSRVRVPPPTPDTEYPVMDKNELVQKAKLAEQAERYDDM

AACMKSVTEQGAELSNEERNLLSVAYKNVVGARRSSWRVVSSIEQKTEGAEKKQQMAREY

REKIETELRDICNDVLSLLEKFLIPNASQPESKVFYLKMKGDYYRYLAEVAAGDDKKGIV

DQSQQAYQEAFEISKKEMQPTHPIRLGLALNFSVFYYEILNSPEKACSLAKTAFDEAIAE

LDTLSEESYKDSTLIMQLLRDNLTLWTSDTQGDEAEAGEGGEN*PAFQPLSASF*NLHSR

PFVIHAVPQIVFLFTIYDRFMLLLFEFLYFPCGFYVLILGE*SQLTLGSYSFSS*GGQYG

MWNFYMSYTCLA*YFWYIVASEGPVLKLLPCLSKENCLHIGVCWRGIIGIMGSSHECSLC

GYCKAWSTCEAVTQTPCGCTLGSCVSACTLDHSSRSCL*TKS*PNLLW*GQKRFTFHYL*

SYLLFAFX

>**comp68339_c0_seq1_1** len=356 path=[334:0-28 363:29-103 438:104-268 603:269-355]

GRVKLWAHVRANQHKMNTLTAWVAVGLVSTLMCHILAVETIDCRKSCHRPASTTTLDGEE

FCCPRTFPRLRSRLTNINGVKSVSCKCFKIRDA*IYFPFDSVFN*CLSDSLSLRCIICX

>**comp68339_c0_seq2_2** len=249 path=[667:0-161 603:162-248]

TIDCQIGQTGCRKAASRITLDGEVYCCPPQFPALRSNLTNINGVTSGSCKCFKNRDA*IY

FPFDSVFN*CLSDSLSLRCIICX

>**comp70759_c0_seq1_2** len=449 path=[1:0-87 89:88-101 103:102-218 220:219-370 372:371-448]

SRPSIVINSCKTVCKMMLGVFVFLSLALYSDALTCTSCEDVACTTVGLPCQPPDYVYAPC

GCCPKCPLELGQPCGSLTQRCQFDLWCFKSSGNKIEAYKNVPNFPSFKGVCAHLDISTN*

NSPVSVKKKTSDHVKTHKQTDYSLFQSLMX

>**comp73608_c1_seq1_6** len=644 path=[622:0-643]

KRASANASSRRFLTISKMESSWIYCVICALVIFPYGAHSRRPREEEQSCGSGWVVMHRAC

YKFDTRKMSWEDARNACRQEGGTLISISSKAEKKKVVRELKKQIKKDDMLTWWVGLKWHT

DLNKFIWQDGGIFIKRITQWGPGEPNNGRGRSVEHCVEIRFNGFLNDRACETLRPFICER

HEITQRPTFQTTVEAVTSTSTTIATTSATTASTTT

>**comp79549_c0_seq1_2** len=2081 path=[4331:0-15 2061:16-1969 4016:1970-2008 4058:2009-2080]

SCRAFVWSRRQRSWAYTQATMSKGPAVGIDLGTTYSCVGVFQHGKVEIIANDQGNRTTPS

YVAFTDTERLIGDAAKNQVAMNPTNTVFDAKRLIGRRFDDAVVQSDMKHWPFMVVNDAGR

PKVQVEYKGETKSFYPEEVSSMVLTKMKEIAEAYLGKTVTNAVVTVPAYFNDSQRQATKD

AGTIAGLNVLRIINEPTAAAIAYGLDKKVGAERNVLIFDLGGGTFDVSILTIEDGIFEVK

STAGDTHLGGEDFDNRMVNHFIAEFKRKHKKDISENKRAVRRLRTACERAKRTLSSSTQA

SIEIDSLYEGIDFYTSITRARFEELNADLFRGTLDPVEKALRDAKLDKSQIHDIVLVGGS

TRIPKIQKLLQDFFNGKELNKSINPDEAVAYGAAVQAAILSGDKSENVQDLLLLDVTPLS

LGIETAGGVMTVLIKRNTTIPTKQTQTFTTYSDNQPGVLIQVYEGERAMTKDNNLLGKFE

LTGIPPAPRGVPQIEVTFDIDANGILNVSAVDKSTGKENKITITNDKGRLSKEDIERMVQ

EAEKYKAEDEKQRDKVSSKNSLESYAFNMKATVEDEKLQGKINDEDKQKILDKCNEIISW

LDKNQTAEKEEFEHQQKELEKVCNPIITKLYQSAGGMPGGMPGGFPGGGAPPSGGAFQAP

PLKRWIKSVQEGVALFHRDPKQVTWNNKTI*IGX

>**comp79626_c0_seq1_4** len=1656 path=[1634:0-1091 2726:1092-1132 2767:1133-1655]

L*RHTLGKTGTRRRMNMDLYITGHRWFIALVLMTICTTEVISQGSHYKRVCYFTNWSKYR

LGQAKFTADHIDPFLCTHLIFAFGDFDDNGNLRKLDSYHDSKDFETFKSLKQQNTELRTL

LAIGGWNFGSIKFSKMAGSPMMRKTFVSSTVSYLRSNGFDGLDIDWEYPAERGGVPADRR

NLVTLCKELKDAFTTESQQAGRESLLLTAAVPASRYKGEIGYDVPALNNYLDFFSIMTYD

FHGGWENKVGQNSPLSASPIEDGDDKYLNIREAIKWWHRAGVPKHKLVVGLASYGRSFQL

TSSSYGLGAPAAKGISGPYTREGGFWSYYEICMEIDNDYNGAHRGTVYYDTDQEVPFYVK

DNFWVGYDNERSIKAKIKWMKERDLGGFMVWSLALDDFNKMCNYTTRSYPLIHAAIEELG

NVVMPAPIPRPTSRPTAPPRPRTTRPRPIGGAFRPKPTVVVNHQTPGVQPFVPSGKFIEC

PSRGHHYFPDHNDCQKYIECHEGQRHSRGCPSGLHWDVKVDACNWEHLAGCTTG*TWDGD

GGWSRRWRMGSG

>**comp80185_c0_seq1_5** len=1716 path=[1:0-517 519:518-558 3443:559-568 570:569-864 866:865-889 891:890-1715]

GTGSRSRLSLRLSSVSTAPSFLALQRSYCQKFAMSILRIHAREIFDSRGNPTVEVDLYTA

KGLFRAAVPSGASTGIYEALELRDNDKTRFMGKGVSQAVEHINKTIAPALVSKKVNVVEQ

EKIDKLMIEMDGTENKSKFGANAILGVSLAVCKAGAVEKGVPLYRHIADLAGNPEVILPV

PAFNVINGGSHAGNKLAMQEFMILPVGASSFREAMRIGAEVYHNLKNVIKEKYGKDATNV

GDEGGFAPNILENKEALELLKTAIAKAGYTDQVVIGMDVAASEFYRSGKYDLDFKSPDDP

SRYITPDQLADLYKSFVQNYPVVSIEDPFDQDDWGAWQKFTASAGIQVVGDDLTVTNPKR

IAKAASEKSCNCLLLKVNQIGSVTESLQACKLAQSNGWGVMVSHRSGETEDTFIADLVVG

LCTGQIKTGAPCRSERLAKYNQILRIEEELGSKAKFAGRSFRNPLAK*SMDRRSLEPPAA

RSSIPDVIPAAQGQPSACPSHVTASLGIHT*PPESLLETQFCNHVIGLNHCFCHLTSQLV

SGALGTPV*SLGCPPSRSPSPQWFTCKNKSRX

>**comp81445_c0_seq1_1** len=1214 path=[1:0-682 2967:683-1038 1040:1039-1178 3014:1179-1180 1182:1181-1213]

DFSHILLSPTVHLYPSNQQSDMSPWDFVVCSSHNALFSLTQWSIIPSRHMKIIKIPL**R

KSIACFHKALKYHRDLASK*QVAM*MPL*EQLLDVDGMK*TVS*GCSCMCVAGAWGL*VR

GGGQLGCHLLT*FTVT*GAMPYRNLIFGCFFKYR*RV*VTKRKER*VPKPASTCHHACSS

VTGPATPRTFFLAYLRCPQKVLSTFHNRKPMP*DTVDVRNSVRECSYFNIVLMAHARMEN

TINP*ATSWNTGFLLTRYTMVTILQ*RNSNTFSPVLSLCGGLDSVAYAYTMNADPHIMGS

SSRSCMLYRSVMWKNQDPIPTTKSPR*HQMMRASSLSL*AITAPQSGGMIIIKLLMVFQN

SAMKGSTT*FSSHQSSVEVTGPQYPSVGAMFDCGNGNAIDSSSRV

>**comp82154_c1_seq3_3** len=1594 path=[1:0-26 28:27-28 30:29-1120 1122:1121-1593]

SQLAGVTIEISVVLTRRMQYTVMVMCAFVAAVAAMPQYVEWFEIEPAMTVTKARVSFAPY

TNEWVSFKKQHGRFYEKHEEEARFEIFKQNLQYIEQHNKKFSLGQKSYYLGINQFADMKN

EEFCMFNGLWREYNRSRDVQCSKHLTPEYLVAPSEVDWRKQGYVTPVKNQGQCGSCWSFS

TTGSLEGQHFRKSGKLLSLSEQQLVDCSGKFGNEGCNGGLMDQAFEYVITNGGIETEAEY

PYDAKQERCHFEKSEIAATASGCVDVKSGDEKDLKNSVAEVGPVSIAIDASHQSFQLYSG

GVYDEPDCSSTELDHGVLVVGYGTDDGKDYWLVKNSWGTTWGLEGYVRMSRNQDNQCGVA

TQASYPLV*SQDSMENLLQPSVIIPLV*LQPDNMFMCKYYKSFC*QANITKAQFYLLCHE

NWYKKKISCCENNEAV*LLSEIYMP*KSMYVCFKFLQCLMINVSSIYVCVLFEIGMYTRN

SYQQ*ALESLSISLGYFYHFIVHHDWNFLQ*L*EYK*L*LMFLIKKF**KX

>**comp82638_c0_seq1_6** len=2489 path=[2469:0-1041 3511:1042-1521 3991:1522-2488]

RRAGCWQAVGKFDRLWDSDCTELFPCCV*RCGLVLWSTCWCDIMDISVLWRALTSGHLVI

TWLLLAVITGGRTQHTCNQCDCTISDPDNGEKAIINVKPKCLEGQITWLSSYGAIRLELN

PQHAGEYRYCVRVQSENIKTIVSQEVLSRTHGNYKLKIVNDVEVRLSPLFTTRGRGREFC

ISGSDPIHLYMETERSAEDTGVARVKVFYDMEKTSSSLRYSPMEECRPCTDRELLRAYCT

SDFVAVGSMDDVNHDDVEETSVIRVSVTRLISQKEAIFKKAENGWSRPHELKGGIHAPHR

CGIRHGVGQFLFTGRVRLGRPVLQCAPRYEQWKSVFQMALLNDELECSYGS*SERGPHLI

MCHIHTRLFTYSDLT*PKQWTHRVCSCAT*VVRRNCLCESVIHCFNSPNLEPEVHNVYCA

WPDHVTDSMILYIMCSLNL**PIVRTLNPQQDLDQ*KMAAVSHCDCV*RWRPDGI*TDSL

GRVHYNMISSVLVSVESLLS*CHSCFESAANRFDQTKCI*NRDLSCSYKVVYL*TIYCSC

LLQRQN*LSAHSVGDTMHSLCIPFGRRHSRTFKTYSCSHLNSLHCRTFYPHSFISWDHLV

SGSTEKFQKCPYSKFPSPSTQSKPLLNQTSYQLVPRTYHTPLTFPQLVLWLQCCRGAVMQ

SLENINLLQSTSLQP*LHPHATHPHTALQHQQQHPSSNMMTTSI*NSIIYMYIITRQNCN

ILNVAPIPIPLHPHENEIFTHSLLLLK*PSRSQAFAENATVQMLPETLIAF*QFYD*NW*

LNSHLSSAQPRAVCAKLILNGIYVTNSYAFIQSLGIGWSDLLTWLTHVIV

>**comp8431_c0_seq1_4** len=630 path=[1:0-629]

PALTKPDTVTRTQANSSDFILKDIPAAIITGDPGAKRNKLSELLEKSKSSYSVPLPELPN

VELTALYSVHSDVTEGNILKTDKTVQKTLSSKVEPTTSVPSSTPTTTLNDNASTAAPATN

TAEMRKQQINDQINEQLSVLNRLHQRQKIIEHQHQLREQHTRLQKQRDILRQQLQTLQQL

RDRNMGINKSSLPLPGGPEGIATAWEGIQL

>**comp84928_c0_seq1_4** len=593 path=[571:0-73 645:74-129 1852:130-130 1387:131-175 1872:176-361 933:362-413 985:414-445 1017:446-450 1022:451-592]

CPTFAVTHLGISKGRNS*QCSASPQTTMPPLSFCQLGLVLVLVGLTSAEYHDVCQLPKDT

GPCRAHFPKYYFNSRTRLCEKFVYGGCLGNANRFETLEDCRRRCGGGDLCSLPRDPGPCK

AAIPRWWYNKRTNNCQRFNYGGCPGNANNFQTLYECRFQCRRTSTY*SLHILKLYSVVTP

ASVKGKLYVVTNKTCLK

>**comp85406_c1_seq2_1** len=792 path=[1:0-591 593:592-791]

KG*IRKTAHSALRKKFNTHILFHGHVIHHAYNFQRPKINPFH*KEY*YP*CLLKNKDHCN

VIFRPQQLYE*ILSALHVVYKKSRNTKYQYNDALFHYRVISFGVQYYVSK*SVKSNDLIV

TETVKPPPRVHELPTGISCCLDAALPFWGGTKESEAEYETNTQGNSSSGRRGRQERGIME

SISILAVVAIGVITVCQAGETTVTKKVYFDINIGDESAGRIVIGLFGETVPLTTDNFLQL

ATGSRGFGYSGSIFHRVIKDFMIQ

>**comp85674_c0_seq1_1** len=1364 path=[2827:0-66 1553:67-628 2115:629-650 2137:651-998 2485:999-1296 2783:1297-1363]

KQGNEIHDGEC**KRDRNQSTMAWMKLFYLSLAITAAVLLVNAAAADDEEDARLELCNQY

RQNVTTRPNNRPKRFQRRNNNFEIISVHNVWRDPDTVYWCDFSLDEEDGIKHWKHYDYNV

THWWVEKGCSGTFVVEECNTKDVRNAGSPVPTRKSPMQGTLAAPKPVTNWMNMMSQRRFD

MGTWDREGFNMF*ENAKCIHKEARPLSIKLTLFKMYALRM*RTRRDLKTVRNIRGSFLL*

TIMLIMATWG*V*GSSRRSYIITTICDIRSPISKNI*SISYYFRRSMRI*LTRVPL*RFS

GINTIHQGIPRLYH*G*NGTLPR*VIL*LDRNDYENRKYQNFELHYGIPNPRQNTAVLLI

PRYTVSSLLII*FPYF*IGFRVSVT*P*FRILYH*AETRDVRNEAEDMPVSYYESDRDCF

KPVMV*VYLNTASFPCV*FFTHSTNFKKSYNLRFV

>**comp88085_c0_seq2_2** len=1291 path=[2880:0-86 1366:87-326 1606:327-351 1631:352-761 2041:762-786 2066:787-1103 2383:1104-1119 2399:1120-1290]

YIDRLRVAL*EATPPPRLRHPPGSREAATMRECISIHVGQAGVQIGNACWELYCLEHGIQ

PDGQMPSDKTIGGGDDSFNTFFSETGAGKHVPRAVFVDLEPTVIDEVRTGTYRQLFHPEQ

LITGKEDAANNYARGHYTIGKEIIDLVLDRIRKLADQCTGLQGFLVFHSFGGGTGSGFTS

LLMERLSVDYGKKSKLEFSIYPAPQVSTAVVEPYNSILTTHTTLEHSDCAFMVDNEAIYD

ICRRNLDIERPTYTNLNRLISQIVSSITASLRFDGALNVDLTEFQTNLVPYPRIHFPLAT

YAPVISAEKAYHEQLSVAEITNACFEPANQMVKCDPRHGKYMACCLLYRGDVVPKDVNAA

IATIKTKRSIQFVDWCPTGFKVGINYQPPTVVPGGDLAKVQRAVCMLSNTTAIAEAWARL

DHKFDLMYAK

>**comp88250_c0_seq2_2** len=449 path=[427:0-23 451:24-62 490:63-69 497:70-80 508:81-101 1523:102-128 556:129-266 694:267-290 718:291-294 722:295-425 1622:426-448]

ELPNFTTENQNKMNTLSVWVTVGLVCTLMCHTLAGDSADDKSKGPTDCLPASDPGCRDST

NKAELNGVRYCCPRGAGSISTSSSNINGVQKDSCKCGVRVKMPEMPKFDWHGFDSPFPWK

I**INFPFYPMSNIV*H*DVLFVSLNKHCX

>**comp89145_c0_seq1_3** len=993 path=[980:0-211 1192:212-291 1272:292-319 3404:320-320 1301:321-347 1328:348-375 1982:376-515 1496:516-542 2723:543-554 1535:555-605 2598:606-672 1650:673-752 2858:753-794 1784:795-835 1825:836-850 1840:851-922 3206:923-934 1924:935-957 1947:958-992]

ERTCR*EGY*L*DNGRKMVLQAITALLSLGLCYGMAPSGSTPTPAVYNGAGAQQSLFPGQ

QAHQPRVGLFPQQQQRNPSYLNMGSYGSSLFDPNMFPVAHAPVGQQGGLLGRAVARTSGG

QANAGRGNQNLMRSIMQNTMTGRLMGLDNQEIAHLNSVRTLGVGRANIDRLLKIDQIPSY

NYYLALKNKPAQFAKAQTYLMTLNRLENSATDAQLETMGLRMMMQRNQDPDIARMFKLDA

AQGVYGDRLQRAIMRKNRMSLLG*LN*SVN*MHRYDDDDDHHHSPVCVFRFLFCLYLYIC

LSRSNISAGSIIQGV*RYHVNVGCVINDK*X

>**comp91400_c0_seq9_2** len=5175 path=[1:0-113 14857:114-114 249:115-255 390:256-4786 4921:4787-5062 14100:5063-5174]

REARRRSISFRALRNCHHVILPGRILIRHGACSGCQF*RCI*TGPILS*VRRGTMWRECL

PCRTAGCCVTSSTSSLTQLGWWTMFRRTQQRNQVHSNGYKQHWITCDIMESNIPLQCRTS

*MGTSSPSWTCCGSSSSTTVCTR*IRMPISAVWGLARSTSWSGVRWSWQHSLTQGTHLPS

ICVKATGSPNCCRSSSEMTLQMTRTELLP*RSSLTLSNTSIISAEISSALQTS*MGQWMN

TH**SMLPCSGGGLGETTKP*MHFPNLRSPREA*TWRWSTP*EARAPPPNTRPTTRRIDI

CHQTVNLSGQTTRRRSSQITSLNRCQFPYTSPTVLEKVRLLSLPGNMDLQKSFLTGCHHH

PLCQSIVKEMGLISLQMMMAQFP*TRMERVC*RRQSVRECMNTCPSQRMKRTRIQVL*HT

D*MTCSQKLQDIPQVEAPTKI*NHFQHPSDLP*IQTVGTSLIVELRKCLELLRSKDPRTT

SCSGRRHLTGSLRDPQAHQISFSLPDPKVTLT*RVLVEGHDQVPGQDQDPMNQDKVYVIV

LYTSLRWIEKGEEPQRICFEANLTVSQIQ*RNTSKPGKMVLYRVEGHIHLIINHQNLLPV

LRKVHLPLMLTWHT*VELKSVIFSLTLCFPISNHCCPV*TLRGWIF*INQVNLLMFLHYW

IT*RKLVLQLTTVCRKKVSEVEGSQTRKTRPRLRMSMTQARKEEMNQLSLKDLLKKLNMM

TLLHPADIYRICLFQGQKPSEGGLHCEGHQGDILLQAPD*APQTLSLHTESWNTSQTCMT

MTICLTLIAAIVICLAGKLGRDPKEAVSCLLMEAG*IL*LKPNLLRYCVKR*KN*NTRLK

QWKALVRQDKLVQLPDPLLEGGEEPLLYS*RGQKLTL*VSSVTYMNTMVTWFLSLLEADH

LVPFIRDKESHQIYQGGLPRQEAGMREK*GGTVPTTQEAGH*VLH*KNREI*KIN*EIEY

PPTDLMGQVTDTWRGQCLTVHTASVPVDTQALWTPCIWMKATRHGIVQGHLHHLMVLQQE

IDCLLYEDFREEAWPFMNQGIVLELGTINLLTLMIRQGPLVLATAVL*EVSLRRSGVTS*

AL*RAWNLRDKTDGRISLL*GSFQMKMSLS*NRHLLAVLWKMTSCRPS*GMQKLMCQKSF

SRQMMFLMIAGDICLSHKQRIWNFVAALSMHGTSLRSCRPE*KAWKTACSRLMLSQIR*E

WKYKRQVQRSTKPLVN*DL*RTLNRSIKV*G*IYLKWRVKMILSDGTLKRCSLTRRRLRA

PSRN*EPAWRK*DKRGPSCLTRSMPSMPAIRNPR*LTS*ATTPRREPVMRWREK*EEGQA

VNHRLDLQVQAVPGLQVWVVHQGRT*VILLQEGPGLMTTSLRVRKTWTLHCESIDAQVHG

TMNWSPVL*SSTLH*TCRVRLIVKTQVTALRN*CSLRVCHHHSGRSTHTGKSSITETNRE

RP*IIGCPLFPLLTTQICNSIQPNSVILIMMQRTRTGFQEKDLGKMEVSERIYFKGSIEI

VFMETTTLTI*IDMNLVRIMAQEETW*RQLLNPHPPATSAQAQTLQTGCLRACRRHCDNL

TMIGRSHQTDSMCPIIQFMTCVTGRV*VQVLRKGS*KMGRRNILTICEREAVALSYRDMA

GLIHLQLNCHLATMLP*KQSQVTANQCMEGHPVLGVLQQN*LMSLTEGESQT*KQATLEQ

SPGTNYSCKYYSVCWLLYI*SGN**LDALLAS*ADSR*YAVIVPT

>**comp91512_c0_seq6_5** len=701 path=[2400:0-117 2518:118-222 2623:223-226 2627:227-364 10828:365-385 11160:386-388 11163:389-389 11164:390-391 7579:392-397 433:398-675 711:676-700]

QPPQQEQQQVNQMNQQQVPPPQGGAQPVHGGHGHGHGDTMKFSSDIHNAEHVMEHLENVI

ETKPKDQMTEEELEFHYFKMHDYDNNNKLDGVEIGKALTHYHAEHNDKSAPTKVLSDEEL

AEAIDHGLIPDVNDDGYVEFFEIKHAIKMGKAF*WYIFRL*WCI*SCYL**L*EFTLRP*

VV*YVEYGCYGWVFLCVRSYFMSSCQ*TCILSADTSIVFISFCL*LC*LLQK*X

>**comp92139_c0_seq1_6** len=4232 path=[4311:0-46 4358:47-49 4361:50-618 4930:619-1740 6052:1741-2273 6585:2274-3976 8288:3977-3984 8296:3985-4201 36:4202-4219 54:4220-4220 26:4221-4231]

TSSEQETSSKPETSSSTTTKPVTSPTVTTSTSPRTSRVPPTTPVPNWRTTLSVLTSTTWE

EKLSPFHGCFELTENNKPVNSDDMASAALMQQYKRGATTCPEAYASNMNWPATVAGTFAE

IKCRSGSGTATWRCGSNPTCWRGEPNLAGCASPKFKKILKKVLEASEEEANEEEQVKMTT

ALVEVTKTEEMSVEDVMLTSKVIQSLATQSQDSKPKDEKAVKSIVKNVVKAGSNVVSENK

SSTWEEMAPEDKARSASNLLVAIESTTVAMAAVIDHPTVIEAKDENIDLELHVIDVENMT

EDALTYDDEGSDNVISIPVGTLKSLSKGGLAKAVFMTHYKMADLLEERPSATDEKRTSTK

IASSILSASIGTDSHGIVQLSEPITFTMKLTQEIPGGVTPKCSFWDIRQDKGYGRWSQDG

CRLAGTNNTHTTCKCDHLTNFAILMDVEGVEMHTVHKMLLEFITLVGCIISILCLFASWI

TFTCFGSLQGERNSIHKNLVVCLWVAEILFITGVSRTENKLACTLIAGFLHYFFLSVFMW

MFIEGIHMVIMLVQVFDAAKSRLPYYYMTGYGIPLIIVSISAGFYYQGYGTDQYCWLTTD

RFFIWSFAGPVALILLVNAIILTYAMVMVCRHSEYVLNSKEKNSGGMRTWLQGALSMEVL

LGLTWTFGYFYISKETIALAYIFTILNSLQGLFIFCFHCLLNKKVLKEYKRILHISKKRP

SASTQSASLMRKQSQSCEMSSKSRKDSSC*SQPPLGQGRERQ*SNATTLMSRS*ARVPT*

N*YLYT*ADLCLWLTSFPITFFSLFKNCNVRHSWNLQNQRQHNFIIVCFEGRNNLRSADT

KNSNLFNLYLLFSLIVFSRRVR*Y*FF*ILPNNYTC*CMV*LYLRPFPMILSWCRSSLWI

TC*STNASRMLSFTLIETNR*QV*YVLMH*DITAMYM*LSMHFRIVLFCVRQETG*IDRE

VTERGPQEGHGRRSSRSQTCLEYISNVELLLSWSHVKIL*PLPVIISKP*RLT*RSLQED

TRDLDSVL*IDLSIGVRSTYRGDCIVNLGSVPWSNLIMGVRST*R*DRLWILSLFYESIL

V***DQLSAKIALWISALYYESI*IYK*DQRGPGLWKAFDDNAFLN*QSMLTKYCHAVFH

TKCSL*SLL*NLDNVPHTINGVFNHLVICTGYESVCSRIVSCVYK*QSKSVIYADI*QWY

LPLHLPFNTICISYDF*TWDATNSVIFTSVLTETLVISLLPSRAHI*TNTLRS*MDLRTY

DEQNHMTNSHRSTFHVTVIFRHNLHTVAV*GTQCWMQISIPYYYICSNWTNNLCFHDALL

*HSIL*SSSEKCL*TLTVCSIKCLCKRNDVYDCSFTSDKETM*YSPGAERLTFVSSGKRC

FAAEAKHF*TTISLQVSRAFRSEMHKKSKIX

>**comp92223_c1_seq1_1** len=1422 path=[5715:0-199 5915:200-218 5934:219-467 17974:468-468 6184:469-484 6200:485-1421]

TYYFRCFCADRQSVLR*LLRKDTIWYTQH*QIMHWSVAIVAAMIFMAGLTDSKPVAQKKG

PPPPHDKAVEGGSDDEQGGTNLPYDRYLREVVMALEEDPEFRKKLEESNVSDIKDGSVAK

HIELVGHQVRTKLDEIKRREIYRLQELRRLQMRSMQGVGGVVIPAHLDVQNPHSFEMKDL

ETLIKQTTNDLEKLDDQRKEEFKEYEMEKEYEKRDKLNHLSEEERKKEEARLEEIKKQHA

QQPKMHHPGSKDQFEEVWDKKDHLEDQEFNPKTFFKLHDIEDDGFWGIEEVEAVLQSELD

KVYDGNTPGTDPMERFEEMNRMREHIFTEMDKDKDARISQAEFLQYTGAHGENEEFKKDD

GWKTVDEDQLFTDEEYQKFVEEHHAQPGLINQPPEHVDPSVMQMNQGQVPLGQVPQQGQN

LQYQGQPQGHDFQQQQQQYAQQQQILQHQQQQFAQQQVVQQQLAEQHAQMQQHI

>**comp92321_c0_seq1_3** len=1134 path=[1112:0-66 1179:67-80 3080:81-420 1533:421-433 1546:434-493 1606:494-672 1785:673-685 1798:686-810 2258:811-832 1945:833-1008 2121:1009-1016 2129:1017-1133]

F*VVQVENMAVVDQFTIYIVTAGIVTLILVVVTLINKFSSSTKKPDERDEARQVNRPRAA

EAPPGARRRGPRRRMRLNRDDSDEEMFEGDDDDIADTLNQGDGKIGAKKLKKLQEKAEKR

AAREQELEERAERKKREALLDEQRKKDDEREKLEEAAREEEEKKIREEKERQEHEEYLKM

KEMFSVDEEGQDDAVLDLNSESLLQEFINYIKDMKVVILEDLAAHFKLKTQDVIQRVQDL

QAEGQLTGVVDDRGKFIYITMDELEDVAKYIRQHGRVSISDLAESSNRLINLNPENTETH

KKLVSEVSE*CVTPSVSKWSDWNDVEDVVEDDVVNDM*QNLLCMALVI*EHVT*PCVFGC

MRLSMTCPYVFDLVFNAX

>**comp92337_c0_seq3_1** len=2500 path=[1:0-462 464:463-463 465:464-1965 1967:1966-2413 2416:2414-2499]

TTRVSRESSRSLEY*WTSCSDYLRKRLFPHSPREL*SHASTDYNM*KT**AHLPRSL*KS

WPPPLSALIALSSVSRQHSLSLHYSQAPGSNTIDR*FSINIL*CEIDGSQQEMAGRRRA*

NRHKA*LWADSVFLPDMRSQETV*GLPTKMFLQNWTGSRLIQDKQMEEKVLN*EVSIITN

I*RLTTGEVSSASNMGLRRRYRRPTDSVWYIAASCVIFFLVGNLLMFQSYHTFVDDVQHR

GNVHSPHVVEVSPHSSLVRDLTDDLLSRSYGKTVNNQYFVFESRLRKLMEDTRENVYAEG

LSSSHTPQIQGTPVTGDSMLNCNDIENITEREFIASGWTKAVFKGKYRGRSMAVKTVYIG

GQDINTCVQQGVSMEECYHRAAQKIVKEIVVLQALPHDNVIKVIGFCVPKQRYDGDSNTA

VVMVTELGETIDLIKLLQLSWEDRLRISYDMTRLLRFMSTTPFGSMAMNDFRRQQFVLVN

GQLKLSDVDDVGFDEPKCQEDRDCYLHFSSSNFTKRLPCLQGRCNGFNEMRNIFNAGRHF

TTFLLPHGAPPLLKPLIDRVVDAYENLSLNSRQLVDNMEKIVSLYKSGRHLNRTAASAPR

PAFIHHEDSDLPGQFDYRCRFSMSGGGCTLSVFDRQEAEDLCNLDPDCKGFVFTNQKTWT

GRTIVHLKNGVASPTHNSNTELYLRPS*NAQDRIKNTGWRFSVKYHCCDMDRTISVPTFH

*WCSFEKSTPLSIHHRANPPEGIGFDRSEARASGSAEVGMRRHATHDLHVLMTHASGSIK

RL*SRLETFVEGVWRVVAIEESREGLIFFRK**GCS*MDASTSEIALTETCFLX

>**comp92949_c2_seq16_6** len=2805 path=[10669:0-168 25616:169-326 10996:327-505 11175:506-523 11193:524-749 11419:750-1339 12009:1340-1367 3329:1368-1370 3332:1371-1405 26308:1406-1409 3371:1410-1419 3381:1420-1443 3405:1444-1676 3638:1677-1724 3686:1725-2454 4416:2455-2473 4435:2474-2613 4589:2614-2804]

GSHSSSFNFRGRT*EGSCELRRVSWTQDRVVFGRT*YFHR*G*DADNGSVVIKEVR*PNS

ADGVPLTTDDLML*ANEDASNSSQILSLTDKRIKTDHSKSDAISCVCGEFVIQD*GGDQF

YFKMALRLSGLAQVQLWIGITIGLVVAFLVTSISRVSPITSFGFSSEQLNPRHIGQTVNT

MIAGTDGVPASGPKGENLHEEFEKHVEKHKAGNVKFEDKHVHHDDDAIAKSLFEKERVLC

WIMTSPKNLESKARVVRDTWAKRCNKVIFISSTTDPKFPTVGLKVAEGREHLTAKTIQAF

RYIYEKHIDDADWFMKADDDTYVILENLRYFLTSQKKTDPVYFGHHFKTIVKQGYYSGGG

GYVLSKEALKRYGEKGHDPKICRQDGGAEDAEFGKCMENLGVKTVNSTDALGRSRFHCFD

PETHLFGGYPDWYYQYDANGAQKGTESISDYAISFHYVPPQKMYALEFYIYHLRPYGIVS

GTQDLNKKHEKPGTQAH*TCVMVPGSQERHA*WRNIITVICEIC*NQ*RFSL*LSLLVPE

SGAFL*LLTSTR*C*CDGRLAAHSMCEVVSPLYQTNETSQMSGTKCLFVQHHVQRDLDTG

GRLVNWSVLDRLTQALVY*NC*LGQIPRMVTSNLISTMSRETRNSSSLVMSTGTLKSRET

CVSLKL*PKNNHFNNDQILNHGVQQGYSLCGRATSSA*PSNVMYVASVQEKKPMYVFSIL

SHRMQTLEALSESWHSK*LHTGLHPCMC*PEVCCLDKQS*LYMVLPYILC*DVCNFYFRA

TQRLRRPSICSF*DAGTVT*G*QHVI*INNVKNIKCICPVMGFTI*WRKESLKSELRSRL

ICEHSHKSC**ANTVA*PEFLRTLAMKLDVGVNQRHILFIMLILCVIK*ILDGILSSPLT

FYDVIITSGLEYFN*FSATL*IHCYFRRSE*V*FX

>**comp93346_c0_seq1_3** len=2246 path=[2427:0-86 4933:87-120 2548:121-145 5716:146-146 2777:147-1197 3828:1198-1347 3978:1348-1348 3979:1349-1349 3980:1350-1481 5820:1482-1753 5873:1754-2245]

*N*PRPTLERVHNRQ*QVARGERKLVNLNNNIPWGYDLTGTN*PQFKPGRQINIMGRMTT

NLTLLLVLLVTSQALPQRLKRNVDSDLREVLLELMELEEDSSKVGSEKREQDQQQEEEDQ

LRGDEYSAELWLEDVNKKLRKWNNRMSLAEWVFSTNITDNNREQFANSETKYSEWFPSIL

EQAREWRKSDDLDPDTNRQLRLLTKYAEPKDPEDVRKQKDLEGRMSEIYSTARVCRSRED

CLQIEPGLIQVMATSTDPAELLWAWDGWRDATGPPIKPLYLQYVKLLNQGAMENDYKDYG

DYWRDAQFDETPNLEQLCDRLWSDVKPLYVQLQAYVRRKLTEFYGEDIVGTNGAIPAHLL

GNMWAQNWQMISNIVLPTSTDPSNDVAKAAEVKLKERYNVTGLFQLSEKFYESIGFFPMT

DIFWEKSMFVKPEDRQVTCHASASDLFARDDFRIKMCSEVNMDYLYTIHHEMGHIEYFMA

YSNKSTVYRTGANSAFHEAVGDTMALSVGTREHLNSIGLIDTATTTKEGDIADLLATALF

KVAFLPYGYLIDKWRWRVFSGDIPEQHINKEYWKLRLEYQGIVSPTPRSEEDFDPAAKYH

VPSNSPYMCYFISFIVQFQFYEAMCKAKGHEGPLHTCDFYRSKEAGEKLMNMLSVGASQP

WPDALQQLTGSRAVSADALLEYFRPLRAWLEEQNQGENLGWAGAQINWEE*NKDVFFYVD

CIWLTCVT*LALVCKHACGRRLEYWFAN

>**comp93938_c0_seq1_4** len=1361 path=[1:0-76 78:77-81 83:82-275 277:276-373 375:374-381 383:382-1309 4482:1310-1360]

ECSECIHP*SLSFLCLQENGFFVDCGAFDGETKSVTLMFEKLRQWRGLLVEPNRDQYFKI

VKKNRNAHSINACVRTDIENYGRPEKSEERYGFFEGRQRNDVKAVPCFWLTTLLLATGQK

TVDLLSIDLNGKEMPLLQTIDFDRINIKSISLEIPISYEGRSILPILDYLKLKGFSAMHQ

FTDHVHKTKDLVLKKTSP*NRPRSGNAVLVIFQTRIGIVDFSANGHSKHIYSADQFTKEY

DRECGKYRVQTLFGHLSMET*RKEW**RHSAESWRSS*CDIVVQGPSHMKREPGHERNN*

NM*SLCGINDYHHEP*SYDLLQLKPLPTVQGHFWVWNNMLDGSQHFTKIVFILRLFPMTI

QGS*SSQSLDVVMMCYPGVHVHIYTSHITHCMQFAV*GQNIVSAFRSQTYVTA*GVCFIY

PYIPAERYKHLINQVIELFTSMHQ*L*SVCGEN

>**comp93951_c0_seq1_3** len=2511 path=[2491:0-1214 3706:1215-1216 3708:1217-1250 3742:1251-1261 3753:1262-1597 4091:1598-1691 4185:1692-1748 6236:1749-1764 4258:1765-2365 4859:2366-2405 4899:2406-2510]

GSCSITQVDAFCTPRSVASTSRFVNCF*TKSFITYSIMRGLVKAKKYDWKDSNMALFGSD

TEKEVKKESAEAEPAWQGVGQEPSLKIWRIEQFEVKDWPEEDYGEFFNGDSYIILNTYKE

EETEELKFDVHFWIGKNSSADEYGTVAYKTVELDTFLDDAAVQHREVQGHESSLFKSYFP

RGIMTMEGGAASGFRHVEPETYEPRLLHFNGKGRNISVREVPLSKNSLKSSDVYILDLGK

RFIQYNGSGCNIMEKSKAMEFMGKLKNDRGSSDDTVIEEGDGCPEFFDALTEEDVDGDSD

DEDEDADGEKKLLKLNAEGVFEDVKTGDISKDDFASEDVFLLDTPSEVFVWIGSGASSAE

KRNGLPRAHVYMSTRPKLAAPVTVIKEGGRSTAFQVAISA*TTTMAILDTRSPRQAWAQI

HQEEDVRSMIHLHPLSHTYKLMALKF*NDHNGNIC*RTVSV*LVIFDSGNTAIKARSNSF

*RNDLYLLTLQK*PL*RSMS*LRDSGVERFLYQ*PNVPCSCYHKHIG**LCMTLFKQCC*

FSKVLPGNLSMT*SYLIYLFLTLLVQ*MINVWYSSSSAKCLTLLKEIEC*S*IYFSHVNK

YHVMNI*TVWKKKIKI*MKAISIIQNKNCRTVIL*KLKPCYMCLKT*TYVIKCM*SFLVT

CICKCMS*CYVIW*ILMLPKSFHTILKQRTNYSICLFYLCSWVYHQRSSGTNPMCYIQTN

LILLTQVSKMSVLLLGLKMVVLF*DNFDICLFCKIYFHHTVLQ*ID*IN*CHFYP*PSAL

CKHCC*MAVACSLPLLSSKHIWLW*LFSFIALYVIVILSSLFSS*HI*IQIKILCCX

>**comp94680_c0_seq1_2** len=2492 path=[2470:0-308 2779:309-309 2780:310-354 6053:355-416 2887:417-471 2942:472-1148 3619:1149-2491]

LEAATISSCGGG*RCWFFNQDSFYRGVWAIHST*KNLSKPLR*SPAAMMRSLSFLVVSVV

LVCALVSSAPVEQAAGEKIVEVAKRDQEAILFGNQQNDPRLKKKKSDPAVSVVPKDSSLD

EQGSVIAVPAKQEDFIPEDQHVAGGVQEQMAVEADDKQGQTEPVVPAIKQQLPEGPVGDG

VSEADTDSVEEELKDILADNSKEGPGEIVGAPVEGPSDDVPAEEEEVSEASEEGDDVDQT

DDSEGADVAQAGSDVSTPLLDLYYYMQENDKARNEPQQQDYYPYDLSYYPYRRRRSSRRS

RAITEGDMIHNEVKRSKRIKRDLLDDIDAYSYGDEEGYPQQLTEEDLYELYRPEPRYEDE

VASLLDMYQQAPKVPVYEPYEPYPAEDSYPESFEELEQEPEYVPSKRQMMSNLPGIRKRY

FYPFSREPETHWGAFIPQQKRDYEQAYKRLLALASALSEDSPYYQDYRKK*ILNLMGRSK

LGRRSNFFII*RNTFRDVSLISPSRMRCEHRM*CILSRVVQRRAWLWIVCMEDR*QIVKY

IY*KGLSTNYLYT*IDNNQFIFLWMCNVVDM**YYLVLDCA*CVVVFHPNYLVSHSPFFP

LISMLNARKET*P*TKLVYTFL*VKTKVIIC*RVNNFGFST*YRINVCACW*EDKGLHGF

QEVSIYGSCIISLGTFRSSRLRKKPRQHNLTQLLCSELFLKLYLL*RISL*MHLLSQYYS

YYSNCSLYMYTHWSDKTQTLSQQVNSSFIGSVESLFLQMNHLYHTKVTTTSNIHMKITYC

SSTLTNHIQRDCQKISN*MCNIPPLPWLPKVVMLKGCVSLFADVNKKFN*X

>**comp94706_c0_seq1_3** len=1255 path=[529:0-183 713:184-211 741:212-327 4367:328-343 873:344-367 897:368-432 962:433-527 1057:528-532 1062:533-619 4462:620-702 1232:703-706 1236:707-794 1324:795-878 1408:879-892 1422:893-954 1484:955-988 2730:989-1005 281:1006-1029 305:1030-1034 310:1035-1051 327:1052-1087 363:1088-1093 369:1094-1127 403:1128-1186 2674:1187-1199 475:1200-1231 4685:1232-1254]

TVTILT*IGLVFVNMQVLKTLCILVSLAAVSLAWNRPAVNGRSLGGSTSSRFASLLKSRK

SSLFGARVRDGSSIFSHLSSKTKQPICYFDTNGVPQYEWPTKEKLCFDKSYKYPVDRVFS

ALEKDPDAMEYMLLVQGCYLHNKMNATGHYPAYRFKDLCSEDTELLERIWWTNTQNGRRE

VCYVIMPEAQGVEVAQCGGHCVMDTTEVKHHYCVPDGMVERDIYVFCPGEIKQCRRTRVT

IPTGCSCKKYECLKYQFV*TVVYTLSSKS*LYACVNVA*LHQLENAKTRHLCVRTEIFTT

CLRIGKCCEHFEQDGQIFLKVL*SFIDHRQIYIF*KISTLQEH*MIVVSTSLLNTTTYFP

IE*HHLYIYHARTCK*FVNIVHNMYI*LWNSRDIFYHFLMPT*RNTKSTVFSSRCFLX

>**comp95104_c2_seq6_2** len=1130 path=[11165:0-21 23254:22-36 23269:37-45 11210:46-221 11386:222-338 23536:339-341 23539:342-1129]

RLTGLGGHVIVFQLRYQLYMVLFDQKHARKRTRHLDNRTSF*KKCNPKPINKPIFVSVYG

CK*YC*TIKSRGTTSITIIKRTY*IH*IWHFSI*LSCVGINWDADVDVGEYICSVKWNDH

IHKGVVHFHKLGIGAEDTDTYKPARGMYVKEDQTWKMRTLPSIMKMLGHEGKVIDVLKVD

VEGFEWFMMQQMLDTGIIHRVRQFVVEWHLVHDFPTKKSYMDLLRIYYKMKDAGFRTFSV

DFRFRGFSVRRWRIQSDVMYVNTKFIPNSN*QCPPVHVSECLLNVVHELM*LI*LIGGCL

RTMLLLRVCF*KVRCSHPLPKTGAVGSASFHPYTHT*VHT*QSTI*LL*NEFWDVSLLH*

AHSFILYFKNFTHSAIX

>**comp96713_c3_seq4_2** len=1246 path=[18995:0-132 19128:133-225 1019:226-251 25:252-295 69:296-306 80:307-875 22586:876-878 652:879-1245]

RLAGATLRRVDITVDNGTVQTSPRLSASFHDMNTLVILLLGLVAYSAAEVAEEDGVLVLT

KENFEEVTTENEFVLVEFYAPWCGHCKALAPEYATAAKTLASEGSGIKLGKVDATIESDL

AEKYEVRGYPTIKFMRKGKATEYAGGRTAADIVNWLKKKTGPPATPLKSADDSKAFIEAA

DVAVVGFFKDEESDAAKAFLEAAGGIDDIPFGITSDDAVFKDNKVDKDSIILFKKFDEGK

NVFDGDFKADEIATFISGNRLPLVVEFTQESAQKIFGGEVKNHILLFMDKGDDFDEKVEI

FKSVAKDFKGKVLFITIDTTTEDNARILEFFGLKKEETPAIRLISLKEDMTKFRPTSDEI

TEESVRSFVQSFVDGKLKPHLMSEEVPSDWDAKPVKVLVGKNFAEVARNQEKDVF

>**comp97143_c2_seq1_6** len=1429 path=[1:0-542 544:543-545 7421:546-566 2967:567-640 7511:641-739 7540:740-740 7541:741-749 751:750-895 897:896-919 921:920-1069 1071:1070-1082 1084:1083-1176 1178:1177-1207 7740:1208-1237 1239:1238-1240 1242:1241-1428]

TMFGLNSVTALVFLGVCSGQAPSTETGGSNDLLVFTQTAMVHIDVTTGQSTSFPKPSIFS

ISTVDHDRVDNKLFVVAVSSVIFSCNMDGSNITYLRDWTSNPSPFTDSLAVASDERLVFF

GGSGRQVVRMSVTGEHETVLVNRPNTVQRMAVDHTTRMVFWCERGMDNNVYKMGFDGGPV

AIHKDLRFFGPQPRAMDFVNNRMFTVEYDGIYAIDLAHLDYTVINSRGTRRDRDYDIVYD

PRRQRVFYSFGNIQRVRMMTPTGEQDATVGPDLGSGKKMLLVL*AISIHTLSTDTVISTY

NGIN*IY*ITMQRRVFIFVRKIE*MLLKQK*NYVILSGTSVN*SSGVCTQDSKPPYILDR

GLTSGRANASTIRLANCP*TSLRPLYFAHILHSALWS*VDLVNLAASMDFC*PIYC*NIS

R*DAFPISRTLQEAWHKLISQKKRYQTFFQYMF*LSIPYCTKT*AFPVFQTKRIRS

>**comp97289_c0_seq1_2** len=3277 path=[3290:0-686 3977:687-838 4129:839-2183 5474:2184-2204 5495:2205-2458 5749:2459-3220 6522:3221-3276]

ATKTNRLLRLCGLLVLLLSQSKSFLDFEELKSTHYGIDIVNVPVKMGQEVPGSVVQLSSK

FGQAYQCSFPNQVEQEKQKEEEEKLALETGILQLLKPFKTQGCLFKTKDWWSYEFCYGKF

IRQMHIEEGKVKGEVIYLGHYESDFDWDNETLKDQRLKTKSFQSKYHSQSYTNGSKCDLT

EAGRRADVRFFCEEGTSDHIARIDEPETCVYIVTIYTSHLCNHPYLKPPTPKKPVPITCN

PVVSEEQYLEYLADTEAEKERKQLAEKAREAAAVAAAEAAAAEAEAATTVADDVDAKTEP

ESTVTDISDSSIETGIEAPQSDQAEPIDEEPTDMDSIFGPSVESLMKKTLSNEMKSLAGQ

STEKPIDVKLSFKVVRDPKDIDSFIKDNFGAEGDEAETEDSGSSRATAPGEPDSDVVLEP

DDGVQEDSKLEAHQVEDGTPTDVVGDDVNVDADEELMEEFDKELGNLKTAYRNKRNKFAN

MKNKVRDSMKLQFDEIIDEAEEMYGEEIDKKLAFKQLAGTLDKLITKLEHTEKEIDNVDR

ELEQFNKKTLTSKSEGNPSLQEPKGPGSQTVTTSKDSPGTKGDVADDDRVKVRVTRVKTG

DTVKDGVDVPEEQKQQLEQTVKDELIKAGLDAGGGKIQVKIITTGYYDNEDDSIHILSQE

DSSNFQNMIVAILGGTNEASKEASRQQQLEENYNYVWGQSKNKKSKSNIIDKGQ*YYGER

*RNSIFYMYC*FSWCSDVWCQWLWNTCLILHKMSILYSHVFPP*SDI*H*ISVTMGKCLG

HKIIYNQR*IFCVCDI*DVCNQ*QHFSTNLLFI*TNNIQRRILLQPVLKS*L*RIVRIVW

S*QNRFEFTASYNCNHYSCQKLQLPV*ETLLLLLLMTFI*TDGMRCKKLESGAKMKYQLK

ICFSTVLENGGKKIRDMLKLILIKLIGFKEFRSSHIVCNYQK**NGNI*VGLCLIIESSW

VMF*MHLQLNDMLSK*MLWSLLWTF*QWSLRDDCVHYDAVYIY*SVCEGNQNTN*VRQPV

ELKPK*PCLYLKPY*Y**R*ELNLKSFFH*ELLPYC*CGFKQSFTHFNSIILGRSGLEVI

FSHLLLVLFLVS

>**comp97298_c0_seq1_5** len=1379 path=[1862:0-140 2003:141-276 2139:277-300 4970:301-352 6696:353-353 5023:354-418 8496:419-525 5195:526-580 5250:581-738 5408:739-749 5419:750-766 5436:767-972 5642:973-1378]

SPLRGCSHVPICLCHALTSLWVFTCAPVLLVFGLVYALNVPSYICAFMALCGDRRVRLTA

VMDVSEDCGSRVNETTRGLKQPDINLAFILLQPRFQFLASAGIRDELEQAEGRV*YRGQT

QTTMANQKAVDNILECVREAYDKMDVNDDNTVTFGEFKKFCDKIDGMEEIAQTFATYDTS

DDQRLSLQELNDFVTGKVPKDQIEVCHVRKLFKSRDTSGDGKLDRAEFRALLVDCGYSDT

VIENTIEAVAGEDGMVTLQEFLDKWEHDE*LCPLFVTLARSQSSYSLDGTY*ELLPLDCF

ASNALPRISQSLTRDRWYDLQSGPTSVPKIAPLSIKLLFLYICLSV*TPLCNSYTKFAHW

TRQSGDWDKGVASCQWQ*HICTGGSAPSIMTPMQ*TLVLFSLILDPDPHLTLSL*SGHHS

RRHVGAPRICESRRELRVRVSPQCVDI*DTDAMSYNPIKX

>**comp97330_c0_seq1_5** len=1499 path=[5244:0-5 736:6-29 760:30-93 824:94-113 844:114-179 910:180-219 950:220-446 1177:447-470 1201:471-564 1295:565-588 1319:589-602 1333:603-626 1357:627-688 1419:689-725 1456:726-928 1659:929-945 1676:946-1117 1848:1118-1141 1872:1142-1351 2082:1352-1425 2156:1426-1449 2180:1450-1498]

SGQLTLSINTKVGR*HHYLPSVNQC*SLVSLSLHYHTMPTLGYWAIRGLAQPIRLLLSYV

DQDFEDVQYEQGDAPDYSRESWTSVKNTLGLPIPNLPYYVDGDIKLTQSNAILRHIARTH

NCLGETEKEKAEGDMMLDQAMDFRNGIVGLCYSSDYENKKAAYFQALPAKLGVFENFLVN

RAFFAGNKVTVCDFPMYELLDQTRIMQPGSLDKFPKLLDFLTRFEAIPKIKAYLSSDKCI

KRPINNKSASFK*TTASCNVLLLPWKPC*TCISTAT*GRHSHRQFLS*PSHEYQNTFKIL

GEVNGKQQYFWKIFSLWLHYSFSEDIVFRPKTSKGTLLLLC*SALSF*EFMYTTYIQKNI

FPVFPLLMRDMSFRSTEDILQKIS*INLGSKVVCHGGCFVLIWQRK*WVPCS*LCVLCDT

VEVKHCRQWHASAVLTFHGFWLRLHMEYIVFIVIQVCLRLYPGVTRGT**SASLCFCFSL

DHISKTDRYSFRLATCV*GX

>**comp97769_c0_seq11_2** len=1651 path=[10057:0-299 13974:300-300 13975:301-302 4695:303-526 4919:527-815 5208:816-837 5230:838-1612 6005:1613-1626 793:1627-1650]

ENSRCVSVCARVCVSVRTCLCVSV*LSLCLCVNAHMIGYYPRPPHCQHHMYLFTATTRPI

RL*CPGTRCYLPDNNVITRIAVIQTTKDDGLGLTLWSYSSRMIRLLSSHLFVALATLMGV

LCCYLVVVEDIFLLGGRPLYKQSTSFKPGGDSFANRSLTHYILPEQPTAPDCLETNICKD

LGNKTDVYFYKRTNFDELVEEPFDVNASISHAVPVVPRIVHFCLFSNGNVTFRFHYMIAV

LAASRIAKPEKIYFWHDNLPVGEYWEETKQKVKNLYLVHRDRPTKIRGNDVKVIEHVADI

VRLEALLEYGGMYFDTDSIVVNPIDPLLVHDVTMGHAVPYVLANGIMFSKPWSDFQKIWH

HEYKTFKDKNWGSHSVIKPYQLSKKYPNLIHIEPSSLCRPNWKELAYLFGGRNWNWQENN

FVVHLYMRFGPKGNRERNRENIKTWNTTVGSIMRYIYYGNADLIEDEDKMN*QKQMSESG

GCWWWWCWSWWYWCLWWYWC*W*FKLLMHITRCGNCILCLIVIRRNYYRHYSE*VSERI*

FYATLSSIPA

>**comp98044_c0_seq5_2** len=630 path=[1516:0-58 1575:59-108 1625:109-129 1646:130-263 1780:264-270 1787:271-569 6139:570-574 6144:575-586 5788:587-629]

EALI*RRRLTSTDTHHSTWPQRYVCSLAMIMKMLKNAGNLHVAFLLCLINPCLSTDVYKY

FTTQGRDRLVEALFDGDRGYADCAVFGDRQVVGTILKEVSAEKIEEISKSDLDLYIEKCH

EFHLTLDDSPLRRKRFAAIYPGTKWCGVGNISSHSTELGVHNYTDACCRQHDYCPDYILP

FASKYGLYNFALYTRYSHSIGGVRVSE*VX

>**comp98461_c0_seq1_6** len=2919 path=[20:0-400 421:401-408 429:409-733 754:734-962 983:963-1115 7550:1116-1123 1144:1124-1355 1376:1356-1360 1381:1361-2051 2072:2052-2918]

CWTHLSVLWILSNIGLCTVHWMTAPSTNISMLINPIDLRPKDH*TIKMSSKLMFRCFLLA

FLFGFLGMLILGYKIFSSNDDHPVYYDNFLVDVNEDHRPLDEELDEESIVSSIKEPIILW

WTPFTGEKGIYKTCGNVKCFFTVRRAYRNHPMTSVFVFYGTDFKVTDLPLPRSPKHEWAL

LHEESPKNNYLFSHPEIMTLFNYTSTFKRESSYPITSQYLVGIDWLESTQYLIPTGQKHK

HQKELAPLMYAHSDCDVPSDRDHYVQMLQKYIKVDSYGTCLHNKDLPDHLKDPIQGMDHK

DFYKLIAKYKFSLAMENGICDDYITEKVWRPLMVGSLPVVMGSPKIKELLPSNHSAIIVD

DFSSVKELADYLKYLNENDAEYEKYFKWKETGITNPYLKKLVEKREWGVDSEEHWSPATI

NFIDGFECHICNAVHQNLDKQQRGQAPFPHRVTNDHYGCPAPLKFDDSGKRTHNNETNRI

WQWDWFYGGQLAKAVRSFIDQNMTFTSVDLKNKLKIVL*NIEFHLGVFYFLRLLV*LYMV

YKHFCYKVQ*LVFMITVHCVTMCQCQMQLFH*QMVTEKWKGGQSLLSGV*LLRYLCLIVL

IIFSCHGIMGCELLNFKIMYNIFLSCGLDVYKVILYWRESQGDYRYNRKSTVNYL*SLFC

FFTECCMRILRVMPTSFITLTQKSQIV*PFVDNILLLIMILVSRS*FPEYYLYQIISLAA

VSGQKCLLAQEVLDHSNIVKH*WWLDFKIYLTFVS**DV*QLCLWQGENNKCFLSIRKPT

MQGYIKTSS*QIYFMSCLKVFCIGFLKSHTQKYKLSQKCIRKIIKICPACLQNTP*LFLY

MFIYSTPASRVSSVSVVPKMIAARPHLHLAQFFLSTTSV*LLTDFLLTVI*KTW*P*HQK

GKRNQTIFSLM*NGHDNIECYSYTRKKTLGDII*LAVHLHGKCGTVYKCLFTRHDN*SES

VSLVVTAFSNIPG

>**comp98892_c0_seq1_1** len=2214 path=[5901:0-17 23:18-1172 1178:1173-1191 1197:1192-1273 1279:1274-1292 1298:1293-1367 4608:1368-1384 1390:1385-1444 6319:1445-1445 6327:1446-1471 1489:1472-1533 6391:1534-1535 4503:1536-1543 4511:1544-1577 1595:1578-1604 1622:1605-1625 1643:1626-1662 1680:1663-1738 1756:1739-1781 1799:1782-2010 2028:2011-2035 2053:2036-2187 2205:2188-2213]

DFRKEGLTDV*LKLKPLKMRVRLKTKAITVYLCTVALTTACVLLIIHFNGRDMETKSQSF

DHMAVDRVMLAHEQRGLWVPEDKPALVNVMEQEPGPQRQEDNQKKAKISHLPEDLSPTEW

LTSLSRAMVANLSYADLSELYHSYVMTVQYECHDVVRLGRVTDGGWEICADEMFVPKSKC

LVYSFGVGNDFSFDDAAARKYQCEVHSFDPSMKQGDHERESKVHFHQKGLADFNGETNSG

WKVGTLESIKHELGHQERPLDILKLDIEEWEWQVLPEILSTQSLSDVRQFLIELHGCETC

SVFNPQLIDKEPTKERYIKALNIFKSLYHLGFRIFWSHKNSACKYVSRFGLKERSACHEL

HMVRVS*SAWPTEITVQLVKGEII*KQSCVPFVIGSVQFIGCVIETYFL*EISYL**SVN

LIRTTLLMNKRYFMYCTTIMFAVYGIYG*YLCLL*A**V*LSYHVH*SVFHPVQRIQHN*

CYWIDLSFPSNGTVQYYQEIGPPCLKCC*NYLVSKSPCYKTCTEVIRVTSLME*NLPATS

T*RSPF*LVC*ATYSTR*PDHID*KHSKLE*QSLNIQWIISVSYFHKYP*ASLCYCFVF*

SKLLS*ARKTCTFTR**QGRETMMSLP*QGYGVKHGSSVSLYSCHVAGVLLGAAFIIVHN

ASYIVDNQ**SIRVLNETYALILIFGVSDIYSCSMVSKVSYITPLYHFRLSLHVG*SEIT

LLKGIVKRILIYTGQNKL

>**comp101644_c1_seq1_1** len=1326 path=[6320:0-664 6985:665-668 6989:669-1293 7614:1294-1325]

WTK*K*LPVCCYLLTTLLPSQDRICRLL*TNTSA*LFVNGGGSVTMTLWLTLLLAVTVGA

VGNESSPGTYIITFAKKIAPGWTVPIRVQILNATQPVSIQIALVNRTADTIIQSTTRSFA

QGQPQTVDFQIPKDLNRPWRWNELQFNFTGTGGLKFRSSETCEFNEKSKSIFIQTDKAMY

KPGQQVKFRVVAVYPDLKVVREPMDIVIYDSSKNRIKQWRGATDPSGVVSRVLAMSSDPV

PGDWKIEVTLDRYKETKMFTIAEYVLPKFEVEIILPSYITLNMTSVSGSVTAKYTHGKGV

DGTANIHIRFSYYYGNFGSKKNPFIRKRVQVVDGKSDFDFSLDELRVLADEGDTYAMKPL

LAADINLQYRSVIVEANVTETLTEKTLNAKLIRKTIYENSFKLNFIDVCPDVFKPGLTYL

CGLQVSQRDDRLVSPQDLEEVN

>**comp170175_c0_seq1_6** len=323 path=[1:0-322]

LTHCNLVPGDYIIGHCIGREKCIPCEASCMGLNDGPNIYPGKALTSEYLDCDKERTRNMK

VCLGGKVFDPMTEDCGFLVCQV*EMPTVADPSPGILGWNAIVRAGSYR

>**comp292963_c0_seq1_2** len=281 path=[259:0-280]

LLLPLAGVIFLALHAAQGSEPRRVCYYTNWSQYRPGKGKFTPENVDPNLCTHYVYAFAIL

LGNKVKAFEWNDLSTQWSKGMFERFIALKTVNPX

>**comp238950_c0_seq1_2** len=684 path=[1:0-683]

FEQWTPDGTWVRHRCPLFTEYSKGNCGCAFLAPQNYNKACRPEMYLPFDDNTDDMSGNSV

HVANEGVTVSHGSAVFDGQGRLVINRFSNAWFGSTLVVTLRFKVAASTSRHALVCNGDCR

VGPSIYIGSNSDSTTEFFAKTYNSPAVNFTLPQSASGWQDVEYILQDGALQGRAAGVWTS

QNTTGALETRQRALIVGGCGGADRFQGSIDELAIYFCNPGTP*SWNAV

>**comp16849_c0_seq2_2** len=1627 path=[1605:0-1454 3060:1455-1626]

DAIIGDLNDDPTILEFLRNSTSVLARRKRKALVDLRWPGTVIPYQITSDFNLFPDSRQAI

LDGIAYWENETCLDFQDVGAAAGSKMVFRRLVGCWSFLGMKPYPQTISIGAGCNQRGIVA

REIGHALGFVPEHQRPDRDLYLNINWSNVRPGKLHKFLRTTWRRIVTLGVPYDYSSLLHY

GAFMFSNNGQKTISGREPDVDRTFGFSKALSFYDVKLANLLYCSGSCPGGLETSMCMKDG

YRNPRNCSYCKCTNGLTGRYCMSVEPGRNTNCSSTSLVATPAWQTLTSPGYNTTGYTAFS

ACSWLITAPNNTAITIRFSGSFDFPCQSPCMDFVEVRFTDLARTGPRYCCSARPLQYFRS

HGNEALILFKANSSLSRNGFALEYRTEPCGGCSGNDSGQPCLETQRFNCPGVFTGNCNFW

GFTRCTNRRRNTCYRIAQSCCQGYRLINNTCYRVLSDWSAWSTCSTTCGGCGTRTRTRTC

IRPPCSEPLSETERCNTQVCSGVPVSSTCSFGFRCGWWFVKRQCTRYSICYRLVRCCSGR

IE

>**comp103351_c1_seq6_2** len=2894 path=[10370:0-316 2498:317-382 17439:383-385 17442:386-391 2573:392-462 2644:463-466 2648:467-1046 3228:1047-1058 17673:1059-1073 3255:1074-1088 3270:1089-1701 3883:1702-1729 3911:1730-1803 3985:1804-1817 3999:1818-2176 4358:2177-2190 18090:2191-2525 4707:2526-2528 4710:2529-2636 4818:2637-2644 4826:2645-2660 4842:2661-2661 4843:2662-2662 4844:2663-2663 4845:2664-2674 4856:2675-2701 4883:2702-2713 4895:2714-2773 18345:2774-2833 18346:2834-2893]

LLSKL*LYIQRLGNSLQAIMNADFIIIINNMDHTV*VIILPISIDNDYQYQALLITTDCH

DDSININK*TVGDKL**EVSVLRLDISSFKLMHLLLM*VMHFEFISDTTTAGTTITATTS

DPTTVATLPSTATTTAATIVTSTTPTPTTRTTSAFSDATTTTLASTTTPTTTPTTTPTTT

PTTTSTTAPASCKMTRSSGTTGVSGYYKFSLRNVDSCSSICLYIRTCRATTYRLGICTLY

PGTSTAASGDIVDFVRHACNNGTFCCMSSSITQRASGSPLLSISTTASLDGCEAICLAVA

DCQGTYYSDDSCFFYTTTSTTFGGTIVFSRKTCPVKTNPGNLEFDGTCKDTNNSASSRII

HGIIIAFLIIHISGTLF*CSFFSKNSCD*LTKR**L*YSGGRKNSGQCDKRNKN**VNV*

RRTLMCVVQKSLCPRYFLALTVE*PRAWSTSGLHQINYIYKYNIESSESFSPVITYLQGE

NPYSS*LYTSFKYLLYMIHVQCSLFVLFHFSQVMDGLR*HYSLYRVAAIRNTKIHCVLIV

Y*LTQ*TCYIV*EIHRS*AVTS*AKKQESINYLSH*VN*NC*N*ISIYKIVA*N*CIYKN

SNEETASCFH*QKYPVALIMDLM*ESLPRNLYV*TYMSGSCNDVYVCASTCTAPEDTQAC

PGLRHCRIVVSIAVHI*ILLL*LLQTAPKLLKVHISYVYFLAFLS*LSLCCLQWCTLGAK

IS*HSNGSIWICRRLITKKYILNQHVNCEL*TGYLSHIFMDQ*YNV*DDQ*ILYNF*RSS

IACLRKGSSHSNTCTWRYFPWHVCGEAVLGIFR*EHTNKDIGISHDATVIRRSIQLVAYT

AVFDERLLHLE*TTYMLITI*LI*RTDILSPATCSQYLYNDVWL*RTKLVAI*VRYNRRL

L*VFHFELTTKANGRKADLPHIVVNFELTTKANGRKADLPHIVVNFELTTKANGRKADLP

HIVVX

>**comp45250_c0_seq1_3** len=312 path=[1:0-311]

ITPPQPQPQQPQQPQGLSANMPNLPQNQDPKIAPHLSEALQRMPEPQRTQFVNDAISAFF

RTLGIQLPSPQQQASNHRGW*CPGQP**LDWVIWSVPTEDVMSX

>**comp406429_c0_seq1_3** len=209 path=[187:0-208]

AMQLWDTYPYKASWHFFKPGFHMEQTRDYMLKPFPDHDVFHVSGSWWPNKLQSWSEAALR

AVDLAVRRY

>**comp68740_c0_seq1_1** len=219 path=[269:0-32 302:33-167 251:168-185 454:186-218]

RTTSTKACNTHGCPVDGGWSLWTDTVALCSVSCGGGSQLVTRTRTCTNPAPAYNGQYCVG

DDRTTFTQACNTQ

>**comp44198_c0_seq1_4** len=232 path=[210:0-231]

PVTGFSRAQLAEIRKASLSKIICDNTDTFFVQANAFRKARRYRNPYQSCYALPQVDLSKW

CEATSKKW*TRHRHGMV

>**comp104856_c0_seq3_5** len=2741 path=[1:0-84 13888:85-89 91:90-97 99:98-160 162:161-169 171:170-268 270:269-319 13967:320-337 4954:338-364 9307:365-381 4998:382-452 5069:453-462 5079:463-519 5136:520-525 5142:526-600 14188:601-718 7400:719-738 5355:739-764 5381:765-802 5419:803-855 5472:856-877 5494:878-903 5520:904-907 5524:908-1002 14760:1003-1009 14767:1010-1082 14798:1083-1105 5722:1106-1152 14820:1153-1170 5787:1171-1222 5839:1223-1254 14861:1255-1264 14871:1265-1270 14877:1271-1288 5905:1289-1485 6102:1486-1560 14999:1561-1683 15032:1684-1690 6307:1691-1691 6308:1692-1708 15053:1709-1827 10525:1828-1843 15137:1844-1847 15141:1848-1910 15193:1911-1922 10345:1923-1923 15216:1924-1927 15220:1928-2093 15251:2094-2094 6711:2095-2124 6741:2125-2149 6766:2150-2341 15344:2342-2361 6978:2362-2740]

I***LASM*INA*TVICHSHVAECVHRKDIQQAT*PSVHY*MYNAP*MTGYVVT*LIHIS

PWHAFCGPPYCVVKRLRTALSYHLGDKGCKSSDANSTHRCKDGLDMAGTLLDIVLIVAVL

SSSPLIEAAEGDHHEHTRLDVFEQVLAITGVSNSTFLNETNTERFLELLLHNFKCETTNP

AGCEDAVCLNVSGLFTVVGANISKGLNEEQFNNATVVIFYYIYNITNYCRQNVNPTINTF

EYYREKVQSILFEEGHDDIEAEPIEHALEHIAESIKAAADHKGETNHKEVDHHGHNHGHE

GDNHEENVQIIDAVCLSPDAVFYQLEEEGEHIEGDRLTDVTSFIVYHLLGGSPIKEKCRL

LPSRSNILDDLFSRLSAINNTLTNTELSALLAKLKLTGTKQEDDHSGHNHRKRRNVDIVP

RVKRQTHDGHNHGAVTVPKECYSVGEIKAIHGLGHDDGVTKTKFVEMSPTLVYMQVAGSC

VPTTNTTTSLTPTMTERYGYGSLATLIICLCAVLGAILFPLPKGLCYSAFMAVFLGLAVG

TLFTDAVLHLIPQALGLHNHGESEDAHAHAETSGPVIEEYTWYAVVVAAGTYGFYLIEAV

MAILGSGHGHSHGNSSVELGGLDGSGYVTKQSGGLVMEEKEKKSELSTLAYMVILGDAVH

NFADGLALGAAFSQSVTSGITTSIAIFCHELPHELGDFAVLMTSGMSFKRALCWNFVSAL

TAFIGLYIGLSISTDPLVRRWIFAVTAGMFLYIALVDLLPSLINTVTTHRKLLFVMHNIG

ILTGFLVMLLIAMYEEKITV*EIGSGVLMLCHVVLPSHYTNIH*LESAARGDNSR*TTSL

CFREPLPSAILKVYWLS*KTV*PVILNINCAF*LLEKDTIYWNKKDTNHESFCLVPFMKF

CVQSETI**IYYMX

>**comp30184_c0_seq1_4** len=604 path=[582:0-603]

NGRTGAQTTTSAPLQVGAQTLIEHLAEVYPISKTHVSGQATAQTAPAVGQDRSPVTDQTP

VAIKEIVPVKTVENSVNAKLSHPIEPRLSPVPNTDPYTGTGNMLKPITNAEFGAIMQKML

VQRLRKYISSQRKFQAYNPEP*ETPSPIRQKNTRYHRWMVPHNMTSHHGVTRTASEDVED

ETRLIAECANICSKVYFLK*N

>**comp56242_c1_seq1_1** len=419 path=[865:0-418]

APYKFFPENIDPRLCTHINYAFAKMNGNMLAAFEWNDMSETWAKGMYERTKDHVKAQNPN

AKIMLSVGGWNMGSEPFTAMVGAAVSREEFATTSVTFLREQGFDGLDLDWEYPANRGSPA

EDKQNFIELLKVLNAAFDAX

>**comp96813_c0_seq1_5** len=1118 path=[1108:0-120 1229:121-127 1236:128-160 1269:161-303 2442:304-331 1440:332-567 3927:568-586 3943:587-696 1805:697-718 1827:719-822 1931:823-826 1935:827-903 4073:904-925 2034:926-940 4085:941-957 2066:958-981 4116:982-1025 2134:1026-1041 2150:1042-1071 2670:1072-1117]

EVG*TRQHCVILLRRKMKLYILLSFLPLIYSHGSYDDLKLTGGWKSSDINNDDVQTMAKF

ALATVNAKENSQRQMVSIESASTQVVSGFNYKMVLRVTNGVTDELCTVVVYDQSWTSTRN

LTQYSCKLQTILGGVKPASLDKDAQSAVMFAVDYHNSRTNSLYRAGAQTVERVTKQVVSG

ILYTFTVNMVDTKCLNSDTNKGKGLEDCAVADNPVKTECSYKVWYQAWKKPQYKLESEEC

GEQKDPAPTPVPPPVSNQLPKIDEIMKPMLAGVKLSNSLPQLKDDLSSKLKDLLSKHKTP

RLEELLGPDKTLPLFGLPNQGKAQLLTNKIVPLLKRQKKDYVPDDLHPLLEHIGTGLLGA

FKHAVGGDDHDFX

>**comp508308_c0_seq1_2** len=217 path=[195:0-216]

VQVTADPQSLVNFLAVDQSVLLLKSGNDITQSEVISELKSYDTINNVGGGFGRPIPLGVV

ARRKRMAIPWGG

>**comp89145_c0_seq2_3** len=654 path=[1328:0-27 1356:28-167 1496:168-194 1523:195-206 1535:207-257 1586:258-313 1642:314-316 1645:317-321 1650:322-401 1730:402-455 1784:456-496 1825:497-511 1840:512-583 1912:584-595 1924:596-618 1947:619-653]

TSGGQANAGRGNQNLERSLMQRILTGRLIGLDNQEITRLNSVGTLGVRRANIHRRMKIDQ

IPSYIYYLSLKNKPAQFAKAQTYLMNLYMLENQATDAQLEAMGHIMRQRNQDPDIARMFK

LDAAQGVYGDRLQKAINYEIMQTTRMSLPG*LN*SVN*MHRYDDDDDHHHSPVCVFRFLF

CLYLYICLSRSNISTGSIIQGV*RYHVNVGCVINDK*X

>**comp246916_c0_seq1_3** len=215 path=[193:0-214]

NRLHSYLSRIYRIPESYIPETVGGAMQLWDTYPFRAAWHLYKPGFRMEETRDYMLKPFFN

HEVFHVAGSWW

>**comp104254_c0_seq2_4** len=2556 path=[5910:0-3 17113:4-27 5938:28-87 5998:88-111 6022:112-272 6183:273-296 6207:297-392 6303:393-416 6327:417-615 6526:616-639 6550:640-797 6708:798-821 6732:822-825 6736:826-846 16885:847-849 16888:850-870 6781:871-871 6782:872-895 6806:896-1588 7499:1589-1612 7523:1613-1747 7658:1748-1771 7682:1772-2080 7991:2081-2104 8015:2105-2555]

LLYASVCLLATSELLQRSTIVPVRLLVGRCRIVMVRT*LTEQLVSRSALRERFYSELSPL

*AISCRWRENPLWLRP*TTCG*ASKI*LYAFTMCKL*NLEHSN*RAVSNLPFTSICG*LY

PIQKSWYECQNTCGMPVRTAPTTSSPCVECHTQLCHSQHVLQPSITCQC*YAERKQRIMG

PSV**KVIMSLVSAVWLSRMLSPVDPVSWKQHRR*KQLTSMSVMLWCCLTESRAGPRC*R

AREYTCTVCAHCPRCWMCCRPRRDSQDRILKEFENSLLRTSSML*VALRGMDLVTLTVHT

RRQRRFTTTQTEHPCAPTLSASSSFRSWRPRRVTWLCLPTSPAVARS*SW*TVWAPTCAL

SRPMWTFWRTSHLSLPRD*QS*HRNTTSSSLRTGSLLTLAAQY*ASTVGACTASPTGPTS

QTPIRCRGRESYRDSNRWD*ARTELVS*LQR*AQQEI*QRETTLQRL*RWLRSTVTLSLG

LFVSLVSHLTQAFYT*RQGCS*QKARTALARTTSPPLR*SPTEGRTSS*LGVASPRQQTQ

QRLPGSTKKQDIAPILHSLNEDEETYIG*IHRGRLLQAPAAVGRDGTTFVRHTCISSPLT

RSHPSFFILR*LLTRDHPQQPSCDLSGVYLDRMCKVGLAKRRTTTAETKANLVCIIMGVC

AAAAAAASIVTSWQTLHPLPPVTLGCSTAILLCLLGYDTR*DTTSIAIQGACSLHYCCYK

FEYSVIMPLISFWCLSRGELGPCMSVYQVRHTFVALQPRRYYHHLLTLSVCYGYLDIPIV

AVLNMSLMNFSDFEM*LSLLNFYINSFCTSEDSLKAGMFDFTDLI**KEFTKSSAHKTMP

YIY*RNPWWIQT

>**comp95780_c0_seq2_4** len=1153 path=[1151:0-87 1239:88-94 1246:95-227 1379:228-261 1413:262-298 4581:299-405 4649:406-435 3988:436-472 1620:473-475 1623:476-577 1725:578-611 1759:612-649 1797:650-695 1843:696-715 4790:716-768 2726:769-830 3266:831-845 1993:846-859 2007:860-917 2065:918-941 2089:942-967 2846:968-996 2168:997-1033 2205:1034-1057 2229:1058-1152]

SRAALPSTALVQLYSSLQTDVDNGRQEKMFLLLSTSLLLLAALAPAHAVVTPVDRVVTPG

YQPAPPCLMGEACRHTYSRSYMNNQVFCCPNGQGMSVNSNGFSTHCTCGSNSFVSNANFV

NGQHMRREMRLNMQRFQAEMAAFKRRMAQLGQNLANMFRGGFW*TSRT*VIPVWSNLKKR

NFRVKDMHPPYVIMITFLSTIVYTGIVFKY*TER*LFANHKRFIDLLLVFYHSMFLKTT*

SCQMGMQRVCLS*FF*WCSCSKRIMTCKC*NVVAVSVMSPIVKL*ENKLFTIQFYLPQSL

FSHLF*Q*NFPRFKVKGSKCISV*VSVTLLRFS*LSELFCQ*TLLLMPINNNIYKTLPMR

NKSLQNVKLYLRKLCLYNKGFQSE

>**comp236269_c0_seq1_4** len=272 path=[250:0-271]

AIRPGQCKGNVCNNDQDCPGNQKCCGKPGCRRCYRPEKPGSCPPRKYDAGVCVAYCDGDF

DCPGNKKCCGSCPRLCEKPCFD*M*R*TCP

>**comp100296_c0_seq2_4** len=1285 path=[1:0-265 5597:266-286 5625:287-300 302:301-312 314:313-373 375:374-376 378:377-486 5775:487-494 496:495-793 795:794-794 796:795-810 812:811-841 5887:842-860 862:861-878 880:879-930 932:931-948 950:949-1067 1069:1068-1086 1088:1087-1137 5998:1138-1196 1198:1197-1214 1216:1215-1264 3830:1265-1284]

GGAMKRPRLCDAPKFGGADCVGDTEELKSCNPNLCPIPGDWFNWEQWAACSVTCGGGTKS

RSRKCDMTSHGKLTSPCEGVDEETIDCHTFACTPLARTCSEWGVRGLMSNTMADIDPEGL

MEPTLVYCDMTSENGTGVTVIGHDSEEEEEVVGYEGAGEYVLRVNYNVSLDHAIAIIDAS

ENCRQFVQWKCKAAVIHNPNANGVITTGWRNRTGGIADYFGDATPGSGMCTCGMTGSCAD

PDKACNCDKNDEIWREDSGYLSYKPDLPVTEFLAGDTGADDEAGYATVGRILCTGVAT*H

AQVTTEPRRAPLRSALWNATGLPPRMSAIPNRLFLICFSISSQLMLFATFELKLFS*LGL

ILFPLRAINLEWRLSS**GA*GIRYGVCFLQSGKDCSLQDERFHMLLKHVDC*CYFCYCK

LCLSKYID

>**comp100950_c0_seq8_2** len=5470 path=[1:0-44 46:45-338 340:339-627 641:628-4132 4146:4133-4317 4331:4318-4319 4333:4320-4600 4614:4601-4978 41089:4979-5002 5016:5003-5469]

PNKRDLGCKRALRHS*QTFVGYFNTL*FSEHFKMQLEAREISAPRVGRFKIYIQGDLKHN

QTAVLTVHDLGCNHSMWTNFLTHPSMEEITKRAAFIHVDVPGQEDEAPDLPADYTFPSMQ

SLGEDLVCVLDQLDVKQVIGLGEGAGANIIARFAMAQPNRVLGVCLIHCTGSTANFMEAL

KDKVRTWKLDQIGMNPSAEAYLVFHRFGSFEKAENKEQLEKVIESFQQSLRSKINPRNLK

RFVQAFMKRSNIADNIGKIKCPILMVTGSKASANHPVHVLFEKMNDQISKKNTEMLEVDG

VANVLEERPTRFAESFLYFLQGLGVAGGVPMMRGQRSSSFEGGTPPCRTRSMSMEEADQP

RGIYSMSPPKFGVSPPKMSSSPPTGSVLSSSPTKS*TGTQKIEEGKVGRGSSPCVYLSER

LRLVGPVLFVLVLAGHTVSIFSAASSCGSPSVQTDCGQWY*QTDKCPLQPCYALERLDCC

IGLGLYVLKKSFIMLF*NCRCCCSKGSCSIE*HVPRY*YPDAHVCMYDKSNQSYYLCSVF

LMFCFF*IFLGYVSYWLMKYMS*VGTGWM*WTCWTFLLLA*PGWETRITFTINTAICCKQ

MSIYEHCLCTFT*IVSLQEATMVLSIKSN*YAKFCFILRNEIVISSFKIVCSCCFLSAVM

G*RELPPLWSI*C*KKRILSLVE*KSVVILFG*VVNWV*CCCIMSSVSAHGSIILEK*TQ

YKYSADM*HL*SFECIFVFAHLTPNVL*CL*LNDICLKHSDKCFICCEY*RQRRCSSISG

LYFLCVYSMGDVQLGLLVVGMQSS*GSVPRLIHSMLISSQQFDHTHYMISCF*EGSLP**

HSI*HSTSDLQVECSNFRKFQQNFISIMQLFCQQ*CSLNIIVSPLSMVCKCQMILLDVS*

MKGIFILFSLYTKAFALQ**LLCFLKIYTVCTESE*HQLVISRMFCLNYFVQAL*PNQQL

QGCKILYEN*KSKHLKFLTQAYDDRLSSFPYLSAILHPT*SSVEYTTKQRQHTIYNIVDL

TFLDCGCFCSCQNVRQVQTISADKMLCLNYFLSRLDSCSPVALRSYLNVCRRYIVCLSL*

PTTNWAYLLQIYQLNAFLILSTDQTFFDTVSNKEYTVSNMKVLLSLKYGW*NID*PC*QT

*ICIC**S**QCCSNDCDCTPFILYSL*MFFFCCTYI*YHRIYSTVFYKQMVASFNRYL*

TTCNRSFPISVLTLVIMLCFLLLILFQV*CNIVSFIVVLSYFVGCSWVAGILLKIIIWY*

GVWN*CDKITSCKYTLLCLGRLTRNMSSP*H*ICFLLDLGCVFSLTLNSKF*D**FIFSS

GYLWSLTCSCNGVEILSHLYRHLLYETKVPDM*VSQPQYC*VEL*QLKLIAGVACWE*P*

GVLPHLSYLQNQNKTAEP*WDVECCPCCIFINYGSTTTVGRTRCPAIKIATAPMFVYFDQ

CCYRSCFQVVFEYFEMLLRLWISLVLPETWGVVS*KYCSDHSH*DDFVED*GSDKKLWLL

LEC*NRHDLFMTVDLFVSYVCWFHW*LEYR*QFS*AFCSYNVCSKL*RILWCGDINS*CH

GKVCGDSVSNDGRCEQCSHPM*WSKTSEDVLSQYSGITYCLTCL*SKNCVILVHKPSVHL

QLCSLNCCLLSNVGLLMRSKWGSSLLPPVSASLTNRWP*C*ADNISTLYIEATFFMNILE

ITVKEILNPIFVRQLLLMIFLLMSRYQNVR*AQTVCSLFCRTLIAGHIGVCST*CVAENH

EPNCSQS*TKLLQ*AMADLLCCRTLEHLDIRITGYVHTFSL*LFAICSYLIFRI**I*II

KMK*THKPHYRVIDVKIFEIKYL

>**comp101323_c0_seq1_2** len=1972 path=[1:0-251 4116:252-276 278:277-452 454:453-477 6289:478-602 604:603-624 626:625-729 731:730-735 737:736-759 6432:760-806 808:807-835 6467:836-953 6529:954-963 6539:964-990 6564:991-1268 1270:1269-1272 1274:1273-1651 1653:1652-1671 1673:1672-1703 1715:1704-1832 1844:1833-1843 1855:1844-1971]

WVRVSTLHRSVLRNFCLSRSFSATVSKHRGVVVGVYEGGEGIQLSQAGETINKQCGGQLQ

RQLKQAGRKLKAGKTRVLFGLDEAGFSSVAVVGVGKKGVGYNEQEQVEEGRERVRKAVAN

GMRQLSEVGEVNVDVDPCGDAEAAAEGALLSLFSYDELKAKDSRKPRVQTSCFTDHIQGD

STSTKESWQRGVTLAEGQNLARYLMEAPSNYMTPSIFARLAQDKLNQSHQCTVTVRNKAW

AEEHKMESFLSVAKGSVEQPLFLEIDYQGGAPGDTPIALVGKGITFDSGGISLKPGADMD

KMRADMGGAACVVGTLLAASRLRLPVNIKGLIPLCENMPSSRALKPGDVVRAMNGKTIQV

DNTDAEGRLILADALCYAETFNPSLILDLATLTGAVAVALGSGATAVYTNSTPMWDVIQK

AGTQTGDRVWRMPLFKHYTNQVTHCDLADLNNIGKYSREGGSCTAAAFLKEFVTNKRWLH

LDIAGVMMNKDEVPYLGKGMAGRPTRTLVEFLNTLSRRKLD*DLLTDGTVLHYQGGRRHT

WSDMILHLPCVLLWFGGSCHVWISHEWGA*CWTVYTTPSLIGLTKCYF*CPHYIYYEMQL

HHGKIKRHTIKVATIF*YFNTQIFIKFAFSRD*FIKSDTLYCVPWE*LSGYRVCYEA

>**comp101360_c1_seq14_3** len=1264 path=[12627:0-378 13006:379-388 13016:389-608 20747:609-637 20774:638-639 20776:640-649 13277:650-653 13281:654-713 13341:714-800 21008:801-821 21029:822-823 21031:824-838 21046:839-842 2687:843-889 2734:890-946 2791:947-947 2792:948-965 2810:966-971 2816:972-975 21135:976-996 2841:997-1051 2896:1052-1108 2953:1109-1129 2974:1130-1263]

MADLLGLLSLFLLLLVYLGECKLTVEECKNLGFSTNLLCSNCEELDKFKLLPLKDSCQEC

CQEDRTEEEKAGLYPYAELIVCG*KLGRYPQVQAFVKSSKPDQFPGLEIKYARGADPIIK

LYDENRAVREELGIEKWNTDSVEAFFHAKLKK*THHNNFEMSHYEHHSLVHF**CFVHFL

QSMSFLIALECQSLSS*FHKMHSIFCYFCTYFPFYLSF*PVCCLPLILKPTEQDICEV*L

SSK*LTQI*WQVVPFQFHCQAVMATGESIFS*KLNIVSSNSTDLDGSKNLSI*NKIC*NL

SISGTLSSMCQGS*FLVTEKLCFLAVIKDVIYSEYG*EKTKVNPRMLAS**DV*YAYL*S

PCMKPDGLWGWMTEVLLEDISELLHHCPSHFVSRPCLRYEKVYLSCGFITLFTSKWKVDM

X

>**comp101672_c0_seq1_4** len=624 path=[602:0-117 720:118-202 805:203-209 13283:210-226 13300:227-227 830:228-247 13367:248-323 926:324-366 969:367-385 988:386-390 993:391-459 1062:460-488 14248:489-494 14254:495-501 14261:502-512 6623:513-560 1163:561-623]

FVPDLPRSAARQNVRVVSSPSYTALGAAELKSTAMQVEVLSVLLCLVATTYAGCPQGFHQ

HRRSCYWFSTIKSSFAEAAGYCRYLESHLATISNRNEDSFVRGYAIRHGKAFNYWLGASD

LNIEGRWLWEGQRRMNYTNWNPGQPDNGGGNEHCLDIRRAFEYRWNDYVCQTPSNFICEK

EL*HVCFPQIPHQQITCNAATFCKVKSS

>**comp101977_c0_seq1_2** len=4897 path=[1:0-314 316:315-330 332:331-1905 1907:1906-1923 1925:1924-2061 2063:2062-2078 2080:2079-2167 2169:2168-2197 2199:2198-2324 2326:2325-3263 3265:3264-3767 3769:3768-3798 3800:3799-4336 4338:4337-4642 4644:4643-4645 4647:4646-4696 4698:4697-4739 4741:4740-4793 4795:4794-4830 4832:4831-4849 4851:4850-4854 4856:4855-4873 12724:4874-4896]

KHPDDTTPRDCGKYNSRVLDGTYRR*IMTKINQLVFFVSVFILVVCLVEFIEGRGILRRK

HEKKQARRSDDSVEIYFKRKSPENTDVVCHGSGCKRAEEHRRRHHHGKPKNMKKRKHGKN

KKKRRKNRQRKEKSKMAAVSLEDMFQSEDVLADHHERASYDQYYAGFGRGFDRLQRPPEA

DRLERRLHPSVGSSAHRLVSSIDRVIMALKSNIHQMHDTYDFAFNHTRYTMMMGVRHELQ

NIAQANDMVYDVVMDISLLTKEIYMRAHRRGDTRPRTYRKPDMPKFSSFRENLESVRNAE

RLTKAEFDHAQNMVDECQKQASYVLQLEEILSENINERELSDLVRHVNMTVWKLQAEFDT

AMVARKMSRFSLTNGGTARDDVITASNLVRKAKEVLRDAAATSRGNLIAAGDLRRQKGGG

LNPLAPVSFTDRRPHGRSKSRSRSRSRPRRSTYITTAERDRLRPIIDRAVNHANELEQLA

RIIARLMNPLIRQATNVVRGDRGGGVSYRLQATLLADRLLAHVQQIHTVLDRIRLAINDE

GELRRRCEELETGSGDDGDTTPIDCDEYIDVGSARGTDTSSPDPDGDGSDFIFDEDQNPK

RIPANSLKNFGDKLRIHVVQLGQKGDITLVKSQTARNKSRAFSTRWNAAKVNLQSTRGFV

DEAKQFLNEWRTERPNIDRISDRIKDVHERVDQVYNITQSKTFQAVLKAERKVKEVKGGG

GTGGTTKEGSGGSESQDYAAALEAKGKAQAKMRSILTLTRLAPSMVTGVNVTVGQIQANI

SSLRSKVEKARMMLASIQMSINKQDEGYLPLPLAASDESLTLRTSVEVCIRPEVADGQIL

LVSGNNSESYSLSMSSNSLTMKVYDKGGYETGDVDSTISLEKDKWYNVLIRRNGQKVALM

VRPEGSDDADVVEDTVDMPFGQPPPLAAYVGGAPDTVPMYLNSDLWKGCVGGLRVNGQSV

GLLQKTSDGKVPAPCTSSCSPSLQPPSMVFEGNGYAHYPVHTAQLRHRTRSVYFEFRTRQ

KNAFLMTVVKRGFQLRAQLQDGTLYIETTTPSEAVVLIEENSKYNDGMFHTLKIGYRDSK

TYPEIDGKEGLFSIERTRPERIQTPTQGILIGGMLPEHRGRMGDKFRPLIGCVRKLQIND

VTMTMSDVERVEGVYIGSCTSNKPLLTCVQFVNTSSPIAYGTTEDAAAVVIVATRASRGP

ILQYQKGDSFNAIISMEENGIVIAESVKSNDGRLDYAKDDNSMFVVIKVTDTGSTLKVEY

QDKEASISYLNWWETFTGGESKTPYTVTIGGQDEDNNLPQFSGGISQMIVGEQYLDMTKY

VTDNGLSSCPGARISDPVSVDVRRR*GTSPSHRTHYVHSCNLSAAKLSQPRAFPSR*PKH

KMRLTHT*IWTYIFFISDDKFASFHNIIS*KYQNIKFIRELRC*NMCDLLNCYSVHSW*E

PARSCQKIHNCP*YCPTIL*NRMLLNKTTLVL*LP*AMLRYGS*GHLSAKMALYSGTLAN

IFKHKPRIFMNIEHQISC*SCN*PWKAPYRDMMYTFPRFPGCKCK*LTVGFDNTFMLLTW

**NNTVETCSVLDMASCPGGVCGTTCQ*YHSTCQRTMNLKQPLTW*TCATFESNVGT*AR

SCLNTTRTHSFC

>**comp102102_c1_seq1_6** len=848 path=[4234:0-520 4755:521-544 4779:545-847]

RTPDPPRISHCSESTIRNFTPPPFVLFIIHCYCFLTFLTWMWPKIHFVISLWPFPPSWGR

VICTVLFITTTDRLAR*PD*EVQLQTCLVSRGERMRVGFCPSCR*IKMSETDKAQAATPG

GDTIFGKIIRGEIPTTFLHKDDVCVVFNDISAQAPVHFLVVPVKPIVRLAEAEDSDKEIL

GHLLLVAKKVAADQGLTEGYRVIINDGQHGGQSVYHLHVHVLGQRQLEWPPG*MDALTQA

RVISVDTFLSSYLCVPFVSVS*SDPR*CNCITESLAK*NVNMX

>**comp102323_c0_seq5_4** len=869 path=[5175:0-26 9565:27-36 9575:37-46 9585:47-373 5549:374-386 5562:387-868]

TMADTGVLAWISVIVVTCLTLSRANQYARGWGDHFDWVNLQDGLAKAKEQNKPLMLVIHK

SWCGACNALKPRFAETKDIFSLSKRFIMVNTENDEEPSEKQYSPDGGYIPRILFIDPEGK

VRKEFINEDGNEKYKYFYPDTEDIVNSMNKVLEAVEDGKFAAGKSKTEL*LSHVVEHGSV

HGPVSSLGVHL*LDLVLSYITLNFKHMFLMKI*RNFRGLCFRNIIIHKK*CG**NRYLSD

LYSL*SAIIQRCPTFDKHYT*SQIAVV**REVSYILREKNKGTQENSSI

>**comp102372_c0_seq1_1** len=2299 path=[1:0-658 660:659-674 676:675-757 759:758-779 781:780-953 955:954-1156 1158:1157-1175 1177:1176-1219 1221:1220-1223 1225:1224-1348 7304:1349-1355 1357:1356-1373 1375:1374-1398 1400:1399-1425 7322:1426-1437 1439:1438-1441 1443:1442-1465 7357:1466-1481 7369:1482-1851 7568:1852-1861 1863:1862-1866 1868:1867-1941 7634:1942-1964 1966:1965-2022 7661:2023-2048 2050:2049-2051 2053:2052-2298]

SYFRTTLLGHAQ*TRKYSKRAFVVM*H*PVGNEPWGATTVVSLLTQNKMLWTVGVLMGCV

VVSVAQTPKYNESCVRGTYPPSIEKKVKTYVVNLDLPPEQRWTHVVEDKAVQIRNILAEF

KKYALDWTPKAQGVIDWVDNNFPTLDKTLPYPFPGEMSGISKASGLKLGEVVLYNLFYEF

FTVCTSIVAEDPTGKLFHARNLDFGLFLGWDIKNNTWDISEFLRPLIVNIEYTRGGKTLF

KGVHFAGYVGILTAVKPSLFTLSMDERFNADGGFIGIIEWVLGDHSGSWMGFLTRRVMEN

ATSYAEARDWLTQTEMLAPAYFILGGNKSGEACVITRAREKTLDVWPMSSAGGWYILETN

YDHWEKPMFLDDRRTPANKCMHKLTQQNVSVNGLFDVLSSIPVLNKLTTYTALMQVDSGH

LETWIQYCKDPCFPW*PSTSRFPWQPSVSTGWSILSFLICSRMMSFVSYRSFRIQLLDRC

*GCGLKCYHGNMSMYHRCSHHDVIFKAYAICTSANWNKVYFTTVIIQASNVLL*FCLIQV

LSLFLLFTDTDSCAI*RIMHNVFFRRF*GQELSL*S*VGNYLTLICKKHSWGKGDVLLA*

SQHVFSFCGKSVGLSL*CVIFTCGTVNFVLHLF*IRVVMEVSE*CNGFHL*EK*LRKKVL

ILCQKN*CIRN*NIAKF*NNKLFHKIT*SEI*TYTEHTIRKIT*QTNLLFKLSRVTSLLL

STLVGMAMISTIIETEVSFCIIYHFDISLLPRCHDSCTHIVLHCK*X

>**comp103038_c0_seq1_2** len=1286 path=[4393:0-220 9263:221-240 4634:241-375 4769:376-385 4779:386-785 5179:786-793 5187:794-1262 11149:1263-1285]

NG*RRSDRS*L*IDP*KIVSLMWIFI*PSEEFTKGFLRNLSITLSFKSSP*QLATDTMRT

FLLVFICSVVQLINLSEQKSLTDYIKHYEELRLDPVSIHRDHERVRRSLDSHLYLRFKAF

KRDFHLKLEKDTSVYSPDHVTSSFDGHLQPVDTSFIYQGVLEGVPRSYVHLSIINGTTRG

HVHIPGETTYHIEPAAQYMQNPHFHSVIYDEQHVDLDPYRHRREAESATCGNDHHFEWMK

RVSESAYEAPNRAKRATENYEEPADSYNKYSEALNRQKRAPRNLGGKNTCYLYLQSDPML

WNYVKKEKFPRTTLSDERAKEEILAFFASHVAALKSIYSTTTFSTYDDSISYKGINFLVQ

RTRIMTDTTEKCNTAAATAWCNPNIDVSNFLNLNSMDNHDDFCLAYVFTYRDFIQGTLGL

AWVGSETKX

>**comp103183_c0_seq9_3** len=3135 path=[1:0-35 37:36-697 699:698-748 750:749-772 774:773-1144 1146:1145-1146 1148:1147-1569 1571:1570-1570 1572:1571-1594 1596:1595-1653 16955:1654-1675 1677:1676-2554 2556:2555-2693 2695:2694-2696 2698:2697-2716 2718:2717-2979 17168:2980-3045 14746:3046-3059 12402:3060-3134]

QSQRTCVCDWAFESRAEPAEGKQDGVCEAGRPLGHD*YHIPQSQTISV*SCRYDVFD**Y

IAGITRRFCV*AERI*IQLRRTTDSSNLELERNTLFV*ETLTKTLTMLTFVCYFVLSVCV

HLVSGKKAYVQLTDTSLTDSSDCQLPTTIHITVTADGRSLPLTLTKNTDVDVNAPIYTTS

VFRDDEVEKENLFDIPDSAEYQDEAQYAAMTVECHKLDKRVDISVRGMITLNGKQYTIER

RNRQRRDTDATSDAGEYEITEDVAPVTSNDYREAPPAVEDAVRTIKESHQLLPLKDATLT

RHKRQSSQDVPLKGANLYRQKRQVSQYYIDVLALVDFGVYKRWYDVSTQPTAAQKKIETL

QNIRRYYAYVMNGVSLRYKSISSLNSRINVRLVGYYVAESEASSPFTESFRISGSSGSEV

DADKVLVYLRDWVTRTSLPANDHVMMFSGYDLYTINPATNTKSTATSGLAYISTMCRSDG

RSISLVEDIGGFQCIDTAAHELGHGLGAKHDGDGNNCKPSDRFIMSGGSYKTTDQNEGNP

WFFSSCSVDYFRNLLASLSRTSTGIQCITSALPVTNVPDTSSVLPGQLYNPNQQCQMIHG

ASSRLCMGPEFGNYSKICSNMFCLDPSSVSTCFLHDAARGTTCGDRKWCIDGVCQYAPEA

PSANENCVFGDQSGVAFEGQTCEDFTGGDNSPYCYQQVVRGRCCASCNRYYTGVESCLYG

NTVRGCDARFCDYIYDDGTRYADNCCQTCGQVVPISTTTTTTTTRAPVTTTMTTTKTTTK

TTTKTTTKAPVPTTIPNTQGDDCTGNLGDRNGITFNGMSCADYVNSDPGVCYSESTQNYC

CYSCRQRETGIQGCMYGDWFSGCVDEACDYIYSDGTPYRFYCCDTCRGRPATTTTTTIST

TTTTPTTTTPTTTTTTTTTTTPSTTTTTSTTTTTTTTTTPTTTTSVSDRCSEKIYGVSCQ

AYIGLYGKGRCYNSDVAKACCSSCGAVSNPSKPGTAQTNSLTKH*P*LHSNNRCS*FATL

CYNITIPQNQVKQK*TLKQRGDVLX

>**comp103384_c0_seq2_4** len=1187 path=[1163:0-170 5826:171-172 5828:173-203 1367:204-205 1369:206-224 5878:225-251 5905:252-305 1469:306-345 5962:346-377 2887:378-401 6002:402-403 6004:404-411 6012:412-414 1576:415-415 1577:416-440 1602:441-475 6038:476-481 6044:482-488 1650:489-521 1683:522-525 6072:526-539 6086:540-575 6105:576-589 6119:590-596 1758:597-605 6131:606-615 6141:616-623 2504:624-660 1822:661-685 6160:686-749 2426:750-799 2476:800-804 1966:805-810 1972:811-878 6291:879-881 6294:882-898 6311:899-904 3180:905-923 3012:924-930 2092:931-980 6371:981-1046 6427:1047-1049 6430:1050-1065 2227:1066-1145 6464:1146-1186]

TQ*AHDRRTLPSFDR*PADENIMVSRLALICTLAVVLYYPRALAQLRTAKNCCLPKQWEG

FQGGIGGVETDKGGQAVQTIAKISFDAIGRRVYTYSNSTTAGGMSSTKLIQDYNTNTMYV

IDVLKRTCKKTKTNLPFNNGCVPDDAKQVFDMTIGAGSETLQTKIYSMRLQQASTLLDIN

FSVTEKQCVPVGENWVGSVNNVKLVMSMGYFGITPGIKDPSVFNPPAFCKQNITGDAAFH

PVLNYRILGL*MRRCHHSNIAAFIIQILICLKLFTNFTDRDTLLVPISSNTTRLFDL*KH

DVIMTSPLESLHSLIL*ASLLIV*HYISISV*ISSYTMWMSDL*NHCDVIMMSLLASSRH

LQEDHCSPSPETT*VGITLIMLTLLCISFTKT*QK

>**comp103470_c1_seq14_6** len=1269 path=[18001:0-239 25907:240-242 1593:243-278 25943:279-283 25948:284-287 25952:288-331 21354:332-362 1713:363-373 26024:374-391 1742:392-446 26090:447-450 26094:451-454 1805:455-473 26103:474-491 26121:492-497 26127:498-503 26133:504-511 1862:512-535 1886:536-578 1929:579-604 26207:605-621 26224:622-628 26231:629-633 26236:634-634 3334:635-637 3337:638-695 3395:696-698 2049:699-713 2064:714-737 26301:738-742 26306:743-760 26324:761-766 16718:767-797 26407:798-805 16757:806-809 16761:810-839 2190:840-871 26452:872-872 26453:873-884 26465:885-926 2277:927-992 26494:993-997 26499:998-1000 2351:1001-1019 26514:1020-1049 26534:1050-1052 26537:1053-1114 23610:1115-1127 16350:1128-1130 2481:1131-1183 26617:1184-1192 26626:1193-1207 26641:1208-1210 2561:1211-1234 2585:1235-1241 2592:1242-1268]

FAGDDAPRAVFPSIVGRPRHQGVMVGMGQKDSYVGDEAQSKRGILTLKYPIEHGIVTNWD

DMEKIWHHTFYNELRVAPEEHPVLLTEAPLNPKANREKMTQIMFETFNSPAMYVAIQAVL

SLYASGRTTGIVLDSGDGVTHTVPIYEGYALPHAIMRLDLAGRDLTDYLMKILTERGYSF

TTTAEREIVRDIKEKLCYIALDFEQEMATASSSSSLEKSYELPDGQVITIGNERFRCPES

LFQPSFLGMESAGIHETTYNSIMKCDVDIRKDLYANTVLSGGTTMYPGIADRMQKEITAL

APSTMKIKVIAPPERKYSVWIGGSILASLSTFQQMWISKQEYDESGPSIVHRKCF*ATDC

IYYET*SAPSVLRSGGAPCGGGPISEGPPAGFPTAVGHNERQFILLFHELLLYFLKEKKN

K*X

>**comp103717_c1_seq2_5** len=1575 path=[17181:0-94 3209:95-106 3221:107-112 3227:113-118 3233:119-121 12056:122-129 6840:130-130 6841:131-145 6856:146-151 6862:152-166 6877:167-193 3452:194-223 15079:224-226 15082:227-259 310:260-280 331:281-283 10631:284-322 373:323-385 436:386-511 562:512-538 589:539-544 595:545-553 14119:554-577 628:578-583 634:584-590 641:591-619 670:620-628 679:629-682 733:683-691 742:692-699 3539:700-706 3546:707-709 3549:710-710 3550:711-714 3554:715-715 3555:716-829 3669:830-856 3696:857-901 3741:902-949 3789:950-1038 3878:1039-1045 3885:1046-1062 1113:1063-1063 1114:1064-1069 1120:1070-1072 1123:1073-1090 1141:1091-1096 1147:1097-1101 1152:1102-1114 1165:1115-1120 1171:1121-1126 1177:1127-1132 13195:1133-1144 10769:1145-1156 1207:1157-1159 1210:1160-1213 1264:1214-1228 1279:1229-1237 6389:1238-1254 6406:1255-1278 6430:1279-1279 6431:1280-1303 6455:1304-1321 6473:1322-1351 1402:1352-1354 1405:1355-1359 1410:1360-1366 1417:1367-1574]

VPLCVRSV*IPRHLSRHRQINLALTTDPLLSA*VAWTVRPNKQRYLHLNISNPL*FNMRE

CISIHVGQAGVQMGNACWELYCLEHGIQPDGQMPSDKTLGGGDDSFNTFFSETGAGKHVP

RAVFVDLEPTVVDEVRTGTYRQLFHPEQLITGKEDAANNYARGHYTVGKELIDLVLDRIR

KLADQCSGLQGFLIFHSFGGGTGSGFTSLLLERLSVDYGKKSKLEFAIYPAPQVSTAVVE

PYNSILTTHTTLEHSDVAFMVDNEAIYDICRRNLDIERPTYTNLNRLIGQIVSSITASLR

FDGALNVDLTEFQTNLVPYPRIHFPLATYAPVISAEKAYHEQLSVAEVTNACFEPANQLV

KCDPRHGKYMACCMLYRGDVVPKDVNAAIATIKTKRTIQFVDWCPTGFKVGINYQPPTVV

PGGDLAKVQRAVCMLSNTTAIAEAWARLDHKFDLMYAKRAFVHWYVGEGMEEGEFSEARE

DLAALEKDYEEVGVDSVEGEGEEEGEEY*TICHMNNDELNKQINX

>**comp103784_c0_seq4_5** len=1930 path=[22454:0-622 6128:623-751 6257:752-1488 6994:1489-1929]

LSCHRTQLV**TCSAYDTQIT*R*QAALTFIRLHKPAVNQGTFRLIQ**TLRKPREIPSI

FH*QLWKRHLRLVVAKATGCVKYSLIPCGSSSMRQNPVVVLL*YC*RIALTCT*RGLYNL

WLYSKHLFVS*SHKITFISDYYCGQSVQVTEDSPMALVVVSLVLGLCMGGLAAPAPDTSK

QIDLLMPHVQPKVPDTYLCHGMKLNSSETYITGFIPNANMNIAHHMLLYGCMEPGMKKPV

WNCGEMAASSSEFDMAPTCASGPKILYAWAMDAPSLTLPKDVAFAVGGKTDIKYLVLQVH

YKNVTTFLPPQNNKDASGLNLITQKTKLPRRAGVYLMGTGGKIKAKSTVYMETACKFRDD

LTIHPFAFRTHTHGLGRVVSGYRIRDGKWTEIGRMSPQKPQMFYDATTPNMTVEPGDILA

ARCTMVNDKDTTVNIG*VAHR*NITTLNIQLDRATVVELKTQI*YRMNFIPKKIPAISPL

GSIMFDVCSRSVLSVKDVYSSSTSMFTAAVDAEFVMATLNPFSDGFIRR*TLKG*ILTAG

SRLVSMCCVCASRLGSRCLSAHPFSSSFTLRIFTLSVSYPCGQILLYNTCFLLSLFCGVC

V*FIVCLI*FVVLSNCN*LLVVLRVLFFGSSLTQ*LNKTKVEVX

>**comp104129_c0_seq10_1** len=1144 path=[2754:0-23 9568:24-430 3225:431-466 13313:467-484 3279:485-486 3281:487-540 3335:541-578 3373:579-640 13439:641-680 3475:681-712 3507:713-878 13652:879-892 13666:893-895 3690:896-913 3708:914-1079 13743:1080-1087 13751:1088-1089 13753:1090-1118 13782:1119-1130 13794:1131-1132 13796:1133-1143]

GVSHLAGHFPLLNACAAHAICKRIHQHNTSFQGYLA*LRSTLLRSL*ISLKSVCHNRTSL

YT*NELLGLISNTSNIPVV*NPIFSYLYIHLLIQISISVFVYHIVTIDDAQKIYTEPI*T

TTASF*IFLSVFSIISTTHVS*TVKMRPLFMVDVIARVMHNLS*RGRTTNERAVVYFHLW

GT*HVVCAGNKGTCTSSLLGASTGAITTSPSHLQERGTLYLITKTDL*YFSTMMKLSVVV

LVLFVGACTAATYKVNTNMTYVEFMTKSLMASKQADISKDKNKLTVMFEDLQQIKADNST

IAGLNNSFAEATFMFENATDQTTAPTSRRLVGTADVFGGKFKVYIDVFTQDGIVTVDGEN

TTVMSGDVRVMVELTGLLFDDX

>**comp104222_c2_seq1_2** len=2062 path=[1:0-128 11134:129-136 11142:137-428 430:429-438 440:439-524 526:525-526 528:527-1102 11701:1103-1103 1105:1104-1125 1127:1126-1160 1162:1161-1486 1488:1487-1497 1499:1498-1689 1691:1690-1714 11877:1715-1720 1722:1721-1737 1739:1738-1768 1770:1769-1784 11910:1785-1901 1903:1902-1927 11971:1928-1928 1930:1929-1952 1954:1953-2008 2010:2009-2033 2035:2034-2061]

VIPFSDKQITFRNVPITLTSSDCR*DYL*V*YPSY*LSVFCFNSTESYMMQISFALTLML

CSVVGTLASECSLPPSLWCSSNDVADACKVTEHCRRTVWKPMRGPAPPVNFTLYYESLCP

DCRNFITTQLFPAFTAVGSIVNLTLVPYGNAEEKKVGDKWVFQCQHGEEECAENLLDTCL

LNIVNNINIAFPFIHCMEEGPERSRAAFRECAKKFPSIPTSQIEACANSSQGNQWEHEMA

TKTDALNPQHQYVPWVTLNGVHNEKIQNEATTNLIKLLCDTYTGTKPSGCKQRQATYCAR

K*NSLNQLRIKVTLCKNINIHGRNNLPDLS*SSPFFY*YISHTCMLPLVVLMSSYVNSQK

SISEKNMIFFQIYFGHQTIRFWLKKDYGDN*KRDFVAIS*QIILF*FVVSVNMLQSLCSR

TVCVNIVI*PLVCDIKKQPNAAK*FAILFMLSLQIYINLYNLFNFLLL*TLVLFANSFVT

EFSHQVIILMLVLLHHKMAL*KTTCICKWRDTQVYNQIKRHDLELSQPAADRMSVHLYHR

NV*VLMSKNIFRHQKMLIPFNIYVLNFFVLFADKTFILLFTE*LILKHRTQKP*CLDILF

LLLCKSCLAILSSPSSSLTCRITWYIQPSGRTYSLVPNASKWLCRYTEGKKWGIK*RRGC

SYIIFQGFFTSFASDNL*KKLEENKGI

>**comp104510_c0_seq13_2** len=1751 path=[15872:0-24 4431:25-449 17172:450-450 17163:451-459 4866:460-482 17184:483-548 4955:549-570 17294:571-590 4997:591-612 17329:613-656 17360:657-659 17363:660-661 17365:662-662 11087:663-668 5075:669-712 11320:713-713 11321:714-1151 17586:1152-1163 17598:1164-1173 17609:1174-1180 17616:1181-1192 5599:1193-1250 11616:1251-1271 5678:1272-1300 5707:1301-1311 5718:1312-1378 5785:1379-1424 5831:1425-1464 5871:1465-1470 5877:1471-1591 17829:1592-1610 17848:1611-1615 12105:1616-1631 6053:1632-1648 6070:1649-1672 6094:1673-1690 6112:1691-1695 6117:1696-1705 15645:1706-1750]

GTVRVTALVSGRCAPKPLCMMACPYGLATDDEGCEICKCAQPIVERRQVKCPPKAMCMMN

CPNGFATDDNGCDVCKCKDTVCPDVMCMMNCPHGFERDENGCERCKCAANPVCSQRPMCR

MSCPFGFEKDGEGCDVCRCAANPACAEKPICRMRCEFGFERDQEGCDVCKCAASPVCAQK

PICRMMCPHGFMKDEDGCDVCKCAPSPNCLNKPMCMMFCPGGFEKDDEGCDICKCAGRAI

SAAKCPPKAMCMMNCPKGFKKDANGCDTCSCAGDSCPPKAMCLMMCPNGFEKDDNGCETC

KCAASASLSDKCSPIQCYKNCPFGFRKDRKGCDKCKCYWPRPKKICRKVRCRMYCPNGYR

KNRRGCKTCRCRW*RQHCAMVTKVEPAIEARPMRRQLPRGSYVRTHRVKCYSTDSVNLLY

MLLSE*IT*CGMCKLRCSYHI*IYIGRLHRGGKHM*ILRSGFGYEIHMYF*L**GSV*NR

ETLRADHIMMLLLR*FTL*HTVSRSPGITSCRYPQFVTRDVEVYKTGYITITKKVLDDRC

RLPFHKVVVAKVIITPYSVTTLVHFRSCCGTCPDELKPD*RIVX

>**comp104636_c3_seq1_3** len=2629 path=[11413:0-1025 12439:1026-1039 12453:1040-1206 12620:1207-1289 12703:1290-1304 12718:1305-1529 24159:1530-1587 13001:1588-2179 5560:2180-2185 5566:2186-2261 5642:2262-2628]

CFLRSGKIMSYELDYTQFVSI*AFQKSFNIKMSSGKRVNTTATARLKQDYMRIMKDPVPY

VKAVPLPTNMLEWHYVVQGPENSPYEGGLYHGKLIFPREFPFKPPSIYMITPNGRFKCNT

RLCLSISDFHPDTWNPAWSVSTILTGLLSFMLEKSPTLGSIETSDYTKRQLAAQSGTFNL

GDKVFCELFSDIAENIRAEIRRREEEMQRSTDSSGNIDGGSRNGRSGLLNGNPNTVLNGQ

NQGFFGTALTNIFVIIGFAAFAYTVKYVLRSVVD*RHELFGCCDLSTLTLDQRSKECLYI

LTGSGVSLYLSICLQMCCIRCLDQRYPLFCIMS*KDLLSVCTYYFCFFYQ*FVMLQK*EE

QCFIQDARLLSRVMVKTFIVLPKSWVQFLNGNPHVETIRRSRKKINHSNSWIVFDRDIIF

EIKCI**FRICMNALSLWIKTWGWDTRLYKNEIFVINERIIEKQ*KYYTMYQYCQ*KQIR

VDDLNLTSSECGLGWLRGSSCALWVGES*CALEGIGELIELW*VRED*GCP*RLE*LDGA

LCK*DYVPLRGSGR**CPYEWERVESALGFAGA*LPF**KARAYT*LLLKQLGKHSILSK

YEVLQSIGLARKEWLAMTQVWEGVDVPL*LGH*LYPGGCARGYGAC*GVSKLMCPHY*EG

VVDALESERKLWIVFVCEVIIFYTS*CVTLHKLCCCFHHLPWWSLKS*IF*CKILSKDML

*FETGETNGIGWSVSLTWLTHVVVSQLRRSMLMISITGVSGLDL*TSCQSAGLLQWAEFN

NNELLH*L*PSFCTDNTMYLTKAVFSKFITVSCHFCMYACPTF*GIMSSCLTRFDYFIF*

CL*AHIFL*CQSNLSSCLLAISFRTATLLE*C*KQX

>**comp104658_c0_seq3_5** len=1186 path=[5607:0-76 5684:77-101 5709:102-189 11333:190-195 14830:196-208 5816:209-229 14857:230-238 8222:239-258 5866:259-295 7928:296-297 5905:298-474 7978:475-496 6104:497-567 16297:568-582 6190:583-705 6313:706-727 6335:728-887 6495:888-895 6503:896-919 6527:920-1044 6652:1045-1073 4444:1074-1132 4503:1133-1185]

LGFVLIDHCS*L*FRF*VLKWWKMSDGSKHAK*TSQFGNLISLPPNQCRNLNHIFLV*WA

RKVVTCDCQSSPDLFESLLLSDHRSLFHVNTTRGFKPNMR*HCSKSKMSLTGLAIVLLCL

PVVTEQLSFLPDTYEQSRVSFTSGDDVTLTCPVTLHPGEDYEQVEWTFLSCGRYFFMNPC

YERSVWSREQGLGIQEGPEISKIPQDSSVKAVATVKNITNGYYLCRVVGTGSTDQSHLFD

VYVLKNASAVPTIRPDPIFVVKGATGSVQVTCKDEAYVPGSIMRIYHGSWSFRATFHELP

TVCTITYDPKTPSETVSCQAEVPASILSGKFTLQNSIHCDVIRPGGKRISLQRRARIVDL

GSGSQTITI*CHAQGQILFDARHI*K*IHN*KACRX

>**comp104761_c0_seq1_3** len=4343 path=[1:0-921 923:922-938 11856:939-960 11865:961-1116 1118:1117-1150 1152:1151-2160 8818:2161-2174 2176:2175-2437 2439:2438-2610 12166:2611-2631 2633:2632-2681 8768:2682-2683 2712:2684-2739 2768:2740-2759 2788:2760-2836 2865:2837-2877 2906:2878-3257 3286:3258-3263 3292:3264-3299 3328:3300-3321 3350:3322-3389 8980:3390-3406 3432:3407-3463 3489:3464-3768 12569:3769-3769 3795:3770-3788 3814:3789-3813 3839:3814-3876 12637:3877-3890 3916:3891-3895 3921:3896-3906 3932:3907-3980 4006:3981-3981 4007:3982-4017 4043:4018-4038 4064:4039-4115 12741:4116-4135 12761:4136-4155 9748:4156-4156 4181:4157-4162 4187:4163-4265 4290:4266-4289 4314:4290-4342]

ITRVKIT*FRNINMAGGMDRARRMPKIGDNEKESMFGYVYAVSGPVVTAQQMSGAAMYEL

VRVGHAELVGEIIRLEGDMATIQVYEDTSGVTVGDPVLRTGKPLSVELGPGIMGSIFDGI

QRPLEDISELTQSIYIPKGVNTPALDRSKKWDFEPLNIRIGSHVTGGDIYGVVYENILIK

HKIMVPPKAKGTVTFIAEPGSYDVNEVILETEFDGERTKHTMLQVWPVRQMRPSADKLAA

NYPLLTGQRVLDSLFPCVQGGTTAIPGAFGCGKTVISQSLSKYSNSDVIVYVGCGERGNE

MSEVLRDFPQLTMEVEGKPESIMKRTALVANTSNMPVAAREASIYTGITLSEYFRDMGYN

VSMMADSTSRWAEALREISGRLAEMPADSGYPAYLGARLASFYERAGRVTCLGNPSREGS

VSIVGAVSPPGGDFSDPVTSATLGIVQVFWGLDKKLAQRKHFPSINWLISYSKYMRALDE

FYDKNFPEFVPLRKKCKEILQEEEDVSEIVQLVGKGSLAEPDKILLEVAKLIKDDYLQQN

GYTPYDRFCPFYKTVGMLKNIISFYDLARHAVESTAQSENKITWSIIRDQMNDILYKLSS

MKFKDPVKDGEAKIKQDFDELYEEMQTAFRNLED*TKVKVTRPCDTKAVFLMWTIFLC*N

TPRRYNNTESPCGCFIKRHRIKC*A*CLLSRN*LRLFGFL*SSLFTVRSILV*HF*SSIK

LGLTL*FCVYLTSEKYWLHSFHNITDCIPGMAVDSGYFVNMDVL*LNQDSKSLYV*IEHL

HRQI*LQQIVYPISASTHRSMKGIWNHSKYFIEIW*QFILGANYSAFTLSAKDISQFLKE

TLVGYY*HLIA*LFLEMEQKFVLL**LRFC*CIC*QDSFVY*LHSCYQD*FIYYILLS*Q

HIPAVSHF*PDISVYMMCNGLL*VKYFMNICKHFLKML*LVKCEK*ALLKFKVFHYTNDS

LEQVKSYD*NPLFL*VNDKL*SFVLRLCMSTTFPKCCLCLDLNICL*EFHPRVLVVEKNY

SVY*PFHPIIDSWNHCLHRMFL*KYCSESYY*KFCLFGGEN*GHYVF*MIYDIKYDIKTL

*IKNFFI*FYYNMIDLCLH*TFWIFTRLNVCSSIVMTKFKLYIL*ELSLKSKQNVCKSFS

KLGFLVLIFRIY*QTDVYHLFPEC*LHILYESPR*AFVEVCHFVIYFMKLFVEAALRQVH

PVASHAPHSNPVLYSTINACSFHDIHFALMQRIVYT*CFFNNECCLIFITSVTV*FRDVH

IL*VMHYVQESTVWYFFL*DTSSICCTKRFWRYRNGAFTIVTMVLADLGQNNHPCNVIMS

DLKQNQPNGL*TCPHANHSAWKTVKVLVMSSLP*FVFLTWLSPFDSYQ*SAHWYGIVK*L

PRIVKYSSDIFLMTKRSLAAAVASPSLGV*RAHHYPPVRIVPHLASSL*KGGVDRL*YYH

YKINMLE

>**comp104903_c0_seq19_5** len=2367 path=[9722:0-35 15312:36-51 2461:52-74 2484:75-83 2493:84-107 20228:108-110 2520:111-122 2532:123-135 2545:136-177 2587:178-179 2589:180-300 20334:301-316 20350:317-318 20352:319-367 2777:368-447 2857:448-508 2918:509-553 2963:554-628 3038:629-917 3327:918-1368 3778:1369-1375 3785:1376-1599 5672:1600-2366]

L*CVNV*IVITHEIGSYLMS*SFS*LPRLAKDITVKRHIHLFGPSSAKMDPCAWMCCICL

CLWCVSFGNGIPVNPLKVRNPADDAKADNAIDSDNLRPLENDQNKNNLLYIRKDENLPLD

KLKPVDHIDAVKMEQDGHINKEYHKELFLGNHEEFEKEGEDSTAKLLDIFIRVDSDKDDH

LSEAEMEAWIMQKMQEHFDEALQENDEVFKHLDPDENGVVHWKEYYTHFLLAKGYDDTRA

KKHVQDYDEIELDSDAKEELVRYKFRWTDADIDPADNQLNKTEFLGFRHPEQSDKTISTM

VFSIMNSMDSNEDGTLSLSEFIALPPGDVEGDDFREMDVHWQEERKAEFKDAIDQDKDEK

VTSKELKAYLDPRNPVQALMESRNLISLMDEDKDKMVSKDEMLKHKDIFISSKIVDFAAN

VHDEF*KH*ATNGIFVDI*NSYEYVPLKLDCLACF*IHRFSGNITFEVHGQLGRKAT*NL

*LRAKLVDSEQLSRKGSEVLEAFFQEFLSLLIMFQICGRLRMSQCYKHTHTYTLHIYIY*

LVRVAGIF*VGYVRGALL*LANIYDLTFVED*QYCL**QTYEDYDGCQCHNVFGKSNCPV

HHIVLFDGQW**LYAINI*PKSRYFL*KTKLLIFKYKFNLCLCFSQM*SLFVSFHIIPPQ

E*YVQIHLELLTR*SEENVYITYVPVGVTLVLPRFECVGFNFDWCDEEF*FILIQILAVN

CCREGQ*VCLVGSWVSAASLSFISLYVLYPVLKTPKIPS*TAGLVTIINYQVGQVTLCRH

RNALSLGKX

>**comp105290_c0_seq2_6** len=2532 path=[1:0-47 8877:48-178 196:179-199 9012:200-202 9051:203-260 9056:261-265 9061:266-286 299:287-319 5190:320-332 345:333-358 5444:359-371 384:372-412 425:413-456 469:457-457 470:458-481 494:482-636 649:637-693 9257:694-711 724:712-731 744:732-765 9295:766-805 9327:806-905 918:906-1014 9478:1015-1043 1056:1044-1063 1076:1064-1064 1077:1065-1079 9540:1080-1089 9550:1090-1298 1311:1299-1313 1326:1314-1741 1754:1742-1757 1770:1758-2031 2044:2032-2037 2050:2038-2081 2094:2082-2194 2207:2195-2216 2229:2217-2311 9840:2312-2324 6821:2325-2531]

GEQKDPASTPVPPLVSNQPLRPDQILPLDGRFNQDKSKLLERQKRDYVPDDLQKLRETKP

HIGGDGHDYQKHGRGNLGGDGHDLCHGGHFKDFMIKHGKVYNTEDEEAKRFAIFCDNMKI

ARKLNETELGTAVYGATKFADLTQEEFKQKYLSKTTWNKMTNHGASVMNKAKIPSGTAPK

AFDWRDKGAVTPVKNQGQCGSCWAFSTTGNIEGQWKIKKGQLISLSEQELVDCDKLDEGC

NGGLPSNAYMSIMKLGGLETESEYKYEAEDEKCAFNRSEVEVKINGAVNISSNEDDMASW

LAQNGPISIGINANAMQFYFGGVSHPWEIFCNPKSLDHGVLIVGYGVEGSEPYWIVKNSW

GPDWGEKGYYLVYRGGGVCGLNTMCTSAVVN*RCLTSSCRSAIFAPQHTELNGKHVGNTV

IWKI*FSLKRAHI*I*MLKADLNFKKQLPCNILFVEV*NLYILYCNIL*HT*ASHGDVIC

CDMFGGKSYFLHPLETYTGK*TMLNLSKLRHLSLCLAI*LTLCAMTMVSKNFISLILIFY

FTQTIE*YLRRNIIFTAKCTIIYIAHIYSEIVHVSIFCKFALVYILIPTFRLK*HFGHYL

VLKKIYIFLILTPS*FLVFFFLRTIVEHLNPVLIRKFMFSLHPFFFAHFSGSSLTNIIFN

QESLTYL*LKRSNTHLYVLQFFVLQFV*IS*DQTSTLKCILVFITRQLF*YVLLVLLMDL

HCCDVLDIYH***CCDNSCFNH*KHVY*HYFQTPQLLILIKVLTFVFVMQIFGKNQFWW*

CMG*ELVVMLKPD*RMDLIRQTTCVLK*RINFVRMYFYVSV*SVRDFFTK*GN*DWSFF*

IKHS

>**comp105740_c0_seq2_6** len=2567 path=[2926:0-41 10721:42-56 6336:57-163 6443:164-172 10788:173-189 10805:190-195 10811:196-215 3142:216-329 10883:330-334 10888:335-337 10891:338-417 3344:418-423 3350:424-447 10961:448-452 10966:453-540 6664:541-543 3470:544-638 11063:639-640 3567:641-641 3568:642-711 3638:712-715 3642:716-739 11118:740-747 6091:748-784 3711:785-846 11181:847-865 11200:866-871 11206:872-914 11229:915-995 3922:996-1023 3950:1024-1055 3982:1056-1057 3984:1058-1140 8180:1141-1163 4471:1164-1234 8840:1235-1253 4561:1254-1573 11827:1574-1581 11835:1582-1584 11838:1585-1586 11840:1587-1588 11842:1589-1612 6957:1613-1634 4942:1635-1677 4985:1678-1702 5010:1703-1763 5071:1764-1789 5097:1790-2566]

AKIMMVAIQIVVTLTFLAVITADKVPVLMWSQSRPLHDLPQAFAGNSIDGQTFHQKYLSP

LTSKQGHSVVAFVQDKLHMEDFSKYADVYNPFSDGGAFKNIKNLMDDNFSVNLPAVHSPK

DVIDSLMKEFKGRVHSVSSPEHVSGLKMEKDKSYLILVLLPATGQKNEEKAISKNDEKVG

EIVKALNKRSIDYTALFTGERAERDTKATFKGRHLLENPDNVTGIFANVSGEMYFYSRGI

QVVIVQRGADDKNTRTVIDFPKESGTYRFNESSWANNTATFVLSTDEATSNTTGANQFQV

MFKFTAFKNVDRWTAVNFTLSVTNTSGNPEVIVDEQDMSLKDTEMAMPPIFSYHCSDLTL

YPINMTNDKEYALLKFDGLQFQPFGVRNDQFSEAWDCVGFFTTPILMGLVPVGIFTVILF

MGMYMISQLSTMDRFDDPKGKTITVNVNE*RGLDKAVCNGLQFVLKNREFCSRNVFLKSQ

ETSSLRNNLQFLFVYAENLVWIFFCYICKLIVLQCISWLCCVWNVSKLSMWSSNY*LILK

EYG*KVKTRILSYE**KKATDTEII*SQTVSLQPALWGINVVT*LTHFTSSFDSSITVM*

LMVLFFMFYEGYSVG*VSVRFSKKLSSITSPFIDCCLEPIHE*LNLSETAESYFPSIRIN

CGHDVVMC*IPIH*PGKATFVTADP*SCEL*NNFHRLTTYDGYHF**LMNH*CDPVKVIN

VLSMAMERKKHL*DCYRCHHIKN*KNRLTF*H*TPCK*QFVSIAQSLDQPVPRNPLICLL

L*SNQVQKSNA*EVL*IVLCT*CIH*IKKNII**KRTN*FVLCN*NWVVN*NVSFHLIVS

TSNFTFHVLTIAPNKX

>**comp105824_c0_seq15_5** len=4023 path=[9028:0-125 19692:126-140 9154:141-429 9449:430-461 18756:462-463 9481:464-481 9501:482-554 9578:555-1165 10189:1166-1478 10502:1479-1492 10516:1493-2595 11619:2596-3071 12096:3072-3733 18526:3734-3755 12780:3756-3979 18107:3980-3985 13010:3986-4010 22586:4011-4022]

VPGVRKKRRRRPLMEEQHRNIAPAT*APCRVCLHSPQTRRHQPNLMEIVP*MLVQIKICD

HLTRGWLTWALGTVKMTLSMKTTSRIQFIAPVPRVHGSSQSPSL*MRMEVAALQRRLRKK

TWMTSP*RERIS*RVTRVSLMT*PQTGVYHRPMEASTMQKKCIYRSMFVHKLYFVT*ILE

KRIVIYVDDQNML*YQQLAKL*NIQMNLSSYSESGSTTKRLKYCIKLYNSQITSFL*VIH

NHCETSSRFIYLINSSISMFWEK*LMSSCQILS*HS*YIT*PG*VQRNLIVRCN**LLLP

VVNLRRWCESPHRN*GASM*ESH*M*QILYKSHLRVLVNSR*SVSNLAMDVMFAHKTKTI

SFPVLI*SLTYKYQVIVLL*HGCSSHQRYFNLLFESPLGITVGLSQIHPDYGFRFINCQM

*AFHQIGKCL*FTSLTYRIWLVDKLAV**PVILQCALM*VILVPQDLKLYSDTFFPIRDL

R**SCKAYWCNYRSITPQCIYVPHAWKKFLI*EWLNFVYPSLYDIVLCIVYPKLLLGHVQ

*RWLRCSHLIHAINICCVFYVTERRY*ITLMFIYNLLALIAINHIGLNCNLQNTKATICL

FSNIVTGKQTGHFAMCSIFQALTQPVEANYCKL*LRLFFLIHATKNFS*SCSYSRPC*QH

*RDVLPDKIPFSVIHCQLPSQVLLKYTSGFLVIM*IS*DRTFWN*FKTH*YVILSRLSCM

KITQVALFASTLCKGS*LYVQSGYNMCPIQPTLSLNHGAKWPMKQVGFEIITGFSCIGLQ

QETGVGGSERRIVRTLPV*N*MC*LKQLQSLYM*YYVSDCNDHIV*IMKSMYC*CMIPCY

D*DDVTTWSLLELVEIMTFILYQPTRLY*NSVHTTLHQYDFTGKMQRI*VHICIFEKIVD

DLCLKLW*SCHHTKM*FLFLPMTADIIWSVFVLN*KSTQLAEEDLVYWYGTEELVKSITI

PSFMCECLFKNQSLLVYQRYSLFVAMFRCVCVCVVCVFC*E*KAFL*RRLALRYVIIC*I

KVSSVPLCGSPSVQDYYSQNVHTRPRHKADILKFN*LP*I*ENS*IDM**YLCQGQVS*M

CASSMQ*RTSGGDASARCSQTSNKKSYSSYK*DKNFTRISSVIFYAWYTGNCKSKSSVTT

GKV*TGNYYRVMYFVCCCTQFLPQQGLDYGVSLLLREFFLSLSRNRGLFLAEC*IVAIQC

QCVS*FII*ILASNWCG**CTVMNWNIVPLLFMSALHKCTDGKN*AK*HKNIR*AFAAVN

IKPFITQLDLDFSRG*RMSY*LKP*SFLSISALS*RCALLKIQEVECIFSR*IFSRNNWL

VSVLEVMFFDAAL*GTVHNLX

>**comp106461_c0_seq3_2** len=3255 path=[22586:0-70 158:71-836 27518:837-838 27520:839-851 939:852-855 943:856-956 1044:957-963 1051:964-1375 1463:1376-1395 1483:1396-1448 1536:1449-1467 27822:1468-1674 27928:1675-1679 1767:1680-1680 1768:1681-1692 27945:1693-1703 1791:1704-1727 27975:1728-1758 22240:1759-1767 28011:1768-1769 1857:1770-1770 22897:1771-1830 1918:1831-1905 1993:1906-1907 1995:1908-1960 28117:1961-1975 28133:1976-2039 2127:2040-2063 2151:2064-2067 2155:2068-2091 2179:2092-2255 2343:2256-2256 2344:2257-2280 2368:2281-2381 2469:2382-2431 2519:2432-2556 2644:2557-2580 2668:2581-2625 2713:2626-2661 2749:2662-2703 2791:2704-2853 2941:2854-2886 2974:2887-2949 22681:2950-2951 26961:2952-2973 21341:2974-2993 21361:2994-3020 21388:3021-3036 21404:3037-3060 21428:3061-3204 21572:3205-3254]

VICGDLAPLRRWII*QVSG*YAVKKSDEMQLLAICLLCVTGASAVIKLGETNVCERPETY

TTIEVQKSDVPVRVCTHTWCFAFPPRCEVCHTEISVKYKRVSVQKIRNVPVCCKGYKEKD

NSCVPVCQQGCDNGRCIAINICHCDSGWRGTSCSSNCAVGSWGENCNLVCDCSGQGTCDP

RTGICTCAPGWVGEHCQTKCAQGTYGRNCQMHCACHNGATCDHVNGACHCPPGFTGNLCH

LSCPPGTHGDDCQQECDCQNGASCSNIDGSCQCTAGWTGTKCNIPCGHLKYGPDCAGHCD

CYNNATCDSVSGACKCGDGYVGERCDSQCVPGLYGANCSSVCNCLHGAGCSPFTGACICP

PGYTGERCERRACSSNRFGQSCQGVCTCEPANTRECHASTGTCDCKPGWTGSNCTNPCPL

LYYGERCSQRCACANNAGCDRVTGVCQCPSGFKGSRCDTPCEEGKWGYNCLLQCSCINAK

TCLAHSGQCICTRGWTGIICQDRCPINKFGENCSQTCTCQNGAECNPSNGTCICSRGYHG

DKCELECQSGTYGFGCRQSCSCHLVNTASCDPITGLCDCKDGWRGVECDTQCPENKWGPG

CTQACKCINGGTCKANGDCDCPAGYTGTTCSKICPRGTYGPNCVSRCSCTVTRPSCDPIT

GQCPCEPGYRGYRCEEVCPVGTYGEKCRGRCQPCGFGTCNHVDGACDCYPGYNGSSCENK

CSAGLYGDNCAFRCNCLNGAECRSTDGRCDCKAGFTGRDCGQACTDGYYGRNCQLSCTCN

ARQICDRFTGCTAGPQGAPLNNNNNKDPNNNGNGQGKVKAPVPSTSTPTDNAGMNPLVLA

IIGVAVALVIVVILLVLFFTRRMRKLKRDTMTAIYDNGPTVNSGPAIGIDNPMYADTFSP

ASSMQPNIPPNIQPQPRLPTRINNLGAGASVSNDLAVKSVNLNGPTTPKLPVAEDYDSDT

DSIDRIKLSNAEVAAGTEQRL*EELFCEEFSCETLKRLSLSLSIISCSLQSLTTARIEN*

K*LFTVESRHT*SLSQINHPYIALMLK*TSRNHSSHIIIVTG*FYWGQWFQGNFDP*RSW

LELVX

>**comp106524_c3_seq3_4** len=701 path=[6384:0-646 7031:647-669 19316:670-676 19194:677-682 13035:683-700]

GT*RRCLD*PRMSLPSLVAVAVVTACLLYTSFPVSAQTTCGVGSACASCTGAMVKTLTFA

SGNSVQVCCPNCRSRFFNFGSTTSGAYCNCNFDRVIGSARRGEECSAPGMTTYFHLNTMP

ICCPNPLVAARVVPNAANGSFIFNCEQRGDCWRGSQCSACNQSFVGNINGVNVCCSNCDE

YGLILGPGSSCSCNRP*RHHTRTITLRSGFHLYLILYALLYFIFKM**SVSFN

>**comp106543_c0_seq12_5** len=1411 path=[3449:0-30 12683:31-58 3508:59-337 3787:338-349 17373:350-528 3978:529-529 3979:530-553 4003:554-889 4339:890-913 4363:914-1410]

PWHCTRGRRTRKQLRETHVDISIWKHQL*TVLKTDYRIMGSAFIILGTSVLAVLLAGYWA

QRYLPPPKPKIVGIDLGTTYSCIGMYHAVSGAVDILQVQSGKKCIPSVVAFSEEGVVVGF

KAMAQAEHNPQNTLYDAKRFIGKVYRKEELQQAQKQYPFKLEADELGMVHYVVEVNGTEK

KITPQDVGSIILDTLRIAAEANLSAPVTKAILSVPAEFDAMQRNYTIKAAALAGLEVFRV

INEPTAAALAYGLHKKSDLQTVLVVDLGGGTLDVSLLNVQGGMFLTQAMAGNNRLGGQDF

NQRLLLFLKDAIEKTYGQKLTDREDLQVLRTQVEQAKLDLTYKQSVNIVITLHSLGNKVF

KEAIPRTLFEKLNDDLFNKVLEPIDRVLQAVHLTREDVDEIVLVGGSTRIPKVRELIRDY

FGKQPNTAIDPELAVATGVSIQAGIIGGMWPLTVSAVELPTRVKKIQIN*X

>**comp106607_c0_seq12_6** len=7128 path=[23573:0-41 1326:42-264 1549:265-279 1564:280-318 1603:319-361 19907:362-373 1658:374-447 1732:448-467 1752:468-667 1952:668-673 1958:674-781 2066:782-850 2135:851-934 2219:935-1004 2289:1005-1076 2361:1077-1111 2396:1112-1163 2448:1164-1216 19510:1217-1221 2506:1222-1852 3137:1853-1871 3156:1872-2531 3816:2532-2650 19161:2651-2670 25386:2671-2819 4104:2820-2953 19341:2954-2975 4260:2976-3415 4700:3416-3441 4726:3442-3705 4990:3706-3731 5016:3732-4710 5995:4711-4948 6233:4949-5864 7149:5865-7036 26107:7037-7038 22803:7039-7127]

WRHNHAVTFIASTTAPLRIVHVQVRGDICEIYMRRRHRSGGRIMLRGKPGLMVLALLLLV

GTLVAYYSWNRGSSVTSNARFEKYRQVSGFKQRNMGKKYKPQDGPQMQDPPNFENRMFKG

KNKFGGVHQMPPLEPKQFGSQEELGRGLNDDYKFREDADDRDGRDFYKDKDVKEDPRKDF

KIKEDVKEDPRSNFQDFSEKKTGGILYPQLYVKPDAVMTERFRKMEHIVHIDLKGAPPKI

DYLIRLFPLMSKLGATGLLLEYEDMFPYNNELQILAANNAYKRDDIRRLLEAAKVNKLKV

IPLVQTFGHMEFVLKYPKYQHLRESTDTPQVITPVMLGSYELINKIIDQVMSLHPDSPYV

HIGCDEVYELGKGLSGEYMKQHNFTKYQLFLQHVKKVAEFVPTKFHKTKVIMWDDELRHM

PDRLIEQSGLGSKVELMVWSYTPHPSQRFPKDMWAKYSKHFPGVWAASSFKGATGSAQFF

TNVSYHLENHINWIQLVKQVKDILPLEKFRGFAMTGWQRYDHFAALCELLPSSIPSMAVN

LMAIQNGGFTADIHNVTSKMLNCSQLIELDFPKKVNNSYIISQDCKFPGSMFYYAVQQLW

GDMDKYHSDAGLQSRIEGWMTNYHVKNQFSNPGQMKVLKKKLGKILDKLKKLEEPVRNSL

KSVFDAETIEEWVQVHLIQRIDDLQDKYNKADMLLQKKVWNKRPPAAATSLKPMMRDLSQ

GYREQQRQQQQQQQPTLQTRGSAFAKYDVATTSYHLITSRIDLHLCDDRPVDTGSDSEKT

WKTEGGSMQVTMYKLSVDVYPFHPAGGERKHWHRYMDNIGSRNPWVQQLFSSFRDEVMQA

RKAFNIQSPTHSQKPSPQHPFPSPQHVSSSPHPSPLPSPAKPMPSPTAPGSRSPQVAGSR

SPQVSPSHRPKKSTRLLESCVVVKLEDFTIYMVSTPDNKRTGHPKFFASDKKLLHLPADM

SVLHLEYTDYFFPEGLESPVPHANLYVLLNPVRLTVDFLTLLWTNYFMLSLSQNLELDNT

TSARKEHVDIKVEAVMPRVIIPNEFRGEGQSERPEYLQLQMSKICATNTRVELKAERSDL

AKILDVYEKEQLLRSEGFPRDDSTTAIPPHFRAHADSRDNQYFDKGVKQKLSEFTSHEFL

TGSVPGHELSTNTLKRDAGCDMWCVLIDQVWLEFLLSLSSKSRPQPFVEAFPLALWICQP

HSLEKSKCAKDSFKDFKSSNPGHKGVNEVKSDEVDRDKRPQRLLLKQYYSCDSDDEGHAK

GDEGETAKDGSICDNDKEKPAVNANNLKEDVQYSDCHVVASVGGKLRIQLNHLQYVLLMR

LNESFSKFQTQINSDLSSLGSKDSMSMCVPLVLPQIEFAAVCPCLINQRCFPDDFSSPVS

PLDKLSHLTMPASKEFANADYPRIEFTESDMNLPSDVPSSPNMMITKSMSDSTIQSHSNG

MLSHSESTSDFERKSSSGNPQPPPLQRTAISALTISGTTNDDEDSNVSSRSYGVDSGISM

DSRRSTGVRYTKAQPQMKKALSSVSSAFSSFTDKLKLKTDDDMLSVSDDLETMSIKTDMS

SDDEFEHLTFEDSEVPAFCHDPPSDTASTTDTYSDMADDTSSVYAESSSTRGKEIISIVL

FKLDTTEVLMTSSDKGMTVAAQVSKLLTCQPGNISAEDFHAKFSSQKGFFQDNTLSPSHP

KTFPYTVQYRADTTTTPNSALLTVRSHDSSLSFKMSSLMSLSEFVEEEKLSEPIPMVVDV

HNLLLILEEDRPSSNPVSPGAMPIDLQIHKLTIRRGSDGVFHLSGGSIESAPLGMQSLQP

IAALKGGNSPSHTSDTSPGKESEEMATPVLHLEQFRQVQMDNMELRRQIQEYQALQKTTD

KHLTQLRAGMEFMEKLTSENETLKQKLSEYDLSKEDESTNRYEMENLQLQQRVAQLEEEI

VNMTTERDSLFATLKLLQDDLMASEKRHRSHTTAS*FCVSCVKDE*MTIDFVTVCVELGL

LCV*LCSC*SYT*SC**DMAILCDVYHEAFHMIQDHLEKGVQWNLEELTWLSLWIFY*DL

RQECV*HCKLSGIFKQNWINHSKL*FLNSLFVLSYEELQNHRYELAQSFAADLRPNISL*

SVLD*PRSSNIYTNVSFWYLCIMIHTLLYKCHVYTS*WLCITRNTILYHIISCVSQYIKY

CIISVATYHNT*HVVLNQASVQVKVLSRICSSELILIADI*TQA*PKQFRI*RSHYFRTI

VSFQVYVSDFLQKISFHPFTCSSNR*VRDC**RHSAICCCLTSVMSVGEESGTG*HNNQI

FCDIFYLNME*MGFDFHVTGKTKDFIMLPHSFITDRATTVCN*YNSILLIITAW*LKCSA

RLNIVFCNELEWRVQGKLVFHL*KKLIESRHFQSEX

>**comp106657_c0_seq1_1** len=729 path=[1793:0-138 1932:139-504 11374:505-512 11382:513-524 2318:525-528 2322:529-549 11412:550-563 2357:564-606 11440:607-618 11452:619-621 11455:622-728]

TFSLSAANMVFRRYVEVGRVAYISFGPHAGKLVAIVDVIDQNRALVDGPCTRVRRQAMPF

KCMQLTDFILKFPHSARQKYVRKAWEKADINTKWAATRWAKKIDARERKAKMTDFDRFKV

MKAKKMRNRIIKTEVKKLQRAAILKASPKKAAVAKAAIAAAAAAAAVMMIWRFDVHIPS*

CIFRTRCDWMQFNVQKG*VDYIT*HIPDS*LMLTDGKKVND*KALFESWVAQNNYVLSGS

NMS

>**comp108991_c0_seq1_4** len=714 path=[692:0-713]

RHCRFSPLAAAMVNPTVFFDITADDEPLGRVSFELFADKVPKTAENFRALSTGEKGFGYK

GSSFHRIIPGFMCQGGDFTRHNGTGGRSIYGEKFEDENFILKHTGPGILSMANAGPNTNG

SQFFICTAKTEWLDGKHVVFGKVKEGMNIVEAMERFGSRNGKTSKKITISDCGQL*FLLT

CGHFTHQTIPSVAQESVPTPSARNVL*SLLSLKFFGFHIFLIPLQV*LDCKVKFMIMN

>**comp109220_c0_seq1_2** len=424 path=[402:0-423]

PRSHA*GLSPAADMRYVASYLLAALGGNSSPSAKDIKKILDSVGIEADDDRLNKVISELN

GKNIEDVIAQGVGKLASVPAGGAVAVSAAPGSAAPAAGSAPAAAEEKKDEKKEESEESDD

DMGFGLFD*IPAPLQIKSFYV

>**comp109271_c0_seq1_3** len=1300 path=[1:0-1299]

SCLHLAAAASRAPVHPFSLRPAQKKKTPKTLIFFLVRYLHWNHQKQLFQ*TVAMREIVHI

QAGQCGNQIGAKFWEVISDEHGIDPTGTYHGDSDLQLDRISVYYNEATGGKYVPRAILVD

LEPGTMDSVRSGPFGQIFRPDNFVFGQSGAGNNWAKGHYTEGAELVDSVLDVVRKEAESC

DCLQGFQLTHSLGGGTGSGMGTLLISKIREEYPDRIMNTFSVVPSPKVSDTVVEPYNATL

SVHQLVENTDETYCIDNEALYDICFRTLKLTTPTYGDLNHLVSATMSGVTTCLRFPGQLN

ADLRKLAVNMVPFPRLHFFMPGFAPLTSRGSQQYRALTVPELTQQVFDAKNMMAACDPRH

GRYLTVAAVFRGRMSMKEVDEQMLNVQNKNSSYFVEWIPNNVKTAVCDIPPRGLKMAVTF

IGNSTAIQELFKR

>**comp1834_c0_seq1_6** len=249 path=[1:0-248]

TIRKSKNILFVINRPDVYKSPASDTYIVFGEAKIEDLSQQAQMAAAEKFKAPENPATCSD

GAPNPLSQPILEESEEEEEIDET

>**comp109751_c0_seq1_6** len=867 path=[845:0-866]

QQPSKMPKGKKAKGKKVAPAPAVVKKQEAKKVVNPLFEKRPKNFGIGQDIQPKRDLTRFV

KWPRYIRLQRQRAILYKRLKVPPAINQFTQALDRQTATQLLKLAHKYRPETKQEKKQRLL

ARAEKKAAGKGDVPTKRPPVLRAGVNTVTTLVENKKAQLVVIAHDVDPIELVVFLPALCR

KMGVPYCIIKGKARLGHLVHRKTCTTVAFTQVNSEDKGALAKLVEAIRTNYNDRYDEIRR

HWGGNVLGPKSVARIAKLEKAKAKELATKLG*MYTKFSVPKYNYKIKKX

>**comp110357_c0_seq1_3** len=483 path=[1:0-482]

AFKMSKRGRGGSSGAKFRISLGLPVGAVINCADNTGAKNLYIISVKGIKGRLNRLPAAGV

GDMVMATVKKGKPELRKKVHPAVVIRQRKSYRRKDGVFLYFEDNAGVIVNNKGEMKGSAI

TGPVAKECADLWPRIASNAGSIA*FSSVFVKYIH*SLCSEX

>**comp111062_c0_seq1_3** len=2207 path=[2185:0-2206]

QSSGGCGCRRSLREAARMLSVRVAAAVARALPRRAGLVSKNALGSSFVGARNLHASNTRL

QKTGTAEMSSILEERILGADTSVDLEETGRVLSIGDGIARVHGLRNVQAEEMVEFSSGLK

GMSLNLEPDNVGVVVFGNDKLIKEGDVVKRTGAIVDVPVGEELLGRVVDALGNAIDGKGP

IGSKTRRRVGLKAPGIIPRISVREPMQTGIKAVDSLVPIGRGQRELIIGDRQTGKTSIAI

DTIINQKRFNDGTDEKKKLYCIYVAIGQKRSTVAQLVKRLTDADAMKYTIVVSATASDAA

PLQYLAPYSGCSMGEYFRDNGKHALIIYDDLSKQAVAYRQMSLLLRRPPGREAYPGDVFY

LHSRLLERAAKMNDSFGGGSLTALPVIETQAGDVSAYIPTNVISITDGQIFLETELFYKG

IRPAINVGLSVSRVGSAAQTRAMKQVAGTMKLELAQYREVAAFAQFGSDLDAATQQLLSR

GVRLTELLKQGQYSPMAIEEQVAVIYAGVRGYLDKLEPSKITKFENAFLSHVISQHQSLL

GNIRSDGKISEQSDAKLKEIVTNFLAGFEP*SPVTVTRYCFGFVIYSGKISTICKGLLLY

S*CTEIT*IKVPYCVLVCVGVGSGVYQDLCGGQRTASLYDSTLLPPGI*CRFLFKARLHV

EFHKLVL*DRAYCVTSAMASLGYSCSSLSAIAGKLLSYPVLAGAVSMDR*FKAVHYEIRK

YFVID*KENSAPEKK

>**comp46879_c0_seq1_4** len=1000 path=[978:0-999]

HGGYSVFAGVGERTREGNELYHEMITSGVISLKDDTSKVSLVYGQMNEPPGARARVALTG

LTVAEYFRDEEGQDVLLFIDNIFRFTQAGSEVSALLGRIPSAVGYQPTLATDMGGMQERI

TTTRKGSITSVQAIYVPADDLTDPAPATTFAHLDATTVLSRGIAELGIYPAVDPLDSTSR

ILDPNVVGNEHYEIARNVQEILQNYKSLQDIIAILGMDELSEDDKLTVTRARKIQKFLSQ

PFQVAEVFTGTPGKYVPLVETIKGFNSVLKGEQDHLPEVAFYMIGNLEEVVQKAEKLAEE

QS*AKAALM*AEKFFFFF*SVVHCWPNLSSFPG

>**comp11407_c0_seq1_4** len=205 path=[1:0-204]

FPFVVLGNKIDLENRQVATKRAQAWCYSKNNIPYFETSAKEAINVEQAFQTIARNALKQE

TEVELYNE

>**comp123733_c0_seq1_5** len=1186 path=[1164:0-1185]

LGSRRARAFQLSIAMAADISQWAGPLCLQEVDEPPQHALRVDYAGVTVDELGKVLTPTQV

MNRPSSISWDGLDPGKLYTLVLTDPDAPSRKDPKFREWHHFLVVNMKGNDISSGTVLSDY

VGSGPPSGTGLHRYVWLVYEQEQPLSCDEPILSNKSGDNRGKFKVETFRKKYNLGAPVAG

TCYQAEWDDYVPKLYEQLSGK*GCCTGAPTALLSCVKHALPPAPLLPSHWEFLAVLG*RF

RVSSLLLPAVTILDSPIPGSV*LSLGSVWGGV*CSDGVILLLI*KKQL*KRKHSARIRSR

GELELVVSVSG*TRKLSRTGHSGVFKGPVPQLRQLKTPILAEP*LSRSGGCVGIKCLKNS

AAVERDPLKPLASQWGRQKSPELPSVLILLVKYK*X

>**comp128817_c0_seq1_3** len=906 path=[1:0-905]

VSPSHEPTVITFPPTDLKPTRQPVPYASILKPTASNAFPDQKPHRQPVPRTDTKSKQQQR

EASVHGNKTYATSGGYEAHFNQRRPSRQPVPSHDGITNAPEAKTQFVQAHNQISKTPQSP

SGPLWKKAPETPWQPGIGGYQPYYLNYNSRPNNHMNWNPVMDLARQYQVKQQRYSPYGNG

NPLSNWVLSGRRASTSYNMYGQMVPTDSYKRNTYDGHLNPFGVVDYNGPCRRLCTMYCSL

GYDVDVKGCPSCSCQHQGSGRQRYFGDAD*LHSEPSL*PRNPSLRVKCRSPPSVHTLDHT

KX

>**comp135009_c0_seq1_4** len=505 path=[1:0-504]

TTVQQRGAAVIKARKLSSAMSAAKAIADHIRDIWFGTPEGEFVSMGVISDGNSYGVPDDL

LYSFPVVIKNKTWKFVEGLPINDFSREKMDLTAKELTEEKETAFEFLSSA*LDTRFDISR

QPKAEESKCRL*A*YQTVIMLHSNCEQQNILNSVCFMICESLSCC*CC

>**comp136401_c0_seq1_5** len=2422 path=[2400:0-2421]

WTLGLLRVCETRTVVAFRGHSLLLDS*DSCLTAERLVLSAGMMKFTVVAAALLLLGAVRA

EEEDKKEDVGTVVGIDLGTTYSCVGVFKNGRVEIIANDQGNRITPSYVAFTPEGERLIGD

AAKNQLTSNPENTVFDAKRLIGRTWNDPSVQQDIKFLPFKVVEKKTKPYIQVDIGGGQTK

TFAPEEISAMVLTKMKETAEAYLGKKVTHAVVTVPAYFNDAQRQATKDAGTIAGLNVMRI

INEPTAAAIAYGLDKREGEKNILVFDLGGGTFDVSLLTIDNGVFEVVATNGDTHLGGEDF

DQRVMEHFIKLYKKKTGKDVRKDNRAVQKLRREVEKAKRALSSQHQARIEIESFFEGEDF

SETLTRAKFEELNMDLFRSTMKPVQKVLEDSDLKKSDIDEIVLVGGSTRIPKIQQLVKEF

FNGKEPSRGINPDEAVAYGAAVQAGVLSGDQDTGDLVLLDVCPLTLGIETVGGVMTKLIP

RNTVVPTKKSQIFSTASDNQPTVTIKVYEGERPLTKDNHLLGTFDLTGIPPAPRGVPQIE

VTFEIDVNGILRVTAEDKGTGNKNKITITNDQNRLTPEEIERMVNDAEKFAEEDKKLKER

IDTRNELESYAYSLKNQIGDKEKLGGKLSSEDKETMEKAVEEKIEWLESHQDADIEDFKA

KKKELEEIVQPIISKLYGSGGPPPTGEEDTSEKDEL*VH*SARAVIL*ILDSGTFVGRKL

RELKSRM*LESSPQSGVENAIAQVAVYCFSLAVAHMSWGQGEEELAILKIEKKVGQGVCS

PWIWSI*QLGHAHVV*ELFSTISDTNKX

>**comp31935_c0_seq1_4** len=586 path=[564:0-303 868:304-585]

FF*TKKFYRMADQLTEEQIAEFKEAFSLFDKDGDGTITTKELGTVMRSLGQNPTEAELQD

MINEVDADGNGTIDFPEFLTMMARKMKDTDSEEEIREAFRVFDKDGNGYISAAELRHVMT

NLGEKLTDEEVDEMIREADIDGDGQVNYEEFVQMMTAK*RHCTECVKFLVQNCLFAFSLF

VTYL*KVPPPLLSKN

>**comp147991_c0_seq1_2** len=2031 path=[2009:0-2030]

APFTEFISPSGSAASMSDKLPYKVADIGLAAWGRKALDIAENEMPGLMRMREMYSASKPL

KGARIAGCLHMTVETAVLIETLVALGAEVRWSSCNIFSTQDHAAAAIAKAGIPVFAWKGE

TDEEYLWCIEQTLHFKDGPLNMILDDGGDLTNLIHTKYPQLLSGIRGISEETTTGVHNLY

KMMSNGILKVPAINVNDSVTKSKFDNLYGCRESLIDGIKRATDVMIAGKVAVVAGYGDVG

KGCAQALRGFGARVIITEIDPINALQAAMEGYEVTTMDEACKEGNIFVTTTGCVDIILGR

HFEQMKDDAIVCNIGHFDVEIDVKWLNENAVEKVNIKPQVDRYWLKNGRRIILLAEGRLV

NLGCAMGHPSFVMSNSFTNQVMAQIELWTHPDKYPVGVHFLPKKLDEAVAEAHLGKLNVK

LTKLTEKQAQYLGMPINGPFKPDHYRY*VLGLSFTFQLPSLFRALSLVPKSKCHQLCSSL

DSASPTQPSPYTLWGWSLCL*PLLYPSYCSRCGGGMERYLVGIHRGHDLRSLGSPEAGCC

**WSQAMHLIIDTLTWSLDLCAWFADPLVSQSRTQERAPPKSRNPWSLKAPESWAFLLLG

SLLNLMILSWYFFWSLFHKG*DRLTKKGQVTD*KGLEKQRGKGLSLLYS*WFLSQAQVTN

RLICFIMFICYLEC*QX

>**comp152168_c0_seq1_1** len=569 path=[1:0-568]

GKGTLTLCPYHSDRQLMSQVAVAGLLTVLVSFLDVRNIILGKSHYVLYGLVAAMQPRMLV

TFDEELRPLPVSVRVGQAVDVVGQAGKPKTITGFQTHTTPVLLAHGERAELATEEFLPVT

PILEGFVILRKNPNYDL*KSGF*RPWLQRNFLFLAGCPACYEGWILFQIFGGIVTSCCFV

TE*DNFVE*X

>**comp157484_c0_seq1_1** len=611 path=[1:0-610]

DSADLLTVPGLLTQRNCTGTTTEISQTFSTWTETDTSHIQSMDAKAVFTCFLLGVISAAA

TDLTIETKVVELVDFHGTIKTPNYPENYHNDYEGIWELNIPSTGGKYTFVLKIEAMDIEG

DPEHCADYIKIDHKKYCGQSPMTIRVPDIEYGVVIDFHTDISITGKGFTATYDFEPQY*G

SMSALHGPRWITIRDIEIV*VYHX

>**comp164267_c0_seq1_5** len=529 path=[507:0-528]

HKLTKEQWEERIQNWHEEHRGMLREDSMMEYLKIAQDLEMYGVNYFEIKNKKGTELWLGV

DALGLNIYEHDDKLTPKIGFPWSEIRNISFNDKKFVIKPIDKKAPDFVFYAPRLRINKRI

LALCMGNHELYMRRRKPDTIEVQQMKAQAREEKHQKQLERAQLENEKKKREIAEKEX

>**comp173367_c0_seq1_4** len=304 path=[1:0-303]

*RPILIRIFTGFVRVTSISVARHL*RLMSGRGVNSLRKTNRFVLCFSSSGFNNLSRVSAG

RQEPGKL*LTASVLIS**LENKYETTS*SLL**CLVSLQFH

>**comp174131_c0_seq1_2** len=1168 path=[1:0-1167]

EFYAPWCGHCKALAPEYAKAAAKLKAEGSEIRLAKVDATEESDLAQQYGVRGYPTIKFFK

NGDTASPKEYTAGREADDIVNWLKKRTGPAATTLSDTAAAESLVDSSEVTVIGFFKDVES

DSAKQFLLAAEAIDDIPFGITSNSGVFSKYQLDKDGVVLFKKFDEGRNNFEGEITKEKLL

DFIKHNQLPLVIEFTEQTAPKIFGGEIKTHILLFLPKSVSDYDGKLSSFKRAAEGFKGKI

LFIFIDSDHTDNQRILEFFGLKKEECPAVRLITLEEEMTKYKPESDELTAEKITEFCHRF

LEGKIKPHLMSQEVPEDWDKQPVKVLVGANFEEVAFDEKKNVFVEFYAPWCGHCKQLAPI

WDKLGETYKDHENIIIAKMDSTANEVEAV

>**comp17459_c0_seq1_4** len=265 path=[1:0-112 114:113-136 138:137-167 169:168-264]

RYWLCAATGPSIKIWDLEGKIIVDELKQEVISTSSKAEPPQCTSLAWSADAQTLFAGYTD

NLVRVWQVTIGTR*KFYDKVLEINWLSE

>**comp182295_c0_seq1_1** len=1052 path=[1030:0-1051]

RRCTDFAVRLLALLPKSAPANMASGVQVADEVCRIFYDMKVRKCSTPEEIKKRKKAVIFC

LSADKKCIVVEEGKEILVGDVGATITDPFKHFVGMLPEKDCRYALYDASFETKESRKEEL

MFFLWAPEQAPLKSKMIYASSKDAIKKKFPGIKHEYQANGPEDLNRTCIAEKLGGSLIVA

FEGSPV*TASQCHELKASILNTILSLYK*SKYIRPGSH*GICLVIF*SKLKIV*CLQTLP

YLNPASKVSLVWN*TLIGQMPVFMR*LVKILLSSGFLITQFTKQYKAV*RELIFANLSSY

FVSFV*KSDCNPCILTKVLYFSLT*TR**DIIFFIMCTTPNIH*QQ*D*AX

>**comp188025_c0_seq1_6** len=266 path=[1:0-265]

QAEAEVASLNRRIQLVEEELDRAQERLATALQKLEEAEKAADESERGMKVIENRALKDEE

KMELQEIQLKEAKHIAEEADRKYEEVARX

>**comp194138_c0_seq1_5** len=479 path=[457:0-478]

LHRMTTLFCINVLSEVCGQDITTKHMLPTVLRMAGDPVANVRFNVAKSLQKIGPILDNST

LQSEVKPILEKLTQDQDVDVKYFAQEALTVLSLA*CWKKKHCRPLVSTLQPPQSYLP*GG

GGGK*LSLPVHGLTPGSSPSTVRFLLCSLGRLLCPPPKGX

>**comp200473_c0_seq1_5** len=556 path=[534:0-555]

SSYYRGAHGIIVVYDVTDQESFNNVKQWLQEIDRYASENVNKLLVGNKCDLTTKKVVDYT

TAKEFADSLGIPFLETSAKNATNVEQSFMTMAAEIKKRMGPGATAGGAEKSNVKIQSTPV

KQSGGGCC*NLPPSFSHSNEFAI*TQVKKQNCLNCTVCSCTTTDSYRFHKVRDCKWSILT

FFFLFX

>**comp205463_c0_seq1_5** len=574 path=[552:0-573]

LGSLVCVLRGADCV*NSRNAMSRGSSAGFDRHITIFSPEGRLYQVEYAFKAINQGGLTSV

AVRGKDCAVIVTQKKVPDKLLDSSTVTHLFKITESIGCVMTGMTADSRSQVQRARYEAAN

WKYKYGYEIPVDMLCKRIADISQVYTQNAEMRPLGCCMILIGIDEEQGPQVYKCDPAGYY

CGFKATAAGVKX

>**comp217972_c0_seq1_2** len=380 path=[358:0-379]

SWMLEGYNHDAHIREELTTLITYNIELLAVKPYTWKCISKVELFLRLWKTFQLNGYLPEY

VKPPPLFKTTTTAEFTKIVLDQYRSRPNTPDLWRKQRKRFMIEAYKTLSAQALHALRMKY

VRDFILX

>**comp22005_c0_seq1_2** len=594 path=[1:0-570 1207:571-593]

RPAVFCCLSGARTFPRSSQQPAGLCSTVTMCDRKAVIKNADMSEEMQQDSVECATQALEK

YNIEKDIAAHIKKEFDKKYNPTWHCIVGRNFGSYVTHETKHFIYFYLGQVAILLFKSG*K

HGLCQTPSDPSKNKDCIQIPNTRD*IFSLAKGTPRLNLLCCVQGFILCTSLWL*N**NSL

HLYLFSSPYFCTPIFFSL

>**comp224418_c0_seq1_4** len=319 path=[297:0-318]

NTQLSKTEFLSFMNTELAAFTKNQKDPGVLDRMMKKLDLNCDGQLDFQEFLNLIGGLAIA

CHDSFIQTSQKRI*SS*FPSNHQVITSPNPIHTCTEPSTPTTTCIP

>**comp229571_c0_seq1_1** len=895 path=[1:0-894]

AAFNRVAFGNPA*PLPPPSVLAMFLTRSEYDRGVNTFSPEGRLFQVEYAIEAIKLGSTAI

GIQTSEGVCLAVEKRITSPLMEPSSIEKIVEIDAHIGCAMSGLIADAKTLIDKARVETQN

HWFTYNETMTVESVTQAVSNLALQFGEEDADPGAMSRPFGVALLFGGVDEKGPQLFHMDP

SGTFVQCDARAIGSASEGAQSSLQEVYHKSMTLKEAIKSSLIILKQVMEEKLNATNIELA

TVQPGQNFHMFTKEELEEVIKDI*EGAVLELLWDTFSSNCP*TLFPALMSWKISSICVX

>**comp23692_c0_seq1_3** len=1292 path=[1:0-1291]

TMAPTRKFFVGGNWKMNGRKKCLGELICTLNAANVPAGTEVVCAPPTAYIDFARQKLDPK

IAVAAQNCYKVTNGAFTGEISPGMIKDLGATWVVLGHSERRHVFGESDELIGQKVSHALA

EGLGVIACIGEKLDEREAGITEKVVFEQTKVIADNVKDWSKVVLAYEPVWAIGTGKTATP

QQAQEVHEKLRGWLKSNVNDGVAQSTRIIYGGSVTGATCKELASQPDVDGFLVGGASLKP

EFVDIINAKQ*ALPIPLPACIAWTRLPRSLVTAPTGHMLLMTSSAPSCGLIHAVPS*AVS

TALLWLGPGQSLYHLQWVESTVTKVAPSEGRE*N*LSLWALTSEITFHWFGLETHGWSTG

SGLRVTLACHLDGLGSSFTPKFYWMSDAEIQSFRV*IRYT*DPKRPGIVPSPTVPKPLYC

VCEPSYM*GK

>**comp24068_c0_seq1_6** len=656 path=[1:0-655]

PRRLVVVLFSLLLSLIADTSRPTGFRTMANLERTFIAIKPDGVQRGLVGEIIKRFEQKGF

RLVAMKFLRASEEHLKQHYIDLKDRPFFPGLVKYMNSGPVVAMVWEGLNVVKTGRVMLGE

TNPADSKPGTIRGDFCIQVGRNIIHGSDSVESAEKEIHLWFKPEELIDYKSCAHDWVYE*

T*RNQNPFQHY*WVSGQSSSSH*QDGSSFLKQ*RLWN*X

>**comp24075_c0_seq1_1** len=701 path=[1:0-605 607:606-676 678:677-700]

ALPLSSQRRAMGISRDNWHKRRKTGGKRKPYHKKRKYELGRPAANTKIGPRRIHTVRVRG

GNKKYRALRLDVGNFSWGSECCTRKTRIIDVVYNASNNELVRTKTLVKNCIVLIDSTPYR

QWYESHYALPLGRKKGAKLTPEEEEILNKKRSKKIQKKYDERKKNAKISSLLEEQFQQGK

LLACIASRPGQCGRADGYVLEGKELEFYLRKIKARKGK*ASCVV**RCLLFYLX

>**comp24358_c0_seq1_2** len=238 path=[1:0-237]

RATELGQPAPS*LTAVRSC*GTSRSGSRASAMAFKDTGKTPVEPEVAIHRIRITLTSRNV

KSLEKVCADLIRGAKEKNL

>**comp24358_c1_seq1_6** len=270 path=[461:0-243 705:244-269]

RGAKEKNLKVKGPVRMPTKTLRITTRKTPCGEGSKTWDRFQMRIHKRLIDLHSPSEIVKQ

ITSISIEPGVEVEVTIADA*DN*INRLNGX

>**comp24678_c0_seq1_1** len=274 path=[1:0-16 18:17-273]

PLDDTNMQIFALSAILLLLGASSVEAARIEKRVTCDILAQLNIGGNSIGHAACAAHCLWK

GYRGGYCNSQNVCVCRR*TQTCTCTLKRNKTX

>**comp24760_c0_seq1_4** len=902 path=[1:0-696 698:697-901]

VPSAAMARGPKKHLKRVAAPKHWMLDKLTGVFAPRPSTGPHKLRECLPLIIFLRNRLKYA

LTGDEVKKICMQRFIKIDGKVRTDITYPAGFMDVISIDKTGENFRLIYDTKGRFAVHRIT

PEEAKYKLCKVRKIFVGTKGIPHLVTHDARTIRYPDPLIKVNDTIQIDLETGKITDFIKF

DTGNLCMVTGGANLGRIGVITNRERHPGSFDVVHVKDANGNSFATRLSNIFVIGKGNKPW

ISLPRGKGIRLTIAEERDKRLAAKQSSG*NGL*ETCWKSVCTSLSRQHTC*IKQHGETKK

>**comp24999_c0_seq1_4** len=1380 path=[1358:0-698 2057:699-1379]

AAGTLYTYPENWRAFKALIAAQYSGAQVRVLSAPPHFHFGQTNRTPEFLRKFPAGKVPAF

EGDDGFCVFESNAIAYYVSNEELRGSTPEAAAQVVQWVSFADSDIVPPASTWVFPTLGIM

HHNKQATENAKEEVKRILGLLDTHLKTRTFLVGERVTLADITVVCTLLWLYKQVLEPSFR

QAFPNTNRWFLTCINQPQFRAILGEVKLCEKMAQFDAKKFAESQPKKDTPRKEKGSREEK

QKPQAERKEEKKAAAPAPEEEMDECEQALAAEPKAKDPFAHLPKSTFVLDEFKRKYSNED

TLSVALPYFWEHFDKDGWSLWYAEYRFPEELTQTFMSCNLITGMFQRLDKLRKNAFASVI

LFGTNNSSSISGVWVFRGQELAFPLSPDWQVDYESYTWRKLDPGSEETQTLVREYFSWEG

TFQHVGKAVNQGKIFK*TSLASRLAACTYPSREMGVIKEN

>**comp25402_c0_seq1_1** len=889 path=[1:0-435 437:436-888]

FTSA*NDVLSEIK*CNTNKQFSRWVRVCSLSCLETSVGGNTPPLSLTCPRAPAALYKPTQ

YPPFPYSDNSERANRAPSRITMLTLIVVAVVFVGGVVGGCPSRDGMVEEGHTTTFGCLTC

TCGDDDMYTCRYPACNHTETVNPCLQYSSGCCAKCLEYGCYNQYFRGHTSLVPLGRVPHP

TDPCVTCSCHLGHGEVICRSVMCERPKCANFRRRPGSCCEYDCPEEVNSDNTAP*SSRAT

TPMVHVLVSLRDSIINFLLTSVFGGSFGFVG*KKLHYIFC*LLMSFSNL**RFCLKX

>**comp25767_c0_seq1_2** len=1013 path=[991:0-491 1483:492-1012]

RSVCSGLGTHIYY*KIQKKSKKSFEKTQKKFTKKSASPPPETFIFFLPLL*NNPVKQPRP

TRPPAAPQLQAGTQRPRPSCRPDPAPPASLPRFHGR*VRVDSG*ILSLCARPRSPPPGSV

PRPRTRSPPLTGKVAACGPAGSRAEMNPSAPSYPMASLYVGDLHPDVTEAMLYEKFSPAG

PILSIRVCRDMITRRSLGYAYVNFQQPADAERALDTMNFDVIKGKPVRIMWSQRDPSLRK

SGVGNIFIKNLDKSIDNKALYDTFSAFGNILSCKVVCDENGSKGYGFVHFETQEAAERAI

EKMNGMLLNDRKVFVGRFKSRKEREAELGARAKEFTNX

>**comp26256_c0_seq1_5** len=485 path=[1:0-484]

ATHRLYNQASFKAQASDFQRPVKMRLLVVFLLMALIVLPTEGLFRRRRRRRWLRVRGSRV

LAGLSVASRLGLIGKRDVQEDLALADLNEDGNIDQSEAENLFDKRDVEQILQLADMDGDS

VVSHDEFIRGVEELFSLDAE*ILSIQVQKEETTFFTDS*K*X

>**comp26587_c0_seq1_2** len=1722 path=[1700:0-1401 3102:1402-1721]

RPGRGAGCEENLGDPQLLLHPLPVAMAAYKLVLIRHGESAWNLENRFSGWYDADLSPAGH

EEAKRGGQALRDAGYEFDICFTSVQKRAIRTLWTVLDAIDQMWLPVVRTWRLNERHYGGL

TGLNKAETAAKHGEAQVKIWRRSYDVPPPPMEPDHPFYSNISKDRRYADLTEDQLPSCES

LKDTIARALPFWNEEIVPQIKEGKRVLIAAHGNSLRGIVKHLEGLSEEAIMELNLPTGIP

IVYELDKNLKPIKPMQFLGDEETVRKAMEAVAAQGKVKK*RRRGRLLPRHPPCLFLPPAP

SPAPATLTMSLGF*V*VVAADGGLWLLFSL*YFIPCTHSLHRI*SE*RLWGCRFSDHIEG

QLPVPGELRDSNYSFS*VFVY*GSA*EEFREELCCGVTNEKLQSSFVPGTCPALFLQLGN

WGHSHSSGEWK*PHGDF*P*FLSDPKGAAGPRRLGSILSSLCYTYFYKKKYIYILSAHKS

NSEVASDI*NSPVWSPENKPFLISVG*TPCTSCCVWIPWPCFVEVKYPWIMSEFLSFTV*

IMFQLLDPTVWVQSSFGRNN*VSFQISIIKE*CA

>**comp310163_c0_seq1_1** len=336 path=[314:0-335]

GNWEELVKYLQMARKKARESYVETELIFALAKTNRLAELEEFINGPNNAHIQQVGDRCYD

EKMYDAAKLLYNNVSNFGRLASTLVHLGEYQAAVDGARKANSTRTWKEVCFA

>**comp32839_c0_seq1_6** len=370 path=[348:0-369]

PAMTATLRPYLSAVRATLQAALCLENFSSQVVERHNKPEVEVRSSKELLLQPVTISRNEK

EKVLIEGSINSVRVSIAVKQADEIEKILCHKFMRFMMMRAENFFILRRKPVEGYDISFLI

TNX

>**comp33268_c0_seq1_3** len=295 path=[273:0-294]

GCSL*EIEMASKRALVILAKGAEEMETVIPVDVMRRAGIKVTVAGLAGKDPVQCSRDVMI

CPDTSLEDAKTQGPYDVVVLPGGNLGAQNLSESPMVKX

>**comp352483_c0_seq1_2** len=480 path=[458:0-479]

RDLLLDPAWEKQQRKTFTAWCNSHLRKAGTQIENIEEDFRDGLKLMLLLEVISGERLAKP

ERGKMRVHKISNVNKALDFIASKGVKLVSIGAEEIVDGNVKMTLGMIWTIILRFAIQDIS

VEETSAKEGLLLWCQRKTAPYKNVNIQNFHISWKDGLGFX

>**comp36515_c0_seq1_6** len=425 path=[403:0-378 782:379-424]

SLSVVRSSSTSYTMSGRGKTGGKARAKAKSRSSRAGLQFPVGRVHRLLRKGHYAERVGAG

APVYLAAVLEYLTAEILELAGNAARDNKKTRIIPRHLQLAIRNDEELNKLLGRVTIAQGG

VLPNIQAVLLPKKTESQKVKSX

>**comp40524_c0_seq1_2** len=240 path=[1:0-168 170:169-239]

STFSPDGRVFQVEYAMKAVENSSTAIGIRCKDGVVFGVEKLVLSKLYEEGSNKRLFNVDR

HVGMAVAGLLADARSLADIA

>**comp361360_c0_seq1_4** len=226 path=[204:0-225]

RFPRCVKDKSVCELTKDAGTCSEMVHRFYYNKTTNRCEEFHYGGCGGNGNNFKTKRACMR

TCRKRGSFFPRP*HC

>**comp409151_c0_seq1_5** len=227 path=[205:0-226]

IDQVVQTALSETQDPEEVSVTVKAFMTADLPNELIELLEKIVLDNSVFSEHRNLQNLLIL

TAIKADRTRVMEYINX

>**comp41851_c0_seq2_2** len=628 path=[1:0-450 452:451-627]

VQQSSAAKMCDFTEDQTAEFKEAFQLFDRTGDGKILYSQCGDVMRALGQNPTNAEVLKVL

GNPKSDEMNVKVLDFEHFLPMLQTVAKNKDQGTYEDYVEGLRVFDKEGNGTVMGAEIRHV

LVTLGEKMTEEEVEMLVAGHEDSNGCINYEELV

>**comp46381_c0_seq1_6** len=788 path=[766:0-787]

LQPDAGDT*PLAIPLPSRRWSRDHMGI*MNWQ*NRDFGRQVGNNRGRL*GRLTLLRDIPN

TMAGLIRSLEAILLSFCVILVCVSALPSPLRSRRDLGGYDDLQDTGLSQQDMMLLQKLFA

PPTKRQLCRFGFTYNPVYGGCRASLGLLRGRGRSGRF*FPM*QVLRLATSIQSHQSPIKP

CYQLCIVVIQ*RCYIVCTNMYVTSPRTYGHSC*RGSWNQHYRYRTKSSFIIYSGQINQFS

KF*PGKSKP*FWKTAIRGIKRFX

>**comp48021_c0_seq1_2** len=1014 path=[1:0-822 824:823-1013]

GRVHTALFLIPIVT*RETYTMSGALDVLQMKEEDVLKFLAAGTHLGGTNLDFQMEQYIYK

RKSDGIYIINLKRTWEKLLLAARAIVAIENPADVSVISSRNTGQRAVLKFAAATGATPIA

GRFTPGTFTNQIQAAFREPRLLVVTDPRADHQPLTEASYVNLPTIALCNTDSPLRYVDIA

IPCNNKGAHSVGLMWWMLAREVLRMRGTISREHPWEVMPDLYFYRDPEEIEKEEQAAAEK

AVTKEEFQGEWTAPAPEFTAAQPEVADWSEGVQVPSVPIQQFPTEDWSAQPATEDWSAAP

TAQATEWVGATTEWS*AAVQVPEQREKRWKENKVAKSX

>**comp48289_c0_seq1_1** len=667 path=[921:0-507 1864:508-666]

ITLGAHSQYAHFLPGQHSLPTMTSTITLMLVLFLTSVQGNVLLKEAIRNAFYGLARDFAS

GGSKQLTEAGHVQSCSPTDPKFNVTWEPKVLDPEGSIVVNYTYKIPHDFNAGTADVALYF

NKIQDPIFEDSFPVTCDEVKKYIPCPFKGGPTVHGSYPYSNLLILKGYDVCI*HLACLW*

Y*LMTSSVHIKHYGRTQQYLKLYGDCLLV*SLGQ*IQ*LKP*X

>**comp49273_c0_seq1_2** len=963 path=[941:0-578 1520:579-686 1628:687-914 1856:915-962]

RLPATMETTHCMWRLLVLVSVGSLTSGQYFSYDSDTMDTMQDMAEDRQTYMMHQRLYGGT

GTQYNTQGYYAAQQRPAYQYNYQSAGQNAYGTNAYGTNNNGYGRNTNAYVQQTGAYNNRN

AGSQNRGSYPQSQSWNSNNGVNSQPSNSPVSAQNYLQKNQGGSSQQASVNSQGYQQASNS

ATNGRPSQSFNNNQQSQQIQTKCSQVMLDRSNGRPAVNVPVAVISITLIQQTQWSQCTSA

SFGFIGRYAYVGNGCSGNFKVCYQ*EKHHAHWDCELIAHASASHYWQCLRLHRAQDEAGL

YLAQCQTIPITFHMCLLAK*X

>**comp503_c0_seq2_4** len=458 path=[436:0-338 775:339-457]

SRRCGRARRGRVASDMGDREQLLQRARLAEQAERYDDMAAAMKNVTELNEPLSNEERNLL

SVAYKNVVGARRSSWRVISSIEQKTSADGNEKKIEMVRAYREKIEKELEAVCQDVLSLLD

NYLIKNCSETQYESKVFYLKMKGDYYRYLAEV

>**comp50471_c0_seq1_5** len=597 path=[1:0-596]

LFLFSTMAQDQGEKENPMRELRIRKLCLNICVGESGDRLTRAAKVLEQLTGQTPVFSKAR

YTVRSFGIRRNEKIAVHCTVRGAKAEEILEKGLKVREYELRKNNFSDTGNFGFGIQEHID

LGIKYDPSIGIYGLDFYVVLGRPGFSIADKKRRTGCIGAKHRISKEEAMRWFQQKYDGII

LPGK*T*SKKLIKFSQKKX

>**comp51682_c0_seq1_1** len=1203 path=[1:0-1202]

AAAAQ*RVRRQQLGSRRADQVKSVRSRELESKPLKMADDLDFETGDAGASATFPMQCSAL

RKNGFVVLKGRPCKIVEMSTSKTGKHGHAKVHLVGIDIFTGKKYEDICPSTHNMDVPNIK

RNDFQLIGIQDGYLSLLQDSGEVREDLRLPEGDLGKEIEQKYDCGEEILITVLSAMTEEA

AVAIKAMAK*PASRVAVVAAVIHEPTEAPPPARFCSGWALGWTPIQIYLTFYFGFPHPLK

LSGRPCPSPSSLGQE*GSHGLGETPCLFSRSPDGGKGVGYCLWFRFPSPFFLFNSIWNQK

AGFW*MVLCPLSYSSPIWSPVLHSPSPPSTTVQTGDQPLPCLCLFPNPCRGGEKRRGGGD

TIPPQASGKALPPRALPFPVGSLPDTFVKNQT*IKLQV*YE

>**comp51969_c0_seq1_2** len=816 path=[794:0-371 1166:372-667 1462:668-815]

GRGGSRCGAWLSELSGAILVAAATLASTAAMTEQMTLRGTLKGHNGWVTQIATTPQFPDM

ILSASRDKTIIMWKLTRDETNYGIPQRALRGHSHFVSDVVISSDGQFALSGSWDGTLRLW

DLTTGTTTRRFVGHTKDVLSVAFSSDNRQIVSGSRDKTIKLWNTLGVCKYTVQDESHSEW

VSCVRFSPNSSNPIIVSCGWDKLVKVWNLANCKLKTNHIGHTGYLNTVTVSPDGSLCASG

GKDGQAMLWDLNEGKHLYTLDGGDIINALCFX

>**comp528934_c0_seq1_2** len=212 path=[190:0-211]

*KHGYIGEFEIIDDHRSSKIVVELIGRVNKAGVISPRYDATIRDIENWTNNVLPSR*FGH

VLLTTTYGILX

>**comp53070_c0_seq1_3** len=765 path=[1:0-692 694:693-764]

GLVPRWKDLFCKGDSKVSLA*RTQRPVF*LKI*RESHLAATMPNFSGNWKIIRSENFEEM

LKALGVNMMMRKIAVAAASKPAVEIKQENDTFYIKTSTTVRTTEINFKIGEEFEEQTVDG

RPCKSLVKWESGNKMVCEQRLLKGEGPKTSWSRELTNDGELILTMTADDVVCTRVYVRE*

VPTGPRTA*DDFRARYRTQTSLPRPSYKLALPLLLRVTASSKAFCSLPSLSQRGAEAQNP

PTAICPSQVSSPSSX

>**comp54249_c0_seq1_2** len=2907 path=[2885:0-27 2913:28-2564 5450:2565-2569 5455:2570-2906]

GPLPFS*PFPSGRHSRRPGEARAMASGADSKGDDLSTAILKQKNRPNRLIVDEAINEDNS

VVSLSQPKMDELQLFRGDTVLLKGKKRREAVCIVLSDDTCSDEKIRMNRVVRNNLRVRLG

DVISIQPCPDVKYGKRIHVLPIDDTVEGITGNLFEVYLKPYFLEAYRPIRKGDIFLVRGG

MRAVEFKVVETDPSPYCIVAPDTVIHCEGEPIKREDEEESLNEVGYDDIGGCRKQLAQIK

EMVELPLRHPALFKAIGVKPPRGILLYGPPGTGKTLIARAVANETGAFFFLINGPEIMSK

LAGESESNLRKAFEEAEKNAPAIIFIDELDAIAPKREKTHGEVERRIVSQLLTLMDGLKQ

RAHVIVMAATNRPNSIDPALRRFGRFDREVDIGIPDATGRLEILQIHTKNMKLADDVDLE

QVANETHGHVGADLAALCSEAALQAIRKKMDLIDLEDETIDAEVMNSLAVTMDDFRWALS

QSNPSALRETVVEVPQVTWEDIGGLEDVKRELQELVQYPVEHPDKFLKFGMTPSKGVLFY

GPPGCGKTLLAKAIANECQANFISIKGPELLTMWFGESEANVREIFDKARQAAPCVLFFD

ELDSIAKARGGNIGDGGGAADRVINQILTEMDGMSTKKNVFIIGATNRPDIIDPAILRPG

RLDQLIYIPLPDEKSRVAILKANLRKSPVAKDVDLEFLAKMTNGFSGADLTEICQRACKL

AIRESIESEIRRERERQTNPSAMEVEEDDPVPEIRRDHFEEAMRFARRSVSDNDIRKYEM

FAQTLQQSRGFGSFRFPSGNQGGAGPSQGSGGGTGGSVYTEDNDDDLYG*VMCQHAASWP

GWTLFPGGGGACPRGTRGVPMACSIPQSEQFSPSQTLDRGFLLQKKKLQKR*NKSDFHLG

GEE*ITSKELGLGPTRFLS*FGVVQVTCVV*TKALPPPSPQ*SIYTQCCPSPLLP*PTWV

DG*GASVCX

>**comp54951_c0_seq1_3** len=757 path=[1:0-121 1001:122-535 533:536-561 559:562-589 587:590-756]

LPHAIVRLDLAGRDLTDYMMKILTERGYSFTTTAEREIVRDIKEKLTYVALDFDQEMKTA

AESSALEKSYELPDGNVIVIGNERFRCPEVLFQPSLIGKEASGVHDCTFQTIMKCDVDIR

RDLYANVVMSGGTTMFTGIGERMTKELTALAPSTMKIKVVAPPERKYSVWIGGSILASLS

TFQQMWISKAEYDESGPSIVHRKCF*ARGQSPLVV*VPELRQF*LISSKYKVIVSRLKCF

RGICI*LKMKSR

>**comp59460_c1_seq1_5** len=527 path=[1:0-432 434:433-526]

AAEVFGQYRFHLSTNMGNIFANLFKGLFGKKEMRILMVGLDAAGKTTILYKLKLGEIVTT

IPTIGFNVETVEYKNISFTVWDVGGQDKIRPLWRHYFQNTQGLIFVVDSNDRERVNEARE

ELMRMLAEDELRDAVLLVFANKQDLPNAMNAAEITDKLGLHSLRHRNWYIQATCAX

>**comp61638_c0_seq1_3** len=239 path=[217:0-238]

ALFNGLTLLILALISLFSIPVIYERHQAQIDHYLGLANKSVKDAMAKIQAKIPGLKRKAE

*KGPKQ*TFIFKGDTPLVT

>**comp62063_c0_seq1_1** len=3033 path=[1:0-911 913:912-1644 1646:1645-2400 2402:2401-3032]

RRHRRRASLFTSDSENPSPSATMVNFTVDQIRAIMDKKANIRNMSVIAHVDHGKSTLTDS

LVCKAGIIASARAGETRFTDTRKDEQERCITIKSTAISLFYELSENDLNFIKQSKDGSGF

LINLIDSPGHVDFSSEVTAALRVTDGALVVVDCVSGVCVQTETVLRQAIAERIKPVLMMN

KMDRALLELQLEPEELYQTFQRIVENVNVIISTYGEGESGPMGNIMIDPVLGTVGFGSGL

HGWAFTLKQFAEMYVAKFAAKGEGQLSAAERAKKVEDMMKKLWGDRYFDPANGKFSKSAN

SPDGKKLPRTFCQLILDPIFKVFDAIMNFRKEETAKLIEKLDIKLDSEDKDKEGKPLLKA

VMRRWLPAGDALLQMITIHLPSPVTAQKYRCELLYEGPPDDEAAMGIKSCDPKGPLMMYI

SKMVPTSDKGRFYAFGRVFSGVVSTGLKVRIMGPNYTPGKKEDLYLKPIQRTILMMGRYV

EPIEDVPCGNIVGLVGVDQFLVKTGTITTFEHAHNMRVMKFSVSPVVRVAVEAKNPADLP

KLVEGLKRLAKSDPMVQCIIEESGEHIIAGAGELHLEICLKDLEEDHACIPIKKSDPVVS

YRETVSEESNVLCLSKSPNKHNRLYMKARPFPDGLAEDIDKGEVSARQELKARARYLAEK

YEWDVAEARKIWCFGPDGTGPNILTDITKGVQYLNEIKDSVVAGFQWATKEGALCEENMR

GVRFDVHDVTLHADAIHRGGGQIIPTARRCLYASVLTAQPRLMEPIYLVEIQCPEQVVGG

IYGVLNRKRGHVFEESQVAGTPMFVVKAYLPVNESFGFTADLRSNTGGQAFPQCVFDHWQ

ILPGDPFDNSSRPSQVVAETRKRKGLKEGIPALDNFLDKL*AA*YCHMLHSAHPSEDTLR

LSHSAPLEAAGATLTSLSTHLLPILFISELQDSGNLSAGWTGRLWGGRDTALNIFRGKRA

DVQKSK*MHSEVFGVHGQVEFPQRGRWGKCLQEGRQPALDLQPGCGNESLE

>**comp62064_c0_seq1_4** len=1568 path=[1:0-1348 1350:1349-1567]

PSSGHAASPRQSPPHRALAVSKDSKSKMATLKDQLIVNLLKEEQAPQNKITVVGVGAVGM

ACAISILMKDLADELALVDVMEDKLKGEMMDLQHGSLFLKTPKIVSSKDYCVTANSKLVI

ITAGARQQEGESRLNLVQRNVNIFKFIIPNIVKYSPHCKLLIVSNPVDILTYVAWKISGF

PKNRVIGSGCNLDSARFRYLMGERLGVHALSCHGWVLGEHGDSSVPVWSGVNVAGVSLKS

LNPELGTDADKEQWKEVHKQVVDSAYEVIKLKGYTSWAIGLSVADLAESIMKNLRRVHPI

STMIKGLYGINEDVFLSVPCILGQNGISDVVKVTLTPEEEARLKKSADTLWGIQKELQF*

SLPRVLALHCPGCSRASRQTTPFSSELWLVQWC*DGVGKHLTPHSSALLPSGTCVVVTWL

V*QSHCL*DTLPTAGFDYPCEPAALLPCTKHA*ADEFPVKSYNLAPVCTSMMHILCINVV

QDILYIICVCSVHCNIM*DVRSAYG*WNQPPKCHAK*NLEQ*

>**comp62359_c0_seq1_4** len=1019 path=[2118:0-31 1085:32-354 1408:355-851 1905:852-1018]

TFTDVCRCYSYMNFQFGQTLSYQKENYFQTMKTIEYFTV*ERPNIILLYS*RKKMILHLW

KSFSLAASQ*LCLHYLFISRQMRYKSPVNTSLVEK*LTFHFHKNILETILIYVLKSKRLF

FLCVPYIATQGRLHAIS*AGCQGSIARARPREDIDRQFDTQINVIRLCLKQESTGLNAYQ

HFVTITKSIVTQWL*SQQSLPPFLSKKFTTLVENRL*FKRGFSR*ARLCVQAT*NPHAWT

SDAI*CRVRKQGVLFFKSPNVVVNS*VWSLVTDTWAQTINLRLWLL*KKENTLRVDFRM*

PGVMYSG*TCHIVVW*HESTICLYHWLY*ILTCCIIGCT

>**comp63837_c0_seq1_5** len=400 path=[455:0-338 871:339-399]

RRK*CSVASATGVFLLPLS*GQRSGCSYRGRSFDNGAIFLGSNRCTTFQCDYGRVKKVGI

RCPYKSRCYAVGANIRAGSNGCMLYRCQRNEQFMAVESDPYKCAGAFNRCKICDE*RITQ

ICQWQRTVS**MSX

>**comp64150_c0_seq2_1** len=699 path=[1:0-281 283:282-443 445:444-478 480:479-698]

RSKG*NNTLESHTLEYS*FNLRTDHK*LISTEHNMDQMCIDVSDDGDI*PGFSHTQNNLR

LIREAPPSAIMKLIVCTFLVILLAGQSHQIPSTEVCEMKQDPGLCLAYMPRFYFNPDSKK

CESFIYGGCRGNANNFNSIAECEKKCLPGSVCHLKPETGNCKALFHRFHYNAATKKCERF

IYGGCGGNDNNFLNIADCEKTCSGF*KMLTVSH*PRTVKSIKSCLYCIKKIYT

>**comp64210_c0_seq1_2** len=597 path=[575:0-596]

RHQLLAVRMALVICVAVVLFSSVSGHDWLTRRDTYEEPGFDCFETPCEIIYEPVFRIMAR

PTFSNLIEDGRLTLSCSRETLTLANECQHTSSGCMPYVEMEDLFIAFDTFCRHKTEILDD

EACWTHGLMPLAVVSCHDENSIAFANCVQARVTNITECSSGRTGAILRQLVLDVRA*TTP

CLHADSEETEPILHVMFFG

>**comp64368_c0_seq1_4** len=1757 path=[1:0-287 289:288-1653 1655:1654-1756]

SQDSRSRDNGPDGMEPEGVIESNWNEIVDSFDDMNLSESLLRGIYAYGFEKPSAIQQRAI

LPCIKGYDVIAQAQSGTGKTATFAISILQQIELDLKATQALVLAPTRELAQQIQKVVMAL

GDYMGASCHACIGGTNVRAEVQKLQMEAPHIIVGTPGRVFDMLNRRYLSPKYIKMFVLDE

ADEMLSRGFKDQIYDIFQKLNSNTQVVLLSATMPSDVLEVTKKFMRDPIRILVKKEELTL

EGIRQFYINVEREEWKLDTLCDLYETLTITQAVIFINTRRKVDWLTEKMHARDFTVSAMH

GDMDQKERDVIMREFRSGSSRVLITTDLLARGIDVQQVSLVINYDLPTNRENYIHRIGRG

GRFGRKGVAINMVTEEDKRTLRDIETFYNTSIEEMPLNVADLI*GAVLRPGPSPGFSPGV

GLRKSWRGEGREPRDGHLVFVLAFFFFCFSFFSL*INVTF*GKKKEP*TF*TPFSLG*AL

PQAPSPSPPKH*CISLT*SPRS*RLSYPSQISKSESRG*KTRLASFAGPNLQGEPLSEVV

QGIVPW*GEQGRENGSHFYIVLYSIY*FRKQTQNSE*NYLETAKK

>**comp64599_c0_seq1_4** len=1531 path=[1509:0-1368 2878:1369-1530]

PDQGQSLSLAATAAAAAAQRLPSRVRVPPPTPDTEYPVMDKNELVQKAKLAEQAERYDDM

ATCMKAVTEQGAELSNEERNLLSVAYKNVVGGRRSAWRVISSIEQKTDTSDKKLQLIKDY

REKVESELRSICTTVLELLDKYLIANATNPESKVFYLKMKGDYFRYLAEVACGDDRKQTI

ENSQGAYQEAFDISKKEMQPTHPIRLGLALNFSVFYYEILNNPELACTLAKTAFDEAIAE

LDTLNEDSYKDSTLIMQLLRDNLTLWTSDSAGEECDAAEGAEN*RHPGHQPPSPQENLFT

SPFLIPLGFPLARKPIRVYGINCLYSLFTLQLWENSIPWFVLSPPSWCAVTAVEKYQ*LH

FIETRVTSRHLCRGVTAHLVFK*SEPVLQVTVFCITAKIWKM*CITI*SNCNNNVLISTF

LF*TFLEVSFQKQLFHALTTNSFLVGIQKC*IEWESA*CIRAWGHRSKCLLCHIFWRLHL

SVTTGSFLMHSSFAAV*VDNFLPNKNSLTP

>**comp65289_c0_seq1_1** len=1940 path=[10131:0-119 1962:120-152 1995:153-1939]

AQPRLAVRVGLGPGPPPCSVLLLGFPVKMPEEVHHGEEEVETFAFQAEIAQLMSLIINTF

YSNKEIFLRELISNASDALDKIRYESLTDPSKLDSGKELKIDIIPNPQERTLTLVDTGIG

MTKADLINNLGTIAKSGTKAFMEALQAGADISMIGQFGVGFYSAYLVAEKVVVITKHNDD

EQYAWESSAGGSFTVRADHGEPIGRGTKVILHLKEDQTEYLEERRVKEVVKKHSQFIGYP

ITLYLEKEREKEISDDEAEEEKGEKEEEDKEDEEKPKIEDVGSDEEDDSGKDKKKKTKKI

KEKYIDQEELNKTKPIWTRNPDDITQEEYGEFYKSLTNDWEDHLAVKHFSVEGQLEFRAL

LFIPRRAPFDLFENKKKKNNIKLYVRRVFIMDSCDELIPEYLNFIRGVVDSEDLPLNISR

EMLQQSKILKVIRKNIVKKCLELFSELAEDKENYKKFYEAFSKNLKLGIHEDSTNRRRLS

ELLRYHTSQSGDEMTSLSEYVSRMKETQKSIYYITGESKEQVANSAFVERVRKRGFEVVY

MTEPIDEYCVQQLKEFDGKSLVSVTKEGLELPEDEEEKKKMEESKAKFENLCKLMKEILD

KKVEKVTISNRLVSSPCCIVTSTYGWTANMERIMKAQALRDNSTMGX

>**comp65289_c0_seq2_1** len=2735 path=[1880:0-81 1962:82-114 6403:115-966 7256:967-2734]

VTSRACSFSHDA*GNPDPRPTNGGGGGRDLCLSGRNCPVNVLDHQYLLLEQRDLSEGAHL

QFIGRSG*NPLREPDGPQ*TGLGEGAAHQSHSQQTGPNPDHCGYRDWNDQGRLDQ*PWHH

CQVGHQSLHGGFAGWCRYLYDWPVWCWFLLCLFGC*ESDCHHEA*RR*AVCLGVLSWGIL

HSED*HR*TNGSWNKGYLASERRPNRVFGGKENKGDREEAFSVHWLSHYSLCGEGTR*GS

Q***G*RKGRERGRERKRRKGV***T*NRRCWL**RRGGEEGW*QEEKEEDKGKVH*SRR

TQQNKADLDEKS**HH**GIWRVYKSLTNDWEEHLAVKHFSVEGQLEFRALLFVPRRAPF

DLFENRKKKNNIKLYVRRVFIMDNCEELIPEYLNFIRGVVDSEDLPLNISREMLQQSKIL

KVIRKNLVKKCLELFTELAEDKENYKKFYEQFSKNIKLGIHEDSQNRKKLSELLRYYTSA

SGDEMVSLKDYCTRMKENQKHIYFITGETKDQVANSAFVERLRKHGLEVIYMIEPIDEYC

VQQLKEFEGKTLVSVTKEGLELPEDEEEKKKQEEKKTKFENLCKIMKDILEKKVEKVVVS

NRLVTSPCCIVTSTYGWTANMERIMKAQALRDNSTMGYMAAKKHLEINPDHSIIETLRQK

AEADKNDKSVKDLVILLYETALLSSGFSLEDPQTHANRIYRMIKLGLGIDEDDPTVDDTS

AAVTEEMPPLEGDDDTSRMEEVD*VTRTMCLLTFIPSDNIFSMIFVYFC*HLKHLCGMKT

KGKIKFLHVIL*YYRFNSRG**SVCYKT*RNLLFMFALV*SV*LLSWIP*ETKLRWLKLH

LKIFISCS*FCMY*S*K*TQVKTT*QEFPKWLVFQSPENNPKFPRSCNSARLVNGNNSAQ

SHSALKDKYRGX

>**comp65289_c1_seq1_1** len=588 path=[9001:0-587]

MKAQALRDNSTMGYMMAKKHLEINPDHPIVETLRQKAEADKNDKAVKDLVVLLFETALLS

SGFSLEDPQTHSNRIYRMIKLGLGIDEDEVTAEEPSAAVPDEIPPLEGDEDASRMEEVD*

SLLEEALPSV*YPRGSPSSPDPPGSLLMSTRIFYPVLCLKAGRSPPTE*QGWVLCIVVFL

FVLFCSKIKSMQNKED

>**comp65739_c0_seq2_1** len=918 path=[3789:0-389 2034:390-415 2060:416-530 2175:531-554 3449:555-917]

EGTMMLGAEGGEGFVVKVRGLPWSCSADEVQRFFSDCKIQNGAQGIRFIYTREGRPSGEA

FVELESEDEVKLALKKDRETMGHRYVEVFKSNNVEMDWVLKHTGPNSPDTANDGFVRLRG

LPFGCSKEEIVQFFSGLEIVPNGITLPVDFQGRSTGEAFVQFASQEIAEKALKKHKERIG

HRYIEVFKSSQEEVRSYSDPPLKFMSVQRPGPYDRPGTARRYIGIVKQAGLDRMRSGAYS

AGYGGYEEYSGLSDGYGFTTDLFGRDLSYCLSGMYDHRYGDSEFTVQSTTGHCVHMRGLP

YKATEN

>**comp68174_c0_seq1_5** len=483 path=[1:0-315 317:316-328 330:329-482]

KKYTFKMADQLTEEQIAEFKEAFSLFDKDGDGTITTKELGTVMRSLGQNPTEAELADMIN

EVDADGNGTIDFPEFLTMMARKMKDTDSEEEILEAFKVFDKDGNGFISAAELRHIMTNLG

EKLSDEEVDEMIREADIDGDGQINYEEFVKMMMSK*ML*SX

>**comp70368_c0_seq1_3** len=272 path=[893:0-86 1273:87-97 742:98-112 757:113-271]

KIKSFLLLAVICLVGVTEGIPLHTSCSVNWTFSLSCTDVQDKLVGQIGRWTGPDGCADGG

EKCLYKLVSKTKTQVKATRETPKQRYVDDL

>**comp71437_c1_seq1_4** len=827 path=[1:0-126 128:127-463 465:464-471 473:472-826]

LE*HRL*TPRGNP*RTAVMPREDRATWKSNYFLKIIQLLDDYPKCFIVGADNVGSKQMQQ

IRMSLRGKAVVLMGKNTMMRKAIRGHLENNPALEKLLPHIRGNVGFVFTKEDLTEIRDML

LANKVPAAARAGAIAPCEVTVPAQNTGLGPEKTSFFQALGITTKISRGTIEILSDVQLIK

TGDKVGASEATLLNMLNISPFSFGLIIQQVFDNGSIYNPEVLDITEQALHSRFLEGVRNV

ASVCLQIGYPTVASVPHSIINGYKRVLALSVETEY

>**comp71699_c0_seq1_1** len=621 path=[599:0-272 872:273-274 874:275-620]

VTI*Q*HDPYYPYFICKP**WFYSIIDEHRFYAINDG*SDHCYHTRGNCR*RKSSVKSRS

GTMVAVILCVLSLVMTLTPTLGGAATIKGTIVVGQVTCSYAEIKNNSGAGPRGLRLFSCK

NKGGNMNLDCYYYPDNPHDCSYYNKNQENYYNLLAKSAMNNGQPCRVGSISEKKCGKTYP

CQKGNCK*ALI*F*TSSYISNKCFQSV

>**comp75869_c0_seq1_3** len=371 path=[504:0-97 602:98-288 793:289-308 2476:309-370]

ENSLFSSRRI*TMESVLSLALLCACALVCSGAVYNRYASYGQYPDVPYRDQYVPNNYPSH

HYPTDVWGGLSQGHRRYRRSVPFYGNRYPSYKQTYPSNVYPSYQPSYPSTHGQTYPDHVN

TYP

>**comp75869_c0_seq2_3** len=550 path=[504:0-97 1288:98-195 1386:196-202 1393:203-235 1426:236-262 1453:263-332 2476:333-394 875:395-423 904:424-447 928:448-497 978:498-501 982:502-549]

ENSLFSSRRI*TMESVLSLALLCACALVCSGALYNRYPSYGQYPDVHYRDQYAPNNRPGS

YYPDRYPTDVWGGRSQGHNRRYRRSVPSHVNGYPSYRQTYPSNDYPTYPSHQPSYPSTHG

QTYPDHVNTYPGHQDTYQQGQQWNQWGSWSGTQKYGSYQG*RDGRHYWLDDLQT*NPHYM

ICX

>**comp76747_c0_seq2_2** len=1072 path=[417:0-718 1136:719-1035 329:1036-1063 357:1064-1068 326:1069-1071]

ST*YLCCDSRITVFILPVGTVS*EI*FCEITMKSWSQVLLWGACLFYMGVADISEATDSP

LPNCPSPRTVCSSVETNPTPDDGRGTHIVFPVCACAGSTSCPTKPNNSTLQMTESLWYGL

CRPVSDVPSCHDDEVASRTVIEAENFDKKNYTEVQCHCTSGILQGEAYVLVDYENFGVDK

GEVGVHARMCDSDVPLPEGEPDATEMRDYKRGLDHGLSSFYKRAIHSGMSRFYKRGQNNG

MSSFYKRDRGMSSFYKRARENGMSSFYKRDPMRHVSNFYKRDLDHVSSFYKRARGDGQSS

FYKRSRDDLSGFYKRGINEGMSSFYKRGVDGDTSSFYKRAPGGDVSSFYKRAPGGDL

>**comp78671_c0_seq1_1** len=812 path=[790:0-691 1898:692-698 1905:699-771 1562:772-811]

RGCKMAVQISKKRKFVADGIFKAELNEFLTRELAEDGYSGVEVRVTPTRTEIIILATRTQ

NVLGEKGRRIRELTAVVQKRFGFPEGSVELYAEKVATRGLCAIAQAESLRYKLLGGLAVR

RACYGVLRFIMESGAKGCEVVVSGKLRGQRAKSMKFVDGLMIHSGDPVNYYVDTAVRHVL

LRQGVLGIKVKIMLPWDPSGKIGPKKPLPDHVSIVEPKDEILPTTPISEQKGGKPEPPAM

PQPVPTA*QGLGSCIWRHLIK*RHLIKS*TX

>**comp80541_c0_seq2_5** len=645 path=[623:0-227 851:228-229 853:230-234 858:235-273 897:274-307 931:308-434 1058:435-470 1094:471-644]

SHTPHICLMMNLKMKYVTGLKVLKH*MTI*SMKDWKGILQITRYERLSNLSAKTNLQVQI

TLQLSTIEQCLITHLCDLFNKIIKFRYVPFNYRLGMVIPIYKGKKEKSDPRNYRGITLTS

CLGKLFEKLLLKRIETKLLENNPYFPDILQFGFRKEHGAVMSVFALIESIRYYIERNSTV

YAAFLDNEKAFDRIWHDGLFYKLHQLGIQGNIWKX

>**comp80870_c0_seq3_4** len=434 path=[2168:0-5 1060:6-59 1114:60-83 1138:84-88 1143:89-112 1167:113-171 1226:172-433]

HVNKCVRISTNCDYFGSWKPGQVAVRRLSCLYVGIYSPRVASSPQTLEAETRLSKMAKVS

LIAVVAVAMLMVVPYVHGAGGSCYAPPFVFGSTCRSDTSVLGSRGWKVRYTVTVTGNAGT

LVCCEARYCDTRGCRMRSIGCGHS

>**comp81210_c0_seq1_1** len=907 path=[885:0-224 1110:225-266 1152:267-293 1179:294-700 1586:701-906]

V**DRNVSYAEIADRSGQKMSMNWDLLLCCIATLCFYSANAICDMTEDKIQVSEFDVIPG

GQKLGHEKDWDGINFSCNFTYQAQGGTKETWLMGLSMNADNTKFSCSVERPGSSSYLFFQ

SFRLMVGGVQAVGAEVKGPDQSLLNPEEYVMDESSSSVSQVDGKFKSMLGRVELCAVKAK

QEL*RVRRIIANTTLFFF*LKSIAVKCDSIKDTV*HSDQLCDRAILS*GQQKPGIM*LPY

RNIDVGLLY*GTTGLKNPLVSKFSWV*MKTNYMILNLQLQIITG*FLLSSTNYCLSE*V*

FYX

>**comp81444_c0_seq1_6** len=312 path=[1:0-45 47:46-50 52:51-98 100:99-139 1200:140-144 1205:145-173 602:174-213 215:214-245 247:246-256 258:257-311]

FIKVDVGDQYYHLRIFAPVPYMNSGPSLVAYQRGKTGKSELSYFK*SP*SCQS*YSQCWL

NRSIIRDIVKEKNFKQLLI*NMFH**YLT*TI**NKVLFHIQKX

>**comp81967_c0_seq1_2** len=4284 path=[4329:0-298 4628:299-2756 7086:2757-2762 7092:2763-4276 8606:4277-4277 8607:4278-4283]

*GAKKFSPRRYQVCFVSSPRVSKGNSMRI*EITFSTKMARGTFIIGLLFLFGLCGTAAIS

LSLPHTLLLLSAQQIIVVSQIDPDDASQDFGDYSYSYEDRNNLLDSTDIVSPGQGHVQLD

IEMRNVTKYKGQSVRLRCEITGYPIPKYTWYKNDAPINDNDEKGRFNVRRTAWGNRLRIS

NLETTDTGFYTCKASNSFGQEETTGVLAVRNEPAPEPPSSSGKNPGKPSDVDFKAELPGD

GDEFNGKYNSEFLPGKEEKEEDQGSGFCQVYRGSTCSKFVGNMSIYVTSKLTQSRKEEKL

IAGFAVIGSSSHLSSKCQEYAIRSLCFHSLPLCDTNTNNPRPRQICKDECEILEESICKT

EYILAKQHQLIGNSLLPDCKKLAGPGTPEGDNCIRVGISITEGYNKKHNCYNGTGTTYEG

NVSKTKSGFTCQKWNSDTPHQHFFKSSRYPIVGSHNYCRNPGNQEEAPWCFTTNGRVQKE

VCAIPKCVGGASGSAGGEEQGNKLMFILVPGITVPLALALLLALVCFCQQSRSRQNGKGK

TANRPNVPMEMSPLNPKPSSRAREFPMGNIRFLQELGEGAFGKVYKGELLGLYGDNSVSK

VAIKTLKDTATPKTQNDFRREVDLMSEMRHPNIVCLLGVCMKQEPMCMLFEYMTHGDLHE

YLLMHSPHSDISGKDDEGAAKLLEYPDMLFMSLQIAAGMEYLASHHFVHRDLAARNILVG

ENLTVKISDFGLSRDVYSSDYYRVQSKSLLPVRWMPPESIMYGKFTTDSDVWAFGVVLWE

VFSYGLQPYFGYSNQEVIEMIRSRQILGCPEDCPARIYGLMVECWHETPARRPSFREMHA

RLRAWKGEIMTGQNPHWSLSQSHSAHSSSTHQSSHSQPSHHSSTGPSNTTAVTGLTGSSN

TSDPMYHPIAQPPQMVMHHPNLPSHMQPLLPPHNTRNGPPPYNKLENSQTKISPPGSVAS

SNSTKSATSQGSSTGNLKPGNHNAMPLYIGNNAKNGTPSVTECNRFNSLMSHNGYIPDQR

TSEI*GVPVCTEVCADCPIELPESVTA*L**YNDAVT*LYKLFRHWNTTRMGMVLLFLAQ

LMPVDVLRHWCAMLNVTVLSKRCNNIVL*SATGFNQMSSTC*TLTRCYEIC*LLYNAVK*

KTCLPSLPYALESWVQLAVTSMVAIPKINFGPIFVALYLEASVFVLFGHRIQLKLTLSSV

HGEI*E*RCDLLKIKYVYLKIRTF*ESEMISTSPLDKCWSSFK*SYSQLL*YIDQYCC*F

CSYFVLS*LAVVYLFYSSLTCE*QHNTILCRPA*RPKIVALKQS*IANNLHNFLLLDGVI

VAF*SVLMLV*VLGT*ETVCVADGRMC*L*GVSLYLAVTNIIA*RQSDNLSGWVGGNGPF

EEETGDN*NNLSGT*KRKYA*MRMCNSPFCLC*CRTDTFPPACVDIAP

>**comp83133_c0_seq1_3** len=545 path=[1373:0-75 1609:76-124 1141:125-134 77:135-271 1659:272-283 226:284-285 1673:286-298 1687:299-312 950:313-343 286:344-390 1223:391-416 1731:417-429 1745:430-544]

ENPPIIRGTNS*RGEDFHFSPKLVNMELYLILTTVALAAIQGVSGDCQTDAYCSSGQCCV

RTWPFSAGICLIRELSTQYCNSPSKPCTRQADCGFHECCKETSPGSATGVCTPHGLEKSS

CNLTPNTNTPVDAMCACEMGFTCTPNPDAPDSHQGTCASPLTRGCQAHSDCPKNKCCTRF

G

>**comp83799_c2_seq1_1** len=820 path=[3192:0-768 3961:769-819]

GASQNEAIASGKQYFTNVAS*QDEGHLLPLHVLAK*QVTMNLRDVILTFTSALVAFSRAH

QGHHHAETQAVEGFHEAGKNEQHIKEHMQNEMNTQRQMSPQELEFHYFRLHDTNNDTMLD

GLEILQALSHMLPPNNLQPHEVQGKTEEQIETMKQDRHKLMFKNYVEIIDKVLRDDDRDG

NGYLSYPEYVLARRRDEHRMRQQHEEMMKQGAQMQGYPGGHPPQGYQQPPPQGYHQQPPQ

GYQQPPPQGYQHPPPQGYQQPPPQGDQQPPPQGX

>**comp86516_c0_seq2_3** len=1210 path=[1901:0-187 2094:188-331 96:332-424 189:425-439 204:440-591 3781:592-645 410:646-719 484:720-775 540:776-800 565:801-816 581:817-875 640:876-889 654:890-901 666:902-963 728:964-995 760:996-1023 788:1024-1156 921:1157-1209]

SVYSVFSDDVVYTFPHSYEVAMI*FKPEMHFSQ*TRLKSLNPLLTRHITKRAECYA*LSA

LIR*SI*RHRNWAVVTTLRKTWKTMMKFTVLLMLVLAGAVMVEGGAGSSRHTFVVKSDCN

ATYLAIHANYTLCLVDNGADASLSAADKQAIVDKHNDYRSNVSPTATNMVKLVWDDAIAE

YAVKWARQCTLGHDDYAARSLASMPGVSIGQNAAGGYDSVIKAIEGWHKEVEDFEHGVGN

TTVGTTVGHYTQVVGFRAIRIGCGEATCTNAKYSRYQVCNYAIGQQTPDVKTPYEKGTSC

SKCPSGKCSNNLCDCGDKLCLNGGSLDSSTCSCTCTGIWTGDTCTEKKCGTSKNWCKNVP

DKNWCTKYRNIPVDCPKFCGVC*ASLPVDPYRPFTL*R*LTQX

>**comp87152_c0_seq1_4** len=1152 path=[1:0-8 10:9-10 12:11-34 36:35-35 37:36-59 61:60-303 305:304-306 308:307-330 332:331-879 881:880-903 905:904-1151]

*GHQYNLHVMESLAILFFVTIGTVLSLDCHGPAEIVIAVPGSEEAVPNVEFRFLEKFLTQ

LVSYYSIAPQNTRVGLVLYGREPVVIADLDDAISTQTLNTRITLLSHRKLYADNLSGGQN

VPKAIQKVHELLAAGRGSVPKLGIIMTYGGSDLTSPDPQTVIAQVSKASAAALNDGIKLF

ATNTGGGLPGFYNITMDACRLFSLGSYSGLSVLLPYLASATCYILDASVNPSPMSCFPDL

LPLPPQVTFQCHGNTVHQEDPTNCAYYVQCPMAIRMPCAPGSLFDPNITRCNSKEAVTCY

SGLSCPAPKGLFPHPRDNTKFLSCSNSIPYVNECPDNLVFYPLKSECDYPDNSIKG**KH

PFSAKIYKEGHTMLA*KLFDQGYN

>**comp88441_c0_seq3_1** len=1542 path=[1563:0-601 2165:602-827 4392:828-847 3856:848-849 2456:850-877 2484:878-1541]

GSRVATRPATTTIAFESFTRRLRALKRVAIQRKYLIMLVATIILVVALPFYVFADDTPKG

PKVTDKVYFDITIGGEKAGRIVIGLFGKTVPKTVKNFKSLAEGFEDGGKTLTYKGSKFHR

VIKDFMIQGGDFTRGDGTGGKSIYGDRFPDENFKLKHYGAGWLSMANAGKDTNGSQFFLT

VKKTDWLDGRHVVFGKVVEGMEVVRKIENVKTNPGDKPAKDVIIAESGSIAVEKPFAVEK

KAAD*KTALTVWDVQGVRRYFYLGILLLKKPGNSIPYFLLANFLSVSSLYDH*NDCLKAS

YFGYL*ECAYIFVNLREQKLSLYSGIFIVTGSVLEIYFQLTIN*KHICNTNYSLNKTCKS

SVPDVTKEPLVHNRCVKASIIVSISSSRPWCLQHAGSGAPWNPK*H*KKWL*MNILSTIC

NQYCHYTTAFTVFNSS*SVSVIKFGNLLNELF*NLFSHIMCSMFVVSSWMLCTRGILFIC

LRVSQFYFGPLLTGSIYVMCSNDRSFLISGIKVL

>**comp90074_c0_seq4_2** len=784 path=[3104:0-92 234:93-443 2205:444-545 2308:546-783]

LFEEDNS*FSYCIPLLVCLMFMCLKYYVAISDITGYLNAVANKPRLHKRWVPLARAAFSV

GRSLFRSFSRGRVSRSGSRLTRQYNRQGNYGDAVRDFNRLNPNGARPISGNNNLQGITGT

MGRHRVTVRNTSSNGRPTLEIRSPNSNGGSTVRKFRYNE*IYGRVGIY*FFC*FRFESFK

FRKIPYSGETSIFYILLLIATVKETF*LKPSKIHYQFSIHLAL*MKAIVTLDKYYISYVH

VSYEREG*MNG*LCCNEFLHQ

>**comp91178_c0_seq5_5** len=972 path=[3835:0-926 4762:927-952 4788:953-956 4792:957-971]

PGCWIPPPCRSADH*PGCWMPGMPRLCSPHHQMSPLLMTPHSQQIRLVYAYREARAWPPV

QSPYFAFPSLAALLPASEQPSTILLLMRHSPHTAF*RV***CYDGADCHRSGQS*HHSTV

VSRSRHCPDHLIHLVGTALTRLEGADS*ASVCHTALPSCPPAR*PASWQR*CHLGHCRGH

HRGRHEHRPLPGLVSASPVIPQ*AVPTSHEVQGQLFFAPQDHLRTWHFPDPQELSSDPPV

WPSH*RYVPLGGDQTDAECYIPSVAAPHFGVSLDASPTRPSLLLYTLYVNVCHRKEVWSF

VIGSTQTCSCQSTGPASAGTCVGX

>**comp91372_c0_seq1_1** len=2171 path=[2149:0-1207 3357:1208-1276 3426:1277-1856 4006:1857-1938 4088:1939-1945 4095:1946-2170]

QVLVALQSQHATKY*NRRVFPRYLR*H*SFKMKVGYLYQLVALACLWLAVVADGKTEKKE

GKKLQIGVKKRVDPDKCTIKSRKGDTLKMHYTGKLEDGTEFDSSLSRNEPFTFTLGAGQV

IKGWDQGLFGMCEGEKRKLVIPSDMGYGDRGAPPKIPGGATLIFEVDLIKIERKEEL*SS

LINSNGSCIYNVSNGSFYGL*P*IDFILQINVQCFEMITQCLFTMFMD*IS*MNSFN*DT

NIRKTVIFVERSYVLFIESMEFQFIIDL*LNYINVCSEVGISVLIPLMHFISTSSYRCCI

QISS*TSNSQTCDREHLAMKKAKLQCPVL**S*KYTP*L*HG*Q*RALLQGYIWCRVPAY

FTCCHLKY*LYFKKSYLKQHVTSII*LDGKAERYKEFVYS*TIQSVDFLTVCRAIVAAFF

INKVKPFFCTCHERHIELCTFGRVINCCP*PLRSTGVL*SCILLVPYMNLMCE*LSPDCA

NSYATTASVMYKECLLSSFSQYMD*LFCSKCSVIMETNTILLVITGAVKIHSYSVN*TLS

LHEFKFLFVIRCINYSRNLKGVLIFKYGCYLSN*DMHNRIYPVQALISIQFDVVSGHCRV

PLSLLTLSEHIRECILLLLH*I**ILQQLLT*IICHPIFALITLFL*YVSYLLSYWICCS

PSIQLTWKYSA*MHTCNT*MVTNIRTTYTFYLKS*FSTFMLLSKCS*MFVFC*M*KRVKM

SEGX

>**comp91382_c0_seq1_6** len=2116 path=[2094:0-775 2870:776-1120 3215:1121-1135 3230:1136-1214 3309:1215-1457 3552:1458-1473 3568:1474-1511 3606:1512-1903 3998:1904-2115]

TS*LRVKMVLSHSLRTFAAMTSCLFVVFLLQSSLFVLCYQLKDVEVMLEIKGKPAEIFSA

RKIAQYDGSNPSRPLYMGVKGVVFDVSKGKDFYGKGAAYNALVGRDCSKAVAKMSLEEED

LTHDISDLSEEHLKALDSVFEGTYMAKYPVVGYMDFLKEQFPDKFVKAAAKEDL*SLMTT

L*DGHTSNYFLTITWVISRTIKS*TR*TNYLNN*TIT*MFYMISCYD*LPFLTSVKYV*F

HRHQNAFTEITVYCCIKSRVISVKFKL*KSLLRIINKIKRICTYAQLLKCANCMLVILFL

FTKFPDLKTKTFTFKRGNMSTINLCQL*RSY*M*SLVMFSLSVYRKKHGCYFFIVSAETI

*KLVLIIRYLYVLWNFIMNKTYMFFIQTINKQLSQANIY*HNLEMFSFAV*AVVMKTFIY

FLLSMSEGDPFYGKCY*LYNGCDMLHIFRLIFSLLPENNNNLPKVLYE*CDCCYYRLAVT

H*GYIKQRCVYSKKTQLIRLQGLSWSVHEYFIAQTLFMFSSLEIKLNCDIVSTANGILSL

HFPESQDILMTFGLLHLKVPNMNLKTVVLVIHTYPLMW*RFQV*FGHQCPCLS*HTSDTV

WLLL*FC*PQFAQHYCDLKYRLGSGKLSCVFIRDKKGDF*KYRNI*SQA*TISLIL*YHV

TITKIVMDSCFLQC*C*VKINLLKSKLMHFKLHVRNWKVRLFMDX

>**comp91393_c0_seq1_5** len=3152 path=[3130:0-1533 4664:1534-1692 4823:1693-2939 6070:2940-3151]

GSSSRLDLSPCSAFIMAENKLTVKQKFTNFCTFLYNSETGEVLGRSGRNWGEITIFYIIY

YICLAGFFAACYAVFITTLDEHFPRLIHEDSLIRGNPGMGFRPMPDLATTLIRYEMADEK

SYKPYVDHINEYLDKNHRNASSDLVDCPDGVPRPDETTACTVDLNTTLTRCGDDFGFKDG

KPCILLKLNKVFDWFPEPFEADSLPDNMPQSLKDRYDQTRVWVTCEGENPGDNENLGEIE

YLPQQGFDMKYYPYKNQKNYLSPLLFLRLANINPNVGMLIECKAWARNIWHDRTDRQGSI

HFEIIVD*CLDLLNNRHLSIWEYSMGQSHIMKA*SLIYIFIDSVKKGRLTVLFVQM*SIN

EV*HCFKV*WKVSLCKYDFRPKGFVLKSLRSLGLEKLTPVIT*TSIRNTPWMSNMKLLPN

VIKKIIIKSY*TLLKLCVNDVYNFN*LYKILDVAE*ISDALCKNV*IEDC*HYEILSFSS

RSTFFFHHWLLEIYASMLLETGHFVCPLHYSELDSGEKMSLNRDAIL*QSEKSV*L*DRF

SFIIL*IKML*INRYIVYHIIWNVS*LEMECLMKQVVYFIWHSM*FNL*FEIVELIHKAF

HTVFTF*DKPMVLSESVHCANAMLIDKVFLVGEVYK*R*SYNW*GSIIVRGTLCEWLSMQ

DNSRIGWLLDI*RKHLWSSYVFDKE*CLKV*FGSVFGRKICMLVNHLLH*RFDCTPN*FL

KERLIITSTHSAKSVCINVYLNKARILMVEIDHRLFHHLSAHSHIGRDIYIYVCV*YLRT

FYSGRNLDLNIFTKQFW*HITLLSRAT*VFDNTSEEYIPKVLPSLYR*R*C*EMYVP*LE

SVV*WKLNQMNISPLPHLFVMITCKKQIL*AIFRNGLEPLFGQDVCLTITSTMHFYIHIV

GF*YFCLFMAMFTVVVLDSCKCYNEFNRPYRL*VQGYHRMIGLLDLHGISSIICVTFFTY

SEQFACTYFQIFKLHHGSFFLSLCICLF*HQFQIIPSDINT*LS*FKYHTSQGKIFLL*G

VICRLLLVLYTTVEC*PDVCETE*NFVLIKX

>**comp91512_c0_seq4_5** len=1616 path=[2789:0-200 10372:201-209 3035:210-306 3132:307-320 3146:321-440 3266:441-448 3274:449-606 3432:607-1300 11160:1301-1303 424:1304-1312 433:1313-1590 711:1591-1615]

QPPQQEQQQVNQMNQQQVPPPQGGAQPVHGGHGHGHGDTMKFSSDIHNAEHVMEHLENVI

ETKPKDQMTEEELEFHYFKMHDYDNNNKLDGVEIGKALTHYHAEQSSKDSSAPPPEVKVF

TDDEISNIVDVVLKENDMNGDGYIEYAEFKRAQDKEKQKPPPTQPPQQ*SAVGRSSLLQI

LFEFDTVSWKDFVFSG*TSFKCNLYNVDFIILVQKQ*QHLKCSKYF*LRWKDFN*PDLFE

YLVYCLYVVLDCRKIW*ICQ*L*RLYLVEVCTTPNAKLMNIEFERLFDC*RKSILE*C*C

REVHRN**SVMISKLFLINDSSLDTLVEQNIISFIKHTLLQIHWSLLRYIYF*EVVELFQ

*GNI*HIFFCGFGKSSICGNIKFNMLYVAPYHTLCNNGHD*YNICISSTFRTVF*SINCG

*R*LFEMNTWRYQ*L*VILS*TFHHYLVF*AQWNQFCMELRWIVVLWYYCHFCYLSFQCL

IKKIQLVPVLSSAFASMIFAEINFST*LNQLKCFQDYH*YVSVHAQDGLGT*EAQSSKX

>**comp91520_c0_seq1_2** len=1858 path=[1836:0-405 2242:406-408 2245:409-483 2320:484-503 2340:504-533 2370:534-552 2389:553-1375 3212:1376-1409 3246:1410-1766 3603:1767-1857]

ATNAKCHYNNNNKMACRKGSFLFVFSIAAFCGLTNAENGGSAGTEISRLFVTKDSHEVLE

LKQPNGTGCGCVGYTCGCCAHLEVKKIGLNDTVCTNLTYLPDEYGVSLTLSVDGVVYFNR

TISARNPPPICVGLPYLKKEASICVKFYNLSIQQHTFSGCVNVLAKLVSVVVESFKLGCF

KIPPRGSPVLAMSGTRNIQDGQQHRFDVAEEKKLLKQHSGSLGSGCSCLYYDCGCCAHME

LDTIGLNNTVCGNLTFEPKQSIVDISLSVDKKVYINKTVSVRNPPPVCVPLLSMKDVSLC

LQLYKLDIKDAKLTGCLRVVTKVEDRVVENYDVGCFKFPLVTTDAKLPASIPAK*EITTL

RSNNYLDGHTIQLTKTVFRN*CFLHLMTTKLLVGF*FCFLALMSFASIMLLYIKVRKAVP

T*TVMTVYAVLL*QHHIATLRMSLYHVVLMR*CLPLLVLH*VKPLRPHCAHLSIQGVHRD

SST**CKHVFLYIVGDPWGARQLLT*RTTVICFMCRKD*NNLVRIMRHSTSIIRQVYLSF

L*LLCIPFQFWVCICY*QFSRPGHE*LQMC**C*AIFTLSRHSRMYCQCSSNILPHAGKV

LSTKFCFIHFM*YVQV*SQ

>**comp91920_c0_seq3_1** len=597 path=[1:0-45 47:46-58 60:59-105 3114:106-130 3139:131-184 170:185-200 6194:201-255 241:256-280 266:281-547 533:548-571 4603:572-596]

HVGNSQVISLIVAGVSSSIE*PGQFHEE*QVSHWWARG*YYTYKFAVTCRYWRASLALGY

NHREPDLWSWDIAVVLLLTFQRQNAGMWRKYLLLVTLTLCVISDGISGRDSNSGRRRGRL

TQLDYRRDFTIGPNETDFTIGPDETARVSPILLSQMCAYCRQQGNRPCIDRICSRTTVER

GSAVA*WLKCHLVTLKTRV

>**comp92088_c0_seq1_4** len=674 path=[1:0-31 33:32-76 2470:77-121 123:122-145 2497:146-154 2506:155-188 190:189-256 258:257-272 2583:273-294 296:295-328 2621:329-339 341:340-350 352:351-447 2654:448-448 450:449-468 470:469-525 2678:526-531 2684:532-673]

WVTGPR*HSPGYT*RAAGKGPEVQTYCG*RLEV*TFMTSAGFAHVAVPGNVCQRQTTPDW

HILRCPCHIRECVHNQDMCMQLATVAALMSTMRGLIILLAAIVTAESTRYLLGTTCSTSR

DCGTGSCCRDANNVLLDPSLKNDDFAPSTNRGTCKPGSAQLKEVCSSGCQCAPGLICYTP

ITGACCPSDRCETQEYVDQMKKYWNNCAPPMCFFPARK*IPMIK

>**comp92459_c0_seq7_1** len=3536 path=[24690:0-221 24912:222-228 10457:229-730 14471:731-737 10966:738-1008 11237:1009-1009 11238:1010-2109 12338:2110-2977 3338:2978-3503 522:3504-3535]

QRSTSSELLRTIISLQRSDVRR*EPSRVNTSTEIAGIHRTS*RIVPTKHGRSESLQSRKY

GNVVSNTAVKEQ*VEALCTTNGPLGMITGTIKDWQISASSVYPQEWDRGCNEKYARVYLP

NKLGWCAKYKSSSEWLQVDMGVAARVTGVMTQGRGDGLEWVTSFMVSFSMDAFHWNYVTD

NYGNQRIFEGNTDSFSVKHSYLDKPIIARFVKFHTVHWHRHPSMRVEIMGCQLCKESIGL

PPYGKITSSSSRKFRKKSSCQPEDGNIFSKKAWCAKQQNDKQWIQLDVGPPTLVTGVITK

GRGDTKRSHWVERFKVSYSNDTTVWYFYKDADHLDPKLFGGNSDKNTDRTHFLNSPFVSR

FVRFHPMEWHGKISMRVGLLGCPYTGVCSSGFMRINDDTPCVENLAFKKESWINSKRHYK

RHIRNQVAEGHASRAVDGNLDINFQSCTILDNLYGDNPVWTVDLGNKQAVAGVIIYTWQG

RKALEKRIASGDPLFTNHKGERQEGTSFQEYMKNLDKLVVYVDEKLKDEDDSYASGNMCN

YVSSLNNALFEKKLILQCIRRHVGRYVLIEAWGVPNSWSRLFSAVLCEVQVFS*CDVIMT

SS*HQRQW**C*LTFYDEERG*RLFFCDLYGS*CL**VCSNQATGINISVRRLLNGSADL

PPLLQGRRHYFSSHDGL*FSSQVTESRSPPVHHIAMTLQRRF*TSRTVDSLY*VSCWKRT

YFTY*Q*PVPLQTVDGRVRSTLLEKMLHKQKRSATFVILCEAG**CQRTHLRRRLRRVDT

VFNVTYSFDSVEQHH*SLF*NF*NKNHY*KLSEL*QSQHSHKS*LQGMRNVFNYHKYFMI

PFVNDQKLILWLFKKYFYTFWTFPKTTSHPGYVVAPLRPL*RYYETSTGMYVHPKQCHRF

HSNAFHIEKLEGTSFCCNFIALPVILTLYITI*VSLVLGAQKNYHRTGFTKRIIFGTC*K

IKLSRNECNGRSFGEAGII*TPSFFSGTNRMAITERDEW*RLEIDR*NLNFPKVLDVFRR

KISLDVI**CY*MVSEVVNMSKFGNNF*NGLSESSHHSSSGLKILLFK*QMSQFQVSAGK

LLIVKFSRKPVH*FFSRCMVV*APAVQTTPVIYFQCKY*SLK*RSSPHSD*PNIV*IIPL

YSVSV*YLCNSSRDCYCYQGYFHVAWQRVTS*IYDVITX

>**comp92803_c0_seq2_4** len=1122 path=[1166:0-291 2288:292-305 2302:306-324 4799:325-342 1509:343-359 1526:360-412 1579:413-434 1601:435-546 1713:547-564 1731:565-710 4909:711-711 1878:712-734 1901:735-840 2007:841-843 2010:844-1121]

RIPLSGVHAVRLVPE*HEWVTLERSFRVGYGKERVVTDVDHHN*PRSWLPNIGPYIPRHC

VSRKRNMASYVYVGALLVAVAAAEIGLPCMQPNVVAQKTRECFASLGSENLINLDPTKQP

GSLGQISNFLTGSRVCRHEDDLASGINCLFDFYGNCLNIRGMFASYPQIQKASRFACENK

DALADSCLKNVAKETLVCYMKKVQERTIKIITGEVKRKSTEEEMCGFLKEELKCMKTALS

GCPASTGNSYYSFVEMLMPTRCQHLTLYNILKHRPRADLASTHMKSPR*VTTNTSEVQVH

IKIFYI*RKAK*SIIPVQCTLQTFLYVCRTGVFFVIKATIKFIIVIFDLNMFYSLRKRIG

HKLFNLMTPSPNLY

>**comp93111_c0_seq1_5** len=3363 path=[29:0-40 7572:41-49 78:50-639 7684:640-652 681:653-663 692:664-701 730:702-705 734:706-2191 7821:2192-2206 2235:2207-2215 2244:2216-3362]

WLSAKRESVG*S*TFGNGLCAYKCIWLSLRVQTTGFQDPRETSVES*RESAVA*CFGFIF

RRVGRSTVMPKTPAVGIDLGTTYSCVGVFQHGKVEIIANDQGNRTTPSYVAFTDTERLLG

DAAKNQVALNPSNTVFDAKRLIGRKFNDRAVQGDMKHWPFKVVSKGDKPILQVEFKGENK

LFAPEEISSMILTKMKETAEAYLGSKVTDAVITVPAYFNDSQRQATKDAGTITGLNVLRI

INEPTAAALAYGLDKNLKGEKNVLVYDLGGGTFDVSVLTIDEGSMFEVRSTAGDTHLGGE

DFDNRLVNHFKDEFKRKYKKDLTGNHRAIRRLRSACERAKRTLSSSTEAGIEIDSLHEGI

DFYSKITRARFEDLCSDLFRNTMEPVEKALVDAKMDKGKIDDIVLVGGSTRIPKIQKLLQ

DFMNGKDLNKSINPDEAVAYGAAVQAAILSGDTHETIKDVLLVDVAPLSLGIETAGGVMT

KLVERNARIPCKTSKTFTTYSDNQSAVSVQVYEGERAMTKDNNLLGRFDLTGIPPAPRGV

PQIDVVFDIDANGIMNVSAQDKSTGKSSKITITNDKGRLSKEEIDRMVNEADTYREEDEK

QRERVTARNQLEAYVFSARQAVGDAGDKLPQADRDTVNAKCEETLKWLDANMLADKDEYD

YQLKEIQKTCQPVMMKLHGSGQSSGGQGQGQGQGPTVEEVD*TLPCLCISTVSYNIASCV

AVG*LID*HSE*FGT*ST*EGVSLVLDKGL**LVYVYFE*DR*VI*YMK*QYVSLISSET

QQLRPILQFAFVYTGILRQQSIKRLGRVFSL*QNILHTYMLDQREIARCVLHIKMCLCYH

RLPLVYPVFKNTSRRAL*PKRPRVKPHRANENEIAPSRHTLSPLAHLQTRTLSAGYCILT

LYITGS*FMRIHLGIVLNSVHYLKTAYSDLKLVFCFSLLNVINLKEHL*MPVNVNEYIAR

LFGLSISILLLCIMGLMLLSGICVV*ILTIIHFHNFFTDSHLTRNML*KSQIVLLEKLMP

HLFMDASFCPTRVLICAGMHVPTVCVVSVHYLYFCEEIHCRAKERKKCSPEN*VRPKRQL

NVTKIVVVALLWYPDQSTTVIP*SKHRCDTLIKTQQ*YPDX

>**comp93193_c0_seq1_1** len=1303 path=[1480:0-38 1519:39-871 3263:872-887 2368:888-1005 2486:1006-1063 4483:1064-1069 2550:1070-1078 2559:1079-1175 2656:1176-1187 2668:1188-1240 3030:1241-1259 2753:1260-1302]

IYSGLLHRHQNQTES*IHSSPKQTMGAPKVGINGFGRIGRLVLRACIDANVPVVAVNDPF

ISLDYMVYMFKYDSTHGRFKGEVSSDGSKLIVNGSAIDVYCKRDPAEIPWGDSGAEYVVE

STGVFTAKEKASAHFKGGAKKIIISAPSADAPMFVMGVNHTKYTPDLDIVSNASCTTNCL

APLAKVINDNFGIVEGLMTTVHAYTATQKTVDGPSAKDWRGGRGAAQNIIPSSTGAAKAV

GKVIPELQGKLTGMAFRVPVPDVSVVDLTVRLEKGASYEKIKEVVKNASENEMKGILGYT

DEDVVSQDFLGDARSSIFDAKAGIALTDNFVKLVSWYDNEYGYSNRVVQLIQYMSSQ*TQ

KNVNMFK**QPKLFC*ILQNW*LASYSCPTSGSMYMCVLKFIQPKESILQGKK*VPKKKH

QLLV**C*PFI*ISX

>**comp93624_c0_seq2_4** len=1542 path=[76:0-21 1724:22-161 1864:162-198 5133:199-215 1918:216-224 1927:225-264 5160:265-269 5165:270-279 1982:280-299 2002:300-614 2317:615-1113 2816:1114-1382 3085:1383-1467 5318:1468-1485 3188:1486-1541]

QL*SNERLTVIRLCLVQTNTWGILVKQIHGSTFGLSKMNGFIITVLAFVLFGGSSVSALC

DSSSNSKIQQCLFKSELFNPSTTPKDEASIQTFCRDNMDSIVKCMEEHTQECVNDEKEMK

LMNLLVSIPDIRKGLMYMCNNISVLADKATCFSAFGESGFLDCLQNAVTTIANAGEATDI

KSGLDMVCKFFDSFTGCFTDPFKGDCEPVGVIYGHVFQGVKYPYCKLNAVERQAAITDYE

TSAIGRTSIVQYSIVSIIAAVSAAKLL*S*FDSYRRPIHVHSGSFNNSQTVLGLIFHWGL

*NQFTKDFFPLSQHRTFTCIQTLAVIFIIQFMTTILKNMHHKPTVCIVYSDV*FFSVRYH

MLNHLNW*LLFSGYSVHYTHILHATNISDMHFQTVDLYIVYLYVKMAFRIHVLYGN*PGL

TQVTMSAILHFAHRHVDFSVSAR*PHVCFI*GISLLTTCLLYITYQLADHMSALVYQPPN

HISAPV*TAHWPHICFSVNGLLTTCLLECKPLTD

>**comp93938_c0_seq2_4** len=1861 path=[1:0-76 78:77-81 83:82-275 277:276-373 375:374-381 383:382-1309 1311:1310-1623 4932:1624-1627 1629:1628-1643 1645:1644-1860]

AHEAL*ATGSALFTRYNYIR*TIHWFVQSPAYLVVNHAKGGRSRRERNKYGYVTYRIKEV

KFDKFSNSPYLAAMLSTFFKRRRRASSVTFLGLVTFMFLCLWNFSTMDNPSQTYVHRFRL

FGGAAEDNLPSDFYKLPADDPALVNIVHSKFRSPSTVEYNLTEPDKRDFSRGQSEVIDQL

LGSKENGFFVDCGAFDGETKSVTLMFEKLRQWRGLLVEPNRDQYFKIVKKNRNAHSINAC

VRTDIENYGRPEKSEERYGFFEGRQRNDVKAVPCFWLTTLLLATGQKTVDLLSIDLNGKE

MPLLQTIDFDRINIKSISLEIPISYEGRSILPILDYLKLKGFSAMHQFTDHVHKTKDLVL

KKTSP*NRPRSGNAVLVIFQTRIGIVDFSANGHSKHIYSADQFTKEYDRECGKYRVQTLF

GHLSMET*RKEW**RHSAESWRSS*CDIVVQGPSHMKREPGHERNN*NM*SLCGINDYHH

EP*SYDLLQLKPLPTVQGHFWVWNNMLDGSQHFTKIVFILRLFPMTIQGS*SSQSLDVVM

MCYPGVHVHIYTSHITHCMQFAV*GQNIVSAFRSQTYVTA*GVCFIYPYIPAERYKHLIN

QVIELFTSMHQ*L*SVCGEN

>**comp94771_c0_seq4_2** len=2548 path=[4980:0-113 5094:114-132 5113:133-275 5256:276-561 5542:562-666 5647:667-748 12210:749-761 12223:762-769 5750:770-784 12238:785-846 12267:847-856 2891:857-1182 3217:1183-1259 3294:1260-1386 3421:1387-1414 12321:1415-1423 3458:1424-1486 3521:1487-1535 3570:1536-1582 3617:1583-1600 3635:1601-1856 3891:1857-1914 3949:1915-2068 4103:2069-2070 4105:2071-2080 12567:2081-2098 6291:2099-2547]

NMKLWNALLLLVIVWIFPNEALRCPLGFIHHKHSCYWFSSMKGSFGEANAVCRYLKSHLA

EINNRDEDEFVRGHIMRHGKSPYYLLGGSDLRLEGRFTWQGQVTPMVYQNWNPGQPSNYG

GRENCMGIDKTMGYRWNDWPCQDQHNFVCEI*VGLYLRGQMKDTCRKYSLLGFKYCI*QF

INT*SASKICFGAQSRGHAM*FDVALCGYFMLK*LVKMATDLY*ISRIPY**SLI*VFHQ

MIILHQ**F*NVASNCLRRPACDPANLRCQYRALL*PCAKTFESTLCTDSFSVV*LSSNM

HACII*SIKFCSGHMLFK*ICA*VLLNPKNIY*I*RKIKEFMAEN*LNKMFTYLVWHAVG

LYVSGYFCTNECATNVVFK*NTGKPKKQKQTIIHGSL*LRS*WKD*KATFLCIPVGSFMT

FFCQNLFIRYIRVLNSFIYV*DKIGLSTTNVFCTKTCVSKRNA*VIRKTACICT*RI*VA

*YNFPSRTVCKFQVITRIFMHSSA*SVVDLPSLNFDVMYQLLSRAEG*ACILLGNHLDNS

LSNRVTFYSLWFLVWW*LMCW*SIVCI*TGLLCVVISS*RYI*SLILCCITVPCRLCPIH

F*CKYPWTLGW*SSFSC**SIARFYFVLSIVI*FYDSVLVGISVML*YFYCPANLNLLIL

ILFSCIVLVLHVYL*HSSCFTVLCRQVFVLSIYILFQCQVLTHSLTLLY*LVLVTIWLKY

HLCDVKYQLGHSLNKYHCK*RVLEIKYP*ADLKRLT*SLQ*SLQGINILFLVPVFVFAIK

YVNLHCFSCDFFLLQRHGG*EGFGLK*RRNSFWKLNLNYVKEKGL*NRRLYSRTITKPTE

LFF*QDGSV

>**comp94917_c0_seq1_2** len=2269 path=[15704:0-542 16247:543-812 21695:813-1689 17394:1690-1691 17396:1692-2268]

WMVPW*CVSCNSALHHADTVQPQHIFVLAIPPPSQN*LDLVPK*KTLGKDNRQ*RICFND

NFNYTLYKSVIALYQLI*QREMSWFEPT*MTSGCHRQEIMYSKKCTQKIVFLVINGQQAK

SSVFYKSTKCKLELFLTSSQTSCTNFMRTGLF*RN*KFSRKHLSLNWN*CL*SGQFW*FC

RKRYHRDVFAQIRKKCIPVKYC*AHEKCHVKVEMMLRCRDLLSTMQISNLKPNIRPNINI

*HYVHNLFS*DT*K*LMQLSVNIHVANNACRWEQGETLHNRSKRTAICLLLPLKKNINMT

LQQLPKQKHLTWLCVHSG*SLHPALTSIL**YNASNCETYLEYQATLRTHQFLQ*RIIPI

DVMSFCHYN*EK*GYCCVKVLLFQKLFKSSFIRV*YGVKDKRCDTTCCIGHVCHH*Q*DV

*IF*V*MPPLVCTVHIINQYVHFYTLKKTPLACA*QRKSTYTNKTFRHLAIQRTKCSHKT

*I*YSNLQSYLH*YNTTNNRLSNNIYLWQYVAVLFVFHRQ*TSIVVCS*GVHETASCVFE

CNF*HFFCQALETNLLTINGWVGESGKGNL*R*QH*ILLISYVYYKHDGTRTHTKHLQYL

KINVRGKRVKIGVCLGPKLGDG*SFDRGGIGYWRMNMAVNDSKQLF*GRYRPTNAKESQV

MKVIILWSCWGKG*VFLNCKKYLHNHKTDLIFVKVYTDLYEIHCN*NFNFQI*NTEISSN

LIHMNVE*SILIFKNISEMHSSLCNYTFVKGPRTQS

>**comp95009_c0_seq1_4** len=1905 path=[181:0-75 5348:76-151 333:152-174 356:175-318 500:319-326 508:327-438 4253:439-446 628:447-518 700:519-560 742:561-1904]

SVSNSNQL*A*YIQLFLRPYKEDQSLY*S*HNETVPCAKATRCKLR*GGRLGRTFISSLR

VS*IDSIMASGITLDEEALDLITKDTKGHVYKYVLFKINDKKTKICLDQMRLISDLGPDC

KTVEADKEEFKRLGEIVPKDNMRYIMYTYVDQTNNYQRSFIKWLGPDAPVGIRMIYSSSE

SVIRNRLGGKLKKMMLEDEFRYPQEQFQEEDPQKKLSN*G*L*KNKLYFSTDS*VLTNLN

AISV*SSSLVYVHVSSSDKGQPLPGCDARMQAENMLTLYAEQYYHLYSLSAISEILSEPS

PPL*KNWHFL*LCK*SFSYIITECELLSSRRCVNNGFKYIY*KLYHSDDLVIKSLLHVSV

TMFCTENS*FP*ICEWARDVCC**FGLETVPCWNAPYLCV*PTGAHT*TQLFPRSDCPQS

KACVFAIRQVNCFDNMTASM*HSSV*RVVLKQVVV*HRRLLSSYSERCIAGMVNSASTKR

*NSNV*TVSVFHVAHSLTFRAERKDFYS*LSLRGFICRGLIKK*P*LCRLIKSNSISKLI

LSER*QLEYEIHL*KAIFLHIDSKPTY*RVKETIHGFGHVKYMSRFSSIFKNWVGRVDFI

FIF*RSGEMY*SKTWNAGSWDGFLKSAGFIHGNLP

>**comp96038_c1_seq3**_1 len=652 path=[2212:0-24 2237:25-106 2319:107-155 2368:156-188 9644:189-297 2510:298-322 2535:323-344 2557:345-368 2581:369-395 17637:396-404 17646:405-422 2635:423-519 2732:520-651]

IMCKPGLFVIMVALPLATLLPYNTMDDPRITKIKIQTEVVKRLTAEASMYEKEVETLEAR

VKKMKDDGAAEHEVRKQIEVLQESRAMIPDTKRRLIIAYKELEQILQNDSDLSEMEEFGQ

AKEALEAEKEAVA*CCP*NVSHSEVSLCECMWLFV*ADDKTCYELLQSYTSNVSILLRVK

GTVFTYGIWCVSKLIIRAATIRFNSSKIEADTLVSIFX

>**comp96364_c0_seq22_5** len=1773 path=[3581:0-91 21921:92-234 3816:235-556 4138:557-580 4162:581-592 4174:593-604 4186:605-616 4198:617-628 4210:629-1299 3131:1300-1314 3146:1315-1319 3151:1320-1321 3153:1322-1373 3205:1374-1656 3488:1657-1677 3509:1678-1725 3557:1726-1726 3558:1727-1727 3559:1728-1772]

TETARLTRLG*HDTYRKHTYTITYIISVVLY*TALAYLSTFCYLSCELGYVTRKHQTYNL

CNVCVAFLEAHEAEQ*WIATIVFISNVQQLTKLRSPSLTSGTRNKLRPWPKVITGPIV*N

LSTAVKVSATKSV*HLPPSRKSCRLGTSHRMSRLLTTYKMFRFVVVAVLAAVAVAADLNR

EWNTFKSTYNKDYLTKADEANRRMVWENNVKYIQTHNLEADRGLHSYRLGVNEYADLTTK

EFVKQMNGFRMTNTTGGSTFLPHSVGELPAEVDWRTKGYVTEVKNQLDCGSCWAFSATGS

LEGQHFKKTGLLVSLSEQNLVDCSKPEGNHGCGGGLMDFAFEYIKVNQGIDTETSYPYVG

REGRCRFKKANIGATDTGFTNIQKGNEADLQNAVATVGPVSVGIDASRPTFHLYKSGVYD

DPMCSTTSLDHGVLAAGYGTSEDLEYWLVKNSWGESWGDKGYLKMSRNKDNQCGIASQAS

YPLV*RSI*GFPISLQIR*FAQMLYFLSQDVPAPTYWVASGEPHLNDVHMARHPMFSTRE

TFLSE*YI*M**H*FFLKCCLIYFGYQLH*DMSINDSVIRVFLNKI*LKEX

>**comp96832_c0_seq1_3** len=1153 path=[1330:0-329 3831:330-333 3835:334-346 3848:347-360 1691:361-583 3982:584-589 1920:590-1028 2359:1029-1044 2375:1045-1084 2415:1085-1096 3669:1097-1108 146:1109-1129 111:1130-1152]

RFFVDMFVDMWQFSISGRLPRH*QEG*CIDRIASCTMSPSGIHVNKLCIVLIAGVFAGVL

VIYRLWPGDSSKGDTKLTGPVEENKMAGKDESKEASWYTQFVDIPADVKTVGNDQVKEVV

VEVSLNGKSLKIFTRQADPKGSATNVDVLFLHGQRFSSKHWLDIGTLSLVSSWGHRGVAV

DLPGYGNTTDVIDDSSHGAFIGALVKALKMTRPVVVSPSMSGGFSLPFLFDQPETAEDRS

RGYLPVAPVATSKYTEAYSKSKIPTMIVYGENDKTLGTSSLKDLIKLPNSKPFVIPDAGH

PAYIDKPPFFHRLLYNFLKIIAAQK*TLL*GNVRVLLESELTGCYKGQESDTNTGLLHQV

YLHRHTDTSLHALA*MHNHTYTGX

>**comp97002_c0_seq2_2** len=1276 path=[5671:0-97 3276:98-659 3838:660-663 3842:664-785 4660:786-932 6483:933-1125 4340:1126-1149 4364:1150-1275]

S*VAYISMKQCPACQAPRAFYDEILTAKRFSPGYERVFDRMVDRRIEKMPRDDLPEFFRA

CGLHPTEGEINAAFNTIFRGAGNTAEVVFVLDCSASLGPVNFKCQLNFLKDIINDLDVAA

DRIRVGLVPYTESVFESFGLTRFKTKQDIIERIDQIKFKAGLTRTDLALKMMREIFQSSR

PGVQKVCLVITDGKSHDVNLTLREARKAQQDNIEIYTIGVGYDVDLEELKCIASSEKNVH

TVSEYKALANLKDLLQKRICLGCCTLCQHVLPRILSEGFYQSEVMDMLWTIFVPLGTRLT

RNIYELVDPYTDDRILTQTDFRTCLKLVIEARLNREGVIAIPSHVRAGVEEVSNAEEAME

KIEKYLLERKAEKEKLETTAPQVAV*SGGCAYDLRATV*YIHSHPIDTYWEHARARIICL

*V*RR

>**comp97057_c2_seq2_1** len=506 path=[966:0-42 1009:43-48 1015:49-189 7173:190-217 1184:218-266 1233:267-288 1255:289-372 7638:373-375 1342:376-384 1351:385-385 1352:386-441 7682:442-443 1410:444-453 7676:454-463 1430:464-505]

T*TMEPGTVDRVLRMIIHSYFVLCIMSVLSAADDCVKVDSCTCKTSKRTINLHPLSANPG

PRFNTSAKSTDKYFYNPCKDFTKGGVTGVVIQEQGGLDYDNGDDSTAAFTGDPDLDTLVL

TYQHTDGGIIRISKIKLLCKPNTDELVFNEENPKVNYHFTLYTQHMCIG

>**comp97297_c0_seq2_4** len=3335 path=[1:0-69 71:70-93 95:94-461 8007:462-685 687:686-695 697:696-843 8128:844-991 993:992-1009 1011:1010-1085 1087:1086-1271 1273:1272-1281 1283:1282-2153 2155:2154-2852 2854:2853-2877 2879:2878-3334]

NTRRD**RCRDRSLA*MWVSHNQQSRSCHWNCLCD*SQDYIMSWRIVLLSLTSVLGLVAS

QPRYFFNRGMTKMAEQHVVELLVVVDYSAYLKWYHKMSGDTAFLKKKNALNAIWKHYSSV

IHGIDTIYRGLGDTGLKISVQPVDIQVVSTDGIVTSTNKFIASETALRAFYYWTQSGNKL

PPFDHAMLITGYDLMENGDKSKSGRAYLGTMCTTRSVSVVENDFSFEIVQITAHELGHSL

GARHDGEENACNAGDGFLMASVIEINSTNRWYLSPCSVEYIETLLSQLRRDRNLCLLQNK

HEVEKNEGPTTSIHMGELYTPDQQCELIEGRGSYFCRDFYSKPEDYSHICTNIWCHVSNS

AHNICNNYEGSDGYACGNKKVCDKGRCVLSNKGVESLDYCPQGDQPGIVWKNYTCAQLVF

YEPEMCYDDNTRSRCCLSCTEINKGIPGCEFGDKSSWCRTDLIIPMGCYKNENLCCGTCK

MYKDLSRPSCPYGDRSLWCNTTLNPPFECYDNEDLCCGTCANHFNQSRVGCEYGDRSTWC

ETTLHPPYDCYKNGVLCCGTCPQYYNPDNPGCEFGDRSQWCETELDKPYGCYLNSEFCCK

TCDSFLNKTRVGCEYGDKHKDCESLEYPVGCYNNDNICCGTCTDMKDSSQIGCEYGDKST

WCTSKLIPQTDCPYNLDLCCGTCSWGGSGSEQSLPRQFLRDSGSGTYQPMQLEGRQFPQA

PNRSSPRPLL*PAFEALIVKNSFVSVFFPLCFSFVFPNVKSYYFELSFLLTMQKSRPVLI

*WPDVLRSGEASSGCLYNAWRSTVV*RRHILQISGQTVRPPRLIRCYGWGFDVIYTEVTM

GLHVPNTLIYNISGLLVDVFAN*SGICPENCLTFRCIMRFPCYPLGRQEVREKLPSPHAS

QV*QFVKIADIYFLN*YLSFFRLKLNLSSR*YSNL*IPYAT*M*GC*NMV*HLQIEMDIC

MSSCICTRQGTGCSEACMHNVITTLTIYSNL*SILLCKYLMWLVIGQMGI*RWSTDRLGQ

NLC*FTDIWVYRIVLVAEFCVFALELDLIKGYLSCG*PKDAQNIVFWLWHNTL**CTSHA

ELMF*RNVIHVNTHC*IIPSFIDIYYSHFAK

>**comp98044_c0_seq2_2** len=1470 path=[1516:0-58 1575:59-108 1625:109-129 1646:130-263 1780:264-270 1787:271-569 6139:570-574 6144:575-586 2103:587-594 2111:595-1113 2630:1114-1130 2647:1131-1259 2776:1260-1391 2908:1392-1418 5283:1419-1469]

EALI*RRRLTSTDTHHSTWPQRYVCSLAMIMKMLKNAGNLHVAFLLCLINPCLSTDVYKY

FTTQGRDRLVEALFDGDRGYADCAVFGDRQVVGTILKEVSAEKIEEISKSDLDLYIEKCH

EFHLTLDDSPLRRKRFAAIYPGTKWCGVGNISSHSTELGVHNYTDACCRQHDYCPDYILP

FASKYGLYNFALYTRFDCACDEKFYQCLKASPDDAAVYVGKIYFNLLSIKCIGYGHPQTC

IKRWWFICLKYGEDKSRTVRKFTNGRRF*CGPWRR*NMFFFISLLLYKIKHCTYVKQH*V

IECQRHSLSDDGNFDTAVVITSNLGTHHKQKTRVVFVAGLQILWKAFLLYKHTLY*RDML

*FGLFLPEH*E*QKFQM*C*FRLASNASLLYDNTYVQVLK*DCDITIFHVFVIRRGECSN

*RVYAMNLTWCFVRGFCTK*MMYCVDRSNVIV*RNTNSCRVKHKHTRTRAQTHINYIYIH

IHVRERECEX

>**comp98057_c0_seq6_3** len=3028 path=[3060:0-809 3870:810-1268 4329:1269-1510 4573:1511-1918 7502:1919-1931 8499:1932-2090 8558:2091-2158 8582:2159-2200 5268:2201-2216 5284:2217-2569 5684:2570-3027]

YISGGDQWTFESENLGYLHYQGPSTMASGASKVVVCDNGTGFVKCGYAGQNFPEHIFPSL

VGRPIIRSTAKIGNIEVKDLMIGDEASALRSMLEVNYPMENGVVRNWDDMIHLYDYTFGK

EKLNLNTKDCRILLTEPPMNPMKNREKMIETMFERYQFQSVHIAIQAVLTLYAQGLLTGV

VVDSGDGVTHICPVYEGFALPHLTRRLDIAGRDITKYLIKLLLLRGYAFNHSADFETVRM

MKEKLCYVAYDVEQEQKLALETTVLVEPYELPDGRIIKVGGERFGAAEALFQPHLINVEN

VGVAELLFNTINAADIDTRPEFYKHIVLSGGSTMYPGLPSRLEREIKQLYLERVLKGDVD

KLSKFKIRIEDPPRRKHMVFLGGSVLADIMRDKNDFWLSRQEYEEQGLKVLDKLLKPTKS

*LPLTTSLTVDQLVFFLNCQP*DTSVYID*GLMSILLTALSIRLGHCILTEVRKHDQSLR

KKIQSHLLLKLSAFSCSILIILISQFLVLTA**TIQ*YYCINEVWMDLTPMNKG**YQWC

LKQEFAMIYML*FSASVINMIQLLGAQLTSFV*SIQKCCMCLSIRIYFYDLKCIDVIYHL

VSVEPFCLYSMHEVCYFVMEVVQSSILFVFA*IK*LCCA*SLMTLKLSTTLAH*MKCDFP

TFHFSVFNHGIDEYYRHTALVFMEITAIIELRLS*FSKCFEKKDFHSKYVFFCVEEGKNR

RITWFKRLLDSLGMSVTVGLLSCIFRVHQDPVIVGLNPRLSLIASWFVSTKRVGFLRWKT

FPSTST*PVMIF*NRVQMSLDKITHSFHTWPVGVALWSACLLCLQKTWVQMTMLMQWERT

SLCPTAYILSRSCVELM*SESF*NV*KIEETLKYFDCLFFGPSDG*I*QI*VHCITTLLL

M*VYHVYHIEGFAIDQVNCPVSEAMIEIL*GIYGIKIII*VKLKGLKPSVYV*RLHWLGL

HFVPMFSFEMTSHCGLI*RYMSSC*LNKKI*YHKATECFLAVQGPVPQX

>**comp98316_c1_seq2_6** len=994 path=[10366:0-31 10398:32-80 3218:81-98 14781:99-116 3254:117-993]

HEFE*TTLSMWSFQT*SGYLQGRTMYNAAYCSVLGLLMFGLSLMIMGEDVSKPKAKRYND

KSFPKHSLVLDRNVTCEDIPEEKVGKFISHTWTCHNIRVDGYRLAYGYFPGIGIDIKCNE

TGGKHEFAQLCRELLRKVATDRPLHALYIPEVSCDYNSDTRVLLNRTSEEEYTWEELQPG

NGWVGTIQCMVVLPGKS*MQTLLPRYASPSCA*RMCDCPHHRDVCLCPLLELAWIIL*SR

QVVCDVIE*YVMQRMHM*SVLVIHDLFVI*VMHDPSKSYIIQVSHI*SN*VSMI*LDHI*

SNWVIHDLIESCMIQVSHI*SK*VI*SS*VX

>**comp98408_c0_seq1_5** len=3318 path=[1240:0-59 12696:60-82 1301:83-277 1496:278-293 1512:294-356 13191:357-366 1585:367-1826 3045:1827-2015 3234:2016-2022 3241:2023-2953 4172:2954-2977 9883:2978-3317]

MVRHIGCREGNRRR*YWLSSA*IVIICVCGVNFPFELCLLSFLVGTR*LLFSKIISYWDI

*AFVYKRLPSGRAKKMGTKDDEYDYLFKVVLIGDSGVGKSNLLSRFTRNEFNLESKSTIG

VEFATRSIQVDGKTIKAQIWDTAGQERYRAITSAYYRGAVGALLVYDIAKHLTYENVERW

LKELRDHADQNIVIMLVGNKSDLRHLRAVPTDEAKAFAEKNNLSFIETSALDSTNVEAAF

HNILTEIYKIVSQKQIRDSPDDENSPSSNVQTITVAPTDNSAGTKKQCCNV*AQITVNLK

LRELGGKKSYGRIGTCLVQLGPCSQWNTKNKGHWKRWLQ*YVFVHSCVSHLCYGPNRENE

FLSPLADHWCITVVHI*Y*NCPSIS*LFFFSSF**M*FRNR*LDNTVPLPR*YGCSVYVK

LMQATNSH*GSVFWTRHLLNTVV*FTELINTGKKHCNAVCQ*YLC*GRGDLLDICHPYL*

FALALLD*LLRHL*DATMITGRLHPTTVKHLEKASYYLYFNFGDYSCQTNGITCRIASSS

LAYLCDR*HIIRYLCDHFKDTAMITCSTPL*SQRYCGIPP*SLVGCLRDQL*DTSMITCR

ILSVFPW**LMYTFHLVLFMADCC**NSS*SSEFLVRLCMPEVLLCSGMMLTSTMHDSLS

GNLYQITLVI**LTE*GATN*STPYSVISLTAPTDFIIQYASLFQTNELNLVVYLANLSP

LSEIHLLAS*NNAQLDVQQVL*VISLWYGFQLRFLYGCSGYYVL*MD*VETTVYIHIDTH

TQPWLPPTNFNGLSFFCLRYKA*NTTVGLFEFH*R*HDLNLRIVKVQYLLLLIMQILLVA

KA*KVDIYFCVIHFADLLPCNDV*NVPSKALPCGRIHTFR*FDI*NLGLEGHVA*RRLFA

TSSTRWFWNQD*VEW*KAVTTKHPVLHHQHDSSQNDGTEHIKIHVPES*SKSHYTMKLVG

SLF*GSIAWSFMKFFMVLPDSISTQFLLNHPSVSTVTFDCSKTTISTSFQA*AVLTVEKP

LIQSPTLPVQFGVVWVK*RRLPLFNIYFSVFWSVLALLVSAFDIVSVLICSTLMGVFRFN

*I*YHIKWM*FCDQIQFRRTCLPKLX

>**comp98445_c0_seq4_2** len=2358 path=[5373:0-68 5443:69-136 5511:137-986 6361:987-1799 12623:1800-2067 7443:2068-2272 12933:2273-2288 5327:2289-2357]

YSMASARNSNDNSTRHQ*IATIFYCCK*ISLKCNMNLYTRYAFVFLFSISTTVGGDFDPF

MDAELFKIIWPGSSEIDIEKTVDESRVVVMTTNKERYQCILPESHDKRDSSKADYGGQTA

DELMNILFTQTTCSYRIESYWTYELCHGKHLRQYHETKDVGQKPKVQEYFLGYGKQDKNE

DSESSNIKIRKVEGISLPYYEVNMTDGTACDLTQEPRRTRILYVCQPDGHGEIYEFKETS

TCEYEMMVLTSVLCGHPDFRPKNAPVGEIQCHALDGSPAKPTQLKRMEKTVVHTRTPSPE

PTPQPTPQAVPDNVATKVPPQYVEQHNLGSTTDKQLLREFLTGEYCLKGGQGWWKYEFCY

GIYARQFHDDSQGRTVINLGVWKEAAHLQWLQQNTQKRPREIGKRKFISLFYSDGDICDI

TGKPRTVEVKLKCVVNTNHPHSVGIYLMEPSTCSYILGIESPIFCSLLDKADDNGILRSI

ET**F*VVLEKGVLC*KQ*SYSCFTNATIWN*IALLSFSRR*YIDLYI*CLCRGPDRKLA

SN*INLI*FTTKSHY*KPHHTHIEVYLFVQSCMSGSYYRRCRSPIHRQYDPWCASTVPTL

ASHDKLKWCCRRISSHSSISCMCRHS*TGSWSYRPYFVIFDVFCSINISH*CR*LLLLRD

I*N*DS*IQVPFTEVMLSWGKQFCLQHFCFVI*YCDMSVALFFSNKRIVKCLYGIFR*SR

QHVFSQAVVCKD*QHASDSKTNTSQCRGARFMEDSFSIAIDATFQCRS*HCYQVIHNIDD

DDLEMX

>**comp98866_c0_seq1_1** len=3558 path=[1:0-163 165:164-165 167:166-323 325:324-2413 2415:2414-2430 2432:2431-2654 2658:2655-2725 2729:2726-2749 2753:2750-2843 2847:2844-2867 2871:2868-3005 3009:3006-3029 3033:3030-3057 3061:3058-3081 3085:3082-3089 3093:3090-3113 3117:3114-3133 3137:3134-3157 3161:3158-3177 3181:3178-3201 3205:3202-3392 3396:3393-3393 3397:3394-3417 3421:3418-3557]

LSQRQATLVIYWTYTLDESAATGGSGVGELFRWD*EC*LHVKKHGYRYIIRPQQ*GRMPS

AS*LTDQP*RS*RMFTKQDVG*TEPPSTLV*SWEGWKTKSDE*RNDGWTIGGVQLNPVLF

LDMFVFRCLRVRRSVLLVIMSLGGFLIVCAYLLALSVLYVVPLSDSLVVSERSSTSDLSA

LGDKLQQEEFSDKTRDSPSLLVSRHNRVLQNSHAQEISERGGKVISGRNKPSLGGNTGDN

FVSKRSKMDNRLTNTASTIYLDEEWSDSGRGEDEEEEYEIFQEGGQVKRKDSTESTRLAR

VQENATKKLPEALIIGVKKGGTRALLEFLRIHPDVRAPGPEPHFFDRHYNKGLQWYRRQM

PTTLPGQVTIEKTPSYFVTREVPKKVFNMSKGVKLIVVVRDPVTRAISDYTQSFTKRPYL

KPFEQMAFIDNTTRLVDTKWGAVRIGVYAKHVERWLRYFPLEQMHFVSGENLISDPAGEM

AKVQDFLGLKRIITDKHFYLNQTRGFPCLKKPEGSGRPHCLGKTKGRRHPHVDPDVVQRL

RDFYRPFNLKFYQMVKQDFGWT*PVRS*V*LLTAVTSCLHDVS*RTLCCVDGDVVC*QGW

SMC*PWGGLIQGIRNRDLV*MAPVLRSGCVLRPSAN*NNETHCELSHDCASRGNRFHTKS

TGKCKQGFL*RHLQ*EANMFTVCPHYTFLMSVALLGVIGTRLPGRPKK*RFVKPLNSEIQ

RPVYIPNKMLLAYFENAVLYILWL*LY*VFLKSFWN*FNLF*KLSF*QKLYHNLYNHSNI

YDIV*NVM*RN*YTEC*ISLK*PESGSDECVRCS*VRDGTYFRSNNVWIYFVEKYLQLLY

LIALPPAGPVLSSYTSDFTR*DLRAHTR*GTNNMAVSLEFFFFLFSITFSRRRDTLPFVD

GHKTT*SIYVSPNIRC*FETLSSM*RQN*QYHRLTRDVLFLLP*KHTGSSDLADSL*PFF

H*MQL*RLDKYTLRNVYARFSYLYSS*VLKQTYFTRVKHTCQMPPYLASKNSHYSNFHAN

*TLSYFIG*WDITC*GDGSRDPVIST*RRYVETTTRYHLKPMVLSFVLIYY*VYIHCKVV

SMKSK*TLTACTDIITMGILTVESVANIVPSSIGTSTTDSIST*NPLIGMVLPGYDEQKV

AEGNCTLVSTCL*SRNM*LIPRDTD*PVGDAC*YCSCYEEINHIFS

>**comp99181_c0_seq1_4** len=2094 path=[5612:0-33 92:34-57 116:58-89 148:90-208 267:209-228 287:229-292 6861:293-316 4243:317-349 6895:350-350 6896:351-352 6902:353-357 6907:358-358 6908:359-359 6909:360-360 6910:361-475 524:476-1171 1220:1172-1177 1226:1178-1204 1253:1205-1250 7055:1251-1855 1907:1856-2093]

RLETTALCK*IRLPVFAGTEKCAVASQSSNRTHTYTINETVDVTHGCKISYLCQVWVTSG

WLFLRMDVRLVFGVFITFLVVPLVSGQARCDLAKASDCIEELESILNDTKDEGRTVEDKI

RMCSMTLRSNNISTCYQMNSAVCPDNPDKQRQASRWTSITGEIDLFCDNDCQDYREIKTC

KEKVDMDAIEQGAPSRFCRTYVESKQCLADDIDGDNCEYKENIYDALFEGPYIRNYYNNV

CRTDCPNLDETVNILQNCYTSLENHNVSDVCPAHRNFSKCLNSFPDKPCEQFRALLPFLF

RYDYLQNERMCQDQACDRARICQSQVQRQYDSTLRYVVQSSDSVQLDDLCLVVIRGNEQT

DCYSKYIDECTDVPEHPLRQQWGEMVMDIRQLCDKDCANVMNGIPSCHDAVLYERFNGNM

WSQFCQSHSNATTCVKNLNCTLGTSMFNKLLRSEVEGFVANVCINECANLDDTLLSLERC

FPFLSRSGTPGDDCKQYYAFENCMGRSTVAPCKEVDGILNFLSPNFTSRKDRCTTTTRPT

SGASTRETTATMLFVTLMTSFQIMGRL*N*DLETVDTQEKTFQQLSAPRQTKIRPSFMVN

IFRLHLG*FTKFYTLF*NLFDLDQYDLKCFKFENSTLWFYSYTDLNTLQGLRSDKSPIVQ

*LYYLPLLAASSHSSQSDKLLQPSLPVSTMKISYRQID

>**comp99476_c0_seq1_3** len=3329 path=[3309:0-264 8103:265-265 8104:266-1076 8211:1077-1088 8223:1089-1279 4589:1280-1291 4601:1292-1764 5076:1765-2067 5379:2068-2209 5521:2210-2737 6049:2738-3124 6436:3125-3328]

RVALADSVCDRYSCWI*CSDMRILLLIHLWTVGLCLEEAEDDGGWTTEKHLLLAHPGPCS

IEVREADSLSQKEFLEKYAFTQPVVIRHATDNTKFQDACRKDYMLSEHGTKKIRLSSANT

YSYNKVDVSLHKYVNEIMKPQTLDMLGNETFYWFGDNNHTEWEPLFTLYKPPPYKLPKMS

AVYSFGLAGAGTGVPFHFHGPGFGEVIFGRKRWFMYPPDETPDFHPNRTTLHWLIEDYPL

LDHMNKPLECTISKGELIYFPDRWWHGTLNIDTSVFISTFLAQA*MGDLVNHRSISLKGK

SDFLQIYKFCQCRMLFMKIYLLKKQTLVIIYIFLFVSYLNIGILHDRIIVSGNKFVNMF*

LRLLSFSVTIS*ISFITIRIKNHVWKVESIFLIPVLNFILYHFSYFNI*YL*SGNT*G*W

SLYQCVRSV*DIFSMNMNDFTNHK*IISCQMI*SHTYQRKHRHIEKHPSQVKWKVRQYYV

EEELFESSSH*KIGPHLHNFIGLPYWIILLHGLHYPLNDHSYSSLVLCHTPQHSRYITTT

CTMVPG*TSGRWNEQRSMMSGDGSQGF*RLMVHPLPQEVPKLDQMTPQCFVEAICISLLT

RQTIGLGSMLSFTLPHTLSC*FSFKCKK*EL*KIPES**IF*MITIRFFE*HKAE**PTM

FSKW*PMTLDNPPYQ*IITL*TE*LAIRFSELNSLKGYGFIMLGIVPCITSVQIPIMIYM

PHLLNFTLILLKHIYI*YDCGN*ILQRCFQTILTELCEICVKYKYGYIIVPHHVICACV*

CFSVEILMCSSMWVPGRLYQ*GVSVTVGRDCTRSEMLMHHGTADLGDTS*C*MTEKC*GQ

FIRRGEEEKSERENDDDLLCVCVWGNQFSTHVRGDVSNFVHSNIQEL*LKIMGGWALTL*

MTCSGVTYFVHFGFFWGEEV*MVPYLYLQCSHIPCDKILFYIVIFCRYRYIQYFVDYTFL

VQWKF*YFLGLSNDWIEPTSNG*LPAAKFVESLVAAWVWSLTLCDGNLVSQSESKGLSPI

LNSPKNSRICTLLRE**KMTGCLEILMTIHNK*VA*CEPSLYNWMEV*MIYSSYEK*TFM

RDSE*W*M*CVFVLFHTCSSSELSFTLL*

>**comp99505_c0_seq1_5** len=2162 path=[2140:0-311 6103:312-326 2467:327-333 2474:334-390 2531:391-431 2572:432-476 2617:477-516 2657:517-535 2676:536-559 2700:560-569 2710:570-635 6210:636-645 2786:646-1182 3323:1183-1192 3333:1193-1315 3456:1316-1363 3504:1364-1382 6284:1383-1701 3842:1702-1726 3867:1727-1782 5164:1783-1783 3924:1784-1823 3964:1824-1856 3997:1857-1911 4052:1912-1936 4077:1937-2003 4144:2004-2006 4147:2007-2161]

VNSQSNAEVFLSSSEIIWANLIGNYTCSATQEADMMKIRDCGERIWTGVWLCTFIAACCV

ATAAGGKTFVCYYQPSARVPIARIPADLCTHVVYAFAEASKTGISPAHTTDLAKFSEMVA

LKKVNPNLKVLVSLQNGFPAVVDGGVAAMETFAKGAVSFLRRLRLDGVDFDWEFPSKTQK

DGYMKLISIFRKTVNDEAKLNGTAPLLLTMALSNNRYIAGWAYDMSVLTQNIDFATVMTY

DFHVFNAKHDNVTGYNSPLAAPKGENPYLSTIGMLSFYIKAGLEPSKILMGVPAYGRSYT

LADSSKHGLHAPAVARGAPGPAIHVHGVYIYQGICMALKSGATRVWDATAGVPYLYHNTA

WVSYDDDESIKGKCEWAVSSNLGGIGVWSLTLDDITGTCTGKSFPLLRTIKSCLG*FFQN

PLPLWCSSVI*PNNIFFFHFISFYALTK*YCHMQQYPFDMGCFRNRSDIVFRWIFQDGNI

D*NTNTPVIGYFETGYFTVVVYWYFPRVNLHSHHSSHLRCVIFLHSNAFYPLVLRLYTHY

**TENPNN*LTFSLPS*EFQVPVPPK*TGNKD*NLRQVVIFVVNGLCLIEWSGLVFCWAL

LDISITCLSCPDSNSVKA*C*FGNLDKTIFLSNLVNQTQTNNATSC*LHPRTTCITSRPS

SYPVMFVDTMFLQNKFTLKTELTFYTLYKIYPLIN*CLFMYYLLTEYVVDIDLILTTHAS

X

>**comp99703_c0_seq1_2** len=2817 path=[2795:0-1060 3856:1061-1739 4535:1740-1764 4560:1765-1802 4598:1803-1812 4608:1813-1836 4632:1837-2307 5103:2308-2429 5225:2430-2816]

RKATRCI*HSV*GQIRNFHQKEDTRTST*DSLKANRGVYLNTFIQDRLYIVHKQEMKSLL

TLCLLALFLSVMWSVRAEEEEDEDADKKKEKDGVGTVIGIDLGTTYSCVGVFKNGRVEII

ANDQGNRITPSYVAFTPDGERLIGDAAKNQLTTNPENTVFDVKRLIGRSFNDPSVQSDVK

HFPFTVINQKDKPVVQVKVGTDTKQFTPEEISAMILGKMKEIAEAYLGKKVNNAVVTVPA

YFNDAQRQATKDAGTIAGLNVMRIINEPTAAAIAYGLDKREGEKNILVFDLGGGTFDVSL

LTIDNGVFEVVATNGDTHLGGEDFDQRVMDHFIKLYKKKKGKDLRKDNRAVQKLRREVEK

AKRGLSSQHQARVEIESLFEGEDFSETLTRAKFEELNMDLFRSTMKPVQRVMEDSDLKKT

EVDEIVLVGGSTRIPKVQQLVKESFSGKEPNRGINPDEAVAYGAAVQAGVLSGEEDTGDL

VLLDVNPLTMGIETVGGVMTKLIPRNTVIPTKKSQIFSTASDNQPTVTIQVFEGERPMTK

DNHLLGKFDLTGIPPAPRGVPQIEVTFEIDVNGILKVTAEDKGTGSKEAIVIQNDNNRLS

PEDIERMINDAEKYADDDKKVKEKVEAKNELESYAYSLKNQLSDKEKLGGKLSSEDKDTI

TEAVDEKIKWLESNPDADVEELQAKKKELEEVVQPIMSKLYEGAGGAGGAGGPPPTGEEG

DHDEL*ERRGVGLVYKYCHVNTHLMD*HFIMEHEASCYYSTVEKHLVQLMWNYLFSFTWI

SDSNRVNNENCKCDQDEL*NMICRF*RDTNHLQGTVLMNLNHGVEKLFVLCFGNYYRE*N

IFVIADVCTSLFIL*GK*HHSD*LSKNWKLRNWFSVFWHFRLI*LMS*LFMASVCEFITL

VYTVITWCVYICIHLIIIRMKAGVHCGCMMNESVKMLKX

>**comp82445_c0_seq1_6** len=1140 path=[1118:0-286 1405:287-323 1442:324-368 1487:369-383 1502:384-1139]

VTSIDPLCQYRGTV*PRPPRSVTPVAITNQALAIMRLLCLVCVLFLQWRSTLAFLCSASE

CVAEATCESGVCICPENLPDGDGRFYCYEKADRLVGIEYGDPLVHSLIPDENYRFPTPCG

HRVLTGNIPGDCYISVYANGPDLDTVKRISRTFINGIEVIMKKGSKETRMIVNNEVEFRK

NDLPVTYKTESGLVDIQIPECSTVIKYHSANPSLVIDIPKSNNLYKGSMLPTSDPAMTLT

QQAERYNLTVKQDTLVTSIMNINSKYSECQQLHDDFASYCQDVSDEDIATLICGQYVFTD

VGEDCLARDKTSRELIRTMTECFLDVCLYKKPPCDVITKHFYSCKQPLSAELASFKCTW*

KPHHSTLPLPVLFNKGR*YX

>**comp292963_c0_seq1_2** len=281 path=[259:0-280]

LLLPLAGVIFLALHAAQGSEPRRVCYYTNWSQYRPGKGKFTPENVDPNLCTHYVYAFAIL

LGNKVKAFEWNDLSTQWSKGMFERFIALKTVNPX

>**comp94020_c0_seq1_3** len=1732 path=[1710:0-267 1978:268-280 1991:281-304 3478:305-310 2021:311-390 2101:391-394 2105:395-1286 2997:1287-1394 3105:1395-1462 5000:1463-1468 5006:1469-1542 3253:1543-1583 3294:1584-1706 5099:1707-1731]

*NFPIYVCAWVVFISSCVDQSSVLAPKMTPSAYLTAALIALVHVLVCFADELKVENTFTP

DECKRQSKKYDRLKMHYTGTLASDGSKFDSSHDRSEPFEFQIGVGQVIKGWDEGLLDMCV

GEKRTLTIPSHMGYGDTGSGEKIPPGATLKFDVELVDIQDGEAPPNVFKQIDSDGDKKLT

QDEVSNFIKEQMKDSGAPTEDDEAHNKMVTDIFSHEDKDKDGYISHDEFSGPKHDEL*AL

MP*DTFNQY*ILVVSVC*NQVFTVSFVSNRTIVALKMLCGIGYCLLVLCRNERKFTSIIE

R*IIHISILDISL*KGNCQKEQHFSYF*TVYIQFCSFVIQKLL*LFL*NMINHILVLNLL

SLLFKTPTRSLIC*EYSKQIFLMNTNQKRQHCI*HHVIDRSLTLSICHHAPVIQFENSI*

NISSSQCFNIC*DFSNRPLLSLRLYYISWVSD*EVSSFSIFPLWKSCLQNW*CFLIQTFQ

SVFVYIKNKFCVCYLVCEIRIKEEKQTCKTCQNIIFSAKRQIAH*NKVLKCLFLSTSKRP

GDS*AICCVCNEDGCVRNYLFYVFQFCQNICKLRI*R

>**comp427051_c0_seq1_4** len=209 path=[1:0-208]

FYCYINPSSTITCDYSQHIDVPLPSRVEIQNFAKQIQGGILQKSECGEFCARFRCIYEAW

SDDGRGTFD

>**comp83487_c0_seq4_4** len=274 path=[1:0-102 104:103-128 4660:129-273]

DLQLERINVYYNEATGGKYVPRAVLVDLEPGTMDSVRSGPFGQIFRPDNFVFGQSGAGNN

WAKGHYTEGAELVDSVLDVVRKEAESCDCLQ

>**comp46938_c0_seq1_5** len=235 path=[477:0-37 251:38-65 279:66-234]

LTTPTYGDLNHLVSATMSGVTTCLRFPGQLNADLRKLAVNMVPFPRLHFFMPGFAPLTSR

GSQQYRALTVPELTQQMFX

>**comp103351_c1_seq9_2** len=2919 path=[10370:0-316 2498:317-382 17439:383-385 17442:386-391 2573:392-462 2644:463-466 2648:467-1046 17662:1047-1049 14805:1050-1071 3228:1072-1083 17673:1084-1098 3255:1099-1113 3270:1114-1726 3883:1727-1754 3911:1755-1828 3985:1829-1842 3999:1843-2201 4358:2202-2215 18090:2216-2550 4707:2551-2553 4710:2554-2661 4818:2662-2669 4826:2670-2685 4842:2686-2686 4843:2687-2687 4844:2688-2688 4845:2689-2699 4856:2700-2726 4883:2727-2738 4895:2739-2798 18345:2799-2858 18346:2859-2918]

LLSKL*LYIQRLGNSLQAIMNADFIIIINNMDHTV*VIILPISIDNDYQYQALLITTDCH

DDSININK*TVGDKL**EVSVLRLDISSFKLMHLLLM*VMHFEFISDTTTAGTTITATTS

DPTTVATLPSTATTTAATIVTSTTPTPTTRTTSAFSDATTTTLASTTTPTTTPTTTPTTT

PTTTSTTAPASCKMTRSSGTTGVSGYYKFSLRNVDSCSSICLYIRTCRATTYRLGICTLY

PGTSTAASGDIVDFVRHACNNGTFCCMSSSITQRASGSPLLSISTTASLDGCEAICLAVA

DCQGTYYSDDSCFFYTTTSTTFGGTIVFSRKTCPVKTNPGNLEFDGTCKERPKWTYTRYQ

QLSFFSHYPWHYYRVPDYTYFWDPFLVLFLFEELL*LTDKTVMTLIQRGKEEFGTM*QKE

*KLMSKCVAQNLDVCSTEISLP*VFPGTYCRIAKSMEHIWAASDKLHLQIQYRK*RIFFT

RNHLFTRRKSIQFLALY*L*IFTIYDSCTMFPLCIVSFFTGYGWSKVALFTVQSCGH*KY

QNPLRVNRLLTYTINVLYSVRNT*VLGGYQLG*KTGKYKLPFSLSKLELLELDFYI*NCC

MKLMHLQKQQ*RDCLLFSLAKISGCINYGSDVGISAAEPLRLNIYVWFM**CVCLCKHVY

CT*GHAGVPRLTTL*NRCKYCCAHINIVVITITDCSQTA*SAYQLCIFFSLFILTIPLLF

TMVYAWGKNILT**WFNMDLQAFDHEKIYS*STRQL*IVNRIFKPYIHGSIIQCMRRPVN

TIQFLKIEYCLPT*RLFTQQYVHMEVFPLARMWRGCSRHIPLRTYQQGYRNIPRCYCNPP

LYTAGRIHGRIRRKVITLGVNYVYAYNDLTDITN*YFVPGHLLTILI*RCMVITN*ISGH

LSSL*P*ATVSVSLRTDDKGKWTES*SSTHRG*LRTDDKGKWTES*SSTHRG*LRTDDKG

KWTES*SSTHRGX

>**comp101634_c0_seq8_3** len=1283 path=[2891:0-100 2992:101-129 7643:130-155 3047:156-234 3126:235-258 3150:259-266 3158:267-290 3182:291-313 3205:314-321 3213:322-334 3226:335-337 3229:338-358 7815:359-359 7816:360-382 3274:383-454 3346:455-502 3394:503-547 3439:548-571 3463:572-574 3466:575-591 3483:592-598 3490:599-612 3504:613-615 3507:616-636 3528:637-695 3587:696-720 3612:721-722 3614:723-766 3658:767-785 3677:786-809 3701:810-827 3719:828-844 6893:845-868 3760:869-944 3836:945-968 3860:969-1043 3935:1044-1086 3978:1087-1087 3979:1088-1118 4010:1119-1141 4033:1142-1247 4139:1248-1256 4148:1257-1257 4149:1258-1258 4150:1259-1282]

RLVCRVIDAQELVLNPNTTIVMSSPNKGLVINCEVEGITDGQRANVQWLDTQGNVIGPYS

STRTPRIYSEQIGNRNRLRFVTLVNEDAGTYTCRGTIVGKIMTKHIQLSLNNNVLPSIKT

PPTWSTPTVGKRFELTCLAEGIPPPSFIFDKDKFALSGDSHDRYNVSVTKGVNETQGVLV

IEDFHYADRGNYSCIAYNQAGRVTRTVFIDTHVPPVIEPILNISKPEGSAAELVCVAKGD

PSPVVTWRVKGSDLPITDSRDITVEYIQKEQPLMHVTTVKLKFTRLERNDANTYTCSASN

VAGTDEKEGRLAVEFKPNLADASYSSIYGSVNHKTEIKCVANAVPLPVLFWKHNSIMISQ

GQSFRISSKQENQTIISILEVSVTAENENTIFGKYTCVAQNILGSSEKDIILKQAAVPSA

PDVSIKA

>**comp56242_c0_seq1_1** len=455 path=[1:0-454]

VDWAAQYWESLGLPKDKLVLGLPTYGRTFTLTSATLNSLGDSVKGGGTAGQYTREAGFLS

YYEICALINAGTPVHRIASQRVPYIVSGDQWIGYDDEESLREKVRYTLNSGYGGVMVWAL

DLDDFTNSCGAGASPLMNTIFNECGLTAPSPA

>**comp118790_c0_seq1_1** len=1114 path=[1:0-1113]

GTPDMSGMGGIPGMPGMPGMPDMSGMAGMPLVPSAAMGPDSIPAIETQVMALENRLLTHF

KYPNPDPAQTAQMVAEYDVLQKNLKAAKRAAAVSSSPMPGGSSSASQLSVGNTALNSRKA

LSSMNNPFDFTGLGSSASGNSSPLPSLVSTPQGLTPADINTLPSPGGLQAAPVLPGLPGT

ATLEPGAKDILEPMFERREDINKARKLAATMSFIQQITDPSSGSSMGPMEPMNGGNNVFP

GTGQSSQPAPSSGGSGASSMSSLGMHSGQLPGGFFPGAASQSLLHSPMPDIFPQSSAADP

QMQQFLQSFQPPAGMSPFFGGAR**FLLEAHVRFEKFARGMMYWLQDEL*PRFDYASKKT

LIQSNKKQKKRX

>**comp184680_c0_seq1_2** len=342 path=[1:0-341]

FGQQSTSAFSHQSPSTHSHFLNQHQSPSTSPFMPDMSRQQPGAGLGGPASAPTQAQLLQQ

FAALDSKLSSMPNPTPAQLQSVMSQFQQLERQMEMANNPASPSSALTPDMSGMG

>**comp186443_c0_seq1_3** len=345 path=[1:0-344]

KTTSQPGNGTKTNDAQPGTMQQSNAAKLAMMQQAAARYEAMMQQARSNFNAMNSQIQAHM

QGSMGGPPTQQPGQFNKHTTFSTMSPPAPTPDVTLPAQSTVNTGSSNPSTSASQX

>**comp188351_c0_seq1_6** len=718 path=[1:0-717]

PSSSEAVTTAVEQGTTSAETATTTTTSATTTVEPASTTAEAANSTMDQTTTTVATTTASD

NAIEVCELEPKDCKDMSFNMTGCFCYCPHKAHTNAGMFIMSVGHVEYAMFCPRGLVWTQA

KCMCDNPPTKPSEPDAIKDLPIFSSGCKTLVNYAYNATSKAFEDGRNFVRQENTINKRLQ

VVQSEGATVNNSAANFINNSIDIPYLNGNDLTDTAFIEVRIKPVADIDNVTRVILSNGX

>**comp34113_c0_seq1_5** len=860 path=[838:0-859]

LSNGVCENMQPDNDGWYKLTLSVKNSKSRFLVNDQECLTLNGRGSIAKTTCNLSIGGDPF

MTDYSQDFNGYIDYVKVIKHCSKEPSK*KRNT*GSRADHLIRKPSH*RIDVNSMNSK*IA

PYIYIPHVPH*HPSNTFLTTRRNVNSHSDCSALT*RVA*YSCDNM*NMNI*LLP*FKSSF

NDFNVKRTLVAVVFLGLTVSILFLSLKLGAGPCVSLCSYSNAVAS***I*NNEI*NIGSR

SVLGPKRPVKGAYDRSFVFYTVLLVSCILVNIVGCSSIFKLFS*LLX

>**comp252421_c0_seq1_4** len=237 path=[215:0-236]

RDQGFFPHPRKCNKFIMCNDEKEVIQTCMHSLVWNPRVNSCVPKTNSVSCPDEDT*L***

RHADMYL**KTS*SGTLAI

>comp52612_c0_seq1_4 len=206 path=[1:0-40 42:41-50 475:51-205]

RVTRPVPQRITSVTLKFQRQKAGMWRKYLLPVTLTLCVISDVFSGTNSNSGPRRARLTQL

DYRRDFTI

>**comp81625_c0_seq1_2** len=835 path=[1:0-266 268:267-271 273:272-410 412:411-560 562:561-576 578:577-753 2142:754-780 2149:781-807 2150:808-834]

*K*RVTQMDVDSNCHACHWRT*TTLTLWWIILHMQVEHGRSVFDTLDYQKCMDSLE*HIT

VKDSWITFEIIHLNTAVLRKPQWTTAMMMKTVLSVALSLCVIGDVIPGTGWNSVMAVVRP

DTFIPDNREDIAGHGGSALLSMMCAFCRQQGNQPCIDRMCSKTSDSY*IARR*GRIPYGV

SGFGSTSLKTF*HCSTHRQDMYFGILSHIISWTSENNDEEPLPHSLEGFNVFVYVIILL*

CIFCNQILHLGK*CLMVTLRK*CLMVTLRK*CLMVTLR

>**comp52399_c0_seq1_3** len=248 path=[226:0-70 297:71-74 301:75-247]

AVGSRTVVIAFVGAALLAVVVLYTTQDQIGYTKNTIMRMISSEVSTQVKGAVVPLNENKD

PLKITEWWQASSLLKWDWEKPP

>**comp59338_c0_seq1_5** len=1104 path=[1:0-1103]

KVTGSGGLTFSNETTLAFNRKSMSILIQTDKAMYKPGQTVNFRAFAMFPNMKVYTGALDI

DIFDPNSNKIKQWVGLKDASGVITNYLPTSSQPVLGDWKIKVTAGSMTEEKVFTIAEYVL

PKFEVTVELPSYALVTDDHITATVKAKYTYGKPVKGTINLQAKMEQYYRPWNYHGEEPMV

KKTLATDGESRVTLSLADMKKVFPNGLQNREIRVSASVTEDLTRITLNGTSKVKIYQYAT

KLSFAPSDPKTFKPGLKYIAYLQVQQQDGRPVQTSTQRVTVSTRVTSQIKETTTHAFYYP

RTTSFTIPDQTFSVPDTGLVPIEVQVPSNASSVSLTVKYGKVSTSKSIEKSYSPSDNYIQ

LLLKSQQX

>**comp32926_c0_seq1_5** len=1615 path=[1593:0-1614]

PTGCGEQTMLGLAPDVFITNYLTATKQLTGDIEEKAIKYMESGYQRELTYQHKDGSFSAF

GDSDPSGSMWLSAFVVKTFHQAKPYTFIDDDVLTRAINWMIEKQNADGSFPEPGRIIHKD

MTGGSNKGASLTAYVLIALLENDDLTGNVQQRINAAAAKAVQFLENQTPTDDYSLAIITY

ALQLAGSTQADTSFATLNSHAIVKDGFKYWHRPEVKTTQSTRSYWKPPSAKKAIDIEMTA

YGLLIYGEKNDFSGGLNIMKWLASQRNPHGGFSSTQDTVLALQALSEFAKMAYSNNFDIQ

VTVNSGSFNHQFSVNQNNALVLQSVELPSIPSSISVQANGAGIALVEVAVFFNVEVEVEE

PSFDVTVKLLKEDINSITVQTCTKWLLNGTSGMAIQELGVPSGFDADLDTLTKLKTLKRV

ETPNKKVVLYFDEIGTTPVCLSVTLQRTALVAKSQPVPVRVYDYYEPTNQVTAFYQSQLL

KNSTICRVCAECENCAVKAGR*KHR*PSHTADHITYRNIKNKEYILNILSFTFV**NFX

>**comp321824_c0_seq1_5** len=364 path=[342:0-363]

VKKTRTQLTAAIGDPRLYDNSHFYLATYSSPLQTKHRYNELWVVRK*KHSASGPQCSYDP

SHNRLLMLLGRCFREHIFMQMIVIEGLTLCRHLLCVVS*IVTVHVMLDSGFGGKDIFFLT

IX

>**comp73368_c0_seq1_5** len=577 path=[1:0-139 141:140-145 1070:146-188 151:189-217 180:218-259 222:260-308 271:309-348 1224:349-388 643:389-576]

RRPTDHCALCCLIPDPDKRQTCKDMYCNSGTSCDR*KKLEAP*FDIHPQLIHMFIWSTVV

SGGAVIIAGVKL*VKVCSYNCRCKVVSEGEQL*LQV*SCTWRREL*FHV*SCTWMCEI*F

HVYSCTWRCGL*LHV*SCTWRCEL*LQV*GCTWRCIGNCWDWCGAVQI*LPMSSWRYTCT

ANQSPCCLRR*FX

>**comp64512_c0_seq1_1** len=973 path=[1:0-376 378:377-715 717:716-972]

HSATATSGNIKCVLICIVYKHHRHS*QHIVEIFRAAL*DCWFVNFNDSYFRRMSFTNAVS

LLLCFLSIVEIPGQGIGNFDFGQGLILSDHGDIGGVHFTDGLLGVADMLPQRNVMIRSSG

NAYTSPAGRIMLSAPRPTTRTIRVSRTPTRSYPESAGRTRMIEYSGNSRDGVGGLDLPGL

LQRMRSLPGQSSFSWTIRRLPQRTTQRRQQVRRQRIPDVFQSFGSQFGLNGMDIINQNPR

STIQPRVNSGRSVIPALPPTSGTGGASSEYGLTQGHIQELLTAHNNARLELQAADMHLMR

WDKQLASEAHSWIQRCVMEHQGVRX

>**comp228173_c0_seq1_5** len=485 path=[463:0-484]

LAMVGGSIMNINSLIEYGINGWYNEKHSWQYGDEFSPATGHYTQMIWSTTTNVGCAAKRC

PDKYLLACFYSPPGNVRGFNPYTKGQPCTKCRGGCQSNLCV*TPINIAATDVSMDLKRHN

ILQR*FMAPTGPQASRKHLTGQPASSFHGYLYGNAHMKRSNX

>**comp24992_c0_seq1_5** len=261 path=[239:0-260]

DADCDGDQKCCGGCPRECTPPAPPIEKPGTCPRLPIATPYGYRQGYCKQQHQCNKDFDCS

GKFKCCGSCPRRCVYPFPYLTSG*SAX

>**comp24992_c1_seq1_1** len=263 path=[477:0-262]

SNMAGSSCILAIPFFAAVVFASIPKEPVNPCAAVHCKVGTVCKVNCVRAPCSGIPRCVPT

NKPGSCPVPKPGQFGICIALCSTDADCX

>**comp99065_c0_seq1_4** len=2210 path=[1:0-324 326:325-450 452:451-460 462:461-530 532:531-594 596:595-630 632:631-644 646:645-679 4616:680-720 722:721-755 4398:756-841 843:842-891 893:892-1008 1010:1009-1027 1029:1028-2068 6394:2069-2078 6405:2079-2209]

SCKVS*TALDISFYTSVTDAAACLGS*GINMKTFFSLMVLAFASQANGQAAGQVVSVGGS

ADKVCTDCINFFTDVSNIVSSANTTAEIEQLLDQLVCSKFGGLEEMCKQLMESYVPQMLK

YLAQEDNPNQICGLLGMCTQEIQERSHPVANKAVSDVVECDLCKEVVTKLRALDRDQTTQ

TELKQFVKDNICPYLGSAQKQCVQDVETYAAILFQLLANELDPTVVCEYLKLCTQAKTVP

LTRIGLASYQTKVKVMGAPKKVKVSVECTICEFALTEVDKILGNNRSEAAVEEALDKICN

MLPSSVSDECVNLVNTYGDVIIKLLVQNLKPDEICKAIGLCAQGVRAVKPKVSADVGCIV

CEFVMTTVDSLISANSSQAEVEAALEKVCTLLPATISAECSQFVEEYGPAVINLLVNKLD

PKSICTTLRLCTSSKGVKVSKSPSFTPKTKVSAGPGCVICEVVLHEVDTLLGNKITQDAI

EAVLDKVCSLLPSSVSQECTDFVNTYTPAVIQLLVQELDASLVCSVLGLCSQRRNKIVPL

KVNDEICDLCVTVMNYADALLKENATVQDAEMVLDKVCNFLSDQMTNQCLAFVDQYAPLV

LDLLASDIDPRQICKTIGLCPTSQKGVQNKLHKLLGQGKCSYGPSFWCASKDNAIQCNAM

EHCQKHVWN*TQARIRNTAKSEIQAQKILHT*ILLFCIKLTIA*HWMLYTVVFPRTCGNW

TNPGDY*APRYILLLH

>**comp100001_c0_seq2_1** len=4276 path=[1:0-167 169:168-182 184:183-477 479:478-674 676:675-2699 2701:2700-3395 3397:3396-3406 3408:3407-4049 10984:4050-4091 4093:4092-4275]

EMSQSDVNSEIVHSGS*PGMRARPKRSLCGDRYNVTQLKRFTKNRQFPCSREERVLIHVA

*PPSKQKPANEWAMIKPCQKEN*SVRGAWITVSGEYLFSR*AFYTTKQ*MNPQSEQSSMI

*ITSTYWVADSQWGLTLSPCKIPLQAVSMAGVTCRG*LVGGGTTLTVLVDPHSDSEGLEG

EVEARGGEKD*VQARYRVPQTPCVSLIAWCTTCFHSRSTTRGSWPVMMELYVMWWESCVV

QEVSGARLRATWSK*FYEISCSSSRVSVIVVSDSVEISVQDL*SKTCTDYNLINMHHQGL

AGQVVVISVIMTLTLGETTLPSPTPIPTPTDKLEETVITSILKIVDEKFETLSSRIAQME

RALNGLQFFNIRQFRGVTNTLQTTAALTQAMHSKLGANDLETRTLKSTVTRMGHDISSLK

ETNSKNFQQLERSIGYINRNIEKKTNELMTALDGLDAGKRGTEEETIEQAYTGRQETVLN

CSVDIKVLEEYIDLRFNHLRNQSHFQYENLRNQSNVQFDKLKYLSQSHYGGLRSQSESHT

NKLKHQSAIQFDKLKLQSETNYERLTNQSQVYFNSLKFQAQSQFNLISNVSQSRTVEKSQ

ETYSRGDDVFIDNEMLFHAITNMTSHVVQSVSFIRHTDSMLEQIISNTETLAYEQGKLRK

DVVEVVLQSPRGSEQTDPPNNLLPTGESQTNVGQGRLRTEGAPGCTLPESVLKDIAKFSK

NGSQLFEILTDLAQMSQVSLSSSLVELQDELLQMEETRARLEKETKLPPSYGSGDANLAL

QKILNTSKATLKMVEAMASNTGWIPYIFHNIQYVEGQLNRSMSVTTRFLTELRGLSSKLN

RPDPNAQTQAGLSNNVPSESEGSKRNGSAINQGDVLDFIYDTSVKLKRIMPALTRLIAEP

EPLITLVDGQNVNEGRVEIYHKGQWGTICNTSLGHTDASAICRHLGYLGGIAAGRGQFGS

GSGINWGFNVTCLRTFQCPAISLDTEAKSCSHHGDFAVICDHMLRLVPFEESSDVNTGHV

EIYHSSRWMPICSHGFGRNEAQVACEQMGYTAGWMRNSRDRSRNSRSLWMTNVQCRGTEI

RLDACRHNNFGARQCPGLKPAAVTCE*VETGSINGRYACAEDTSMHWSKTSYYLT*SVRT

VLKC*FQLLEVGFGRWNVMSDARTGR*HCTTWHTINSFSSTFASHMSTVLTLDYPNGRWI

NECHV*TRHLHCALTPMGNG*LWIHILGISRLFIFAAVRV*RNISQDAAGLLLLSLGKYI

IKQHYLKYLNFS*ITRFL*I*SSITIAI*KNKLF*AGYQLFRFPCP*FIIRKTVIRK*PG

KIGNTRPLSGELSLQ*IDH*VCFKFMSINMTMYLAIPILITRMAGFLVCL*KETICVGSN

FTSALC*TVYFSECVGPSRVEHKCFLPHLALKDNITCTMKYYQFCX

>**comp100163_c2_seq1_3** len=3661 path=[6689:0-1864 8554:1865-2809 11971:2810-2817 11979:2818-2821 9511:2822-3036 9726:3037-3044 9734:3045-3326 10016:3327-3660]

RMNCWTFER*QHGTGVRRSGNWCIE*VVEIHDREIESPAGMHQLWLNFAVSVLYLATSSA

QSGPSLHELGLQTDPGAAEPFKGISKWIKVLFDKGVSFQALFRDPFGVERNHDPMAEHCR

YGIIYDESMVCRIQSMYPACTIKQIRTSTDMGFAASIYKETTLPYTRIHSHDPPPFKDPI

PEIIQTCKDPKNADSAKCQIVTIVEVREKILTQVVNYGKARLLHLSRYLTQSWYRHPKLR

SVCRGRSDELVNYVKELSSIKTLKDKIVPLLDTNLWPINVTAPFRTEVDDVCKTSPDDRF

CMYKKVFLLSEPIREVKKLADKYKWESDVVQAAIAAFSMHLQDFKKNSPNEFKNIPPLSE

KTLYMENTRQIFAIIQKADFAELNDLMSIYSRFEATYFADKSLSGTTEQVLLYNLHKLVE

QARLIVDRYQETLKTRKVLTTEVDLKALLISTEMNKIIDFVKGQTSDLLNIAKYLTAQIS

QSVANQFEGLQTYFTKVESFNRDKSMADIKYVKGMLDKFTKSSKKLQGTVNDNIDKVVKY

ALKAAQAQVGEQAAMLALRVAEACNPMDWLINGGSASEIMETAALLANSIADLAVVEKLK

SSVSSLIKETKALSVGFAKNNDFLKIIRRVIDNIDSPYSSFEADKTFFLTKYADYQPGIT

RPQLVGVEVFWGVVVDEACKVIENSEASASAHFKREVGKSGICWKTKIEIAKMIETFSEI

YDFQFDLIEDMASYMRAQTAVNAAQSMNANFEAITAKAGTAEDRLMVMEQMSAILVVTHK

VHTWVIVDQYCNILEYKSGGQRPTVCKGVKSEIPALLAQPEMACDNEVRLYVNIPTVPAK

SSDKSYVNISDLYAGNEITFKIENQQWLRDNGWIGVNDNIIASYVKAFEVYAPTKTTSYR

RVKSTVIGLLNNELIPGQTEYVIVPQHPMVFEYQEGLLLQNCRKGKLQNPYTTCTNDKIS

YICPLSNDLTTCEELKTKTLLFPSVFSTWKIQLQGFKFSPVPDPATELPIKVGILMCTMY

NKKTKEQTTRRNIRDTSSQHSVSERSGSSLGCDKSCCPEGQYLSQKTFTCKPCPAGSVSA

LGGYYCQKAPAE*VRSMTVRCVALMARGLFFVNAL*SF*FHTNTTIDSVTLLMS*KVCLI

RSASVYHYLMKQCTQPFALLYWCLRICAIMTCRVKRVTFYTYLLSSF*VKVPAYVSSRQY

VKAIFSYSKLTYVIRYVITG

>**comp128433_c0_seq1_5** len=539 path=[1:0-538]

DKRANNQMRQLRIQKLCLNICVGESGDRLTRAAKVLEQLTGQQPVFSKARYTVRSFGIRR

NEKIAVHCTVRGAKAEEILERGLKVREYELRKANFSETGNFGFGIQEHIDLGIKYDPNIG

IYGMDFYIVLGRPGYNVSLRRRRKSKIGIKHKVTKEEAMKWFQQKFDGIILPGK*N*TEX

>**comp101142_c0_seq1_1** len=2799 path=[2777:0-207 2985:208-218 7751:219-362 3140:363-372 3150:373-648 3426:649-1106 3884:1107-1128 3906:1129-1158 3936:1159-1171 3949:1172-1385 4163:1386-1768 4546:1769-1826 4604:1827-2314 8265:2315-2334 5112:2335-2350 5128:2351-2357 5135:2358-2422 5730:2423-2440 5218:2441-2501 5869:2502-2535 5313:2536-2798]

PQA*TSLSNTSVVSPLRDLRPALTAILG*KRVACTCGPGISHTASRKQDVMAGRELYICV

CLAITMTLTSCDPVDQGELHVIHKRQAPGERPSCQTGATVPATRVDTTSRLQAIRSLFPN

VAGGTVHAYIVPSTDAHQSEYPADYDLRRGYISGFGGSAGTAVILTNKAALWTDGRYHLE

ADENLDCNWTLMKQGIPGVPSITDWLVDQLKTTAGARVGASPFLMNARTWKSYSEKFAKS

GISMVEVDNDLVDQVWTSGRPAQPNSPINALPMEYAGKSWDSKIGDVRGKMRAKGAAAYI

VTSLDETAWLFNLRASDIAYNPFFISYAIVESNRVRLYILNHAAKLTQDPTDEATTSKLY

QHLSTAMDGSCTSSSGPCVEVLGYSPIGIKDAVTDIASNSSNNIWVSLLCNQAIYSAIPE

AQRTHDKSAIALMKSKKNIVERDGMQASHNRDAVALINFLERLEREVKAGQQWTEVKAAM

ELKKERLKQDLNRGLSFPTIAGSGSNGAIIHYNPSNATDKQITTSDMFLLDSGGQYLDGT

TDVTRTLHFGQPSDYEKECYTRVLMGHIDLALLKWPKGLYGREIDAIARAPLWEAGLRYL

HGTGHGIGAYLSVHEGPGRISLSHSLNPSDQALEEHMYFSDEPGYYEDGKFGIRLETIVT

IEFAQTAHPFPNSVFLGFKPVTLVPYEPHLIKFDMLGPKQIKWLNDYHKRVEEEIGPKLS

AAALQWVKARTMPISYQTTNSATTYTHSVLFMMGSAVVSLMSL**IHSPLVALVN*NI*K

NPNNSHSYYACHFLGIVERSEPLFFIHILISPVV*TSKIEFKFRFQNIF*IISSLLFYSQ

ATVTNSNSYLTTFVY*QSTALCIS*PYIWFGLYEDALHCLHTNNETSDVCLVVMEYLDMV

ENFLMFYLQTRLLMTVF*YTSALPAFLALTILM

>**comp101187_c0_seq1_6** len=2185 path=[44:0-71 116:72-118 163:119-148 193:149-151 196:152-835 880:836-845 890:846-968 6837:969-970 6839:971-1027 1072:1028-1049 1094:1050-1339 6963:1340-1341 6965:1342-1642 1687:1643-1703 1748:1704-1706 1751:1707-1727 1772:1728-1858 7142:1859-1885 4798:1886-1900 1945:1901-1962 7189:1963-2184]

EQTEIYID**EMMRLYLILVFVSQTCGDIYLHNMRGSNNRLGEKSATRTNDKRMFDSQNN

NRGGYNVGDATDAPYGTDEGKQYRMKYFQSGPVKSLQNGAGESRLVLEWTNQHGCGGNED

DSPQKQNCALILQYLCQDDVDTPTGSADTLRNGVKTQTQDYKKPDSLTESKSDSQTRKDN

AVKSDRGLHESWAWYDKCYLRERNKGLFTADQKLKTNNGLGYSSATYTRQNPQGGRYGYE

CPEERDYFPYWHPTPWKDIAVMAENASMCSYYQTLSFNVQPKSECVETYPDNTRRHFSRW

NNQAECEANGGQWVELHNYLEKAPQFTSESACRNARSAGLTYIWAVPYDAEVVTVKQCLV

QLSRPDCSEAPWSRSNHLGNGVDGEPLNYTWVLPHFPSGKTKRCVFRIRYNISTDDYDPY

HTDSKYNGNMSPVKGNPYVDIGVGRSPLRLNINTNQYGRIFQDRSHVFLLRPRPAGMQND

IIHNLNVRGKRGNIVQVYPAVEYDFVPNNLRMKTGELVHIQWTGSNTHKNGPNGGDGQTG

DDGEGKTATDRNNLVEIFDRNDNFPIPFEKTTMWTGVDIKWIYHGKTSVSSKNLAVNMAS

SGYYRCIKTADCSEAAHRDYTVESRDKLHNQLDNAPASYEGAVVKFRQGTYHYMCTRNNN

FTNRSQKGTIIVS*RQLYQSSSFTSIIGDIMVCCIMFTHSASS*GRGLN*P*NAVPS*LL

NKTGISWS

>**comp101219_c0_seq2_6** len=2292 path=[7651:0-502 8154:503-515 8167:516-818 8470:819-999 8651:1000-1211 8863:1212-1476 18212:1477-1790 18527:1791-1917 1931:1918-1977 17898:1978-2291]

RTWPPSRRKKKDGEILCDFVTLLSLRCRSSICVFY*HFYWSREAI*WHRWP*RGFGNIKT

FESLS*KTEK*SLGLSVQAQLCCLLANTES*NWR*LTEFRCY*GVPHAQQLGGELSERI*

MVAHAGGQEEESKHQAIWTAMELSWLDRKWNPEPIPEP*CPCQLHPEWIQGARDVHSLTI

DYIGIWNERSYNTTYIKLLRKVLDDTGFSHVRIVAADGLWDVAVDILGDQDLAAAVDYIG

VHYPGTETKMAALQTGKQLWSSEDYSSFNNNIGGGCWARILNQNYVNGFMTSTIAWNLID

SYYEGLQWDRDSLMTARQPWSGHYIVESPIWMSGHTTQFTEIGWRYLKHGSGVGLLDKGG

SYVSLVSPDGKDLSIVIETMTHNHSQCIRPFLPPYTVTPQTITLELGGNFRSVTKLYVWY

SKLGFNDTKAPSVFFKSLPPVQVTGGTVSLKVGLDEIYTLTTLTTGAKGDYGTPPQYRTF

LLPYTEDFESYENNAEPLLMAQQNGAFEVVHIDTTVGNVMRQMVLETPIAWCAGYEEYNT

TMNLLGAITWSNIYIDTEVLVSMENGTSGIYVAARINQTGCDTLTSGGIFFFLFPVNQSY

IVSNDIGRISIINQGPQTLSSQYGWNQVSLSVQGRQAVGLVNGELLFNITIPVQPSQGFA

AVGTSDYGIADFDNLQLARSPEEAVQGKSNRKNGHRDGSHDDTLYFKPGRR*EYTLLYVK

RP*SDQNSPSIIFRVYIMINLHSYPGCIHGSAR*YYYLPLLAPX

>**comp101254_c0_seq1_3** len=2326 path=[1:0-118 6771:119-257 6801:258-276 278:277-301 6835:302-1057 1059:1058-1060 1062:1061-2097 7204:2098-2118 2120:2119-2174 7242:2175-2177 7245:2178-2192 7260:2193-2296 7349:2297-2303 7337:2304-2315 4903:2316-2325]

F*RFPEDTRLLYTNPRWCRNMEKVRGLVYIQAVCQTTVIRQAASLHDCILRRHSEAERKS

TQSRPVRNTEVT*FKEITLKFVVLKRMAPKGNSPAIGIDLGTTYSCVGVFQNGKVEIIAN

DQGNRTTPSYVAFTDTERLIGDAAKNQVALNPQNTVFDAKRLIGRQFEDSAVQKDMKHWP

FRVVKSSGSKPKLQVEYKGEKKTFAPEEVSSMVLSKMKETAEAYLGQQVTDAVITVPAYF

NDSQRQATKDAGAIAGLNVLRIINEPTAAALAYGLDKNLKGEKNVLIFDLGGGTFDVSVL

TIDEGSMFEVRSTAGDTHLGGEDFDNRLVDHFLQEFKRKTRKDISNNTRAMRRLHTACER

AKRTLSSSTEASIEIDSLYEGVDFYSKISRARFEELCSDLFRSTLEPVEKALRDAKLDKS

RIHDVVLVGGSTRIPKIQKLLQNFMNGKDLNKSINPDEAVAYGAAVQAAILSGDSSDAIK

DVLLVDVAPLSLGIETAGGVMTKLIDRNTRIPTKASQIFTTYSDNQPGVSIQVFEGERAL

TKDNNILGKFELTGIPPAPRGVPQIEVTFNIDANGILNVSAVDKSTGKSNNVTISNDQSR

LSKADIDRMVSEAEKYKEEDEKHRERIAARNHLENYVFSVRASVDEMGSKLEDVDKQTVS

KACEETLRWLDNNSLAEKEEFEQQYKQLEKLCSPVMSKLHSQGSGQQQGGSNKGPSVEEM

D*THTVRTDVTCIEIRLVKCVCNIVWMLMDT*QIYHFVNCVIYFE*TVDGEVFTX

>**comp101372_c1_seq27_5** len=1598 path=[21696:0-24 2331:25-46 2352:47-98 2404:99-264 25116:265-335 2641:336-803 3109:804-828 3134:829-869 3259:870-1597]

KAGQYIGKVDDGSPAQAAGLKDGDRIVEVNGVNIGNENHQQVVSRVKAGGEETRMLVVDN

ETDQHYKDERKVVRGDLPEVVVIHCPPHDTEPEQAPPSYTEVRDEEPVTEVVTNTETSFT

TSTSGSSSEFTARLCHIKKRADFNGYGFNLHAERDKPGQFIGKIDEDSPAERADLREGDR

IVAVNGTNIESETHQRVIELVKSGGDETTLLVVDKASDEYFKSSGITVTQDMSEVRRVSS

VPEAVQEQVEATQAAEVEAELPPPGKCG**HRRRGCHCC*WCRAHSAGR*SK*HIPICQA

WLRFKIVYLFFLLFKSNIFHNVMCVIYSNWKHG*FVELPMCRICSRFISNIPKLRKLLNV

ACTGSSEPT**SSVCYFSPNTVITSDCAFNSTQSRLFTSFILMIFVCSLESERQLATSSE

TRKGGVKAFPRVNCFEKCWKYPFSDFIFNK*DVLVVGMCASVD*ADMLLFLH*L*LFNCL

FLLSVCY**ASFDHCLSII*S*NGHHLVIEWASFEHHAGII*TSSRHHLIIVX

>**comp101468_c0_seq4_3** len=6153 path=[3107:0-49 3157:50-615 3723:616-625 3733:626-2191 5299:2192-4635 7743:4636-5442 8550:5443-5450 8558:5451-5482 8590:5483-5665 8773:5666-5676 8784:5677-6152]

VLCTGRSHGVVMWRIVVILLPALVTCQVWTQGQLDNFAQTLQVRWKVKDNIEFDNKYFAE

VTLTNNGSYTLSKTGQWNIYLDCIQMIEPDVLPRPEGVVLEGQGVKFTHLQGSHFRLEPT

DEFLDLPPNTSRKLKFYVQYWEVAKTSIMPNWYLMAPGLTPMVIESTVGEDLSFVDDFIK

PQQVKRYGADKYAPFTPQERYRRNHLTDPADNGEVRVIPKPLNMTVVKEQSLTINNSTWR

VYALDGLSTEAEYFKQMTNIDLGGADVGNDVIRMRIGNVDVPEGGIPNSPDAYRLTVDVG

NRVIEITGQGASGVMYGAQTLLSLMSQRGNMIYKMDITDSPRYAYRGLHLDVGRNFHLKS

EVLRLLGNMVMYKLNKLHLHLSEDEGWRLEIPGLEELTEIGSKRCFDLTEMTCLYPQLGS

GPTNSTPGSGFYTTDDYREILREANRLHIQVIPEFDMPGHSRAAVKSMEARFKILQGQGR

EEEGRKYLLVEEGDNSSYITAQMFTDNSINPCLESTYTFIDHVIREVIILHQDIQPLTVY

HYGGDEVGRGAWVNSSACKTLLGSDTVSTGQVKEYFFTRLANLTVARGLNLAAWEDGLIR

QGNEVFDRSLAENEDVYSYAWDNIWEWGTGGRAYDMANKGYKVVMAHASHLFLDHPDEPD

PEERGYYWATRYTDAKKTFSFTPENLYSLIEVRRSGATLPREDACGVNDESCPPLQRPEN

IAGIQACSWTETSRTQDQVDLRLFPRLLSLAERAWHKAPWEDDSGTEAGKAAMKEDWESF

AKQIGRYELRRLETNGIKYRLPPPGAVEEKGFLKLTTTYPGLTVQYSKDGGNTWFVANGN

VLVKPSDTLQLRTRSLNQQRFSRVVTLNGIEPLPLVDQSVIDYMAEQVTLSYSVLDNFQD

GTSTYLAEVTLKNTGSEDVVKGNWGIYYCSIFMVPDDQPELKLEQMTIKHVKGCLFKMEP

TIDFKPLKSDEVRKVKFRVQNWSVSKTDVMPNWYMWAEGLKARTIASTKGPGLEFVGYFT

TVNQWKRYDTPDLKDRYDPYTPDVRYGMVQDVTDMGSSQHPVIPAPVTMVIDDSVEMMIS

AIKTITTDPLFQSDADYLKDELSKGGHSNISVSTSAPPQRGYIQLLQRDPQVIINGKMTS

SQEAYTLTVDPVNEVIQIWSNSSEGIFYGIQTLLSLALKYSSDGKLYKSTIEDAPRFAYR

GMHLDVGRNFHGKESVMKLLDAMAMYKLNRFHFHLTEDEGWRVEIPGLEELTQVGGKRCH

DIDEEKCVISQLGSGPDASTSGSGFYTVEDYKDILRYAKARHIEVIPEIDSPGHAHAAIF

AMQARRQRLLDIGDLDNADTYRLVDVDDPSDYLSVQMFKDNAINPCIDSTYNFIKYIVDM

FVDFHKDISPLKTFHFGGDEVARGAWMESPACQALLRANVSLKEHFTRKVANITSLKNLN

LGGWEDGLMESVFEPFNKELLANEDVLAYAWDNVWEWGVSTRAYRLANAGYKVVLAHATH

LYFDHPYEPDPEERGFYWATRFTDTWKTFGYMPDTVYENVDVRRNGDPMTKEELCGKDGS

QCTPLTKPENVVGMQGHLWSETVRTQDNLFYMVFPRLIALAERAWHKAPWEDIMEAETRN

SEKRKDWIKFANSLGYQELPVLDKLNITYRVPPPGARNSNVRLSLKSPFPGLALETSTDG

GNSWQTASAEITAKQGETVLVTLKSADGKRRGRAVTVRVPGSNTVTGSGAGSRLQTTSIA

LLVYILQLFYRHMEL*VTKCEYCVGCLKYSALLPIYKETNYTSFADRKLYKSPTTPGLRN

KNSWNYISL*MVCVSFYESALVKEGGGCEAHFWCTQP**FWIISKVA*K*THSLTPDNEM

HSCAHYAENV*IFTSRKVRMACFLNTKVQLTFLFRLLE*LLIVTYSYVSILTIYLT*TEC

LRITET*MSINVLYVQTIGAGWLDYTII*QSL*II*LTYWGIMFC*LDCITVQYPHNVYC

TLQY*FFTAFVV*GHT*QHQWQHKAYKVWC*GTMQFEIERIV*FDYVYLTTFHSSKPVAA

LMLLAYC*KQX

>**comp101468_c1_seq1_3** len=1576 path=[1554:0-1575]

SNSPPSTSLLQLKYNQSLKMAMSGIAVSQDCEKAHERQHKKEKDIQWTVYRIDQSGKKAE

IVVECQKEREFCANRASQQQTGDVEKRNRQLWLDFIDSLPDAESRYAVFDFVQPTSSGAF

KDAIRFVAWSPDNGSIKNKMIYSSTKDTLKKKLDVQKEVFISSKSQEEIEDSFKQLLN*L

AIVSQELKCLMPGN*GTVSCKL*NDDISFFAPDHKLIADYFVSQRQRTLTFIMNYENT*P

*STLPEQKTDLKCIHFCIL*TNTL*MTCQNKLSEVIFNHVGMNIMLEETF*CK**IFL*K

L*MSNLFKFYLNPKMKVSGHFQVVYRRNLWRTNVRMLQ*MYSVS*GLHGSVSGHLDTPAG

ETFELERGLTMTCCERNKMFCPDKELVFSLPCAHYTYPKTLILYPRG*CLF*SGMAKRIS

NRKHRR*EKL*MIISMKIVINNIVHELLR*KKVLFFSHNMQMHGLLLFNVQDAVSLYSMH

KHVTTHH*CEID*YLSDIFAQGKVSFIYV*TVDIKLKIIDYRSLX

>**comp101565_c0_seq1_5** len=4906 path=[4884:0-315 5200:316-677 5562:678-679 5564:680-934 5819:935-1335 6220:1336-1351 6236:1352-1360 6245:1361-1686 6571:1687-2756 11796:2757-2778 7663:2779-2791 7676:2792-2983 11874:2984-2994 11976:2995-3219 11911:3220-3226 8111:3227-3524 8409:3525-3541 8426:3542-3808 8693:3809-3841 8726:3842-4905]

LASTSYVEITCSCVSFLPWIAT*DKTSLNNNLGKNEVKCAR*VLIK*HKYALPQLSLFAD

QQHKQSGGRSLCSRKRTRICEFPDASCHCFPVSRSVHLG*QLRLHCLLSAVHGESRPSTV

MAWVRVVSLLLIILCCSSTCSGKDRVYYIACSEEVWDYAPGGEDLVRYGQESSASVFLNR

SSNRIGHMYKKAIYREYTDSTFTIEKPKPVWLGSMGPVLKGERGDSIIIHFRNNASRTYS

MHPHGVHYEKAHEGALYADGVHGQPSATDIVPPGQSHIYNWTVGVESSPAEGDPDCLTWL

YHSHVSSVEDISSGLIGFLLTCRNGTLLKWEESPLYAIYVSVFDENLSWYLDDNIRSYTT

GVVDKGDEDFLESNKMHAINGYVYGHLPGVKACVGETIHWHMGALGTEIDVHALAVSGHS

FQYFNHRKDSIRLTVAQFATADMTALARGKWLFRCQVSDHISAGMEAFLTVDNCNAIPRV

DIRVDNIRRYYIAAEEVMWDYAPSGRNRYDGGELTEEGSDSEVFFKKDSSHIGGVYKKAL

FKEYTGYMFNQEKPQDASETHLGFLGPVIRAEVGDLVKVVFYNKASRAYSIHPHGVQHSK

LSEGSLYNDGIYGKYDDYVEPMSIHTYYWTIPEEMGPTPEDPPCLTRMYTSSVDVIKDAY

SGLVGPMLICRKGSLDENGNQINVTKDFFLMFSVSDENKSWYLDENRRISGVDPSTDKDG

DEFVESNLMHGINGRLYGNLEGLDMCVGGTVRWHVFSVGTEVDLHTLTFNGDNFMNLNNQ

KSASDVLPGDMKTLDMIPKHKGSWGLVCRTNDHYTAGTKAIYNVMRCGEAADPETSLPAE

GQVVRKFIQVMEVPWDYIPDGKDAIYNEPFTVEGSHGATFVNEGDTRIGGTYIKAQYQEY

TDDTFSIPVVRGVDEEYLGLLGPVIRLEVGDVLDIVFKNGATKQNYTIRAHGLHFNGDDG

TFSASDQPSVPPMGVRRFTWNVTESDGPGPSDPECVARVYFSDVNPVKDVNSGLVGPLVI

CRKGALELVRGKRIRKNVDADFALYFSVIDENESWYLDQNILNFAGNASAVKKDDEDFIE

SNLMHAINGYLYGNQKPPKMMEGQTVDWYLFALGTEVDMHSVHFHGNDITIFEDKPHSND

VAQLFPSVFTSVRMHAVKVGKWLYHCHIHDHIHAGMMGVYEVEKDYWNSVGTS**CG*GE

SVRDCGFKGLS*LQGLFEGVGMGSES*TAF*KYSQPYQILCLTKCEIISSC*PNIGS*GG

QFHSSFRLNVPHH*SMVRFSYSHTV*LIALQVRAL*GRQSVTSYLNLKSHNAEVKL*Y*E

NNLFSINKPTENMEHSSTVLCFKYSFQEALEAYVKYLCKCVL*PILKQTEYKLSIIPESV

AQPHRRSGFGSHMGAMCDALFWRPHYYSTITSMQLQ*IV*V*GSCGCC*ACAV**QLIRP

MLLGFSNRSQ*PMLVVSCGLLDPGVKFHHLVVCMSK*PASSMLRVSITILSESVMYTRVS

YSLGYSELYTNTRHRSELLGNRRSSEMSLVLL**LGHLNMPGTILFRSTGSKPSVQLSVV

HLNLE*HYFVNVFVMMEIFGTTMMGVSVLNIPVSLSW*YFECSLVSLLMPMSTSLPHSCT

CTFLLRCNTYMIIYKX

>**comp101644_c0_seq2_4** len=5041 path=[1:0-618 620:619-713 715:714-1166 1168:1167-1220 1222:1221-2965 2967:2966-5040]

PQDLEEVNVTITYQVVLPTTPAPPTTTTTTTAKPKPKPDMVLEGNGTTGSGRPLPPITMV

DPDFRRPGPGYYPRYKTVVLSSNMVNFNPKTGLAEVKANIPTDAESINLQADGFNTHAYR

YASKSHSPSGSYIQLTVPDAQTRVGDNMEFQLRSTTPISDFTYQVLSRGRTVAVQDVSVP

SPSSSNVFAIRMTAEMAPNPQIVVYFQHDDGEIVADAISIKVDGTFDNNVRVEFDQRSVK

PGAAVNVKVHADPDSLVFLLGRDKSVLLLKDGNDITEEMVSRELMTYDYGSYFGWFDSWF

RGCWLPRSSGGTDAKGVFSGANLAVVTDANVYEHRDYYYDNRFRLAVDSPMSAGGFGGAE

DFGGQSNAVLEQGNFKSVERTRSNFPETWIWDMVVIGTAGHSDLSLQAPDTITTWITSAF

AVNPEKGLAVATRTANLTTARKLFLRLELPYSVVRGEEFVLQATAFNYHERDLPALIILK

QNAQFHNVFLSASRGQEKDIYSTQQSHSIDIPAGKGRAVYFVIKPMVVGDVILEVDLLSW

ESGDGVRRILRVEPEGLERTENVAMLIDLREYLTFQESTSIDFPVQTVEGSQKLRVSVAG

DILGPSLTNIENLVRMPGGCGEQNMVRFAPNIYVMKYLQRTQRLTTPIKDKIEIYMEKGY

QRELRYKHREGCYSTFGESATYSYGNRNCSMWLTAFVLKCFTQAYPLIPDVIDTNVLSQG

LQWIVHQQDEDGSFPEEGHIFNKRMQSGTGMGVGLTAYVLIALKEAQAAQVQWPSNNDVP

VDTLGGDLSRASRPDQREPASVLTDKMLLDSASKAVQYLESRLDTLNSTYDKILVAYALA

LAGSERASEAQSIVEAAAVRDVSSVHWKDPSPPKPTGYFHYRWYHDTEAKSIEMTSYVLL

LYSLTNNVTEGLPVMRWISNQRNDRGGFMSTQDTVAALEALSKFAGDVYSPQTSINVSVN

IQSYSKNFDVNPNTALLLQADDIPMANVPGVLTVTASGSGVAVLQVLKQYNENQPSINPK

FELRVEVLEQTDRQFVLKTCFRHLEEGSSGMSILNIGVPTGFDPDLNERTHTAVFSRSER

NENILTLYFNDISDSEGCINTVMTMVDVVIDTRPSTVTLQEYYEPDNAVSTMYSRKSTPM

CSVCPQCSFRCQIKTQPVM*TACVNNETQILLLSAFKPDLLPWNML*AYLHSLVWHLNPA

SSKEAHSLNALCIFHAFIKFPNNLTKQIYLLYFFQGCVLFYRAS*KKASVKKCIRLESSF

IQLQLLS*KQI*CYMYCR*HRKDCSTVVVFFRNSSVFRGIYYTYTLYEY**EEIISFL*V

C*PHIFT*VNEIHQNIKLYLCLDICYALCM*YNFRYLSIFYEAYNFMDNFLMHRATFQ*R

FLPFRWV*KVCLQLQ*YFL*TRLHCGSRTPYSLDLSLMMHCI*LCHISIFSFMLDYERTT

TLLLLLCY*NLNNN*TSGNKYILIMINIWLEPENIKFSSCFCFFCSVELFLYCAYHLIFP

KVVLLF*AGLFNCNVSFKLEFMISLDSKVFKCVIKTNSVLCFSFKK*TF*MSVLLHSEYL

IKHPCFISLLYDV*VRVNNA*GHISNFASK*QHIYFSALLVRSYFHKGST*YELECNQSQ

CVHC*TLHWASCQICGAYHYSFCCGSVNYFILS*TSISNLSIHH*EIHFVAYSLSDCGIK

>**comp101668_c0_seq7_2** len=1636 path=[5406:0-67 5663:68-68 5664:69-171 5767:172-681 836:682-720 7215:721-1201 7696:1202-1635]

RNYHRHDASATCPCFVLSRF*GKIFINNEFVSSTSGKLSSVSNPATEEKIADIEEGDKAD

VDKAVAAAKGALEFGSPWQSMDASKRGRLLDKLAELMERDKAYIASLEVINSGKTYREAF

FVDVLSSVRVLRYFAGFADKISGKTIPIDGEYFCYTRPEPVGVIGQIIPWNFPIPMMVYK

IAPALACGNTVVLKPAEQTPLSALYLASLIKEAGFPPGVVNIVPGFGPTAGAAIASHPDI

SKVCFTGSVEVGRQVMTMGAQSNLKRLNLELGGKSPIVVFEDSDMDYTIEKCHHALFFNA

GQCCMAGSRTYVQEDIYDEFVQRSVNRANTRIVGDPMDPATESGPQLDEAQFKKILAIIE

SGVSEGATLHCGGQRHGDRGYFIKPTVFSDVTEDMRVGRDEIFGPVQCIMKFKTLDEVIA

KANSSDYGIAAAIFTSDIDKIMLFSSRVKAGTVWVNCYNWLTPQTSFGGVKQSGMGRELG

ECAIREYTEVKTVTMKAPAKV*DVMDKVDSWQHLPLCVCYLYIAVYSQVFTEV*RHRVYS

**N*N

>**comp101701_c0_seq7_1** len=1047 path=[1322:0-40 1363:41-70 1393:71-122 1445:123-153 9311:154-156 1479:157-179 9330:180-202 1525:203-241 9361:242-249 1572:250-367 1690:368-387 9410:388-397 1720:398-459 1782:460-517 1840:518-558 1881:559-651 1974:652-769 2092:770-836 9581:837-856 9601:857-864 2187:865-871 9619:872-880 9628:881-891 4813:892-1046]

SCF*KWWASKRL*KMVLQW*TN*ICIFL*IFRTPKHVWSKAQTCLVSQANKALFSVNTFQ

RKMGGVNCKDLFKIFDCMISPILCYGAEVWGTSYCKLIESVQIKFCKYFFKVGESTCNNV

VLGECGRLPVQIVYMTKVIKYWCHLLYLPPNRLPKHCYKMLKSLDDVGRVTWVTNVKHIL

FTYGFGYACVSQDIGNVKYFMFLFKQRLADNLKHPWYTSIENSHTTV*YCQYKPLLDVEK

YLKCNICLNLRSALSKFRCGNFNLAIETER*EIIASNERFCLFCKSKGN*CC*R*VAFCI

LVFVNVSLMFEINIL*NI*VSL***TTLYILCNLLMQKYR*DAYLHKLR

>**comp101758_c0_seq11_2** len=3041 path=[1:0-452 454:453-456 458:457-532 18994:533-535 537:536-581 583:582-594 596:595-1354 1356:1355-1366 1368:1367-1503 1505:1504-1511 1513:1512-1734 1736:1735-2306 2308:2307-2963 2965:2964-2974 27145:2975-2987 15771:2988-3040]

CP*TMLLLLYLAPLVAGEFPFRNTSLPWGDRVNDLVGRLTLDEIKLQMSRGGSGPKAGPA

PGIPRLGIKPYSWNTECLRGDAGAGDATSFPQAIGLGASFSPDVIFRVAQATGEEVRAKF

NDYMRKGQYGDHKGISCFSPVINIVRDPRWGRNQETYGEDPFLTGVYAANFVKGLQGSDP

RYIRANAGCKHFNVHGGPENIPVSRFSFDAKVSERDWRTTFLPAFRSCVKAGTYNLMCSF

NKINGVPACVNKKLLTDILRNEWGFKGYVISDQAAIENIISEHHYINNSVDTVTACVKAG

LNLELSTNLDQPVYFSVVDAVKQGKLTEDEVRTSVKALFNVRMRLGEFDPPDMNPYSKLN

LSILQSEDHRDLSVEATLKSFVLMKNQNVLPLKASSYKKVAIVGPMANNTIQLFGNYAPD

MNPKYTTNPLQGITRLWSVVRFGAGCTDNKCIQYNSQEVQTTAKGSDLIFVCLGTGQELE

SEGNDKANLDLPGQQLQLLKDTVNAGQATAAKVILLLFNAGPLNITWAVESPNVQVIMEL

FFPAQSTGEALRRVLLAKGPNSVPAGRLPITWPKYINSVPDITNYSMVGRTYRYAKYPVL

FPFGYGLSYTRFVYEGLRYPNQVKAGEAITGSVSLGNRGSFDADEVTQVYISWCNATVTV

PQLQLVDFNRVFAPRGVQTQWNFTISAESMAVWTDEKGWVVESGVILFWVGGQQPNTKPD

VGSNVLSGKFEVAGQKVLGRY*SESNVEL*NLYI*KNIYHMFSHFTCEIYVLCVFNM*TQ

*LYDKHVLVKYLSTVMKKKR*ACPIVGYRHKQVQRHAHCPSPNRELHQAEASSLIHVILR

NLEIEG*QSIHVSHVGLHNIHFHINPTIVYFLVHTNKYELCSETCLLNVIIA*LGISD*P

ISQFLMGTSITFTCADTLRSKLLAKERDHFISNCIKKTTPKQNRWYRFEPTHKTFVKVPI

TTQQSENVIQIVHVSRKT*LRDIGPSASA*LWHKYRCWTEQA*SNKKSNTQAQX

>**comp101858_c3_seq10_6** len=2289 path=[23058:0-188 23247:189-606 23665:607-615 23674:616-720 29219:721-721 29220:722-796 369:797-827 400:828-1156 729:1157-1180 11218:1181-1819 11857:1820-1840 11878:1841-2217 22023:2218-2288]

*LADTRLSAVGCHSKLD*P*LLEKMAQLLAKPGSSGQLPRLPPRPGKTGPFNIKDEECKG

DYYLQCQQLQAAQATSHIDHMCCLDIDSEPHMVRKSGIICTIGPACREVPTLQKMINEGM

NIARLNFSHGTHEYHGGTIENVRAAASAFSQPRPIAIALDTKGPEIRTGLIKGDPSAEVA

LKTGNTIKLTTDPQFYEQCSEDVLYLDYVNITKVMKVGERVYIDDGLISVIVREIGDNFL

ICEIENGGDLGSKKGCNLPGVAVDLPAVSEKDKQDLLFGVQHKVDMVFASFIRSGAGIKE

IRSILGEEGKNIKIIAKIENHEGVKRFDEILEEVDGVMVARGDLGIEIPAEKVFLAQKMM

IGRCNRAGKPVICATQMLESMVKKPRPTRAETSDVANAVLDGADCVMLSGETAKGTYPLE

SVKMMHHICREAESAVFHNQLFEELRRETPMFTDAAHTIAIAAVEASFSSMAAAIIVITT

SGRSAYLVSAYRPRCPILAITRNAQTARQCHLYRGIFPIHYSGKSREITHSTLLNT*MF*

HLAACTGLWPGKL*QTWT*EHTSPFSSEIYLCINYNSRLYIFLQLVYETDQQQKLKLEKR

SRTLF*WVGGR*C*LCFTVRRNEIDK*LLPLIERKGT*HIFILMHKSRGHIISCHVKSCV

SS*SSATCFNFCSIFFVNQLKRNLRPNFAFHCSLFTSV*NTCLASFHIAQYLETKQSSAI

VTICLCLMKYLYRHFLSIHHVNQAHQCSQKPTHDV*GAASDFX

>**comp101949_c1_seq2_1** len=2490 path=[1:0-190 192:191-197 199:198-268 270:269-326 328:327-332 334:333-515 517:516-571 573:572-590 592:591-1038 10352:1039-1041 10355:1042-1054 10368:1055-1060 10374:1061-1069 10384:1070-1154 1156:1155-1178 10395:1179-1214 1216:1215-1216 1218:1217-1412 1414:1413-1435 1437:1436-1525 1527:1526-1588 1590:1589-1601 1603:1602-2270 2272:2271-2277 2279:2278-2489]

VRPAAR*VYTYLYVLWKLRFS***SLLISIA*QSLFCGWGRWFEYLGSTQLRGQTSLSMS

SLRCCIISRDSKADSK*LPTTR*SLSSLSIFSVVNKQHIRMTMYLVWTLLFLPGVLGGRV

GVDLTIAPGASGTAVVKAVVDRIRSNCILGDDRLFLRRLAYVETTDGTNSKTYRSGYDGG

IWQIDESKFLETKTCPLSIVAECNKIQSSFNIDWRSTSWTDLRKPLYSGLAAALYIKKYN

SGSPLGDVTSQASFWASRLRPSGSESGFITKARTMTGTDCKTQLDLAFILDGSGSVSRAD

FNRTIEFVRNVTENLTISRNEVKVSTVEYASSVGDDIKFRDFASKSSLVSAIGRIRKSGG

GTNTAAAILHTVDVLFSPAAGARPNAKRVAILLTDGRSNSKTATISAAQKAKAAGITMIS

VGIGNADVSELRGVATQPYCTHVIMLSSFREIDSLLYQLKESSCGALTAVKKNKTINMPL

PVNKTVEDSFEFKDDNLTTAVPGQDKAVVGEVVCGTLVMYASYNIPRPGPAFYDVKDTGR

DGKPAIIYFNKTMQGRQLYITVLGTRLSVSLAQMASCVNASFSVRVSQQPPKARVICKEG

VKERECSRKDLEDNGYNCDDTFYNVSNPCSPGNACDHAHPSDSSKFIRCDLKGSMYVTQC

PGKTQYNPDTISCGAEPVEGGISCNVTNPCTPANIARHRFDFPYPPNPSRYIQCDAFGKL

WMRDCPGGTVWNEDRRSCSSQSGVTTTLKPSILRTSTSSVSPVSNSCNGNNNGAMFPHTD

ASKFILCDHGAEVVMSCPPSLQFDVSINGCNHG*APHRRRFHVLYEIKCP

>**comp102265_c0_seq4_6** len=1592 path=[5117:0-180 5298:181-244 12531:245-248 12535:249-267 5424:268-391 8690:392-400 5557:401-503 5660:504-508 5665:509-1591]

RGACDERLQRDNMGCLCSCLHPEVNSEASAKTPLLSGTTSPPNNSNNNGQSARQNRPPRK

EFLTSVEELAIVQCAKLPLPALDKTFQDHSKLYNDLLESFNTLKDTIHDFKAVFEYETSG

IPVLAECLKLLRKRCGVATLTASRTRFSITVEYDRLEVSRLCKGPAEDTLEALELFNKAN

KLTRSILEKAPKVKNTIQVVLDDEQSLRREISKSNISSTDEPEVMKLCVANINKLRKLPG

SVETIQKHTQRQFNELKESSKVLFEES*LWIIFGWLLSVHTNLITIGAHF*CSQCGTK*K

LVS*KNVINIFS*RSYC*CLTVHIYVITLGNNS*CSQFGTNSEFVSWLDLMNIFS*KSY*

FCCEAHYYLDVPRMKQTENWFLDRN*AVLQTEIQS*LSE*WRLGEVGRSEVR*TYLCAIF

WDTMYRGYVHI*LRIGFCS*HECLGSISNKETML*HPTLYPTQ*PRDPVTP*YCWISARP

GSQFTRFYI*YCQMYPYYGLVMDYMIHIVFV*SKFCTH*GIQDVCK*VSEX

>**comp102412_c0_seq2_5** len=3172 path=[1:0-264 8741:265-271 273:272-360 6912:361-380 382:381-417 419:418-476 478:477-481 483:482-505 507:506-857 8892:858-952 956:953-1061 8985:1062-1083 1087:1084-1154 9026:1155-1156 9028:1157-1273 1277:1274-1286 1290:1287-1339 1343:1340-1342 1346:1343-1445 9140:1446-1461 1465:1462-1863 1867:1864-1927 1931:1928-2162 2166:2163-2283 2287:2284-2293 2297:2294-2538 2542:2539-2590 2594:2591-3171]

VKMAASFFQDVEMAPPIEVFQLTRRYNEDTFPQKVNLGVGAYRTDDGKPLVLPVVKTAEV

AIASDTQNHEYLPVVGLPAFREAAKKLILGEDSRALAENRTEGIQCLGGTGCIRLCAEFL

KKILNYDTVYVSKPTWGNHRTIFKHANFSNIKEYRYWNPKTRGLDLEGMLEDLNGAPEKS

VVILHAVAHNPTGVDPTPEQWKDIADVCEAKKLFVLMDNAYQGFASGDLDRDAYAVRYFV

ERGFELFIAQSFSKNFGLYNERTGNLCIVTKTPEVLSQVRSQCEILVRTMWSNPPNHGGR

VVATVLNNKSSYAEWKAQVKAMADRIQQMRQLMYEKLRAKGTPGTWDHIIKQIGMFCYTG

LNVDQVAYLQKQYHIYMLKEGRINMCAITSKNVDYVVDAINDAVVNIGSTQVS*WASLIQ

NRTSSCHFFFQLPRLNILSTSFTFI*CRRGESTCVA*PRAIWTMLRPRLPIQWLQSLPSC

RLVSSPVMV*Q*YIPVTVNLQ*YKCDI*QCELTILHVLTCYTDTFLSSMTNVWIDVF*QF

TDLLLFRYIFVNIIST**RELFDSWLRKKKYYMIFNIFDIFSSPPY*VPHIRNTWHGL*L

AKHSISNDGFFCHE**KFRLLQKLYCSISLFKFINFNREEPMNKDHNLSRFVITELRREY

Y*TTYSI*YNKIILIKGVQITQYKGDNCLIL*YIQTVHYHEPTVSYNRYLYRYVYFYEVH

*CNWICI*FDLLCQTNLTKHVL*IKYPQSAGQGYSQN*VELT*YIRV*IFTVIVFTTVR*

K*LQASISFILMS*HVH*MSCYEILAEFCGINLFLYISDRACGS*E*LNYEFTAISPYVS

TQSY*PE*LFFSSQRFVLVLYWYEIMKSFGTYY**SWSFDCNLTWSYSYQTMYLCSLLLL

LQLTIVISCICF*KARKKRNYVSCR*N*SLLPFPKDCDNGKSQTVTGIVNSNLKNMMIET

GRCSWGVESEILILLHL*RYVSQRVGL*L*DILYQSFCVESRNVVFNLESIW*RCDCDCS

MYGKPYVGTNVI**THITLFVKMDCYNNIYAELN*LSX

>**comp102820_c2_seq56_4** len=5503 path=[47:0-7 55:8-354 403:355-489 551:490-610 672:611-650 712:651-1103 1165:1104-1105 1167:1106-1248 1310:1249-1516 1578:1517-1525 1587:1526-2963 3025:2964-2970 3032:2971-3033 3095:3034-3349 3411:3350-3644 27196:3645-3645 27197:3646-3649 27201:3650-3651 3713:3652-3780 27261:3781-3785 3847:3786-4286 4348:4287-4293 4355:4294-4663 4725:4664-4688 4750:4689-5419 5481:5420-5502]

AAFGRRVAKLCLTILLLSTFVCSHGQKIDDSLSNDGNVHREKRAALDNNFGGNRKPYINS

NSDGKINTKFPQRNNGVGNNPQNNGVPYVNKQQQPVNQKPGDAVPRNSNFQFNPKRTGNV

DQMHKPTRKGYQIKISDSEECGAEVRKYCSQNLWDKNFMVLDCLQNDLKMETELSESCQH

FLWMYKRNLTKDERFEHASQDVCKTELQTIKECSGLQRGKGTIISCLIENFDNVTSSSCR

QFLNKMATIVFSDYRLIYHFAEDCYDDISKTGCGRSEGDQDEALHTQGRTLSCLAEKMTN

LSGKCKVQVLRTYELQSDDYHLDRPLYYACREDREHLCPTVTAGGGKVFNCLYKHVADKA

MSHECREKIELRQKLISQDVKMDKSFYKACKVDIEKNDCLAGSGIHGDTKRASVLLCLEN

AAKTGKKLKGKCVAQMEDVRKMLMEDYRLSPVLVARCGNEIKAYCNDKDIGGETLHCLMA

LAKEVTDKPKITPACTRELEDLMREADVGSDIKIDGALQRACQSVVDKLCDDVQPGDGGV

INCLMENIDSDDMTDVCEERLLEIQFFIVRDFRLDTHLYKSCRTDAKTICKAGDDWFDPH

SKQPAKDPFILPCLHQSQVSRACKQEIKRVMKSYALSVELNPQVQAKCLSDLGSYCSSNK

EDTAVGAEMECLQNNYDELTEGCQQVIGELMEEEDKDIELDHILMKACTPMIKRFCNELL

DSDADPGDVLECLIEHKNNHEMDSKCAAGVEHHQLITQKDFKFNHKFKEACQKAVSKYCN

YKKTKSDVVVCLSEHVRNDTLLDKDHRIEKQCRHQLRLQILQRGESISLDPELQGKCKDD

IKTFCEDITPGNSAVMECLKKKQSRLSPPCHRLLFKREKDEFAIADYGLLSACKRMIKDY

CDPDDDQPQILRCLKKEKNNPDFDAKCRSVVIRREIMQSKDYRLNPSLRKACHLDIGKFC

AGLVDQHKQDVELEGLVISCLKKQYAAKRLSRECDMEITEIIQQAAGDIREAPVLEKACM

SEIRVHCKDKMMEADGKDNDEIKEAGKGQVEECLKEKFRQRKLTNKNCIKEVSYLIQEEE

IDVNVDPVLKIGCQKELNRLCRGVRAGEGRMMSCLLSVLEEDPKSMSDKCRTMLHNRKEM

WEYAAQVAPIEGFQEIYTQISESPAKFYILGVLTAVVCMIFVFGIGCGRVTKRVRAEMKN

K*YQVYM*K*HLTLSLTTRSSHLVKLYH*QG**KRGRGDPQL*VLCD*LSFRFTCTFHPF

IDRYKHFHWLRECQ*KG*RDQIPYLKSSLSKRVQVILICIAVF*HI*TGLISKQLFCHNA

KLI*THFDIICIHFQSKKWI**WLIFIPAILFFLYFCS*NHLH*TINNRFNLTSASINLN

*NGNSIHFLILTYLPR*QVVVQVLVTYF*ISIIKCTFL*FKTCT*QSH*KWMD*RAEILS

SCSVSVSEKSA*KGSSE*KPQNYKKIGYFDTVLI*KNISNFNKTAFVL*FTRACIITTLP

ACQFSENIPTQYTFQS*QPTHPTATTHNLFPHHSCQLSLSLVESQ**L*LVDAYHMDLSK

YY*K**GLC*HGKHLPAANVSSFSPNTLM*Q*LSTYYFFHTYTGCNNTKLPVGR*ALQFH

VVKFSMQT*TSSFCGWIVSH**MLRYEIMFVVVIFF*LYVFWFVLGD*ECCSI*SR*NML

PKPRS*W**NSDNCFKI*YECFIKICYFYTFSPFFLYVFFISGICF*TMVVVVGLHLLIC

TAHNGNILYLNIRSIATKFSGKFLQVG*TWFEAQFTLIMFLSLSTQYTKVVNNDQHQNGY

PSNPAHSLKGISFLW*LFASSYLFS*G*PCG*SI

>**comp103009_c1_seq5_2** len=3553 path=[11089:0-35 12159:36-36 12160:37-288 659:289-412 783:413-591 962:592-605 976:606-2509 2880:2510-3297 3668:3298-3299 3670:3300-3552]

PSWPLMNSLIFSGKDLQISGEFFLACHRSLRKR*NSGHFSAANAWQNDSCNNS*VLYY*D

TDVCVCSTGILCGRGQS*PLGIELA*PEKGREQGDHPSPLLLGVFRAILFYLFFWTHTRL

LQTQTNRDLFA*LPETEIFCGVSSEWLL*CGHTSSVDLWIPCVTERLGILSK*LRNLYIF

*CQLNWNFIQYSGTCQTEQNTA*QILL*EKLLWIGSVLPVH*TLQQL*RISRIPARTLSD

LPVKPRRLCMNEMKFTQGMAMDVSITVMLWMLLALSVASGLTVYEACVAMPSGQSEIFVD

VQESATVGSVVKKMQYNASTDEVLVTGVANTYFDYNDTSQEIFLIKKLDADNGEETERLE

LKCQLATSPQPLLTIAVTMLIKDFNDHPPVFDKDNYYINVSEIEPNGTVIPTPASATDKD

ADKNSNGRIYYSIPEMYSSYFEIKNPLRTSLTLISPLDYETTPNMTIQVTAMDQPTGGAT

QMTATATLHISVIDMDDLPPAFDEPSYAGTVPELSPKGTIVNVTKPISARDQDVTINAPL

RYSIKKATNDYFKIDPNTAVLSVNNPPPAQEFSIMIEATQVDNSFRTDTTFMKIVVSAIN

SKPPVFQQQSYTANVPESLPLGSTIVTVTATDSDFGSRLRYYFAKEENVFTINGESGVMQ

LRQALDYERETGYSILVVVTDGQFNTTTTVTVSVSDVNDNNPVFTQKEITVERERVTDEL

ITTVEAVDYDYNTVLSFSLASHTSLFAINAQGELRISADKDNLTENQYDVVVIVTDNGVP

SRQSSAVVKVKFPPQSPTIGNVTMQEGGSDVVSICLGVVAATLLIIVIILTVYIVRRRLQ

STEQLDKAKNRSSLDPRGIMFKKHPGSRAPSRVAIDFANEESTDGGTTLAENPFTLDGNS

NYGYMHSDESENDRDVDEIQIETAVIPYRNVGKFSSNGPVFPNMEDEDGLPINDHSYHNS

SLSTFKDSNDSLHSDSTSSKKGLMKNMNGIGTKSLPNKKLSWAGNGEMPSNKSLNSLDHD

PGISPIDEKPEITVYF*TVMYYCV*P*PS*GTHGHVHVQFMDVRTQR*HVILYNHQIMYK

RLTVLIQDTGQLDDIPSSKCWFCCCSKLRTTDVRSL*IHADGQCWY*SQTDH*FAESCLG

SLTGLLTYSSKVSECGFMPFSAIFRNITAGDTRNGHHTLYPRGE

>**comp103103_c0_seq1_2** len=2248 path=[1:0-105 6828:106-677 679:678-699 7040:700-704 706:705-720 722:721-872 7102:873-873 4587:874-888 890:889-1082 1084:1083-1095 1097:1096-1695 1697:1696-1696 1698:1697-1758 7372:1759-1759 7373:1760-1827 7582:1828-1884 1886:1885-1897 1899:1898-1944 4664:1945-1961 1963:1962-2010 2012:2011-2043 7517:2044-2247]

*PCNLVIERAPRNKCRCGELEILSYDRHVNDLLSHEWVGPTDNHRRKRADVILINMGRLT

TIVVVVLACLHISAAYVPRFLNGRPRGGMVGRPASKRVSTNPLPPDMWMEQTLDHFNDAN

TQTWQQRYFVNEKNFKPGGPVFLMIGGEGTADPIWMVEGMWVEYAKTYNAMCFQLEHRFY

GKSHPTPDISDESLQYLSSEQALADLAAFIQFAKSKFNLADNKWISFGGSYPGSLSGWLR

MKYPHLITGAVATSAPVLAQLNFINYLAVVRDSLSTTGPGCNENILEATEEVELRLQTQP

GRDELKKLFNLCDDIDVSNANDVANLYQTLAGNFEGVVQYNKDNRAFEGGVGANITLDTL

CGLMTDASITPIERYANVNNLLMTTYKQKCVDFVYSKMIDDLKKVDWNSSASEGGRQWTY

QTCTEFGFFQSSDLDNQPFGHGFPVSFAVQQCMDIFGSQFNADLIQHGINRTNTNYGGYG

IKITKVVFPNGSIDPWHALGITSDLGPDATAVFINGTAHCANMYPPRDSDLPELVQARAT

IQKLIGQWLE*TPIRK*CYTFLQSSCSNLTF*L*MYNLILLNNGSLKHYSFSKRDTFVPY

FCQDIEY*F*T*SRFPGKNILVDPNQSMFVIRGQSHDLADCMSLQI*LFGLMTISHRIAL

SILNRSQTAVVSLAFSRVWHYATETNKQYFYHW*IFAIPDQTLHQVNECFSLAVIVLFFN

QV**RSFKDV**MYYFCTVF**IFICFKK

>**comp103134_c0_seq6_1** len=1824 path=[5760:0-44 5805:45-140 8523:141-144 7983:145-154 206:155-592 4054:593-593 4055:594-617 669:618-835 887:836-1006 8807:1007-1075 1127:1076-1086 1138:1087-1192 8868:1193-1217 1269:1218-1373 1425:1374-1429 1481:1430-1463 1515:1464-1466 1518:1467-1789 1841:1790-1797 1849:1798-1799 1851:1800-1823]

GCCRMESN*VDHSPLHQARVRKWHSGLYQYITSMSDSNS*STDDAHQVTSPGTVSCQRVL

EVTGTTNMKLVVCGVVLPALILATQGILEHRTIYPQPEQIHLSYGAAPDQMVVTWVTLDK

AGTATVNYGEGKLDQTQYGTTTVFTDGGTERRVLYIHRVTLSGLNLGHQYTYRCGSDAGY

SSLLTFRAMQGGSDWSPRLAVFGDLGNENPRALPYLQEEAEAGHFDAILHVGDFAYDFDT

DNARLGDEFMRQIEPVAAMVPYMVCVGNHEHAYNFSNYRNRFTMPGGDGEGMYFSWNIGP

AHIISFSTEVYYYGVSTESIRAQYEWLEKDLQEANLPANRAARPWIIVMGHKPMYCSNSD

KGELCDNDDNPIRNGNKYYPASLEELFYKYGVDLEFFAHEHSYERLWPVYKTKICNGSID

TPYVNPQAPVHVVTGSAGDREGQTKFLHTVLPWTAFHTDDYGYTRVTVQNATHLYMEEMS

VDKEGAVIDKVWIVRDSHAAGQFNCQAVPT*RKRTTIQSYLPSRKK*LANQQATENLILF

YHVI*CTLYIYILQLS***TLFNNQ*TINISKLLII*IENIVVNTNIPGKSM*L*NCST*

W*I*TGPP

>**comp103210_c0_seq2_1** len=2310 path=[1:0-38 40:39-105 107:106-710 712:711-717 719:718-766 768:767-779 781:780-910 912:911-914 916:915-1042 1044:1043-1057 1059:1058-1092 7826:1093-1112 6228:1113-1114 1116:1115-1132 1134:1133-1241 7869:1242-1266 1268:1267-1306 1308:1307-1364 7922:1365-1392 7950:1393-1467 1469:1468-1512 8019:1513-1523 8030:1524-1530 1532:1531-1578 1580:1579-1620 5640:1621-1642 1651:1643-1679 1688:1680-1694 1703:1695-1746 1755:1747-1747 1756:1748-1771 1780:1772-1797 4968:1798-1820 1840:1821-1878 5154:1879-1925 1945:1926-1983 8232:1984-1984 8233:1985-1989 2009:1990-1991 8240:1992-2007 8257:2008-2029 8279:2030-2032 8282:2033-2047 2067:2048-2053 2073:2054-2096 6578:2097-2105 2125:2106-2253 2273:2254-2309]

PSCSNDIVLHVQRQQGAVFKSHCAHAIRKLERVFPAGDGTYMRSRSSSCPDVIREVS*FG

VCLD*QKMSLVVVSLLVALSSSGYAAPAAQCACASVDTVIERTYGSKDNTFGTLRSPNCL

PYQGDQAAINDGRIYAHVLYQGQGAWLLTSDIYIKNCQESFDCVCATATVDVLTGYGTGS

ATIATLTPGQCVTLQGKTYNSTGGTWVQVSVNNKIGWIKKGNVVFHKNCGGHANPGSSAV

QLPGCPQIITRAEWGARAPRTPHVKLSHTPYYAFIHHGATAGCHTRGECVRMIQSYQNYH

MDGHGWSDIGYSFVIGEDGNVYEARGWDAVGAHTYNYNSIGLGFCVIGNFMQHLPDQAAR

DVLQKLIDCGVCNGKLTSTYTMLGHRDVGAKSGGTSCPGDTFYALIQKWPHYNAPVVPST

K*KRFMILLMKHF*FICTHFKECLAF*RLRLVHPVRFVHHNYFKQ*KTTIF*K*QV*RLY

G*ALFMGEKEGETPSAPCLHITSTTGTFIALAPKYDLFYWQGLCGYF*NININCHINIRL

YPLFFQYNHKQIDQQILLI*V*FEQFHIRFSTK*NFNFGNNLPFNENVNQFRKIWLP*QF

N*TECQMMIVVIV*STTSDHISTWCSH*LMLRKAPFLPSCILGMSESCHVKKSYFLLLSS

FSSMSFTY*NNENYTPMFFGAFAASEHSTHSFAIMCEPLNVLGCDRLME*TFPLCSSGVK

RIIYHISGNI*ALVIYENMIDK**PESSRMIFDPTSWKPRQKVSVTTQSS

>**comp103248_c0_seq6_5** len=3269 path=[1:0-530 533:531-620 623:621-622 625:623-680 683:681-682 685:683-827 830:828-1784 1814:1785-1918 1948:1919-1935 1965:1936-2023 2053:2024-2108 14197:2109-2185 2215:2186-2241 2271:2242-2247 2277:2248-2329 2359:2330-2421 2451:2422-2427 2457:2428-3008 3038:3009-3115 3145:3116-3235 14602:3236-3236 3266:3237-3268]

EKMAEARLPNAVTSEELDLKTVRVVLVVEGLEVGPLGGKKNAEVIVDFKETSFCVQAEVR

RKKGVVKYKQVIKKLPGEIDPDKCIVKYKKDQIILDLKKKEESSWAVQLSTSGLEQASSD

ESD*HVLCLSHCQMCCIWSMSVTYAVNLVAHMFQLMHNVKRNFTSPHPSVSMIIRQPF*M

*HFCMSVTE**EYVPKVK*HGIRALRLICSFDVCLTYYVSMSCFGSMSYDKTVYTNQS*L

DSFHLRFTSRTCICI*KQGKLEHVLKVHVKHIYMLS*KDNKYYLVMWYC*FISSVQVRTT

NL*SIRA*GRWRLKSVCFPVGSICLIGLLDSSLNCIVMKLLGSRR*GSICACLLIKYIKN

HEISKLKLSVLMQ*KHILHFLVH*CIADLFTEIINIKSKYTSRYDYEVQMRYLYYMAYL*

FGVSALIVFKD*G*FVCYNWQVYLLIQNSSFCKVFSVARDYICSI*TTVI*M*QLGYCLI

FIIQIC*ISIRIVILRMKPVLFLYVK*LRRD**HISYTCFNRCSIFHTFLLTTICFPCDV

QLYDVTITAHKE*NVEMRKSFQFKSPYATMF*VWYVHLHCSDCQQDLCNPQNMLNQQTFI

QSYIQVISSKRYHTLPHLVIAKTVHTSIESIK*TPPPPPQKKDF*FFKS*HIANIVCPSI

WEPVKTDQNIGIRV*YLPQILLSTL*HFDFRITGSKSC*KLCD*RTSVLDELCMSVMLMI

VDFHLLNYTGTAIF*YLLNDRTVLTVII*LVNSQIRVTKLKS*VFETHDNVL*GNKLI*I

NRMYFSKSLMAKITFYFRCIYFPSIAVLLSSYFTVMLLYS*KH**SFLAGYILSFVNIML

QVSVSIVLLLYLCD*FMCISFADLHVSKGIHENYSISQMSVNP*QMIAENCNWS*HFIES

PFCIL*TSF*AVSCQSCFNKL*HIHFALNYTLFRRKDLL*LHIIQMTFDCSLEYIS*LTL

QMLT*VHFFIDSNEY*TVTLTIAME*MAFYKRG*SLGLVLK*MNVWFCEPCHNHKIDTSL

SCTLRLAHLS*ICTMNAKVFMPWLYSFTLRLLVWE*PVLNKLKCDHILSYSICNLTFIIS

ISCI*RTVSX

>**comp103341_c2_seq1_1** len=2910 path=[9071:0-58 9130:59-181 9253:182-218 9290:219-302 13969:303-314 9386:315-402 9474:403-418 9490:419-461 2744:462-641 2924:642-745 18774:746-760 3043:761-761 3044:762-767 3050:768-791 3074:792-861 18826:862-943 3226:944-968 3251:969-969 3252:970-974 3257:975-981 18887:982-1023 3306:1024-1052 3335:1053-1061 3344:1062-1129 10110:1130-1151 3432:1152-1175 10155:1176-1247 3534:1248-1449 3736:1450-1531 3818:1532-1564 3851:1565-1579 3866:1580-1658 3945:1659-1831 4118:1832-1856 19104:1857-1861 4148:1862-1868 4155:1869-1913 4200:1914-1960 4247:1961-1970 4257:1971-2008 4295:2009-2036 4323:2037-2069 9066:2070-2074 4361:2075-2131 4418:2132-2197 13088:2198-2207 4494:2208-2225 19260:2226-2235 7014:2236-2247 19276:2248-2560 4847:2561-2621 4908:2622-2649 4936:2650-2680 19452:2681-2718 5005:2719-2742 19503:2743-2752 5039:2753-2775 5062:2776-2850 19536:2851-2862 14064:2863-2909]

HGGTSGFFSGFFSY*YNVDLINVYSCICYHFIQFILP*WPEIVLGITVT*QKQRSTLNLT

AGGKVFWRFPWLPFLPRYSRSGGLHPYFHSSLMHYIPSDSLPETSVEFIMHNYRENIHRP

EHGLIKKSLE*SGFADLSSGILISQLPRLSRIDISFRVRMKLVYLLCLTAVLVVMGTDGW

GRRRWIRVRVPRIRFGRFRVPRIRIPFRRLKPFIKPVLGTVAKTYFGPYHKGIKVGCGLY

RKFTGREIRALDANTDGLVDRSEAASYFGVDHSDAQLDHFMNMADAKRDGTVDLDEFFDA

ALAFEDEGPESAP*SAGGIQNEATECGTCTVNYFPSC*YWRDVCID*TFTQTFMFVLSGI

MGNQVSRTIR*YQIRIYSFVFVT*EFFSCMCRGCETSFTDRLKC*DLRLTYITVVAAGEE

TLKEENVKSLYLNSFYQFLPIIQKIKYILFGWCVCIYLVNIRVWLRQPVFDTVSSYRPVM

LH*NGIC*KIDHLVS*QSSGRPT*ADG*PPRVT**GIESAGN*RFQPSPETSGPRSAACG

WMTAY*PVMGLPLIPFSSYVN*FLCREMIITCKGEENVCYLMITLYFNKCTHRTLCNAYI

LVW*KTTT*YTELLLNTKVKYFQLQPKDDIKGNQVAV*EFQRMSAKLFLNLSSPHV*SS*

TRNVSNVCIAFRSRTVTILQ*SLTECITIY*TDSLDTDVSWEMPYLNLSFPATFQETFCP

*MVTMLSGLNLPLAISSCRMVAMSPWLTVWAPPPLVILTRTVPLMCTSASKSNSLSVSVT

QSLAVVRE*GWALTVEFRDHLTACLTVLSSCCCIWTSLPKLAVALEMVSGTSTLMPTLPP

LEKPLVRLVLTTASVDLSIWKVWLCTSPVLFS*FDVNTTWDRPDAVFWDRRAPWRCC*YW

GLS*QLELTVEKWTLCQSISMALLLAFPTPTRFSTHFLTTVSRSITPMLISEKKRVKLNK

LTNTVLVNRC

>**comp103344_c0_seq1_6** len=1781 path=[1:0-39 41:40-80 82:81-81 83:82-84 6157:85-105 6178:106-107 6180:108-120 122:121-122 6195:123-125 127:126-126 128:127-232 6243:233-233 6244:234-260 6268:261-436 438:437-461 463:462-491 493:492-531 533:532-569 6429:570-570 6430:571-578 580:579-601 6450:602-659 3612:660-708 710:709-1749 1751:1750-1755 1757:1756-1780]

RELVIADEVLLLLFLAKMLAVVVFLCSVSSAYSVQCMSSTGKPVDWFIVYKLPELRSNKS

NPMLRDGYGHFYMDVLNPSWQLSKVSMNDTNHAVYNTLQAIYESKPTDDFMYLMYNDDDP

HDQESLNHGHTKGVVAFDNKTGFWLVHSTPQFPRYKNESYTWPPSALIYGQSFLCVSYSY

SQLNSIAKQLFFNYIRLYDHYTPDSFLQNNPDLASVIKNTRLEKPPWSNKVVLSSLSGTK

FTSFAKFSNFHADLYKDFVAPSLQSDLMTETWQNSGSRMPSNCTGKFKVYNVKSIVLPEG

IVFKGTKDHSKWAISRNGTNWICIGDINRDVSQEKRAGGTVCFQNPKVWTSFNLAVNTIE

SCQVNVSSFHGQRVTRFEEDRGL**AWRAVVASGTKTNHIDRLVDECQIRPPTLIVITSL

F*FQLLCSSNTIFQYRCRYSLDEVTCFCTKLKLKSTRRPIFVDSSPSCGYRKVAIH*SQC

HSIWNRLC*DSISIAKSRDGCFVILVSEGTSVCGVSKLQNGFAV*SVQWLFL*KKSSQIL

TFI*KTLFYRIVRRQTKVLVVMVLVPVVETGQV**TKSSQDQGWK*HIAPTSES

>**comp103430_c0_seq5_3** len=8632 path=[26617:0-83 3185:84-1242 4344:1243-1594 4696:1595-3106 6208:3107-3109 6211:3110-3443 6545:3444-3619 6721:3620-3800 6902:3801-4563 7665:4564-5231 8333:5232-6939 10041:6940-7029 10131:7030-8580 11682:8581-8631]

PMPIQGLLSKHNTTP*RV*LPPRTVTQSLQTRIVNGENATAYRSGRVEVSNDGGTTWGTI

CDDFFNKSAAIVVCRSFGFTDGEAIAKAYFGSGSGAVFLDDVECQGFETDLSQCRKSKWK

DHDCDHTEDVGVYCYNSTDGVFKMQLAEGSAATTLNTIWGRLEVALGNMKWGSVCDDLFN

ENAAGISCKYFGYSYGQRLTEEGFRNGERTYMDEVNCRGGETSLLQCRHNSWRTHDCGRE

EDVGVVCFNTTVRLRSKSLTIRNVGAVELWKAGEGWMEVCDKGWTEDDAKVTCRELGFVD

GMALHGSALAGNMWGSPWGKHPWSTGRTVSSYSSVNCTGAETRLADCPMVRSGRGCSTLS

SASVICYNTTIDEVDNSTEVRLSDGGDNWGRVEIYHLGLWGQVCSDKWSDREASVVCRMI

GYKGGKAFGDTSRDNLPPWLTDITCVGNETNVLDCDTGEWGESVYTCTPAHVLCYNTSVT

VSLDNPTAGYGRVDISYDGVNGTICNDGWSDEESSVVCQMVGYTDGRAMNVDVQPGSGPV

WLSSVTCGFGDDSLFECDSKGWGKSSDQCADHQNDAGVRCYRNVRLTPGTHSVGVVQIYS

RGRWATVCGRTGFDDNAAKVVCSELGFKNGQALPLGVFGRQRLDSVRPNITCSSGYISSL

LDCDYDKSLTATCLPYSISYASVACYDTVVFGEEYKLEGGNIETDQASGRLRVFKHQTWG

YVCDKYWDDNDANVTCKELGHKGGIAYRYAGNYPGPFFISEINCVGSESKLEDCPKGQEL

CTSLQAGGVLCYSGEAPRLQFSPKGGKHGRLEVIINGEAGRICGKLWDDNDATVACSQLG

FNDGVALKYPKGRGSVYMTQLRCFGNEPSLFRCTNSGWKNVINDEPCVQDAGAFCFRDVR

VTGGSVNQSMAMGRISMLIGSRWHMVCADSFDQVDADVACRSLGFSRSKILRTFRATYNP

NYITNVECNGDESSLANCKYTKGQCPYSVGQAKVICFSDSQQAGTKYHIHNGFNGRVIVE

RYGLNGTICEEGWSDVDANILCKQSGYAGGVAYGTPRNSSFELVWYSEVNCTGSEASIVD

CVKNDTVSRSCRNSATSAGVLCYKSTGIQVRLADGGKNFGRVEISYDGVWGTICDYVWSR

YDARALCRQLGFVEGIAYAQSRYGRGSGPVYMDEMRCRINDKSIYTCPNRGWNNSAGYCH

DHDNDAGVYCFPRVRLEPKPQYGGLEIWTGYSYGLVCSAGFTDTEAKVACRQMGYPDGKA

LCCSAFGDMGKSIAIGDLKCTGEEASVMDCSHTKTSQCSSNHYASVVCSNVTDTSNAYIV

KLDNGTTGRVVLRHFSHDGLICPNGFDDKDAKVVCREIGMTNGGFSYKRINNNRRSFVKE

LRWMTNLNCTGEENLLETCPGITWNQINPCDRESDAAAFCYRSPGIAKPRFRIEGGTRLE

GRVEVNINGTWGSICGINVDDNVAGLICKDMNHPSGIAGKAGSRGKTVGPVWINQLDCVG

NESSIFDCPLTGFGEDDDLCKSHLYDAVIQCHSTGTIPVRLSGDINGRTDYGRLEIKLNG

TWASVCDNGFNDNSAEVACHQMGYNKGKRQCCSALGNLPRSMDPKVTRITCEGHEQMLAN

CTIAPTGECASYVSVACSSTELSEVLKISLPKNGFTGMVNVSRYGIWGAICGEGWNDTEA

ISACRQLGYIGGVAIPGSAIPLGMPIIMGNVVCRDDDTNFSDCSRDDFDTHHGCAESRAG

AAVLCSNSGEGVQYRMAPGSVGDKGIAQLNINGQWGYISRLEFDDKDASVFCRSIGMVAG

QVLWRSPSSSSLRGNILASYVNCTGSEAAVHQCQVVWDTERNRYIPATFAVSVSCFRGVR

IERGDSKTNGIVKVYNNGVWGAICSKDFDNQDATVICRELGMTGGLALCCNPYGFNFDDA

VIADLRCTGKENSIRECKFSSRQRTSLCYRQNYASVACYNDTLSNNYTISLSGGSRYTGQ

VAITFANVEGRVCSDNWDDPDAAVVCSTLGYSKGVAYSHYRSSFSYFDYTGPYWTSQVNC

TGNETSLRDCPHIGWGNVTKCSSGHFGGALCFNNEGLFYRLSAGGDQWGRVEVAVDGQWG

SLCDRYWDKREAKVFCKNLGFDDGDPFYGSYNDTATGPVWESNLRCDGGEKTLNQCPHEG

WAVSTSRSCLDHTRDAGVFCYTKVKLSTGVGRSIQHGGVLWNQRGDWHYVCDTGFNDMSA

RVVCQSLGFKDGRSICCSAYGNTNSYNKDIITNMTLRCTGEEESISECMITEDCVSKMYA

SVVCSDNPQEFVNDNYTLTIKTGEKNSGQLRVAHYGVQGRVCSRDWDDEDATVVCGARGY

TGGMAYKHSYVGTYSTQRQLGPYWLSSFNCTGEEKDLMDCPHLDRTNLGNCSNKHTAAVL

CYNDSGIEYRVAGTGLDYFGRAEIRIGGVWGTICDNYWDAKEANVFCRQLNFSDGVAISR

ARYGQGSGPIWLSHLQCTGNEKYFHECPHRGYKDLYSAPSFGWPFTLPCTSHKDDASVFC

YKSARLNQRFGATKGGLEIFDDGKKVGVCDTGLDNTAATVACKSLGMNFTHGRAIGGSVF

GNISGPIVFTSIKCKGTEKDIKDCTLSKEGTCTTGTYASVYCSKTPFNDTGFQIRLAADG

LSKDYHGIVEVKKNGVWGRVCMQGWDDMDASVACRSLGFKGGVAYLHIVKNTNAILMRNF

RCSGNETSLDKCPHHETADKQNCNYDSNDAGVICYNKTGIEYRLFGSKISNYGRVEMGYD

GEWGAVCSWAWSTSDAKVFCRSLKMGYQDGLPDRNTIPNLPRRDYLLSGAFCNGNEQSLV

TCLNSGFNTTLLSYLCSGDAYTTCYTTDIALTHVRLGKDRSGDQSVDRGRVEVYVSG

>**comp103502_c0_seq4_5** len=296 path=[4621:0-65 4687:66-84 4706:85-145 4767:146-160 12072:161-172 12088:173-176 4798:177-189 4811:190-205 12130:206-215 7692:216-220 7697:221-236 12189:237-244 7721:245-295]

FVQLGIEVV*Q*IIVSNRYCWCVLQGRMHIK*NPENKKGWFLR*SKLTLISDRTCIFSLF

QLLHVPPCNTNK**RFGAVIVC*GTFFLG*VKF*DLKVX

>**comp103559_c0_seq3_2** len=497 path=[1:0-329 331:330-330 332:331-369 371:370-413 3748:414-417 7106:418-473 7146:474-496]

SAGCKVKIQENNMMAFASLFGVVLLLSMCSAVEWKHLTGRVPNGDRVPFDIAMDGKTMIT

VSHKPEGSAENFRETTNLFVFNRNLVAIRDSVDKRCYIQHTDATYAEMKAMVNGDAQDTN

GDVTGDEERIDVSSLTCKPFTPFAVKLLFGDEIESFCRFHDVYFLX

>**comp103563_c0_seq8_1** len=2699 path=[4547:0-732 5280:733-1338 5886:1339-1473 6021:1474-1583 6131:1584-1607 6155:1608-1710 6258:1711-2698]

AAGTQGLEMRLVAVVLTCLIACTLGLNHSFTVDYNNATFLQDGKPFRYISGSLHYSRVHP

YYWKDRLTKMRVSGLNTVQTYVPWNIHEPTPGKYQWTGLADLETFLKLAQEGGLNVLLRL

GPYICGEWEFGGFPAWLLTKNPDMVLRTSDPSYIAEVDRWYSVLLPKIKPFLYENGGPVI

MVQIENEYGSYFACDYDYVRFLYNKVRSVLGKTVVIYTTDGDGDSYLKCGAIAGAYATVD

FGVSYTPTKNFAAQRDYEPNGPAVNSEFYTGWLDHWGSPHSTTDMRSVAKSLDLLLDYGA

NINMYMFEGGTNFAYWNGANAPPYQPVPTSYDYDSPLNETGDITDKYIAIRQVISKYNPL

PPEPIPPNTPKCPYGNVQMDFMTTIQGSLQTTSPGGPIKSRFPLTMEQIKFYYGFVLYRH

VLKSNLTLPTPLVTNGTRDRGYVMVNEVPMGIFNRDLTWQVNITGAAGQYLDILVENQGR

IGFSTLMNYNLKGLTTNVTLNGSILEDWEVYPITLENIRTPAHFTRQYRAGPKKSASGDL

ETPSIYMGKIPVPNMAGMPQDSYLDMRPWHKGQALVNGFNLGRYWPVEGPQVTLYVPKPE

LVAPPGTNSLIMLELESTGCQANSSCTIKFVDSPFINETPKGSTLSSRVVRENFDWHRFH

*GNIVSWCYNVYDVCHHVCMSLLQFDMQ*HSTYLPFKVGYFVSGSFLTLNVLNILIIFLL

SFYIDLKKPETIYFIVQQDYFTLYIIYGIAS*QGSVNYNVYNVFLHI*LSD*SNSSDTVR

EM*LTLLVSLLKLLVFRPLLL*YFSAVCDEHH*CQSMSRLTGTI*CDDDSLCSITMMKED

SDYIYCVYFS*LFVC*CCD*SHKSHCILSIFKRNCCCSRFVFVAAVVLLTTT*VKAPPPX

>**comp103663_c0_seq1_3** len=4523 path=[4546:0-1935 11582:1936-1960 6507:1961-2037 6584:2038-2248 9114:2249-2280 6827:2281-2326 6873:2327-2330 6877:2331-2546 7093:2547-2552 7099:2553-2706 7253:2707-3014 7604:3015-3474 8064:3475-3482 8072:3483-3727 8317:3728-3748 8338:3749-4002 8592:4003-4007 8597:4008-4116 12201:4117-4133 8725:4134-4162 8754:4163-4522]

SEADRSALAAETHV*YSRSCRYGNIYCEYISKVDVLFI*DTDQ*RASFPVLVKTT*RQES

HPVLW*SDKMADSRGLTAEQRNELCELFSGVDEDGNGSISLDEIKDALAAVNIKLAGYEI

REIVERYDTDGNMKLDMKEFEKLYVEELKKRNFGLKFKTSVAPKRGVRSHEGVSQASTVG

TTHTVKDSELVAFSDWINTSLHEDQDCKAYLPLSGDTLCGQCRDGILLCKLLNQTEPDLI

DERTINKTNLTVFRKHENLTLALNSAQSIGCSIVNIGGEDIEKGTIHLVLGLIWQIIRIG

LLSDIDLTHHPGLVALLNEGEDIGDLKSLSPEALLLRWVNYHLRQSGCSRQINNFNEDIK

DSEAYSYLLTQIAPEDSGVTLAPMQAGDLTERAEGVLNESDKIGCRSFVTARDIVTGNPK

LNLAYVANLFNQYPSLDSAGKDIDLGEVHEETREEKTYRNWMNSLGVTPFVNYLYNDLGN

GLVIFKLYEKIRPNLVNWKKVINQFNKMKINFEKLENCNYAVSLAKDIHLSVVNIGGEDI

REGNPTITLALVWQLMRAYTLKMLTGLADDDKPMPDQDIVTWANQRLEGTHKIKSFSDPA

IQDGVVILTIIDKIRPGVVNWDLVSKAETEDMRCLNNAKYAISLARKIGARVYALPEDIV

EGKKNMVMTVFACLMTIDNPYKREREQAKLNDAAKPMMQGLQ*STPCVKGGGALHGRDLH

TRIDGVRCFIYTKISCTKSMIFILQIFKDA*GWC**GTLSGLSFWIL*KHVSNGGLRKAG

WVLSVMQCRGRCCQDELASRPIYNYYSKYCVQVS*PIFVSLSIAYHYCIIRILLLAKTRA

K*FIDVLFR*NETNVLCRFMHQHLNLTCFWLLRNRPQSCVHLRLNPCMQVPVI*QHQDL*

QKLWNFVSRLFQHICLKLPAYKYEPIVIHINTC*RLSFYS*LFCFLDFYPKIK*Q*LVEL

LFLQKTKIEKADISKLYISYSPL*SIMIFLVVKCIIFTPLQASSSTSCSGRWVLLVSVQA

HW*GHADDDDEPDIYDVLQMTLSINHLLCCVILGRTKHQNRNMNLMGPMLENDDFYGCIF

LILSFHF*SV*SQRLPFKVEMYYFYEVTFIMIVCVWLPF*RSQVDVYVYAGILSELKSCL

LKQVE*FQQ*TEKGKVVYGSEFVQQLMYCFRVNSHVYIIFNC**FPAIVNA*WMHQ*PSL

YCQDSFQKCQ*SAILTFCKSISCFNCFNMDIVSFSVDNDHVLIMMPCCSDLPGSEASSNT

YLYVGMQC*ARLIINEDVAFSMQSDMVGLLPWIQGPCLLLVQDDEQYFPFVDFVLH*LMQ

P*SDILLKDKYC*LYIMSEL*SEQIYVFILICYITRLLF**LLQEKD*DALVVSMVTYIM

FNDRAMKPVISRSHPRDLQNSEIGTSGCEV*SRF*RDHFTTELVFIAIL**N*FLCNVSP

K*L**LTSVECSKNYLRIKSKLFC*TDNTFLAILFASLLFTMCWSSCTGKPSNCVTCSRQ

FGDCTSW

>**comp103706_c0_seq1_3** len=612 path=[11338:0-116 23326:117-366 11705:367-611]

KRMYQTTCIFTSSTKI*RLIRYK*GGATVIDNSVQVKQLFFCMMMLRPTLVLLLLVLMLT

PEESEGWRRRRRRRRRVQVITHNTNRCHCNTVQADIIKLLRDLLRSRGLGKREVSTVFDV

EEDDPLLEEFMQTSMNKRDGLVDRKELLATVERAAQAPQKRNSNHCLCGRMLLEGAIRGS

SSSG*T*GG*RF*PTRLTNKFSKX

>**comp103751_c0_seq12_6** len=2216 path=[7068:0-37 23519:38-53 18143:54-88 23565:89-112 18202:113-225 7294:226-301 7370:302-1567 8636:1568-1583 8652:1584-1878 8947:1879-1947 9016:1948-2215]

PRIEVMVYNYQSVC*TLELSSLWRLILAGSVHASACGSVSEKQPRPFLIGHDNAENGAGR

LLSSYKGLTTSPFSTMLRLLAAICLVTYSDCSLYPYGPGEGDVTLNTDNVPVNLGNSYNY

FGRKFDTVYISKDGVVGLNPDVKYEEVRFQDGGRDPDHDLSFLAPFHFNGNNITSDSEGK

IYYKLYYSNTTEDQDFLTELRYYMLNATVGNLPFEPAVALVVTWENVTAASQEACACQRA

TFQMVLVACSLNTFIIFNYNDISLTIQQFYQAGINGGHGVGWTSVCTGCNLIDLPSTRGS

DVTGRFIFRVSKDQLERSGCTIDGSTLQILPKYAGMFGGELLDISGPCLDDINVSVMCRF

GSGESSVTSEGFRFNSMRLKCRVPRLTGRGPVPVAVSVDGGSTFTGDGTIYVVLPLRLPT

KSLVLGKGWYDVTPSELNMTWDPLEMTSDPDAKVDIKLVGYRETKTELIWKILRVIASSA

NNTGNYSFRTAEHQCQPADCQLFEVAIIEVQLQKPYHALATSRVAMSPDDAVTIGWFVKN

AMTTQYGSNWPPQLCTQWSNRDKVESSWMNQLLYCPCNLGQALADFGRWQADLGCNIWSS

SSSNCFYHKGAVHCVRSFLPTSTGAGNQCCYGSDGVLKYAADTYQGSTPDRSHDWGAAPY

NKPDFVPYFSHWIHDVVTFYYCCLWTDNSNCDDYMARRPTRDCNGYRPPQAAMAYGDPHI

MTFDGKLYNFGGKGDYYLV

>**comp103936_c0_seq2_3** len=3060 path=[1:0-115 119:116-139 143:140-484 8891:485-504 508:505-514 518:515-520 524:521-687 691:688-695 699:696-1358 9049:1359-1722 6570:1723-1741 1745:1742-2145 9191:2146-2166 2170:2167-2206 6155:2207-2228 2232:2229-2230 2234:2231-2340 9273:2341-2348 9281:2349-2352 9285:2353-2374 9325:2375-2375 9308:2376-2390 9323:2391-2394 2402:2395-2491 9424:2492-2498 9431:2499-2520 2528:2521-2753 2761:2754-2774 9570:2775-2781 2789:2782-2900 2908:2901-3059]

RLSTVG*FTCVGPLT*LDGSNEGRARVVLVKHGLYTHTPGYTMTTPWCRLAWVGILATIS

LTAGSRYHEQFDKTVEAFLLDNGYTGGALAAMKDGRIIYAQGYGVTNEGREVKDTTLFPV

SSISQSLTAVAILQLAEIGALDLEGKVFGHGGYLHMLKAHRPDSVDPRLHDITVRHLLHH

TGGWDMNKAPLFDVMLNPYYLMKGYNVPNISHIMNSPPPLSHYDTIRYMMSQRLDFTPGT

KTVYSNLGYIILGRIIEEVGDTSYEDFIKQNVLKPCGMWHTRLGSKEKIDKIDNRSNRMS

KDSSDWQDLTLYDVLHPAVIDSALGWYSNVFDMMRFLRCIEHHDSSGLINKNSWDTLARR

PEAAPVQHSANWYGTGFRASIQGNIWQDGYKHANDVLLFHSHKHEHAIHLPDAWVLLLDG

QKLRHLKHKSHELMEYLGVGFSHDEIILRDLSDVQVPATVVKFSVDEHHLHAYINALKQE

SYDVTWISGHDAHRHTRFTVIAEKTEQPHDYLVEHGLTEKKLFSRKLALEDEGFNMTYLQ

NYKSASHDERHAFLAVFRKSAFDKHTHMKWGIHHYPRPYDKLLGLYIEKGYYPTVQSYIH

HEDEALLSFIFLKRDNQRKVNFKEYHGLSIPQLERMVRSNANQNRKLTYVDVCMVYRKPR

FSAVFTNQNPKQWIFVPELDQDSADKIIREKFAEGYVPRKIVGYARTDKSVKYALYVEKE

*NSLGYYHYSGVLHTQVPAYRLHGGHKM*NTVFDLFKIGLIRSFGVYLCSEMTILED*RK

YIYTTQKQLNI*LFSNDYS*SRMSMTD*FSSIYELSVLNFFGVLYYILLMIRRNDCIQLQ

FILIYYH*ICQRSFYTSYRVNNFIHVILAEWKMNLKD*KILISSNVSRMENSCSDGTLDT

FLDTNNSTTLRHILTSMQLLVCNVRCLKSSG*HCHVV*L*RMY*RIW*IMWKGYTAPVYI

TTSAIWLIIVCIVYR*RVKCYIEATRMAFMELVHMCRNLYIISVLCMSK*MCVIKPLYKX

>**comp104220_c0_seq2_1** len=2270 path=[2472:0-45 2518:46-145 8356:146-147 8358:148-227 8383:228-239 2712:240-261 8404:262-661 8601:662-730 3203:731-734 3207:735-886 3359:887-900 3373:901-910 3383:911-917 3390:918-924 3397:925-940 3413:941-962 3435:963-964 3437:965-1010 3483:1011-1034 3507:1035-1049 3522:1050-1092 3565:1093-1106 3579:1107-1126 3599:1127-1130 3603:1131-1165 3638:1166-1168 3641:1169-1172 3645:1173-1189 3662:1190-1192 3665:1193-1196 3669:1197-1203 3676:1204-1227 3700:1228-1249 3722:1250-1273 3746:1274-1305 3778:1306-1307 5923:1308-1329 5945:1330-1331 3804:1332-1359 3832:1360-1371 3844:1372-1395 3868:1396-1459 3932:1460-1466 3939:1467-1490 3963:1491-1502 3975:1503-1596 4069:1597-1631 4104:1632-1745 4218:1746-1769 4242:1770-1813 4286:1814-1841 4314:1842-2018 4491:2019-2053 4526:2054-2077 5081:2078-2140 4613:2141-2141 4614:2142-2175 4648:2176-2176 4649:2177-2217 4690:2218-2221 4694:2222-2269]

QDYMLLGYTALSALLKMSHRKDSMISAFVLLMMATGARAIECGKDDNPCKIQLVVDPRLT

MMDGSRRVMPSNGGLRYYNETVPILDTAVLDRVLTADGVYARLVISVNGQFPGPELIMYE

DQNVEVSVTNELHTDSVTVHFHGMHQRNTPWMDGVAYITQCPILPGATFTYKFKASPAGT

HWYHSHIGDQRSMGLYGPLIVKKRESPPNIIDKHIIIIQDWNHDMDPETAYQKMIYGIYN

CDEKSTKCERYQETSTVDGALFSRFELHTGLINGRGRYHYGPRPQDHNGAPLTRFEVDAN

MTYRFRVISATTLYPFRVYVAGHPITIIASDGRDLKPIPVDSFVIQSGERYDFYLETNQQ

VGDYMIVAESLEVNKTSMHIAEAILRYSRPADDSTNNILVSSERTCSKLQPCLIFNCPYR

YYPANQYRNCSTYNFASGIEDDKDRDEILKEKQVKQFFLNFAFPGEPGNTPGSVNGRQFV

SPKVSALTQPSQVHAACSDLGDKCGADKICSCTYTIDISEGDIVELILFNMGKGRGWSHP

VHIHGHSVYVLAYGFPQYDTATGMIREDSDDIECEDDYCNVAKWRGRQKRQTNVQRNSTK

RIPPLKDTLIVPSGGYVITRFKADNPGVWFLHCHIDLHNTNGMAMAINESFSKHPKPPAG

FPVCGDYTDFKLPPVSGAEGPLLSPETLLVLVLLGADKLMPWSY*YNYHHQQICTDFHVK

YSRNCQLLHKQSRFYQQKSSHTQGNIYMGILLNICTX

>**comp104253_c0_seq4_4** len=1117 path=[18587:0-325 18913:326-676 19264:677-821 19409:822-894 19482:895-995 19583:996-1116]

LSKSRPRRDHVMRVSAFTMQCNPVFSFNATERSKMLLLVALLFVSSVNLLEGRCCAPRQW

EGWGMRLGGQMAGATHERPELIKAKINLHYDADVPMVVAVANTSVGTRKRLLTAIQDFRH

KTLYVLEDGSCKVKPLPEPFMELCSPKGQDGTFMWLGFGSTAYPALEYVYTFKQQTVDTR

GTMCAVWTQDSCSPVSESFYGTFIGNPQVYTFSYSDINKGIRDPSIFHIPKMCREANMTM

TDALPHHDIPLTSAQYFKV*ST*SSVFVYER*TTSSPSVHECASKVTFVQNIVRHIFVYI

HTCSVL*SYVTVRL*SRSFREMTRDFSHFVK*LKISVTS*ND*EDQPFR*WLYKLVWMFP

SNLI*MCLITIA

>**comp104257_c0_seq2_5** len=1992 path=[12055:0-248 12304:249-272 12328:273-429 12485:430-453 12509:454-480 12536:481-489 16043:490-509 12566:510-677 12734:678-701 12758:702-816 12873:817-840 12897:841-863 12920:864-866 12923:867-887 12944:888-891 12948:892-934 12991:935-958 13015:959-985 13042:986-1009 13066:1010-1027 13084:1028-1051 13108:1052-1174 2592:1175-1200 2618:1201-1262 14713:1263-1286 2704:1287-1297 2715:1298-1321 2739:1322-1342 2760:1343-1351 2769:1352-1366 2784:1367-1420 2838:1421-1444 2862:1445-1498 2916:1499-1588 3006:1589-1610 3028:1611-1666 3084:1667-1670 3088:1671-1684 3102:1685-1690 3108:1691-1695 3113:1696-1708 3126:1709-1732 3150:1733-1748 3166:1749-1765 3183:1766-1809 3227:1810-1836 3254:1837-1860 3278:1861-1967 18823:1968-1991]

TSSISPSTTEVMTSSTMVTPTTTTAPTTTAAPVMDVEGKFSIVEGRNFTEELLNMSSPQY

MNLKQDLQQKLDGLYMTSSLKGLYLGISVMAFSQGSIVVSYTAEFNSSNNVTGDSVTKAF

LDGLSDGNKLGNYTINNDTVSHEVVPRTTAPPASTTPAPTPVPQDEFPNWAIAVIVCGSI

LLIFLIFMICVLCSRRHTRQKYRMEEDPDDIGYRRTWADSPHDYAYDNKMAVPESNENGK

DAPVRYDMTDTLPRDQQRTTSL*VSGVQDLMV*VL*SNLVYVVALTPLR*FTQYVMSHMT

LLKVGVVTYVRRYDTERN*TFILNFTCLFSAFLLPILLTPHFKSKYPVCQIPDCLNQTKN

KHPSLTRRCNHVICIGS*MHKL*ILH*TGSLCPFRKAICSDCDDSSKCDTYSIHGGSYLP

VYQQLSCVSCPLVFSWDQSARCQVIYLRSKG*IASLQTEVGIPLWIKYFDHVMAPRSVT*

LGVFRGFV*RKT*TVSPRTSRPITV*CKANSRRRQR*R*TEPG*VLLTNSGCFSGDITL*

HLTRLTRL*NGF*RNQNNDVTDRQRPSFSNEFSTTKPNLLINHFNADCPPWNDT*LI*HN

IILKIN*SQ*ASRDDVVNAMSMIKCGGDSSLPSVLTM*CKTSVWHQTFIYQ*RSC*ELIF

SNPX

>**comp104383_c0_seq1_2** len=2832 path=[1:0-9 11:10-58 60:59-85 87:86-137 139:138-202 204:203-407 5642:408-433 435:434-797 8779:798-809 811:810-816 818:817-984 5968:985-990 992:991-1018 8870:1019-1193 5836:1194-1212 1214:1213-1310 8980:1311-1380 1382:1381-1402 1404:1403-1499 1501:1500-1521 1523:1522-1537 1539:1538-1738 6918:1739-1751 1753:1752-1760 1762:1761-1947 1949:1948-2009 2011:2010-2317 2319:2318-2346 2348:2347-2447 6762:2448-2454 2456:2455-2763 2765:2764-2831]

NACSDGITVDLTPPTAGTVHIRGLTGMYKVSTTEVEVRWTGFTDVEEQKSASHWSGIASY

QVAIGSVAGGEDVVKFTDVGSVEHVTLHSLNLQAGRTYYASVKAIDFVSKTTMAVSNGFL

VDSTAPSLTGRHIQLEHRYIASRFLHVCWTNVFSDLESGLSAFMVALGTRPGYDDLVDYS

FTREECIDMDTNGRAVDGHSYYISLKGYNGAGLYTLSSSRPLIADTMPPSAGHVYDGVQS

TATPNDKDKDYITSVSEMEAYWEGFADPHSPIAVFKVKVGTCAGCDNTLEEIEYGLTPNI

SLQHILMSPGRKYFTTVTACNTAGVCSSPSSSDGVILDSSPPVTGTVQDGTGDVDIQFQA

ARTFIGCKWRGFYDPESGLDHYEWRVGTTSGGDEILTARNAALEEVIFHTLSPDQQLPVG

QTIYATVRAYNKAGLYTESTSNGLVVDDSAPTVVISPTLSASIASAVARSSISRTTLSAL

WKFEDSQSSVERQYLSLSSHQLGEFERSTIEIPSFLTEYTYTGLELHDGSRYMVKVIACN

MAGLCTKAETEDILVDNSKPQTGTFAIETDHAAKLSRHQRGGMTWTQTSLSLNWLGFSDL

HSGIDNYIVSVGSKQFGTDYNKGGSPAEVSHDASGVDKGDEGVIQTFTVPTETLPPEGSV

FVAIWAVNRVGLQSDAYHAELELSGDRILWLVRRCQAYNCEGHCVCAVQGGTCGEPKGCT

QLNNGGGNSVLTVVDVVDLRLRPAAGFSPVNTLMAATWSVTTSKGLPITRYEYSIGESGR

PSPQGVFSVATERVWYDAGQQTSAILPLPQGRVLTSGGRYSFFVRAWYDANTYSIFTSDG

VLVDTTPPAVTDKLGRAVKEVEGPGKTKEIDFQIKADKVTYTWTNKFISGQSGILKYRQY

ICTVPKGHSIHDSGDLTSTSYTATGLSLRPNTVYYSSVLAYNKA

>**comp104422_c0_seq2_4** len=3842 path=[3820:0-112 3933:113-123 3944:124-156 3977:157-157 3978:158-1701 5522:1702-1703 5524:1704-1915 5736:1916-2336 10745:2337-2337 10746:2338-2340 10749:2341-2484 6305:2485-2555 6376:2556-2565 6386:2566-3428 7249:3429-3429 7250:3430-3605 7426:3606-3652 11076:3653-3841]

FIRYDNCARLSAT**NCVVDKLGISGVKMGSQVDPAFRVVTPNRSGFFVWRIEDMKPVQV

PKDRIGQFYKGDSYIILSMKEVKGQRLEQNIHFWLGSKTSQDEAGAAAYKTVELDDFLGG

APVQHREVEGKESELFLSYFAKGLKTIEGGHKSGFNHVDNTFQIRLYQVKGKRNPRISQI

DINWGSMNHGDAYILDLGKVLFVWVGNDSSRKENFKALEHARSLRDERPGGIVVTVDDGE

EEKMTADEKKLFEQHLPLNEKSQVKTTESVAADNVFERKSAENMKLFSCREEDGNLTIKE

VKTGPLSRKDMTSDDSFIIDCGPEHIWVWIGKQATKSERSEAMRNAVGFIQKKGYPTDTS

VTRVVDGGEPSDFKCLFPDWPQPPPPGKVYSRSRIAKTVQTKFDASTLHNNTALAAESQM

FDDGTGKVEVWQVQDFDLVEVDKNTYGDFYAGDSYIVKYTYKVNGREHYLIYYWQGSKST

ADEKGTSALKAVEMDDALGGAATQIRVVQNKEPPHFMAMFGGKMVIFSGGKAGWQKGDKD

LGPGDKYMLQVRGTSELNCKAVQVDLSATSLNSNDVFVVLTKSTVCIWAGKGSTGDEREY

GKNIASRSPRTHEMVFEGQEKESFWNILGGKQPYASDKRLQDDESAHPPRLYQISNASGR

IRAEQIQDFCQDDLVPEDVMLLDVWDSLYLWVGSGSNKKEKDSAETVALEYLSTDPAGRD

KDTPIIKVKQGLEPPTFTGFFGVWDRDLWSKGKTYEELKQELGAQAVQGSLVQEAMNGND

QDFNEVPKYSYSQLTVATDELPSGVNPENREIHLTPDEFKQIFKCSYEDFSKKPAWKQQH

IKKGLQLF*TG*PPSFK*CITHAICSQNIHYLSPLPG*MENSLVISGIVHLRKGVLDELN

EVVLPYIFHELYLYSYICIWSNFLFSRFKSTVNEIMVFLIG*NNQSGPITARCTLIGCLF

L*IQKVEKRFVYYLQTDIQLTRPHILIYRIQPHTVGLVV*WGGG**MDSQVGCH*FSIQN

KVQVGSLIKIKYFQLKMFSVSSKYYIGCIF*MKNKGEAQGFAIYSRFSVQYFA*HIYVDS

KSKLSFFANILHIFGLPNTKLRAFHLPQY*YVYI*IFCNWHI*KYSFIYFIV*FLNKS*V

KVSQGFCVYIIISLYGRETKEHYHVHSPVFQHIQPSCIPPSLGGTCYT*PMIQTRPITHV

FCLSLGHVFQLLRDQLCITGRRML*IFFIHPFSTFVRTGHTFGKYEVLLHFVFSLRVRKK

LDPGRWEGLPKI*LTD*LSL

>**comp104443_c1_seq4_5** len=1584 path=[1774:0-254 19784:255-302 19840:303-304 6695:305-305 6696:306-320 2307:321-341 2328:342-365 2352:366-387 2374:388-421 20394:422-437 2424:438-438 2425:439-444 2431:445-581 20616:582-593 2580:594-668 2655:669-688 20661:689-756 2743:757-836 16670:837-838 2825:839-1128 3115:1129-1147 3134:1148-1287 3274:1288-1306 3293:1307-1344 3331:1345-1348 3335:1349-1521 3508:1522-1583]

QGQHTLKVVLSNLSYSTMATKEGSTKPVKYTLVLVRHGESEWNKLNKFCGWHDADLSETG

IAEAKSAGEILSEKGFEFDVAYTSVLKRAIKTLFYIQDATDHHWIPVKRCWRLNERHYGG

LQGLNKSETAEKHGEDQVKIWRRSYDIPPPPLEESDERWSGKERKYDDLPKSDIPACECL

KDTVARVMPYWESDIVPDIKAGRRLIISAHGNSLRGLVKYLDNVSDSDIVGLNIPTGIPL

VYRLDEDLRPVSSEYLADQEKVKAAMEKVANQGKKQ*REMGTLHDATSLLLTKPC**GGV

T*RL*REYAHCISEVSFLFSQKQFVGIISVYLNVFSWSLVDDY*TGLYCLPSLSTYMCDS

SSHESVHMQFSKQNLSFTPIVLDSNDPKLLNLTHE*QGLLHG*F*VLISSSHQMC*VNRL

FFFVFLTYIQKARCVKFVCGSWVSYKGR*LSHA*QESNTTALNTNRFSPLFVLLLMYNLV

ANSTP*C*CFHVSGWGCL*TIDIYCVYIILQQSHLYCVLTCLHC*DNX

>**comp104510_c0_seq3_4** len=952 path=[10630:0-117 16629:118-146 10777:147-227 16798:228-236 16807:237-300 11934:301-322 10953:323-381 17616:382-393 5599:394-451 5657:452-472 5678:473-501 5707:502-512 5718:513-579 5785:580-625 5831:626-665 5871:666-671 5877:672-792 17829:793-811 17848:812-816 12105:817-832 6053:833-849 6070:850-873 6094:874-891 6112:892-896 6117:897-906 15645:907-951]

DDPLVGL*FIWARPTTRPEVNKGRNTIWSYDHFGYDNFVERKSTSIV*DFLCYSYVTRLI

YLHISRHELWISTRGDPRTPTHSVLQSKLS*Q*HHNVICTKCFTILDRTLSKLKVHVYLI

PKP*PQYLHVFTSSV*PSDVYLYMIRTT*FTHSTSSDLFS*QHV*KFNAVC*VALHAMGS

DIRTSRQLSPHRSSFNRGFDLNKETG*MSEYRFTSR*VIFQ*YHTGEH*KYDSYIASM*G

IEPRSSC*EANTLARGLLSRRLGLLVWM*IVRRPNHLCLGCSGY*SQIWL*FPTHPKLEY

C*ITNCSANRVAQQREN

>**comp104585_c0_seq4_2** len=1566 path=[201:0-43 8100:44-63 265:64-73 8128:74-88 290:89-91 293:92-112 8159:113-151 353:152-188 8225:189-199 401:200-200 402:201-202 404:203-276 8301:277-278 8303:279-302 8327:303-304 506:305-308 510:309-349 8365:350-362 8378:363-374 576:375-395 597:396-539 741:540-544 746:545-593 8496:594-636 838:637-644 846:645-727 929:728-770 8622:771-833 8675:834-845 1054:846-861 8700:862-873 1082:874-891 1100:892-942 8758:943-963 8776:964-1063 1272:1064-1085 8863:1086-1114 1323:1115-1177 8934:1178-1207 8964:1208-1268 1477:1269-1277 1486:1278-1280 1489:1281-1292 1501:1293-1293 1502:1294-1304 1513:1305-1364 1573:1365-1418 1627:1419-1442 1651:1443-1460 1669:1461-1462 1671:1463-1480 1689:1481-1484 1693:1485-1504 1713:1505-1521 5:1522-1533 17:1534-1538 22:1539-1565]

LSGPL**A*VNQ*CMVKHELADINVMEH*ELHS*QQKHRIVGHGVFDSNIAIMKIYICDT

PSDYHVSVLNVYR*QHLHQPQHLRIQHYM*Q*HHSGSYSNIYIISYNLFTYAERIRPLSP

LSLNITSYEIFVHY*NICIL*IY**NILPLISV*IYYACKYANMVC*CMLLSCYVCYIML

SYSAYPSLRLCHTLTW*NTFYTIKCFITQSVSYMNHQL*NCISLNLGRDEASVT*IGSQR

FTYFGVHIPCSKVRFSPGQ*LLMQVVLPHCCLFPSDKYEHFS*DLILFCYGDLGQVFKYN

MKVFICIYRLEFSKLEFSKDIVICFISYCSCALIFPSFINRVDL*VYIVIL*D**YV**Q

QITKL*LIF*TSFFIVSFVQEKVTSLKVFLDL*ILFRVWVDVRQKAFILKYNSSLKVLVI

FF*CLDRVTFIMVRCFHISSFMRRYLVCLIKSK*RFQGK*TFHF*RFFTIIQTQQYYSLM

TAVFISSVFM*KDH*LASLASAKQLRSLGPYIG*PCLIHNAX

>**comp104737_c0_seq1_2** len=3249 path=[12085:0-133 4782:134-501 5150:502-503 5152:504-662 5311:663-1188 16327:1189-1193 5842:1194-1239 5888:1240-1512 6161:1513-3067 7716:3068-3080 7729:3081-3102 16877:3103-3247 11236:3248-3248]

CAVINSCHSRPG*TGRPVASVHGKMKVVLLFHMLIAGAVSADECQVCTTPECVATSAYIT

SLMNTTQDPCENFYEYACGGWEHNTLIPPDKAKYGSFQEVETKNKALLKKILDSKETMYK

GKNSTSVAKAKTYYKSCMDKQAVESLGIVPALELIKQLGSWTVTSDSVSGTWQESSWNFL

DSLLIIHKLNRPAFFHLTVGQDDMHSDQNIITFQQSGLTLGEREEYTGNHTEKYKTAFLG

FSTRMGELLGGDNSTRDKMTKVYDLEKSLAEIFMPKEELVDPVAIYHKMTLEEFQTFIGS

QFTVKTYVDKLFKTNIPLSEEIVVYTVDYFKKLGDVIKNTPKEVLANYMMWQVLSMLPGY

LPDKFVDAALILTKAETGVSDVSPRWQRCVFKTSDALPFVTGALYVQEKFPPDSKQEVLN

TIDNIETAFTRNLNTVAWMDDETRDVAIEKVMTVTDMIGYPDYILDPDKLDTHYENMTIT

DGQFLVNRISVMSFQKQYNLNKRGKKPDRTEWDMGPSDVNAYYSPSNNQVVFPAGILQKP

FFNPLFPLSFRYGSLGMIVGHELTHGFDNKGRNYDKFGNLHSWWTNSSAKSFKEKTECMI

HQYSQYTVEGIHLNGEYTLGENIADNGGLKTSYSAYQLAKHTAGPPSKLPGMNITPDQLF

YVGFAQIWCSYYTPEYAKQTVMTDVHSNAKYRVIGPVSNSYDFANVFNCPVGSPMNPSKK

CTVW**RCNFR*SPHVSLYSHQLHRCCLVHVKLLF*YVWL**EGIKRSQTKEYTDILRPR

PPYASVHYVLPSSDLFFKNIYHSVSL*K*FQ*NTYSEIIFT*HYSLEFRM*IFYAVILNT

VKFLILKNV*VNRF*SFSFIGL*EIW**CFVWGGLPLCLMKESFPLPTSSLFNLNRNIG*

TMCILFITLYNLFYVSFIFSSIVLFFLIFWG*DSTVDEVFTCIIDGPSLIPHMDTMCTVS

FIPKHLQVDVIYCTTIWLLCRPPNNINNSTF*YQVKQQKHFFKGLTKVLSVDKPTPFYRL

LFGSSKFVLSVVIYDNCFMYTQILTV*YQFHTSKLFFIFIKL*NISNVNALSFCRTNSFY

*KT

>**comp104779_c0_seq1_6** len=7839 path=[24261:0-153 4384:154-1484 5715:1485-1493 5724:1494-2789 7020:2790-2797 7028:2798-3118 7349:3119-3122 7353:3123-4102 8333:4103-4106 8337:4107-4189 8420:4190-4217 8448:4218-6932 11163:6933-7716 11947:7717-7808 12039:7809-7823 12054:7824-7826 12057:7827-7838]

TTQGPTTQGPTTQRPTTQGPSTQGPTTQGPPVTTSQSLPEVTAPVFIVEVLDSIEGIFSN

SCINEVHIEVNSVVNQLPSLFNAPQLQHCAGTSDIYVTLLDGRINFSTNRANVAVIYGLK

YNTSSKESVEACAAEFINFTRVEFPKLLRMIQDKVVPNFCNNVTYISPGCNFTRKEWGCE

DGFILDSAEWICFRGTTAIVTTPASTVAPPTPAPVPTVPRMILTLGGSFVSSGQVPEYCI

TQYHSALITELTWMQAHVQTILLEFCKDIFVSVTENLTLSADGDTKINGVGRLHLYPLSE

NIDQSRMLVCYGYAAGQLTEPKLHLTNLTLAGSKTHNCSDITLSPDKLEISNNDFEYYCP

DGYVYDKDNFKCIKESIPTFVTTATLRMQVSQNATMCTSQLAVNTGSAIKTLVSQVMAEL

QNNTLCDVTSQVLLQLKQGQIATSIKEVMISIPFGLLSMSGVKSDVQKCAAGLKTYLDDT

LETTLTSLPTQVTSYCGRTSIIKNSFRVTSSEWTCFGSLSYHPSSGTCVDEPSTSKGKGR

SPRRKRSSVRSNLKIHTFNQDNTPRWNASVPLCQDTQAPVSTDCPSGNIEVELGPNGPVP

ANITLPTFSDNSGATPTVTYTPSDFRLPYLFTKNMTVTVAATDSMGNVATCPLHVFLVDV

TPPTVVCPSSIVVELYDSDTQDVQLSFQSQPINASDYSGIERIEYSPPEGTVIRMLRPIE

ITATVYDNAGNKAICSFVYEAQPRECPEWSLPTPEGGDKACTGSVEGNKTAFSCHLQCPP

GMDFVYEPPLQYICELGGTWFPHNIVPDCTDKVAPDYDLDLDFEFEYDGNNSESCVFHLK

DKILEVLGPVITEPCAAANDFLNITATYAMSIETTKVTVMVKVYIQMVEPKRPPYNLASA

CGKLILEQANTTNLDIHIEGCGNATYRPPCTGGGDNQCPPGHIPRNEYCLECTRGTFANL

TSGQCQSCPKGSYQDEAGQASCKPCGTGTSTEQEQSVEESQCIAQCPPGESSQSGLVPCS

QCEKGWYQPDTGATECVKCPGDQSTQEPGADAVDKCFGECRPGYVSCCGVEPCYPCPKNS

YNDKYRSTYCIQCPGNTVTIGVASNSSTQCIIVDQCEGHSCINGATCLNNDTFYTCDCLP

GFTGSFCEIDIDDCASKPCVNGGTCEDSVNNFTCSCPPGYHGGHCEMVTDECQPDPCING

ICVDEHLTFRCECYEGFQGHLCDVNTDDCDGAPCLNNGVCYDMIADYFCDCKGTGFEGKN

CEHNIDDCCDCSCLNGATCNDLINGFNCTCPPGYGGSRCEIDINECNSNPCKNGGRCENL

PAEYECICQPGYTGVNCETEIDECEAEPCENGGTCSDLVNDYRCDCSSGFDGRNCEINKD

DCDPYPCSTQGTLECIDGVDEFTCDCLPLWTGTFCETQLDACQTEKPCASGATCVPNGDT

FTCSCPPDYTGTLCSTKIDNCASKPCLNNATCIDGTYNYTCNCSSGFLGRNCEVNIDECE

SDPCHNGGICNDIIDGFTCSCLPGFNGTQCESDIDDCASMPCHNGGTCRDLVNDFECDCV

RGYTGKSCETEIDECAVKPCKNGGKCVDGIGTFECECLEEFEGELCETAIDDCAFNNTCL

NGATCVDGWRSITCICPPLFGEEDCGKEKSDDYDLHCQGQNGSVCELPPIYVGTNLTELT

FCAWVRFGQLGGNGTFLTLMEINKQNEETGDEGGRPMVELSGNQATIDLFNDLDTVEVDL

VSNDGVWHHVCFWWTSGEWNLWKDGELQESGSSYGNNRNLPQWLQVIAGQKFEANDDDDQ

FRGEVSQANVYSSIFNDSIIQTMAANCSGLRLAGDIYNWVIFGDYIRNDVIIVEPGLCGE

SDCPPGYRGTYCDIKIDKYPPEVEYCPEDILVVSSNNRLSVVEWEEPVFTDDVGVVNIEQ

THRSGQTFAYGEYIVSYVAYDAENNSAECEFDIIVKPFDCVEPPAPLNGGKACRAWQHGH

YCSIACFPNYEFVEFPPPFYRCGKEGFWDPPRGSPFLFPSCAMPYPPASSILGGGVDFSG

PQCTEAFKSQLRDRFIAIMRQLDARIGLCTPEECSYDDINVDCGNRRRRDTSLNVVFHVS

FSVNLSANGNALENEGDAYITEELENTVKRGEFNFMNFTADVTTLSVNVELTCLAGQALT

TINQTKKVCLDCPMGYFLNETTGDCEICSEGTYNSADRQTQCTACPPDHTTGAPGAVNVS

QCYAVCSPGHFYNSVAGQCQRCPLGEYQNEIGQTSCKSCPPGQSTETTGTIHGEDCAYLC

RLGEELGVTATCQPCPIGSYRESFLTDFCQQCPYGWTTEKEGSTSSSDCSVVECSRGEYR

TENNKCQSCPKGTYQPKRGATSCIPCGPGLTTPGVGEVSEDRCYPGPVNECDLNIDNCDT

NAICEDLQNGFNCTCRKGFHGNGTYCVDDCEGYCGDHGTCHKDNQEEVFCTCNDGYSGPR

CQETSALSGLTDAMIGGIAGAAVALVILLVGVAVCFYRSRRGGKELVNDRIYRDNKPPVA

FQNNAYESIDRTPSSMLRLESSSNSSDAQPWKRSVFSNPDEVDSQVIFSSHL*MPVTPDR

LPA*HLQDTCLT*HRLF*PLTVPCNPTLSDIAL

>**comp104887_c0_seq13_4** len=4001 path=[875:0-42 918:43-43 919:44-61 937:62-83 959:84-554 1430:555-976 1852:977-1000 1876:1001-1746 2622:1747-1770 2646:1771-2158 3034:2159-2182 3058:2183-2463 3339:2464-2487 3363:2488-3286 4162:3287-3310 4186:3311-3480 4356:3481-3504 4380:3505-3721 4597:3722-3743 4619:3744-3779 4655:3780-3864 4740:3865-3890 4766:3891-3914 4790:3915-4000]

KMSEGDVENGETAGLISQRDRNVRIDPKVCDTDIANREIKYNLTPNRPRIHSRSVSQSST

DSYSSASYTGSSSDEEDISPREKPQKNSKGFSDFCVRNVEHNAFGRREIEIAEQEMPGLM

ALRRRAEADKPLVGAKIIGCTHITAQTAVLIETLAALGASVRWAACNIYSTQNEVAAALA

EAGFSIFAWKGETEEDFWWSIDRCINADGWQPNMILDDGGDATHLMLKKYPAMFNMIKGI

VEESVTGVHRLYQLSKSGKLTVPAMNVNDSVTKTKFDNLYSCRESILDALKRTTDVMFGG

KQVLVCGYGEVGKGCTAALKGLGATVMVTEIDPICALQACMDGFRVVKLDEVVRQIDILI

TCTGNKNVVNRQHLDRLKNGCIVCNMGHSNTEIDVASLRTPELTWEKVRSQVDHIIWPDG

KRIILLAEGRLVNLSCSSVPSFVVSITATTQALALIELFNAPGGRYKQDVYLLPKKMDEY

VASLHLPTFDAHLTELSDEQAKYLGLNKAGPFKPNYYRY*YSALFINKMAMPVMSSTSEH

SISTRCS*TGILYI*KEMHQPSM*CPTTSIIRLVLRFPRQVSQTESMCCDKSQHFCQSLI

ISLMPNHRHNKHLPQSVTNQDHVEANLSNTHYNKCNTLMSATQVLLSLVPSCIVWRLSTI

AFMGIILLLISGVLHLVV*ISFKYPSKFWILNH*HIVFIQLLSLNFKLTRLMYI*DLTHP

SFMSVILLFQEWFYEKGWS*NFS*VVTIKKYLYYLCFYSLQ*VEDKLSCKIDWSI*HM*F

KPITQMTCINLLV*HMAC*EYYITSLVQCMWLRIV*FQA**NMLSKEL*EFYVCRVLLLR

NQ*PVLF*KRDFTTYSVFLRAVIQMDVGLYIFPWYPYTSLTLFPEQQILPSLSCL*ILCS

AGVDCFCRFHV*LC*C*PSLGIRMFPPFH*YVYICLFLPPPCLQCIGNKCCKSNASQILG

LAGCQTGSHCCSRRR*IVEKMKTVV*SVTRTGYSDSPP*VLVACLHDLAKDGKLVGPHTV

HSMGFSTVYLFIYHISQTT*RSPDSRLCSKCSNSMHSSLAQTIWRVVDTKCSDVW*PGD*

QDACSHHLPAIQHMLGAGRRDSPPQHTHYIQMSNYLLALHIYKYIYIYIRR*V*NDV*EY

*LCCLSFFIIVPSVFTSPSTLMN*IHSSMSLVHSTEITNQTLINLS*KASLYSLWCIMKI

HVNLKAQLFISTCCVPVKGVWSRADNVSKIS*QGSDISIKNYSFRMFIP*MSVLLHFARS

ILFLVLDVSWMKMPRHSVEVKNKTVKVDRNVISCSKRHIVATRVQTKTHYISVRNTCLPA

CRGWVIFNTDCQI

>**comp104917_c0_seq4_4** len=6533 path=[6530:0-385 6916:386-388 6919:389-568 7099:569-573 7104:574-631 7162:632-752 7283:753-793 16447:794-827 16476:828-828 7359:829-841 16480:842-895 7426:896-897 7428:898-944 7475:945-958 7489:959-1199 13344:1200-1224 16591:1225-1232 16599:1233-1233 7768:1234-1593 8128:1594-1633 8168:1634-2365 13137:2366-2397 8932:2398-2562 16900:2563-2584 9120:2585-2614 9150:2615-3071 9607:3072-3314 9850:3315-3515 10051:3516-3526 10062:3527-3831 15152:3832-3842 10392:3843-3973 10523:3974-4746 11296:4747-4770 11320:4771-4805 11355:4806-4829 11379:4830-4867 11417:4868-4887 11437:4888-4900 11450:4901-4901 11451:4902-4911 11461:4912-4983 11533:4984-5008 11558:5009-5077 11627:5078-5101 11651:5102-5478 12028:5479-5502 12052:5503-5545 12095:5546-5558 12108:5559-5569 12119:5570-5582 12132:5583-6532]

AIQRRCRFIALWVLDKFVKMAEQNEEPVEVGEDDVDNPNYKPPAQKTLCEIQKLDEDDES

LQKYKKTLLGEVSDPPKPDDPRNVIVEKLAFCSEGRAEIEMDLTGELSKLRTFIVKEGTQ

YKIKIYFYVQREIVCGLVYKQKITRKGISMETMKMMVGSYGPKKELHCYTTALEDVPSGM

LARGTYVIKSLFTDDDKNKYLEWQFGMEIKKDWQD*TINEATVYEMYSFIFL*APLHILV

LSV*CCWLLRHYCFYK*HVYIMMYAYRDSNHLASIKGTLWEAVLVINVGFDTTNA*NLVN

LLEFPYFLVSLATTCDIKSLAD*ISV*MFSACSFSRVPIIACDTIFALSHNDHISIF*AK

VHIHVATVTTFVIMCW*TFMISIQVISNVYVISKVKYYFDAFVLP*LTASDTQYIMIVTR

PTIPNPGILLAASRSVYIFQF*CTFPFTVQSFRMV*HQNILLHIFISIQYARIC*E*VIV

QNVRC*SC**ILHYF*QLSFLLGSLENWFLE*IPVVIECQQHINCSATPYFDLWVENTMF

*RLDDF*TFYKYKFFVRAGIKMLWNIFLIMDIIHCFKMFLTVHI*GTGYVLIIPVSLTAT

FVLYTYAFYLGKCVFIWYFVAVGCL*NALHVVRQNIPVHLQPALATGVTIIGCPRGTSDA

PRQ*LTCHVRCSLVSQCDM*SPLVFSAMIL*PHTRYVCNGHHPI*LNMTSLAKHNFLFLR

FQIFFSILPVLISIYLVTRIECHVDTSNYMSIFGWVWPNTP*VWPVVHHCSCALS*IICL

CDLQ*HCYGCKQGYLVSISAIHVFPPEFLSCEHEGRGGGVTKWLWHSLLMPKTQVRFPTW

VQCVKPIFGVPLM*CCWNIAIVLTHYTSGSKSHRCAKVVVTPLL*H*PAQRKATIAEVFH

TTVEALRSLCAR*PRTTK*S*RSNQIITICQSMGIKIILVLITLCEIGTENFQ*LNNCVQ

VMRHYKFNVHIRVSPFESNIRDLFLTNFQW*NVVIRLDFL*IDVKDQHVTSSQTFEMCML

NL*SYQSHYHTLLLLWLPGNSGVSLTCWMYRHSIT*PFIITLLNSVKDPRVFSSFDSSDI

ARLLTSTFLGVS*LCRRYVIIAGLCWYCKRLSIYQSIYSAVAPILTLIRSGLYSDIQYNK

DS*YFQVILFSLVT*HTVISSI*SLNMGKNNIKLSISLSMSTRYIQKYVYHRISGTKYQW

QNTENKTYIKRGHITYAILK*PQTIYNLELCSQSSFPLQDMVLLIFIIVTTYELIPNIRP

SMNTYGLYVVLLVKL*NEDVSI**MTVRTLRWLTLKRRVE*VAT*LHDTLKETCQSLSDV

S*ASLARANIGFFLQR*RSSFDSLV*NSDYTAKWPYAPNGVGGIYCIKY*RLLPCVKSAT

CT*NPQIIIIDG*FINRMQCLYNENRSETFGRRCTDSLSYSTYITKTPKQY*LFMQLNLH

IHLVSIAHRKENR*KT*KYPVWGRH*LLIIIMYRKHVPPALIKILKRFAIVTVFGGKCTL

SSKIMDVKTI*FKCSSDFFQQYKWTATNL*AK*F*HVFV*YGHK*IS*HILLPCSLKHTL

LSQYP*HNAIF*PHSTHAFIVK*NVFLFYNTLD*VTIDS*LSP*SNKILTLLTNEKAKKI

ILIFNI*HKYCYNLIKYI*QIHDDKHYPKLI*L*NCKKNKKTQEHVISYR*IVFTHEITT

NTRDSTWYQHLQKLYATMQIHAISLPFIKDSSSKNKYFWGIFNHFSLPFK*QIYLRVDIL

IVFL*SLQAAQAASALFQFHCDYTDN*GANTTHNTQIKLIKLSSQDNAAHD*KINIGQQM

CKGAQLTQTHTFLQVPAVCLQTLVEILWLLQNRKISLTE*IHIKDRIVLYSLTHSCQ*GN

KTCLH*ICIPKTALSFLNMSKQHCILITSRIYIHSQSLEVWRNSLLQYQHTKICHLLCPF

LFFQENKFSVLFSYEPKMSEGVW*MFHLQSQESQILV*LDSVEPICPQKQS*STTTEDNC

WTSCST*CAACKHTSRAALNS*TIPGT*QIWNYDISGVSPREVSKVTKARRTV*LTENTN

SKQILL*K**HVSTLLKYWWVWIYNL**LAQIIISRKHFKEI*RRI*NHRQMTEMFVVKL

IHSMFA*YTQESQACHQFSMVKHHEIFPIKKPTQQSQ*QKINIYILTETNKGSQEMFLHY

FPALITTALFKDGDENW

>**comp105092_c0_seq2_6** len=1729 path=[15173:0-13 1159:14-26 1172:27-50 1196:51-52 1198:53-72 1218:73-142 1288:143-206 1352:207-230 1376:231-260 1406:261-311 1457:312-335 1481:336-356 1502:357-380 1526:381-492 1638:493-539 1685:540-587 1733:588-611 1757:612-674 1820:675-683 1829:684-707 1853:708-764 1910:765-788 1934:789-848 1994:849-886 2032:887-908 2054:909-911 2057:912-938 2084:939-1047 2193:1048-1071 2217:1072-1072 2218:1073-1082 2228:1083-1106 2252:1107-1114 2260:1115-1117 2263:1118-1121 2267:1122-1128 2274:1129-1138 2284:1139-1141 2287:1142-1145 2291:1146-1152 2298:1153-1169 2315:1170-1177 2323:1178-1193 2339:1194-1201 2347:1202-1237 2383:1238-1244 2390:1245-1282 2428:1283-1290 2436:1291-1334 2480:1335-1342 2488:1343-1366 2512:1367-1370 2516:1371-1373 2519:1374-1380 13795:1381-1399 10139:1400-1403 6685:1404-1436 6718:1437-1460 6742:1461-1478 6760:1479-1492 6774:1493-1516 6798:1517-1544 6826:1545-1593 6875:1594-1617 6899:1618-1636 6918:1637-1660 6942:1661-1678 6960:1679-1728]

HINIAQRARSMRRSSRDKPPRLSLGTSW*RTQHCTESTTGRPYLRAYAVISCNGVNRIRW

RYAYLHVS*FHLAVMTVTSQAETYGKCLTVTFSIVNGLGACLYFTCVQYHQMNLPRQLKC

FTIQSYWQYVT*MVILVITPR**QREAYSLETTMGHLKIWLLLILSGVVYCRTVPEHAAE

DPFVISVVTNGTYSIQVKNVTWLASAPTFVHVGGKTYTTEDNSLKLNLTTATSGLDRLGQ

WQTICFDMMAGASSVIGCIKTYNSPDLPIVIFQQIFPGGATDTSTGDPDKVVSGFPSFQI

PESEPRLGYLSYGGLMFGDDKKLGVFTADKAKLQSGLVSGPLVLFDKDNNVVITAPFSQF

MAASMFQDVDRKRVSWGIFGGAELLPDGYTYETIMYYSSNGINAAMSDWGSLMRRYYGKE

NAYRQSDLTLNYVGYWTDGGAYYYYNTEPGKNYDETILDVRAYVNRTGIPYRYVQFDSYF

YPKGPQDGTLTWVPMAELFPSGFQSLYDKTGWPVGAHNRYWSSKTTYARQNGGQFDFIVE

EATSKAIPIDQAFWDYLFETSREWGLTLYEQHQHQX

>**comp105236_c0_seq17_4** len=1566 path=[24558:0-100 24659:101-176 22823:177-235 22882:236-254 12399:255-1565]

SGFRDEDPYRRVIREDPYRENPYQRREDVYGRVATNGRFNDVAGGDQNSLLDYAQHSNEP

VFELDHLATYTAGTRSGVISVEDGLHRLRKMENTTGIWTMRCILIVERRALVVLDKSTGE

ELERFPMECIVDPTAVFKNDRREVYNNLILFTVVEDPRRKSSQGDLHIFQSIRVPSQDIV

DEILAARGGGAKLGQRIPPPPSQRPPEPPGMYGTRTTEQYPGPGHRRAGNHIPFVQDGGQ

NEVLEKDVQLLNHCFDDIERFVARLQQAAEAYKELERRKRERGNRARKRQSGDGMLSHRA

RPPPASDFIDIFQKFKFSFNLLAKLKAHIHDPNAPELVHFLFTPLSLIYEASRDPFHGNR

DLADQAISPVLNNEAKQLLLNCLTSKEIELWQALGPKWTASRDEWRGYTPPFNPVFYDGW

RPSPSVIEDSPPLPPPQVPLEAAVLAHHNQIMNHTHESDTRPMVILFNPSSQEHFTKWLF

RLRNS*IF*NYHVTLLIFHNVVFMFVFNFFFKTYCFFEI*AI

>**comp105353_c0_seq8_3** len=3029 path=[3578:0-81 3660:82-84 3663:85-527 4106:528-528 4107:529-552 4131:553-560 4139:561-996 4575:997-1019 4598:1020-1233 4812:1234-1237 4816:1238-1661 5240:1662-1695 5274:1696-1714 5293:1715-1788 5367:1789-1793 5372:1794-2007 5586:2008-2019 5598:2020-2122 5701:2123-2149 5728:2150-2193 5772:2194-2250 11258:2251-2252 11260:2253-2272 5851:2273-2304 7320:2305-2329 5946:2330-2340 5957:2341-2599 11343:2600-2617 6234:2618-2625 11363:2626-2715 6332:2716-2717 6334:2718-2770 6387:2771-2782 6399:2783-2810 6427:2811-2811 8707:2812-2832 6556:2833-2844 6568:2845-2871 11527:2872-2943 11570:2944-2944 11571:2945-2948 6673:2949-2983 6708:2984-3001 6726:3002-3008 8445:3009-3028]

ALYAGLSDAIGTVKTIYRACWAAVLQLSWYRIDTTMSTGDIYTSEKRGDDKEGGGTKEKP

FKTVLQAMRHVGGEPFPTIHVDPRDETKSGTDYDVISKSQYKKCLKLWQKEQSSAEYKST

REAEDAMRTERNLAEARKVTIKMDTTLPKPKLIRIREATRNRNTRVKVEGWVHRLRRQGK

NLMFVVLRDGSGFLQCVLHDVQCQTYEALTLATEATIAVFGVIKELPAGKTAPESHELYA

DYWELIGTSPAGGADNLLNEEAHVDVQLDNRHMMLRGENTSRVMKMRSVIMQCFREHYQS

RGYFEVTPPTLVQTQVEGGSTLFKIPYFGEEAYLTQSSQLYLETCLPSLGDVFCIAQSYR

AENSKTRRHLAEYTHVEGECSFIDFDGLLDRLEDLVCDVVDRVLKSPYGQIVYELNPDFQ

PPKKPFRRMQYTEALTYLKEHDIKKEDGSWYEFGDDIPEAPERKMTDQINEPIFLCRFPA

EIKSFYMLRSQADHRLTDSVDLLMPNVGEIIGGSMRISDQDELEAGFKREGIDVTPYYWY

IDQRKYGTCPHGGYGLGLERFLCWLLNRYHIREVCLYPRFVGRCRP*SNRQCARTTESWH

FPEIRGRYSFLYSSYNSSDQNMAQQFP*YLHWETPQYNLIKTCTDCS*WKRIVGYR*YFV

HATNTARQKILIHI*FQDFYFCCLILIIIME*GII*ISA*CFFNHVF*NSH*AP*IDYTF

YVTLFIWF*T*QLWRQIIFLKMTEITKERITLTVYEKSPNPSQTST*KCYQSKMYITSNL

FTRPPGWPAVNLDGPSEI*PVSGGRTGLISYIAP**AK*AEQIKQA*NQTK*LHMNHLNN

MGLIQ*EALYNTA*GIV*YRMIVTEKGSMDTQEISDKHLVTSVLHICCLKFEKITGPHDI

DIEVSLNRYTITDINICTTSTVR*GE*KYCMN*YDHWIELLFVQPSR***LTNNYRSKVL

IIL**IITVIISIIS*THLLTKKSFHFRDNASHLKIY*SN*SIQK*QNL

>**comp105776_c0_seq1_5** len=10130 path=[14434:0-68 27860:69-3479 17914:3480-6116 25006:6117-6121 20556:6122-6145 20580:6146-6549 20984:6550-6817 21252:6818-8037 22472:8038-8040 22475:8041-8148 22583:8149-8170 22605:8171-8920 23355:8921-9780 24215:9781-10129]

LASCATVRLKTYCFAQIAVSTRRGRGRE*PSHSLRCVRTGSRGQITEE*VME*ER*IDPN

HHTKKGL*LGRGEWKFGKLWIVVRWDNARQSAKRRGYLDSSGYPNFRFKGENLNRLYQDC

EITCVFVSGCWFIHANC*PCVKFFVSEGCES*RFFNWKHRSRFL*GSLTLTNMAASTNRC

VCKAAFLLHSFLVFVSSVHGLRSQDHNIIYMQDVDLNSEISPYLTHHRHARSRPFHERVK

RAAPVSLQRTIAENVTFTDPIFQLNSEVTGPHLFEIVSSTVDMFAVDRTSGNVRLKPGRT

FDYEDDTQRSIQLVIKATNQNNITDETQITLTLTVTDVNDASPEFKNKPYPFLTTVGPNT

PPNEKVYRVIAEDPDTNANISYRIDSGGNGRFYISTETVPLEGTFIKVGNIYTKGQGRFE

EGREFNLVVFASDDNATPPQSTMANVYVKVGLRPPQFYENPFRGTVRENNGIDQTLNGEF

KVEALAFQGNSITYQLRDATNATSAQFSIDDSGVVKTLQVLEFDVPREQRPPIYELNILA

TESVTNLQSTMKLFIEVLDDNDNAPIFELSRYSARFPEDIPINMTVLEVKAVDDDSGTNA

EVVYSINHDHFYVVTENRTGPGVGGMEYVGVIKVKQRLDYDRLPGHVYSFTVTATDKGSP

PNSGTTSVQVFMTNVNDEPPEMSLENQKIDVREDAKVGDAVTVIQAIDLDKDSVEFYFSP

RNTDFKIFRILPASGLITLSQTVPADVDEYILNITAYDDGSCCGGSPILSSEAYLNVEIV

DINTHKPSFPQCSTYNNATILEGKPENTHVIRVQAQDNDRGENGRVTYSILRPTVNPTVN

FQVDEDSGNVRSAITFDREAVSSYTVTIRGVDQGRPPLDGYCTFTISIGDINDNKPVFDL

TSYTATISQTLEVNRSILTVRANDDDLGINAAIEYFMVDSDGGVFKIDKEAGIIALNKTL

GVKNQYDLKVMAKDKGTPPLNSTVDVRVRVIDLTSQPPTWNRADYDATPYMVDETAEPRH

VIASFSATSNVDDPTVSFSLIAADGSPVQSLPPFGINSGGSTVNLTVFTTTMDFNVKNRY

ELRLRVSNRGSIPLSSEIHPVVELRDTNNKVPYFRGLDSSTGFYPGSIPENSPPGQTVIE

IKAYDDDPTYPNNFLTFEIVPKENISDKFAIRKIDDNTAVLTSTYSFDREEKSQLFVDVK

VYDGTESSRPGHNPGQPNTATVPVQVRITDMNDNSPYFVKAFYNASVREDAPVQQHVLTV

SARDPDDAAVLTYTIISGNERGAFGVQSKTGIIYVGRQLDYETERHLYDLTLEVDDGIYK

NTTRVNIHVSDANDNSPVFVIDPYIITDVVEEDTSITATNKRFLVQVNATDADIDRPTQI

RYSLLGFGTSAPNKYFEIEPETGRLYLLTSLDRDLPNGRPNYQFNIRAQDEPDPLPGSGD

LDQPQIGFASVQVKPIDINDNKPFFISKLEGSVMEHSDRGSSVMTVVAIDYDEGINGTVR

YEIVEGPSDGSDMKFQIRENSGLIETSTNSSGLDRETTPKYTVKVKASDRGVVAQSTEKQ

VTINLDDKNDQIPVFEKPLYRVTMSEAQTSGAIARVRATDNDIGENARLDYRLNNFISFF

DVEGVNNEGSIVVFRAVDYENPDQRFFNLTVVVQDPDPSHTATTHVEVTVEDFNDNAPEF

ERPRIYVEEYEDAAVGTFLAHFSADDKDSGINADFEYSVDRKTDPDKIFAINQLGNVTIR

NMMDREKLSYHKVFILAIDKGDPAQTGTATLEVKVLDVNDNFPIFAKPYHPVVMENAAVV

PELVIVVNATDLDADPFGPPFGFAEPVCSDGSNSCPCNSRPTCADFSLLFKPSGDNNKGN

AEVSTKKVFDREQQKFYYMPIVMWDMRGSGNPKAQTGTNTLTIEIGDLNDNKHGPGHKDI

FVYNYKGMFGPLEIGNVYATDPDDWDVVDKTFTYTGPEYMKKYFTVDTNTGMITMAKGIP

SDVSYSFTVDVYDVTWKITAQCTVTIMVRDISEEAVFSSGSVRLRGITAEDFVARPKKPN

SGISEYEKSKYDLFQEFIADKISVPKENVDVLSVQNNGEFTDVRFSAHGSPYYPSSKMNA

VLAVNKDALQKAVNIEVDQVPIDECKEERFEGSCYNFLNVTGQPAMVNTNGTSFVGVEMF

VQPTQGCRSDFYPPIDDCTPDYCYNGGTCNKDDWGELSCKCSPGFNGPRCQGTHHSFDGS

NYALYDPLEQCEFGRTSLYIITLEENGLILYNGPVDDFAPGSDEPTDFISLELSGGYPRL

RIDHGSGTLELYVDGRDAHGIAKMQKLSDGKWHRIDIVRNGRDVEMIVDHCERATLGPGG

SVEDNKMCRAAGTTPGENIYLNVNTLLQLGGRYSAPNKPTGIMPTKFDGCVKNLMHNSIL

YDLTVPGNPGYPAGENTCRREDVICDENSINGPKCGDNGICESTWQPDTTICICKPGWFG

NKCETVATTKDLGEDSFIQYRLKDQFFNNVSSRVMKMQILFRTRDRDGVLWTISTSSGSM

TTTLQIVDGILTLTYNLGNGIRNLELSRRFVSNGQWHTAQVERNGREFVLKMDNMEGANY

NFTYGFPDDKQELHMHNMMFAGAIVSFTNSLPVLASDLQDTCLQDIRLNNEWFPMEVSEQ

ALSKAAEIQKVSHVKDGCVRNDCAGVTCPPDRVCYPLWGIYECRCPEHFEEIDGSCQRIN

YCADHRCFADATCEEDLTEEKNYVCRCSRDWTGEFCEQRQPAGLLAGITTGAIVAIIVSI

FVLLVLAVAIILLVKCGRRPSPDDKYILEVDPEDDIRENVINYDEEGAGEEDHDAYDIHR

LRKPDAESLHKPPLDTMPRREMPRRNAPGESPDVGNFIDDRLVDADDDDNAPPHDAVREF

VYEGEGSDAGSLSSLNTSSSISEQDYDYLNDWGPKFAKLADMYGAGMEPGEEDV*NVDVC

DDA*EYPQGTVPYAGNMESHLINDFWWRLFTQVAYNGTILLTNSALIPSGEDFAV*LE*R

MKLRDEWILKLQQNLGTLFQDLGGLTSQNIIICHAY*FCKPLAIECCSY*MFCF*KHITS

F*HRCRITLVSAVNIIHSPIQILKQMRSSF*LLIPAFYQ*HIRYCL*SLLLLSEGIYP*K

MLYIFVFVV*YIYLRHPA*LVMV*ATIDAVTSCCRVCVQ*LFICLSLDFRVLGNNLIKKA

EGV*WVVFAGYARFDLNWDNIYTVAMCIIVLYVHNILYRTS*FL*PEFWKYFFLSRKVCL

SFR*EVSITHFPKYQKTQGLTAFLTSQKSAGITCFPDQSEVSSYYLLP*PIRN*LA*CLV

*VAQDFSSQLLLWSFISAQF*QSSKMFKFIFKTYRNWFSSFSFMFINCIFLEHYKHSLLY

KPLSICDSCFICVTFSX

>**comp105805_c2_seq17_3** len=1072 path=[21153:0-105 139:106-1047 23380:1048-1048 23381:1049-1071]

TFYFCSGQGFADALLVQHNVCKQWEYRYDQMFSYEIPLYKFKTARQSLKEHGMSIESLAR

KYSVGNLNGPDPEPLSNYLDAQYYGPISIGTPAQDFKVIFDTGSSNLWVPSKKCSLTDIA

CLLHNKYDSTKSSTYIANGTKFAIQYGTGELSGFLSTDDVTIGQVTVKKQTFAEATKQPG

ITFVAAKFDGILGLGYSSISVDQVVPVFYNMVQQKLVPQSIFSFYLSRDPKAKEGGELIF

GGSDPAKYSGNFTYVPVSKQGYWEFKMDGITLGSTTFCQGGCNAIADTGTSLLAGPTSDV

TKLNQLIGATPLAKGEYMIDCNKIPSLPVINFTIGGKVFSLTGQQYVLTVSQAGTTX

>**comp105893_c0_seq20_6** len=3250 path=[14483:0-32 16318:33-35 16321:36-54 301:55-94 341:95-133 16455:134-136 383:137-184 431:185-234 16521:235-241 488:242-384 16628:385-405 652:406-426 16670:427-428 675:429-436 683:437-565 812:566-567 814:568-649 896:650-711 958:712-822 16780:823-832 16790:833-916 1163:917-925 1172:926-1447 16947:1448-1458 1705:1459-1459 1706:1460-1469 1716:1470-1483 1730:1484-1621 17022:1622-1622 1869:1623-1707 1954:1708-1708 1955:1709-1721 1968:1722-1754 2001:1755-1790 2037:1791-1814 2061:1815-1820 2067:1821-1844 2091:1845-1869 2116:1870-1931 2178:1932-1957 2204:1958-1973 2220:1974-1981 2228:1982-1997 2244:1998-2006 2253:2007-2027 2274:2028-2045 2292:2046-2051 2298:2052-2180 2427:2181-2345 2592:2346-2369 2616:2370-2526 2773:2527-2551 2798:2552-2651 2898:2652-2690 2937:2691-2744 2991:2745-2795 3042:2796-2872 3119:2873-2922 3169:2923-2946 3193:2947-2969 3216:2970-2993 3240:2994-2996 3243:2997-3028 3275:3029-3044 3291:3045-3068 3315:3069-3095 3342:3096-3135 15044:3136-3249]

SGCVLYSSIAFHLYVKTQIISMKQVLQCYSEFSTLNHIRSKTPYDVQSTAAGDLISRVIG

NRSSDFLVSVDPSVGPKNRDTFKLVSNGAKLSITGTSGVAVAMGFYYYLKYYCGGQYTWA

GQQVALPAVLPAIASPGKTVTSNDQFRYYQNVCTVSYSFAWWDWTRWEREIDWMAMQGIN

LPLAFTGQEAMFQRVYMAMGFTNEDLASHFGGPAFLAWARMGNIHGWGGPLPITWIDGRL

ILQHKILKRMRSLGITPVLPGFAGHVPAAITRVFPQANVTRLGGWSNFRQNYSETYLLDF

NDPLFAKIGGSFIKAMEYEFGVDHIYNADTFNEMSPKSSDPAYLASAAKGVYNGMLAGDT

QAIWLMQGWLFMSQGFWKPPQIKALLTAIPQGKMIILDLISELYPIYSRTESYYGQPFIW

CMLHNFGGTMELYGALDKVNKGPSTGRSFPNSTMVGTGLTPEGINQNEVMYEFMNEQAWR

PGPVNLDDWIDSYQKQRYGGGDKYTSLAWSLLKTSVYNSTSGHMDFNGVLYTRRPSVVMK

EFIWYDPEQLYQAWDNMVLASKTPALGNNSLFTYDLVDVTRNSLQILSSMYYHDIIAAYN

ATNTSQLLIAGQKMIDLLSDLDTLLASDGHFLLGKWTEGARKFSIDVNDTILYDLNSRNQ

VTLWGPTGQIRDYAAKQWSGLINKYYKPRWQLFVDSLVNSTKTGKPFNNVEYDQAAFSTV

ELPFSNDHTSFPTTVTGSSVDLAAKFHQKYRSETKSKFFSQFPRKFDRNTAFSKLQARQW

GRALA**A*GCNYITPKLP*LRHV*RYNLIGWWRCVMQTLRLS*DHKCVQDFHHFKEGYF

CNKFYNTIFHVIRLKDDILLPLPQISQGIYMISMKG*HLK*LVSHEILITLKTPWYI*VT

FTVKKLVITCVILKNHKCILRLKKQVSLSFKINSIS*VLYFHGALVEKFHYVSLLALGHN

QVAIRLHHQILLAPCYQ*GCVLVNNNNN*FIERLFPKDIAQWRYKEKRL*TMSVGKKGCL

NR*VLRAVLKASVGSISFRASGRMKVK*IKNIVLQ*IVIQVPVL*YIGNLSVTQTKTSYM

FFT

>**comp105913_c1_seq27_5** len=3282 path=[13671:0-6 13678:7-243 13915:244-745 3386:746-970 3611:971-986 3627:987-1033 3674:1034-1050 3691:1051-1075 3716:1076-1152 24572:1153-1156 3797:1157-1359 4000:1360-1433 24680:1434-1761 4402:1762-2294 4935:2295-2385 5026:2386-3116 18203:3117-3281]

RLLTDHRHLVQKVLTAVLNETFNMVLCPSLPPSLSSSSSSPILFLSY*LLITISEESAES

EPAWKGSGKEVGLQIWRIEKFKVVHLPKEDYGNFYSGDSYIVLNTYKEQDSKELKFDVHF

WIGKESTQDEYGTAAYKTVELDTFLNDAPVQHREVERHESTMFKSYFERITYLKGGIATG

FRHVKPEEYKTRLLQFKGNKKKVQVTEMSVVASKLNPNDVFILDAGRMLYQWNGKDCNKD

EKLRAVQYMQKLKSDRHGKASSESVEQGDEDEDFWKHFTDDDDDDADEEDATEDGVKKLY

RVTDEGGSMKTILEKEGQIFNKGMLDVKDVFILDTTKEVFLWVGRRASTNEKKNGMGYAH

RYLQSTCHPLVPVTVIKEGQASQGAFNSHFSAAA*NVITT*IARKMRT*CVRYFR*T*HC

GIYFKDFHNFKMTLTY*PISVHDLDMIDMQVKVINGIIFHDLVFDFVSYPETISSPCYGS

*RSVS*GMQLLVTYRLRTSLVQYFFFILLCLNQYTCNGIHCYLSSHRIYTSVYHKWK*YL

*DLVETKAHSMKYTTFIFLF***QFIRFSF*WIWN*LSHCNSITYHIAMYHNERVISQRN

*NCSKQTTTSCFLTSAMGRFESSKQGI**HNSVETPNDAVLFSLFS*EFPRKPKV*K*LK

ELTLLKISF*PCLA*WGSLVVRFPTCIQCVKPISWCHLHAIDGMVLKVAKN*THSLLLCI

LKC*KHFRINTFCNYYLISLLYILENG*IILLRKKYK*TIMTVGYSNFALSYSGIELSHT

TY*HKGTDKRV*STAQVVLICVRYQLKNRMIYSLNQGVVVQIDLSAKVIVAPLLYHRLTT

VVALWLLCTSARNTICYTYCYTTCAVMAIEILPRVFNVVNDNF*RHMPVKSSCVTA*IIN

SAKITKQC*LLLSRCPGNDNSLLQ*K*DCLKMV*VFASVKMLENR*NQ*LSLPTPKFLEL

IFLSNIKLLNWSLKFFDCTVLLRFKLLCNTHVNMFRYSCCFSQHQNVRLKLGPCFTLEFI

VSCTSSPHPTIVRNILMELVKTIVVLFRKPLLV**QIYL**NRLMFMHYWVDVTKLKWAN

NSPTDVYYFFIVCX

>**comp105920_c0_seq2_5** len=7217 path=[12041:0-2087 31292:2088-2101 14143:2102-2102 14144:2103-2105 14147:2106-2862 14904:2863-3283 15325:3284-3434 15476:3435-3556 15598:3557-6067 4895:6068-6098 4926:6099-7216]

VN*TYINLRIIARSCAFQGKFYNWKPPFATTP*C*PYDSIVEAEFANFEQFARSKTS*NW

DRAPDAY*EELGDGRGDSSDSGLLRRTEGPSDNY*LASENDNLLVKMACCLTEELKEQKR

INQEIERQLRKDKRDARRELKLLLLGTGESGKSTFIKQMRIIHGAGYSDDDKRTFIKIVY

QNIFMAMNAMIRAMETLKIAYRDVNSVNNQTDNAMLIRNVDYETVTTFEPQYVEAIKCLW

ADQGIQECYDRRREYQLTDSAKYYLNNVDRIAAPDYLPTLPDILRVRVPTTGIIEYPFDL

DSIIFRMVDVGGQRSERRKWIHCFENVTSIMFLVALSEYDQVLVESDSENRMEESKALFR

TIITYPWFQNSSVILFLNKKDLLEEKIMHSHLVDYFPEFDGPKKDAGAAREFILKMFVEL

NPDPDKIIYSHFTCATDTENIRFVFAAVKDTILQLNLKEYNLV*RRVEPGSLKLAINVFL

*KCP*SLIDS*CS*FSQYYWCRHYVKSPLTCTVPPPSPPLP*ND*FLCVRCDKRLSLHLS

RTCHRHQYRWMFVILSTVPVSGRPR*QFDVYRDCSAWNMGRGGRDGMGMRY*SHVVSCSG

VLAFSDSTELPLAQDPRHSISQPSLNLFLLLSQCFCFHLTASHV*HPV*LADAKWQHLLL

LLLNTKPWTTFCHFDNSTTQPPLSYPVWVVISRTEGTIQEMSKRGREDGERASRT*RLFL

SATDLFPAVLLAPKFA*RACLFVSPHRAEPAHQRAQDHSEGLAGMWEVNLDPPVSSQ*QV

HRRGRREIHTDFVPLKVYTGARYGSSTTPARRTLPICRYRKLYIAFNSGQHLCLFLTGKC

VNFLCLWCQLVFISKYNSSVTVCSEQYTMSPFCLPF*PSSTLSTVDRVRRPNYSNWTFLL

LWCISPST*IANTDHY*P*E**CMCKRGLYSNCCVKSSCV*LCCHNFHTTWI*QAVTRF*

YILTVYV*EK*C*YFDVQMCECL*LLW*I*L**LLLLLLLL*L*WFIAVEIYQGDLIQDV

GASWSMLLHGCGVGTQRWDSQTEGLCRCSSTDQVYLHAGICRNSLPEGVPCRQD*HE*HK

PKYLSCDRHFMLTF*KWLYLAFNN*LCPGLMIHVEGEWQHPHVVRAFPIDQKTKAICRPC

VD*PLTLQYCHRLLSLQPLTAPAVGGKPVIAIFVNNFWFERN*YCFVS*DIHIS*TYCVF

G*ELSPFDFFTLIELV*NFEKGHFMLRLELEIAS*HICYFPLLKRGRGDKFERETILAKK

IVMYVIYVCMCN*SCAP*LLDVLYECVCMWPSLRCLSPYRSDSLFWRKKDLMG*IGTVHT

QL*PQACILSTGFSKL*GSIQTLRDSLRSVSDSRQTDCQACMG*NK*FAGSTSSNKNTSI

VQSSKPYQNVLLTLAIQSSSYLKLT*FVNTFMAFMLKTPN*GNIYC**IFANENDVLSNE

HLLLKLFRLFFITDYRIQLFTRTMLTTPYCMFFVGMMTRVFLLYVVVFLIGFNRAEFSRI

*KPEHLYVCVHFISELIKYECCEP*VASACQLL*VETFSNLRNCLWTRQNCHPQTMCTPS

PG*PGRGELVQRLLADCCHGDVKVHMAVF*LLQLHKKVDLIHLVKCATGRLLPVIRCLYT

VCVPVFNTSRERASCMYSDSP*YICV*IYKYLLHVCGRLSDCAPF*NNYL*QVEGK*RLF

YTEHSYDVLN*ATGIYRCYHS*SP*GERYRMYSVAPHTSPFSSWSPFRFVGHIGGFFTSE

KGRWFVLSQLLVSLEFWVLEEKMIETLPRTQLAFHLCCLNGLMCGLVQLTSFDHD*CLLM

ECILYLWLISGD*VIGPMASVAARVLISLFN*LFVIAWGTGVSPTEFYRVVSLYTAHFCC

HCQNVVWKIGHCFETMFAIGFE*SSLNVNVKIVSDLEKHDEASLYCGIYVSDNAMLL*LT

*FNPRITTNN*CLALSLFVNSCVATMDLFIIGSRIFLYASVLLFNSVTVIL*QV*HVWLN

GPGATYISFLLERPGFSSCPTLFGSSLDWSGAPDGVT*SMLL*SDMLDLSCSGAYKAKER

LSG*LIFEHVGTI*IIWAFCIM*ASLEVFETGLF*S*GKVVSDLQIANDVLLTLDPPVLQ

VKLGVPFQNLQI*RETSVT*FWKMP*WMKSFYKSPNK*ARNSGTRLWTKECWCERTRKVS

WSQ*RVLLGRMASWAKHGVLVTMNLQNVVS*DKIGIMTPKDRCRGYCAIW**LFRAFCWL

SCPTIEKVSPHF*VHNLTKCLT*ISYFMVFCPHIIQMRSQKDVCIFMVSWNF*IPDLLMA

IVLAYFKLEIGHKLGGIESVNVQLTTYCKFVCNMKCLFII**LEVCSI*TAFCRLIYKYC

DLEAAVSAVSLPPPVVSLGLATCGCLSSLLLCIKAQVCTIWLLHSVHFLCKCICTTVHVC

KYVCMX

>**comp106047_c0_seq6_2** len=4887 path=[18864:0-70 5108:71-122 5160:123-165 5203:166-231 21452:232-236 21457:237-270 5308:271-276 5314:277-311 5349:312-357 5395:358-473 5511:474-507 5545:508-632 5670:633-808 21723:809-812 21727:813-819 21734:820-840 5878:841-977 13712:978-981 6019:982-1074 16226:1075-1084 21844:1085-1091 21847:1092-1134 16321:1135-1140 6178:1141-1250 6288:1251-1307 21984:1308-1312 21989:1313-1386 6424:1387-1424 22051:1425-1442 6480:1443-1503 6541:1504-1519 6557:1520-1543 22098:1544-1575 6613:1576-1790 6828:1791-1826 6864:1827-1885 22207:1886-2102 7140:2103-2109 7147:2110-2155 7193:2156-2199 7237:2200-2232 7270:2233-2319 22492:2320-2384 7422:2385-2403 7441:2404-2474 7512:2475-2497 7535:2498-3031 8069:3032-3177 8215:3178-3201 8239:3202-3614 8652:3615-3849 8887:3850-3908 8946:3909-3933 8971:3934-4183 9221:4184-4204 22994:4205-4439 22886:4440-4460 9498:4461-4886]

LIVIRRNMFCSSVQHYAPVRR*PRQGHWTRS*DDADMSTKSQGNFVQCPVCRTSHVPTNG

ALKNGVYASTCENCGHFFKFKATGPLKSSVTLTINGTSFTVGNEYAAALSLNEFMRSQGV

SYGTKVMCMEGGCGVCLVTTTLYDPITKAVQTYTVNSCLVPLYTCDGMQVTTIEGLGNPR

DGLHPIQDRLAQYNGSQCGFCSPAQVMNMYGLLKRTPKPTMQQVENAYDTSICRCTGYRP

ILDAMKSFAADAAPNLLGGAIDIEELDKKICSKTGKTCTGRCSSERQTDPEQVPLHIVLQ

GSQWYKPTTKQQLYALLGQHQADNYRLVFGNSGYGVYKEIGPWMYDVLIDLRGIQEFYNI

SIDSSKVMFGPSITLTNMLEVFEKNSTNPALSYFRDFASHVAVIANTGVRNLGSWAGNLM

LKNLHTEFPSDVFTMFETVGATLSIGSSDGTEKTYSLMDFLNLDMKGKVILSVTLPAFPS

GDVYVKTFKITPRHQNAHAYVTAGFRLQLDRSNNFLVKTKPSLVYGGISKTLVHATNTET

YLQGKQLGDPAVLNSAITTLSAELVPDTTPGLSSTTYRKSLAISLFYKCVLGMCGEKAAA

RFQSGGTNLIRPVSSGTQTYGSRKDEYPLTKPMTKVDATLQTSGRVQYVNDVPPVQNEVF

ASFVITSVGNADIDSVDTSDALKYPGALKFISASDIPKGGSNSFRQPGAGVEEIFCSGQV

LYSGQPVGIIVAVDQLSADTAASMVKVTYKNVQPPIITMEDAIQKKSIFPNVAQEFKVGD

AAAAIASSDKQISGRIKMGHQYHFHMETQVSICYPTEDGIGVSLLSSTQWTDLEQLIVAK

ALNIPESSVNVEVRRLGGSYGAKITRAHLTACAAALGTHVMRRPVRLSMNFHTNMKTIGK

RFPYLADYTVGVTNAGKLNGIQYTVNADCGMSPNDCSLSSFPQWMDNAYYCPTWDFKPIA

LKTNLPTNTACRGPGSTPSIFIMEAMMDNVAKILNMDPLEFRKINLFTKGQKSANGMVLQ

YCNITDMVAQLETSADVQNRKQQVTAFNQANRWKKKGISVMPLRWDLNYANANYNVYLTI

YHGDGSVAISIGGVENGQGINTKMMQVCAFELGIPMDTIKVKPTTTLADSNSITTGGSIT

SEVNALGVIQCCQQLKTRMAPVKAKMGNPSWQQLVAKCYSEGMDLSARSFTNPTGVSTNY

NIYGATCTEVEVDVLTGENQINRVDILYDCGESMNPEIDIGQAEGAFVMGLGYWMTEQMI

FDPKTGTALTAGTWEYKPPLGKDIPIDFRIQFLKNAPNPRGVLRSKAVGEPPLCMAASAL

FAVKHAIEAARKEISKDTYFPLDGPATVEMIQAACLVDNSQLVFGT*IRSAAPRDIDQLV

QET*ISMKYRISLRREVNPLMFEIQISFGELLGRKINVLACVCLVRVCICVAV*FVVKSC

*LLV*SDSEVFCIFHFV*VLIWLTALIIISQKDT*NCSTVTFSS*SKLLSRSSILIHIFI

N*RDDVMTQIYFSADPQLKYIKK*NTSLLSHILNNLRDNPAECEM*VCFDYDVMV*QSLL

KRRNSLCRVTSLDT*GAWECGFESLTILLGLSAPYRY*WVLIGDIPPCEADRPLYNSKRC

SKRC*GRWG

>**comp106275_c0_seq2_4** len=629 path=[8109:0-3 8113:4-49 5497:50-107 5555:108-140 15358:141-145 5593:146-156 5604:157-169 5617:170-235 7834:236-252 5700:253-254 5702:255-307 9732:308-326 15422:327-351 15452:352-352 15448:353-375 5823:376-413 5861:414-414 5862:415-472 5920:473-477 15528:478-498 15549:499-503 15555:504-507 7973:508-511 5959:512-517 15563:518-528 15574:529-536 15582:537-548 15594:549-554 15600:555-563 15609:564-566 15612:567-628]

FNLHQFGRGKVR*RT*DLCCFFLVLVGVLTPASIGVPLANDADDRQGHAEHSRQKRAVLT

AIKEVGCGAYTAVKECTTDLDSCGGFCDFVDSVCSAISHSGTAVSLLCDTNVDKYESTVM

DDYRQSSHGTPRDAERFIMKLGDDIEHLPKSFWEKLANFFKKLWKRICGWFSG*FS*LIP

DFDVTLNFVSLHLDIEHCCYV*VSE*GLF

>**comp106447_c0_seq14_3** len=668 path=[428:0-15 3717:16-16 3718:17-39 3741:40-75 3777:76-105 3807:106-129 558:130-140 569:141-144 573:145-159 588:160-162 591:163-163 592:164-176 3524:177-184 3532:185-237 3585:238-255 684:256-259 688:260-260 689:261-261 690:262-300 729:301-302 731:303-310 739:311-334 763:335-352 781:353-378 807:379-379 808:380-382 811:383-391 820:392-398 827:399-421 850:422-422 851:423-431 860:432-448 877:449-472 901:473-504 933:505-505 934:506-543 972:544-544 973:545-553 982:554-556 5073:557-589 1167:590-592 1170:593-600 4260:601-611 3208:612-615 3345:616-667]

VALNKPAWMSSAWDGNHPASKAVDGDTSAEHEIYVAHTGTGQIIAWWKVDLQTQVQSARI

ILYFRTNYKYRRNGVHLYTSGTNSCEPTEGNLCHTVTGRADGTDIPDVLNVTCPGTWRYL

TVYTEIDNDNGGVILDFAEVQVWTCSVGMYGVSCNKGCNSRHCDGSTESCDSFTGACSSG

QCQTGWTGTDCAVCVDGKYGPSCTRDCSSRQCKVFFSTCDHI

>**comp106499_c0_seq7_3** len=1147 path=[5605:0-45 8881:46-47 2649:48-52 12654:53-94 12683:95-99 6296:100-124 12713:125-130 2732:131-154 12747:155-165 2767:166-210 5928:211-227 4731:228-255 12823:256-276 2878:277-348 2950:349-372 12901:373-381 12910:382-395 12924:396-396 12959:397-406 8693:407-408 12937:409-531 13021:532-762 5136:763-854 5228:855-859 13205:860-880 3482:881-913 3515:914-968 13249:969-976 13257:977-983 13264:984-985 13266:986-1038 13319:1039-1039 13320:1040-1047 13328:1048-1059 3661:1060-1060 3662:1061-1096 13366:1097-1101 13398:1102-1102 11956:1103-1146]

TQHCGTVYGRYNFTSLGPELPVRYHHSATAVPGTREVYHIYYSTRPVSCNVTLTETAGYI

NSYTGYGSSPVYSRSNNDSAHCIFTIKGDNAVGQTIVFRLDQFQLEDRDNSTSLCKDYVD

MNGEERLCGYQYGNRSFSFDGTFIVTFISDNSQTNMGFNIHYNIVPDGCLVSIFSTEGTL

RSPVTSNGLYHNNLDCRYTLSSPYSMAKITVTVNRFDLEPPSYSSTNCYNDYVYYTDGSY

TSGRYCGFVAAGTTFIWYTTDVQFDLVFHTNNEISRPGFNVSYKFEEDRCDQVLSDPIGT

IRSPVYSTGLYRSNLNCTYIIRPPTQGPYRFNLTYSQFDVQRTYYNSSYYSLSTSCYYDY

VETRFSGLTGTNRLCGRMSSAX

>**comp106832_c0_seq1_1** len=13721 path=[13699:0-1807 15507:1808-1811 15511:1812-2199 15899:2200-2202 15902:2203-2228 15928:2229-2245 15945:2246-3199 16899:3200-4277 32933:4278-5358 27456:5359-5371 19071:5372-6317 33164:6318-6341 20041:6342-6350 20050:6351-6419 20119:6420-6489 20189:6490-6496 20196:6497-6564 20264:6565-6674 20374:6675-6676 20376:6677-6758 20458:6759-6763 20463:6764-7955 21655:7956-7967 21667:7968-9160 22860:9161-9184 22884:9185-10112 23812:10113-10808 24508:10809-10811 24511:10812-11273 24973:11274-11276 24976:11277-11712 25412:11713-12105 25805:12106-12610 34090:12611-12616 26316:12617-12630 26330:12631-12675 26375:12676-12731 26431:12732-13049 26749:13050-13143 34306:13144-13720]

LETIS*AVGDNPTCPTRYSKSVEVPLRVLL*PLKLNSLVELSQKPPACLLKIGYRFYLAS

LTRYHA*GLPSRPVRTLKRLNTGI*QSVSGGAVYIQHGAVNEHLVNTCGQGTTLTYCRFS

GIFQRWQFSIVIIVGFKCQ*LCETRQGPSTAMPGVYLWVSALVVLIAVKTSVQDDVPLGC

QSGWTLVGVQCFKLYKGIVPWESAASVCKSYGAEMAKVTDYYENEDVGKFASSQSTDKFW

IGFTRANAAAGNQRIGYWSDGQETSIAAGFWKNDQPNSQDGNCAYVEKMDRYYWSFGPCE

MKLSYVCERPACPTGSYHCTSGRCLNQNLVCDGEDDCGDGSDERNCAQRCKYYYMQAAGS

ITSMNYPSNYINALNCLWVIEVPLGQNVYLKFTNFSTEMGADIVQVLVGGPTESQGQLVA

TLSGDRRASLPEYISTNNFMIVKFTSDSRSTALGFRAEFSGRSANLQPYQSLQATDTPKT

LIAPNYPSMYLASQDYTWIINTVMQTQIITLNIVEVDLKGMDKIIIRNGDSVKANLLYTF

TADTEVDNIPFIFSTGNSMYISMQTRGYQVGKGFRFIYKQGCDMMLTSMKGRLFSPGYGL

DGITGMDYPNDITCNFRVNVPGKPKITLISQGFKTQAGADVVTVHYGGASPDTLSGDSPV

PSRSNNGEFNVTFETNAIIREKGFLFEYSIDCPNPNFNDKTVLTPSNANWQFMSEFTVTC

KDGYRFATQEYQDTTKSPGFESFASTRMKCTYGGKWDRETTPNCQPKYCGPAPLVSSAFV

NSSMGQSSSVGGSVTYSCYPGFTLTNSATIRCRNDGTWEPTPTCTIASCSALPTTLNFGS

VTRVQGDGTGFGSIMKFTCNAGYEISGDAILFCTSGSTWTAPVPSCIPLSCPIPRIPHGR

MTNRQAAVFRDVRSVVCDSGYRLNSTSNQVTCGANQMFTNLDSMACEDIDECPSPCAHNC

MNTVGSFWCSCRPGYTLHFNERTCDDVNECDAGNGGCNQVCVNEPGSYKCQCNQGYQLFT

TNGQLNYTLPNNEDGTMAGDIYRYNHTCVRIQCMDPPPVANGYILNNRQYHRYQDTIIVS

CNIGFNLIGQSLRICQDNGQWNGTAPTCSAATCPAVTIPGGLKTTPSVVPSGSVNYLGVV

NISCNVPGRAPFLKTRQCLLDPSSKQYKLFGAQLECGLIDCGTPTGVPGAVIPAGIATTY

GSQFEFVCRNQYTKEGASSLSNNTVQCKSNGYWGWGTLRCLGNTCSDPGRPKDGTQHATS

YEENTQVNYTCNRPGYTLVGATPLTCILNNGVLQWDRSVPTCLDTQKPTFSNCVAEKTVS

KYTTGLNAVTPPTPSDNTAIMSMTVTPTWPSDKLLIANEEDFVFTAEDFNGNREICTTKV

KVLDEVPPTIKCPDPIVEEFKEENEYKLLQFMPSLLSEYSDDSGITPSVLFNPPQLNISA

AFINQANYVQWITATARDAAGNSNSCRFQVMVKAAICSPMNIPRPVNGQKSCTALSNNAG

YTCDLTCDANYAFYDHPNPTSVTTSCLTGNPFPLAPTPACSFTRSETAKFTQVIRFIYNA

STASPLADTCKQQYATMMTPVLNGQRTRLNSLCTTLGQVPDLTFLIANPQNDININTAKN

QVEIDYTLIYEGSNTGVLSSCATLVDTILKDFNFNQTKIIAGISSQLIPVSCPSISSSTP

GTETFRDFSCASNRKKLSLGNLVFACLRCPVGTYNSGGNTCTPCPVGQYNDIEGATSCKD

CSTAQWTSRVGASSNLMCRPQCNSGSFSATRLPPCQVCPLNTIWVNTTYCQPCGTNEITR

VYGATVSTQCKARCQAGTYNAIDGYAECTTCPKNFYQDQAGQKTCKACSTNEVTAGSGST

SQSNCTSGASSAECNSYCQNGGTCDFIRHSPSCTCPKGYSGGRCENGDNPCASSPCYNGG

TCNVLNVTTYSCNCPPGTSGARCETDLNDCTSGTLCQNGGMCQDQINSFKCLCKDGFTGT

LCTSPQPICNSQPCSNGVCNPIGSFRRECSCLDGYTGKNCETIIDNCADNPCLYGGTCTN

GVNSYTCQCPLGFTGSRCETRLNKCAGVTCGNRGQCIEDHVENTYRCVCSPEASYGEYCE

YSLSLNKKLETPTSSSNDVGKTLMDCRDTCNSQGATCRAFSYFKTEQRCMLHSTRAGNNL

PLVDAQGASYYVKKCSYIKDNFYTEWYNTPDNRTTGNYVLLNDMRNTLQLDICGGSFPVA

IECRDNTTKKISTNSGARCILNQGFLCDNSQQPFGNTCNNHEIRFKCGVSRVFKEKTCTI

PPYCTSSPCMNGASCRAVGLTFACDCVPGYGGSLCQNDLDDCASTPCLNGGICTDRVNTF

TCRCAPGYEGPTCAGTVNFCSPNLCNSTGSSRCTSLTTGPQCHCKPGYDNPVCSNNIDEC

ASSPCLHNGVCTDGINDFTCGCKAGWTGKRCEVLITPCSSSPCKNSADCFDLFSNYYCRC

KANTYGQNCQYAPSVCSNANPCVTGMCSETAGVTKCACGQNYTGSGCDVQVQHCKAGTCK

NKAECSVPALDDYKCSCPKGFTGTNCDVNINNCLGVTCQGTATCIDGVDEYFCRCPIGKS

GTGCTQDLDRDYDMLFNLPQKTGYASLPYPIRLTANKFSVSLWVRFLEPSGTGTFFTMYS

VDGPNSLAGKKELIRIDHSGLTISVNGTSTNVRLQYFNFNNGKWNQITVTWNGVTGLLNF

VVNTIAQDVQDYAKGMTIDKYIWMVLGSKYNPVTEMPVPNEGFNGWLSQVSLYNRDLTFT

QEIPLKLENPRQFFADEIFAWNEFRRSQGVSLVIPSTASSTSCPRGYSGGNCEITNERKL

LVSPTKCPSDIVKLGTSRITEVTWLDPEFAGKDSTIISTHKSGDVFLWGRYQVVYLASNS

AGNKGLCTFKIFVQYSSCTPLQNPIRGNAQRSTNNDPLITTSIDCDSGYSIVHPVPRLYT

CPRIGSYNPPMMYNTFRLPPCGVIVNNNLYQVIIQLFYTITSTFPSGIETSMRTEILRAL

TTLNAEWGNGLCDKTDCSDAVINIRSGVTSGRRKRQSTLDTNVNITLPMAKALSTSGSLS

LSPGDILRLAVLNQDKFDFSRNIPNAIPKRDLLKILLSISCESGQAVVNGKCVKCAPGTY

YNAATRTCDLCPVGQYQPNEGQSGCVTCGTKTTELAGSANANDCKVTCTTGHKFDYTSDK

CVLCPIGFYQNMTGQFFCYPCSVEKTTRGTGSIAEAQCYVGCTKGNQLAPNGTCQACPLG

KYRSATMTQCEDCVSDLTTNSTGADDVSLCNVGDCQAGFYRNISKQTECLKCPRGTYQDL

KQQYSCKPCGASPEYTTPGDGSTSSAECRFVCGDGLQRFVQQSVDTCIPCPVGFYRNMTL

LPYDVCASCGLGRSTPSTNSKSFSDCSILKCSVGEQPDTSNSQCVNCPLGTYQNQANQPN

CISCTGQTSTRQNGSTSSTQCEAFCNGGQEKLADGSCRTCPLGYYKDNSVYKFTSCQKCP

ANFVTSAPGADSVSKCNVYQCQAGFKIISSGCEACPLGYFQPEPYKSSCRKCPDNTSTRQ

NATVNATQCEAYCQSGSEKDATTGLCTPCKRGFYKANSDGVFNQCVMCASNFITASSGST

SAADCSVANCTAGQKTISTGCEDCPKGSYQPNKWQPDCTSCTTDKTTTSTGSKSATDCIL

SCPPGKEDIAGVCVECKQGFYKSEQKASQCTACPANKTTAGNGSTDSAACNLIGCPVGHY

QDSSTSCEPCGYGFYQPEKWKSLCRKCDTGSTTYRIGAASVTDCVLECPKGSQFNAGTGR

CDPCPQGYFQDKLNPTVFECIKCGDASVITAGPGATSAANCTVRNCTTLGQFRNSTTNLC

QDCRIGTYQDTKWQDSCKMCPTGYTTRNTKTPSSAGCLRDCDAGKTQVGDGCTDCPVDTY

RNKTTWTCQACPTGLKTVKVGAASVSECNVSACLPGTYYNRIDSRCADCPINTYQDSTST

FGCKECPSLKFTRTVGSRSPSDCIGFCDTSNNCSANAKCVEDRITKYKCECNSDYQGNGL

TCTHLCDTDYCQHGSTCSRGPPAQCLCTEFYIGDQCDIRRAAELVSNQETLTIVGLVIGI

LGFILLLILIAACLYRRRRPTPPRQAASEYDDRTSLASPRGPYDYPLAYVPSKAASVISG

PRYMLPQNVEKSFDNPQFIISDDDPAVYKA*Q*LRQDLQGHSHLSSHIFCSPVNSICL*L

HTIPFFLQNDTFQDLMQLNSLEMFIISSSCRMSREIN*LTCLSF*EIMLYISIVNPFFHL

VYN*YCNLEIHADVMNLNECTNSVCYALRTLWHHILCTQHARRA*YVHSFATVKLISFT*

SVFIKFN*KYSLLKEPCKYHLHQLWMNQQMFFL*YIIPNETLHFLYK*IVLVCTFLLQKR

NLLK*NIMLPISMNQRPH*IQR*WLMPQVCDITFWCKICIYCEYSKNRRDYTMHIKCQK*

Y*CVIKAHCINCTV*NCLAKVIF*LQYHGPSQMYY*HLETTNPWLNLMSSKILWKLILLY

SVYLIMDLT*QKASQLDETEKPLFFIERDTGLKIILTVCYKSNQRLMPYIKIIVY*PSLI

SPNSCIVNQVLWIX

>**comp106854_c0_seq4_3** len=3390 path=[3371:0-258 3630:259-359 3731:360-403 13182:404-411 13190:412-412 13191:413-430 3802:431-482 3854:483-515 3887:516-539 13252:540-556 13269:557-579 3951:580-581 3953:582-606 13312:607-655 13362:656-656 4028:657-664 4036:665-731 4103:732-751 13378:752-850 4222:851-976 4348:977-1002 13455:1003-1017 4389:1018-1018 4390:1019-1020 4392:1021-1108 13516:1109-1114 13522:1115-1183 13560:1184-1236 4608:1237-1280 4652:1281-1322 4694:1323-1324 4696:1325-1349 13684:1350-1356 13691:1357-1365 4737:1366-1386 4758:1387-1409 13713:1410-1419 4791:1420-1426 4798:1427-1471 4843:1472-1487 4859:1488-1494 4866:1495-1534 4906:1535-1537 4909:1538-1561 4933:1562-1644 5016:1645-1645 5017:1646-1669 13842:1670-1764 13916:1765-1765 5137:1766-1834 13929:1835-1845 5217:1846-1879 5251:1880-2026 6910:2027-2101 14078:2102-2108 5483:2109-2363 5738:2364-2477 5852:2478-2496 14186:2497-2531 14221:2532-2546 5921:2547-2556 5931:2557-2561 5936:2562-2649 6024:2650-2699 6074:2700-2736 6111:2737-2778 6153:2779-2785 6160:2786-2821 14328:2822-2836 6211:2837-2843 6218:2844-2857 6232:2858-2893 14366:2894-2896 14369:2897-2978 14406:2979-2995 14423:2996-3025 14453:3026-3057 6432:3058-3112 6487:3113-3136 14473:3137-3389]

YNNIHNPH*IKISSFAEQLPQITTAVHQQTPTCT**YSWVLGCRQPIRQQEPRLTNQWI*

ATPLAAAYTYTGG*NWLFVSCSCQYGSIYLHICNVHTVFPMRYQRRWSNIPKGDQERQQP

PAVFH*QRRIRDLPTNFILQRYRYSHNFRRFNRFNTMSVTCTHVALLLLTTCWHVQACIT

ARNEIQEPVIGIDLGTAFTCVGVVRNDQVEIIPDQQGNWITPSYVAFTPDGETLIGHAAK

NQMTTNPENTIFDVKRLIGRTWDDPSVQRDIKRFPFKVIHKENKPYIEVRVGSEDKVFAP

EEISAMLLSKMKEMAETYLGENVTNAVVTVPAYFNDAQRTATKDAGTIAGLNVLRIINEP

TAAAFAYGLDKLDGETNVLVFDLGVGTFDVSIITIDHGVFEVVATNGDTHLGGQDIDQRL

VEYLIKTYKTRTGQDLLEDSGAIQKLRREVEKAKRALSLQHQTRVEIESLMGGHDFSEVL

TRAKLEELYVDLFESSLNYMQSALEEEYLNKTDIHEIVLVGAASEIPKVQQLAMEFFDGK

APKKGIHPEETVAYGAALVAAFYATYDETKDECFDIMEINSLSLGIETVGGVMSKIITRN

TAIPTKKTQQFTTIDDDQDVLEVLVYEGERAMTKDNHILGKFELKGIPPAARGDPQIDVT

FEIDVNGILTVTAEDIGTGNKNNIVIQNDENRLSPEDMEKMIHDAEEFAEEDKRVKERTD

ARNDLDNYVYSLRNQINDKSKLGGKLGTGDKSTIEKAVAAAIKWMDSHEDASAEDYQKQK

TELEDVVRPITSKLYEAAGEEPEEEKEDKEDKSSHPHEDL*RSV*SYLPSTLFNVIRIHS

CHSGYGKARVCMKDVR*WWCILDIRHHSHLSVNSTKLNNTSWS*CGMICILIAF*NVTVL

YLNVTVLYSNDTILYSNIMIEANKILSLFS*ILNLWTYLFYRKDHPLHLILHIFQ*HLEV

IHKKGKFKGQLL*KCFSFYKS*HP*ISHSDP*CLI**S*MCNEDEVTEDNFMSFLDKLQI

SCLLFRVSDMKPTISFHSQACVAKICTYTHLTLCHGMNAS*MSTRAGKIDR*SEMSSIIK

*Y**ISI*SLCYQ*YHTLTFVVEKSFFHKVSFCIHGLFLVSTM*R*IITX

>**comp106862_c0_seq1_3** len=6977 path=[6962:0-1296 20050:1297-1621 8584:1622-1642 8605:1643-1701 8664:1702-1730 8693:1731-1872 8835:1873-1882 8845:1883-1992 8955:1993-2052 9015:2053-2092 9055:2093-2112 9075:2113-2419 9382:2420-2431 9394:2432-2457 9420:2458-2477 9440:2478-3169 10132:3170-3184 10147:3185-3666 20548:3667-3716 10679:3717-3856 10819:3857-4580 20725:4581-4581 20726:4582-4593 14279:4594-4594 11558:4595-4598 11562:4599-4621 20755:4622-4732 11702:4733-4855 20905:4856-4857 20907:4858-5076 12046:5077-5082 12052:5083-5135 21046:5136-5142 12112:5143-5159 21056:5160-5597 12567:5598-5654 21164:5655-5705 21202:5706-5756 12726:5757-5771 12741:5772-5814 12784:5815-5860 12830:5861-6015 21351:6016-6030 13000:6031-6037 13007:6038-6063 13033:6064-6215 13185:6216-6321 13291:6322-6330 13300:6331-6378 13348:6379-6417 13387:6418-6550 21590:6551-6623 13593:6624-6629 13599:6630-6643 13613:6644-6700 13670:6701-6753 21705:6754-6790 14628:6791-6855 13825:6856-6879 14716:6880-6906 21770:6907-6976]

KQNRTVPCGPRTDNSPHDVI*QSTRKRNMLPSGLVSVLVVATFTTVVQGNYFIVSPSVFR

PNEDFKVEITYTGSGTASAKATIGIMGGSDVAASTEQTLTNGQGLLVVPISPNVEKGSYK

LTVVGKVNGQEVFRNSTEVRLVMENSFVFIQLDKSMYKPLQTVRFRVLSLDQELKPVAQS

IRTDISIVDGNNNKLEEWLGVESMAGVIENSYQLSDQPPLGKWKITAKHKFGEDTKEFTV

EKYVLPKFEVTAEALPDYIVNGETDAINIKVTSKYTYGKPVKGKAKFIFGKGEVVKETML

NTGEDGVHTEKIMMKDVSTAKTWNGLKVAVNVTEEPAQVTETSNQLNIPVYSQRVKLEFL

PTSSQVYRSGLPVQLRMKVSNLVDKTPAHTGKQLTVTTYLGSNQEKTLTIDSQGMASTDF

DIPPSYSNDIHFTARVVGEDGISGSHQVKEYRTKLNTVLSLEVSTTQAIEVGNSFDIAIK

STATLRHVAYMVLARGVIVEGGKLNMPGTGLTHTLTATRPMSPMAKVIVYGVVDNGTMSE

VIADSEDIEVSSVFDNQVGLRFSADTSRAGTTVDLTVTSAPNSKYYLLAVDKSVLLLGTG

NDITQGDVISGFHKLGMEDTPSQNEGQPEMLGDVIMPRRGGGSESDLPTSAGEVLKRLGL

VFMTDIIVEKEARQWEFERVMDADIMFKGGVPEIQHSPVAGVGAGAAAKPKYAEVGRTRK

FFPEAWLWKDGTVSGDGTVTVSETVPDTITTWVATAFAISAEKGLGITAQSSKIKAFQPF

FVSVALPYAIKKGESFEMRVTIFNYMNQSTPVRVTLKKSDDFGVEQTVNGVKSVVSKEVV

TEVQVESNVPKGVTFWISAKTLGPIKISLVAQPDESTGEKGDSLERVVEVKPPGIVQRMV

VGETVELDAETTLYNKTLMLDFPNDVVEGSRRVEVMATGDVMGPALEGLEKLIRMPTGCG

EQNMITLVPNIVVYKYLNAVNRITDEIGRKAIQNMKSGYQRELNYKRDDNSFSAFGQSDD

SGSTWLTAFVMKCFSQARNQITVDRDIMTKAFTWMKSSQKEDGSFRDKGRVIHQEMLGGS

TGERALTAFIYIALKESESFLAETDKSSLTRAVTYLEGLVDSNQLTGTYEMAIVAYALKL

AGSTRATVLLDKLEGRTKPWEKTVEPVNSGGKVMGRQFIYHPPQAAASDIETWSYVLLAY

VHGQQLAEGRPYMKWLLTQQNGNGGFRSTQDTVIGLQALAEYAALVYASPQTDISLTVKA

NAVSGTESKVFMVQRNKLLLLQSYVFPKDTTSLELTATGMGLALVKILWQYNTNTTVVKK

TDTKQIDVSVVTYKLQDDLYNLKGCFSSQQSNLGMTVVSFEMPTMSSLANEDMLAGNRDV

KRVDNDGDHVHLYFNEPRKEPQCVDVHVRRDAPVANLQAASVKAVVYYQPEVEKDVLYTL

EEGEDICNMCGTSCPSSCDTSAASIQRLHSFLLAGVICLLLSLILAL*PWKQLSDS*QGD

SRGCT**RT**ILKIL*LPCLFLFMYTVNFIENVLSTLRF**CRCFSRNMLGYLRRGVVN

IPLLSNTK*SLCFNLQNDS*FEENILKMWVLLLYNLLAVNIGHALVLN*W*NEDSNLFCF

NLETAVHCFRDLKIIINLTMTT*CTTGLSLFQPIGLIKTWR*MFNVAAKAFRESVITFEI

VLVHLELL*INTYSPIAGCPDTDGNKRVRLL*IRTTKMS*RLMNPYLKETDAFMYKMKLC

*CLWCLIKEKKTIIIYVPRQ*IAKYNIQPFHEDCD*PCSL*WTHLFDIYMYIFFSIFLTW

*WTLYEDQCMNDSE*SVFRIRRLTIPC*SAGHNFGWCLLAIFSCLYMSV*KYQKHNNCLP

LCLH*TVL*YYIKQH*GHYDLIMTVVSLPPVLVTLYVSSMSPFLHHVCFHSH*QD*GT*N

EWHAIISFKITPKYKYIHKYFLSHVMKKKFRISLLMFGPHHRNNLNDSTSETKVL*KY*F

VVAVS*ILWSHCLTVYVVMVIYVSLNLSFV*ISLSSMSE*VWF*AAVSNIPAIQPSG*SI

RLSCRRLSLNFHMGTVFETHFWCRLLEDC*KHIKPNSLSHSLKKKSVNQKSAPVIIKRNT

VSVTKQWIFHLHACLLKIILKIYTCQISF*YLRFSWCF*QGLLWFQLETQHQF*FQIPCS

GSTFCENINICSLRVLMESI*TSTSSQWWWDQAL*FTINTLQFVFEFSRCEIVI*LMRME

NME*KENGSLLL*VGPPATCVQSFRKSWNSKSFVAMLNSLFQFYQLWYQHVGLVYFLI*T

R*MMLTVLCVSIRT*LDYISLFPVYMTNVPLILRRHYFHGRWGSL

>**comp106997_c0_seq1_2** len=4332 path=[9723:0-82 4463:83-220 4601:221-411 4792:412-463 15133:464-519 4900:520-521 4902:522-566 4947:567-670 5051:671-738 5119:739-839 5220:840-1190 15436:1191-1203 15449:1204-1270 15505:1271-1351 5732:1352-1356 5737:1357-1369 5750:1370-1499 8781:1500-1536 15653:1537-1630 6011:1631-1647 6028:1648-2220 6601:2221-2440 15894:2441-2472 6853:2473-2730 16006:2731-2733 16009:2734-2758 7139:2759-2772 7153:2773-2824 16057:2825-2897 16126:2898-2907 16136:2908-2911 16140:2912-2916 7297:2917-2934 7315:2935-3126 8897:3127-3150 7531:3151-3280 16306:3281-3336 7717:3337-3350 16342:3351-3413 7794:3414-3414 7795:3415-4029 8410:4030-4050 16668:4051-4170 8551:4171-4181 8562:4182-4227 8608:4228-4331]

PI*PAASTLNPNTRSR*LWPPIPGRWREATSTSAPRETHKMATTPSAQAPVMLTCVLMLL

TCIQISQQQVSFYNTCQDASAEGTLRVSINEVDNQATINASPNEYRAGLGLAGNTQNIGL

QFDANNNAQLPPAEYFALESGPRSGSVTGREWFLRLKKPIDRDGDRPDSLDDNFVFEYTL

QCTDLTINTAFFMLLRVQIIDVNDNSPVFQNDPYAVSVNELTPIGTTVFRGVTATDLDFD

FNKNIVFDIIDGAGSDKFAIYIPSLGYVTVKNTLDFESLNSAGNTNYLLSIRAMDSAQPP

EVKRTAITTLNVTITDGDDQKPIFVYPTCYTDSQNRCFNPTYTASMVSGQLPGEISVFPY

PAANPNVPVNILARDQDTLNNPIIFSVELTEPRGYENRFQVTSEQVAGTSTYRGRLQLLQ

PIDRSVVGELRVIIRIQENSPNQRYNRAILYLTVTAANNNPPSVSTSIGSQIGYIRENDL

TGAYIKDQQLQNPLQLIITDADIAPGDTPQQYTFDVTPASPFYIDSNNYLRLTAGPLDYE

SVQQYTFNVIVREQGTAEQRSGSISLTVNVVDINDNTPFFVGTPYRRSLPEGDYTQGAGE

FIVQVQAQDRDSVDTSITYQIQSVSDGGAGKFTISNTNGQLLLRAVVQVGEEYTIIVNAT

DSTVPRRSASVPVYVTITSSGNKDPRIPAGLYTVYVSEGIDVNRDIFTIPATDPDNQALT

FSIINGNNGNTFAINNNGVVSNRVTLNRETVPEYNLTITASDLGTSPASTVLRVIVTDVN

DNSPVFLNSVNNRYFFNVQEGQTPPATVGSVAAVDGDQSGTPQATVIYDMVGPNTFFRID

GQTGAITTLVPLDYETQQQHEFTVRAIDSGASPRANVATVVVSVIDTQDSIPLFVSVDYE

GSIQENLNGTSVLTVSAVDADQTDNIVYQFAGGEFQSFSINPTTGVISTVIPLDYEVKNR

YLFTVTTSDGVNTNAVSSTATVTISVIDQNDFTPVLTTSTLVDTISVLENLGISEELVDV

NAADNDPPNTPNSRVTFSISNVSPPSGTQLFFINPTTGQLVLRNPFTDDPGVPTYTVTIL

GTNEGSPFRAGVLTLTVNVIRNTAPVFNPTTLPANIDENSINQVVSVITATATDVDTVSP

FNVLTYTLRGDGPALDYFQINSASGEIQTRALLSSTTDRSFTLRVRATDGGGLFADLRID

LTVNKNLNAPTLSQQNYTETIFENRALGSVITTIRANDADRQPPHNELSYSIAGDATAQQ

FFAVNNRGEIYLIRSPAENQAISRFNMIVDVRDQGLPTNRPANPNPAFVTINVIRNANPP

IFFTREYNRVIPENTTIGQFVETVTATDADSQGNFGTLRYSIIGDGTADTYFQINQNSGI

VSVRTSLTNTPINDFTVRVVAQDGGTPPRSATALLYVTVTRNFERPVWSSATYATSIPET

APVL

>**comp107084_c0_seq5_1** len=2361 path=[3718:0-107 3826:108-140 15493:141-183 14611:184-188 14621:189-584 4308:585-590 4314:591-2006 5730:2007-2075 5799:2076-2093 5817:2094-2255 5979:2256-2258 5982:2259-2314 7504:2315-2360]

ECEELQG**SLQFMLPGSCSQTCTAQI*FAETHITSQLRLSTSKLDLVKTLGQGTNRSRC

L*MCRDNNNMKLVVIFVVLFVGLGASTSPGFKARITQSGLNYANKMAMLALSQSIKKLHI

PDQSGKSGKVSYDITGMTISSFTSPRSSMLLQKGAGIKWNAAGANLAMHGNVHYKYKQGW

IHISDHVSFDASLSDISFSETIKLGDAAGKPKLSSSSCSSGIGGVKIHFHGGASWLYNLF

SGLIEKKAKDLLRSQMCKLVTAAINTDANKQLQKLPMTVSLLKKFLLDYSLISNPTFDTK

FMETMLKGEVYWKGGKEECPFSPTPLPTTPNTSRMLYLWVSDYMGNSLAYAGFKHGFLQH

NLTAKDLPPGNRSIFNTTCTYKCIGFFIPQIGKKYPNSQVTMRMHATAPPNITITGGLIN

MNFSGNVDLYAKTPSGKTPFLLTISVVLVAKVTANIERQLVKGKITSTNLQLDVKNSTVG

PVSSTALQLVMNTAINLFVLPQLNAQGQKGLVLPVTDNIHFTNARLTLLKGALLVSTDLS

YSPSTDEDDLHLNKANVPNTTRDRAMTFKVVGEKQPVSNQI*LALYPASLSLIVTYLFRR

CI*TRH*IYFTISK*RKKVKCLIIDFEKDILEINRVIK*SFWVQWEPMVTCCKHQHYLNS

QIYVQIFSLFVTQ**TTIFPVFQHPASVTIDLVTVMYLHTSIFVEPLSLTVTCVLITIL*

VGLQTISINPAFCHFYLCPGVFIAGHRGINIYFWFPIQLYISINNIPFISFKDRIYSRGY

KYINH*L

>**comp107134_c0_seq1_6** len=18397 path=[18394:0-271 41822:272-289 18685:290-733 19129:734-1246 19642:1247-1905 20319:1906-5752 24166:5753-5759 24173:5760-8137 26551:8138-8156 26570:8157-11611 42906:11612-11627 30041:11628-12118 30532:12119-12149 30563:12150-13025 31439:13026-13105 43150:13106-13110 31524:13111-13111 31525:13112-13121 31535:13122-13351 31765:13352-13366 31780:13367-14416 32830:14417-14435 32849:14436-14674 33088:14675-15996 34410:15997-16093 34507:16094-16117 34531:16118-16408 34822:16409-16432 34846:16433-16549 34963:16550-16574 34988:16575-17184 35598:17185-17900 36314:17901-18239 36653:18240-18356 36770:18357-18396]

ASSDNRVDIITLARHQKRNLPTLPEPAD*SSFTASVTPSEAAGMGGFQQLFLLFKKNFIL

RKRQPVTLVLEVLWPLLIIAVVALIRQGVPPTKRDTCHYQKRAMPSAGVVPFLQTFVCNL

DNQCHSPYTLNQAQETTYSVSKLVQQLAPSLSSDDVLQGLMAMDQGVSVMDALVNMANDT

DLLSGLDSFLTVRSFFDDPNYVKEVLVTRYRVMNEEQADALLDSTVNITTIFDIIGYPDF

RGVACNPARLSKFLLFKPNVDVRNISSSICNISADKISEISKFLQSQFDIAELIRIVGKF

EKLKQRLGMPYTSSQAFGDIADMVDLALKSPSLQSMLGSLSSLRQLPDLIRKLPDMIDKI

EKFDYFDMAPFVRLVGILDPVVESLKPNDTIWYTVKYTVLVAADLSDLIQRRTNGSASDT

IDRLLGSMQKMLESVQQLAGAMELDLKLVLGAMAQVDWLNIYDQIVTGAVNTKATEQNLQ

KLQTTLQSQPFIWNVTAPVVMVMNRILDIGTIMFQETKNLESVVSNMVNKIGPTQTALTT

LFNKGPNITLAVLSAFTDSKALARLFESPFSYQDFCDGIMDDVAKTTDAAVVAELRAVMC

TANISASVNNLFMSLRAQDIQQIMEDTVMHIRNLMNGKIPPANITMVFLRAQDMATAVIN

FVDITNATWRNVFSTFHLPNIDLSARQWEPVLDQMNQNYVASAFLAMFRGFGTAMGSGNV

GPMIGQYVHMADAVIKYSYQYLKMYIDMYSPTSTMGRMVEMISNYAPELLQAMSNLAKNP

QQIIQIVSNPDPMAAMCSWVQQMPLPSYVPHTDLSNMVCYELPEVFNQVMGMSSAVEVMV

QQIINATLPQPGLTYDLQKDWSEIMDYTERSVTMLSQPGNLLTIVDGPLSLMMVANFSRM

EEVSTVMGDIMKGFTYKDMASFMDISFAVMHQLEPSMQNSNEWKVVEHSLLGYNAMIKLY

NHYYSSYNATNSYSDLLKMFPAEFQAFASKMAALTPDFIEALKNTIINPEKLVRKIFALQ

AGYAGPDCSTSWLTDFLDIDTNSSLVEFEQLACSFNWTAFGMRLLQQDPNMQEYINQLTI

LSNPDLSSLPDVTVNWTELMVNTEQYLQWISGGLVMNPTQDFNFAPFNVSAIDMKWTEFV

AAMQSLQNIDFQRLGDLVTMIQMSLEKLDMTMGNSTENIAEPLILNSMYGQLYMNHHIMN

FMNKILTYMTTHSTVNFYDYLGSDELKKSSRPTELAPEFTELVLDSLVSYFAEPQKFTKF

LNMPDFWGQLCSNVSVFTSVFDMSKSRADPSTLQATLCSAGSLNYTVMYEQLRQNWDGFA

EFTDALIGISLNTIPVEQLRVNMSTLIADQLTYQSLLNRTIMNPPNIVFFTNNDWMNATI

YAELGETFLQDMNAVLSRFQDPGFVVETQTRMFEMVFASLADIPEAMVAMKYMEVISESV

LLQIRRNSEPLNETLQDFPNMLKISQLMNDLPFVLEVTLYTNLFSPEKTARWSSAMQSLE

TFCGTDPVTLFTVPPALMFSMTPFLRTMCSINITALMEESQRYSGSDTMAALMAGNMTEP

VNVTALRLTMMQIVEALSNVNTTSGMLQSLPPMFDEAIWMAVIQRMGGYLNQSSSVYMSP

KSIFLLAQNIVTSVPGLRSQLKPMEIVMIVVETILDRVLILENATSFAPRDIFPQSPQLQ

AMIDLLQEPGVLVVLMESLNSQKMTPLFAMMNVTEVFMTLCWPGADISQYLLVPPGVTFD

VSALQRGFCAINITELEPEVYAAFDIPRIERVVNGSEQVNWLEVGDKFQRVLDQVTRWVQ

MPPRVVLPPVWENETYWLNMLQQYSMARQDPAAIQAEIENLMTRMGPLLMQEPFRQYGIV

MEAVMRLLNENIMGLQNESLTLTSMFNQVPILRDVITAIGLKGDMLETLMKAPVKDTELF

TRALIDPKSSNVCTSPIVWRDILYLPSTFDFSALTQAICNLNTSTMVANLVRNLDLERVI

AGLNNMSVVPDWKGIMTQSEQLSQNINNLIQNPPSFNVSATLDLLEKEYNQTNLWNMVTV

YSALAQVFGNTSEFQAVEGYMNGASLVLNFLNDLFQKMSVNGMTLDLGSLFSGSPTFTGL

VNAMLHLKPDPITALVSLQLKNSQTMAFSAFVSDPQRLASLFCDEAVFRQYFNVAPGFNL

SSLLPQLCRLNFTNMAQELDSNFMVSALVAKFQQMGSQPFNMTSYMAVYQQLNNKIMELV

GVRNVTFGDYDLERLSQVNMTALLQVSELQSAAAMRDYPAYINSVFDSLQQSLGNTSDWK

TVVMSLKILDLYLKYFNDNLKTISGQPLSLELLVRNTELGRVLLPVMTDSRWLEQVLQLQ

IKADKLQELLQSPDPETTLCSSALWDAFVTPSNSSALQYLQQQMCSINTTVLMWQSITGI

AQGYQFYQQIMLLMAEIERGASTVNTTELTSDLTNTINLISAYANSLINSSLSADNFVNV

AGFQNVLSRFQGTIGQILMQMSSQLSTTMNTLIWPSIPDRAAADSMARSINTMKVIMDVI

NNRLEDIKAGNISLDTLSGKSPALLALMEAYINVTKFGLQAWMDGQIDMQKIIELMSDQS

VVASRCQDGSVAAFISNTSTPSIVARNLQTVLCTYMTELPQELSNLVDYQQIQQQITDIW

NNTGEVTPDFTGYTASTERFSQLVTEIAQANIQVSQGLKNMFDYSSLLNSLNNLVKDPSL

IFSLLKPLGMVLDKPLQHDIAKGVLSGIDSYMLIPVSHYLQILKDNGLTFSSMLTDPMKM

IKAMGVMASFDTQLSLSIETVVKPMFKSISQNTGFISDVLCNTTLVESYMLPLINPGIAT

ILCHDPPSMWVPKLRQLGLENVEIYGLSGKLSSLQIQYSLSSSCGTNCTEDGVCVQFCES

TMWSPLSNDITWVQFLSDLEKMTKLFMSESGGVMTMNTTAIKAGESLEAIWNSIGKSVLG

NMLETSFGVMKMVDISQSSDEAWSKFKQIIHFVGTVNSYLNGMMERFTNSTSEIYLQDVF

PDSKQVARLLTAAVGQNTAAELLTATFNPATFFNQLSNPASWVNILCDPALFSSTFNFSS

GVNVGAIQTAMCTMAVNQTSSLEQLVNLLDAGKVLKELEALMAPQQSNATFGLPLWDRVY

NTTLQLIGNFEKLSNVRVNGTSAMAWFNPILAALQSLQTPNMASGITACNDLVTYLRGTD

MFREVQPIISQTVFNIKMIADQMPLLSVADEFVCTLMRDANLNGAVDILKNSGFWASLTQ

VNPTVNPSGTDDLQCSAMYQTGKNLWDIINSTLLMGNGPDWDQMGQCFVDSSKNFRSFAA

GLNSALSVATDMMTLIQDPSLRQLINTEGSLAPVLDFALRVFMEQRPVLLKFSDLLKNDT

SVQNYLLGILQLSPELVSSLLKSSINLDAAMFLNQPVEQIQEILCNATLLGEVLTLPDFV

TDIPTLSRLLCTNDSLTTATSLKSAAEVTSIIQQALAATSTGLSQQFFNNISNHVLNLVS

DLQFVTEIAGIFSNGFDVDKLRDNIPKIDRFLMSSGPERLVQSLTNILEDIRKVLPGNSE

ANTVLKEIGIFIRGFAGLDIVQSYFLEALTVSDFVKSPTAVYNYLIQMGMSPSSAKTVLE

GTFSINVFLNTSYILDANIPCPEMFNRLLSLNTTSRLSITQITDDLCRVNETVAVNIANM

LIPHLTFGDLLQRYVTFSGDDIFKSANITAEAAADVATKLSKAQQDLIHAAELLNGKSTT

MNYKSSEMLGELFKLPQAQTGANTMDSIQPLLCGKKPGDLAVDEFNVASVLGRGSKATTN

EEELSELAQADKDNGGDFCQQLYNDIQSQSLGSIIWAYLKPIMRGKILFTPDTPLTREII

GQANRVFDVLKDVKRVAKIWSDGTPNLMAMTDRAKKMGSVKEVLKNDFITSLLKQTSGLD

AKELLTGIDALESGNFNSSSMKGMKVAADLLVNYTSCMELDRFVGASSEEEMEKQAFSLS

ATNNFLAGVVFTNLPSDDGGRKKRDTGKLPEHVQYKIRMESENVRITNRLYPRFWEPDSY

DRFTSHLQYLRGFMQLQDMIERAIIAIQTGENATLPGAYLKQFPFPCTVDDSYIGILGSY

LLPVVMTFAWLAALAIATRNLVIDREEGLEDALRIMGMKPILNWFAWLVSTLVLMVIVSA

ILTMILKFSQLFRMSDPTIVFLYLICFCFSSTMLVYFVSAFFTRVTLAILMVLITYFLSY

LPYIVLVSMEVSMTFWQKSIACLLSTSAFGFAAQYLSRLEMQNIGLQWDNIGSSPVVEDP

MTFSWACYMMLIDSAIYLVLGWYVRTVMPGKFGTSQPWYFPVSPSYWCGRKHKGTSGLSS

RVGSQNVTLFESGGGGSGIPGMAVRNLTKKYGKKVTVDNISADFYEGQVTALLGHNGAAK

TTTMKMLIGILEPTSGEVFINQKGSAGTIGFCPQHNTLLDYMTVQEHMELYSGIKCDWSS

TNRAREIKSLLLDVDLYHVRYVRVSQLSGGMKRRLCVALAFVGGSTAIVLDEPTSGVDPH

ARKHIWNLITKQRLERTILLSTHHLDEADTVGDTIAIMHEGHILCCGSPMFLKSKLGGGY

HLTAEKAAKMEATGTFDGNQSDCNSGQVLSFMKTMLPRVQLVEEYGTEMTFSLLREDSVH

TPFDIFFRKLDENCDRLCISSYGVSDTTLEDVFLKVTALADDHVVLDEEVLEKERLKPLR

TRTVTETASDTTSETSSQETTVNMLDSGRVRYSGLSLKLQQMSALLLKRFHHYRRDWRMY

LSIVLLPFLLFLTSMGFATIRPEMEAMPSLLLTPDLYGPDNYMFFKDMSYESLSDRIAET

LTQKPGVGTVCMEGFDSGLPVTCDWTKRNFTPRVSQTNNDGCSCEDFKQICVPGSVPFPK

HIRTPTGNYLQDLLGEDISQYLLHSFDEFIEKRYGGWSYEVGPEGTDSDMAATVWFNNQG

YHAMPAFFNAFSNSLLRAKLGQKAGTDPSKYGISAYNHPITLHTMTLSLDNMGQQATDAG

ISLVFLLAFTFISSAFMVYLVNENLNKEKQLQFISGVGPVLYWITSFVWDMILYSLTVAL

AVVAVAIFKLGPYWHRQNLAGVVSLLMLYGWSVIPLMYMTLKLFKSPSAAYLTLFCLNML

IGILTIITIFVLIIFQTIGTEIGQAFDVCRYLFLIFPQFCMGQGLIDITVNHYKYLLFVR

FGDDVYVDPFSFELLGWNLVAMGIQGLVFFIVTIIIESRGSSGTRIPSRLLEIPEEDGDV

QNERLRIQHGQTKDDLLYVSGLSKMYRRGRKSFLAVDNLSFGVSKGECFGLLGVNGAGKT

TTFRMLTGDTSPSSGDAYLNGHKISTGDPYLGQEIGYCPQEGGLDGFLSGEELLFCHAQL

KGMSQQYAHKVVVDLVGKLHLAEYAQKAINTYSGGTKRKLSLAIAMLGEPPVLFLDEPTT

GMDPATRRLVWKCITKAGQNGQSIVLTSHSMDECDALCSRLAIMVNGRLMCLGSAQHLKS

KFGDGYTVTMHIQGLSNNRFNIEQAFLSRFPGATIKDQHSSVLEVGIPRNSTSVSELFSI

LQTAQDKNHITRYSLSQTSLDTVFVNFAQDQSDSISDKDDVSTSDGETGSNQSGILPGPF

TNQTYAYMNPQYMADPKDERFSSQYAANLAHASTGISMKEGKINKAFQDEDKVFDTRL*S

TTAIPTDD*LKYFVKTCFIFRQNVTVVMYSCIYLPCKRCVQILFFMIDILHDNHRTNIHV

F*LLTTYQSRDQTYPVHQLKVGVVCNKIIENIIHFNISTSKS***TCGVFNVP*NFRYIN

FIEISCRPLSAQ*TNWHNVL*YCLITMQIFVLSCLQPSQRGQLFYSIHIF*KSISVDFKI

*LSICMTT*K*FNLHTFYMIFLIITFSFSGYSRGRKPSIPLYQQLFKYLLHATISFSTMT

KDFVMKCLYFM*KKTPNCVEITFNFLHIF*HYGFCTDEYL*WGW*HILQCYIQFQQHLKG

KIIAQSFIFFKL*TKPCKRCLDNRVQYNVCLVETSVYSSVSHTFLFRNVMSRYVLMLSLK

SVDQ*FTGKN*DSVL*LV*RGLSTHCII*NSAVITFCHTVFLTCCFFITITWHGARELSF

NCSKLFTDIMYHDQNHQLL*CVYIMLIHLFCGHRLICTDYFMTFIFSITQV*IDTIYYFL

YF*QNNVCLNKP

>**comp107184_c2_seq4_5** len=2441 path=[18680:0-7 12584:8-106 3417:107-185 14896:186-208 3519:209-263 15635:264-281 18947:282-449 19083:450-467 19101:468-571 3882:572-572 3883:573-624 3948:625-838 4162:839-1358 4682:1359-1625 4949:1626-1716 5040:1717-1729 5053:1730-2140 5464:2141-2160 5484:2161-2285 5609:2286-2440]

TRKHCLLKKVCFICDLSCHPVLRKRASFDEWIRGYRTPHISYIPLRMYIVPSHIVCLCQA

FQHVSRCTGAS*YKARSSFSARYSSVGHMTLSSCARSSRQRGEFVSATIGSLGHCDTGYL

*MS*WQTV*DIINMEFTLLLLTAAFVSVFSIPLTFHSMKDEIESHRADAERIINLVVNGS

AKGQTYNRLAEFVDTFGSRIAGSQNLENAIDHMMKKLKEDGLENVHGESAMVPHWVRGKE

SATLEQPRILPLNILGLGGSIGTPPEGITAEVIVVKTFDELKRRAAEAHGKIVVYNEDWV

GYGATVQYRSLGAVEAAKVGAVASLIRSVTPFSIDSPHTGWQDYSDNVTKIPTACITIEI

AQMLHRMSLRGEKIVINLKMEAQNLPMVKSRNTVAEIKGSTYPDEVVIVSGHLDSWDVGQ

GAMDDGGGAFISWQALSLVRQLGLRPKRTLRMIMWTGEEEGLWGAQEYFKQHKAEIPKQS

LVMESDMGTFNPRGLQFSGNKAATAIMKEVAQLLSSINRTTLYSPADVSDTTLWENIGVP

SGSIENDNENYFYFHHSNGDMMTVENSSVLDLCSAIWAVAAYVVADLDTLLPRDTAQGRL

QL*SSPISTRHDARTNMLHIISIPVNAPKSR*HNLEINDHINLSWYNTNPITVSKGINRD

KLTVLAQSQMSRMEVMFER*GLTIFASILYVAELWSHVCNNILFS*ICIYIWCAYTWRIR

VVIMVRQLFRI*FWKAQLFDTECKSNFELWCSGR*YVLQCLLSEHAW*QLYVWIVWASIF

NIILPEYMSRII*LNYLVYPQDTKYISVCVTIYX

>**comp107245_c0_seq1_5** len=10256 path=[26608:0-0 12199:1-605 12804:606-1785 28185:1786-1789 28189:1790-1846 14045:1847-1870 14069:1871-2058 14257:2059-2082 14281:2083-2132 14331:2133-2140 14339:2141-3214 15413:3215-3432 15631:3433-3445 15644:3446-5546 17745:5547-6331 22719:6332-6335 18534:6336-6491 18690:6492-6495 18694:6496-7126 19325:7127-7703 29084:7704-7721 19920:7722-7740 19939:7741-8125 20324:8126-8126 20325:8127-8318 20517:8319-9073 21272:9074-9093 21292:9094-9697 21896:9698-9721 21920:9722-10255]

VCRGAVQGVPVAVVLYTMGR*NKRDFHQPVMVCFISAGTLSLQ*RRSQAF*PPNFHPIHS

VITERT*VSGLFRNMRGVHLFAVLALAATATSQAVDDCATVFCGPGRTCVVGPNGNAACV

CPRGYYGTNCGDSVCTKLKEAASNLDLCTLFGATTATCVGDIAGGQLCQCPNTHNGVICE

NLGAAPVSLTDCQRQQRLFSYITYMLNSTDSRNATLNSLRTIFNIQKLKTLLRDSTSQML

PQRECTNEGFNSVQCEVSVDDLNSRKCYCADTSTGGILPGHSKSSMRPTDCSVSVPDPAA

VVMGRFELFMGWRQVFNDTSSAEYRSLVLNITNALTQRFKDIPNLAKVEVTGVMLQASGA

AVIGYKLLFSSAPTAAQQMMFLNFTNTVKTTGLIVGGVRYNGTDSARHCMNNSMCGNGYC

SNNRCVCNQGYWGPRCQNSSHSTNSSCSITCYNGGSCLQSNGLSFCMCSNGYTGYQCQSR

MPHNKTGMCPPMNSTMMGICVEACTSDANCTGVEKCCSNGCGHTCQQPVYYHNSTGYCTY

NTECNNGNCVNNRCRCHQGYYGYRCQHSSHSTNSSCSRHCYNGGSCMRSGDMIYCYCNNG

YTGPQCESRMSSGNDTVKACVPLVEYTGLVDVVTNCLTFTMTLNATNFNSSDVCRLYAAS

VACARKEFRKNHYNCSFMELSAQINSYFSLMRSVSNMSVPDYSQCQAPMGNFSMPKPLCE

DPRLLAMYSAQSICFPPGSPLDPCMLLNGSVSCVQLLVKCPASRIYDAVINSSMVIQQRT

GYNFSKCHRPNEGSNACSSLLTKSQVLVKVIQTCFQPFMMMNSSVSDADKCSFYKSGLSC

ARQVAHIEGVRCSLEELHGMLIPYLTMSGSTFTLSNCSVSASDNYTVKPLCNDSYLLAVY

AVSSTACMPSQLMIDQCSMMPSISSCVQMYLNCTQSTINYALINNTAVVRRRLSYDFSSC

SGQPAVIYKYNDTVRIDMPWRNDLYNPASSYYITVKSAMERTLKPAYSYLPNFKNVTVLG

FWPGSVGVNFTVDFDAPISNITGYLVSAMKTIESTSISIDGNTYPFVKSEKPGVCPPVSG

NSTGICGPVCSSDGDCSGDSKCCNNGCGRTCMNPYNRPGNTTCFTNDECYNGGTCFRFNC

ECANGYSGVFCQNYTGTQGDCRSSNWTCYNGGSCVHGFDGQYFCACQNYYRGRSCEILIQ

NTTDVCDSSPCMNNGTCVDYGYSSYGCICPPGTMGINCEERLCDYFDSNICMGGSCIGDM

RTGEICQCGSDRKGLFCEKQGYEFIWGCELKERFSAYIQKVLQGTAVPPPINYARANNLI

EILQKFNLTSYIKPKCSPSNPTQYLSPTCEVDVLTGRTLCFCVDNYGYIRGNKTSQPLTA

ADCGVTNNPCQSNPCQNNFSCINLGNGSHMCDCPSSYGPTCEDVPIYMCQCRTGKKCIRK

ERCGTSGCKETNMCVDYYYRSPCDAMPCSYNGQCVERAVQGFYCNCMEGYTGVHCQTNTR

VCSMFGSVNICGAGECVGDVQNGMLCKCPEGRRGAFCERLGTARTQCERQQDLTDTVQRI

LNGTETVPGVNNASTMVGSIMRSALGSSYLPLTNCSVNGDFAPQQCQRDIYTRAERCYCV

NSRGQEISTNVTMVPGQPPCTAPDPIDISKLICSALSNICRNGGSCVGEMDRTPRLCLCP

DGYEGILCERRSEPGDKPQNYTVCTYSHESYNMMMAILNDNFTLTVANITINKNTLMSAF

YQGAISSFLTDSTNSTLNRPVMPISINGTTRLAVIRPNCTLSGEFAPMQCEYFLDSGERH

ACYCYNRAGRVVPGTHTMAPDYPRCDGTHPECENTTCSLDCPHGLRKDMRGCFVCECRKP

CDGVMCGRGLRCVGSDYGAECRRTMKHGSCPMDTIPTMESLQQMFSDNMQSCDATCHDDA

DCEDNKKCCGGCGNKCVTPYMPENCQKKKEVLMAHWNLYQNLSNFLQDGNMTSMANQSYP

AMMHSAMTWVKNTTHMWRPSCNASGDYERVQCEYNLRTGQKDHCFCSNREGQRINGTDTR

PPSMPMCTTKPGYCPTGVPMTTECGVKCTGDYDCGGHDKCCVSGCSARCLRPVNDTTSPH

QVANVSALLRSVCVRFMDLNFCQSGSQCVGNWEQGDLCACPDGYKGPFCDRMIPPGESAP

SVCQKRHSLFGMIMDPEYAHLKMMFMTYANMSSLAVMKANCSGKEFNPVQCDHDISNGSR

KWCYCVNQSGRPIPGSGVPSPRWPRCGDMHCDAGHRLYNSTSPGNARKCDPRSKPCPPGY

GCKQVAYGQHVCCIDQYGHSDICRLTPNPGQWCDNKENRWSNYYYFNSTLRKCMLFLYRG

CHGNQNMFETEAACNQRCARKEKPGMCPRMMEVNLNQRYCKDYCMDDRNCTEDRKCCQTH

CGRRCSLPIVDDNKKKCRVGVEYHQPNGTVVYCGRGQPRCPDGYECNVDPLDGPSFCCPK

ETEPLNICHEPKENGPCKAYMPRYFFNRTSEQCERFIYGGCRGNNNNFNSLQECCSQCGT

NTSRCKQGKCPKPQVNYFAPCQDECSSDAECMGNTLCCSNGCGRACLVPEMKETCHTKMS

EAYMKLKAHKGGCPMISIPRCSIDGTWRPQQCLDSYGVCWCVDPHGHKIPGTFVRGYANC

TSMGRQNISQPNTSQPNISRPEGEMDLPAICEDGTKAKCCDARLCHMNSCPTHPAAVCRI

NPCGGCRAVFYDEMNREVNCNEGLTKCQMKRHQVKTEITQHKMNMMASMTDMLGNMTMNM

TFGDDMMPGPVEEPDSIPAHCQLAPETGMCRAYMPKWFFNHTTNRCEKFVYGGCGGNLNK

FDTVEECYKECNEDANPCKMKKCNEQEKCSLAIDHSCQTGKCAHWPECSYNVSSPHITGV

HVPTCTAQKFTTMQCRHGYCWCVSPEGNAINGSLAKTYGVMCQDDGNYNIFEGKAVTCSN

GTQPNLRCLDTCMGKVCPGKPKSTCVVDLCSNTCSYKFMMGPEEVECGEDDCAVMYAEPK

PSCQVEPDCPRRICPNVCETQTCGSDPCAMCKVDPCTCQAVFTDMMTNKTIADCASVSHS

HCQMTWCTKKMEKHREMLMTGTTTISLPTCGTDGRYEPKQCEDAQCWCVDGVGQWLKRSD

LYDVCGRNETVKVVIEMKFNNDFSLVANKIEAFKSALVMSIIEASSIGDAASYIKEIQVY

EGSIIGKVTLEDKEDMATPSTELPVIADRIEKAVKSGTMQVKFEDMVLVAESGLYSRTDR

FEGDPPIDVTTTTIMPTYEPEPEAEPLEEKYIIIISVVAGVVGLIVLSALVYCCCCQRKN

KSETDSSSGSSDYSIEKKQDFQSPAVTNATYGMVYPGTDNRSYKQDDDFIKIRL*VSYLS

R*DHPIKLAGEQSGPI*PSC*ATKLQTVFL*ITEEKF*NVRSHLKG*RCLLYSLHVFFX

>**comp107325_c0_seq2_5** len=23389 path=[1:0-1580 1582:1581-5577 5579:5578-5617 5619:5618-5929 5931:5930-6112 52603:6113-6270 52633:6271-7110 52723:7111-7121 7123:7122-8290 8292:8291-8665 8667:8666-8898 8900:8899-9708 9710:9709-9952 52965:9953-9996 9998:9997-10275 10277:10276-10312 10314:10313-10317 10319:10318-10351 53062:10352-10415 10417:10416-10440 10442:10441-10507 10509:10508-10531 10533:10532-10713 53124:10714-10833 10835:10834-10904 10906:10905-11098 11100:11099-11211 53208:11212-12256 12261:12257-12577 53234:12578-12619 12624:12620-12784 12789:12785-12786 12791:12787-13115 54050:13116-13468 13473:13469-13572 53429:13573-13578 13583:13579-13580 13585:13581-15988 15993:15989-16965 16970:16966-16969 16974:16970-17798 53586:17799-18424 18429:18425-18430 18435:18431-18479 54059:18480-18481 54061:18482-19257 54070:19258-19912 54078:19913-20364 20369:20365-21083 21088:21084-21103 21108:21104-21282 21287:21283-21297 21302:21298-21867 21872:21868-21892 21897:21893-22809 54019:22810-23046 23051:23047-23388]

LAGATDTRFMHLTPAMAMYMRYLVL*SVKMSGLRRTTMNEILAGFVTFLVCVNARFINFV

PGFEYQYKFNSKADVKSIGEFHISAKIGYTNIREDDERQEVSLRVYTLSLNTKKSQDVVG

HDWDFSRWFSFVITPHGEIVHVYHPAHDDDEAVAIKKGLAALFAGRLHHESEKVSRRTEV

GWRYSVQETGQEGVHQASYNVRPSKEGHVFTKTRHPKGHPVKHASSEYTKTLYYSKDLGN

IHSVEINETFKVMHKVQDGYEPFENSRPVKAVNEFTNLEYPELSALGTGKLEFLTRYKLR

HVPSRPTDATTRSSISIDKVSRKKPNTVVVKDEMKYITSNLTCMRNEPQKGSKRLTQCFM

GVLACLKVLPDDYIDMLAKKYFTPKPRHPRDVKDRNHMFDAIAAMNTVHSQRLLLDTVLN

VTRPNAFLVKRLLMHIVTMETPPIDDIISKLEELCFRPDHSPSELQDAETHHRIVLAVGV

VAKRLWAVGRKQEASQIVGKIESWLGLHDPWLYRHKRALMTEKQMLTYDHWRVVLLESLG

NAGLDQSYDYIVSHVNDTNSQWVKRAGIHAMRKYDHDAAVHLMMKTALYDEDDKVRFEAL

LQYQAHPRAASVAARYIKQGTVNGSIFYQNPSLGSQDIQVLDRHKRGILDETIEFLLQAP

GVNWKKLLGSLKIGASFGVNLENLLDFKIAPLDGHAKLTVYDEAYAMVHLGVLGQNVDFF

RARLCFKGSASYNLNLLQEFDIDSIKKLVELYDKIKGDVVDAIEVGVDMFKDIIRGDPSI

GDMMDEFKQALEEMPEKVVEVGKKSALAMAAMGEIDEDQLPPFIRPTRNLIVKVTNLFKD

IKGAVMTFYNTLMETITVVIPRAGEQIFKSIKTIVDGFKNFNNDPKQAISGIAGNVITIG

MEVKNLVEAINKTKQACFFLKKEKPYWWDLRGQISEIRAMASNATSALRTGGSAWVNDVV

KGKKDPIAEFTKGKTTMAEQKQEVIDIVKGIVDDLLAPLDGLKNLGGRFVETYEKVFKVV

QDIKEAFDALREGYRTARSLIDRVFGPKCHKAFPRTLRQTGGGCDGHGSYPSRLQNGKPE

YENDGVDITIMKGKSVVAPFPGIIMLSNKPNEVVIKATGGSLKNTDIIITNVNPNSTIQH

PNDGLYVDNQVVAGQVIGVATESGCGSNNHIHFAMRRPGGPVEPTRFLEPRIPEIPKWIQ

ECDDYKLVWKFETVAAGSVIGLLGKDENDTSPERKGENIDNPPNVDPDKDPSKILSAANN

QPDSMYQKQKTKKTEIDNKASKTNDTALKALFKKPAAFMKKFSVRNLKLGALLDIMDILG

LDDSQRKMADVITLIKEMIDNKPCFNPNQLTDDQLRTELTERGQRADGTREQMISRITTP

QNKCPAMKIGMPKNIYCTFDSMCLGLECCAHFKLFMFRKAYKVYARLDPCDFQFIVGVEK

KFEKKFGALDGLKDIVAGFDKKLKTGIKLNILGGIELVVRVRLEKDEFVSLITLGAGFCA

QDDAENCIAFFNILDEAPTPLPICLDDGTMRWPKIDYKALFDKEAIKKRIREKGSQLVKD

TINKGIEEVLALVPCVSKTAPGKSAPCHRPETFTQDLLKQTLQERGLAISGTKSQLEQRL

RDADKMCTVLGKTLTLPAIRSSKLDKIIYMSISSTCLRVDVCVDAEIKAIGFNKAFRAYI

ELDPCRFTLLLNFETCTHEVILFGYAWGTPSELKLSDEVIIKYTIDRDETRKVFIVTMGL

KLGIGSEPILDSTFLENFDVPIPLCNENFSLPGTGSIKDLAKKMGGKLIGEVVDVIFKKL

KLDTVFTNGPCSLGSSPADCPWQLPNITNYLPSSFQDKVTCGLPENCFGVQCCVDFVFNI

PLFDQPLLKSIPFFLKFEPCNFTVEVGFGSYYHKETLLEYNWGSVTALEIGEGDPSPIII

SFSVSKYSQGFIIDLSVTTCIPIDDDSFCFPDGGLQLMKGEKIPACDAKALVEFSKQNFS

LAEWTKELGLDAAQALSQSAARLLLDKFGIAEYLKEERCDVQRDPYTPSVDGWRNECPKS

IRKLPKLPQGLHCHLSASCTKIDCCFDVPFLQMSFNAILNVDMCDYFIFAAVEKKNFTFN

MLGDNVNLNTGVSTGVTIADVFIIDFGLKKQDKMFMLDLKVQVCFENDNCMLDLPVMTGT

EVPQLICDLDAKVNLRNFSLSDWAKAKGQDLGQGLAKAAVNLLLEQLGIKDKMLDPPCDR

SSLKYQPADADNWKNDCPLVNSAFSLPKLTIPANCYINDKCLGIDCCMHSNLLDLSLHTA

FTIDLCNFYIEGSIEKFSFRFNILDYKWGTETEVTIDGVPVLKMNFKIERILSQQMFIVD

LTVSLCLQGGDCELDLKIFHQSQLPMPGCAPIQGFKIPDFSLNNWLKERGGEITAALSAV

LLEQLGIDKFLLDDQCETYVTPYKGAIQRWKNDCPENMTLPALPSNVVCYISDFCTGISC

CAKVPQIRRNFNAHLFIDSCNFYMSVGIEKLEFSHTLFEYDWGKTEEIDIGGVFKAEFSI

QNLPGEKKFLVNLQLKVCLTAGAECQFKLPIFVDMLVPKPFCDWEATKQLKDFSLTEFVS

TQGAQLSDRLSDLLVDKLMDTLGLASYLQDTPCSLATRGPTGWNSACPLDITFPTLPNSL

SCSVPEHCTAIDCCLDVPLISKNINFKVDLDMCRMSLTLGIEQLEFRKMLFKYSWGTPDS

FNLKGVFRADFTIDNLEGEKQFLVSLNISACFEADKACMVTVPVVTQARFPNPLCDWNST

KALKGFSLGNWLSQQNIGPGSLTSLLRSQLAEALGISQYLLEPMCNRQVGIYKPDSNGWN

TACPRNLALPSLPDSLSCHVPDYCTGLDCCVTAASLGMSFNAKILLNTCDYVLTVGIENL

VFKTALFNYKWGEWEQFNLNGVVRIEFKIDDLKGERKFLVSLNVSLCFEAESTCLLSVPI

ITEAKFPKILCNWEDKLSLKGFSLKTWLAERGSAVGSQVTGVLLSKLLDELQITEYLRDP

QCRADAAPYAGAVGRWKKDCPASLTLPSLPSGVTCHLSDPCGTVECCMFIGFIGRSFNVK

IGIDPCTYTFTMGIEKLVFTRTLFTYRWGQVEHFTLKGVIHADFMIDELFGEKKFLINLN

VSVCFEEDSCLLSFPVLKDVKIPKLLCDWDSTVALKDFSLENFLKDLKATGASKLSSAIS

SQLLEQLGVGAYLRSPQCGSDSSMYVPAVKGWKNECPLQGLDSYLPALPNSVVCHVSDIC

TAINCCIDIDFLSRSFNIFLTVDMCTYQLKVGVENLVYKRMLLDYQWGTEEHFYVKGVIR

VEYTINRLMSEKKLEVTASVSVCLKQNECLFSKTFLDKAKIPQPLCDWEARLQQRNFSLT

NWASGKGLSSTVSTLTETLISQLVDDLGVSQYLVSSACDQSASPYSPAGINNWNNECSSR

AVPSLPDSVRCHLTSNCSTVDCCVDVRLIKRTIQAKFDVDICNLQMTVIIERLSYTVSLL

EYVWGTEGHFILGDMARLVYKLEHIPSTQTLVIDLNIKMCFEDNTCVLEVPVFSQSELAY

SPCNPSLPVPFDGVSFDFWKSKKCALQTSACPASLPTAISSTCRLTDNCMGITCCVDVDL

KYLGIYSITAGIEVDHCNDQLVYHIDNKEWTKKLQVVDYDKNHTVLVGNAITVSYVISKT

ASSYEVSFHVKLCVMNVHDLINKCYYYKLLDEKVFQMPSSCSPVGRRRKRSPLDLIHTGD

AKRILNDAMNQNFTNEEIKDLFERLKTQAKRDSSLMFTRTDEYGTATNTKSAIRKMGIAN

PGTILYSGEVGGGNVVLGMEGGEKIMKVLGQVTDILGRGDQAYTVGKGLTGKGLELLGAK

LANMSIGEIIAMFDAKNIDPELALRLTKQLRDLALALYSDIINAIINGDGTNAFSSFDIT

LQGDFSIPRKSIPFFRFKYYFLIGGIVPMTFEFGAGASYGMGIVVGAKILGMTVFGEVVP

YGSAHVYGELGIGLILYGKLRLDGYLMNVAFPSRAEIGFYKFPLDLSLKMDIELIPLEMT

LRGLVTLEVNLWLVTIKKILYQAVIWRFATPVIRKRLIDTGKKEEDKSPPQFLSYVDNTG

GSGRKKRAVSTSRSCLVRQLPNRDYTEPAVEIAIAAQDDRSQVQIFVDAGTKPGLSDVLR

KSTLGGPSTIITQRFSKNGYGVPVYFTVYGENSAGARSTVTCAIPTYDVTPPGGRLTADF

SSTSNPAELRGNVVVYEDSDLVKSSVGVGLGRGIYADEIIAYNSINLRGRHTAGYDPSSD

QYGHEALKHFTGLKKGRLIGPVFAEFSRMNHAGSCVKECMKFPETKCLSVNYDYGPSGHC

ELLEGIEGHDHKIFISDQYSHYERLGVGLAHEFIYKDLSLRHGVMYYFNLHLINNLQFDS

ILHSKGVVVDLTPPEPGPLANVSLDVLEVTSCESVVPDDRPDWEVRCRGVNSQLKNHRII

HDGTGSKTVFNGDEPLTDLLYTRANRYVSANWDGIMDKETGILGYSLTAGTQICEELIHP

HHDPHRHLFDESEWTHTGLISPIPAPYDPLADGKYYITLRALNKVEYGGPLVTTICHTTP

LGVDNSPPLLYEIYNVSYNEDTFFINAQYNASDPHSDIREADLCLGRTTRDCHHMDWQRS

SHNDGDITRQFQIPGGTPVWIKVRVINNVDLMKVGVSDHPIIVDTSPPEPGIVYDGPFFR

HDLNFTKDADKICANWFGFYDPESGISHYEVSVLDDTNTTISEPVSVDHKTHETCVQLSS

NQRLEHGKAYRFYTTAFNAGHKQLNVSAMSDGVIVDLTAPVPGDVVDGIRDSFVDVEFST

HVATVGTQWRNHSDPESDIRDYAVQILRAIGLSSEFEVLRNWNTLGKDVRQIEWHNFDLN

HQDIVKTKLKTINNALGTVEQTTDGFVVDLTPPRMVFLGDGREQNKDTDFQTSSTTVEAN

FQFQDPESGLDHYKYQVYELYRGSKHQIYPDSEGWETSSDPSITSLSQSGLSLRPGAQYS

VRVGAVNRAGAVATYDTNGVRVDNTPPRMKWVYVGIFSGSEEELIDGHVIQSDPSGIKAT

WFAMDHDSGIKSYMVAVGTTKGGTNILNWKDFGSDRDKYMDGLTLNVTDPDTMTPVYYVS

VKASNGADLTGDVITSNPIRVVDQDKAGIVIDGADSTERTNFMDIGNDTDYQKDTGVVTV

QFAGFESHEHGVTYYDWAVGTTPGGEEVQPFIMAGLMHEEAETMVPGNGIASMGFGQTVL

PLSPGTTYYTTVRGITNGGNILESTSDGFTVDITPPDIHMESYGSESNQSRLTSTTTLYQ

AEVDSISSSWKVADSESPVKKMYYSVGTYPHGEDVQPRTEVDILLTGEGALPTGIQPTTD

GKPNILTLTAENDLGLSSSMISPYLVVDISSPTKGTLTCPSFIQPRSAVECTWTGFYDAE

SQVVEFEFAVGSQEGLEDVITPVKLPGHVAKYVVSGLADKVTHGQRYYAKVKAINQVGMT

SSSISSAINVDTTPPAPGTVVELKSAYIINVTDEIITEKLNTYKCDTEEDCLAIDAVCQE

SLSAVNVVWQTFNDEETEIVKYEIAVGTSPGGGQLRGFFETDDVTKRYLSATGLILKGSR

QIFVTVKATNGAGLSTISTSNGIYMSYLSQSLEPLTHVGIWDGESHDGDLDFQTSLSRMG

AKWDVSGDPCPVVKYEWAIQRADGLRVQEYFDTEGRTHGVNDQLAMQNIERYYQYLRVTN

AMDFTYTIRSNGITIEDDPLVPGQVNDGDVVGFDLEFLRTRSKVSANWDKFGSDGNADEV

SAGIKADNVPEEEKEKSSSQEVAFYEVALGTDRRFPKTRDNVVPFVNVGLNKTVTFYDLD

LTPVTALYYFTVRAHSLSGSKTDVTSNGFSVGFDGGVSVGIIDMKEFVNTDTYVDVPWDG

FESKIGMMMYYVGISSSTDANNYTCGQFTERGSISDDERRGIFNVVDLQNVGKDTFMKFE

NLSLDHNGIYYAWVIGADKAGECNMTYHRFRVDITPPTQGKIRTGPYYDLVASYAASSRS

LQAHWKDYRDEESGLRCYHVSLVKRATCQDGAVEEVVVPSIEIEAEYNSYKFIDINMERD

TPYIVRLMTENNAGLRSITDSPPALYDNSAPTPGRIVDGVDFTKDISWIGTSSEVKGTFL

HHPVPDTSACPVRPIKFNDAGWKFFESNRNFDSHNTSLSLTYSAAYVQRTTSGDSMDIKL

TRDTKKDAMMSGTYFRDADLVNGGEYEFSIKSAKGHGKVVTSVLFWDGPEDYIIDYDYTP

VPPWEDSNCACCFVDPIPTSCKCNCTVYKKVKEINRSLSKRSVVGDDPAVEYEVVKLTKK

EKEALRKTDSITDGAEVAQIKREPRSSCGLQIFAGGDKPGRLVAWCSYADLLNIPMVDVR

DLNIDPSASYHAYSINFFIQRPEARDSELTWCMTVHMDGELISEQCGIPHLSPQTKLYMG

VWNHNNYIPETGRDAEGKIKVWSTEASFRDLVMPPEKDKLCRYGDPFKGGNNAIIRYEAA

IGSATGLSDVVDFQPVHTPCIPCLKPCDVYTCDNVCDSSTTSQVIISLTNLTLTESSLID

NEFKPVINYLTVKAVLGSGASAIASSDGFYVDTTPPVFEEDVLLYFDVSQGEFTPVRYQG

SNDTIKAVWKCSDNTSEVVGYQWSIGTTPGGTDIMEATSSGENPGAIKSGLTLEHNTTYY

ISINCTNGGGLKTNYIDTKGVTVLLEPPPAEDVNMTIEGAESLGDTVVPPNSLKTQDQNS

VSASWTVSPDESVRRYDFCVGSSEASIEDIFPCTWVGYNMSGTVTIKDGFLKIDDINIRK

LSEYRPDYNDTMNYTDASAFTMPPGTEMFIFMKLCNEAELCTRKLLGSSVVETDKSTLVT

STNGSSVTASIGGSGNTRKKRATGDITVQTPDGLTPGQSIILTQLTKQDLEKEYKSDAST

EFVPYITNPATSMPDPAFLDRILRRRVNYSDTDISFSVTSVGGLAMPGPLTVTFPFNPDN

QDDVAMLLHWDPAKQKWFQSNATCRFESNTEVRSSTTSQITVKVCNTRATESTSGTPSDT

FFSHETLFVVTNVRASIPNDPPQLTSTTTVSMEEDAGTLIYQLSGVDPDGDIVKFRIDPS

SDVASTRDLTLTPNGLLTFTPALNYYGNFTVPVVLYEVDVVDIPAASTLVIIAIQVTADN

DAPSVFAFSGGVSLILADPTAPIMTLIEQNLVNDSSITYEWMFGAYDVDTAENLTMYFTQ

PGNGTLTYDDESAKVPNCSAETTGILCSNLSLPHSPTAVSWIYRTFQYIPDTGYTGYDEV

RMYTQDKAGVYSDVITVRLAVMARPCQNDGTCSSRNESLYTCNDQRRAESFDRYYTCACA

PGWTGSRCEQDVDECLSSPCSWPFTCYNDVNRYYCACPENKPNCDGFESWMIGLIVLAVI

LFVIISVLAWYIFMVKRGRLKWSTCFMKLRCRAGSQTSSEGKDTATAFVNQAYQDSDGDE

LSLDEPFRRHSQDSGASWVIYGGRPDPTCGTITPDQRRIRSVPSEMKGSRKQQIQNPYED

SWNRPLSGEITLNEEISQVAEVQSPTPRTPVGSKSIISPVFEPEPDYFTPSGNM**YLCC

FILVLNISIIFKFSNKTSMNDCNNYSKI*VTRHHCLLRLGSNILIY*NITLTNSVK*DIP

ETGVLVWISYKVHELLEGAVVMLAVYFLLYFCKTEICIFVLRYHATFIILNSCPSLKSKI

W**VFTHFMNFTLFGQSQNKNYQTPFL*LVIAIFKDGLCE*ISMKKIYQLFCVDLLDFSI

>**comp107333_c0_seq2_6** len=3625 path=[4383:0-2 17147:3-26 4410:27-42 4426:43-66 4450:67-124 4508:125-137 4521:138-161 4545:162-251 4635:252-275 4659:276-299 4683:300-416 4800:417-417 4801:418-425 4809:426-440 4824:441-457 4841:458-464 4848:465-545 4929:546-554 4938:555-569 4953:570-578 4962:579-747 5131:748-771 5155:772-785 5169:786-792 5176:793-844 5228:845-845 5229:846-851 5235:852-884 5268:885-892 5276:893-926 5310:927-1177 5561:1178-1201 5585:1202-1216 5600:1217-1246 5630:1247-1252 5636:1253-1367 5751:1368-1398 5782:1399-1560 5944:1561-1799 6183:1800-1823 6207:1824-2085 6469:2086-2153 6537:2154-2156 6540:2157-2179 6563:2180-2186 6570:2187-2231 6615:2232-2642 7026:2643-2666 7050:2667-2780 7164:2781-2996 7380:2997-2997 7381:2998-3021 7405:3022-3035 7419:3036-3103 7487:3104-3127 7511:3128-3203 7587:3204-3356 7740:3357-3380 7764:3381-3624]

SSPIDIRPA*TKVIVPVNTTLPYMGHRRQIRYFF*SQMIFG*I*IGRYLQSKTEHRQKGS

MMSVQYCIVFWICSLATWNFSTGLEANGVNVEAYTTYRSSRYTTACGFWYRGRCTRYRQR

TGTNYRCKTGYRTTDNRGCPHPVCDNEINPVACNFDYSTSHIVYSNGRTQYKSGGSCTSP

GICANCNEGFYPSRERCRMCSAISNCDLETCTSSSNQVCSRCEGVVLDQAGHRAYVASSD

RRSCTQACSWRSDSTRCYPGTCRNEYASNCACSSGFTGKHCQTITTKPTIHFNLLRLTAS

NGDTTEAPPNINSGPSQSTSWSNINSPSRMYYKFTAEYRMVPPSRHAFIENFRVGIVSGA

ATFKLKRGAGFVSTKVYNCGGVSRSSPDTDLYTCEGNPPGTSVLPLPFHHRDVMEFTYST

SNGGYVKVWNEESNTLATFYYNGATQTHTFTVSIDLVDPYHCTGTTACVGSMLTVPDVIK

TPTVNLRWSGWSDAYAGVDHFVRNVYELHAVGDVLRVKRRVKTTTLTSSTTSNIYTLTTA

GVYSVVLSVYDKGGNHRSTRRILIYDGTSTVTTQAKTSLSVTTASSATSEWQSTSPSVTV

DWTNRYINTVHHNNKWLLGVASVGDISSDYDDKEGDRSVAAINNVQGVTRFLTSYKVDHQ

GGSSIISPPHDNLFTSQGLNQSQTITPSLVDGDTVRFWIRAYDIRPEYLEEHVTVQIDTS

PPVLENLWLTRGDRLNISVHGAEEFSEMTMEWIAYDEHSGLETVSWRIVDKYKDLDIVHG

VQHITAQGNTSSIADCKSANTDAPRGANCYCTPAKGCYHRHFQVKPIVVDSSLPHGGIFS

NKSRGSHDSDYYLEVNVTNHANLKTKMEFKVTIDTSPPHPGVVQDGQLGNPEVDYQQHLQ

LYGHWDGFFDKESGVKFYQYKFGPHCLSANEFGVNKSSFEVTETYSTSASWTAPSVGKYH

VTVVAYNRALEASDPVCSDGVTVDTTPPSVSEVAVRDSRVMEGLVKSSDNTVWFIDAHRR

RTLVVNPDGICSSKATPVDDKRLSLFPIHRYNNGSGMQLGINPECESLLALPVSFTDSLY

VYREHHLFVNWTGSDSESGIYDYELGLMSDPSGEAAPDILPYTSTHHHPQYQGYHPRLSE

GQQFYIAIKAINKAGISTVRVVGPVTVYTRYPAFTGSVTVTLRHNYLIARWPETAFTDTG

YLKYEAAV

>**comp108743_c0_seq1_4** len=1812 path=[1790:0-1811]

SASAILESAAQALPFSNRI*LLKNRRSLHISTLLVEIARMTDQAKEFDELVYKARLAEQA

ERYDDMAEAMRQVTERDQKALTPDQRNLLSVAFKNVVGARRSSWRVVCMIENKPETADRK

KEIAKEFRQSIEKELNKTCNVVLELLDKHLIEKAEDDPAKVFYLKMKGDYYRYIAEFSEG

GSKAEVQQKAQQAYEEAYEIAKKKMEKTHPNRLGLALNFSVFYYEILNSPDKACALAKEA

FDDAIVEIDQLGCEDYKDSSLIMQLLRDNLTLWTSDAACEDEQGQTQE*TLKRNCAKIG*

M*SWNSLNSHSCSLVCPMDSKAWTS*ICCLHTLQNDSIMYELQSCARKEFSYFACFLLRK

LGHPYY*VLTLHIMFFLVQSYNMISVRIVC*HFNNTGHFSGIQVVTVLSEFLSSLL*LQK

ER*ANNFLSA**YIDKQHVLNCYVILINIKAL*YIHPNNLCLCFSIE*IRT*QNILSAST

GSIYSIGVTPLV*CMC*VTATFE*SE*SRYDSMSIY*E*LKYL**IMTVFRLVSVILLLI

LAVA*ICTIFHLQIDFIYDMYWHLN*SDFHVHF*AFENFNLDFM*IDTFYKSCHLPLSWQ

QGLL

>**comp109446_c0_seq1_5** len=447 path=[425:0-446]

*LKADNFRMRNMLVFVLVIVVLCVAQATYPPRGRYYYPRPYGFGHPGYYPQQYHNYYPNP

YQAPQRFPPYPRDVKVIVNADQHMVNNNRPGRFGQGEQGSSGINAADIYILSHPKKKSKT

IVSKN**RGMM*QHLGCVSTP*HNKSL*X

>**comp146963_c0_seq1_4** len=1497 path=[1:0-1496]

DKCMSCTCQIYGIWKCVKEDCPGICEVKGLKTVKQFDQFSFKVSNKGSAECSFTLLELKG

KMKVEASTAPDMSLMTVTVTRGSDSYAIGVKDKTFKVNGQTKVLPFTKTGLFISTAADTY

VVEFMDLTVSIDKSSGIVISARPSVYSTRASGLCGNFDYNPDNDLIDPATLGQFSEDDFI

AGNSQERCLAFDAYDSTTKIPQCENMRKAITSKCPSMEISKLDEYEVACEAMKGEGVCGL

LKGMLRDCGICLTQFNDTATVDCSSMTTCATGTGNQDGDKEYSCCVKSCDTSCRDLSLSG

GKCTGDCLQGCTCKPGNYLDQDNNCVPQDQCNCYSLDDPTTPIRPGDKVTIGCQECLCSG

GKLSCTNTTCEKMTCLGTQTLAELPDGTCKPVCQMPGRNSACKQLPQRNGEFILQEACYC

PNGTVEDPSGKCIQAGQCPCYDTGDYFAHGSIISAGCHQKKCNNAHWDVLDSSTCRQICT

LSGTQQFFQTFDGDAYYFP

>**comp172842_c0_seq1_2** len=250 path=[228:0-249]

DKLALPKLARVTKVLGRTGSQGQCTQVRVEFIDDSNRSIIRNVKGPVREGDILTLLESER

EARRLR*ILLAATFLYLCKMKIN

>**comp186485_c0_seq1_2** len=291 path=[1:0-290]

PRAQVATMLKLLVVVALLGATFAKDSYVILSPTSVRPGMDFEVSANILKATGSVNVKATL

VKGTQSIASASGVLAQGQPQSITIKVPKDLKSGSYKL

>**comp191803_c0_seq1_2** len=480 path=[1:0-479]

AVTTSFNEITLTGKPLVDNVVSSDENIFLPLETTTSTDQEKTFTLDPGTDEVILITKARP

TGITVNNNPVDVTKSVEGSRKGLPVFITSADVVKGSTEVTIGIAGVELLDVFEKATGEST

GPSTFEFLINRPLESIILSQSATLSRLDICEHETTGTTTX

>**comp204975_c0_seq1_5** len=466 path=[444:0-465]

RGTA*PHNTTADRRKKAHKTRRKARSRLLASSRP*PLSLELGSSSFVVTLTQGEFSKYV*

TPGCSMSIRL*MG**LRMKIRTKYTQSRTKIQSKRTLGTRYGRHRPRLK*RRRNKSRIH*

RLPQTPNLTLSFVWQCI*SL*N*GDI*WCKTDLWRX

>**comp22563_c0_seq1_3** len=1128 path=[1106:0-1127]

TTPAEGTCVSTCLGIPVSSERYPHCTNCSLYVQCLSGGMVVTACPSSLHHNDANDECDLP

SNSGCTYTSPTTSATTSATTTSTTTSATTTSHTTNPPNVCLSSCNNVAVSNERYPYCGSC

THYVQCLNSGMNVVKCPANLWHNDAIDDCDVPSTTTCYATTSQTTSQTTTATTSATTPGS

TTGTCVNSCTGKDDGDYQHCTDCYKFVACSSGIKYDMPCAATLVWDNVEKQCLGTSNTCT

PISG*IVVLTRQCS*TSDWFQKTERNIHLLSL*TL*TAA*KK*PPK*SFKSFPVTD*NFS

SPQPNFSHPPKIRI*RTSIPDLSFCLFELLVFVFNTRFGVGVWVLSP**TSFIIDEETWV

PLGLIWR*LRIRCTVX

>**comp24206_c0_seq1_4** len=428 path=[1:0-373 375:374-427]

ISHQGVHYVSCLRKT*KMRYVAAYLLANLGGSNNPSAADIEKILGSVGIEVEKDKITKVI

SELKGKNIEELIEEGQKKLASVPTGGVAAAGGAGSAAPAAGGAAEAKKEEKKEESESEDE

DMGFGLFD*NLNVYFTQVKKQT

>**comp24250_c0_seq1_3** len=1123 path=[1:0-1122]

HRQVAYFRFVQWTMAATLKPYLTAVKHTLTAAMCLQNFDSQIVERHNKPEVEVKSSEELL

LVPVIISRNERERVLIEGSINSLRVSIAVKQADEIEKILCHKFMRFMMMRAENFIILRRK

PVEGYDISFLITNFHAEQMYKHKLVDFIIHFMEEIDKEINEMKLAVNSRARLSAEEFLKR

F*ASQLHSVLVKCPYSEHAMDDKGNFWSQFEMEEHASSLRPAQSLRHVLMDGRKFCKYVC

IVTAMFEYRESVGQRCILNNVPGKCYNPLNHTPRVQKGVRLRINIQYKLKVFVRVQY*QL

*KNYYCKPWSI**GS*FMNSYVMEVKRPCLLCSTFV*HIRHTKSLGHIRSFQMSLL**IF

VSHTRGFRAVG*PX

>**comp24679_c0_seq1_5** len=1229 path=[1207:0-1228]

SEVASARLKTPKMNSVICVLAALACTCTALTREGIWLQMHFGGGVKDGENIPQLATTLGE

KTLVELVTKAGLADTLSSTAPPAPFTVFGPTDAAFDALPQQIKDLLNNVTILKDVLLYHV

LSGEVYSTQLTNELVAKSLSNGLPIRINIYQDGKVVTAQGSPIVLVNQNATNGVIHGLSR

VMLPPAGTVVDVVSSVPGFNDLKTAVIAAKLLNVLAGDGPFTVFAPTDAAFAKLPPGTLD

NLLKNITQLTSVLEYHVVSGTFYSAGLTNNMKVTALNKQDFTVMVSSGGVSINTGSGTAN

VIAADVSITNGVIHVIDSVLIPPTMLYHLQK*TC*IRLAPTGL*CECFLPY*NYWLDNCA

PV*TISPLSHAPTLRITIVACGRYFIDVQRVVNVSASIVPTVFTFISLLX

>**comp24946_c0_seq1_4** len=554 path=[1:0-259 421:260-553]

LTDRMICLM*LHHVPNHPLDCLRH*RVT*DLTRSYL*VVKRVLCYFSFTFKVSTHMFR*P

GVVMIAARFIRTLL*YIAGVYACNQFNSGTVIKSTLQGRAVHADMTDEEMVEALATRDIN

ELFEVEQLSDADKAELNNIQREARDLMDQLDALDD*KASFNTNMYMTKYEKHDAFVIS*I

KRSN

>**comp325898_c0_seq1_3** len=230 path=[1:0-229]

SKPKSRITAMHLKHIFILLVVTVAVSSAYRFQSYRNKSPYIRYDYEIEMPDLPEYDFPMY

MLGGGRRGGRGGRRSQ

>**comp328175_c0_seq1_1** len=225 path=[203:0-224]

DAIQSAANNYTRASREFHASLQKIDMTNPIAVRGVNDKLMTLEKAFQVHTGLPTRKEYKH

VLLSPSSADYYKTTT

>**comp338365_c0_seq1_4** len=434 path=[1:0-433]

FSDEETHPEAVQPYNAFSPSGQPQAELVYVNYGRDVDFENLTRLGVSVSGKIAIARYGKI

FRGNKVYFAHQYGAVGIILFMDPGDFAKGDNFYPESMWLPPTGVQRGNIVLIKGDQATPG

YPATWYTSRLTEEEVKMRMPQIPC

>**comp40287_c0_seq1_1** len=308 path=[1:0-307]

AVLVFVLLVAFVGAQRQFVNYCPQHNCPFNSGSRLDQCLSDTDCCIGKSCCVDPFSCIRE

CVATDPRLLGGFNNRF*DVICSDLSRDRRIVNGSSMGNIVPAA

>**comp42014_c0_seq1_4** len=750 path=[728:0-749]

LTEEEVKMRMPQIPCHPIGYSDAFMLLREMEGDAAPADWQGGLNITYRLGGTMKDGKRVK

LDVRNHLKTVTIYNVLAKLEGTLENDRVVLVGNHRDAWVYGGTDPSSGTAALLEMSRVYA

KQVKNGWKPRRSVIFCSWDAEEFGLVGSYEWVEENVKWLQSQAVAYINIDLITIGNYTLN

LKATPTLKQAVYRAARKVPDAHMNGRTLFDVWRERNPDALDKAYPRVTLPGAGSDFLPFE

QDVGIPTTDG

>**comp424095_c0_seq1_4** len=436 path=[1:0-435]

WGGNLDCSGGRNADNCFSTRYMWRSGGDGVVYAYIPDSQKTGFCDRNHVHCNYDYGHDLG

RTWAFKTGTWQKIRQTVKLNSPGQQNGVLTVKFNKKKVLQMKELVFRESDNIEIEGIFFS

TFFGGGSPSWATPVDTYTYYKDFKL

>**comp449303_c0_seq1_5** len=401 path=[1:0-400]

NVTHTCTSRLTQKLNVYT*DE*FMLSSKFVHYRMVCMHLHVCLPVLVRSNTGWCCSAGQM

RHYRYLTETQYTNSNLTGACLHAECHTSTNKY*HPQL*LGQWVKLLTFHSLEDAD*NAPT

DKMMSGQLLFSITX

>**comp44977_c0_seq1_2** len=894 path=[872:0-893]

EAPGESYCYVGKFCSGSKKSEFTAEGTCCTEGGSWGNKNTCNICKKVNATVIGGEGDFRT

KTCRTFGECSYRTFDGIDYNYCSTCTMTLIQNTNIKVTAYTSCDPGSTCKCKKSISITMD

GKTYEYSSATGKVKSPDGTEIDVTSEPKSLGDITVYEKLERKNFKLSKFEITVGVDDDGM

GVVTVDRGTLEDGGFTGMCGNGKGDQAAEYALLSNPIEVKGIAGNFRSADKCGEGLRRCA

AGDEEDQANKACAWLRSSGFRTCNDIVDPALSLFECKRAYCNAATPEKKQEAVCNTLS

>**comp44977_c1_seq1_1** len=283 path=[1743:0-282]

AVCNTLSLYASMCISKNVYFKWRTKNFCPKQCPAGKIFSVYASPCPAMCGMLDTDNLKRL

TSPSCSQHMAGCVCESGKILDNAGNCVAPNDCKCX

>**comp46614_c0_seq1_4** len=554 path=[1:0-553]

TSGTTTTSGTTTAGTPCENIMEGQDLPSAVKKVGSSVGIYESGDIVSTTEPSDGSILNPL

EAEEATVSLPGPKDIIVITTGASPDALKKDNSVVTPSETLSFDDKTVYVYRDAPEGTYTP

VSGSDTFVEENAPQNGDVPLSGQRFIILSDSPTAPVEESNIKVKICKPTTGTTTSGTTTT

SGTT

>**comp46746_c0_seq1_2** len=1743 path=[1:0-1742]

VRLTQLTYHIIVGGIAITWDQGLYVKLEVPESWANQTRGLCGNYNGNSRDDKTLQTGALA

ADAAEFGNSWRVSPDTCVEYVPTGKEPCEVNGDRKDWATSACSVLNSPEFQQCKNKMATR

THTLFKFCQSEACGCDKGGDCECLCNAIATYAAECMNLHGIAIKWRTQRLCPIQCEGGSQ

YRACGNPCQPSCAKPTISDTVGEICNSTHCVEGCFCPEGSIMDEETKQCVPLEQCPCKDN

NGFIYQPGQNFTRDCQLCTCKEGQLECTGKTCGECDSTQHSCGEVQGVLMCISKDKVCDG

MRDCSDGSDEVKCNGTCTDLQFRCNSGQCIPKTFTCNSLKECDDGSDEISCNVTCSNETF

TCADAPTMCLPMSFVCDGEADCKDKSDEAGCHVCPKNETQCVENDSCIPKDRSCDGHDDC

GDGSDEKGCTTPMSTTPECDVEKVSNGYLIVQTKDNNVMAQLAANGGSFNTTVDGDVELE

LTIIAKAGTEPKVFSVSFGADNMTDAKVMIETLEGGKKVYENPTIENDIAKFVITGDKVD

KILVEIKVLLTVTVIHDIEVEACYEPGETTIPTTSETTTSG

>**comp48398_c1_seq1_2** len=350 path=[328:0-349]

PSLDILTTHIFENKVLEVSGLAIKYRVCVDREREANLPSFTCTDLWFALN*ITAPLSMVE

PVKAI*KK*ILYTATASDSAQ*LLGPLR*TVCMLRLTNLPMNTAAKKTFTAVYH*IX

>**comp49086_c0_seq1_6** len=655 path=[653:0-32 686:33-654]

TTSGTTTTSGTTTTSGTTTTSGTTAKVCEDNDSESTVTLTSGPLTDATSTRELVREEIQG

EIMSFESPGTEPVKIFAIDRLLTVNDGDAVTGPIFIEEATVGDKVTVTAGNAETFNDKFT

VVEPAGIDQITISSLSPGDKIYSLTEVTDAALPLKTSPTGSTELLVIANKGFSFNQVTIK

ACVKTLTTTATTSGTTTSGTTTSGTTTTSGTTTSGTTT

>**comp49884_c0_seq1_4** len=375 path=[1:0-374]

PLFYGFGTDSIAAIAYQFNDTRNEFENTVAVVSRRDCVPIISTTTILRPGNNYLKALGYN

NIFPGIRDSSVFDPPSYCRKSAKNAVNTVYDIRRV*RSFTCLTV*YEAGNAIVEQRTN*L

*LRIK

>**comp51373_c0_seq1_3** len=516 path=[494:0-83 578:84-515]

KRLNMSLRVTSLVILFLGLALLASSSYRQVCTLPKDSGPCYADIPRFYFDYRKGVCRKFV

YGGCYGNANNFHTLKECRAICGGGDQCRQRPDPGHCYASILRFFYDKFNGTCEPFYYGGC

GGNCNNFPTKQQCQFQCNC*TLISDSLFQVTCHCIVCKESFRWR*CTERFAX

>**comp51688_c0_seq1_2** len=594 path=[572:0-31 604:32-42 615:43-593]

TTTTSGTTTTSGTTGTPCSKVDLMNPQDNAATPTPDDLGLPTNTLLEFPLSDSPLVDSVT

STSPSLYSPVEETSPGGPFAVQPRTKKILLITDTTPTDVSVNGQPVDTTTSVSGNRNGKT

IFVTVVDITEGAPVTITTTGGATKPEVFEEVEGEKIPGTDSFKVPINKELSSIIVPKGTD

VEKIEICEHLTTGTTTSG

>**comp55503_c0_seq1_1** len=666 path=[1:0-25 27:26-665]

MLQAVRRSCFGAFRAVNNALSYPKAYPAALKASTTYFTTRNPYGTQAASPAAAATASGRV

VTVIGAVVDVQFDDALPPILNALEVVGRKPRLILEVAQHLGENVVRTIAMDGTEGLVRGE

KCLDMGNPIKIPVGPGTLGRIINVIGEPIDERGPIDTKHFSAIHQEAPEFVEMSVQQEIL

ETGIKVVDLLAPYAKGGKIGLFGGAGVGKTVLIMELINNVAK

>**comp63434_c0_seq1_2** len=1168 path=[1:0-940 942:941-941 943:942-965 967:966-1167]

INRFTGKTRYSISSNTMQSIMALGLSALCVVLYFSVANAASGDCGTFSKAKFDVCVAAFS

TANVLNLDDRKALIAADNTTCPTDILSKSCTSFNVMIKCVNAFGFPEGCEMEMDKELKNQ

QFECTYKELAMLCPDATTIGGAAGAIASMSLLAACLITAIFRF*TYTNKFF*QEGAQILR

LHRNNLTRKYLQE*TSVSR*TDRILFSNEKELHNIITWVKRPPRSMILPQQTCAGALRVN

RSYLPSTTLALTMLCQYPRFFHFESC*CNYNMTIICTHNSICAFSEPMLNVCSHFLSFLF

ILKEELCWFR*GP*WSVKQVRYGIILNIRSILNGTCKNPEFR*CFDTWLFTH*THTENSY

LNPSFIFLIIYIVCIYLSLN*DINI*T*K

>**comp63526_c0_seq4_3** len=1096 path=[1:0-862 984:863-1072 3475:1073-1095]

AGAPLRPSFLAIACSPHSARTSPVRVRPRRSFSSRSAAGNRAEATESAMEREKEQFRKLF

IGGLSFETTEESLRNYYEQWGKLTDCVVMRDPASKRSRGFGFVTFSSMAEVDAAMAARPH

SIDGRVVEPKRAVAREESGKPGAHVTVKKLFVGGIKEDTEEHHLRDYFEEYGKIDTIEII

TDRQSGKKRGFGFVTFDDHDPVDKIVLQKYHTINGHNAEVRKALSRQEMQEVQSSRSGRG

GNFGFGDSRGGGGNFGPGPGSNFRGGSDGYGSGRGFGDGYNGYGGGPGGNYGSGSYNDFG

NYNQQPSNYGPMKSGNFGGSRNMGGPYGGGNYGPGGSGGSGGYGGRSRY*ASSYLPWEGV

LLQVT

>**comp64297_c0_seq2_6** len=650 path=[1:0-380 382:381-407 409:408-462 1361:463-464 466:465-649]

LEAETAKMRLVVAGVLIAAVLGMAQAHYPTDPEDPYRKDPYSKDPYPTDPYPKDPYPPAP

QEAVGPQIPGAIGGPQIPTGPGGPGGPGGPGGPGGPGGPGGPGAVRRTFRPRRNNPFIPN

IHVIVNTNQRMHNNNNAGRRGSPGLPFPFDGPGNVAFIGIPPGNVFRPRPRFSQPKQFG*

HESLQALIFDLRQHQALLTQDSNESCWQWQNHEFNCV

>**comp72472_c0_seq1_4** len=918 path=[1:0-917]

QDSRSRTCCSRKSGTMSTFLYMAVVICVSTCVQGFQTSGRIDTEKHTFVMQSPYYGLYGT

ITFTYEISFPEMYGGIDLLAFPRNEMDMAKNSNTSCSDAKYHWHRTLHSFEPSCRYNGED

IYGRMLKCNGRFDLTDYSGQEFYFAIRNCNGMGIRGTHYNLMEGMEVVATASASVSVPST

SCILVLFAAMATVLRI*RCLYVHL*GSFAGDPASLCNQCLV*Q*A*SDPDFHLRCSFVYS

KVHVKQRLCERTRI*IQTVFYRRTLNIINCSSDLQ*RDVNAALV**SNANVALALCYQLL

TD*VWF

>**comp7312_c0_seq1_2** len=228 path=[1:0-123 125:124-227]

LTTPTYGDLNHLVSAAMSGVTCCLRFPGQLNSDLRKLAVNLIPFPRLHFFMIGFAPLTSR

GSQQYRALTVPELTQX

>**comp75441_c0_seq1_1** len=683 path=[1:0-682]

QASRVMNSYRFVTVLSKICRVEPRHSLTFVHKMATEAKRGKLSDEERSSSLSPLTAVGWS

MVEGRDAIYKEYLFKDFNQAFGFMTRVALLADKMDHHPEWFNVYNKVQVTLSSHDVSGLS

QRDVKLATFMEKAAKTMQD*QSWPAPNIHSMSFLVKLQTD*SQINDD*PAQVIVNE*GHL

STYHSDGLHNMMINILSKVRRP*MRIFLNTPF*LRERS*FIARGV*WX

>**comp76692_c0_seq1_2** len=541 path=[1:0-204 206:205-224 226:225-303 305:304-540]

QTQEETMVLSGEDKSNIKAAWGKIGGHGAEYGAEALERMFASFPTTKTYFPHFDVSHGSA

QVKGHGKKIADALASAAGHLDDLPGALSALSDLHAHKLRVDPVNFKLLSHCLLVTLASHH

PADFTPAVHASLDKFLASVSTVLTSKYR*AAFCGACLLAMPFFSPLHLYLLVFE*SLSRK

>**comp78504_c0_seq3_1** len=1908 path=[1886:0-39 1926:40-689 2576:690-1360 3247:1361-1907]

KKESMSYTLDYHDRPQTQGSYDDEQRDKVEQRQQPKQSDDKRSFDDNPDDKEISRDAIPD

TNMVIESNWTSVVESFDDMKLNEQLLRGIYAYGFEKPSAIQQRAVMPCIAGHDVIAQAQS

GTGKTATFSVAILQRLDISIKSCQALVLAPTRELAQQIQKVVVALGDYLGAKCHACIGGT

NVRDDIRKLEDGIHVVVGTPGRVFDMINRRVLNCKHIQMFVLDEADEMLSRGFKDQIYDV

FQYMNANTQVVLLSATMPVEVLEVTQRFMRSPIRILVKKEELTLEGIKQFYISVEREEWK

LDTLCDLYETLTITQAVIFCNTRRKVDWLTEKMMSRDFTVSALHGDLDQKERDVIMREFR

TGSSRVLITTDLLARGIDVQQVSLVINYDLPANRENYIHRIGRGGRFGRKGVAINFVTEA

DTRTMRDIETFYNTQVEEMPMNVADLI*FKPNLGFSSLVPAEKQELVTRILFEKNLKLT*

CIH*LLRNVYCFLMLTVCKYGHMRLSAV*SISHVYTN*IMPNY*QHSLFLLPNCFLCCAC

KKAPQYHILILCNTKHL*IFKNYKKIAIVI*KKM*VKKIPVQKLKCIILCCSSSIALWYA

SKPTFLPPLIGAGGSCWSARDRDYHCSKKRGKNILD

>**comp81221_c0_seq1_1** len=1356 path=[61:0-1332 3508:1333-1355]

VVC*LSCELIY*SR*CHWAKTARIVVASKDGNIC*KKPVSYLGNYRVCWRARRLVPINL*

TSLPSVVCPLSLGDQTKSTTSSP*SSNTRLC*YSQTSSMSTKLDKDDVRAAYEDVRDDAS

ATLWAVFKYEGNTIQRSATGDNYDEFQSQFGDDERAFGFIRITTGDELSKRSKFALVTWA

GSLLSALKRARLSTDKSLIKQVIQNFAVEIMTSEQDEIRIEYITDEVKKAGGANYGTGQ*

*QRT*CS*TVLTMLNVNRND*NCGL*AEIFKDWNSAVSLTTVPCVWGLLEHMFCGSRFEN

WGTDFEEAASGSTTPHLKRLTFTVIFS*IFLVIFSCTFVSNAVSNSG*LSHITEVPSVQT

KYT*CCIVNPD*LVDLIFVYSLLLFQE*QWKHRITTSVQLRADSAMQCIHEDHLCNDFMQ

SHHVFNH*QEDPES*SNCDFLQPLWY*FKQFS

>**comp81189_c0_seq1_4** len=2849 path=[2827:0-1522 4350:1523-2848]

ADVSKLYGRHLPTNQVTAYCRRDLLRLAGYRVVVEQLWRSAGESEQTTSMSEGYALLPVE

EDAEIPQTYEGFAKNRRHGDTLQVPYKGLVRMNKSSVLKVLLSLSVLLNLALIGFLLWTR

FMPFDYWSKDDQQVSQTKPVIGLNGHSSSTPPSVDPNHHAPTVPTQCGGKSSSRLTPPAY

KNIFHDLRSEEITSVKEYLFKVKDFDIVEAFQAELTSNFIFSIELQTPNKSSALHFLDNK

GRPPAREALAVLFLPTFNPPVVEEYVVGPLPNPTYHYRNPRRDKSIDFRVRQFNNVEMGH

AIRTVQNALGSKIEEFLHKHYQAHFFGCSGQCLTFTSSAVSSAFSKERRLWINAMYMVEF

STLHALGFQFLLTMVGLDSSKWYIEKVWYADKLYDSFDDLIEKYERGETPPINLKFPKVK

AGEDTLRGSLNIKGKQFPKVPQAGPRQYEPQGHRYTVQDEHVEYLQQWSFNYRMSYASAI

QLYDVKFGGHRIAYEISLQEIVVLYAGANPATMHAQLADSAFGLSRNAHGLVPGVDCPDH

ATFLPVTLFGADHPNPLDYPNSVCIFEHNNNVPARRHKAHSYRSGSYYQGLVDTVLILRT

VYVMYNYDYILDFIFHQSGALEIKVLSTGYILSAFYTEEEKPFGFRVLDDTVGSIHHHMF

NFKIDIDILGPNNRYETLNFKVDNTTRSWYDNGQPIPQLSFERDLKRTEQEAAYKFNFDT

PRYHVIYNNNMENVFSGKRGYRLQVHGFSKQMIPEDLGALNTYTWSRYQLAVTKRKDEEP

VSSSMHAIFDSADPVVSLQTFIDDDEDIVDQDLVVWATVGMLHLPHTEDIPNTPTTGTEA

IITLLPYNYFQECPSMGARDAVRIDGEPKFSVNNHGIPQNVSCVPSHMSNDDISKQKDHI

FMAAG*S*RIISKHQKQSSFV*ISSFLYICHVKLVSVHLCSISLLLLKW

>**comp82066_c1_seq1_4** len=1285 path=[1316:0-668 1985:669-669 1986:670-693 2010:694-784 2101:785-1284]

FGISKAMEVLAVLSVCLLVSLCHSASPYLKFVSKFSKDRDQSKWPPKFCHGLDCPSFTVL

KSVKGEYEAREYSASQWVSTNSAGVDYAEASRTNFMRLFKYISGKNSAGDKINMTAPVII

KIIPGPGPACESNFTMSFFVALDNPPRPTDPKVSLQRFPSFRAYVRSFGGYYMESITPWL

KQAEKLSGYLNGTSYNTDYYYTAGYDSPFTFFKRHNEVWFIAE*LNPRY*KKASQGIHSC

YFTDLNRSFFSTSSLMNLSNIPCLIKRDLIRLVKMRNR*KDWYDLMSEVVLMCVSSPAAI

YIVNTCTCRIIPEMRSVQKSV*Q*RHIFVDNLSLKIPVQDKYY*KC*LPANFFILFILTL

SFHINT**FESGSHKCLFQ**KYVWKNSNISHL*CFSCCAFQLLLLLGL*LMPQILFDLF

CLHFGR*K

>**comp82828_c0_seq1_3** len=1320 path=[3184:0-142 367:143-1247 1472:1248-1319]

MFGDRYH*CAERRFAGEEFVFFSFLQKMSSFRSYRAPKHGDARDIQIKINKKYDAGLAAE

MLEWISSMTGESFSTSGDMDNFQETLKNGVILAKLANVLKPGSIPAKKLTPSTLSFKQME

LIQLALDVFKKLDVKETEIFQSVDLTERVNLNQVVICLQSVGRKAGHGPKESKENKRDFS

DEQLKAGQSVIGLQMGTNQHASQAGMSIGKKRMITNDEKQ*NQQSHVVTST*SIQTSNTL

LLAFHTDDLAGFTELARDQPVSMTHFV*SS*TMGVSINDIAVRFRTHRHDLTGVFPWDQL

*V*TNSMGGLMLWWFHQ*SCYQTRRVRQC**RYL*ICISYC*NKYVLEHCA*CV*LEWNL

QFGTFCQIYMYITHISYHKRNISKRRFLYFAHFHCHPGTNFNSYIIIAIFAILVIETSLH

MLRLDSCVWRKWTGIKIYSX

>**comp83123_c0_seq1_5** len=1212 path=[1190:0-421 1612:422-427 1618:428-1211]

L*RKIICLPVFTNILLSSIIKFYSA**SSLNVRRNENISMTMV*QEDGSLNGYLAQEIAR

NIRLLPNPTTAEPKDISVAPSERTNFEQEWR*CFGFTRL*YVIDISYKVAEENLWRMNAR

SQVFELSVEGRQIEEVVQSIFHTLLMHRTLGKFHYKQEGSYSIGTVGIMDVDCDYIDFTY

VRVASEELDQNLRREVSMFRDTLRSHDGPGSGQISLEFYQKKRARWPFQAECIPWEVWTV

KLDIITLANEHERQVCRERLGEVLAEKVMYVADTMNRHEYVPKMPNQSDLDLIFDTVHFD

VQPYLYRISHQTTGPAASASVGTTMRKFLKDTLAL*QILDVGDNDLTSSTLY*YLPLVLC

ARD*LSVY*LGYAFMLIIVRLNNA*VQLGVMWLLSADNCLACFX

>**comp83798_c0_seq2_2** len=2287 path=[6633:0-39 4063:40-1536 5562:1537-2270 6296:2271-2286]

IRYICHVTRILFAQVYPTLIAKVHNQFVPLDSIIYNHTALRDTVTGNFLTEISWFPFNSV

TDAEAEDYRKDGTIPTAWNAKRDFIWLRSITIVSEAELGGRPLEPTHFLPTKGSLSGGSE

KGLLRGREAVAIAKRVPTVSYHHLVNAFPVILPPRWGTETSAAFMLNIDNQFTRAAAALQ

FIIEEAENQIKSNGSTPLNALLPRFFENSDCDLCPGNYDISQPDDSNRTLVIDFLAPPAQ

YGFYPTARKFVDHFRDQKVRPHWGKRHDNIPGIIDHIKLVYGDNLDHFQRMRKLAQVDPC

DMFMNTYLIEIFGRSGNRWCA*PVDTHYACVIPSRFLNHYCRHSVD*YATGDGNRRLKHI

CE*IHGIRDILLFPTTFNL*LPSTSKNGVT*RDVKVNATNSWYVLSQ*VSICQLTLYQEV

KITFVI*NKRRTCGHFELTTIFGLTGDDIGEQGTSFLNTGKFQLTLVLIFLR*IVICNCN

VIFQARYFAFIESQTIG*DDCYVCTRL*RNLYQSV*AVIFVQDCKGIFLYIYHSQIELSR

CLYVDMSRCYIQSVTTAVGLREKDGSHNHIAFGSL*RMWRLTIPLHELSPFGHSTR*RKT

QLIDFWQRYGRHEGMTKKLPRNYFYEIYLFW*GTALSHRRCSIALEH*TRYILLIFPIML

NYSAHSRVYPLCLSMGRRLMVSNALFVSLLRRGRCLNMCCL*LLQHQFSPGFQTQQNFIG

DVRQNSKPE*LYYS*NITKDVHVCMYCVLCCRVLCCHVLCYR

>**comp84429_c0_seq1_1** len=1376 path=[1354:0-61 1416:62-65 1420:66-406 1761:407-413 1768:414-1375]

ADSHLQ*NRQ*SS*D**HVLAWR*GGTKSRSQTQRMSLAEPNTVAPTRCQSNDSLDTGCR

YNMTSRTKAIGTC*RALAFSVGCRWRLFH*PSADSHK*VCSDKTLWVPRMSPVVLFVFAL

TIVNGAPTKESLLPRVIEAVDRAIKFFSSDYSSINVDGLFGLRIGQGQIIEALEECESRP

CSPELKDLLRRFRSELEVTCQKAMPYIEQESPDYFARFRETISKPYILKYRPFRFHGMED

VEIGHNKEYDEEEGDRCYARLLGTYQENDRTIPRCNVTQPCLDLVTKGDTKRYTITHQLL

YFIVVEHTGCDEKLSEQSSSLSASGIREIEDRFCQKIYKEASILAKEGSVNIGSQDLFLE

QSVLCGSLGYENFFKPGYIDMVLKMQAPEGCFTMGFETDSVILATLERLRTSRKLQREQA

MKDGCLSHKSGLGIGTLALYLRYLVRSMI*RRGY*THWX

>**comp84564_c0_seq1_4** len=210 path=[1617:0-11 190:12-39 218:40-126 305:127-209]

CRDTVIVKMKNLFAALLMALVLALSQATYQGYDRKYDYDTPGYVGYDYPKGDSYRPRYDY

QKPKYDSYRP

>**comp85374_c0_seq1_2** len=2398 path=[2376:0-114 2491:115-316 5523:317-323 5530:324-457 2834:458-1083 3460:1084-2397]

ALQGRLFLRRVLKGRCFSSEHSKSSNKMSITTLHARQIFDSRGNPTVEVDLRNENGLFRA

AVPSGASTGIYEALEMRDKIKGDYHGKGVSNAVNNVNNLIAKELVGKTIDLKDQKAVDDL

LLKLDGTENKSKLGANAMLGVSLAVCKAGAAAKGVPLYRHIADLAGKKEVILPVPAFNVI

NGGTHAGNKLAMQEFMILPTGASSFTEAMKMGSETYHHLKAVIKKKYGQDACNVGDEGGF

APNILENKEGLELVKIAIENAGYTGKIQIGMDIAASEFCKEKKYDLDFKSKDSNPAEWLS

SDALADLYKSFAEDYPIVSIEDPFDQDDWEAYTKFTGSVKFQVVGDDLLVTNPKRVQKGI

DIKACNCLLLKVNQIGTVSESIQACKMSQDAGWGVMVSHRSGETEDTFIADLVVGLCTGQ

IKTGAPCRSERLAKYNQILRIEEELGSSAKYAGANFRRPEA*SERNC*IQNALCKNNYIV

CSLLISIAF*ICHINMSHREK*LSPT*SSATSAVGW*PETAYHSGSVLLMF*FHLLFCCI

FCYQRFI*NSCWYIVIYCFHQND*RIMSWYNNPRFNLKK*LDFDVTPMQMALIEDRHIIS

CETFSVHITVVM*LYVMNGCT*LHS*SWQQLFIEVLSTGENVNAFTLTLHRSCGHCQESP

*LDSVFPQQGTVDTFLRRVKYITFTVEHFTSALLSVAKYFRVDECSELFSGHCFVEQGRT

LKS*VPIPALVSQCTVSDSVQS**CEVVASLGCVDLCSDVGLLLLQILHHSWLIQC*PYQ

VLMFCFLLHVLHPLNKISH

>**comp85491_c0_seq1_6** len=790 path=[2047:0-179 1021:180-205 1047:206-230 1072:231-326 1168:327-439 1281:440-479 2760:480-484 1326:485-497 1339:498-613 1455:614-623 1465:624-659 1501:660-662 1504:663-703 1810:704-789]

EPDRPVAAMHHLLPATLAVLCCFSLVTGYDLFVDLTSGTTLQTSSSLSWATSLYGSGTCG

KKGVLKLSFGAPGSANRKNKVLIDMWFNNPSGWVFNIGDSPTNNGYGGDGATTSRDAEIQ

GTSSTFRIYGNDQNSPPGGILLTRPNIITNRLSVIIADGLVIWNNWGSEFNYYLNTNLLY

ALNGQSDSEGGSPNYDIYFGINRSIGSGGRVGTGLCHAYVTWLS*TIWTA*DP*ECD*RF

ETICRSLL*SVIC*CDHQKIELP

>**comp85508_c0_seq6_5** len=3318 path=[11509:0-129 7442:130-1966 9279:1967-1986 9299:1987-2267 9580:2268-2902 10215:2903-3317]

PPHISEM*VLVNVKNGDMNR*SYLCG*VSLQPFAHYYRCSFLTCVLRRTYIFVKKCSYHL

CSNQMATNFSSLIIRNRATLACVPL*LQIL*PSHLIMLMLLSQN*SVLKEYAHQNCRSRG

*FL*LKAHIVSDLTHSVTMAYTKWKSDDCKCRSHTFNSDQLKLALPFDHRGGLL*MDWTT

TPLYVHK*GIF**ACKHSSSKPTTTHNDQSLSYSTPFSLRYKLTCCKI*RRVRGNVLRVD

INFQMEAVCHTGGR*KQE*ICQ*YFQTVST*CIQSSSTSIAVSQLFSWLVDQ*SEAAELR

MRQLGTHLYIDAQLTGGKLTSGTNRIA*RTVGNGNHIAKTDDSSITIVDIHPASTSEYSS

NGNNDICTAGRSGHNMH*SSSCGY**ECYILMTLVTGFSYCLICLLMRIITRIVSLVPNK

TAFPT**TQKVF*FLLKQTYTPKTSQRLQNLHYLDRVIKERQTHVQLLSSWKYFT*ASSR

DTAIDPHHIASITPTGTQPASLHHIIPQ*SHLWICLLRLCHPS*N*YCTVCGWL*AVT*A

PLCTCPSHGNTW*TSCVPCTSWPTEVCGDAQAA*SGCVGCKPVHF*MLV*SPHHWSSCQP

RGRLALHYPES*HHHQPDHPGKSLHISLIQ*SIPSHWSIETGDSTS*TQHRDHMAVAHQC

RPRRSSLALHLR*SSGWCIHPCTSCRQSALPTWLHRGLPSSQPTHTQCHACPHYQREP*E

CSKNRNSEEMLRLVQSAHIW*R*DCH*LSVGRTQ*MVSLIPCLMFLMDACQCG*QTCA*S

GIETSDSLVVDVVVPLPTCPETELQTSSVN*R*R*ASPCWKLSGPARSTQYTCIHPLVHP

LHLPPHLQVVPLAPSTDTGTHSCHHCRTHLVLPLGGAQTSCLPTQKTPFHPDPHSTQKPV

SQLHQQTCQAVLA*RVPTDLSSSCHAP*SP*DRHLCE*QTLQCGCSQQEAHHPSQRTLS*

RSAALHLLNSGKHWHTLAFPSLNTVPEKASMLLLESVWWISCLSRQTRQSCSRGYTHILC

PVISAPTLPWEFQLGAS*GSLSRVLPCINVVLSCQNRTVHFPETI*NYYNRYSI**CSTS

*WGSLLANVLQECSSGVN*KFYTDKX

>**comp85564_c0_seq1_5** len=727 path=[705:0-332 1640:333-341 1047:342-407 1113:408-408 1114:409-430 2057:431-624 1560:625-633 1339:634-726]

VAHNTLQGTD*EILTFEMQIFGVLVLIALAGKGLDAVKTSGPAASAKAPTAAVAPTVAPA

TGAQSLPIYRPRQPATKPAVNSAAGSGGYNPYLSHPSSPFRGINPLLLDPDLIYPDPLID

YPLDPLGHDLGYLNPLGPGAHLSPLASLGIAESVYGDLHPSSEYLLQEALLRRAYGAPKP

TPGAPTGAGSPGAGASGAINNGGYNPYDTFLGQYLVGF*ETSSRHIDKRLKYKCGFTIN*

NLX

>**comp85761_c0_seq1_5** len=587 path=[1:0-73 75:74-94 96:95-115 117:116-118 120:119-149 1822:150-153 155:154-185 187:186-251 253:252-291 293:292-318 320:319-325 327:326-403 405:404-409 411:410-459 461:460-463 465:464-586]

SSHKMKFTLYVCLCLVASSWTEFMVQARGPLVYFRAILPKDTTYGLRSTIVFSQVEANKG

SGYNPSSGVFHVPIAGLYLMKCQLETTSQGTSEFFLEKEGSDQSVLTIQHHHSSKTMSVI

IHLNVGDHVLVRKRYDIKNAPLRGGVWSQFSGYLLRKD*IKCP*IGVIKIIMNCHDESFI

TQSC*TFEINCIFDGX

>**comp85967_c0_seq6_6** len=620 path=[1:0-477 479:478-619]

ITVGGGASCLVVSYRAHPTPLLTSRSKTSSL*ATTMPPKFDPTAIHEVCVRAVGGEVPAT

SSLAPKIGPLGLSPKKIGDDIAKNTTEWKGLKITVKLIIQNRQAKVEVVPSAASLVIKAL

KEPPRDRKKVKHVKHSGNITMDDVFKIARQMRPRSMAKAFSGTVKEILGTCQSVGCTVER

SHPHTIIEKIDSGEMETPEE*TGCSMX

>**comp86036_c0_seq1_4** len=814 path=[792:0-565 1358:566-813]

ADGDTVRFWVRGYDIKSDMKEESVTVHIDTSPPIIENLWLTRGDRLNLNVHNVEEFAEMT

MEWVAYDEHSGLETVSWQIVDRYHGRNIVHGIQHIAAQGETTSIADCKTTYAGVARGANC

YCTPVKGCYHRHFQVKPTVVDSSLSHGGIFSDKSKGSHDADYHLEVTVTNHAKLTTKLRF

KVTIDASPPSPGVVHDGQSGRPEVDYQNSRQLQAHWDGFFDKESGVKFYQYRFGSNCFQA

NDFSLEARPQVTETSSTSASWTAPSVGKYHV

>**comp86117_c0_seq5_2** len=748 path=[4074:0-40 903:41-127 990:128-282 1145:283-300 704:301-499 1163:500-581 1245:582-683 1347:684-747]

LLMS*NKMSSCVTAERLHDFTLEVFRKNPTTSTGARSVLCGTYNGRVTEPGKTVSVKCIG

TVRGRYVRLRGKKDGEGDLLQFCEVKVFGSIIADVKYNLARGKPATASSVYGPLPRVATD

GLPYTDWKRGSCFAVAEGDSHPWWQVDLEAYSQVFEVAVTSRSDCCPERLHDFTLEVYTK

QGARGALCGTYDGAVAEPGKTVTVKCTRPVKGRYVRLSGKKNGEGDLLQFCEVKVFGYAF

*PRLIKACG

>**comp86258_c0_seq3_4** len=1642 path=[1:0-745 747:746-1147 1149:1148-1463 1465:1464-1641]

H*ERASKH*RTMGKEKIHINIVVIGHVDSGKSTTTGHLIYKCGGIDERTICKFEKEAQEM

GKGSFKYAWVLDKLKAERERGITIDIALWKFETTKFYITIIDAPGHRDFIKNMITGTSQA

DCAVLIVASGVGEFEAGISKEGQTREHALLAYTLGVKQLIIGINKMDSTAPPYSEARYDE

IVKEVSGYIKKIGYNPKAVAFVPISGFHGDNMIDQSDKMSWFKGWAVERKEGNSSGKTLF

EALDSILPPSRPTDKPLRLPLQDVYKIGGIGTVPVGRVETGILKPGMIVTFAPSALTTEV

KSVEMHHESLPEALPGDNVGFNVKNVSVKELRRGFVAGDSKSDAPKECASFYAQVIILNH

PGEIKNGYSPVLDCHTAHIAVKFSEIKEKCDRRSGKKLEENPKSVKSGDAAMVVCIPSKP

MCVEAFSSYPPLGRFAVRDMKQTVAVGVIKEVTKKEVTGKTTKAAQKKK*LARDKAPWLT

KGKANYNIGTISLLIVICSMEMLAQLVLISWSTWLLQSKLHRKEFRRKRILGH*GLNFIA

TLQMKKK

>**comp86399_c0_seq2_3** len=1790 path=[1768:0-372 2141:373-415 2184:416-1103 5015:1104-1191 2960:1192-1789]

ALTISG*NFRQVLSKARATMRAVAIFVALFGAVFCDPTVYFQEKFEDGWRDRWVDSTAKG

AEQGKIELSAGKFYGDAEKDKGIKTTQDARFYGLSATFDKFSNDGKSLVIQFTVKHEQNI

DCGGGYAKVFGSDLDQAGMHGDSPYLIMFGPDICGPGTKKVHVIFNYKGKNLLTKKDIRC

KDDVFTHLYTLIVNSDNTYEVKIDNEKVESGELEADWDFLPAKKIKDPDAKKPDDWDERE

KIDDPDDTKPEDWDKPEHIPDPDAKKPDDWDDEMDGEWEPPMVDNPDYKGEWKPKQIDNP

DYKGKWVHPEIDNPDYSPDENLYRYTDIGAIGFDLWQVKSGTIFDNVLITDDVDFAEEFG

KSTWGKTKDPEKKMKDAQDEEERKQREEEEKKRKEEEDAKKEDGDDAEEEEEEDDSDKKE

EPDHDEL*RSSRD*PIIFIRHLSPFISCHSHDV*YGKSFSRGQKLSSKLLKEIC*VFVFY

FVFIGHCV*DVLKKSQTAFHPNMPP*TH*KQSVCVKMICMGQSYVLGNGIAVNIVRLSVI

LDDFTPCFVFKYLMYNLSNV*RTIVNFMLLM*IAL*NITNVGVFIPK*GNKCFFLQ

>**comp87007_c0_seq1_6** len=892 path=[870:0-172 1762:173-187 1058:188-259 2040:260-266 1137:267-334 1838:335-340 1211:341-464 1335:465-527 1398:528-891]

YYTISI*SFTPSVKTDHLVVFYFEDCTIRHSIMPVLSAIAVCVLLLGCSAAVQGYHVVGK

FGNQIIPFDITFGKDIMDVKAYKPANAPDNFRATSNLHVFSTNFVAIKNAADQQCYIQRF

NATMAGMKKRVEEAKRANGVLDGVKEEWVRADKLFPLKTWTVELLFGQQIADFCADHAVF

LVDRRYSGLVDPTHVNPSMRRKRGLFFNWRPKVKCVLQIAQRLTSGYVYDPCNTNFPIVC

*TNCMLHNKETRPNGANYCLLIQLLQEVTPSQVPVCDAIQVSHKSAFQDEYIFVNKT

>**comp87087_c0_seq1_3** len=678 path=[656:0-151 808:152-181 838:182-231 888:232-241 898:242-280 2569:281-285 942:286-309 966:310-358 1334:359-407 1064:408-470 1598:471-560 2623:561-577 2640:578-578 2641:579-580 2643:581-582 2645:583-585 1541:586-595 1252:596-677]

LVFMAKGKLIQGPR*RLGVCLCITLLAVCETPF*W*L*CSP**LLPNGFQFFPI*E*AVN

GSTT*LRWYKMAANLHVLTPLILLALFTSSSGWFFRRIKLHDVKNCFCKAVNNHNYGQVF

KDFGSIQHCRRFVHCPCSARELITCGTECDRKVKEWACDHGCGGLPAGTKVRASYGASVC

NSGRAADVYVCGKKNNCHRG*VTHVVMPVTRCSN*RPE**KH*NCX

>**comp87110_c0_seq1_1** len=316 path=[1126:0-51 3231:52-62 1189:63-150 3358:151-162 3370:163-164 3372:165-170 3378:171-174 583:175-315]

LRRKSEISTMKTFAVAVCLVLMVAMASATFGGRGYGYYPGYAHGGNNAWNNQDFNQYATI

SQQQDNKQKVDQDYYYRRGSGFYFPRGKGYYG*YDCPFTLSIIQDX

>**comp87110_c0_seq4_2** len=388 path=[1771:0-159 3378:160-163 1300:164-187 3405:188-191 1328:192-197 1334:198-387]

AET*SSTMNTLAVALCLVLVAMATATYPPVGGDRGYGAYPGNSVDNYQYPTQVANINQQQ

SNKQFADQDYYNRGYNYGVPYYGGYRRGYYPTGKGFRY*DASSTMSITPYYIYCVCLLYD

INKLLCKLK

>**comp87346_c0_seq3_4** len=2350 path=[2328:0-663 2992:664-723 3052:724-923 3252:924-1631 4726:1632-1656 3985:1657-2349]

AKSR**RRVIELEAVDGEDIRASYKHSHRQTYSRTMAKAPAIGIDLGTTYSCVGVFQHGK

VEIIANDQGNRTTPSYVAFTDTERLIGDAAKNQVAMNPENTIFDAKRLIGRRFDEANVQS

DMKHWPFNVLSDGGKPKIQVNYKDEPKTFYPEEISSMVLTKMKETAEQYLGKTITDAVVT

VPAYFNDSQRQATKDAGTISGLNVLRIINEPTAAAIAYGLDKKVGGERNVLIFDLGGGTF

DVSILTIEDGIFEVKSTAGDTHLGGEDFDNRMVNHFIQEFKRKHKKDISDNKRAVRRLRT

ACERAKRTLSSSTQASIEIDSLFEGVDYYTSITRARFEELNADLFRGTLEPVEKALRDAK

ADKVSIHDIVLVGGSTRIPKIQKLLQDFFNGKELCKSINPDEAVAYGAAVQAAILHGDKS

EEVQDLLLLDVTPLSLGIETAGGVMTVLIKRNTTIPTKQTQTFTTYSDNQPGVLIQVFEG

ERAMTKDNNILGKFELTGIPPAPRGVPQIEVTFDIDANGILNVSAVDKSTMKENKITITN

DKGRLSKEEIERMVNEAENYKAEDEKQKDRIQAKNGLESYAFNMKSTVEDEKLKDKISED

DKKTITDKCNDVISWLDSNQLAEKDEFEHKQKELEGVCNPIITKLYQEAGGAGGMPNFNP

GAAGAGGAGGAQTGGSSGGPTIEEVD*TPPTSI*CA*LEQLTTT*PHSSDTDYRSCLVAS

SCTSNEKE*TFLSVKNNFGH*TFSGFISISEKFHCQ*SIL*RLNTCIYHHPLTFHFCL*S

K*K

>**comp87374_c0_seq2_6** len=4270 path=[1:0-132 9647:133-138 181:139-3245 3288:3246-3855 3898:3856-3871 3914:3872-4269]

GI*FHIL*I*F*TEY*KVD*YYVF*YRVVTL*Q*FGYFIIFFFRKY*SERCDTLCH*QPK

VGPAIRHFEAVLIISVCRS*NLTLPKSVKMGTEKAKYVEKAKIAEQAERYDDMAAAMKQC

VECCTGKTSDILTNEERNLLSVAYKNVVGARRSAWRVISSIEQKGDMAGGEGAERKQGMA

RTYRTKVEEELKKICEEVLELLDKHLIPQTKEDSSDVKAGNAKDNKNDSRVFYMKMKGDY

YRYLAEVRSGEDRAVIVDKSETAYKEANETAAAKMEPTHPIRLGLALNFSVFYYEIANNP

EKACQLAKSAFDEAIAMLDTLSEESYKDSTLIMQLLRDNLTLWTSDAQGDDGEMGEQHDQ

Q*DRSSLALLAVCSCCCQWHEGGKVAGRGR*W*LQRTFSDGH*DKTCRQIDSCWFAISGT

AGSIAI*D*FVDHCVGHFYTADSQAQCHLFPCTLITKNMSWFN*DKLEVK*FTVPRLCET

ENGNESCI*CGIFFYYLCSATSHVSILDVNPSSSVITDPTVFTQPCIHTYIHTYLQ*LVQ

HCS*QVEHGQIALMMMMVMMKIAQKLFTNVCNLYYRV*TGQLVHFLLIFLCVARAVDTMV

T*VLDWN*QKKCFYMLLCMFVILY*F*SIVVIHYWRKYIVVSPVISELLVVLMHRLSFQV

CKLLLLIYHY*YYCGT*IIINWKSDKHVLSIFEHRISLEIR*KFKILSKQKHALPKVHY*

PYIT*IYIFIVCMYVLLTRMMSVIMQTAATFFVNIIGRIYLFHEWLGIMIYQSVKLFTNC

ALLMKSFCFDLSEMSQKCSDQLNFLYFSLSRFILMPEKAG*AVAL*ILIVW*MCLLHVL*

HLCYFVPSNL*LLAYKAICVRIHTVCPLLVRSDKTTVHQFSVTAVMKTLNFICCTDFPTQ

ISKDQEF*IPHSSL*PDP***CPRNQTLMLLCALHLLLCPSHEMNVCLCCHPNVGPKFS*

CLSLSSAGELPSWVQPKILS*HHS*NGFAWIVIILCSQCVIAVAHC*LYVDNCCQIGSFV

ITGKRLADLFKTIN*DAVCWSREVLRGVLNNADTGYTVWIKHWHVPNIFFFFCEY*CFIY

PLLDKDA*YCFTSP*NFICLVFFLAKRKL**LPYISVVHGCQLSLSNIHIK*TE*ILGS*

DMTLCPSSPIVDPGVTVVIT*RCSSDNLYATVQLSTVSNHCLNMSTATGNIGVVKCVDFI

CISLFIHNWNSKQNCDTVCECLNNLALPEN*DLYFVAFEMWI*VNM*RL**LYAIDG*IV

RLQS*LT*AICMKSA*FNTF*LIHLNEGILISNNRFC*TV*VDIVFLIRHCNMTG*HFYT

YIFISRFLAGIRNEHTWKVMAVGKFS*CSI*IHR*MILIGGKSFILWIIL*ISFQEIHTQ

FLSIVISVS*KNEALYNKMSIIQMVKSLLMYGYCPF*MVD*KT

>**comp87569_c0_seq1_5** len=979 path=[957:0-95 1053:96-131 1089:132-155 2553:156-214 1172:215-222 1180:223-291 1249:292-311 1269:312-978]

RVAELKQTTLYNGREKMAAKSTGSFIKKEILSHDHDHAGPPEGSRVVVVAVDGSHHSEFA

LDFYLSNLKKANDYVVLLTSPEMEDILKATWTEGNYNIDTDHIAHHLAKEKEKVEEKLHF

FKEKMLTREMRGKVKAVSSHNPGDAICKGAEEEDADFIVIGCRGLGAIRRTFLGSVSDYV

LHHSHIPVVICRHKHSHEHHANIGHKWNL*WHHRIHDHQRPGYM*IVKLTVKMYNLRIRI

VERIIRI*H*SINLKFQDTVLE*LMLYRPIHRADWPLDVLPRTVLQES*TLHLTESVRLF

PEQRKMLLGDVFVMQNMYY*TFLITCX

>**comp87737_c0_seq2_3** len=1037 path=[5321:0-34 1650:35-46 6585:47-62 6601:63-70 6609:71-83 5602:84-90 3767:91-1016 471:1017-1017 472:1018-1030 123:1031-1032 6854:1033-1034 6926:1035-1036]

KTKY*YSFKCTDNCIVFEKVLMHRGIPTIQ*LTVFWLGVIRLQSMTAG*KEDDSC*VMMP

S*EH*GINNCGNNHFSHCYNLNESTPQTLHDANTCSWPAGTRSRPLCVFVRDSSSTTSGM

YQECSS*APLQTRGSGCMQ*YKHICHTHITQLKQLKLSL*AHGLYNTSLEVFLRIFNSSL

GHSIHNCPSTCFLPSRMGNTVWKLVLALLL*SSCV*GGRD*VGKHGKDGEGEGSGLVHVY

HELKEEIM*IAINGLQQHLKCIDCSLAAHKKSLTH*GQHCTELLG*PGGLLNRTEVRLQN

TVQNGCTNLAERFTGLLYQPK*ILYRTVVQAKQNTVQDSCTSPGE

>**comp89292_c0_seq1_4** len=325 path=[1747:0-324]

PSRMYYKFTSDYKPTPPTAHAFIEGFKVGIVSGTTTIKLKRGGRVTSTKTFNCGGASRTS

PDTDLYTCEGNPTGSSVLPLPFQHKDVIEFSYATSNGGNVKVRNKESN

>**comp89520_c0_seq1_4** len=1098 path=[1:0-97 99:98-99 101:100-141 143:142-154 156:155-551 553:552-920 922:921-921 2187:922-945 4135:946-1010 1012:1011-1097]

LI*PIRSDLHPSVKMSPTFLPALMMVVSQSGLLYAHVALLNTNPWITKFSICVPRKEYAT

CVVQPDPTAPYKVTGTVDFSQIDYCGYYGDLLIRVTLKGFPRSDPVKDHGFHVHQYGDLS

DGCTSTGGHYNPFNMTHGAPSDEKRHVGDFGNMPVQAGQVKQFRRDWMATIFGRYSIIGR

AIVVHAGTDDLGRGGDPSSKENGNAGSRVACCVIGRGVK*SAADNQ*LVYSE*TVDIS*I

LNTNTVPEQYIHGAI*MVEDGTIKISRSRFTLYVCIMFFI*FFLSVPLLNTL*EGCLSST

YST*SLPYAVVPGG*NEP*RHRCITSFVFTVALIRAKQYHGTLTLVCIDSITVYNRLICI

*NKSVC

>**comp89562_c0_seq3_6** len=1172 path=[1290:0-366 1657:367-509 1800:510-534 3572:535-1075 2848:1076-1171]

*GVRCEGGDDYILVVALLVCSTAVIKIEHSVRKE*TMELKGAFVLLALSCLVVIGSSKDT

PTFEELVDQSERRLVKRDILDDIRNAFKSAGDTFTNVIDKIKGFSQDAYEKFVKSLPSLD

KEDMKKALDKIRGYYDSLQDKTSATAQRMKMTMDELTKDIAANGSNRVYQSSLVASILSV

ILAVFMSL*GGRVI*GAGS*AELRPLLQGSGRYQQL*TQVYKRPKR*TSQTYVTLGFLNT

VTNMHEVL*KRP*NHSSERLPIINNCLSLLSILVIHTFPKM*YNPYDNQDCFVRNT*FKS

YDYQEGSTPTWSYGMYIL*I*LNRYTLY*Q*RVISKPPFLKVMVSLHLRMSTSFP*TEES

CFGFVERVPGK*TFPI*IVLYFVTSGNKL*X

>**comp90174_c0_seq4_3** len=3377 path=[1:0-592 594:593-672 674:673-712 714:713-2863 2865:2864-3094 3096:3095-3095 3097:3096-3376]

QNKMEVCRLLVLVLLFLQQICMTVSRPGYSSDFTIDAPTCGYESCNPVKDGMINVHIVPH

THDDVGWLKTVDQYFYGDHNDIQTAGVQYILDSVIPELQKDPTRRFIYVEIAFFARWWRE

QDDSMRHVVKGLVNEGRLEFILGGWCMNDEASTHYNAIIDQHALGLEFLRTNFGECGRPR

IAWQVDPFGHSREQASLFAQMGYDGLYFGRLDYADKNRRLNHTTMEMIWRGSPKNIGGRA

DLFTGALPNMYQPPPGFCFDALCTDHPIMDDERLHDYNVPDRVAAFLKAVHDQAKHYQTD

HIIMTMGSDFQYSNAHTWFKNLDKLLRYVNEQQTANNSNVNVLYSTPSCYTYQLNKADKT

WTTKEDDFFPYGDSPHAYWTGYFSSRAALKNYVRRTNNFLQVVKQMDALAQLEDTDNSTY

NIEILKEAVGVAQHHDAVSGTEKQAVAYDYAERLANGVNECQKVVNDAYGKLLPLGTAKP

PGQGFCTLLNISVCTATESQKEFQVLVYNPIGRSVTYNVRLPVVGSAYSVTGPLGEAIVS

EVYPISEDTFRIPERKGALAANELVFLVTVPALGFNTYFVSLNSGAESQVPPRKSFTVGA

DTTIGNQYISLTFDGTSGQLKSMKNLKKNLDISLGQSFMFYPGYAGNNSGDVNQASGAYI

FRPNATSATNIGSGAQLSSSNYIREGRLVQEVYQQFAPWATQVIRVYENEQYAEFQWTIG

PISIDDKIGKEVITKYTTNLATQGRFFTDANGREVLERQRNHRDTWDLNVTEPVSQNYYP

VNSRIYLQDTSRNVQFTVLNDRSEGGSSLSDGSLELMLHRRLLKDDGRGVGEPLNETGAD

GKGSIVRGYNYVFLDTIADSARLHRDLGERLFMAPSMSFTNVQMKYADWSKNFRTNWSGL

KRELPANVHLLTLEQYAGSGPAPSTMQPYLLRLEHFYEKEEDAALSAPVTVSLKDLFVPF

DIEAVTELTLGSNLPLSQLNRLKWKTADSGHTDTTLRFKPVSLLAAAPLDITLNPMEIRT

FQVKLTQH*PQYHLLVTDREVTPDPYSYNIMLMNVSL*FELV*SSNILSSVWASQVDVC*

LNWCEHSLY*SSICAQSLPD*RSNTWRVCVCLLVQACMCEYRLVC

>**comp90611_c0_seq1_2** len=3646 path=[8397:0-3645]

TVDMCTYQLTVGVEDFVYKRMLFNYTWGTPDHFYIKGVFRIDYTINKLPAERKLEVSATA

SVCLKESDCLFTQQLLNKAKVPQPLCDWKGMLQARNFSLSSWSVGKGLSSTVSSLTDSLV

SQLLDDLGVSQYFLSSSCDQAASPYFPFNINNWNKACSSLTVPALPNSVKCHLAASCSAL

DCCVNIPLIKRTIQAKFDVDVCALEITMSIEKLSYKLSLLQYTWGVAGDFILGSMTRISY

KIDHVAASKKLVVDLSIKVCLEDNSCILEVPVFSQSEIVYSPCNPSLPVPFKGVSFDFWK

SEPCTQPAAPSGCSVNLPASLANVCQLTDNCMGVTCCKDIDLKYLGIYSISAGIMADHCN

DQLRYHIENKQYTKNLAAITYDKNFTEPVGNAIEISYMVSKTPSSYQLSLNIKLCALNGY

DLTDTCYYYTLMDKVTFPIPSCAPLSRRRRDVITITDTRDPRELLNKALDRNATNEELYA

LFDELQKLEEADTSMNLQYQDTDGAATNTKTALNEMGPANPGTILYSGEVANGDVTVNME

GGEKIMQILGDAKDIAGRVGQAFTVGKGLTGTGVKLLGAKLANMTIGQVIAMFDTKNIDP

EQALQLTRKLRDLALALYSEILNAIINGEAGQAFKSFDLTLQGSFGFPRISVIFFEYEQF

FLVGGLVPMTFGWGAGASYGMDIVVGARILGMTAFGTVIPYANAHVYGELGIGAILYGKL

RLDGYIMTVAFPSTAEIGFSKFPLDLALNMDLELTPIELVLRALVTLEIKIPFIGRIKKT

LFSKKLWRYATPTIRKRMIEVGKKEKDESPPQFLQYEDRTGNSRSKRAATTTQCSVRQLP

ERDYTQPAIEIAVQAQDDRSQVELFLDAGTKPGLSDVLRKAGLGGPSTIITQRFSKHGVP

IYFTVFGENSAGERSEVTCNILTYDVTLPGGRLTADFKSTSSPSELRASVVVYEDSDIVV

SNVGVGLGRGIFGDEIIAFNAIDLKTRNNRAYDASSDPVGHVALGHFTGLKEGRLTGPVI

ATFTQMNHAGKCAKECMKYPNTKCLSFNYDYGVSGECELLEAIEGHDYKISSSGRYTHYE

RLAVGLAYEFVYKDLSLRHQAIYYFNLHIINSLMYENILHSKGIVVDLTPPEPGPVANVT

VDMLEVSTCRNIVPDDRPDWEERCRGIDGDVKNHRTLHDGPGSMTLFNGDEPLTDLLYTR

ANRYVAANWDGIMDK

>**comp90707_c0_seq1_2** len=3314 path=[3292:0-2269 5562:2270-2411 5704:2412-2719 6012:2720-3313]

SHLEKSRWLSKENVS*CPCKQCRLVAMDEEYDVIVLGTGLKECILSGMLSVSGKKVLHMD

RNKYYGGESASMTPLEDFYKYKGRTYPGDNEKFGRGRDWNVDLIPKLLMANGQLVKLLLH

TKVTRYLEFKSIEGSYVYKQGKIYKVPSNEKEALASSLMGLFEKRRFKNFLTFVSDFKED

DPKTYKEVNVKGTALQLYEKHGLDKGTIDFAGHALALYRDDSYLNGPCVDLIKRCQLYSD

SLAKYGKTPYLYPLYGLGELPQGFARLSAIYGGTYMLDKEDATIVYEDGKVVGVSSAGET

ARCKMVVCDPSYAKEKCKKVGEVVRAICILNHPIPNTADALSSQIIIPQNQVGRKSDIYV

CCVSYTHQVAAKGFFLAIVSTTVETDNPEVELKPGLDLLGPIVEKFVDTSDLLAPTDDGT

DSQVFITKSYDATTHFETTCDDVLDRFEKITGEKFDFSNISQGDDQQQ*N*TKQCDFHCN

YVAHWTVLIPRHSRHRTTLPKLRRRSTILEC*ETVVPW*ICFVKSG*SPFVCHVDQTTV*

SCNKLIELNVSHGMLLLVQLSFFMFDHVL*APLVLCPNCWSCQGVLGVVRFCLIFLSSIM

YKPFLKTFYPV*VTRSFSVCQMNCG*VGTDVIAALIILCMLSIDNFGVVP*HESLLC*W*

FDNESVQLYFILSIDTVLVVIILAHQEVVSSFLKFHKVYFILLNSTMMYMYLTAVMM*IN

S*YMK*LQFRQLI**WHNAKLKFEDVDKSFLKHLELLGTRSAWLSFIQMTAPHLT*NLFI

PNWVILMWSSDSCT*EDHVESLLRVTL*RYWAGVPVFMYISDSVASL*IGS*SIYNR*LD

ILLYSICSG*RFWKLYNLSAVQCTNIQCILQSEVDKSK*ARTTTSITRQKWSVVNQCQRQ

YFMQWNLF*KQLSKLLIVTHMYLSDLGFGRRMFCESVHGNFKLIFYRDLLQTGKY*LCNI

FKSR*LLCLLIP*FFWPQSKICS*SLFTIPG*MDEIHCHRSILDMVHLFLKGFTPAGPCN

SLRTCVC*YVLYHLSLLFIRPDMYSLCKQIKLV*FCSVVDKS*YFTQKATNVL*ENFSFH

*HPDEFLAHV*VKSSH*LLSIKM*X

>**comp90883_c0_seq1_3** len=1777 path=[1756:0-886 2643:887-888 2645:889-960 2717:961-1284 3041:1285-1288 3045:1289-1443 3201:1444-1543 4477:1544-1544 3302:1545-1554 3312:1555-1621 4524:1622-1626 3589:1627-1648 3406:1649-1649 3407:1650-1776]

HFAGSCRSSESARITSSKMASQRKFVVGGNWKMNGNKSSIDGIISFLNAGPLDANTDVIV

APPAAYLDYVRSKVNANVAVAAQNCYKVDKGAFTGEISPSMIKDVGATWVILGHSERRHV

FGESDELIGEKVKFALSEGLNVIACIGEKLDEREAGQTNDVVFRQTKAIADNVSDWSRVV

IAYEPVWAIGTGKTASPEQAQEVHQSLRKWVSDNVSPEVAQNVRIQYGGSVNAGNCKELA

AKPDVDGFLVGGASLKPDFVTIVNARKD*CNPRSIVVPSTSNTKSW*HVSTHSEIQPAQF

RAETLKHFFGSIECFVIVFSSFICNVTTDPR*VLCIQHEILCLSQMTDW*STVISVG*QQ

LSVLAINSCQCWLSTVANGNLQL*TSKVTIVM*MI*QAIESQTLFI*VNTD*HFRINDLA

CCHVCTAMFGICKTILVVSL*YHGCIKQCGLDRIRVQHLSLVSTNILC*DFVLIESA*LH

RFFNRFLYPIFLFLYIKSSVDNIRTIPY*HSPSRRSVCAMFLSLEDKHVL*SKTIMTVTH

LAL*QHLIQEAVCLLHGLTIYLQQCYVLTILK*RYSQKNKTDYT*ALLTEYX

>**comp90993_c0_seq1_2** len=1150 path=[4879:0-30 49:31-625 644:626-631 650:632-845 864:846-870 889:871-911 5272:912-917 5278:918-921 940:922-1149]

DHITGVSATTPKMMSSGNLVLLLCVCLCVQLAAGQVGCPSGCSCTTSNNAMNCRHLSIFP

TYVPSYITTAYFYYSNFREIPSGRFHGRPNLKSISFTYGSLGTIRSCAFSDIQASVSFSR

VNITNIRGGAFSNLDKNINRQITFSYSNITTIDSYAFHNLTGMNSVTFSYGKIFTIQPYA

FKNIIYSRSLRIQYTNITNLVSYAFDLPHYSFQSSQISRGSVMNIGCNTLEELTSVQYLT

TPCNCDNVELYKRRTRFSGRQHCFAPNQIQDLTPGSINRCQRPDVSVPSACPGSFNNVDT

GVTPTVRPPIPTSGNCTREQIRLQLANQNYREALACFRFNFDMSDNNLSFKFDMILN*SR

A*SLACFINVSC*YEETINIKLR

>**comp91382_c0_seq3_6** len=2048 path=[2094:0-775 2870:776-1120 3215:1121-1135 3230:1136-1214 3309:1215-1457 3552:1458-1473 3568:1474-1511 3606:1512-1903 5632:1904-2047]

LGSHATLTEADGLVSLYILSQLVLYNYITFENCLVYLTDIS*LFVAFRPSRPLYMGVKGV

VFDVSKGKDFYGKGAAYNALVGRDCSKAVAKMSLEEEDLTHDISDLSEEHLKALDSVFEG

TYMAKYPVVGYMDFLKEQFPDKFVKAAAKEDL*SLMTTL*DGHTSNYFLTITWVISRTIK

S*TR*TNYLNN*TIT*MFYMISCYD*LPFLTSVKYV*FHRHQNAFTEITVYCCIKSRVIS

VKFKL*KSLLRIINKIKRICTYAQLLKCANCMLVILFLFTKFPDLKTKTFTFKRGNMSTI

NLCQL*RSY*M*SLVMFSLSVYRKKHGCYFFIVSAETI*KLVLIIRYLYVLWNFIMNKTY

MFFIQTINKQLSQANIY*HNLEMFSFAV*AVVMKTFIYFLLSMSEGDPFYGKCY*LYNGC

DMLHIFRLIFSLLPENNNNLPKVLYE*CDCCYYRLAVTH*GYIKQRCVYSKKTQLIRLQG

LSWSVHEYFIAQTLFMFSSLEIKLNCDIVSTANGILSLHFPESQDILMTFGLLHLKVPNM

NLKTVVLVIHTYPLMW*RFQV*FGHQCPCLS*HTSDTVWLLL*FC*PQFAQHYCDLKYRL

GSGKLSCVFIRDKKGDF*KYRNI*SQA*TISLIL*YHVTITKIVMDSCFLQC*C*VKINL

LKSKLMHFKLHVRNWKVRLFMDX

>**comp91506_c0_seq5_3** len=1142 path=[2661:0-41 2702:42-65 130:66-93 7394:94-100 578:101-198 676:199-392 870:393-770 1248:771-1141]

LVPPPPVTALLLVLPLLQVQVTPQLPVQVLPQLVFLQQQVLQLPLLGM*TLLVLPHLP*F

LPEQLVQLVSQVPELLLLPEHFPLSAQFVFLMVQLVLPFVPLLILQASLQQRVPLP*LLP

ELVLLVPLWQVSLLELRVLKFVQGLLLLVQQAQTLLLPPLLRVLLLVLEKQVP*HFQMFE

QSLMMQQQHRR*LHVHLKE*TDPDLQSVQVLAQLL*PLQHRI*GLSGPQ*IPQKQSG*GH

FLACPQQQRPLRQQLVPYAVSHRRRQVFAWIGVRPFL*FLDR*NAQMKTAVLRH*ALYIA

GRVEVGYNARSLHSTFAIVLV*SVQTDTCMCRKHRTLE*QLARND*SVYGSTLRNRACSC

DKNTLNDPVLKTIIS*LYNK

>**comp91733_c0_seq12_2** len=3750 path=[3874:0-17 3892:18-92 3967:93-279 4154:280-1503 13339:1504-1833 8048:1834-2683 8898:2684-3524 12168:3525-3526 9741:3527-3705 7869:3706-3720 7840:3721-3737 13741:3738-3742 7862:3743-3748 7868:3749-3749]

FKMADGKEGSMKAVQRFERRGALRQKNVHEVKDHKFVARFFKQPTFCSHCKDFIWGFGKQ

GFQCQICSFVVHKRCHEFVSFGCPGADHGPDSDATNLHKFKVHTYGSPTFCDHCGSLLYG

LLHQGLKCDACDMNVHKRCEMNVPKLCGIDHTERRGRINLRITHLTGKLKIEVLEARNLP

PMDPNGLADPYVKVKLIPDESNKSKKKTKTVKSTLNPEWNESFTFDLGSDDAHKRISMEV

WDWDRTSRNDFMGSLSFGISEILKNPADGWYKLLSQEEGEFYGIPVSDEVEPTIEELKQL

QVERQESNEKKMVPLDKAQNVSRQDIVRASDFNFLTVLGKGSFGKVVLAERKGTDELYAI

KILKKDVIIQDDDVECTMIEKRVLALPTKPPFLVLLHSCFQTMDRLYFVMEYVNGGDLMY

RIQQEGKFKEPVAAFYAAEIAIGLFYLHSQGIIYRDLKLDNVMLDAEGHIKIADFGMCKE

GILNNKTTRTFCGTPDYIAPEIVLYQPYGKSVDWWAFGVLLYEMLAGQPPFDGEDEEELF

TSITDHNVSYPKSMSREAVSICKGLLTKNPQKRLGCGPTGERDIKDHAFFRRINWEKIEG

MEIQPPYKPKINDPKKAENFDKLFTRIPVKLTPIDSLIIMNIDDDAFRNFSYVNPYFNLQ

T*AVVCTVWSIVCKTHP*LTQL*SEAFNISLRNLHQFIST*SISFLTWD*LENCAYVHSF

CFVFGVEEETFCIVCFFKIIVTFTKK*TFSDLPNMSITC*VSLAG*GSTC*EKCGSHIFI

PFFIHQVFNCTRCLFSLGMF*KLFPSVLTVLHSNECFS*FYISLTSVIQFGVEHNITQLR

SLQSLSHSCQS*DMNLKILVSIC*GLYLLLKIAHTEQYIYKGDFFVKNPIPL*NVFSKYV

F*NG*Q*C*NTVNK*NTMP*KII*LSSINCDRLCLIFDLKARFLL*NMLLSLS*CLISFA

CICIP*YLCFT*NRICQS*KSLYQSHVSLSSVASLLTSRTSRSRLASSVQCSLLCFFSFH

LLVEKVAICSFCLFAPLICFHISFSTLYVSFSSL*GFHGTVSSFILLNGKKWKGVWKLFL

FHFSDIIHKVAIF*ITFCSLPLINFSNLLYFELHI*IF*YIVFINPVILLSIGAVGNVVF

IQYIFTECIMVLLKYIPLVR*SRDC*QRPPSD*LIHQLTNCMVCHTKHLFNNTDVLYSTS

HYLC*CQTMLIDAGLTVLSYQKLNSDIIFSLFLL*LVS*KWDVKSSLYCX

>**comp92274_c0_seq1_1** len=1763 path=[1:0-14 16:15-23 25:24-783 785:784-785 787:786-934 936:935-968 970:969-1509 1511:1510-1602 1604:1603-1682 4386:1683-1762]

ARKGQAPSTYQYFLKMAGIVRKLVQATAAPKAIGPYSQAVIADKTMYISGQIGFVPSTME

IVTGGAAAETDQALKNMGAILEEGGSSYNNVVKTTVLLKDINDYASVNEVYAKYFSNNKP

ARAAFQVAALPRGALVEIEAIAIVGNIVDSQ*LDASARL*VPSRPASLPIVSTHKHLVTC

HLMAQE*SNEYDFLKYGIIM*L*DRVLVNPSHLFIIVKDLLSCAYILVKGVDTMIWSTLK

YFEAAVR*LDYNRDRQHTQMHVELELPSQCAIILKRFALHVCSLRQTIT*VSS*YLGVSG

STMCI*PAPHW*F*QLLSNFSVTYQCDNYRTKQG*TELGQMLEYVRRLMVLKWLFVLGQK

LSTDQVFTAIYDFYIQDFIQIVSDCIRLFQIAFEERKL*FFLKGKLPEPKYIMAV*YAAA

YCGEQWNKQK*ISTCMVE*QSKTFTTW*FLCM*NIFIQSEVRHITISLFCLLT*IHVSFE

YTLLNTDYASISGVSC*PSSVVLRGKLCNQFVVE*FWCFVVIMELSAGISITYES*RSF*

GVMLESLSQ*SVLAACVYLVVVEEVFKELC*RVYLSNQCWQLVFTWWX

>**comp92385_c0_seq9_2** len=1818 path=[1888:0-182 2079:183-1063 2960:1064-1153 14336:1154-1154 14337:1155-1156 3053:1157-1327 3224:1328-1817]

FDDKLCGMLVFINVPVESVRSAHDYNM*LSHDQYNRESYS*ACSAVVYCLLFGDSRNDHS

SVLFDKIRMLTSLFVLTVCFHQTTHGHGAFRKMFPEKYPEMLRNGVDPGEPLFLTPYLER

GDIQKAKEMSLTTSPIITKGYSGFLTVNKTINSNMFFWFFPAQTDPANAPVLLWLQGGPG

GSSLFGLFIEHGPILVDAHGNLQRRNITWNSKYSMLYVDNPVGTGFSFTGEDSGYATNEE

DVGRDLYSCLTQFFQIFKEYQKNDFYITGESYAGKYVPAISYKIHMENPTAKVKINFKGM

AIGDGLCDPITMFPKYADFMFNIGVLDESERDYFQSQSDLAVKAMQQQRFGDAFKIMGKV

LNNGQDAYFYNVTGLDDLYNFILSEEPASFNYYGPYLARPDVRRAIHVGNLTYNDGNAVE

KHLMNDVMQSVKPWIATLMDNYKVLIYNGQLDIIIAVPLTEAWLQTVPWKGLEEYKKAPK

LIWKVNPNDTEVAGYVRQVGNFYQVIVRGAGHILPYDQPVRGFDMIQRFIEGR*MNTAKL

LRSSRC*CNSTFVF*WFNYAYYHC**YIFKVFLKLYIIICVHTFRACIFWEIYVHMILFV

YFDDMX

>**comp92517_c0_seq1_1** len=9741 path=[9719:0-600 10320:601-2050 11770:2051-2057 11777:2058-4041 13761:4042-9740]

VLSGKGQNRWAGLRVERKGYPSINVKKEAAVLRIQSDRLVPVSGFNVRVKISC*KMVSLY

GALVMTVTCLTLVASQGLSRQSCDPSKSQASIQSGADAIIGGVFSMHDEGTNGFGCGKIT

SDMQAYEAIRWALDLINKKDQKLNGENLTNTYVPGIKLGMNIRDYCGWSEGALSAAQNFY

PQFSSNSRACNQTTSGITLGIVGASDSDASNEVSNFAQNFNIPVVSFMATAPELSSAQLY

PTFMRTIPPDGPLMDVLIEAIKKFNWKYISIVYTDNSHGRQSYEAIRPRLVTAGICLTAA

ISTSPSDTQDVTIDAVLDRLLLTKTVGVLYFGTPSVAYALLERSNTAAYANMGNLQWVFT

DSISLTKQFGTNKYPRGIISIVPTSRYIVEFEDHWVRINENNPSAENPWFQEWYSEQYEC

SFPTINNAQFPTPCSSKIQTERQKRESFIQDRFVEPAVHAVYTYAVALRNAQKAMCVGAE

ANTSCSALRSMTSKTFFENYLMKVNFTYGKEERVPSLASDQYAPYFSAAKVRYNENGDIV

NPAYDVWNFNDAPIGGDTPGFKFRRVGSWINGILSMNMDQVKMYDLTRDNAVTPLPPSPC

PQAGCTPCLGMPSPTKYLYIDGDIILNGIFSVHNPGEGQYSCGAMDLNARSGVQYTEAMV

YALREVPRRFKAVNPQFLRDVSLGGLGIDDCKSGTLSRNFISDVQRGTLVVKDSAGNTLD

PRNVDAYVAAHTSGLTIPIADLMNDVKRPVLGYSSASSVLSDRRKYPYFLRVHPGIDEEM

AAIINILKKQGWNFVQTVYVEGDAFSLNGNEVFKKMAAAHRICVVASHSYGSDIFTRLRT

RPMVKPVLVIAHESQYRGLLENMISSNATGEFVILANSPFGRKQKLVRGLEEAADGVISL

KWRVPDLTFFSTYLSSLRVDSNQQNPWFTEWYENLFDCYIGPQNPNGKAALCTDRNTPIT

QAPTFELTEESIFVINSVYAVATGLHSTLTEYCGPNYSGVCGNFTSSGTRGNRLLENIRA

ARFSLQGSNPPTDFAFIGYEGNVMIDVYNYRRNIGYMLMGEFNPETYGYTESISALLNGG

MVPSSAMSMCPGTCIECLYIYQEMKYQYIDGDIIIPAIFDIHMGGLTRFTCGKMRVRNGF

QYTEAFAYALQLINSGSMFNNVRLGGLLLDSCSSPSRSKALVNNIYSGQLMLQDSNMMNL

RTQDFLSWMSYDSKSTIEVSDILHRIGVPIVSPAATAPLLNTKERFSTFFRTVKSDMHVA

RAMAKLTKQLGFNYVITLNAPDFSSRESRDEFRKLANDEGVCLLASYEFDTDGSMELILR

SINESTTQVVVVFTEPDSYISELLRAKQKNYPTNHIVFIANRRWTLPSDITRLQLTINQT

VLNSITFNFNSPPVPAFTTYLGSKRPTMTNPNPWFREYYEERYQCNLPGTFRFNVNCGQN

YGSVTQSDFVQDLRVLSTMNAVYAIAEGVKRTLEEKCGSQPGVCRNFTNSADTYEKIMAY

MDAMNFTDVVGDFFRFINREADRGYTIQQFLEDGSQVQVGSYNGTSLSLVDERNLQLKFR

SVKSACDSTCLACQRSANNFDDFTYINGVFNLVGLFDVHKKGATPFTCGDINTKHGFELL

EAFNYAVEYVNKKQGIFEGKLTGIGIGAIGLDVCQSPTRAANLVANIHSGNINLKKDGET

IKPKNFDVYIGTFETKASIRVADVLNSLSIPQISYGATSLELRDSRSHRYFLRTVPADDK

QARAIISYLKRFKIDNVQVISTFDSVGEKGKDEFLRLAYLNRICVSQNIVIGRNGTVFRS

EASMTLEKIAGNINAKVVVLFVDDPRAILLAAEDDDKIRGNYFFIGTDKWGMDIDLLDGL

DRLIQDRKAVTFDVETADLPGFDQYLESKIPSNSKDNPWFDEFFQEIHDCYLNNPTSEFS

KPCTASNRGIPRSNGYIQDPYVLYVVNAVFSAALGIHYAVAEVCGADYGFACNLVRNSGE

RRQRIYRNIKQNARFEDATRQPFYFTNGGESDRGYHIYEPVRNPVGPGYVYKNVGSYNDT

HYLKLDIRYEITERSVCQPKGVCQCVFPDYQPSRYMLRDNQNDLNLVYVADIHSNDPKNL

FGCGAINTGFQFQNLMAFFYAIESVNNNTNRRFPTSLQLGGLALDTCDQQYRLGQDLYSL

MSGEGLCGTGQLGQVIPPSTIVAFIADGSSTALPVSSMLSPLNVTTLSQSATTVELSNEQ

FHPYFLRTVPPDNIQAIVMLQIMKQFNWDYASVVYSTIPYGIAAKDTLLQQANAVGSTAC

ISALEGLKVGATLTDAERILDKLSQQVGSRVVILFTTPEHSRLLLQATRSKGLAGRFIWL

ASDYWANSQFIVNGYEAEASGAVTIQIRSEEVQAFKTFMKSLTLNNRHGIPDDWFEELYQ

TLHKCRLLSATLKKDYSSICTGQERITDDMIPSDPFILHTIISVYITAYGLNNLEECRNL

PQLSISACLALQEDRRRQLIYNSILNSQLTVPARELSGTSFSFKFTEGRYGDVGYDVLNY

YRDAGVKRYNYNKIGSYQGVLTLDKSKYTGSGILDTSGVVPLSQCPAGNPCSCLNQDGVG

THIYARQASTSSTSYTSAIYILKEDGNYYNSLTDQLVEVSKVPTVVGRFTDIWAIIVATL

AAIGCFIAACMFIYLLVVYPNRGGTSILGYTLCFGIILLYALVFAFIAHANPEICGLRRF

SLGFVYALCYGSLFVKLLDCWRTQDKEEMDNVKYNKLGRPCGLFLVVVLLVLVQVIINAE

WLILVPPETVRVLYNNQLWPRCTPNDFYDESLVLSLVYIMLLIALSVIFGFATFKKSKNH

YESRWILGIAVLSIPCWVIWCLVASIGVYKVRDAAVAVGLLINATFMLLLGPLRKLYLLN

KYQALIEEEEKEYLASHRSEYASYGRQYDNVGRFHEGSIHGSSHGSTYPRQKYAASLNGT

QRSTGSAYNPYADPNHREEREIQQ*INWCLRDTSTVRLLGAS*CETDI*QTVMGRIFGIL

INK*NALYI*T*QSCLLN*VLNSK*T*H*ILLLLGPSYGRSCRPQCVTGNTDMTFLQPCV

RDDRLTLSFHLKIFKMYDSVSVVRVKLVMRNSLLI*GVE*VLKFVKNVLLRPDKFNFLFR

KVYSIVLKQNGNMYNSQNPSRLDLQFPLFCFC*LT*VKTFSSDKKMSWSGLLNNIYI*IT

VHLYLCHPQGLCTTAYKSYYTLLLIQSSSDVML*FVNIFAKLY*HMSDYSNFVYTLILNC

NKHLYFV

>**comp92600_c0_seq1_6** len=1641 path=[1:0-64 4356:65-74 76:75-100 102:101-118 120:119-164 166:165-170 4408:171-259 261:260-281 283:282-322 4476:323-331 3347:332-347 3363:348-395 397:396-523 525:524-542 544:543-685 687:686-705 707:706-1640]

FDGRLAERYEEARLQAHRKERRFRMYAVLFVALTCLGAHVNAVNNGLARTPPMGWMSWQR

YRCITDCVQQPDNCISENLFKRMADRLVSDGYKDVGYEYVNIDDCWPLKDRDAEGKIVPD

PARFPSGMADLAKYMHSKGLKLGIYGDFGTETCGGYPGSKFFMDVDAETFADWGIDSLKL

DGCYTNPDDYAAGYPLMEFFLNKTGRPILYSCSWPAYVKDPDYKTIADNCNIWRNYGDIQ

DSWDSVKSIIDFYGDDKDNFTTFAGPGNFNDPDMIILGNFGLSYDQQRVQMAMWAVMASP

LLMSNNLEDIRPETKELLLNKNLIAINQDPLGIQGTRKSQVGNIEIWSRPLSPVGKFAIA

ILNLGDGGCAAIVNTTLSYVGLTNQAGYTVTEGFTGAAVGKFSLTDHFVSKVNPSGIQLY

VASPN*VHDYNSIAPTLKYVMMYV*IYRSHDLPMTCFVRTIFTFSVVMCKFCLRLSDLV*

I*GRCRSCILLCSIQLPLI*YNELTNHMVAS*HVIVCTATLFSKCTMIALLLCH*DCMRV

IK*YQPX

>**comp92750_c0_seq4_1** len=2029 path=[4060:0-91 4152:92-814 4875:815-836 4897:837-1812 10530:1813-1814 9406:1815-2028]

SY*LTSVPTL*RPYCEPVLYKTRLFSIDSSRGFQHQSGF*GDL*HSLVLDGG*GYTIKMP

KPVNVRVVTMDAELEFAIQPNTTGKQLFDQVVKTIGLREIWFFGLMYIDNKNCITWLKLN

KKVLTQDVKKEQPLQFKFRAKFFPEDVSEELIQEITQRMFYLQVKEGILNDDIYCPPETA

VLLASYSCQAKYHDYSSDVHKPGFLSNDRLLPQRVYDQHKLSREQWEERITTWYCEHKSM

TPEDAMMEYLKIAQDLEMYGVNYFEIRNKKGTELSLGVDALGLNVYEKDDKLTPKIGFPW

SEIRNISFNDKKFVIKPIDKKAPDFIFYAPRLRICKRILALCMGNHELYMRRRKPDTIEV

QQMKAQARDEKLARQRERDELLREKQQREIEEREKKQLQERLRLLEEEREQMKRDDLEKQ

KVFEEMQLKMSAQEAAIAEERRLQEELERTRMEKEEAERQMQAAESLKEEERLQLEQELL

AAKEALESRQCELQEQVLFVNNLKEDLDKTKADAEAKERELAEQLRVREEERARQLAELE

EQERMRREMEERLSAAVHVHEHEDDNEEHGSSELQVEENYMNLARPEESRQVIADTNKSL

SAKLKVGYRCNVI*TH*GIENITQ*VTVKSLGLVH*STVTMGISGDH*F*FYYHNS*CAE

VYVALQNW*SWGLRAYX

>**comp92770_c0_seq1_5** len=4375 path=[4354:0-39 4395:40-188 4544:189-4374]

CTPSVDYAS*CVLPPQAVAQLSLCVQASSPQVTHTRRDQYTCQLPPIHPLYSFSLGRA*D

LE*ICGVEGARAQLVVTVMDFRDASKVNVNRRCLYFLIFLLLVLPVVVGVLVWYFIPKCD

QNDPSSEKISGDGSTTPSVPTTPGFSETEPWKNLRLPRYIVPLHYDLTLYPDFYDDNGWF

YGNVTVEIRINKDTDYVLIHFNYLNITKTELRENSTGNQIGIKRTFAYAENQFWVIEAAV

TLREGASVKLSLQFDGSLTRAIVGFYKSSYVNSETGQTRNLATSKFEPVDARRAFPCFDE

PNIKAAYTITLIHEPDYTPLSNMPPKESQPTKVPWSPTLVSTRFQRSVMMSTYLVCFIVC

DFNYVEDTTSSGTKIRVYATPDKINQTRYSLQIGKHSMEEYERLFRLPYPLPKQDMIAIP

DFVSGAMEHWGLITYRETNLLYDPSKASSANKQRVAVVVAHEIAHQWFGNIVTMDWWDDL

WLNEGFASFMEYLGVDTYERDWDMMAQFGIEDLQPVMVTDAGVESHPIVVPVYKPTEINA

VFDSISYSKGAAVIAMLQSIMGKDKFFEGIAKYLKKYEWGNAKTDDLWQALGEVPGAPDV

KHIMDTWTRQMGLPYVNVTFQPSSSGGTTVRAVQKRYLSDPKAVYDVDESEFKYKWYISL

SYITSNGSTGTKIMDMTEVETIDVNVDMTDESQWIKFNVNQTGFYRVTYPDDIWRRFATV

LATSNPSNFPISVADRSAFINDALNLARAGLLNYGVALQMTSYLDKETNHIPWESAYTGM

AYITKMFRSGGSFGLWRQYVLAKVKPALDRLGWEDTGSHLDKLMRRNLIELACGHGDVEC

LGNATAKFRNWLDNGVEISPNLRPRVYKYGMRSSGSVADWDKMWQKYQTESVPQERVNFL

YALAQTRTVWLLHRYLDYAKNESIIRQQDFFSVMSYVNLNPVGNNIIWNWARENYDELLG

RFTLYSRSFGRMAPNIIKNFNTEFKLTEVEAFFKQYPEAGAGDRPRKQALETIKSNINWM

KTYQDTIVSWLQKNM*K*LILTGDSGIDLSEIIRQAPRRLMKLVNLFIEDLGEDD*LSIL

HC*K*K*KDFTALRVSSLVKQNNRHIVFTAL**CLCFTIH*ELC*HDSL*SLNV*WHLCH

YFL*HK*ICLAN*KWFAKDLRKDKMYLEISKHFKFPDCNW*VAMSGTLVILSGGSIRK*W

R*MIGCLQNTLFAFQCMRTVFVVVIFEFVNTYT*MERHD*IWHVSMCVLVCVIFYVLDIR

NYHFAS*F*QFQFCH*QIFRYCTDVILPLMSQVCLY*IRSYTYHTH*NTIKQI*YFLMY*

KLNVYVCESA*LNMHFLDCTLSCKIYQFSLQVVTSLRVCGVLVCSRCSQLHT*RNIYRIY

IILSCFLSPVSS*KKMKCVVMPDTSVYSRNIARKYSVLFYIVHHHSSCIVNIQTEFYWKC

IILYIQYPL*QNTNKWPKX

>**comp92777_c0_seq2_2** len=1524 path=[1506:0-76 1583:77-764 3034:765-786 2293:787-912 2419:913-1107 2618:1108-1127 2638:1128-1129 2640:1130-1152 2663:1153-1153 2664:1154-1458 5035:1459-1523]

KTSRPEKMFSRLARQPLIISIVKNAFSTSAKTDAKVAVLGASGGIGQPLSLLLKESPLVS

QLALYDIAHTPGVAADLSHIETKAKVTGYLGPDQLKECVSGAQLVLIPAGVPRKPGMTRD

DLFNTNASIVRDLSDACAQYCPKAIMGIVTNPVNSTVPIVSEVFKKRGVYDPKRIFGVTT

LDVVRANTFIAEAKGLDVSKINVPVIGGHAGATIIPIISQCTPPVSFPEEERDRMIKRIQ

NAGTEVVEAKAGAGSATLSMAYAAAQFGFSVLEALGGVEGKVECAYIYSDETKVKYFATP

ILLGREGVEKNLGMGKLLGYEVDLLKAALPELQESIQKGEDFVLKG*KPTLRKQDNTACL

LSLFWRLN*CLF*DAPYMCPGKNLPKLYYMLAVCINEHFT*TSIQNSLSCMVEISSWFV*

SK*IIRFVILCIIICNGTDTIK*CL*TVSMWRPDLGIFHVQVPYITSQHVL*VWTYLCPF

VSIDSAVSHLVCERLRFVDM*SLLACFV

>**comp92958_c0_seq2_5** len=1533 path=[3447:0-11 3459:12-86 4376:87-105 4395:106-108 3739:109-109 1520:110-133 4444:134-137 4448:138-157 3983:158-171 1573:172-422 3192:423-439 1841:440-796 4609:797-817 4630:818-818 2922:819-910 2300:911-1532]

FTEEWGKSCDCHMSISKRKVLPIIVREDTGR*RYTHLTSRMHQYLCLLLLCGVVHGLNNG

LARTPPMGFLSWERFRCNLDCKNDPENCVSEHLYKSIADVIVAEGYRDLGYEYVNIDDCW

PAKERDAEGRLQGDPDRFPSGMKNLSQYMHSKGLKLGIYEDFGKETCAGYPGSEFFLRTD

ANTFADWEIDLLKFDGCNSDPKDMDVGYSTMSKFLNLTTRPMLFSCEWPGYLRAVGIKPD

YQAIKENCNIWRNYNDIQDSWDSVLDIINYYGKNEGNFAAVAAPGGFNDPDELIVGDFGL

SYYQQKAQFGMWAMLASPLFISADLRNIKPEFKAILQNKRVIAINQDPLGAQATRLYQAG

AIQVWRKPLSVNGTYAVAVLNTDNQGMPTKVTLTPAQIGVPNPNGINITETYDNVHVGAF

KPGQNFTLSINPTGIFLATVVPLS*WEANLLGLL*IH*EVNMIHVSNIGKQME*WVTNGI

LEKECNNKS*LMCSMNPVKQ*QIIFPCPDSX

>**comp92980_c1_seq2_3** len=2514 path=[1:0-833 835:834-850 852:851-1152 1154:1153-1162 6714:1163-1742 1744:1743-1759 1761:1760-1883 1885:1884-1901 1903:1902-2475 2477:2476-2513]

DFVDLLIKLGLYNNMVLIDTGVSPDDKNSSVHIIVIDQAQLGMPSRDYYLKPRNDDHIMV

YQNLATDVAVLFGADRTQAEQQMKDMVDFEITIANLTLAQAERRNMDTLYHRMTVKELTD

SIPGFDWLRYLKGVFGPFNHTITDSEQVVVYTPDYIRNMLQVIKKTSDKTVVNYMMWRIM

MNRVNNLDEKIRDSQRDYNKVIFGTKRMPPKWRMCVDYVNNNMGDAVGRLFIDNYFDREA

KQNAVTMIRYLREAFENLLRKATWMDEQTRKYALNKAGAILEKIGYPDKMLNDTVLNDEY

GDVSFNETTYFENVLTLLKDRVRDNAFYLRLLVDREEWTTTPAIVNAFYSPNRNSITFPA

AILQPPFYSKEYPMSLVFGGIGMVIGHEITHGFDDEGRQYDMVGNFHQWWTNSSLTKFTS

LAKCLVDQYSSFSINGDHVNGKMTLGENMADNGGIKASFEAYRKWVTENGEEPPLPGLEL

SHEQLFFVNFAQGWCSLSTDAALHNQIITDPHSPSKFRVIGTVSNSDYFRDAFKCTPQTK

MNPDKKCGVW*LAMTWSSLIG*KLTQCD*FTSTWTSCVVKP*Y*NRTNTVNVNNSVDN*D

SFVESLY*PVALIWMICFLYIEYKQCINLEYKTRKQRNKKMTICILYIHILLKLSNGTHS

FAATKASYPHY*D*K*NICGLCRHIIGEVYTDLTAVPLFLFISRC*YDLVLLMCRSDIFV

EHVIVICDNIHVRHVSLLLV*LVTVSVFVYICWLLVL*KYLFLENISFV*CFSCCSSVGR

VIISRDKFL*IWKTLL*NQWKMKTAKLISNPSYR*IAYYPLMSF*KNDNSSNSKLGPX

>**comp92993_c0_seq1_6** len=1148 path=[1:0-46 48:47-65 4306:66-84 3010:85-125 101:126-158 2567:159-205 2614:206-404 418:405-527 4470:528-529 4472:530-544 2899:545-584 598:585-631 3720:632-635 4514:636-770 784:771-842 4136:843-850 864:851-991 1005:992-1061 1075:1062-1108 1122:1109-1147]

QELSFTHHPTMGKVIYSIVALVCLVAMTTALPPLPKPACYSQQLSSDQNFDLSFVCNDGR

KYPEQEIEEALYYADDHWRIYLNLTSTCFKDQDTYFLNKYSVPKYYNLKSVCNTTRTMVH

TVYAVDGQVCHVVRPHAQKIYYATCGGECNLGYDGVTGYHCIQRGITYRNLLGYCRFPDP

AIFPETDPIYALDPSVDNFYDPRAAPLTYRMQHNLLQNGAHYQPKGYFTELSVTLPSYCG

CRGYFCPHDRIL*MMTIYKRLVQHEHQPWTSRFLQSFHF*HGMKIF*NQNCFK*IGITDT

ILYTGNYFSNKDSFNLHHECSLGLKLAGMSIVNILRIPCKYFL**IL*TCI*SFFVQCKQ

TSSLIVRACI*CTDVNM*K*KHR

>**comp93615_c0_seq4_5** len=4904 path=[1086:0-376 15526:377-389 15537:390-696 15618:697-697 7984:698-761 8048:762-765 8052:766-968 8255:969-983 8270:984-1007 8340:1008-1783 9116:1784-1790 9123:1791-4903]

SHHPATHTWS*EQLPPALPAAVTVVGAGAPRCNCCGYRRC*RCTAPAGND*HWHHIGTTP

RRCQLSAPVTEFGGEGATASARVAAAKHGHE*RA*TGRQ*QQSSSSSST*SHPSPATTRT

TAAHTATSTI*SSSSRHLRCV*CHPPSSYTIIPATSDSLPASASRCDYI*C*YNTNTQLS

DSGITTGCSE*HQLQCDTSGSRSLSTCTSRESVG*HPGDSVTDPAATPGRPTEPAGATES

ATAIGTEAEAESDVLWSPT*PV*AQQQHRSQVQYTEHSSMWPRTGSVTASLQAGADSDEI

WGH**QLPAILISRSCTSSCD*LM*LR*RSQVYDNLLDCRPRRSWAAVYTI*LFLVFQ**

A*VYI*H*TKCPIQVS*IR*CV**GARRVLNF*TIFTGKQ*KCARICDP*FQCSSQPEGT

LQATSVERRAGTDTRCCQWHQKDPLSCPCHIHSPGFVHATTQPTISGNLRGTLSSHNHAS

NMDTSSSQSGS*SIIPKGRRL*CSITQTG*GICVF**EGNSRFFK*TF*NL*ASVC*SLR

RVKVQESPAENKIVCL*SGRPCIGDPCDFQFDLCDPKCNLSPASQAGATCTE*LRHLP*C

TPG**VCFVHLSITNVLQGCIRTRAWCAGSCSSDTDGGR*YAFCADP**PSDNYYT*GFR

FLSLLPGLTCDISCPQMILTTMILTFSRSQ*SHVGPRHQPLFCQRLRRYIDLVDILFIM*

F*YGILNNCEVFLLS*LCSAMIMKPHFPL*LELVCNG*KCSSELMSSSSPLCLKMCDNV*

KIMKTGWGKTPSTCVLHIY*KKAGQFISNTGATACCMVRLHYITSTF*THSVMTQRHL*Y

HKWGIEIVWVEFNSKLFPCSISYCQFLNFSIILEFRISCGHFVNFNKACTQIPY*TFWAR

GNFYYQYVLLSG*TGYDTVF***NLAGTVLSYVIGTPQNGIDPFFILYSGTTVWAIALCT

T*SIYSIT*FYKTTYASALRRRFEAWKI*QARF**QILVEKTKYIKLSQ*ELQLNPFTPT

*ILLPPVYNHHPSTSSSRIPLLYHKEHHWMLQHSRTYCNCIIIVSMWMLHPPDETDRVDR

LLSSDRVLTSQVFYPAG*HRCV**PACTHYVKYRQADDYLNISAPLRFVTVYLRSLPI*N

YKQAETF*RWRNLIQFPTSPAGAARVYTSLAEHTNMAVLTRILLPYPW*YQGRVYL*HL*

KINHYFFRKSDCDII**V*HL*IGCLPFTGIWKGGMEGD*VKGTTIQCVLNSVNTCTLVY

IASFSASQVGNVALEENRNYSP*NIPHFQSPVNYIRH*DNRCFRQCYNRCFSVMSDV*DT

*ITVISDRQ*HSHEIQHGLIHRLFLSSS*SKQFSCFCMS*TVMIINSQQHHHAASLIRKQ

RKNLTRHSNHACSDSHELNTIP*KIVSILATLTPRTR*LLLDDPSRDDPHNTRCSPCSQP

WRIVGCTTARILY*FVITSCCSSPCPASVFPNRAISRIIVCFLKPWE*TYCYMELE*LQW

GMLRGFIDRVPFKRQYSVTKWKTWDVSLCSRCLFCITIQGWTQQLVCSENLDQLVYSHSY

LIYRNKSRMSCGLVGSLTVSTLLCM*RVTRRNISNWLSHCIHVLDVEAPCVAPCT*HHGP

ISQNDHRAKMIVTPX

>**comp93713_c0_seq4_2** len=664 path=[1686:0-106 1793:107-469 3394:470-563 3488:564-564 3489:565-663]

DVTAMQGLRHFVISTLPRAVTGRYLSTTAALNMPIKEGDKLPEVDLFEGNPHNKVNTKEF

GTGKIVIFAVPGAFTPTCSETHAPGFIKIIPELKQKGVAAVVCIAVNDPFVMDAWGKSLG

ADTKVRMLADTAATFTKKIDMAVDLTEILGGVRSKRFVYHVAWKSLS*M*LPL*MLVDAF

HDTGL*PFSVTMHYYTLL*DAVTTEHYCNTLHYHTCGINTH

>**comp93747_c0_seq2_4** len=2566 path=[1:0-650 7687:651-666 668:667-671 673:672-740 742:741-762 7770:763-779 781:780-801 803:802-805 807:806-1133 7854:1134-1134 1136:1135-1150 1152:1151-1185 1187:1186-1209 1211:1210-2464 2466:2465-2565]

SAAVLGVCAVAILCVTGVAGVGMLYPRDSESREVKNLDGMWNFRADMSPTRDAGLTQSWF

SQPLAKTGPVIPMPVPSSYNDVTQDRALRDFVGWVWYDRSFFVPQDWQNRRVVLRFGSAH

YYSMVWINSQMVVEHNGGHLPFEAEINKYLNFAGSNLLTVAVNNTLTPHTLPPGTIKYET

DTKKYPEGYFVQNLQMDFFNYAGIHRHVRLYTTPTTYIDDVTVTTDINDGKGVVNYQIVP

GGSTQGNSIKVEVEDREGNVVGTSATFSGTVSIANANLWYPYTMMPNSPAYLYTLKMTLT

SAAGTDVYRLPIGIRTVQVTDTQLLINNKPFYCHGAAKHEDSDIRGKGLDYALIAKDFNM

LKWLGVNCFRTSHYPYAEEIMDQADQQGIMVIDESPGVGISTDENFSNESLSHHLEVMGE

LVRRDKNRPSVMIWSVANEPATSKDIAAPYFKSVIGHTKNLDPTRPVTFVCNHHYNDDKA

IPYVDIICFNRYYGWYSDTGHTEVIQLQLGTDMDGWRSKYNKPLIITEYGADTVAGLHRD

PSSVFTEEYQVDFMTEYHKLFDTRIGKYLVGEMVWNFADFMTVQGVTRVVGNKKGMLTRQ

RQPKAAAFLLRKRYHDLMNSTRHH*VTLTAQ*CYKCYIPN**I*NSNITVTEIFNRDTHT

G*FV*KFASQIWLNAFDYCNFCYFMFNILSAMFA*Y*FII*NEETEIKCHYHKISS*YHN

*N*SNKKNSTNYKTVLTKVFVKKSYKRMKSPSMEMLHIQY*YFGFVNT*HISV*C*GIYR

VSVYIYTAIFNSAGAFSNYALVLIYILYFCLVLVCFKTGRVLGMSLI*KFSINTTHLYCN

ILIHQIIPKYVFVLA

>**comp93947_c0_seq8_1** len=3375 path=[1671:0-592 2264:593-779 2451:780-1160 9128:1161-1247 9215:1248-1301 9269:1302-1378 9346:1379-1497 13015:1498-1753 13271:1754-1880 13398:1881-3374]

VCVQ*TRLSSSCGNQGRLCLLRIFNFTCSN*IVIMQQQTTITKTSSSSYRPAINNRTTIV

NRNQMRSSHGGGSAYSFSQRGSISPAYAPGTYQHLSTSGVANVRDSRQKEKKEMQGLNER

LAGYIEQVRFLEARIKMLEDQLAQMGHREKFDLQPIRDMYEAELNQARAVIDDLSKEKAS

VDARIAGLQDEVQRQAGVIDILEKHKLDYISKINQLTTQVGEYEGELGSLRLRIRNSEDE

NEKIRELLNRANDDLKKFRADLDGETAAHIEASNRAQTFEEECEFLRALLDKIPQETQQT

TRIGGMDMRAWWDKEMAQAIRDLSTEYENVVGTMRDDMEMRMNAQVRQLQSGAVKDNLES

THLREEVKKLKGSLSDRTSALNDAEARLRTLEAELNDLARQLSESRRDTDELRLKYQTDV

DRLQSELETCLHELQLIMDAKLSLELEICCYRQLLEGEENRAGLKQIVEQAVGVQGSGAQ

RLSEFITTSSSSSSAVAQSTGTNRSAGMNSEASGRMTQSRSSRGPIAFVEASIDGSYIMI

ENTTSGTKGKNQDLSGWTLKRSIPGKADAVFTFGNTVLKQGNKLRIYAAGNPPGPGDKIP

CDYIINKECFSWGGGSGTTILLDDSNNEKASLKSTISGLK*TTNCTERATKNADQFKTVL

EHLLCQYT*HFSSTFNWLYRAPCQRPVLQQSLVFSV*FTVRKLSRNIYFLLAIFGSVAQR

VLCLILKTVGETGMITFVGSDVS*CSWSGMKTGMVRL*SIMLLC*NQSLALQSEIHLTKV

YINSILVHAITQRQWRSSICL*LLFCK*C*CEWVSHVCPLC*EHSGGCESY*HRV*RSRL

L*P*FLKVMVAGKVVTISAEQG*SEPAKCPLLSQVPVDNVSFGSFIGS*IFHQFDKHNSY

DGNVELHALPISLSLYKTLM*PNATLSLVNGILS*GIMGTLDGREFDIVSIEWVVFCMLD

*IIIIVKYVVESLVMNSIC*LLRCLCGLHCQYGRE*SVNRK*RHNVTWCCIHHYSPLLMT

QHTMINPSNTGTAKHIYIAII*AQKVLLCQCTRVWSRFES*QTISV*HVVMTHYLLRFLK

LVDIFILL*FYVF*TVRSHMVLYILGSHIINV*PF*NKEFKK*KK

>**comp94035_c1_seq3_6** len=3615 path=[11239:0-99 13280:100-626 1894:627-651 1919:652-690 2458:691-1155 2923:1156-1381 3149:1382-3550 5318:3551-3569 20316:3570-3575 20322:3576-3583 5351:3584-3614]

SSRAGETRFTDTRKDEQERCITIKSTAISLYYEVADKDTCYIKRPEKQWEKDSNAFLINL

IDSPGHVDFSSEVTAALRVTDGALVVVDCVSGVCVQTETVLRQAITERIRPVLMMNKMDL

ALLTLQLETEDMYQTFQKIIEDINVIIATYAGDEDPSDPDNFNNDLQVSPNKGTVGFGAG

LHGWAFTLKDFARMYVSMFKIEEKLIMKRLWGDQFYSKKEKKWYKKTDQSVPADAKRGFT

QYILDPIYKVFKTCMNEPKQKALELVDKLKVPLTSEERDMEGKPLLKTFMRKWLPAGDAM

LQMIVFNLPSPVTAQFYRMELLYEGPLDDEAATGIKKCDPKGHLMMYVSKMVPTSDKGRF

YAFGRVFSGTVGTGQKVRIMGPNYVPGSKADLYCKNIQRTILMMGRYTEAIEDVPSGNIC

GLVGVDQYLVKTGTITTYESAHNLKVMKFSVSPVVRVAVECKNPGDLPKLVEGLKRLAKS

DPMVQCQIEESGEHIIAGAGELHLEICLKDLEEDHACIPIKKSDPVVSYRETVTEESNIT

CLSKSPNKHNRLHCRAAPMPDGLPKAIDDGTVSPKQDFKERARLLADKYEFDVTEARKIW

SFGPEGTGPNMLVDCTKAVQYLNEIKDSVVAGFQWATKDGVLCDENMRGVRMNIQDVTLH

ADAIHRGGGQIIPTARRVFYASALTAEPRLLEPVYLVEIQCPRIAASGIFGVLNKRRGHV

FEDNDVTGTSMSQQKAYLPVNESFGFTSALRAGTGGQAFPQCVFDHWQVMPGDPLDSASK

PGVIVTDTRKRKGLSETIPPLDKYLDKM*IIINLCFNKLSHVFTCNLNSDQWYWDMKQLD

CQQS*KKSAKYVDDHKLWTEDFNKGGQIKQIRFLMSSDC*HLQNVCNLESDACSPSHFKR

KMTNLE*IVLHYLLCMHVLNLEIRCKGK*TCPFCGTNMVWLSWILGKDIVDNLDILLS*P

NL*TSRLELVFSNSRLS*EATNGIGWSGLTDMVDTCHHIRMQLITGFCADLIIYKQPPLA

EILLTVALNKNRQTNNLTNICSHRCEKGPLMIAPNFYSFQSTMEKNYLTGF*NLSLCA*I

FINLRLCARSSCPRMFSIRRSSSAVASSPLNSFQNNIAE*ILSGSFSFP**ICSNSTIFS

SGKCNFFIK*RSSFLLRSYFILSNILFRSSSCFTSLFLLLYPQFAQQTRNPSSVRCQEMS

NAPGX

>**comp94177_c0_seq2_3** len=2876 path=[2854:0-234 3089:235-782 3637:783-1871 4726:1872-2040 4895:2041-2052 4907:2053-2298 5153:2299-2546 5401:2547-2590 5445:2591-2840 5695:2841-2875]

Q*LPHHSWRCQAHVCQQLQDGLVRTCY*HNLGRDDCVLAF**GQRDSFTSNDRLVFVIVV

GFIHVQFAGTWDRSAGRDTLVTVMDIRLSVVLLLGCMGITWATVWMKVSEEGINTISTQV

VETSPHRVLDLSDLIRNSSQLQIYGVDVHSPSGVTSRVEVSKVTNGEFNAEWKIQSLVID

VTGQASVGTLKFRIDSRILLRDSTLFLKYIIDKDRLLRATSCGFSFIIDRIILSDQNLQA

FVSVLQQQFARMPTAAICNGITRLHSKNPLFMFGIKEKSGIEMTVEGTMIPYSLEIRNSH

LYIGYQARSDPSSSLNVLPEVPDGDSSMLTVYFPSVFLVDTIGHVIDEVLQSRFLEISNF

RFSIRNLGSYFEQAASILQGETGYIGVFTIDEPAGPITLDGDGMLRAVAAKMNYTVSSSC

GDHVLFSLEVELDIAIKVVFNDRLFYLGVSDFRSAVTVTDSKIGKTKDLLNMADTLGIGK

NGFVVQELNGKLRSIDLSFFLTNTGVLSGTEFSVAEDYLLLRSNLSSQQTTSGWRQFGTV

FKGEHIRTEQDQSFGETIAIEPCNIKLSTESGTGDLYLSLRTVLTLAMSAIFFFVVSV*T

R*LLFGMYSPPFM*RCESLMVLKFAQRETLQYDLL*VRYCSRNLYKN*FGPTGEFHCVA*

IHVCGRKTIVWFHKETNH*SSDTSNLDQIPCNAAMM*RANSGFTGNFINV*NLRCSNNHC

KFCKSIDGTPGLTSGYSMLLRFTYKYAGEGARLIREQSGLLGTSIGAHRKFTQLRIHQQL

EIGYQT*RAQYKRLDNAY*QCKRDSK*ARSTYLPCFSRIRFFFVFSWIHKKDSDSQ*VLN

QYFVGHL*IMLI*ELQPMKRATFF*NDTKKYINRKGF**ITVMSLYST**RTLSKVSLHY

PFVCPDGNFNFLSKRAIDNTKTARIDELPPLMNGMISWGEGVEIHTGQLKV*FGLERE

>**comp94219_c0_seq2_6** len=332 path=[2599:0-292 12460:293-304 2904:305-307 2243:308-331]

RCEHYSMVTCGNRTEPLGKCDYARYACLGGKDCGPPCYIHNPSCRGRPDGLNAYPNRPGS

GSYTVCVNQRLGYTGVCGDSKIFDATLRSCKCW*IYVVLKLKLEIKEFINX

>**comp94247_c1_seq1_6** len=1133 path=[1:0-356 358:357-439 441:440-471 2680:472-483 485:484-515 2833:516-575 4698:576-618 2348:619-761 763:762-787 789:788-799 801:800-821 4793:822-833 3190:834-853 4808:854-885 2244:886-899 2258:900-914 916:915-960 3418:961-980 982:981-1083 1085:1084-1085 1087:1086-1132]

HGGDHIAFLDSLIHNNRCSLELYS*NMPINAVCVLRGTILGTIYFSQRNADGPVNVAGTV

SGLTEGLHGFHVHEFGDATNGCVSAGSHFNPYGKTHGAPADENRHAGDLGNVVANAAGVA

DIKIDDQIISLTGVKSIIGRTIVVHAGVDDLGKGGNEESLKTGNAGGRLACGVIGIAK*L

*AAIC*TVTSYWSRQHHVVSYP*HRLEYVVLNHVSYYTWDL*SHKFRFVTVKKIHA*YDC

DIGVNVWENTTNQQFPKSIGAFCFVQD*IFVL*NVYML*VGHHYFHSKTVCMQIDYISLS

*LKGLLLTVNP**N*IL*NVAQFVVG*SLSLIKQNKSYCCILLCFVCTT*ANATNKVCVT

HRAS*GFWFDP*VMTDTX

>**comp94326_c0_seq1_4** len=1416 path=[8056:0-52 8446:53-64 6790:65-219 5594:220-260 5635:261-1415]

QSVNMAVRLVWFCFSVVLVLPANAIERTITRDPIAATVGAPWPLPQTIKQSSTLFTVDVQ

NFMFVTTGVSCDILSEAYTRYLPLTFGQDYANKKHWFKRPGVGAGVVSSLNVSVSTNCQG

VYPSLESDESYNLQITSAGASLKANEVWGALRGLETFSQLVTQQSSGEFVVMESMITDAP

RFKHRGILLDTSRHYLSVSTILQNLDAMSQNKFNVFHWHIVDDQSFPFESAAFPEMSNKG

AYNPVTHVYTRADIREITNYARLRGIRVMPEFDSPGHSQSWGKAITNLLTPCYSHGKPDG

SFGPINPLPESTYTFLKAFFQDVGEVFPDHYVHLGGDEVSFTCWMSNPNITDFVTKMHYT

NYGQVEQYYMQKLLDIISGLGKGYQIWQEVIDNGAKVQPDTVVEVWKKPWEQEMSKVTRM

GYKTLLSTCWYLSSIHYGQDWQDYYQCDPLDFDGMPSLVACLHSSFTSTAYI

>**comp94743_c0_seq3_1** len=3288 path=[1:0-118 120:119-135 137:136-359 361:360-789 791:790-808 810:809-1376 1379:1377-2507 2510:2508-2527 2530:2528-3000 3003:3001-3016 9096:3017-3287]

AAILFVLQGYYCCQSCVINAFKMSSNATESSTEREDYVYEAKLAEQAERYDEMVLSMKKV

AKMNCELTVEERNLLSVAYKNVIGARRASWRIISSMEQKENPDGQKSEDKKVLITEYRSG

IEKELKDICNEVLHVLESFLIPSASSGESKVFYYKMKGDYYRYLAEFATGNDRKEAAENS

LVAYKAASDIAMTELAPTHPIRLGLALNFSVFYYEILNSPDRACRLAKAAFDDAIAELDT

LSEESYKDSTLIMQLLRDNLTLWTSDMQTEGEESQKDQLQDVEGEDGS*RLVVLSTLKLT

LFVIFLSKC*ML*PGP*NTVCSILLSQPLAMASCS*DFIYIQDELLHPVDSRSHCVHSCT

*FICLVACNINFDKLYIPSSVRYSAFQLTQKCR*KLSCDMT*LMLICYHHYLYLSVLDLI

PNISN*QGPVICQ*SCRLFGNLNVENKQKPNFLLTVCPHLSPKSCSVLFL*RYIIR*WIL

PKL*S*TLAISLLRNRCHGH*R*QGGWNILPNKHVCLNLC*VSWIYLTECGLTLCQCDIL

DFLCGPRYESFIGSR*SQLTQVWNTAINLTLQFQCSWI*WKISY*M*F*PKQKKT*TVEL

QNYCHLSDGAPLFSDITSFNAVVQCEDFQT*LCNHVLSVMSNNMM*VWYRLDPICQDCFV

VINLCSKCLFFLFFIMLSYD*SSYFALFNVTSSVSIKLCLFFYLERK*FLFLDLAFAFWP

PRPMCLYLCPLLYVTEHNHSSTLYMT*VAAALLLPQPARLVL*SQLVLAVTMISMSSGQE

IF*EKCNNFIN*CHAFIIN*SIFKCR*LLLFIECRAYRLLKLMVFITQRSGTIPIEVMSK

SPFHPLIDQ*NAQQPTLASQKATFSGYDGTNLECANSHSRLFLKIDELLNSKFEN*TMTI

LKYLRRRDYWNYFLFKYCSHWRDTFVVQLFQICHTACH*ATLAGGKVILTWNGIEDLCEA

IVSTRKVHLNRYSEKC*DFLGQFCENSPEMKAYL*ISSVQNAS*G*HSSAIVIVDSLNKS

GFHVINSLAFGAD*VFLFGLEL*TMAETKVLKLLTVVTVMRFPVGCDTVY*KEKCLND*L

TLNT*MKFPNIK*LVP

>**comp94766_c0_seq2_2** len=3475 path=[3453:0-76 3530:77-1007 4461:1008-1009 4463:1010-2063 5517:2064-2088 5542:2089-2301 5755:2302-2303 5757:2304-2440 5894:2441-2479 5933:2480-3209 6663:3210-3348 6802:3349-3474]

*SSLQTFNR**TRKDRRGSGRVLERRQQYLVALRNSFSLASIRMTSNGLVNEGYDGLPEK

ENGYPNGIDPIDNKVIEVEEEYDKEKENEEDPWKVTTIEVEEKPWAELNCAELIFRLCKY

ILGVALILGFLYMFICSLDFLSSAFKLLGGKAAGQVFQNNEILSNPVAGMMIGVLVTVLV

QSSSTSTSIIISMVGADILTIPIAIPIIMGANIGTSVTNTIVAVGQLNNKSDFRRAFAGA

TVHDMFNWLTVIILLPLEVITGYLSRLSGLIVNSIPDLAPDKSADRDFLKIITKPFTKMI

IEVDKSAITKIATNTHKGESLLKTCSDEVTPWSCCSDELKDMNVHNNESYSFTKQIQVCS

ALNSCAQKHTCSVDFWKGDTFSCDNFALDNTCCETFRTGYTFPTTINDTVKNTFCGELVA

NCQNNYLVTKDCMESAWSNNTAFSCDTWIGIETECIKPKTHLFMGLYGKIDDIYIGAILL

VISLAVLCICLVCIVKLLNALLKGQIALVIKKFVNADFPGRCSYFTGYLAILLGAGLTIL

VQSSSIFTSTLTPLVGIGVIELDRMYPLTLGANIGTTTTGILSALAQDGSKIRDSLQVAM

CHLFFNISGILIFYPFPFFRPPIPMAKFLGTVTAKYRWFAFAYLAAMFFFLPAAVFGLSI

PGWYVLAAVLIPIAIVVIIICIVKLIQRKRPTCLPPKFRNWKWLPEPMRSLRPYDNVMQK

VCCPCLLTDDLKEEDDDSKQLKSNGTAVVKQPRGKNVDTRL*ISQNQRTVSF*KHLKTIS

RNIVFYITRF*AITIIACSMVCISTSKCIITL*VHRIII*YVTLY*ILGMILQYGALRKV

IVYRSYRSFFQMSYVLHRSMKT*G*NWQFI*FS*EATNAIAD*CMSSIRIA*IDARDASH

LIVWFRFNYVQTDVI*LEPVRR*TTTIYVTVFCFLHTPEHRTIWSQSHINT*HISHE*LL

ASLY*PCVTVIKKDSDMYEGQNL**LKPV*L*RCEQK*GT*NCMLVYHFKKQPA*PWLQF

SPGEDQLNTYNIFKGNLYNIYDNLLYLLTVQPIC*KIKLKLFRKPDNSSPL*HSF*SSII

RGLPLYIIYKLFLYLRIKVFRLCD*ENVCHF**NYPYDIYISFHRHMLHHLTCVICEIYV

SFVCYLII*YFTLNIFAK

>**comp94779_c0_seq1_5** len=2069 path=[7555:0-14 7570:15-20 6986:21-23 3495:24-37 3509:38-236 7621:237-237 3709:238-1198 4670:1199-1200 4672:1201-1732 5204:1733-1837 5309:1838-2068]

VT*L*QGSTHTAI*EGRLLALPCNTRKTSGSRRWFRGTGLCSVDRVLTIRVDRTTNVEMS

SMPTLKPRLVALVKQPGVVSFGFNVTATRSEPFRYVGIVVDNSLAKQAGLRSGDRVHEVN

GVPVDGLPLPDVQGMVAQRRDRVDLLVADPMTDQLYRDNNEVIRGGLPETICIQVPSDDL

SPSQQEFTNGVEEIEAEVVDPDAFLGDDGMEDIPPPITGYQSNGVIHSESSPMKARVCTV

KKWPDYQGFGFILHGDKDDPGQSIGKVESGSPAEAAGLRPGDLVVEVNGVNVEAEYQQQV

VGRIANGPVPQQTTLLVVDPDTLRFFRGHSVSVHGSMVDIERIETPPRALVPASTGNIKV

RLCHVKKCADFSGYGFILHGERGVPGTFITNIDEGSPALAAGLLDGDKIIEINGVNVEGE

EQPGAIRHIQIGIDGNTNETRLLVVDPESERYYKRQQVQVSGSMADVVYLTNPERELLPP

PPVIEPQSRNNNSQYRARLCHLKKWSMFDGYGFYLQSEDGVRGISVGKVDPDSPAEAAGL

RKNDRIVEVNGVNVEDDSQNDLVEKVVAIPEETRLLVADSATFQYFLDAGIMVNGNMTDV

DIIEAPDR*TDGDGTSRKDACQIVTRASPLQLKSKTFISL*N*SCVKLPSACMYTTSSPM

NRL*HRRYSDHM*TGYNIDVMQPTCELVIX

>**comp95150_c0_seq7_2** len=1582 path=[1:0-449 18140:450-456 18147:457-498 18184:499-515 517:516-519 521:520-693 695:694-739 741:740-903 905:904-1581]

LD*LRTDLSQTLAMASLDELWGKLSGSADCKSLLKKHLTKERYEALKDKKTSFGGTLAEC

IRSGCLNLDSGVGIYACDPEGYTVFADVLDAVIKDYHKVEKLDHPEPDMGDLDKLNFGDL

DPSGEYIVSTRVRVGRSHDKYGFPPVLTKQERLKMEEDTKAAFEKFTGELAGKYFPLEGM

SKEDQKQMTEDHFLFKDDDRFLRDAGGYNDWCSGRGIFFNSAKNFLVWVNEEDHLRLISM

QKGGDLAAVYKRLVVAIKTMTSSGLSFAKRQGLGYLTFCPSNLGTALRASVHMKIPNLAA

QSDFKEFCDGYHIQARGIHGEHTESVGGVYDLSNKRRLGLTEYQAVEEMRKGVEACLAKE

KELAAKK*TNNLSPSRPLKAVLCSELVNNSTCVFIYLCPQLGKAPREAII*IFR*GYLPF

SIHRSTRLALSLATQVVGFPHTGGHKPTTVKGQETSDDQC*CNLVQCVCPAVHSVSGVGG

RTVGHGSILLSLPPIGAYGAIP*CPVISTRIIVSKPYFMTPE*KQEE

>**comp95150_c0_seq8_1** len=1884 path=[18147:0-41 4736:42-199 4894:200-209 4904:210-236 4931:237-403 5098:404-416 5111:417-440 5135:441-491 5186:492-509 5204:510-517 5212:518-521 5216:522-1263 5958:1264-1267 5962:1268-1349 18907:1350-1350 18908:1351-1357 6052:1358-1359 6054:1360-1692 6387:1693-1713 19027:1714-1883]

LKMEEDTKAAFEKFSGDLAGKYYPLEGMKKDDQTQLVADHFLFKDDDRFLRDASGYDDWP

SGRGIFHNNEKTFLVWVNEEDHLRIISMQKGGDIGAVYKRLVSAIQEMEKKLTFARDERL

GYLTFCPSNLGTTLRASVHIKIPKLAAKKDFKNICEKYKLQARGIHGEHTESEGGVYDIS

NKRRLGLSEIQAVQEMVTGVQEIIKLERELQKGKGNKSSSCAIL*ALPSPLVEAAAILIQ

PAYLFSRHFETIGRHFDSTSTVIRPPF*MYQYLYTDIILKRHSYMRP*TSFL*SNNFVCM

SIKTKTFI*HNRHFYTVSVCSGFKVPRAIHLF*ASPFKIQKYVEVGSYRINPDIIGMKMS

IVLPGWCECAGVYYKS*CYRLFFKHAHCLNR*RQCQNLYV*NTNDLPPS*IFFSQR*L*I

NNNALFTLVSRMLTVSTSIL*CVRVTMLICNRFVCK*S*SENMLLSLILQGAFDYYTNST

NTLSFVAGVKMDM**M*ALHSRRFQYTVIYFYNLYCIYRFGAFDTNPMIL*RHH*LQEAT

GLSNCSCEYTTFFDTDYYRHLCQRTVQIKNSLLRTLRSLSHCH*TTLFYLLPRPPCNAKA

QMKQV*ISPAAGCESPNSLGSFLV*MGL

>**comp95608_c0_seq1_6** len=2002 path=[5207:0-14 77:15-206 269:207-215 278:216-424 487:425-440 503:441-502 565:503-536 599:537-585 5333:586-622 685:623-629 692:630-1091 5413:1092-1099 4341:1100-1109 1172:1110-1153 4249:1154-1173 1236:1174-2001]

TSHQSL*AI*HCVLTHFTCGTSSGTEGNCNDHQTIPALHHLEMLPALVILLVIGQHSVRA

ESNEEWMKIAKDELNRALNKRDYNDVAKNVILFLGDGLGISTVTAARIYKGQGQGRSGEE

TLLEFEKFPNVALSKTYNNDRQTPDSAGTGTAFLCGVKANLGTLGLSGAARRGDCSSAKG

AEVTSILDWAIAEGKSVGIVTTTRVTHATPAAAYAHCPERNWESDILVPYTNGTCKDIKD

IALQLIEKKKIQVILGGGRRAFMMSNQTDPETNGTFKHRQDDRDLIKEWLYQSSIGGQSA

KYVWNKQQLADIDPQTTDYLLGLFEASHMQYDLERDTSPTGDPSIAEMTSKAIQILKKND

KGFFLLVEGGRIDHGHHANSAKKALQDVLAFDEAVAESNRLVSLSDTLTIVTADHSHVFT

IGGYPNRGNNILGLVDPDYPNGPNDGMPRTTLVYGNGPTGQNRVNLTIVDTTADSYTQEA

TVKMPYETHSGEDVAIYARGPMAHLFHGVHEQHYIAHVMSFSACMGLYKGDCDRVTSTAT

ALQFNTSVVFLLSLLFWSRL*SLEK*TLNINFCKA*IPPKVVPGLWATRICILN*IATER

RPQNMFFAGCITYL*HLLNLEHLIIVLKCIYSADRVTSEKYDK*M*QKRVWVVRLTDLFD

VWHFKSX

>**comp95743_c0_seq1_6** len=5098 path=[5078:0-84 5164:85-530 5610:531-550 5630:551-778 5858:779-851 5931:852-866 5946:867-921 6002:922-3952 9033:3953-3968 9049:3969-5097]

NRS*TQ*RPQLLCIGGHGHCELRPPFWTDYPSDHVI*EPETNIII**YSLTHC**RSYFF

R*PNLIPSVTR*LGGIFRNTPEFSLVTSLD*QPSSSNIRHAC*ETAQSKMASSKVSKANQ

PRAESYRYAVTPSENDDKKKSKKEKKKENLDELKQELEMEEHKIPLEELYERLGTDPNMG

HSIEKAKEILLRDGPNMLTPPKTTPEWVKFCKTLFGGFSLLLWIGAILCYIAYSIQAGAM

EDPPGDNLYLGIVLTAVVVVTGCFSYYQEAKSSKIMDSFKNMVPQFAVAIRGGQKHNIHA

EELVLGDIIDVKFGDRVPADIRVITAHSFKVDNSSLTGESEPQTRTADFTHENPLETRNL

AFFSTNAVEGDCRGIVVRTGDKTVMGRIANLASGLEVGETPIAKEIAHFIHIITGVAVFL

GVSFFVIAFILGYFWLDAVIFLIGIIVANVPEGLLATVTVCLTLTAKRMASKNCLVKNLE

AVETLGSTSTICSDKTGTLTQNRMTVAHMWFDGRIVEADTSDDQSNATYSRTDLTWMNLA

RIAMLCNRAEFKLGQEDTPILKRECNGDASESALLKCVELSIGNVTEFRRRNKKITEIPF

NSTNKYQVSIHETEDPNDPRYLLVMKGAPERIMDRCSTVLLNGKEQPIDDSFREAFNAAY

LELGGLGERVLGFCDYFLPIDQFAPGFSFDPDGPNFPITGLRFVGLMAMIDPPRAAVPDA

VGKCRSAGIKVIMVTGDHPITAKAIAKGVGIISEGSKTVEDIAAERGIPVEEVDPREAKA

AVVHGADLRDMTPAQIDEILRNHGEIVFARTSPQQKLIIVEGCQRQGAIVAVTGDGVNDS

PALKKADIGVAMGIAGSDVSKQAADMILLDDNFASIVTGVEEGRLIFDNLKKSIAYTLTS

NIPEISPFLLFILADIPLPLGTITILCIDLGTDMVPAISLAYEQAESDIMKRQPRDPVKD

KLVNERLISMAYGQIGMIQASAGFFVYFVIMGENGFWMSKLLGIREEWDSVGINDLEDSY

GQEWTYSQRKRLEYTCHTAFFVSIVIVQWADLIICKTRRLSLFQQGMKNHRLTFGIFFET

ALAAFLTYCPGLDQGLRMQHLRLSWWFPAMPFSLVIFIYDECRKFILRRNPGGFVERETY

Y*GLFILSKDKLG*P*NLQMPLGGDFAGERTLGKGGYSLGSFKCERK*FYAQEHFYGLRY

DVMLHLSELLLCK*SCHCYLQIGAV*LRIRNVNHLLSHVRLILGSDAGKMAALLHQVTLQ

LPPPSRVARSASFIVFDGSQHTLTHTHTYSRLACSYRNPAWAMSTVSSDRLGIGLLLLLL

LDII*WNDFR*QNFIINLYCTNCLIFFVHFSMYFAIFFKL*L*LHVYVCVRVSWI**NG*

*LHFTYQHAFFCSPHSMLKCDLGHTPELLDEQFQDSGQPLQPHLHGKHGSVTRVE*SYHF

LFTFVCISLILKRNLNINSKVWGNKSLFVFVL*TLGFISYKRILCYLWSSEMLGSSQLL*

CLPD*RPCKLLL*SITHPSSKRYKILRYNYLTPIKSLYLFIGYSLNISPHSSCIGFHLTK

NMGID*CQNCNKQICVRIYFCLL*MTPYQIMQLMSPCISKIMHVEYATEGSILVFVLHTF

SVTSYLARQ**LLIFPQLERDIRML*HMQYK*L*MCEVCC*PHVTPLCRDAPPGGPALHY

VVISPWNFL*G*KNKVI*A

>**comp95784_c0_seq3_3** len=3920 path=[1:0-33 11837:34-34 36:35-245 247:246-1613 1615:1614-1633 1635:1634-1648 1650:1649-1782 1784:1783-2946 2948:2947-3077 3079:3078-3099 3101:3100-3124 3126:3125-3126 3128:3127-3687 3689:3688-3693 3695:3694-3919]

VFNTECSRSCKMNMFKVTGLLLALCCLQSIYGAPVEDSQGGSEPPVDLDAEQQRETGMEI

VNTETGHRQKRAFFYTRPWPDNIIPYRFSPNVPVSREKEWRGFMATLENNTCIRFKEKAD

NSSTVPGANNTKGWVYIYHSGICAAHIGYSSKGRSLSPCAGAITHAHEFGHMLGLVHELN

INVRQEYINANLDNAGNHYRPEQELASDVEQQNYKSCGFSTQSIMMYNNYLISKYGWPTY

TYLYPGAARPSVVSQFLFFKEISINHQCCAETSCDSITCANEGYMGKFQDKCQCVCPPGL

DSRTNCTTALNSRYPVADWPTPVSYLAPEEGCPPSGFVMGTRVANSQWNITYSSPNTLAR

AQSPGALKLDFCTNIGNTYTGKWTPGSMCFYNPGIECSDYGFSDAFYQVNEASTQDLCED

PVTNCGLPAGEFGEDAVYQFCCRDDRFPGDKLSLPNDFPFTLLKTQNKDCEQVAGMNVQS

EYFTFLQDGPSEFNFGDTLPSFTESSDNSLKLDYCRYEPVDYSCGGVIDLSETHAEEVFS

SPSHPEAYGKNKECNWVFKAPEGSRVLLECDNIDIGCDASFQISKNMLGEGNVDPIYANG

NRMSPLLSIANKLRVTFSTGIQSCSFTGFQCTAKLASSNQLPHSLANRGEDYCGQMSYAV

NEEGFSQCLPWSDVFEANPEFFAPQASTRNHICGFDSNYCRNVRGLKLMPYCATSVHLGN

VTFTYCDVGQFIKPFNVFDDCETSLILDATCGDPETRRTCFKACDDAGLISDYPIAEAAA

PDVSCGDPTLPAGTTNLNVNPAGYNVGESITVKCNSGDNSVQFVCLSNGQWSTQDNFCAG

CPAGFIERSGNCYKFPAGTFKGHAARAECAKDENANSVLVYPESEDELNNIIVDLRDNVL

GLSNAKVWFGMTPSGGGWVSDFDGSTVVASYLWKNGNPNGISGNPAANMAWLLPGHHRFA

NKLAKTNLNKHAAYALCKLPMAGGSGSCSDKKGDCATILSGNPNLGYSASFAKEFCEQTS

GNCAPGTCTVSTLPTGVVADAFSVETGKVVNYECDSGYVYQSGNRIRACQQDGTLTGTDI

ICEDGATAPRQMNYYELTDSRQATGENQILAGPSPYLSVPVDSSVKACRAYCRNDGALNI

QFWNSTGSPNEFTLTATKQVECLAGRAMTWTFDTPLSAKAGDFVGVQDKIGNLVSMHPCT

GTTKLLTVNNVMRSKSTSDDPAVGDTVTFGGATCVTLRIDCFYVPDAHTGGDVPVTFRN*

DGISSYAFLFITCVYIHYITRSTIRVDSCRVHVKCRTCMIP**TIS

>**comp95930_c0_seq1_6** len=1578 path=[1:0-123 125:124-149 151:150-171 4451:172-175 177:176-177 179:178-547 549:548-558 560:559-1020 1022:1021-1514 1516:1515-1535 4815:1536-1556 4816:1557-1577]

QPGYGQPQPGYGQPQPGYGQPQPGYGYATTDNVNVVVAQPQTMVVQPAVRPPDYLILSIV

SCFFCWPMAICALMYSNNSRNSAHSGDLVSAESQGRTARNIAIAAIVIGIIIVVIAIVVR

VVLVSSYSTTYYYG*HRTGVGQRTTRLL*INCK*EIDLSLV*LNSSVVDRKV*NPYHPVK

DFHSCHTWKCCNHCAGEVTLQYHEKLSAPGQGVCLIYRIRSVHVVIFMKHTDMTTFHRVL

SSKTSPCRVFR*RQQQVNVLSLTVSKETLYVHFHQLHNLIVDLFVSHLQLFTSYMHTNNI

RFCFEQFVIST*TWLEVSVIKI*SFAILFSIQLGSIRCLVSRLKLVQRLVCRK*DGSLCL

VNTKRCIC*TEQLLCVL*S*LFVKHDRKYA**IFFPVSFVVSFIHKHDDVAVCIHTYNTV

FTQTLLFEITYRYFRVTEFEMMVKRLNIPFLFADVIVLFICEQSSFFLKKR*ITVC*LIM

SMVHSYFNLTSIFS*KLFLPFV*ILFFVYRFHTFIVKSN*TVLYKX

>**comp96136_c0_seq5_5** len=3090 path=[1:0-417 419:418-420 422:421-1744 1746:1745-2463 2465:2464-2631 10180:2632-2772 12595:2773-2790 8923:2791-2814 12621:2815-3089]

YLCFHIY*NALQKKQFHQSALKQINNSLKTRVINIMPL*QECVTVMCNCTSSTS*LSHSH

YITDTNSTKLN*LDTNLD*YTRNVFATYVVSQIRFS*LHIKYEPDSRAGKIYFLPAIHRI

NPSWAFGCAVVSYTDVVLCYAMCLCLLLL*FQTFNGFLTGQPESKLTKALRALNEDLDNG

KRFFIDDMLCYVDCHLLPRLQHIKVATKRYADYNIPKEFPAIWKFLAYAYQQPAFIATMP

RDQDIIFHYKKKVTAFPEVKNSTIEKFSYDTYVPEEILEEIRQENFGNEEPEPEPEQVEE

VEANSEMEPQFDNNGLEY*VPRV*RRHCDGREL*MCRQCRLDW*LSIRPRPVREPARHHS

LARAC*L*TDMKTFVKREC*LIIYPDGT*WHSHNDQGPRRRC**SSDLILDCNGSYQKHV

IMLDMQFKICGISLTF*FRTKYNFAVFLLTFKLLHLRTILQERTC*LDLSVFLFPSDINM

SFVEPLFF*EDNCRSGRSENQSRERTERL*LVILILLNSILEQFLEIF*EIK*CVI*H*G

YTIKICNCFMNKACRCCR*CFAGQYFSVRHII*MIKDNIYRLPIKMAGMYRTIQEANPAS

AVRGKPL*HFYLSMHFVNRTFIYVSSWFFC*RCLWDKKKP*ITCLVVQ*LLSFITLNLDQ

IIVFCVCLLWYE*EVSWFQ*RPYS*CLYTLWRHDIQF*GEDVSFCI*QPLQIKD*KY*NF

EQNWDPIFSLDAKDGYDSHCSLS*SDILRWGV*NKNSSTFTVKYVGCGDSIKGQEESV*L

INTELFMISSIKHNHKLPCNVNEEFVPVSD*HSGNVQIPVFPFTRFVFIIHNFDMKSC*H

LCILCKEYHLLHFANGRSYGLIVNQIE*ASQPYMFSLLCMASAEFGNIK**HFS*F*QKI

ETKYMVSILRLDL*LFCLFSKVCDTRTNLSDPGLRSLILDFSQIVYQHDDSCLYENSMCW

TSSCIVHVG*SRMVRCCTYYSFVICAFIVWYIILLVV**PDYTKSPYTLISWMSISTAL*

HSVII*EIKX

>**comp96576_c0_seq1_4** len=2097 path=[1:0-136 138:137-141 143:142-266 5509:267-273 5516:274-421 423:422-506 5620:507-528 530:529-715 5687:716-729 731:730-732 734:733-964 966:965-1002 5754:1003-1066 1068:1067-1070 1072:1071-1421 1423:1422-1485 1487:1486-2096]

LPAYTLLTKYIQNSFLDAYQLVGEKFKFATKCTEIICKMHISTTTSLSPVSYSATRGAHI

KFSHK*PRFRGRSEHQDRGQILGVADSSQFHSKKVDSQISKMAGTDADAERTVVIAMDGS

KHSDEAFQWYMKNVYRKNDHVIFVHCPEYHTIIQSPMVMADVTVLTDLYRDEEKKVKEFL

ETLGKKLREAGIGGKVKSIGGSPGEVVCQVADEEKASLIVTGTRGMGQIRRTFVGSVSDY

IIHHSHVPVVVCRLKGSHDKHGH*WLISLKLTLPVETEVVG*YNTWTLLSSTLWLFSSSP

CFLLPRQSSKLEKVHGLC*KVLLEK*SLNIVESISCALF**I*VVYSVF*AGYTEFALNF

LTDCDSW*SWFREICLFVWTTMS*TNVFEKSNS*SL*EKLRTRSQLHNSIQTMQKIVSTW

MSLSYQG*FSLSFV*LSKVFSFS*IYINWDHFNSNLEYLLPFAAGLGPNMNL*HGLASVL

QRIPNQRWI*LLSVGEISVENLLHN*LENMPGQDQRIL*V*FFTY*SECSFSFSCP*KNT

KN*KNLHIQ*LRFWYIFIAIHIIKLFDCCLQFCFQSGWEIKRY*PMAVLQH*LSSL*LDN

HTSCLLYN*TDYQLSKYQLVLLGVIRKTCCLSRQINTVLAS*NVCEM*TCDKLMINLQLL

GLCASLNETVCFNEY*LLILLLYAEACYNQKSLIKQLVG

>**comp96622_c0_seq1_3** len=2801 path=[1:0-237 239:238-261 263:262-778 780:779-783 785:784-1063 1065:1064-1258 1260:1259-1259 1261:1260-1283 1285:1284-1380 1382:1381-2253 2255:2254-2800]

RPFPQGHRQGIGVGLRLGASAVFCPCAHSRNLIQPATMKISVFLPTVTLLLMFLIEEGLG

LQCHVCNSFLSESCAGTLTMESDTLTPCTNATACRKIEQEVYYDDQYDTRIIRQCGHEED

PPLTCISRTGTYRYKVQYCHCNEDGCNSAVTSTISVTMLFLTLGLAHFAMKHL*VTDN*P

GFFLPELPNSLQYVNRSLFLCTLFHNGFRLASEIFSSDIWIC*V*GPFMY*IRDHKLHMG

YKKKTIVPNFSVGKSCKGF*ISFSLKC*RPYSDWS*KVALFRIAFGVICR*IKHI*YFYD

TV*SLY*THSLENHLKELSFF*AIKSIHHYDFDIILDCF*TVAVFSVQDVALMGGWRILL

TV*KQNRLHVTRELYARK*SFYVTLNIQ*MHILHCNFHCLCE*NRQCLDVMSCTCFTFPE

RKYRPVKRLQSVIMNRNLSNVMNGFLHLKTFFLRISV*FSR*VKCKSRCKKILFHTLFDV

MVSH*NEKAFS*YSNCSSAQTVYCNIEYYVITVC*IMERMRFYFLHIM*IFVYC*NVC*G

IDSDCNPDM**CLNIII*PRCFCTVI*KNLHVFYHLSQSDFSQNHQYDS**VPSI*FWCV

*VYRYVKMLMLFVHHGPYYDLFLRKVFILFSYLIHFEMNSV*PSMLSLEHAYNGLEMLFS

TRF*GPFLCNILLLFSKSLCSLFSCWRFYSLP*MLHIVASSFEK*CHL*QYIYLK*TVCM

EVNFKIYSVSRHWRTHYPETNCSIWTASFNRKK*MFFPHQLLGISSSRYLLHHVVGKTPA

HSLPHSACFREVKFDLKLIDWQVIIMYTSMDSSRQFQEISGQF*QFEDLEGNQSFVTFEI

WS*YCI*IFLSLRRGMYSDF*PVFHVNLKSFIVL*AWFNFNSLL*FILSCDHLVWNELV*

FNRIQEGLLLRLV*TCVLCECSCIFFQINR***

>**comp96833_c0_seq1_1** len=4779 path=[1:0-1769 1771:1770-3466 9665:3467-3572 3574:3573-3618 3620:3619-3641 3643:3642-3960 3962:3961-4015 4017:4016-4031 11456:4032-4312 4314:4313-4331 4333:4332-4778]

VASRRCTY**PRYMAPIVRCVSFL*TQHTLWVAREQHTLRTVPSLRHSVRGSDLSIDAIG

QARSGIPNSLSSQSSYGLRTVYRTSLQSLGSMLTIYDRNLVGPGSQSDPQIPTDPPTRII

TRRREHKLLTV*QFKNIYAGVQVESVRIYNGWYTQCSCIQDCR*RQHQRLHLPTPGSTTV

N*V*RRLGMASDYPRIQTASAVLGQILLVCVILINAKTPKYEFEIDFQHKPVPWHKCPQD

WIQFGGKCYKVILDSATWDEAKIICRSYGGSLVKIKGFNKNKGINKIIKDLLKGNSVKIS

EAAAQYTWIGFQRAANGSYSWSDGETTAEQQGFWSEGNPVQYKHGSSKCTDVDLLSSKYG

EFRWSLRGCEKKKPFVCEVKACNKHSYRCADGKKCYCKYCKCDGQQDCQDGSDEWDCDNG

RRCGGELSGASGTFHTTNFPKKYPKHTDCVWRINTAVGTKVQLEFVNFDVESKYDSATVY

DGADDTSRQLGKYTGNKRPAVPLASGNFLLVKFKSDHSKEKYGFNATWNAVTFSGPQDSC

GGMLTARRPENWFTSPQYPNNYPESIVCDWTITAENSGDIVTLQFEDFELESPYDWVEVR

DGSGEDDGLFERFTGDSLPKIIISSGQSLFVRMKTDFAYVLRGFNATYSSGCDVTIRSGH

AKIESPGYGVSNYPNDISCSWRLFDPQRRELSLIFQANFDTETSIDQVFVYNTSQEVTGS

HASIHSGRISPGPSRTDVGEFFVRFTTDERISRPGWTAAISYDCPALDVSSPLNINTTET

AYSTVVSYFCDLGFLREGAATVICDMEGLWRPEKLPVCIEIDCGSPGVPTNAVLVSLDKT

TYGGTAFYQCLEGYDLIGQNQVTCSEQGWSPLPACELVTCPKVHAPVNGRVEAEENHYGA

VITYYCNNGYHLVGSAQAHCTADGTWSSVAPTCQPDQCPAIQGVTNGRPNVTGPVKVGES

VSIDCNHGYRPDKDNVLLCQEDGLYDKSVPACLDINECTEEDLNTCDFHRCVNQDGGFKC

QCESGYHHPDGDQTRCEDINECDSNNGGCNQICNNSPGNMTCSCNDGYFLYPGPDSANID

GQVLVPEKTCIATCSGFNVTDGEVFISGTPLPSGKYIHPTVAYIDCQPGYVPDGPGNVTC

QVAGGWTATVTRCTATLCPKPDHPPNGEAMYSGLEPGSTLLYTCDAGFILVGPDQRFCDL

YVDPDSAVPRHDWTGDKPTCEAVDCGVPARPENGEVHYSGTGYKSVATFTCLCGYILDGS

AVRTCQASGSWSGNSTRCNVRSCPRVPSPAYGSIVGAWETFPVGSVVSFQCDRPGYQLSD

PWPLECIADPHLRISLEKPDYLISLSVDYRATASIPKHCVEYYGWESVRNLQAFKASLQL

LCTEFGQPVINIYQTTNGSVTGSVVNIQGSIGFQVRDGEAIATACQCGSSVINALVGLED

PTMLDIRASTELGCPAVSAISGSISSNASQWQCPPGLILDNGNSACGENTPMCLKKCLEE

LEPTPTPTTSVTIIPTSPAVTSTDAPAATTDTEQTTTRPLTPTTQDVHSSTPGSLTTQGV

STQPTTTGRPSTQSPTTQGPSTQGPTTQGPTTQ

>**comp97336_c1_seq3_4** len=1306 path=[41:0-116 158:117-170 212:171-208 250:209-229 271:230-255 297:256-526 6777:527-531 573:532-549 591:550-596 638:597-600 642:601-803 845:804-830 6876:831-1039 1081:1040-1267 1309:1268-1305]

RKCLTDGRLHV*KGCMSSRSFARAVMRAIRVTEFGGPEVLKVQNDVPIPQPKASEVLIKV

SAAGVNPVDTYIRSGVYPAKPALPYTPGMDIAGVVHDVGADVKKFQKGDRVYSMRTSSGG

YAEFTTVEEKYIGRLSDGITFQQGAGIGVPYYTAYRALLLRGEARPGQTVLVHGASGGVG

SAAVQIAKSLGIRVLGTAGTPEGVEAVKKNGADLVFNHREEGYIQKIMDATGGEGPDVIL

EMLANVNLEKDLGMIKLRGRIVNIGSRGPTEINPRATMGKECTITGVMLMISSDADWKEM

HAAMEAGMSQGWLRPIVGKEFPLEKAAETHDLIINTKSAQGNIVLKL*KLKQLQQPCNSA

HSHSNSHWLLRQSFTVTIATISAKCFP*TVVYAALCNVYPSLLNVYPISVLVL*SHANIQ

DL*VFKGRWGSQVVK

>**comp97362_c0_seq6_5** len=2670 path=[2648:0-275 2924:276-288 2937:289-685 18235:686-1160 18339:1161-1166 3815:1167-1550 4199:1551-1828 4477:1829-2197 4846:2198-2213 4862:2214-2245 4894:2246-2263 18635:2264-2270 18642:2271-2272 4921:2273-2669]

AKDIGNRHMRHSGVSVIGGKANSICYPFI*TYHEF**VLIS*GYFYRVYAER**LRSHAP

LINHCQHCFGT*PKLHICLTFVT*LQRSQTDLGCGLLQAFLVNGAHPVIVALMMGMFLNL

ACCLLLTATIAEAQSCGGNLAASSTSKNATSQGYPDSYPSTTNCVWTISADNQDSKVRVT

FLEVEILDGYDVVKVYDGNSKTGYLLGQVSGTDKPTFTGQERSLTMELTATANATRPSTK

VGFVFGYAETQDTLPLQRSVEAQSDKAVLMSPRFPSGRVKDMNAIWSVRPELFNKEVLLS

FVEVDLGDNCNNNFIKVYQGNTSAVEVSKLCGNQTDTFFSKHSELSVVFRTDSNLARRGF

KADYTMVGFAYFPGCYGSPNEVRVFENVNGELESPLYPNKYPNSMKCRTLLKAVLTDIYI

KVEVTDMNMPQSEGCTKDYVEIFDGTTSTSPNLGRVCGQTKLTVTSKTAGQDVMVTFVSD

SNEGGKGYKIKYVGVKGNGQATISSPVGLIVGIIVGCIGFYW*SLLSS*GESV*GNDHWN

NVNFTVVMENQGFLELLPNQINQRRKLCLEAIYIIYSDHVFKSSGGIK*YQMPVWVRDKL

SQVMP*NECKNK*LNHFKLSLTKR*TNLFLYAYESQFLVGLAKHIILANKF*FNF*VKRM

ICIKLLISFQYCTHLNLKNRK*VRPSSV*IHRFRLTQDSPKDCVIQEMTFQYY*STSSSC

KAIFQQL*CLHFTISEVQSSILLFAKSSKIKATIFKASAKLPRMFHSRQWRTF*KSMHH*

QK*CQMQVPILTYTIHSRCFSGEHLFSLCVCL*R*GNILWKFCDHLILLLLERDL*QTKT

QCRPIYSS**YSRNIAC*LYNRLCF*QISIAFWFVC*LYCSAPHSAIFQX

>**comp97435_c0_seq2_4** len=1886 path=[1:0-269 271:270-282 5087:283-390 392:391-446 448:447-548 550:549-563 565:564-1223 1225:1224-1348 3964:1349-1354 1356:1355-1385 1387:1386-1386 1388:1387-1410 1412:1411-1685 1687:1686-1688 1690:1689-1885]

GRQRHKVFRPLFDLTIKTGHFHRAVCVGQRI*QILFGKILGL*EVTFRVLILGDSFVWNM

WKIGVVVVVVLLGMMMETDAKKPRRRKPQKPGPDAELQSLKTNISSLWTEMHKKMESRVM

VHVHGDGDDSAETHKGQDGTHVHIHLHVDDHHGGRRGHDDHFDHYDDDCDEDYNHDNHES

HEDGEEGHAHNDSHEGHGHHDSHEGHGHHDSHEGQGHHRGCRGRKRDEYIYGRCNMDMRN

EPVKSGPRNNITGAIYLRQKFHGPLEILLHLRGFYLPSDKSDHSVHMHGFHVHEFGDFSN

GCTSSSGHFNPSGAAHGGHDAKTRHAGDWGNIACDDAGEVHTNMTDRYCDLYGPRSIIGR

GLVIHETADHLGQAPMGKKKMEGHVGYGNACCTIARIGKVDWDMLLQHKH*REKT*VHF*

ETLGLE*GSSRSSVRS*QHQRN*CVSILMSRRHFLSLIYSPTPFLYPL*NQ*LAFQTYQA

LYMPLQN*KTCFSKPWNTRIAENGIVSKLWIGMKN*RICWKKWVKLFSVELIIRCMFFQA

WNSCLLQTGL*MYTYTRYV*IT*QNGMTCGKLGLNYVIRE*FTRELLQ****V*TKWESN

PYMLLNALHEGWGLYFGIVCSCIKGRWG

>**comp97454_c0_seq14_3** len=1708 path=[5551:0-117 2574:118-279 2736:280-298 2755:299-771 3228:772-788 6868:789-1060 6896:1061-1062 3519:1063-1174 3631:1175-1341 3798:1342-1342 3799:1343-1366 3823:1367-1684 7065:1685-1707]

QRSHDLADRHDKHDKNLR*TYASTT*HEIFPDF*PGAARASTVTPTTTPSSQPPTRGTYL

VTEEKGSRSSPQQPSSQTKMAANDVFVCNQEDLKDGEMKEVDVPGGKVLLTREKGQYYAV

GPKCTHYGASLSNGVLCNGQVRCPWHGACFNIKTGDIEDFPGLNSLPTFEVSVRDSKVYV

KAEEETVKKRFRVKSMVKCSGDNSQTFVLVGGGPATVECAETLRQEGFTGKVVIVTREKH

LPYDRPKLSKAMSIKPEEIALRHADFYQSHDIEIQTEKEVVSVDGSGKSVKFSDGGSLKF

DKLLIATGGKPRTLPIPGLDLQNVCQLRSPEDANYIASAAQGKKVVIIGSSFIGMEVASC

LAEKAENVSVVDLIKVPFQLTLGDRVGAVLQKMHEDKGVKFYFERSVKEFVGVDGKVTEA

ILSDGTKLEADVCVLGIGVVPATDFLKDSGISMTTRGFITVDKSLQTNVADVYAAGDIVE

FPLFMAGNQQANIQHWQMAHKHGHIAALNMLGKTTEVRSVPYFWTVQYGKSVRYTGYGPG

YDDVIVHGDLEAPKFAAFYTKDDPKSWTX

>**comp97821_c1_seq3_6** len=1748 path=[8350:0-18 3383:19-52 10368:53-70 3435:71-221 10435:222-267 3632:268-290 3655:291-293 3658:294-1534 4899:1535-1747]

RTLSRMGELQLFEREEVT*QR*HNALIRLNIVMLCFCVGFPLCRHWSIPNSQGAANVGHS

GHNISNNI*IRMVCRRNRRLLFWTFAVLCVFLIWSGLTHVFRANTEVKRPRRLASYSSMV

SSISRFENDTSSLLVWPNGEDNHPYFLSPVLQFPNDKFRQKTRYNLTGVEKIFTQNKTDE

KLIIIYNMPSWHTFETRYKDSLENCRLQNCRLSGDHKLLPQADAVVFYVLSIDNDTPPPK

PANQMWIFFAHEAPWDFNRWNDRFNWTMTFRYDSDIVCPYFVLLPKPTADVRDFQSITEK

KSKPVMWLVSHCNARSKRDGYVKKLQQFISVDIYGGCGPLKCENSGPNSCNNLQQTTYKF

YLAFENSLCRGYLTEKVFRSYSLDIIPVVRGGASYGNLLPKGTYIDTADFSSPEKLADHL

KYLDSNNTAYAEILRRKSQYTITDPKSTLDSSLCDICVKLHDLERFGNTYHDVNKWWNTD

SCSSATDF*SEDGLTCTPWTP*HSRCIQLK*CGRFIEMEDFA*LQGLDHRQTVDVILNRL

TMQRCVLLRRLVPLQDMDIWGQLTM*LCVLLLRRLVPLQHKRR

>**comp97886_c0_seq1_6** len=2035 path=[1:0-28 30:29-83 85:84-100 102:101-206 7478:207-230 232:231-348 350:349-749 751:750-772 774:773-909 911:910-933 935:934-934 936:935-1866 1868:1867-1868 1870:1869-2034]

RTSCSCLQYSVSLGWLLPRYIDTFRRVTVFSST*RITMSGLIKAKKYDWKDSNLALFGSD

LEKNVKKESAETEAAWQGCGKSVGLKIWRIVKFQVKSWPQADYGKFYDGDSYIILNTYKE

DDSEELQYDVHFWIGQHSTQDEYGTAAYKTVELDTFLSDAPIQHREVQGHESSLFKSYFK

TITVMRGGAETGFRHVKPEEYTPRLLHFSGTRRNVEVREVPLCKSRVNSDDVFILDLGAK

IFQFNGSNSSKDERFKAMQFCQELESERNGRAKAEVIEDESTDQSHEFYKSLTGEDDSDG

PYTAADPSKELYRLSDASGTIQFKLEKKGDISKSDLDSKDVFILDTKSDLFVWIGQDTSC

NEKKKALEFAHNYLMKTDHPFIAVTAIAERQKSASFNTAIAA*CDRPTCRELPVT*LDVA

H*SHDHRHSSTSAVCFDIHCPDSSSFL*ENILKKYCAVDPESEKSIENKEQIANRTNFA*

INAFL*LVYATGCMYIPRF*FLQLSICNITISAIFG*GIRLFSGSITLCSVI*FLERNSK

FIVHLFTPSSPVTEQELCPLAVIISPGFAATYFCH*WWD*NGVCFACINMTNLEIFETN*

ALL*SLIWSNFCTGVP*VYSSGVFYCFFFSPQCRISVMLICLPKSF*YTEQH*KCTTIYY

ILYYTPSDGSVPMISEHR

>**comp97911_c1_seq1_4** len=1696 path=[1:0-71 73:72-102 104:103-278 280:279-320 322:321-324 326:325-345 347:346-455 4378:456-490 492:491-663 665:664-681 683:682-842 844:843-862 864:863-1274 6916:1275-1394 1396:1395-1418 1420:1419-1486 1488:1487-1498 1500:1499-1561 1563:1562-1593 1595:1594-1695]

IKAINKAGLTTIKVIGPVVVHTTYPGFSGVVRVSVGSSHLLTQWADNAFSDPETAYLKYE

VAVGSEDNRTTVFPFTALQSGEGCTLTSPPTCSAVALTDLHWDLHHSHTYLVSVRVTNIV

GLSTVAVSEPYVHDVALPSRGVVEDVIPAGEEALFDMKAFEDTDHQVSTSVLRARWYGFD

TGVSAVTYKVGIGSRPSQTDVKGLVSVGTKLQHEFSGLSLEENKKYFVTVVAAAEGGEIT

ASSDGVTIVRSNVDATDVTIRDGPGCSGSGIEHLAGQANQKVCTNDITYQVSPSTYSAHW

ARSAVSKQRYPDVYWSLQEKFPLSDGLWHDVRDYEHLGTSEKIAVGGVALEPGHTYRAAV

KFCAGKVCTNPTHSDGVTVIHHPPAPGSLALTYEESVGGQARIQVTMTRFRDPDIPVESE

SFDVMDKYEWGLTDDSHSSKLFHNWQKVDAESITTDGDKIRFQITLPHHLTFIKCRRLAV

RGYNQVGLYSTVSADVKDCRAFDPLNIVPAIVIDAIGKAKSSDEGYSISLDQNNRWVHDD

VDYTPYKNILSAVWPTLRHRNYTWA

>**comp98077_c0_seq6_5** len=1876 path=[6589:0-125 2804:126-399 7219:400-400 7220:401-402 3081:403-835 3514:836-859 3538:860-1038 3717:1039-1062 3741:1063-1342 4021:1343-1470 4149:1471-1487 4166:1488-1494 4173:1495-1511 4190:1512-1522 4201:1523-1546 4225:1547-1663 4342:1664-1684 4363:1685-1687 4366:1688-1708 4387:1709-1710 4389:1711-1734 4413:1735-1764 4443:1765-1783 4462:1784-1807 4486:1808-1825 4504:1826-1831 4510:1832-1875]

NLATGARKLPSKPILKDKSKEAPVGAPQVLIEDDKAAMSRSAHEAMSRSVMDISSKPTPD

IMSKSVDMSSMSKSTQDISVEEYKAKLAEKRRQAREKAEKEAEIERQRLEEERLAEEARM

KAEEEEQRRQEEESLRLAEDARRAEEERLRKAIEAEDSRKKEEAERLEQERIAKEEAEQR

AKEESERQEKERQERMKKEEEERQERKKRLEMIMKRVKTDGSESPKTQSPAKSATSSPSK

SGESSTESSAEDGTEETMTTTVTTTGSVTNIVTTISSEDSRPDSGPSSPRGSSPLPSTPS

EKAERQDGGDTPKFRSPLLQQLVENKSSSSSSDRPKFKSPLLQNLLGKNKLGARSNEEKV

EEKKTDSSLVVNGKQGSDTVINSKCDTDTSSVKEQLRYDDASDEDKDVDTSEKSDGRKGS

VEHIEDFPLDVAVKDSTESASVSDLQENKAGVPSATDSGILMDLGTSSSSLAPGPVNGLS

HNGDTKDLVDSSISMKSVESVTAPMNDGMTESGLVTSQNDFEEIIDLSVTNKNIQLNCGQ

ESRGDNSEDLLNFNNVDGASEDNTNRTPLIAFEENSAARQDVTGR*RR**GHPAVRYCKE

GWMVV*CRIMFVVQQPNIKAVSKYVX

>**comp98123_c0_seq1_3** len=5655 path=[12132:0-140 12273:141-153 12286:154-250 556:251-299 606:300-343 650:344-1291 1598:1292-1312 1619:1313-1883 2190:1884-3257 3567:3258-4782 5092:4783-4802 5112:4803-5570 5880:5571-5575 5885:5576-5654]

ARHCACANCPCVEMYKQREDRCVGSCE*VSTDSEYERLAELNLAISLPPTLSGIGIYIHV

DLPEQVYQ*SDVFATLRPIPAIPR*LTDDVYCEARLTHWAVAVNRAGAMEACILFVLAAV

IGQSLCQFQNQPINGQVGNQNGNYLNVNAGNAGNQGNQWNNVPRQQGQQQQNLVKQFNQN

SDQFAQQNLAGAAARPQNNNNNNRNLNNNQQQIHNGVIGAQGIGNNQNQGNIQNVAPNQN

QFNAGQGVQGHKEQDPVVQPGSSNIQARVQNEAHVNPTQAVGNGAQGQQMNQQNNLNQQN

QGSQGNQFIQGNQVNQFNAGDQANLLNRANLPGQGNSQVREEVNVPPVQTAAPPVQQDAG

QGQNDRRADTIAEDDRQRSARLERPVDTGSDNRQNSDNGDRRGLIQNVDDGVYHNNPQPN

NFNNNNGGGGYQNFNGGGVNMNDQGNNYQHQQREIGGIRLTWDWSDFAITFDDYGGAEMK

VRRAPHSTTGEPWPLPQYYVKKEKRVYKLDKDLFTFKVVGETCDIIQDAVERYRTRVLED

AVEDMYDNLQNAPGTNIDDPSLKYDKDMYTKAHVIAKVDIKIRKPCQKFPSVDSDESYDL

VVKKARAFIWANEVWGALRGLETFSHIVWRGLDGEDPDDRAKNANLFVKETVISDYPRFP

HRGILVDSSRHFFFKETIFDILDGMEQNKMNVLHWHIVDDQSFPYQSEVFPELSRKGAFH

PTFVYTQKDIAEIIDYARFRGIRVMPEFDTPGHTYAWGLSRPDLLTQCYQGGSPVKGYLG

PIDPTKNSTYKFLRTLFAEVLDTFKDQYIHLGGDEVPLGCWQSNPDVVNFGMELSKQQSD

NPQSTYNGYYSSQFDVRKVYEYYENRIMKDLREIGKHRKDGVKFVMWQEVMNNDLQLPND

TIIEVWMGDMADVNRAISMGYQVVYATCWYLDHVEYGTKWPKYYQCDPADNTFGYMIDEK

KVLGGEACLWSEYIDNENMMTTLWPRASAVAERLWSAKDVRDLDTAGARLSEHRCRMLNR

GLSVAQISGPDYCLKRGIGRSRDKYRTNSTCTSGRCVKSRDGTFVEMDDIEVRQPHRRMK

VPECPKVPAQGGLLIFLGTALIILIAVGVGVKSTSGRFAQARICKNRTILILFISVLLIY

FMCYTSIWMQVFDFSGSVHKREDSDLTNFKH**NRGNAPCETIRTSTGVFRLADVTSLTS

VSTEST**VDGNQCQWI*C*RAL*WR*A*CSDARCVLVYKWTCEKRGFVAGAFNQWDMSI

SSM*L*CDHQKWTGPLVDDNDCDVDDCDDDDYDDCDDDDSNLHLQTVICNLNV*IL*KGL

FIKGPLIQIFKRNILLM**RVFELYIDSVLS*HARAFRDMNESHVCVSCMSAVWVNVTCL

RVLHVRV*RQTSSHRLNRSEPRVNLRCFHS*IGKHFIFLFACSAMKCGLYIGGAAHLLDR

FLYTGSESAELFPDIQHSKVMLDIYFST*HGAQLSLINSAN*FQYVNDIFT*L*GEKNRD

KKRERSSLREVTPNLVRVEYLHMTFQNQTGL*VLNSL*CTYGAIFIHVCFRTFSSAKFYD

GKLKRHFKD*LKSNQLCKWKRLHM*ALPAFCFFGNTNSKFVVMATGLSCRLNVWCRISCR

ANNSIILAMQAKSFIIFNCIFLNFK*NQNRFGFMCDCSGILS*DLDILLRVMISHRKYFP

VHEVLSLVIYQIFYSSSNDSFIYLRPWRGWILCAFWTNDLHKQMFS**YVLDIELFSVRI

FTLMEYSCLEMNHMLY*ALF*LPASCATFYIS*K*HVFRLFCNIRIVCT*RSLSGPQLHN

SLTHTFRILVSGHY*ISECGV*MRTTFYY*ICFTPHSK**GGLRELHETSMTFQM**HAY

LLIDCWLLAPRLS*LMLS*VSWILX

>**comp98351_c4_seq1_5** len=1352 path=[4561:0-133 4695:134-143 4705:144-399 4961:400-402 4964:403-636 5198:637-677 5239:678-709 5271:710-765 5327:766-792 5354:793-928 5490:929-1351]

SPVV*HAHVTESRRRSVQVSPSTHYCTRTVLATTGKVQQVRMEAVIFVLSIFVSLSGCHV
[truncated: 301,365 more chars]
