# Supplementary material for: In-depth proteomic analyses of Haliotis laevigata (greenlip abalone) nacre and prismatic organic shell matrix
Source: Proteome Sci. 2018 Jun 15;16:11. doi: 10.1186/s12953-018-0139-3 (PMC6003135; doi:10.1186/s12953-018-0139-3)
Supplement: Supplementary file 2 — Table S1. Organic matrix yields. This docx-file shows the organic matrix yields of individual shell fractions as determined by weighing after lyophilisation of acidic extracts. (DOCX 14 kb) [file 12953_2018_139_MOESM2_ESM.docx]

**Table S1: Organic matrix yields**

|  |  |  |
| --- | --- | --- |
| **Method** | **Fraction** | **Yield** |
|  |  |  |
| **A** | Nacre, soluble | 2.2mg/g |
|  | Nacre, insoluble | 15.1mg/g |
|  | Prismatic layer, soluble | 7.1mg/g |
|  | Prismatic layer, insoluble | 2.5mg/g |
| **B** | Nacre, soluble | 2.9mg/g |
|  | Nacre, insoluble | 13.8mg/g |
|  | Prismatic layer, soluble | 3.3mg/g |
|  | Prismatic layer, insoluble | 2.6mg/g |
| **C** | Nacre, soluble | 2.2mg/g |
|  | Nacre, insoluble | 13.7mg/g |
