# Supplementary figures and images for: In-depth proteomic analyses of Haliotis laevigata (greenlip abalone) nacre and prismatic organic shell matrix
Source: Proteome Sci. 2018 Jun 15;16:11. doi: 10.1186/s12953-018-0139-3 (PMC6003135; doi:10.1186/s12953-018-0139-3)

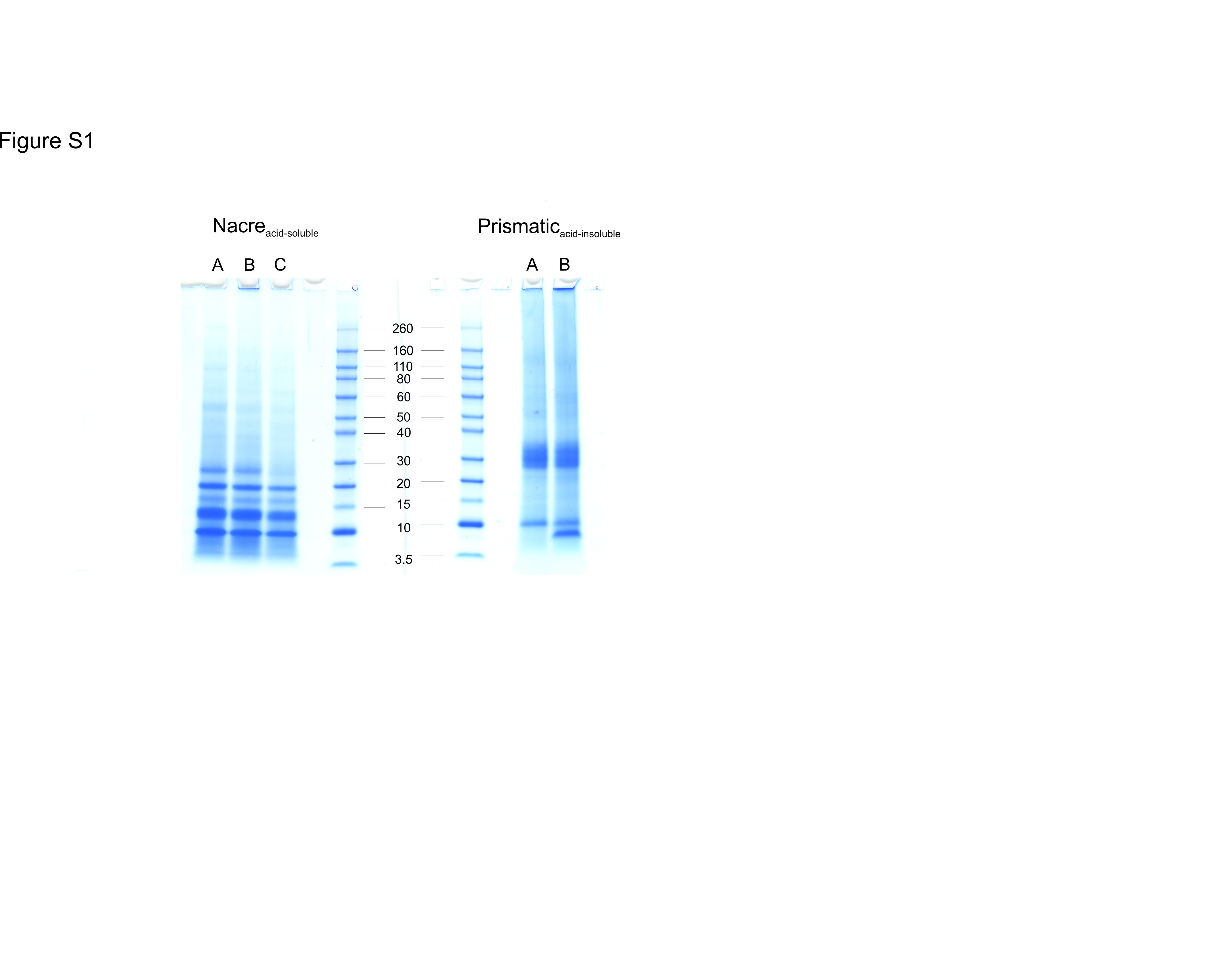

Supplement: Supplementary file 3 — Figure S1. SDS-PAGE of shell organic matrix. This figure in jpg format shows a SDS-PAGE comparison between the nacre acid-soluble fraction obtained with different protocols A, B and C, and comparison of prismatic layer acid-insoluble fractions A and B. Similar amounts of matrix (ca. 200 μg) were applied to each lane. (JPG 1284 kb) [file 12953_2018_139_MOESM3_ESM.jpg]
