# Supplementary material for: In-depth proteomic analyses of Haliotis laevigata (greenlip abalone) nacre and prismatic organic shell matrix
Source: Proteome Sci. 2018 Jun 15;16:11. doi: 10.1186/s12953-018-0139-3 (PMC6003135; doi:10.1186/s12953-018-0139-3)
Supplement: Supplementary file 4 — Table S2. Nacre proteins. docx-file listing all accepted identifications of Haliotis laevigata nacre proteins including most similar database matches, number of identified peptides and abundance in different shell fractions. (DOCX 306 kb) [file 12953_2018_139_MOESM4_ESM.docx]

**Table S2**

***Haliotis laevigata* proteins identified in nacre**

| **Accession ^1^** | **Protein** | **Highest scoring**  **FASTA match**  (maximal allowed e-value of 1.0e-4) | **% iden-tity** | **FASTPe-value** | **Unique and razor peptides** | **Total peptides** | **% of total**  **(iBAQ)** | **Frac-tion^2^** |
| --- | --- | --- | --- | --- | --- | --- | --- | --- |
|  |  |  |  |  |  |  |  |  |
| **H_sp_Tri_60358_c0_g1_i1**  (aa15-329)  **Comp100296_c0_seq2_4**  **etc** | Similar to SCO-spondin; domains: TSP1 (aa14-68,67-125), Fibrinogen_a/b/g_C (aa117-177); SSP (aa1-16); 11.8% G; pI 4.5 | K1PYZ9_CRAGI  (aa1300-1628) | 50.5 | 8.7e-57 | -  -  -  2  2  - | -  -  -  4  4  - | -  -  -  0.001  0.002  - | I_A_  I_B_  I_C_  S_A_  S_B_  S_C_ |
| **H_sp_idb_22145_c0_g1_i1**  (aa1-635)  **Comp101254_c0_seq1_3^3^**  **Comp101254_c0_seq2_3** | Heat shock-inducible protein 70; domain: HSP70_peptide-bd (aa392-548), HSP70_C (aa524-624); pI 5.6; IDR (24.3%; aa1-12, 494-635) shares several peptides with other HSP70-like proteins | C1KC84_HALDV  (aa1-636) | 96.5 | 0e0 | -  2  2  -  2  - | -  3  2  -  7  - | -  0.001  0.002  -  0.001  - | I_A_  I_B_  I_C_  S_A_  S_B_  S_C_ |
| **H_sp_Tri_88331_c0_g1_i1^3^**  (aa67-452)  **Comp101550_c1_seq1_3** | Similar to endoplasmic reticulum resident protein ERp44; domains: Thioredoxin-like_fold (aa48-177,172-268,265-395); pI 6.3; IDR (19.5%; aa365-452) | K1P6B3_CRAGI  (aa7-394) | 63.8 | 6.1e-110 | 4  6  5  7  8  3 | 4  10  9  35  16  8 | 0.006  0.007  0.006  0.008  0.008  0.003 | I_A_  I_B_  I_C_  S_A_  S_B_  S_C_ |
| **H_sp_idb_10164_c0_g1_i1^3^**  (aa2-1062)  **Comp101565_c0_seq1_5** | Similar to hephaestin-like protein; domains. cupredoxin (aa21-208,218-355,364-561,564-709,721-906,918-1064); SSP (aa1-22); pI 5.2; IDR (1.0%; aa132-143) | HEPHL_ACRMI  (aa8-1076) | 45.7 | 0e0 | -  5  3  -  -  - | -  6  3  -  -  - | -  0.002  0.001  -  -  - | I_A_  I_B_  I_C_  S_A_  S_B_  S_C_ |
| **Comp101638_c0_seq5_5^3^**  (aa4-358)  **Comp101638_c0_seq1_5**  **Comp101638_c0_seq4_5**  H_sp_idb_21744_c0_g1_i1 | Uncharacterized; domains: ZP (aa3-291); TM (aa320-342,357-379,392-414,429-461,511-533); pI 8.4; IDR (5.9%; aa1-22,288-297) | C3Z6R0_BRAFL  (aa187-533) | 28.9 | 2.1e-26 | 5  8  6  4  5  2 | 14  29  21  57  53  4 | 0.036  0.107  0.056  0.010  0.009  0.001 | I_A_  I_B_  I_C_  S_A_  S_B_  S_C_ |
| **Comp101644_c1_seq1_1^3^**  (aa48-430) | Similar to thioester-containing protein(-G); domain: A2M_N (aa171-259); TM (aa36-58); pI 9.0; share almost all peptides | D5FT55_9BIVA  (aa2-363) | 38.9 | 2.5e-38 | -  1  5  6  1  1 | -  2  19  21  12  17 | -  0.001  0.016  0.002  0.001  0.004 | I_A_  I_B_  I_C_  S_A_  S_B_  S_C_ |
| **Comp101644_c0_seq2_4^3^**  (aa35-1158) | Similar to thioester-containing protein/CD109-like; domains: A2M_N_2 (aa135-269), Macroglobin_a2 (aa388-475), A2M_comp (609-950), A-macroglobulin_rcpt-bd (aa1019-1137); pI 6.1; share almost all peptides; IDR (5.2%; aa1-71,779-794) | D4QA02_9CNID  (aa375-1475) | 34.3 | 3.2e-89 | -  -  22  -  -  - | -  -  83  -  -  - | -  -  0.018  -  -  - | I_A_  I_B_  I_C_  S_A_  S_B_  S_C_ |
| **H_sp_CLC_1485_c0_g1_i1^3^**  (aa3-1513) | Similar to thioester-containing protein(-E); SSP (aa1-17); domains: A2M_N (aa126-207), A2M_N_2 (aa569-630), A2M (aa749-836), A2M_compl (aa966-1311), A-macroglobulin_rcpt-bd (aa1380-1498); IDR (3.6%; aa409-463); pI 5.5 | D5FT53_9BIVA  (aa2-1461) | 35.2 | 7.1e-108 | 6  11  -  -  9  4 | 9  23  -  -  15  7 | 0.003  0.005  -  -  <0.001  <0.001 | I_A_  I_B_  I_C_  S_A_  S_B_  S_C_ |
| **Comp101858_c3_seq18_4^3^**  (aa1-523)  H_sp_idb_10046_c0_g1_i1 | Pyruvate kinase; domains: Pyrv_Knase_brl (aa7-386), Pyrv_Knase_C (aa364-523); pI 8.1 | A1L3K2_XENLA  (aa9-531) | 100.0 | 0e0 | 2  7  8  11  13  - | 2  11  14  28  37  - | 0.002  0.005  0.007  0.003  0.029  - | I_A_  I_B_  I_C_  S_A_  S_B_  S_C_ |
| **H_sp_CLC_2877_c0_g1_i1^3^**  (aa16-850)  **H_sp_idb_3129_c0_g1_i1**  **Comp102281_c0_seq4_1**  **etc** | Similar to dystroglycan; domains: Cadherin-like (aa70-160,420-522), Alpha-dystroglycan_domain_2 (aa182-303), SEA_DG (aa530-638); TM (aa12-34,707-729); pI 6.0; IDR (22.6%; aa302-323,640-706,748-850) | V4B4U7_LOTGI  Lotgidraft_224800  (aa1-831) | 45.1 | 3.0e-73 | 3  2  -  4  3  2 | 7  5  -  14  12  10 | 0.013  0.008  -  0.002  0.002  0.002 | I_A_  I_B_  I_C_  S_A_  S_B_  S_C_ |
| **H_sp_Tri_129753_c0_g1_i1^3^**  (aa1-144)  **Comp102930_c0_seq1_5** | Uncharacterized; domain: UspA (aa1-147); 9.5% G, 10.8% V ; pI 7.8 | A0A0B6ZQD9_  9EUPU  (aa1-145) | 45.5 | 9.6e-23 | 2  3  3  5  4  3 | 3  7  12  25  22  5 | 0.006  0.011  0.033  0.026  0.015  0.004 | I_A_  I_B_  I_C_  S_A_  S_B_  S_C_ |
| **H_sp_idb_17123_c0_g1_i1**  (aa6-334)  **Comp103017_c0_seq2_6**  **etc** | Similar to calumenin; domains: EFh_pair (aa83-142,161-330); SSP (aa1-33)/TM (aa13-35); 14.4% D, 12.0% E, 9.6% K; pI 4.6; IDR (37.4%; aa31-69,234-279,291-394); if SSP:  16.1% D, 13.4% E, 10.4% K; IDR: 62.4% | A0A0B7A268_  9EUPU  (aa29-357) | 66.5 | 1.5e-78 | 3  4  4  3  2  - | 3  7  6  5  2  - | 0.007  0.012  0.004  0.001  <0.001  - | I_A_  I_B_  I_C_  S_A_  S_B_  S_C_ |
| **Comp103384_c0_seq2_4^3^**  (aa31-231) | Uncharacterized; domain: ependymin_fam (aa106-229); TM (aa22-40,255-277,314-336); IDR (2.8%; aa1-11); pI 8.7; shares most peptides with Tri_16732 | V4BP88_LOTGI**^4^**  Lotgidraft_233583  (aa10-215) | 33.5 | 1.3e-22 | -  8  1  2  2  1 | -  21  1  11  14  3 | -  0.027  0.005  0.003  0.015  <0.001 | I_A_  I_B_  I_C_  S_A_  S_B_  S_C_ |
| **H_sp_Tri_16732_c0_g1_i1^3^**  (aa9-209) | Uncharacterized; shares most peptides with comp103384_c0_seq2_4; domain: ependymin (aa83-207); SSP (aa1-22); pI 9.0 | V4BP88_LOTGI**^4^**  Lotgidraft_233583  (aa10-215) | 33.3 | 4.4e-21 | -  1  1  10  13  9 | -  2  2  37  41  20 | -  <0.001  <0.001  0.016  0.081  0.010 | I_A_  I_B_  I_C_  S_A_  S_B_  S_C_ |
| **H_sp_idb_4412_c0_g1_i1^3^**  (aa31-229) | Uncharacterized; domain: ependymin (aa88-212); SSP (aa4-26)/TM (aa4-26); TM (aa157-179); pI 9.2; shares 1 peptide with Tri_16732; if SSP: 9.3 | V4BP88_LOTGI**^4^**  Lotgidraft_233583  (aa32-233) | 30.2 | 3.6e-19 | -  -  -  1  -  - | -  -  -  1  -  - | -  -  -  <0.001  -  - | I_A_  I_B_  I_C_  S_A_  S_B_  S_C_ |
| **H_sp_Tri_131427_c0_g1_i1^3^**  (aa1-376)  **Comp103470_c1_seq20_6**  **etc** | Actin; pI 5.2; shares most peptides with other actins | Q6U1K1_HALDH  (aa1-376) | 99.7 | 1.4e-159 | 9  17  14  11  13  8 | 28  75  76  137  130  65 | 0.099  0.258  0.344  0.054  0.141  0.027 | I_A_  I_B_  I_C_  S_A_  S_B_  S_C_ |
| **H_sp_Tri_56398_c0_g1_i1**  (aa105-359)  **Comp103479_c0_seq1_1**  **Comp103479_c0_seq2_1** | Uncharacterized; domain: IG (aa23-134); SSP (aa1-19); pI 5.0 | V4CHE6_LOTGI  Lotgidraft_238272  (aa18-267) | 30.8 | 2.2e-16 | -  5  4  3  4  - | -  12  10  4  11  - | -  0.012  0.005  0.001  0.001  - | I_A_  I_B_  I_C_  S_A_  S_B_  S_C_ |
| **H_sp_Tri_23898_c0_g1_i1^3^**  (aa21-190)  **Comp103559_c0_seq2_2**  **etc** | Similar to leukocyte cell-derived chemotaxin 1-like protein; domain: BRICHOS (aa75-162); SSP (aa1-30); 11.0% V; pI 5.2; see also Tri_53798 and Comp94109_c0_seq16_5 | D5FW85_HALDI  (aa7-173) | 30.1 | 3.6e-7 | -  2  -  -  4  - | -  6  -  -  7  - | -  0.005  -  -  0.002  - | I_A_  I_B_  I_C_  S_A_  S_B_  S_C_ |
| **Comp103717_c1_seq2_5^3^**  (aa58-508)  **Comp103717_c1_seq11_6** | Uncharacterized/similar to α-tubulin; pI 5.4; domains: Tubulin_FtsZ_GTPase (aa56-322), Tub_FtsZ_C (aa303-494); see also Comp88085_c0_seq2_2; IDR (7.4%; aa98-101,491-524) | V3ZLS0_LOTGI  Lotgidraft_236629  (aa1-451) | 97.8 | 6.6e-200 | -  7  7  11  -  - | -  15  21  36  -  - | -  0.014  0.024  0.014  -  - | I_A_  I_B_  I_C_  S_A_  S_B_  S_C_ |
| **H_sp_idb_14486_c0_g1_i1**  (aa1-347)  **Comp103784_c0_seq4_6**  **Comp103784_c0_seq2_6** | Uncharacterized/similar to peptidyl-glycine alpha-amidating monooxygenase; domains: PHM/PNGase_F (aa22-171,176-319); SSP (aa1-18); pI 5.1; IDR (17.3%; aa328-393) | R7UVI8_CAPTE  (aa9-349) | 56.5 | 1.1e-88 | -  -  -  6  4  3 | -  -  -  22  12  3 | -  -  -  0.004  0.005  0.002 | I_A_  I_B_  I_C_  S_A_  S_B_  S_C_ |
| **Comp103921_c1_seq6_3**  (aa1-298) | Malate dehydrogenase, mitochondrial; domains: Lactate/malate_DH_N (aa2-128), Lactate/malate_DH_C (aa129-296); 10.1% L; pI 7.5 | MDHM_MOUSE  (aa41-338) | 100.0 | 2.1e-122 | -  -  2  -  3  - | -  -  2  -  3  - | -  -  0.001  -  0.001  - | I_A_  I_B_  I_C_  S_A_  S_B_  S_C_ |
| **H_sp_idb_7892_c0_g1_i1^3^**  (aa12-365)  **Comp104004_c0_seq4_5**  **Comp104004_c0_seq2_5** | Uncharacterized; domain: Renin_receptor-like (aa9-365); SSP (aa1-29), TM (aa324-346); 11.9% L; pI 6.4; IDR (8.3%; aa286-314) | V4CR93_LOTGI  Lotgidraft_211795  (aa12-365) | 61.1 | 5.2e-86 | 5  2  2  10  7  7 | 13  2  2  118  52  32 | 0.015  0.002  0.001  0.045  0.023  0.022 | I_A_  I_B_  I_C_  S_A_  S_B_  S_C_ |
| **H_sp_idb_5674_c0_g1_i1^3^**  (aa131-626)  **Comp104092_c0_seq1_6** | Polypeptide N-acetylgalactosaminyl-transferase; domains: Glyco_trans_2-like (aa196-380), Ricin_B_lectin (aa500-624); TM (aa12-34); pI 7.6; not all peptides in alignment! IDR (21.7%; aa32-160,627-632) | V3ZHH4_LOTGI**^4^**  Lotgidraft_123129  (aa1-496) | 83.5 | 2.0e-198 | 3  2  5  -  -  - | 4  6  10  -  -  - | 0.004  0.003  0.003  -  -  - | I_A_  I_B_  I_C_  S_A_  S_B_  S_C_ |
| **H_sp_CLC_4272_c0_g1_i1**  (aa24-691)  **Comp104530_c1_seq1_2** | Uncharacterized; domains: FAS1 (aa25-179,190-331,332-478,539-679); SSP (aa1-18) | V3YVD0_LOTGI  Lotgidraft_237218  (aa23-693) | 50.1 | 1.4e-149 | -  15  14  -  2  - | -  49  44  -  2  - | -  0.064  0.032  -  <0.001  - | I_A_  I_B_  I_C_  S_A_  S_B_  S_C_ |
| **Comp104605_c0_seq2_6**  (aa9-525)  **Comp104605_c0_seq1_5** | Similar to nephrin; domains: IG_like (aa21-115,126-243,232-335,328-443), FN3 (aa430-543); SSP (aa1-27); pI 8.2; shares several peptides with Tri_113162; IDR (2.5%; aa759-778) | E2BTY3_HARSA  (aa425-915) | 22.7 | 1.9e-11 | -  5  -  -  -  - | -  10  -  -  -  - | -  0.007  -  -  -  - | I_A_  I_B_  I_C_  S_A_  S_B_  S_C_ |
| **H_sp_Tri_113162_c0_g1_i1**  (aa26-518)  **H_sp_idb_44566_c0_g1_i1** | Similar to nephrin; domains: IG (aa26-98,144-219,226-329,322-432), FN3 (aa431-516)¸ SSP (aa1-13); pI 8.0; shares several peptides with comp104605_c0_seq2_6; IDR (2.7%; aa468-482) | E2BTY3_HARSA  (aa425-915) | 23.2 | 5.6e-11 | -  1  -  -  3  - | -  3  -  -  5  - | -  0.002  -  -  0.001  - | I_A_  I_B_  I_C_  S_A_  S_B_  S_C_ |
| **H_sp_idb_6726_c0_g1_i1**  (aa1-1615)  **Comp104774_c0_seq13_1** | Clathrin heavy chain; domains: Clathrin_H-chain_propeller_N (aa5-334), ARM-type_fold (aa337-492,892-1051,1190-1521); 10.5% L; pI 5.5; 1 peptide also in comp409151_c0_seq1_5; IDR (1.5%; aa1618-1640) | V4AE67_LOTGI  Lotgidraft_233411  (aa1-1610) | 90.5 | 0e0 | -  2  4  -  -  - | -  2  4  -  -  - | -  <0.001  <0.001  -  -  - | I_A_  I_B_  I_C_  S_A_  S_B_  S_C_ |
| **Comp409151_c0_seq1_5**  (aa1-75) | Clathrin heavy chain 1 (Fragment); domain: ARM-type_fold (aa2-75); 10.5% E, 13.2% L, 10.5% V; pI 4.4; 1 peptide also in idb_6726 | H1A1A6_TAEGU  (aa42-116) | 100.0 | 3.5e-28 | -  -  -  2  2  - | -  -  -  2  2  - | -  -  -  0.001  <0.001  - | I_A_  I_B_  I_C_  S_A_  S_B_  S_C_ |
| **Comp105740_c0_seq2_6**  (aa10-445)  **Comp105740_c0_seq1_6**  H_sp_Tri_41929_c0_g1_i1 | Uncharacterized/similar to V-type proton ATPase subunit S1; domain: ATPase_V1-cplx_s1su (aa1-449); SSP (aa1-22); TM (aa405-427,499-521,588-610); pI 8.0 | A0A0B7ANW2_  9EUPU  (aa10-449) | 37.3 | 1.1e-53 | -  -  -  10  7  6 | -  -  -  44  36  19 | -  -  -  0.008  0.007  0.006 | I_A_  I_B_  I_C_  S_A_  S_B_  S_C_ |
| **H_sp_idb_30241_c0_g1_i1^3^**  **Comp106275_c0_seq2_4** | Uncharacterized; SSP (aa1-20); pI 6.3 |  |  |  | -  -  -  -  2  2 | -  -  -  -  26  5 | -  -  -  -  0.057  0.029 | I_A_  I_B_  I_C_  S_A_  S_B_  S_C_ |
| **H_sp_idb_14366_c0_g1_i1^3^**  (aa2**-**934)  **Comp106461_c0_seq3_2**  **etc** | Uncharacterized; domains: EMI (aa22-97), multiple EGF-like; SSP (aa1-16), TM (aa809-831); 13.8% C, 11.7% G; pI 6.7; IDR (8.5%; aa782-813,850-900) | T1IUZ7_STRMM  (aa6-934) | 38.3 | 1.8e-116 | -  -  -  2  -  2 | -  -  -  3  -  3 | -  -  -  <0.001  -  <0.001 | I_A_  I_B_  I_C_  S_A_  S_B_  S_C_ |
| **H_sp_idb_40604_c0_g1_i1**  (aa1-185)  **Comp106524_c3_seq3_4**  **Comp106524_c3_seq7_4** | Similar to latent transforming growth factor beta binding protein; SSP (aa1-25); 10.0% N, 12.5% C, 10.6% G, 10.6% S; pI 8.1 | E4W3F5_HALDV  (aa1-186) | 78.6 | 1.5e-61 | -  -  -  3  4  - | -  -  -  11  12  - | -  -  -  0.020  0.049  - | I_A_  I_B_  I_C_  S_A_  S_B_  S_C_ |
| **H_sp_idb_2214_c0_g1_i1^3^**  (aa1-430)  **Comp106543_c0_seq12_5**  **etc** | Uncharacterized; domain: HSP70_family (aa33-410); SSP/TM (aa1-22/5-22); 11.0% L, 9.7% V; pI 7.0; if SSP: 11.0% L, 9.8% V; pI 7.2 | V4AQB2_LOTGI  Lotgidraft_159745  (aa1-430) | 68.1 | 5.0e-117 | 5  5  4  8  8  6 | 11  13  10  62  72  8 | 0.021  0.021  0.006  0.017  0.025  0.003 | I_A_  I_B_  I_C_  S_A_  S_B_  S_C_ |
| **H_sp_CLC_2126_c0_g1_i1^3^**  (aa3-98)  **Comp106756_c0_seq2_6**  **etc** | Similar to cystatin-B; domain: cystatin (aa2-98); 12.0% S, 10.0% T; pI 5.7; IDR (37.0%; aa1-14,78-100) | M4H503_HALDI  (aa1-98) | 66.3 | 4.7e-28 | -  -  -  4  3  - | -  -  -  42  18  - | -  -  -  0.137  0.052  - | I_A_  I_B_  I_C_  S_A_  S_B_  S_C_ |
| **Comp106862_c0_seq1_3^3^**  (aa51-1458)  **Comp106862_c0_seq2_3**  H_sp_idb_22196_c0_g1_i1 | Similar to thioester-containing protein; domains: A2M_N (aa49-138), A2M_N2 (368-498), A2M (626-717), A2M_comp (845-1148), A2M_rec_bd (1239-1349); multiple TM; shares most of its peptides with idb_9842; IDR (2.8%; aa1-30,624-640,1158-1175) | A0A0E4B804  _SCOSU  (aa24-1428) | 30.4 | 5.1e-132 | 1  5  2  1  3  - | 1  7  2  2  5  - | 0.001  0.002  <0.001  <0.001  <0.001  - | I_A_  I_B_  I_C_  S_A_  S_B_  S_C_ |
| **Comp106997_c0_seq1_2^3^**  (aa76-1443)  **Comp106997_c0_seq3_1** | Similar to protocadherin Fat 4; domains: multiple cadherin; 11.2% T, 9.2% V; pI 4.4; IDR (3.0%; aa1-43) | K1PTY5_CRAGI  (aa36-1403) | 39.4 | 4.7e-101 | -  7  -  -  -  - | -  12  -  -  -  - | -  0.005  -  -  -  - | I_A_  I_B_  I_C_  S_A_  S_B_  S_C_ |
| **H_sp_idb_1321_c0_g1_i1^3^**  (aa10-2982)  Comp107313_c0_seq1_1 | Similar to protocadherin Fat 4; domains: multiple cadherin; 11.3% T, 9.5% V; pI 4.5 | K1PTY5_CRAGI  (aa1284-4267) | 43.1 | 9.4e-215 | -  11  2  -  -  - | -  23  2  -  -  - | -  0.004  <0.001  -  -  - | I_A_  I_B_  I_C_  S_A_  S_B_  S_C_ |
| **Comp107313_c0_seq1_3^3^**  (aa2231-7451)  H_sp_Tri_26792_c0_g1_i1 | Similar to protocadherin Fat 4; domains: multiple cadherin-like; multiple TM; 9.5% T; pI 6.1 | K1QB61_CRAGI  (aa1505-6530) | 38.8 | 4.3e-165 | 3  32  3  2  -  - | 3  55  3  2  -  - | <0.001  0.003  <0.001  <0.001  -  - | I_A_  I_B_  I_C_  S_A_  S_B_  S_C_ |
| **Comp107245_c0_seq3_6**  (aa93-2720)  **Comp107245_c0_seq1_5^3^**  **Comp107245_c0_seq2_5**  H_sp_Tri_34963_c0_g1_i1 | Uncharacterized/reeler_Egf_Cub_1; domains: multiple EGF_like, TY, WAP, Kunitz, SEA (aa966-1071); 9.3% C; pI 7.9; IDR (7.4%; aa1049-1995,2039-2053,2233-2264,2575-2591,2635-2672,2723-2783) | W4ZEF6_STRPU  (aa1945-4472) | 23.2 | 6.4e-30 | -  -  2  -  2  2 | -  -  2  -  4  2 | -  -  <0.001  -  0.001  <0.001 | I_A_  I_B_  I_C_  S_A_  S_B_  S_C_ |
| **H_sp_Tri_109898_c0_g1_i1^3^**  (aa1-176)  **Comp85993_c0_seq3_1**  **etc** | Ubiquitin/polyubiquitin-B; domains: Ubiquitin (aa1-48,47-124,124-176); 9.7% I, 11.4% L, 9.7% K; pI 6.2; IDR (35.8%; aa1-28,141-176) | J3QKN0_HUMAN  (aa29-204) | 100.0 | 2-0e-67 | 2  2  3  4  3  2 | 10  12  10  62  63  35 | 0.046  0.080  0.052  0.067  0.120  0.029 | I_A_  I_B_  I_C_  S_A_  S_B_  S_C_ |
| **Comp108991_c0_seq1_4**  (aa12-175) | Peptidyl-prolyl cis-trans isomerase; domain: Cyclophilin-like (aa3-177); 10.1% G, 9.7% F; pI 8.9; IDR (6.3%; aa1-7,188-195) | Q5SVY2_MOUSE  (aa1-164) | 100.0 | 2.6e-73 | -  4  5  4  4  - | -  7  8  8  8  - | -  0.012  0.016  0.004  0.004  - | I_A_  I_B_  I_C_  S_A_  S_B_  S_C_ |
| **Comp109153_c0_seq1_5**  (aa1-159)  H_sp_idb_67060_c0_g1_i1 | Ribosomal protein S16; domain: Ribosomal_S5_D2-typ_fold_subgr (18-159); 11.1% L, 10.0% K; pI 10.2; IDR (9.9%; aa1-6,148-152,175-180) | Q5CZY9_MOUSE  (aa14-172) | 100.0 | 1.0e-66 | -  -  2  -  3  - | -  -  2  -  5  - | -  -  0.002  -  0.001  - | I_A_  I_B_  I_C_  S_A_  S_B_  S_C_ |
| **Comp109220_c0_seq1_2**  (aa2-128) | 60S acidic ribosomal protein P2; 17.0% A, 10.6% S; pI 4.6; IDP (55.3%; aa1-20,84-141) | L8HUF0_9CETA  (aa6-132) | 90.6 | 4.5e-34 | -  -  2  -  3  - | -  -  3  -  7  - | -  -  0.010  -  0.005  - | I_A_  I_B_  I_C_  S_A_  S_B_  S_C_ |
| **Comp109254_c0_seq1_3^3^**  (aa10-348) | Glyceraldehyde-3-phosphate dehydrogenase; domains: GlycerAld_3-P_DH_NAD(P)-bd (aa17-165), GlycerAld_3-P_DH_cat (aa170-327); pI 8.7; IDR (15.1%; aa350-410) | A0A0A0MQF6_  MOUSE  (aa21-359) | 100.0 | 5.8e-143 | 3  5  5  5  5  2 | 6  15  17  41  35  16 | 0.040  0.033  0.037  0.016  0.022  0.007 | I_A_  I_B_  I_C_  S_A_  S_B_  S_C_ |
| **Comp109271_c0_seq1_3^3^**  (aa52-433) | β-tubulin; domains: Tubulin_FtsZ_GTPase (aa54-312), Tub_FtsZ_C (aa297-433); pI 7.2; shares most peptides with idb_19738 and idb_19739 | G9KVY5_MOUSE  (aa1-382) | 99.5 | 9.6e-172 | -  1  4  2  3  3 | -  2  4  4  4  3 | -  0.001  0.002  0.002  0.001  0.001 | I_A_  I_B_  I_C_  S_A_  S_B_  S_C_ |
| **H_sp_idb_19738_c0_g1_i1^3^**  (aa1-447)  G9K382_HALDV  Comp83487_c0_seq4_4  Comp68763_c0_seq1_6 | β-tubulin; domains: Tubulin_FtsZ_GTPase (aa1-254), Tub_FtsZ_C (aa244-429); pI 4.7; IDR (7.4%; aa415-447); shares most peptides with comp109271_c0_seq1  _3, idb_19739 and TBB_HALDI | W4XHC0_STRPU  (aa1-447) | 98.9 | 1.7e-187 | -  9  13  14  1  - | -  22  34  34  1  - | -  0.023  0.032  0.010  <0.001  - | I_A_  I_B_  I_C_  S_A_  S_B_  S_C_ |
| **H_sp_idb_19739_c0_g1_i1**  (aa1-446)  **H_sp_idb_19741_c0_g1_i1**  Comp89963_c0_seq5_4 | β-tubulin; domains: Tubulin_FtsZ_GTPase (aa1-259), Tub_FtsZ_C (aa244-429); pI 4.8; IDR (7.2%; aa415-446); shares most peptides with idb_19738 and comp109271_c0_seq1_3 | W5M384_LEPOC  (aa1-446) | 98.4 | 1.5e-192 | -  -  -  2  -  - | -  -  -  8  -  - | -  -  -  0.001  -  - | I_A_  I_B_  I_C_  S_A_  S_B_  S_C_ |
| **TBB_HALDI** | β-tubulin, fragment; domains: Tubulin_FtsZ_GTPase (aa1-189), Tub_FtsZ_C (aa170-341);pI 5.9; shares most peptides with comp109271_c0_seq1_3 und idb_19738 |  |  |  | -  -  -  -  9  3 | -  -  -  -  22  3 | -  -  -  -  0.003  <0.001 | I_A_  I_B_  I_C_  S_A_  S_B_  S_C_ |
| **Comp109277_c0_seq1_4**  (aa32-495) | Heterogeneous nuclear ribonucleoprotein K; domains: ROK_N (aa32-74), KH_dom_type_1 (aa58-145,168-301,410-492); pI 8.6; IDR (28.1%; aa1-82,149-164,258-416) | F6WZ20_MACMU  (aa1-464) | 100.0 | 2.1e-131 | -  -  3  -  4  - | -  -  3  -  6  - | -  -  0.001  -  <0.001  - | I_A_  I_B_  I_C_  S_A_  S_B_  S_C_ |
| **Comp109621_c0_seq1_5**  (aa7-220)  B8XW76_HALDV  H_sp_CLC_853_c0_g1_i1 | Ribosomal protein L10; domain: Ribosomal_L10e/L16 (aa10-179); 10.1% R, 10.1% K; pI 10.1; IDR (28.1%; aa1-82,149-164,258-416) | RL10_HUMAN  (aa1-214) | 100.0 | 3.5e-98 | -  -  2  -  2  - | -  -  2  -  3  - | -  -  0.001  -  0.001  - | I_A_  I_B_  I_C_  S_A_  S_B_  S_C_ |
| **Comp109641_c0_seq1_1**  (aa11-202)  E6Y2Z1_HALDV  H_sp_idb_37947_c0_g1_i1 | GTP-binding nuclear protein Ran; domain: Small_GTP-bd (aa32-195); pI 9.6; IDR (12.3%; aa1-25) | G7PJG8_MACFA  (aa5-197) | 94.8 | 5.0e-69 | -  -  2  -  2  - | -  -  2  -  2  - | -  -  0.001  -  <0.001  - | I_A_  I_B_  I_C_  S_A_  S_B_  S_C_ |
| **Comp111062_c0_seq1_3**  (aa18-570)  H_sp_Tri_97542_c0_g1_i1 | ATP synthase subunit alpha, mitochondrial; domains: ATP_synth_asu-like (aa63-142), ATP_synth_F1_a (aa154-435), ATP_synth_asu_C (aa439-564); pI 9.3; IDR (2.5%; aa1-11,729-735) | ATPA_MOUSE  (aa1-553) | 100.0 | 2.4e-202 | -  3  7  2  4  - | -  5  7  2  4  - | -  0.001  0.003  <0.001  <0.001  - | I_A_  I_B_  I_C_  S_A_  S_B_  S_C_ |
| **Comp111239_c0_seq1_1**  (aa1-282) | Similar to 14-3-3 protein epsilon, domain: 14-3-3_domain (aa28-272); pI 9.3; 11.8% L; IDR (11.8%; aa1-37,261-302); shares 1 peptide with other 14-3-3 proteins | K9IRD6_DESRO  (aa6-287) | 99.6 | 5.3e-115 | -  -  4  4  6  - | -  -  5  9  7  - | -  -  0.002  0.001  <0.001  - | I_A_  I_B_  I_C_  S_A_  S_B_  S_C_ |
| **H_sp_CLC_3369_c0_g1_i1^3^**  (aa35-291)  **Comp94743_c0_seq3_1** | Uncharacterized/similar to 14-3-3 epsilon; domain: 14-3-3 (aa36-280); 9.3% A, 11.0% E, 10.0% L, 9.6% S; pI 4.7¸ IDR (21.7%; aa1-9,22-40,102-110,265-291) | K7J834_NASVI  (aa2-256) | 83.3 | 2.6e-87 | -  -  1  -  -  - | -  -  2  -  -  - | -  -  0.003  -  -  - | I_A_  I_B_  I_C_  S_A_  S_B_  S_C_ |
| **Comp112217_c0_seq1_5**  (aa1-288) | ATP synthase subunit beta, mitochondrial; domains: P-loop_ NTPase (aa1-169), ATPase_ F1/V1_b/a_C (aa169-280); pI 4.9; IDR (11.2%; aa278-305); shares 2 peptides with comp21825_c0_seq1_2 | ATPB_MOUSE  (aa242-529) | 100.0 | 1.9e-107 | -  2  3  2  4  - | -  4  4  3  4  - | -  0.003  0.003  <0.001  <0.001  - | I_A_  I_B_  I_C_  S_A_  S_B_  S_C_ |
| **Comp21825_c0_seq1_2**  (aa1-226) | ATP synthase subunit beta, mitochondrial; domains: ATPase_F1/V1/A1_a/bsu_N (aa56-130), P-loop_NTPase (aa132-224); 14.2% A, 11.5% G, 10.2% L, 10.2% V; pI 6.5; IDR (14.2%; aa1-5,24-50); shares 2 peptides with comp112217_c0_seq1_5 | ATPB_MOUSE  (aa2-227) | 100.0 | 1.3e-77 | -  1  5  -  3  - | -  2  7  -  4  - | -  0.002  0.004  -  <0.001  - | I_A_  I_B_  I_C_  S_A_  S_B_  S_C_ |
| **H_sp_idb_10968_c0_g1_i1^3^**  (aa1-117)  **Comp112534_c0_seq1_2** | Similar to tyramine beta-hydroxylase/temptin; SSP (aa1-19); 11.6% R, 11.6% G; pI 9.8; IDR (22.3%; aa102-127) | K1RV04_CRAGI  (aa1-117) | 49.6 | 8.3e-22 | 5  4  5  10  9  7 | 19  15  19  121  90  72 | 0.092  0.060  0.054  0.258  0.167  0.222 | I_A_  I_B_  I_C_  S_A_  S_B_  S_C_ |
| **Comp114698_c0_seq1_3^3^**  (aa1-121) | Histone 2B; 9.5% A, 13.5% K, 10.1% S; pI 10.5; domain: Histone_H2A/H2B/H3 (aa8-121); IDR (46.0%; aa1-38,118-146) | H2B_PATGR  (aa2-122) | 97.5 | 1.3e-44 | -  -  -  2  2  - | -  -  -  10  12  - | -  -  -  0.009  0.009  - | I_A_  I_B_  I_C_  S_A_  S_B_  S_C_ |
| **Comp118163_c0_seq1_4**  (aa20-239)  H_sp_idb_26847_c0_g1_i1 | Phosphoglycerate kinase (1); domains: Phosphoglycerate_kinase_N (aa14-91,92-225,226-239); 10.5% A, 10.9% L, 10.5% K; pI 9.5; IDR (6.7%; aa1-16) | PGK1_MOUSE  (aa1-220) | 100.0 | 3.0e-89 | 2  2  4  4  4  - | 2  4  5  19  7  - | 0.004  0.004  0.004  0.004  <0.001  - | I_A_  I_B_  I_C_  S_A_  S_B_  S_C_ |
| **Comp121020_c0_seq1_1**  (aa1-179) | Phosphoglycerate kinase (1); domain: Phosphoglycerate_kinase_N (aa1-166); 10.4% L, 10.7% S; pI 6.2 | PGK1_MOUSE  (aa239-417) | 100.0 | 6.5e-76 | 1  1  1  3  2  - | 1  2  4  9  9  - | 0.001  0.004  0.004  0.003  0.006  - | I_A_  I_B_  I_C_  S_A_  S_B_  S_C_ |
| **Comp128817_c0_seq1_3^3^**  **H_sp_idb_42198_c0_g1_i1** | Uncharacterized; domain: hirudin_antistatin (aa219-257); 12% P, 9.3% S; pI 9.6; IDR (56.6%; aa1-84,190-241) |  |  |  | -  -  -  4  3  6 | -  -  -  10  25  25 | -  -  -  0.003  0.009  0.017 | I_A_  I_B_  I_C_  S_A_  S_B_  S_C_ |
| **Comp133740_c0_seq1_1^3^**  (aa1-478)  A7L6B1_HALAI  H_sp_CLC_1079_c0_g1_i1 | Catenin beta-1; domain: ARM-type_fold (aa1-480); 14.0% L; pI 9.1 | A0A087QH36_  APTFO  (aa129-606) | 100.0 | 2.2e-209 | 3  3  3  3  3  2 | 3  5  8  20  28  12 | 0.002  0.006  0.004  0.001  0.006  0.002 | I_A_  I_B_  I_C_  S_A_  S_B_  S_C_ |
| **Comp135434_c0_seq1_4^3^**  (aa1-105) | Histone H4; domain: TAF_TATA-bd (aa1-104); 12.6% R, 15.3% G, 10.8% K; pI 11.2; IDR (26.1%; aa1-29) | A0A023FTG4_  9ACAR  (aa5-109) | 99.0 | 1.4e-39 | 4  6  7  5  7  5 | 9  22  24  42  27  14 | 0.100  0.106  0.351  0.049  0.121  0.045 | I_A_  I_B_  I_C_  S_A_  S_B_  S_C_ |
| **Comp136401_c0_seq1_5^3^**  (aa42-696) | 78 kDa glucose-regulated protein; domains: HSP70_peptide-bd (aa452-583), HSP70_C (aa584-686); pI 5.3; IDR (18.1%; aa554-699); shares several peptides with Tri_2770 | GRP78_MOUSE  (aa1-655) | 100.0 | 0e0 | -  8  11  4  13  - | -  12  14  5  24  - | -  0.009  0.008  <0.001  0.002  - | I_A_  I_B_  I_C_  S_A_  S_B_  S_C_ |
| **H_sp_Tri_2770_c0_g1_i1^3^**  (aa1-670)  **Comp99703_c0_seq1_2** | 78kDa glucose-regulated protein; 9.4% E; pI 4.9; domains: HSP70_peptide-bd (aa426-568,571-653); shares several peptides with comp136401_c0_seq1_5; IDR (27.6%; aa20-41,289-296,489-495,523-670) | Q75W49_CRAGI  (aa1-661) | 84.5 | 8.5e-201 | -  -  -  7  -  - | -  -  -  22  -  - | -  -  -  0.002  -  - | I_A_  I_B_  I_C_  S_A_  S_B_  S_C_ |
| **Comp31935_c0_seq1_4^3^**  (aa10-184)  **Comp144616_c0_seq1_6** | Calmodulin; domain: EF-hand-dom_pair (aa8-161); 11.3% E; pI 4.5; shares most peptides with Comp68174_c0_seq1_5 and Tri_60708 | S7MGT2_MYOBR  (aa1-167) | 88.1 | 5.2e-50 | -  4  1  1  -  - | -  10  1  1  -  - | -  0.039  0.005  0.001  -  - | I_A_  I_B_  I_C_  S_A_  S_B_  S_C_ |
| **Comp68174_c0_seq1_5^3^**  (aa7-155)  H_sp_CLC_2355_c0_g1_i1 | Calmodulin; domain: EF-hand-dom_pair (aa4-154); 10.6% D, 13.0% E; pI 4.2; shares peptides with Tri_60708 | CALM_SACJA  (aa1-149) | 99.3 | 2.2e-48 | -  1  2  2  3  - | -  3  6  12  11  - | -  0.012  0.014  0.004  <0.001  - | I_A_  I_B_  I_C_  S_A_  S_B_  S_C_ |
| **H_sp_Tri_60708_c0_g1_i1^3^**  (aa1-149)  **H_sp_Tri_60705_c0_g1_i1**  B3SND3_HALDV  Comp31935_c0_seq2_5 | Calmodulin; domain: EF-hand-dom_pair (2-149); 11.4% D, 14.1% E; pI 4.1; IDR (46.9%; aa1-59,119-128); shares 1 peptide with comp68174_c0_seq1_5/comp31935_c0_seq1_4; N-term acA_2_ | B3SND3_HALDV  (aa1-149) | 100.0 | 1.9e-49 | -  1  1  1  1  1 | -  3  1  3  5  4 | -  0.013  0.009  0.003  0.003  0.002 | I_A_  I_B_  I_C_  S_A_  S_B_  S_C_ |
| **Comp146988_c0_seq1_6**  (aa1-291) | HSP90b1/endoplasmin; domains: HATPase_C (aa1-102), Ribosomal_S5_D2-typ_fold (aa146-288); 11.6% E, 11.6% K; pI 4.9; IDR (25.0%; aa28-52,91-139) | Q3TUD6_MOUSE  (aa17-307) | 100.0 | 1.5e-95 | -  -  2  -  2  - | -  -  2  -  2  - | -  -  0.001  -  <0.001  - | I_A_  I_B_  I_C_  S_A_  S_B_  S_C_ |
| **Comp147991_c0_seq1_2**  (aa16-447) | Adenosylhomocysteinase; domain: Ado_hCys_hydrolase_NAD-bd (aa206-367); 11.2% L; pI 7.6; IDR (4.6%; aa1-17,573-586) | SAHH_MOUSE  (aa1-432) | 100.0 | 3.1e-182 | -  -  -  3  2  - | -  -  -  4  3  - | -  -  -  0.001  0.001  - | I_A_  I_B_  I_C_  S_A_  S_B_  S_C_ |
| **H_sp_Tri_808_c0_g1_i1^3^**  (aa18-132)  **Comp157484_c0_seq1_1** | Similar to zinc metalloproteinase nas-39; domain: CUB (aa18-134); SSP (aa1-19); 11.0% I; pI 4.7 | NAS39_CAEEL  (aa550-646) | 35.0 | 1.8e-5 | -  -  2  2  2  2 | -  -  5  4  4  2 | -  -  0.012  0.001  0.004  0.001 | I_A_  I_B_  I_C_  S_A_  S_B_  S_C_ |
| **Comp16557_c0_seq1_3**  (aa1-121)  **Q6YNH2_HALTU**  **etc**  H_sp_Tri_36561_c0_g1_i1 | Histone H3; domain: Histone_H2A/H2B/H3 (aa1-121); 12.4% A, 11.6% R, 9.9% L, 9.9% K; pI 10.8; IDR (32.2%; aa1-39) | F8J4D4_9BILA  (aa3-123) | 100.0 | 1.2e-47 | -  -  2  -  2  - | -  -  5  -  2  - | -  -  0.024  -  0.042  - | I_A_  I_B_  I_C_  S_A_  S_B_  S_C_ |
| **Comp174131_c0_seq1_2**  (aa1-389) | Protein disulfide-isomerase; domains: Thioredoxin-like_fold (aa1-89,98-198,186-319,321-385); 10.5% E; pI 5.0; IDR (2.6%; aa310-319) | Q3TF72_MOUSE  (aa49-437) | 100.0 | 1.0e-157 | -  4  6  5  4  - | -  4  7  10  5  - | -  0.002  0.004  <0.001  <0.001  - | I_A_  I_B_  I_C_  S_A_  S_B_  S_C_ |
| **Comp18904_c0_seq1_2**  (aa1-202)  H_sp_Tri_59763_c0_g1_i1 | Proteasome subunit alpha type; domain: Ntn_hydrolases_N (aa1-197); pI 9.0; IDR (16.0%; aa183-219) | Q542H2_MOUSE  (aa47-248) | 100.0 | 4.3e-78 | -  -  3  -  3  - | -  -  4  -  7  - | -  -  0.003  -  0.004  - | I_A_  I_B_  I_C_  S_A_  S_B_  S_C_ |
| **Comp205463_c0_seq1_5**  H_sp_idb_35864_c0_g1_i1 | Proteasome subunit alpha type-6; domain: Ntn_hydrolases_N (aa25-192); pI 8.7 | PSA6_MOUSE  (aa1-171) | 100.0 | 1.0e-76 | -  -  3  2  3  - | -  -  3  9  6  - | -  -  0.002  0.001  0.004  - | I_A_  I_B_  I_C_  S_A_  S_B_  S_C_ |
| **Comp229571_c0_seq1_1**  (aa23-263)  H_sp_Tri_30403_c0_g1_i1 | Proteasome subunit alpha type; domain: Ntn_hydrolases_N (aa29-269); pI 4.7 | H2N6I5_PONAB  (aa1-241) | 100.0 | 4.3e-98 | -  -  -  -  2  3 | -  -  -  -  3  3 | -  -  -  -  0.002  0.001 | I_A_  I_B_  I_C_  S_A_  S_B_  S_C_ |
| **Comp23247_c0_seq1_2^3^**  (aa46-424) | Fructose-bisphosphate aldolase; domain: Aldolase_TIM (aa64-407); 10.7% A; pI 8.7;IDR (16.3%; aa1-79) | A6ZI44_MOUSE  (aa40-418) | 98.4 | 8.9e-162 | 2  6  10  5  6  2 | 2  13  16  15  16  3 | 0.002  0.012  0.015  0.003  0.007  0.002 | I_A_  I_B_  I_C_  S_A_  S_B_  S_C_ |
| **Comp23692_c0_seq1_3^3^**  (aa1-250)  Q45Y86_HALRU | Triosephosphate isomerase; domain: Aldolase_TIM (aa2-250); pI 7.9 | TPIS_MOUSE  (aa50-299) | 100.0 | 4.1e-107 | -  -  2  6  5  3 | -  -  3  18  10  3 | -  -  0.002  0.002  0.004  0.001 | I_A_  I_B_  I_C_  S_A_  S_B_  S_C_ |
| **Comp24068_c0_seq1_6**  (aa26-179) | Nucleoside diphosphate kinase; domain: NDK-like (aa27-179); pI 9.3 | E9PZF0_MOUSE  (aa114-267) | 100.0 | 2.0e.66 | -  -  3  4  3  - | -  -  3  6  9  - | -  -  0.005  0.003  0.023  - | I_A_  I_B_  I_C_  S_A_  S_B_  S_C_ |
| **Comp24075_c0_seq1_1**  (aa1-218)  **Comp24075_c0_seq2_1** | 40S ribosomal protein S8 (Fragment); 11.1% R, 13.2% K; pI 10.3; IDR (32.9%; aa1-6,137-153) | G3UCL9_LOXAF  (aa3-220) | 99.1 | 4.6e-93 | -  -  2  2  4  - | -  -  2  3  4  - | -  -  0.002  <0.001  0.001  - | I_A_  I_B_  I_C_  S_A_  S_B_  S_C_ |
| **Comp24760_c0_seq1_4**  (aa1-268)  B6RB83_HALDI | 40S ribosomal protein S4; domains: Ribosomal_S4e_N (aa8-44), S4_RNA-bd (aa49-95,142-190), KOW (183-216), 40S_S4_C (aa217-264); 10.6% K; pI 10.6; IDR (8.0%; aa1-11,288-300) | A0A0H2UHX3  _RAT  (aa23-290) | 100.0 | 4.8e-120 | -  -  -  2  4  - | -  -  -  2  4  - | -  -  -  <0.001  0.001  - | I_A_  I_B_  I_C_  S_A_  S_B_  S_C_ |
| **Comp24999_c0_seq1_4**  (aa1-436) | Elongation factor 1-gamma; domains: Glutathione_S-Trfase_N (aa1-86), GST_C (aa81-234), Transl_elong_EF1_G_con (aa274-436); pI 6.6; IDR (15.7%; aa211-282) | Q4FZK2_MOUSE  (aa2-437) | 100.0 | 1.2e-160 | -  2  3  3  8  - | -  4  3  7  14  - | -  0.001  0.002  0.001  0.002  - | I_A_  I_B_  I_C_  S_A_  S_B_  S_C_ |
| **Comp25767_c0_seq1_2**  (aa146-337)  G9K385_HALDV  H_sp_idb_8508_c0_g1_i1 | Polyadenylate-binding protein 1; domain: RRM (aa155-324); 10.4% P; pI 10.0; IDR (53.6%; aa1-158,315-337) | A0A024R9E2  _HUMAN  (aa1-192) | 100.0 | 2.0e-47 | -  -  2  -  2  - | -  -  2  -  2  - | -  -  0.001  -  <0.001  - | I_A_  I_B_  I_C_  S_A_  S_B_  S_C_ |
| **Comp26587_c0_seq1_2**  (aa26-279) | Phosphoglycerate mutase 1; pI 7.3; IDR (2.8%; aa1-16) | PGAM1_RAT  (aa1-254) | 100.0 | 2.4e-109 | -  2  3  2  2  - | -  2  4  3  2  - | -  0.001  0.003  <0.001  <0.001  - | I_A_  I_B_  I_C_  S_A_  S_B_  S_C_ |
| **Comp26600_c0_seq1_4**  (aa12-146) | Fatty acid binding protein 5, domain: Fatty_acid-bd (aa14-146); epidermal; 10.5% L; pI 8.6 | Q497I3_MOUSE  (aa1-135) | 100.0 | 3.0e-58 | 2  2  -  -  2  - | 2  10  -  -  31  - | 0.060  0.121  -  -  0.209  - | I_A_  I_B_  I_C_  S_A_  S_B_  S_C_ |
| **Comp310163_c0_seq1_1**  (aa1-112) | Uncharacterized; domain: clathrin_heavy_chain/VPS, 7-fold_repeat (aa1-110); 12.5% A, 9.8% E, 9.8% L; pI 6.9 | K7GQL8_PIG  (aa384-495) | 100.0 | 6.4e-48 | -  -  2  2  -  - | -  -  2  3  -  - | -  -  0.006  0.001  -  - | I_A_  I_B_  I_C_  S_A_  S_B_  S_C_ |
| **Comp32839_c0_seq1_6**  (aa3-122) | Putative actin related protein 2/3 complex subunit 4; 9.8% E, 9.8% L; pI 9.3 | K9IGN5_DESRO  (aa2-120) | 100.0 | 2.4e-49 | -  2  2  3  -  - | -  2  2  6  -  - | -  0.002  0.002  0.002  -  - | I_A_  I_B_  I_C_  S_A_  S_B_  S_C_ |
| **Comp32983_c1_seq1_3**  (aa1-123) | Proliferation-associated 2G4; domains: WHTH_DNA-bd (aa1-29), Pept_M24 (aa31-76); 9.9% A, 9.9% E, 9.9% L, 9.3% K, 9.3% P; pI 8.7; IDR (60.7%;aa65-163) | Q3TGU7_MOUSE  (aa272-394) | 100.0 | 3.4e-40 | -  -  2  -  3  - | -  -  2  -  3  - | -  -  0.002  -  <0.001  - | I_A_  I_B_  I_C_  S_A_  S_B_  S_C_ |
| **Comp36515_c0_seq1_6**  (aa14-141)  H_sp_Tri_52307_c0_g1_i1 | Histone H2A-J; domains: Histone_H2A/H2B/H3 (aa8-102), Histone_H2A_C (aa105-137); 11.3% A, 9.9% R, 12.0% L; pI 11.0; IDR (43.7%; aa1-45,126-141) | H2AJ_RAT  (aa1-128) | 97.7 | 1.2e-47 | -  2  2  2  2  - | -  5  4  18  16  - | -  0.025  0.092  0.016  0.041  - | I_A_  I_B_  I_C_  S_A_  S_B_  S_C_ |
| **Comp40524_c0_seq1_2**  (aa1-80) | Proteasome subunit alpha type (Fragment); domain: Ntn_hydrolases_N (aa1-80); 11.2% A, 12.5% V; pI 6.5 | R0JT34_ANAPL  (aa6-85) | 100.0 | 4.1e-32 | -  -  -  2  2  - | -  -  -  4  6  - | -  -  -  0.001  0.008  - | I_A_  I_B_  I_C_  S_A_  S_B_  S_C_ |
| **Comp41851_c0_seq2_2**  (aa9-159)  **Comp41851_c0_seq1_2** | Myosin light polypeptide 6; domains: EFh_pair (aa12-82,83-158); 11.1% L, 10.1% V; pI 4.9; IDR (4.8%; aa1-10) | H2NHN7_PONAB  (aa1-151) | 100.0 | 9.7e-58 | -  4  6  4  3  - | -  5  6  7  5  - | -  0.007  0.011  0.001  0.001  - | I_A_  I_B_  I_C_  S_A_  S_B_  S_C_ |
| **Comp45831_c0_seq1_1**  (aa1-150)  **Comp148966_c0_seq1_4**  H_sp_Tri_53506_c0_g1_i1 | 60S ribosomal protein L26; domain: Rib_L2_dom2 (aa5-132); 10.8% R, 15.7% K; pI 10.7; IDR (77.1%; aa1-60,99-164) | R0L0B4_ANAPL  (aa1-150) | 98.7 | 1.2e-53 | -  -  2  -  2  - | -  -  2  -  2  - | -  -  0.001  -  0.001  - | I_A_  I_B_  I_C_  S_A_  S_B_  S_C_ |
| **H_sp_Tri_23502_c0_g1_i1^3^**  **Comp46614_c0_seq1_4** | Uncharacterized; 12.8% S, 22.6% T, pI 4.1; IDP |  |  |  | -  2  3  -  -  - | -  4  9  -  -  - | -  0.019  0.055  -  -  - | I_A_  I_B_  I_C_  S_A_  S_B_  S_C_ |
| **Comp48021_c0_seq1_2**  (aa21-315)  H_sp_idb_46813_c0_g1_i1  B6RB17_HALDI | 40S ribosomal protein SA; domains: Ribosomal_S2_flav_dom (aa29-220), 40S_SA_C (aa222-315); 12.5% A; pI 5.2; IDR (34.3%; aa223-336) | RSSA_MOUSE  (aa1-295) | 100.0 | 4.3e-119 | -  2  4  3  3  - | -  3  5  7  6  - | -  0.004  0.003  0.001  0.003  - | I_A_  I_B_  I_C_  S_A_  S_B_  S_C_ |
| **Comp48128_c0_seq1_4^3^**  (aa19-541) | Elongation factor 1-alpha; domains: TF_GTP-bd (aa21-260), EFTu-like_2 (aa278-343), Transl_elong_EFTu/EF1A_C (aa354-459); 9.9% K; pI 9.4; IDR (1.2%; aa1-7) | W5PHA3_SHEEP  (aa1-525) | 97.7 | 3.0e-131 | 5  6  6  5  8  - | 5  20  16  39  59  - | 0.005  0.025  0.019  0.009  0.039  - | I_A_  I_B_  I_C_  S_A_  S_B_  S_C_ |
| **H_sp_Tri_2952_c0_g1_i1^3^**  (aa43-180)  **Comp48289_c0_seq1_1**  **etc** | Uncharacterized; domain: MD-2_related_lipid_recognition (ML; aa73-175); SSP (aa1-18); pI 6.3; IDR (3.7%; aa181-187) | K1RHT2_CRAGI  (aa486-625) | 29.8 | 6.1e-10 | -  -  -  5  4  2 | -  -  -  11  9  2 | -  -  -  0.006  0.007  <0.001 | I_A_  I_B_  I_C_  S_A_  S_B_  S_C_ |
| **Comp48409_c0_seq1_3**  (aa11-259) | 40S ribosomal protein S6; 10.6% R, 13.2% K; pI 10.4; IDR (46.2%; aa160-276,281-303)) | RS6_MOUSE  (aa1-249) | 100.0 | 3.9e-82 | -  -  2  -  2  - | -  -  2  -  2  - | -  -  0.002  -  <0.001  - | I_A_  I_B_  I_C_  S_A_  S_B_  S_C_ |
| **Comp48749_c0_seq1_3**  (aa19-133) | 60S ribosomal protein L30; domain: L30e-like (aa18-123); 11.4% L, 11.4% K; pI 9.9; IDR (14.1%; aa129-149) | RL30_MOUSE  (aa1-115) | 100.0 | 3.0e-46 | -  -  2  -  2  - | -  -  2  -  5  - | -  -  0.004  -  0.002  - | I_A_  I_B_  I_C_  S_A_  S_B_  S_C_ |
| **Comp49273_c0_seq1_2^3^**  H_sp_Tri_105572_c0_g1_i1 | Uncharacterized; 10.3% N, 14.0% Q, 10.3% S, pI 8.7; shares several peptides with idb_46434; SSP (aa1-27); IDR (61.0%; aa42-220); tandem repeats in aa88-96 ([NAYGT]_2_) |  |  |  | -  1  5  4  4  6 | -  1  24  17  22  76 | -  0.001  0.051  0.007  0.016  0.112 | I_A_  I_B_  I_C_  S_A_  S_B_  S_C_ |
| **H_sp_idb_46434_c0_g1_i1^3^** | Uncharacterized; SSP (aa1-22); 11.7% N, 15.2% Q, 10.3% G, 10.8% S; pI 9.3; IDR (63.7%; aa52-194); shares several peptides with comp49273_c0_seq1_2 |  |  |  | -  2  -  -  2  - | -  4  -  -  62  - | -  0.009  -  -  0.087  - | I_A_  I_B_  I_C_  S_A_  S_B_  S_C_ |
| **Comp50924_c0_seq1_4**  (aa7-210) | Ribosomal protein L15; domain: Rbsml_L15e_core (aa7-201); 14.9% R; pI 11.5; IDR (13.2%; aa163-192) | A0A0D9RBM0  _CHLSB  (1-204) | 100.0 | 2.1e-90 | -  -  -  -  3  2 | -  -  -  -  7  3 | -  -  -  -  <0.001  0.001 | I_A_  I_B_  I_C_  S_A_  S_B_  S_C_ |
| **Comp51700_c0_seq3_3^3^**  (aa17-214)  B1N694_HALDI  H_sp_idb_57414_c0_g1_i1 | Peroxiredoxin-1;domain: Thioredoxin-like_fold (aa7-201); pI 6.4; IDR (6.5%; aa201-214) | PRDX1_MOUSE  (aa1-198) | 100.0 | 2.4e-88 | -  4  3  4  3  2 | -  9  8  28  24  18 | -  0.014  0.010  0.007  0.032  0.005 | I_A_  I_B_  I_C_  S_A_  S_B_  S_C_ |
| **Comp51969_c0_seq1_2**  (aa1-271) | Uncharacterized/GNB2L1; domain: WD40/YVTN_repeat-like (aa34-263); SSP (aa1-22) or TM (aa7-29); 9.9% G, 9.6% L, 9.9% S, 9.6% T; pI 8.5; if SSP: 9.6% S, 10.4% T; pI 8.3 | G1QR41_NOMLE  (aa48-318) | 96.7 | 1.2e-87 | -  -  2  2  -  - | -  -  2  4  -  - | -  -  0.003  <0.001  -  - | I_A_  I_B_  I_C_  S_A_  S_B_  S_C_ |
| **H_sp_CLC_18921_c0_g1_i1^3^**  (aa9-603)  **Comp52213_c0_seq1_3** | Uncharacterized; SSP (aa1-20); pI 5.6 | V3ZWU1_LOTGI  Lotgidraft_154902  (aa9-600) | 32.7 | 1.2e-81 | 3  7  10  -  2  - | 5  14  28  -  3  - | 0.007  0.018  0.027  -  <0.001  - | I_A_  I_B_  I_C_  S_A_  S_B_  S_C_ |
| **Comp52297_c0_seq1_2^3^**  (aa10-221)  **H_sp_idb_10314_c0_g1_i1** | Similar to Sushi, von Willebrand factor type A, EGF and pentraxin domain-containing protein 1; domain: ConA-like (aa42-224); 11.1% N; pI 9.1; IDR (12.2%; aa239-270) | K1R3V2_CRAGI**^4^**  (aa2487-2688) | 34.3 | 1.1e-12 | 2  2  5  4  -  4 | 4  2  19  19  -  34 | 0.012  0.004  0.101  0.005  -  0.043 | I_A_  I_B_  I_C_  S_A_  S_B_  S_C_ |
| **H_sp_Tri_44993_c0_g1_i1**  (aa1-191)  **Comp52403_c0_seq1_5** | Ribosomal protein S9; domains: Ribosomal_S4/S9_N (aa3-106), S4_RNA-bd (aa107-178); 14.7% R, 13.1% L; pI 10.7; IDR (26.7%; aa1-18,159-191) | A9LMJ6_HALDI  (aa1-191) | 99.5 | 7.6e-80 | -  -  3  -  2  - | -  -  3  -  2  - | -  -  0.005  -  0.002  - | I_A_  I_B_  I_C_  S_A_  S_B_  S_C_ |
| **Comp52564_c0_seq2_5**  (aa31-496) | Vimentin; domain: Intermed_filament_DNA-bd (aa37-131); 10.2% E, 11.2% L; pI 5.8; IDR (41.9%; aa1-118,325-394,441-484,495-515) | Q5FWJ3_MOUSE  (aa1-466) | 100.0 | 1.2e-108 | -  -  6  -  2  - | -  -  6  -  3  - | -  -  0.001  -  <0.001  - | I_A_  I_B_  I_C_  S_A_  S_B_  S_C_ |
| **Comp53070_c0_seq1_3**  (aa42-179) | Cellular retinoic acid-binding protein 2; domain: Fatty_acid-bd (aa43-179); pI 9.4; IDR (20.0%; aa185-203,224-254) | RABPL_MOUSE  (aa1-138) | 100.0 | 4.7e-50 | -  -  -  2  2  - | -  -  -  3  4  - | -  -  -  <0.001  0.001  - | I_A_  I_B_  I_C_  S_A_  S_B_  S_C_ |
| **Comp54249_c0_seq1_2**  (aa8-829)  H_sp_CLC_2239_c0_g1_i1 | Valosin-containing protein/epididymis luminal protein 220/transitional endoplasmic reticulum ATPase; domains: CDC4_N-term_subdom (aa45-131), Cdc48_dom2 (aa130-221), AAA+_ATPase (aa223-485,495-784); pI 5.5; IDR (22.2%; aa1-50,209-223,727-831,856-900) | G9KX59_MUSPF  (aa1-822) | 99.6 | 0e0 | -  2  10  7  5  - | -  2  12  11  9  - | -  <0.001  0.003  0.011  0.001  - | I_A_  I_B_  I_C_  S_A_  S_B_  S_C_ |
| **Comp59223_c0_seq1_2^3^**  (aa17-123) | Similar to putative ferric-chelate reductase 1-like protein; shares most peptides with Tri_28544; 10.3% S; pI 9.5; IDR (35.0%; aa116-176) | K1RDF5_CRAGI  (aa518-626) | 45.5 | 2.0e-10 | 2  1  1  2  1  1 | 10  9  7  48  19  10 | 0.144  0.097  0.034  0.142  0.049  0.028 | I_A_  I_B_  I_C_  S_A_  S_B_  S_C_ |
| **H_sp_Tri_28544_c0_g1_i1^3^**  (aa3-361) | Uncharacterized; domains: Reelin (aa10-185), DOMON (aa215-336); SSP (aa1-19); 9.8% T; pI 7.4; shares most peptides with comp59223  _c0_seq1_2; K1RDF5_CRAGI: aa15-362; 31.7%, 1.2e-24 | V4AG13_LOTGI  Lotgidraft_162625  (aa7-359) | 38.8 | 2.4e-46 | 13  16  13  16  12  6 | 102  108  100  215  171  31 | 2.420  1.627  1.247  0.287  0.221  0.012 | I_A_  I_B_  I_C_  S_A_  S_B_  S_C_ |
| **Comp59460_c1_seq1_5**  (aa10-175)  H_sp_idb_68343_c0_g1_i1  B6RB72_HALDI | ADP-ribosylation factor 1; domain: Small_GTP-bd (aa29-175); pI 7.1 | A0A096NAX4  _PAPAN  (aa7-172) | 100.0 | 4.0e-59 | -  2  -  2  2  - | -  2  -  3  2  - | -  0.002  -  0.001  <0.001  - | I_A_  I_B_  I_C_  S_A_  S_B_  S_C_ |
| **Comp62063_c0_seq1_1^3^**  (aa6-880)  H_sp_idb_65205_c0_g1_i1 | Elongation factor 2; domains: P-loop_NTPase (aa26-382), EFTu-like_2 (aa432-507), EFG_III-V (aa522-597), Transl_elong_ EFG/EF2_IV (aa643-759), EFG_V (aa761-850); pI 6.8; IDR (4.3%; aa1-22,981-1007) | EF2_MOUSE  (aa100-974) | 100.0 | 0e0 | -  3  10  9  12  - | -  7  14  15  28  - | -  0.001  0.004  0.001  0.006  - | I_A_  I_B_  I_C_  S_A_  S_B_  S_C_ |
| **Comp62064_c0_seq1_4**  (aa2-359) | L-lactate dehydrogenase; domains: NAD(P)-bd (aa29-187), Lactate_DH/Glyco_Ohase_4_C (aa188-357); 10.6% L; pI 8.3; IDR (7.1%; 1-29,514-521) | G5E8N5_MOUSE  (aa4-361) | 96.1 | 1.0e-141 | -  2  3  3  6  - | -  3  3  7  15  - | -  0.002  0.004  0.002  0.005  - | I_A_  I_B_  I_C_  S_A_  S_B_  S_C_ |
| **Comp63077_c0_seq1_4**  (aa4-154)  H_sp_Tri_84611_c0_g1_i1 | 40S ribosomal protein S13; domains: Ribosomal_S13/S15_N (aa4-63), S15_NS1_RNA-bd (aa69-145); 13.0% L, 11.8% K, pI 10.5; IDR (12.4%; aa1-21) | RS13_HUMAN  (aa1-151) | 100.0 | 4.8e-60 | -  -  3  -  2  - | -  -  3  -  2  - | -  -  0.003  -  <0.001  - | I_A_  I_B_  I_C_  S_A_  S_B_  S_C_ |
| **Comp63526_c0_seq4_3**  (aa49-349)  **Comp63526_c0_seq1_3**  **Comp63526_c0_seq2_3**  **Comp63526_c0_seq3_3** | Heterogeneous nuclear ribonucleoproteins A2/B1; domains: RRM_dom (aa55-142,145-254); 17.9% G, 9.9% S; pI 9.3; IDR (47.1%; aa22-56,218-278,282-358) | F1LM82_RAT  (aa1-301) | 99.7 | 3.0e-71 | -  -  4  -  4  2 | -  -  4  -  6  3 | -  -  0.002  -  <0.001  <0.001 | I_A_  I_B_  I_C_  S_A_  S_B_  S_C_ |
| **Comp64232_c0_seq1_6**  (aa1-65) | 40S ribosomal protein S18; domain: Ribosomal_S13-like_H2TH (aa10-66); 12.2% R, 11.0% G, 13.4% L, 13.4% K; pI 10.7 | G1R552_NOMLE  (aa111-175) | 100.0 | 8.2e-26 | -  -  1  -  1  - | -  -  1  -  1  - | -  -  0.008  -  0.001  - | I_A_  I_B_  I_C_  S_A_  S_B_  S_C_ |
| **Comp64232_c1_seq1_4**  (aa1-97) | 40S ribosomal protein S18; domain: Ribosomal_S13-like_H2TH (aa19-83); 10.3% A, 10.3% R, 10.3% I; pI 10.0 | G1R552_NOMLE  (aa18-114) | 99.0 | 1.2e-37 | -  1  4  -  2  - | -  1  4  -  2  - | -  0.001  0.006  -  0.001  - | I_A_  I_B_  I_C_  S_A_  S_B_  S_C_ |
| **H_sp_Tri_111928_c0_g1_i1^3^**  (aa36-274)  **Comp64272_c0_seq1_3** | Uncharacterized; SSP (aa1-24); pI 9.1 | K1QJ54_CRAGI**^4^**  (aa303-536) | 23.5 | 3.8e-5 | 2  7  9  3  4  2 | 3  16  30  36  18  17 | 0.013  0.046  0.134  0.018  0.025  0.012 | I_A_  I_B_  I_C_  S_A_  S_B_  S_C_ |
| **Comp64368_c0_seq1_4**  (aa1-403)  H_sp_Tri_26397_c0_g1_i1 | Eukaryotic initiation factor 4A-I; domains: RNA_helicase_ DEAD_ Q_motif (aa29-57), Helicase_ATP-bd (aa60-231), Helicase_C (aa242-403); pI 7.6; IDR (13.0%; aa1-24,487-523,570-585) | Q4FZL1_MOUSE  (aa3-405) | 100.0 | 8.3e-149 | -  2  5  5  7  - | -  3  6  6  16  - | -  0.002  0.003  0.001  0.005  - | I_A_  I_B_  I_C_  S_A_  S_B_  S_C_ |
| **Comp64599_c0_seq1_4**  (aa1-283) | 14-3-3 protein theta; domain: 14-3-3 (aa41-280); 11.0% L; pI 6.2; IDR (16.5%; aa1-53,270-300); shares 2 peptides with comp64599_c0_seq2_6 and other 14-3-3 proteins | F6VW30_MOUSE  (aa28-303) | 89.4 | 1.8e-98 | -  1  2  2  1  - | -  1  2  4  1  - | -  <0.001  0.001  0.001  <0.001  - | I_A_  I_B_  I_C_  S_A_  S_B_  S_C_ |
| **Comp64599_c0_seq2_6**  (aa32-283) | 14-3-3 protein zeta/delta; 10.9% L; pI 5.3; IDR (15.8%; aa1-53,270-293); shares 2 peptides with other 14-3-3 proteins | A0A0G2JV65_  RAT  (aa5-256) | 99.2 | 3.4e-94 | 4  6  9  7  7  - | 4  23  24  32  34  - | 0.008  0.023  0.034  0.006  0.014  - | I_A_  I_B_  I_C_  S_A_  S_B_  S_C_ |
| **Comp65289_c0_seq1_1**  (aa29-646) | Heat shock protein 84b/heat shock protein HSP 90-beta; domains: HATPase_C (aa40-255), Ribosomal_S5_D2-typ_fold (aa312-567); 12.8% E, 10.7% K; pI 5.2; IDR (22.0%; aa1-43,243-310,568-588,637-646); shares several peptides with comp65289_c0_seq2_1  (HSP90-alpha) | Q71LX8_MOUSE  (aa1-618) | 100.0 | 1.1e-165 | 1  6  11  7  12  - | 1  7  11  11  19  - | 0.002  0.006  0.015  0.004  0.003  - | I_A_  I_B_  I_C_  S_A_  S_B_  S_C_ |
| **Comp65289_c0_seq2_1**  (aa282-743) | Heat shock protein HSP 90-alpha; domain: Ribosomal_S5_D2-typ_fold (aa321-558); IDR (23.0%; aa1-39,57-112,232-306,563-576,713-738); shares several peptides with comp65289_c0_seq1_1 (HSP 90-beta) | Q3TJU7_MOUSE  (aa85-556) | 90.3 | 4.4e-167 | -  2  4  2  4  - | -  2  5  2  11  - | -  0.001  0.001  <0.001  0.001  - | I_A_  I_B_  I_C_  S_A_  S_B_  S_C_ |
| **Comp65289_c1_seq1_1**  (aa1-119) | Hsp90ab1 protein; 11.2% L; pI 4.7; IDR (51.0%; aa1-24,86-150,186-196) | F7GJQ3_MONDO  (aa244-362) | 100.0 | 5.1e-43 | -  2  -  -  2  - | -  2  -  -  18  - | -  0.004  -  -  0.039  - | I_A_  I_B_  I_C_  S_A_  S_B_  S_C_ |
| **Comp65317_c0_seq1_1**  (aa1-292)  H_sp_CLC_44_c0_g1_i1 | 40S ribosomal protein S2; domains: Ribosomal_S5_N (aa101-165), Ribosomal_S5_D2-typ_fold_subgr (aa177-257); 14.4% G; pI 10.2; IDR (19.7%; aa1-60) | Q58EU3_MOUSE  (aa2-293) | 100.0 | 3.0e-84 | -  -  2  -  2  - | -  -  2  -  2  - | -  -  0.002  -  0.001  - | I_A_  I_B_  I_C_  S_A_  S_B_  S_C_ |
| **Comp68339_c0_seq1_1** | Uncharacterized; pI 9.2 |  |  |  | 2  -  -  5  4  3 | 4  -  -  34  11  8 | 0.034  -  -  0.056  0.027  0.012 | I_A_  I_B_  I_C_  S_A_  S_B_  S_C_ |
| **Comp70759_c0_seq1_2^3^**  (aa33-116) | Similar to perlustrin; domain: Growth_fac_rcpt/IGFBP (aa32-98); SSP (aa1-32); 10.3% C; pI 8.0; IDR (28.2%; aa116-149) | PLS_HALLA**^4^**  (aa1-84) | 70.2 | 1.2e-24 | -  -  3  5  6  7 | -  -  12  68  75  67 | -  -  0.296  0.307  0.396  1.392 | I_A_  I_B_  I_C_  S_A_  S_B_  S_C_ |
| **Comp71437_c1_seq1_4**  (aa19-275) | 60S acidic ribosomal protein P0; 10.2% L; pI 9.2; IDR (7.3%; aa1-20) | X5E4X2_MICPE  (aa1-257) | 100.0 | 1.8e-103 | -  2  3  4  3  - | -  3  3  5  4  - | -  0.002  0.002  <0.001  <0.001  - | I_A_  I_B_  I_C_  S_A_  S_B_  S_C_ |
| **H_sp_Tri_128009_c0_g1_i1**  (aa12-113)  **Comp71699_c0_seq1_1** | Uncharacterized; SSP (aa1-22); 12.6% N, 10.7% G, 9.7% K; pI 9.0; IDR (8.7%; aa116-125) | V4A983_LOTGI  Lotgidraft_157812  (aa26-125) | 34.3 | 1.0e-4 | -  -  -  2  2  - | -  -  -  8  6  - | -  -  -  0.020  0.020  - | I_A_  I_B_  I_C_  S_A_  S_B_  S_C_ |
| **Comp72553_c0_seq1_3**  (aa27-311) | Heterogeneous nuclear ribonucleoprotein A1; domains: hnRNPA1_RRM1 (aa38-118), hnRNPA1_RRM2 (aa131-207); 11.6% G; pI 9.2; IDR (27.6%; aa16-43,110-132,198-310) | H9F0Q1_MACMU  (aa1-285) | 100.0 | 6.5e-65 | -  -  4  -  2  - | -  -  6  -  3  - | -  -  0.001  -  <0.001  - | I_A_  I_B_  I_C_  S_A_  S_B_  S_C_ |
| **Comp73608_c1_seq1_6^3^**  (aa47-180) | Similar to nattectin; domain: C-type_lectin (aa27-181); TM (aa20-37); 10.2% T; pI 9.6; IDR (17.7%; aa1-11,189-215) | G1FKF5_EPIBR  (aa32-161) | 35.1 | 2.1e-15 | 3  2  4  4  2  - | 5  2  15  7  2  - | 0.008  0.006  0.030  0.001  0.001  - | I_A_  I_B_  I_C_  S_A_  S_B_  S_C_ |
| **H_sp_Tri_25175_c0_g1_i1**  **H_sp_idb_71559_c0_g1_i1**  Comp75869_c0_seq2_3 | Uncharacterized; SSP (aa1-19)/TM (aa4-22); 11.0% P, 14.0% Y; pI 8.7; IDP; if SSP: 10.3% Q, 12.8% P, 16.2% Y; pI 9.1 |  |  |  | -  3  -  2  2  - | -  5  -  15  4  - | -  0.061  -  0.010  0.006  - | I_A_  I_B_  I_C_  S_A_  S_B_  S_C_ |
| **Comp78671_c0_seq1_1**  (aa1-247) | 40S ribosomal protein S3; domains: hnRNPA1_RRM2 (aa9-96), Ribosomal_S3_C (aa110-192); 9.6% G; pI 9.9; IDR (15.9%; aa208-250) | G1MF88_AILME  (aa2-248) | 99.6 | 4.4e-91 | -  2  8  4  6  - | -  2  8  7  6  - | -  0.001  0.005  0.001  0.001  - | I_A_  I_B_  I_C_  S_A_  S_B_  S_C_ |
| **Comp79549_c0_seq1_2^3^**  (aa6-663)  Q17UC1_HALTU  H_sp_idb_28957_c0_g1_i1 | Heat shock cognate 71 kDa protein; domains: HSP70_peptide-bd (aa407-538), HSP70_C (aa539-637); pI 6.5; IDR (29.8%; aa1-22,510-693); shares several peptides with idb_22145 (HSP70) | M0R8M9_RAT  (aa1-658) | 97.8 | 0e0 | 3  12  14  11  16  3 | 3  21  26  39  42  5 | 0.002  0.014  0.014  0.005  0.018  <0.001 | I_A_  I_B_  I_C_  S_A_  S_B_  S_C_ |
| **Comp79626_c0_seq1_4^3^**  (aa30-540)  H_sp_CLC_4455_c0_g1_i1 | Similar to chitinase-3; domains: chitinase_II (aa46-420), chitin-bd_II (aa476-534); TM (aa20-42); pI 8.7; IDR (14.1%; aa1-10, 426-477,537-552); shares most peptides with idb_43266 | J7FIC1_HYRCU  (aa8-515) | 40.4 | 3.9e-70 | 12  16  18  1  11  6 | 37  55  66  13  90  19 | 0.112  0.162  0.179  0.001  0.027  0.004 | I_A_  I_B_  I_C_  S_A_  S_B_  S_C_ |
| **H_sp_idb_43266_c0_g1_i1^3^**  (aa16-520) | Similar to chitinase-3; domains: Chitinase_II (aa33-407); chitin-bd_II (aa463-519); SSP (aa1-28); pI 8.1; IDR (10.0%; aa414-465); shares most peptides with comp79626_c0_seq1_4 | J7FIC1_HYRCU  (aa8-509) | 40.3 | 2.8e-71 | -  -  -  11  -  - | -  -  -  109  -  - | -  -  -  0.027  -  - | I_A_  I_B_  I_C_  S_A_  S_B_  S_C_ |
| **Comp80185_c0_seq1_5^3^**  (aa34-467) | Alpha-enolase; domains: Enolase_N (aa36-173), Enolase_C (aa176-464); pI 8.4; IDR (10.1%; aa1-4,462-505,563-571) | ENOA_MOUSE  (aa1-434) | 100.0 | 2.9e-176 | 5  7  6  7  10  4 | 9  18  22  63  57  13 | 0.004  0.021  0.014  0.009  0.016  0.001 | I_A_  I_B_  I_C_  S_A_  S_B_  S_C_ |
| **Comp80870_c0_seq3_4**  (aa56-141)  **Comp80870_c0_seq4_5** | Uncharacterized; TM (aa59-80);10.4% S, 13.2% V; pI 9.5 | B3TK42_HALDV  (aa1-86) | 65.1 | 1.5e-23 | -  -  -  -  2  2 | -  -  -  -  15  5 | -  -  -  -  0.047  0.013 | I_A_  I_B_  I_C_  S_A_  S_B_  S_C_ |
| **Comp81444_c0_seq1_6^3^**  (aa1-44) | Similar to cystatin B 4; 9.5% K, 9.5% S; pI 9.7 | M4H503_HALDI  (aa33-77) | 58.1 | 2.1e-7 | -  -  -  2  2  2 | -  -  -  29  14  18 | -  -  -  0.022  0.038  0.057 | I_A_  I_B_  I_C_  S_A_  S_B_  S_C_ |
| **H_sp_idb_12724_c0_g1_i1^3^**  (aa15-307)  **Comp82638_c0_seq1_6** | Uncharacterized; SSP (aa1-30); pI 7.1 | V3ZWU6_LOTGI**^4^**  Lotgidraft_154590  (aa4-293) | 41.2 | 6.3e-50 | 5  6  5  5  4  2 | 11  16  20  29  30  9 | 0.021  0.031  0.024  0.005  0.013  0.002 | I_A_  I_B_  I_C_  S_A_  S_B_  S_C_ |
| **Comp83799_c2_seq1_1^3^**  (aa53-269)  **H_sp_idb_22374_c0_g1_i1** | Uncharacterized; domain: EF_hand (aa91-199); 14.3% Q, 9.6% P; pI 5.9; C-term: QQPPP- and similar repeats; IDR (67.1%; aa1-13,57-102,128-175,180-219) | V3ZTJ0_LOTGI  Lotgidraft_234943  (aa23-233) | 48.9 | 7.3e-17 | -  -  2  2  2  2 | -  -  9  36  39  31 | -  -  0.023  0.030  0.022  0.027 | I_A_  I_B_  I_C_  S_A_  S_B_  S_C_ |
| **Comp84033_c0_seq1_3**  (aa1-92) | Similar to peptidoglycan-recognition protein; domain: PGRP_domain_met/bac (aa1-92); TM (aa97-119); 9.4% A, 9.4% G; pI 9.4; IDR (6.2%; aa123-130) | F7DQG1_HORSE  (aa98-184) | 55.9 | 4.2e-18 | -  2  3  -  -  - | -  3  4  -  -  - | -  0.009  0.017  -  -  - | I_A_  I_B_  I_C_  S_A_  S_B_  S_C_ |
| **Comp84928_c0_seq1_4^3^**  (aa41-166) | Similar to BPTI/Kunitz domain-containing protein (fragment); domains: Kunitz_BPTI (aa45-105,108-162); pI 9.4; SSP? (aa1-47) | KCP_HALAI**^4^**  (aa1-126) | 83.3 | 9.1e-53 | 3  4  4  7  9  4 | 7  15  17  46  56  23 | 0.044  0.132  0.152  0.146  0.445  0.242 | I_A_  I_B_  I_C_  S_A_  S_B_  S_C_ |
| **H_sp_CLC_148_c0_g1_i1^3^**  (aa13-134) | Similar to BPTI/Kunitz domain-containing protein (fragment); domain: Kunitz_BPTI (aa16-78,80-135); SSP (aa1-20); 10.2% R, 11.0% C, 11.0% G, 10.1% L; pI 8.8; IDR (14.4%; aa37-54) | KCP_HALAI**^4^**  (aa1-122) | 82.8 | 2.8e-50 | 7  7  7  8  9  7 | 41  33  36  107  94  62 | 2.048  2.819  3.650  0.409  0.539  0.446 | I_A_  I_B_  I_C_  S_A_  S_B_  S_C_ |
| **H_sp_CLC_77_c0_g1_i1^3^**  (aa13-137) | Similar to BPTI/Kunitz domain-containing protein (fragment); domain: Kunitz_BPTI (aa16-78,80-133); SSP (aa1-20); 11.0% R, 11.0% C, 10.2% G; pI 9.2 | KCP_HALAI**^4^**  (aa1-125) | 83.2 | 5.2e-51 | 8  -  -  12  -  - | 41  -  -  186  -  - | 4.953  -  -  2.193  -  - | I_A_  I_B_  I_C_  S_A_  S_B_  S_C_ |
| **H_sp_idb_26484_c0_g1_i1^3^**  (aa1-199)  Comp85406_c1_seq2_1  Comp85406_c0_seq1_2 | Similar to peptidyl-prolyl cis-trans isomerase; domain: cyclophilin_type_PPI (aa30-187); SSP (aa1-20); 13.9% G, 10.0% T; pI 4.5; IDR (14.4%; aa174-200) | A0A016S171_  9BILA  (aa1-197) | 67.0 | 3.4e-57 | 2  4  3  2  2  - | 3  14  8  13  19  - | 0.020  0.043  0.019  0.004  0.007  - | I_A_  I_B_  I_C_  S_A_  S_B_  S_C_ |
| **H_sp_CLC_1642_c0_g1_i1^3^**  (aa31-201)  **Comp85674_c0_seq1_1**  **Comp85674_c0_seq2_1** | Similar to aragonite protein AP24; TM (aa36-55); pI 7.0; IDR (12.4%; aa152-176) | Q9BP38_HALRU**^4^**  (aa1-171) | 77.8 | 3.4e-67 | 12  10  13  14  11  13 | 37  38  68  154  82  88 | 0.411  0.252  0.336  0.234  0.171  0.190 | I_A_  I_B_  I_C_  S_A_  S_B_  S_C_ |
| **H_sp_idb_47982_c0_g1_i1^3^**  (aa38-303)  **Comp86516_c0_seq2_3** | Uncharacterized; domain: CAP (aa62-234); SSP (aa1-18) or TM (aa13-35); pI 7.1; if SSP: pI 6.7 | V3ZVE9_LOTGI  Lotgidraft_160631  (aa51-314) | 50.0 | 1.0e-47 | -  -  -  2  -  2 | -  -  -  6  -  8 | -  -  -  0.001  -  0.001 | I_A_  I_B_  I_C_  S_A_  S_B_  S_C_ |
| **H_sp_Tri_53798_c0_g1_i1^3^**  (aa6-142)  **H_sp_idb_13782_c0_g1_i1**  **Comp87007_c0_seq1_6** | Similar to leukocyte cell-derived chemotaxin 1-like protein; domain: Brichos (aa66-148); SSP (aa1-22) or TM (aa4-26); 9.6% A, 11.5% V; pI 9.3; if SSP: pI 9.4; 10.2% V; (1 peptide not in this region); see also Tri_23898 and comp94109_c0_seq16_5 | D5FW85_HALDI  (aa3-138) | 27.3 | 5.5e-5 | -  -  -  -  4  4 | -  -  -  -  11  6 | -  -  -  -  0.009  0.003 | I_A_  I_B_  I_C_  S_A_  S_B_  S_C_ |
| **H_sp_CLC_4146_c0_g1_i1^3^**  (aa4-343)  **Comp87152_c0_seq1_4** | Similar to endochitinase; domains: VWA (aa9-293), chitin-bd_II (aa239-293,294-347); SSP (aa1-17); pI 5.5 | K1R034_CRAGI  (aa7-350) | 36.8 | 1.3e-43 | -  9  8  10  12  7 | -  25  32  32  48  25 | -  0.053  0.060  0.026  0.056  0.032 | I_A_  I_B_  I_C_  S_A_  S_B_  S_C_ |
| **Comp88085_c0_seq2_2^3^**  (aa18-430)  **Comp88085_c0_seq1_1** | Similar to α-tubulin/putative tubulin alpha-1b chain-like protein; domains: Tubulin_FtsZ_GTPase (aa32-242), Tub_FtsZ_C (aa275-430); pI 6.1; see also Comp103717_c1_seq2_5 | K9ISP8_DESRO  (aa4-416) | 99.5 | 4.0e-186 | 3  2  2  2  7  - | 4  4  5  11  23  - | 0.006  0.008  0.007  0.004  0.009  - | I_A_  I_B_  I_C_  S_A_  S_B_  S_C_ |
| **Comp88250_c0_seq2_2^3^** | Uncharacterized; TM (aa15-34); 9.6% S; pI 7.1; IDR (20.7%; aa1-12,39-57) |  |  |  | 4  6  4  3  4  3 | 30  36  35  60  34  20 | 6.096  2.351  1.675  0.061  0.111  0.038 | I_A_  I_B_  I_C_  S_A_  S_B_  S_C_ |
| **H_sp_Tri_97777_c0_g1_i1^3^**  (aa1-189)  **Comp88441_c0_seq3_1**  **Comp88441_c0_seq2_1** | Peptidyl-prolyl cis-trans isomerase (Fragment); domain: cyclophilin_like (aa26-194) SSP (aa1-18); 13.7% G, 13.7% K; pI 9.3; IDR (7.4%; aa194-208) | A0A0H4Q136  _HALDH  (aa1-189) | 94.2 | 1.3e-74 | -  -  -  7  6  5 | -  -  -  27  30  14 | -  -  -  0.015  0.023  0.011 | I_A_  I_B_  I_C_  S_A_  S_B_  S_C_ |
| **H_sp_CLC_5354_c0_g1_i1^3^**  (aa1-146)  **Comp91372_c0_seq1_1** | Peptidyl-prolyl cis-trans isomerase; domain: PPI_FKBP_type (aa48-141); SSP (aa1-21); 13.6% G, 14.4% K; pI 8.8 | K1R1I0_CRAGI  (aa1-145) | 76.9 | 1.1e-45 | -  -  3  4  4  4 | -  -  3  10  16  18 | -  -  0.005  0.013  0.015  0.025 | I_A_  I_B_  I_C_  S_A_  S_B_  S_C_ |
| **H_sp_idb_15781_c0_g1_i1^3^**  (aa32-167)  **Comp91382_c0_seq1_6** | Uncharacterized; domain: Cyt_B5-like_heme/steroid-bd (aa47-147); TM (aa10-32); 9.0% A, 10.8% L, 9.6% K, 9.6% V; pI 5.6; IDR (8.4%; aa154-166) | A0A0B7AZJ8  _9EUPU  (aa29-161) | 59.9 | 7.9e-30 | -  -  -  4  2  3 | -  -  -  12  3  7 | -  -  -  0.004  0.001  0.006 | I_A_  I_B_  I_C_  S_A_  S_B_  S_C_ |
| **H_sp_idb_39359_c0_g1_i1**  (aa29-215)  (aa220-350)  **Comp91520_c0_seq1_2** | Uncharacterized; domains: DUF4773 (aa65-182,222-334); SSP (aa1-32); 10.7% L, 11.0% V; pI 8.2; IDR (7.2%; aa191-214) | K1RDG1_CRAGI  (aa5-189)  (aa43-174) | 46.6  42.0 | 2.0e-33  1.1e-25 | -  -  -  6  4  - | -  -  -  7  9  - | -  -  -  0.003  0.003  - | I_A_  I_B_  I_C_  S_A_  S_B_  S_C_ |
| **Comp91920_c0_seq3_1** | Uncharacterized; 10.3% R, pI 9.3 |  |  |  | -  -  -  5  4  4 | -  -  -  20  14  15 | -  -  -  0.025  0.012  0.011 | I_A_  I_B_  I_C_  S_A_  S_B_  S_C_ |
| **Comp92088_c0_seq1_4^3^**  (aa118-215) | Uncharacterized; 9.0% C, 9.0% T; pI 8.4; IDR (5.8%; aa1-13); shares most peptides with Tri_3731 | V4ALS8_LOTGI  Lotgidraft_239117  (aa118-216) | 80.6 | 1.6e-12 | -  -  -  4  6  - | -  -  -  13  24  - | -  -  -  0.004  0.010  - | I_A_  I_B_  I_C_  S_A_  S_B_  S_C_ |
| **H_sp_Tri_3731_c0_g1_i1^3^**  (aa41-138) | Uncharacterized; SSP (aa1-30)/TM (aa5-27); 10.6% C; pI 7.3, if SSP: 12.6% C; pI 7.6; shares most peptides with comp92088_c0_seq1_4 | V4ALS8_LOTGI  Lotgidraft_239117  (aa118-216) | 41.4 | 1.4e-12 | -  -  -  -  -  5 | -  -  -  -  -  14 | -  -  -  -  -  0.009 | I_A_  I_B_  I_C_  S_A_  S_B_  S_C_ |
| **H_sp_Tri_129647_c0_g1_i1^3^**  (aa103-805)  **Comp92139_c0_seq1_6**  **Comp92139_c0_seq2_6** | Similar to latrophilin-3; domains: GAIN_dom_N (268-463), GPS (aa491-542), GPCR_2-like (aa552-796); multiple TM in aa555-793); 10.8% S, 12.8% T; pI 6.5; IDR (25.2%; aa1-188,809-840) | A0A210PIR3_  MIZYE  (aa156-943) | 42.1 | 8.3e-75 | 3  2  8  11  9  10 | 5  3  27  102  58  95 | 0.007  0.002  0.014  0.010  0.006  0.019 | I_A_  I_B_  I_C_  S_A_  S_B_  S_C_ |
| **Comp92223_c1_seq1_1^3^**  (aa53-437)  **H_sp_idb_2286_c0_g1_i1** | Uncharacterized; domain: EF_hand_pair (aa250-347); TM (aa30-47); 12.7% Q, 11.4% E; pI 5.1; Q-rich C-term; IDP | A0A0B7AA85_  9EUPU  (aa22-394) | 57.3 | 6.5e-49 | 6  8  7  8  5  3 | 9  25  24  62  46  22 | 0.015  0.037  0.028  0.009  0.008  0.005 | I_A_  I_B_  I_C_  S_A_  S_B_  S_C_ |
| **H_sp_Tri_12530_c0_g1_i1**  (aa126-473)  **Comp92337_c0_seq3_1**  **Comp92337_c0_seq1_2** | Uncharacterized; domain: Ser-Thr/Tyr_kinase_cat (aa125-409); TM (aa13-35); pI 7.6; IDR (8.7%; aa89-118,418-493) | V3ZKZ9_LOTGI  Lotgidraft_62659  (aa1-347) | 65.8 | 7.6e-105 | -  2  -  3  -  - | -  4  -  9  -  - | -  0.002  -  0.001  -  - | I_A_  I_B_  I_C_  S_A_  S_B_  S_C_ |
| **H_sp_idb_10147_c0_g1_i1**  (aa1-283)  **Comp92459_c0_seq7_1**  **etc** | Uncharacterized; domains: galactose-bd_like/FA58C_1 (aa24-179, 180-288); SSP (aa1-21)/TM (aa7-29); 10.7% T; pI 9.0; if SSP: 11.1% S; pI 9.1 | A0A0B6YGS6  _9EUPU  (aa8-291) | 77.1 | 4.5e-110 | -  -  3  3  -  - | -  -  5  7  -  - | -  -  0.005  <0.001  -  - | I_A_  I_B_  I_C_  S_A_  S_B_  S_C_ |
| **H_sp_idb_18825_c0_g1_i1**  (aa1-467)  **H_sp_idb_18822_c0_g1_i1^3^**  **Comp92750_c0_seq4_1**  **etc** | Similar to ezrin/radixin/moesin; domains: FERM (aa5-295); 15.0% E, 10.5% L; pI 5.3; IDR (46.3%; aa299-555) | R7UYN1_CAPTE  (aa1-460) | 69.2 | 2.5e-62 | -  2  7  -  -  - | -  2  12  -  -  - | -  0.001  0.004  -  -  - | I_A_  I_B_  I_C_  S_A_  S_B_  S_C_ |
| **H_sp_Tri_47901_c0_g1_i1^3^**  (aa16-363)  **Comp92949_c2_seq16_6**  **etc** | Similar to glycoprotein-N-acetyl-galactosamine 3-beta-galactosyl-transferase 1; domain: C1GALT1 (aa173-297); TM (aa13-30); pI 8.3; IDR (23.0%; aa1-11,33-103,359-391) | K1PSC7_CRAGI  (aa31-370) | 62.0 | 4.5e-98 | 4  6  4  9  4  - | 12  10  11  47  21  - | 0.042  0.032  0.018  0.017  0.016  - | I_A_  I_B_  I_C_  S_A_  S_B_  S_C_ |
| **H_sp_idb_11270_c0_g1_i1^3^**  (aa1-633)  **Comp93111_c0_seq1_5** | Similar to HSP70; domains: HSP70_peptide_binding (aa388-544), HSP70_Cterm (aa519-622); pI 5.5; IDR (23.4%; aa490-633); shares several peptides with comp79549_c0_seq1_2, and Tri_2770 | S4U403_9GAST  (aa1-635) | 79.8 | 3.4e-300 | -  -  -  2  5  - | -  -  -  11  7  - | -  -  -  0.001  0.001  - | I_A_  I_B_  I_C_  S_A_  S_B_  S_C_ |
| **H_sp_Tri_3904_c0_g1_i1^3^**  (aa23-647)  **Comp93346_c0_seq1_3**  **Comp93346_c0_seq2_2** | Similar to angiotensin-converting enzyme; domain: peptidase_M2_fam (aa70-647), SSP (aa1-20); pI 4.9; IDR (6.1%; aa38-73) | C3Z9J8_BRAFL  (aa38-666) | 47.2 | 5.7e-126 | 3  3  3  -  -  - | 4  4  5  -  -  - | 0.004  0.002  0.001  -  -  - | I_A_  I_B_  I_C_  S_A_  S_B_  S_C_ |
| **H_sp_CLC_4246_c0_g1_i1**  (aa36-318)  **Comp93938_c0_seq2_4** | Uncharacterized; domain: FkbM_mtfrase (aa138-296); TM (aa39-61); 10.3% L; pI 9.2 | E9GEJ2_DAPPU  (aa3-275) | 30.8 | 6.7e-24 | -  2  -  2  2  - | -  5  -  8  5  - | -  0.006  -  0.001  0.001  - | I_A_  I_B_  I_C_  S_A_  S_B_  S_C_ |
| **H_sp_Tri_8133_c0_g1_i1^3^**  (aa3-456)  **Comp93947_c0_seq9_3**  **etc** | Similar to intermediate filament protein; domains: Intermed_filament_ifa/ifb (aa1-597); 9.5% L, 9.3% S; pI 5.6; IDR (37.2%; aa1-85,294-352,426-489,505-512,593-599) | V4CQU1_LOTGI**^4^**  Lotgidraft_109284  (aa6-457) | 51.8 | 7.5e-63 | -  3  15  -  -  - | -  4  31  -  -  - | -  0.002  0.017  -  -  - | I_A_  I_B_  I_C_  S_A_  S_B_  S_C_ |
| **H_sp_Tri_91049_c0_g1_i1^3^**  (aa1-363  **Comp93951_c0_seq1_3**  B6RB97_HALDI | Uncharacterized; domains: villin/gelsolin (aa67-193,194-303,304-395); 11.3% E; pI 4.6; IDR (15.7%; aa1-40,254-270) | R7U815_CAPTE  (aa1-365) | 58.3 | 2.6e-78 | 4  6  7  6  -  - | 7  13  17  11  -  - | 0.012  0.018  0.014  0.001  -  - | I_A_  I_B_  I_C_  S_A_  S_B_  S_C_ |
| **Comp94109_c0_seq16_5**  (aa87-244) | Similar to leukocyte cell-derived chemotaxin 1-like protein; domain: Brichos (aa141-234); SSP (aa1-18)??; pI 8.7; IDR (3.7%; aa192-204); if SSP: pI 8.6; IDR 3.6%; see also Tri_23898 and Tri_53798 | D5FW85_HALDI  (aa1-156) | 62.0 | 4.2e-43 | -  -  5  -  -  3 | -  -  19  -  -  18 | -  -  0.198  -  -  0.068 | I_A_  I_B_  I_C_  S_A_  S_B_  S_C_ |
| **H_sp_idb_32872_c0_g1_i1^3^**  (aa41-241)  **Comp94706_c0_seq1_3** | Uncharacterized; SSP (aa1-19); pI 8.7 | V3ZY26_LOTGI**^4^**  Lotgidraft_154423  (aa10-212) | 34.1 | 7.3e-24 | -  6  5  -  -  - | -  17  15  -  -  - | -  0.063  0.033  -  -  - | I_A_  I_B_  I_C_  S_A_  S_B_  S_C_ |
| **Comp95780_c0_seq2_4^3^** | Uncharacterized; TM (aa29-51,187-209); pI 10.0 |  |  |  | -  -  2  -  2  5 | -  -  19  -  23  60 | -  -  0.116  -  0.028  0.126 | I_A_  I_B_  I_C_  S_A_  S_B_  S_C_ |
| **Comp96038_c1_seq3_1**  (aa25-130) | Uncharacterized; domain: tubulin-binding_cofactor_A_fam (TBCA; aa28-127); SSP (aa1-19)?; 10.7% E, 9.8% L; pI 5.2; with SSP: 11.5% E; pI 5.1 | A0A0L8G2J3  _OCTBM  (aa28-127) | 55.7 | 6.8e-15 | -  -  -  3  3  - | -  -  -  25  9  - | -  -  -  0.007  0.004  - | I_A_  I_B_  I_C_  S_A_  S_B_  S_C_ |
| **H_sp_Tri_108314_c0_g1_i1**  (aa10-334)  **Comp96364_c0_seq22_5** | Similar to cathepsin L; domains: Prot_inhib_I29 (aa32-92), Peptidase_C1A_C (aa118-333); SSP (aa1-25); 10.4% G; pI 5.7; IDR (2.9%; aa104-113) | V4AKY5_LOTGI  Lotgidraft_208974  (aa1-328) | 67.1 | 4.3e-105 | -  -  -  2  2  - | -  -  -  4  2  - | -  -  -  0.002  <0.001  - | I_A_  I_B_  I_C_  S_A_  S_B_  S_C_ |
| **Comp97143_c2_seq1_6**  (aa47-276)  **H_sp_Tri**_**80461_c0_g1_i1** | Similar to Low-density lipoprotein receptor-related protein; domain: 6-blade_b-propeller_TolB-like (aa32-266); SSP (aa1-19); pI 9.2 | W6AC86_ICTPU  (aa1370-1616) | 25.8 | 5.8e-5 | 2  2  -  -  -  - | 3  5  -  -  -  - | 0.004  0.004  -  -  -  - | I_A_  I_B_  I_C_  S_A_  S_B_  S_C_ |
| **H_sp_Tri_137490_c0_g1_i1^3^**  (aa18-139)  **Comp97298_c0_seq1_5** | Similar to calcium-binding protein; domains: EF_hand (aa9-44,45-77,84-115); 13.7% D, 9.6% E; pI 4.3; IDR (11.0%; aa1-11,142-146) | B0EE99_ENTDS  (aa6-130) | 30.2 | 2.8e-4 | 4  4  4  3  -  - | 6  14  19  5  -  - | 0.059  0.109  0.124  0.005  -  - | I_A_  I_B_  I_C_  S_A_  S_B_  S_C_ |
| **H_sp_CLC_9082_c0_g1_i1**  (aa82-357)  **Comp97769_c0_seq11_2**  **Comp97769_c0_seq3_1**  **Comp97769_c0_seq10_2** | Uncharacterized; domain: Nucleotide-diphossugar_trans (aa109-268); TM (aa5-27); pI 7.7 | T1EYE7_HELRO  (aa104-381) | 39.2 | 2.5e-41 | 4  3  3  3  -  - | 12  12  6  44  -  - | 0.063  0.022  0.006  0.013  -  - | I_A_  I_B_  I_C_  S_A_  S_B_  S_C_ |
| **H_sp_Tri_52786_c0_g1_i1^3^**  (aa17-224)  **Comp98044_c0_seq2_2** | Uncharacterized; domain: PLipase_A2 (aa111-232); SSP (aa1-26); pI 7.9; IDR (4.2%; aa94-103) | T1INX7_STRMM  (aa10-213) | 35.8 | 1.1e-24 | 3  3  4  12  7  4 | 7  9  13  47  15  9 | 0.044  0.022  0.020  0.021  0.029  0.028 | I_A_  I_B_  I_C_  S_A_  S_B_  S_C_ |
| **H_sp_Tri_87730_c0_g1_i1**  (aa1-216)  **Comp98408_c0_seq1_5**  **Comp98408_c0_seq3_4** | Uncharacterized; domain: Ran_GTPase (aa4-214); pI 5.6; IDR (19.4%; aa175-216) | V4AD07_LOTGI**^4^**  Lotgidraft_225073  (aa1-216) | 95.8 | 4.8e-70 | -  -  3  2  -  - | -  -  3  3  -  - | -  -  0.004  <0.001  -  - | I_A_  I_B_  I_C_  S_A_  S_B_  S_C_ |
| **H_sp_Tri_123348_c0_g1_i1**  (aa145-440)  **Comp98866_c0_seq1_1**  **Comp98866_c0_seq2_3** | Similar to sulfotransferase; domain: sulfotransferase (aa180-440); TM (aa13-35); pI 9.6; IDR (33.2%; aa58-185,389-406) | R7VCQ2_CAPTE  (aa72-364) | 67.1 | 3.6e-89 | -  -  -  10  7  4 | -  -  -  58  41  8 | -  -  -  0.013  0.009  0.002 | I_A_  I_B_  I_C_  S_A_  S_B_  S_C_ |
| **H_sp_idb_9026_c0_g1_i1^3^**  (aa28-351)  **Comp99181_c0_seq1_4**  **H_sp_idb_9027_c0_g1_i1** | Uncharacterized; SSP (aa1-22)/TM (aa5-22); pI 4.8; IDR (2.8%; aa468-481); if SSP: pI 4.8; IDR 2.7% | V4BEK2_LOTGI  Lotgidraft_171050  (aa1-332) | 25.2 | 5.1e-10 | -  -  -  2  2  - | -  -  -  4  2  - | -  -  -  <0.001  <0.001  - | I_A_  I_B_  I_C_  S_A_  S_B_  S_C_ |
| **H_sp_idb_19419_c0_g1_i1**  (aa11-595)  **Comp99331_c0_seq3_6**  **etc** | Similar to alpha-actinin; domains: CH (aa6-250), spectrin-repeat (aa273-380,392-496,510-611); 9.7% E, 11.0% L; pI 5.7; IDR (3.0%; aa1-19) | V3ZQ63_LOTGI  Lotgidraft_189716  (aa1-585) | 89.7 | 0e0 | -  -  3  -  2  - | -  -  4  -  2  - | -  -  0.001  -  <0.001  - | I_A_  I_B_  I_C_  S_A_  S_B_  S_C_ |
| **H_sp_Tri_100799_c0_g1_i1**  (aa3-262)  **Comp99476_c0_seq1_3** | Uncharacterized; domain: JmjC (aa140-264); SSP (aa1-15); pI 5.5 | A0A0B7B6Q7  _9EUPU  (aa22-281) | 66.2 | 3.0e-82 | -  -  -  2  2  - | -  -  -  6  2  - | -  -  -  0.001  0.001  - | I_A_  I_B_  I_C_  S_A_  S_B_  S_C_ |
| **H_sp_CLC_1125_c0_g1_i1^3^**  (aa10-345)  (aa11-850) | Similar to chitin-binding protein; domain: Cellulose/chitin-bd_N (aa28-214); SSP (aa1-27)/TM (aa5-27); aa510-610 T-rich (56%); pI 8.3; IDR (62.8%; aa263-870); if SSP: 9.5% P, 13.7% T; pI 8.5; IDR: 64.3% | A0A075LZK8_  PINMT  (aa5-362)  K1QE07_CRAGI  (aa1-837) | 42.1  23.8 | 1.2e-26  9.5e-19 | 22  23  26  30  29  27 | 81  95  119  340  251  212 | 0.140  0.125  0.171  0.104  0.080  0.129 | I_A_  I_B_  I_C_  S_A_  S_B_  S_C_ |
| **H_sp_CLC_12_c0_g1_i1^3^**  (aa45-461) | Uncharacterized; 9.7% T; pI 6.1; IDR (9.7%; aa31-49,438-463) | V4AKF1_LOTGI  Lotgidraft_228685  (aa30-466) | 29.8 | 1.1e-22 | 6  6  5  7  3  3 | 11  16  19  34  24  18 | 0.037  0.064  0.049  0.012  0.008  0.006 | I_A_  I_B_  I_C_  S_A_  S_B_  S_C_ |
| **H_sp_CLC_12027_c0_g1_i1^3^**  (aa81-180)  **H_sp_idb_54497_c0_g1_i1** | Uncharacterized; SSP (aa1-19)/TM (aa7-29), TM (aa65-87); 18.3% G, 14.1% M, 10.0% P, pI 9.4; IDR (57.8%; aa8-111); tandem repeats (Fig. S2A); if SSP: 19.8 % G, 15.4% M, 9.9% P; pI 9.6; IDR: 63.9% |  |  |  | 6  8  9  14  11  13 | 17  32  47  303  202  249 | 0.212  0.592  0.747  5.298  6.692  11.716 | I_A_  I_B_  I_C_  S_A_  S_B_  S_C_ |
| **H_sp_CLC_123_c0_g1_i1^3^**  (aa2-455)  **H_sp_idb_32947_c0_g1_i1** | Similar to tyrosinase; domains: Tyrosinase_Cu-bd (aa18-271); SSP (aa1-20); 14.6% G; pI 9.0; IDR (24.5%; aa350-457); tandem repeats (Fig. S2B) | V4AN59_LOTGI  Lotgidraft_160808  (aa13-518) | 43.3 | 3.0e-38 | 6  7  7  4  4  3 | 37  57  60  85  31  31 | 0.813  0.791  0.682  0.052  0.015  0.024 | I_A_  I_B_  I_C_  S_A_  S_B_  S_C_ |
| **H_sp_CLC_1320_c0_g1_i1^3^**  (aa1-133)  **H_sp_Tri_133893_c0_g1_i1**  **H_sp_Tri_133894_c0_g1_i1** | Lustrin A (fragment); domain: Lustrin_cystein (aa67-108); SSP (aa1-19); 10.3% C, 24.8% P; pI 6.7; C-term IDR (20.6%; aa109-136); tandem repeats aa115-136 (PPA)_7_ | O44341_HALRU**^4^**  (aa1-133) | 76.7 | 7.3e-23 | 4  6  6  6  5  4 | 17  45  39  85  81  56 | 0.606  1.190  0.773  0.146  0.408  0.260 | I_A_  I_B_  I_C_  S_A_  S_B_  S_C_ |
| **H_sp_Tri_116352_c0_g1_i1^3^**  (aa16-652) | Similar to lustrin A; domains: multiple Cys_repeat_1; 11.0% C, 19.6% P; pI 6.5; IDR (10.1%; aa1-40,123-139); shares several peptides with idb_288, Tri_116349 | O44341_HALRU**^4^**  (aa226-872) | 62.6 | 1.3e-103 | 12  12  14  9  6  5 | 45  57  73  95  63  34 | 0.152  0.243  0.413  0.027  0.049  0.044 | I_A_  I_B_  I_C_  S_A_  S_B_  S_C_ |
| **H_sp_idb_288_c0_g1_i1**  (aa1-207) | Lustrin A; shares several peptides with Tri_116352; domains: lustrin_cys-rich_repeat (aa34-70,134-176); 11.6% C, 17.4% P; pI 8.3; IDR (9.7%; aa188-207); O44341_HALRU: aa682-899 | A0A088CBA1  _HALTU  (aa121-324) | 76.4 | 4.4e-60 | -  -  5  -  -  - | -  -  29  -  -  - | -  -  0.371  -  -  - | I_A_  I_B_  I_C_  S_A_  S_B_  S_C_ |
| **H_sp_CLC_608_c0_g1_i1^3^**  (aa1-179) | Lustrin A (fragment); O44341_HALRU aa1175-1362; 16.1% G, 26.1% S; pI 4.5; IDR (56.1%; aa1-101) | F6KD05_HALTU  (aa205-380) | 78.3 | 8.1e-28 | 2  3  2  2  2  2 | 10  9  8  28  27  20 | 0.492  0.462  0.776  0.053  0.073  0.064 | I_A_  I_B_  I_C_  S_A_  S_B_  S_C_ |
| **H_sp_CLC_14282_c0_g1_i1^3^**  (aa136-654)  Comp104776_c0_seq5_3 | Polypeptide N-acetylgalactosaminyl transferase; domains: Nucleotide-diphossugar_trans (aa187-517); ricin_B_lectin (aa523-648); TM (aa45-67); pI 8.1; IDR (21.5%; aa1-35,72-177) | V3ZR56_LOTGI  Lotgidraft_194702  (aa1-519) | 75.3 | 3.7e-196 | -  4  5  -  -  - | -  8  12  -  -  - | -  0.007  0.005  -  -  - | I_A_  I_B_  I_C_  S_A_  S_B_  S_C_ |
| **H_sp_CLC_146_c0_g1_i1^3^** | Uncharacterized; domains: VWA (aa50-236), ConA_like (aa712-884); 14.0% S, 13.6% T; pI 8.8; IDR (18.8%; aa321-421,549-572,595-632) |  |  |  | 15  17  20  15  9  6 | 41  56  105  55  32  15 | 0.149  0.103  0.183  0.007  0.003  0.004 | I_A_  I_B_  I_C_  S_A_  S_B_  S_C_ |
| **H_sp_CLC_15249_c0_g1_i1^3^**  (aa35-337)  Comp82445_c0_seq1_6 | Uncharacterized; SSP (aa1-32)/TM (aa5-27); pI 4.8 | V4AKU8_LOTGI  Lotgidraft_228332  (aa25-334) | 22.1 | 1.9e-5 | -  2  6  6  5  10 | -  2  15  26  15  28 | -  0.001  0.026  0.006  0.006  0.013 | I_A_  I_B_  I_C_  S_A_  S_B_  S_C_ |
| **H_sp_CLC_18633_c0_g1_i1^3^**  (aa12-222) | Uncharacterized; domain: Chitin-bd_N (aa32-220); TM (aa7-26); 13.3% T; pI 8.5; IDR (31.7%; aa221-364) | V3ZNR4_LOTGI  Lotgidraft_119849  (aa4-211) | 51.2 | 1.6e-27 | -  -  2  6  5  - | -  -  5  10  12  - | -  -  0.002  0.001  0.002  - | I_A_  I_B_  I_C_  S_A_  S_B_  S_C_ |
| **H_sp_CLC_1876_c0_g1_i1^3^**  (aa2-198) | Similar to ependymin-1; domain: ependymin (aa71-198); 11.8% L, 10.6% S; pI 7.7; shares several peptides with Tri_31892 and idb_40080 | EPDR1_HALAI**^4^**  (aa1-198) | 71.2 | 1.6e-65 | -  -  -  -  2  - | -  -  -  -  4  - | -  -  -  -  0.002  - | I_A_  I_B_  I_C_  S_A_  S_B_  S_C_ |
| **H_sp_CLC_1949_c0_g1_i1^3^** | Uncharacterized; 10.9% R, 17.5% Q, 10.5% S; pI 11.6, IDP; shares several peptides with idb_12174 and CLC_8754 |  |  |  | -  -  2  1  2  2 | -  -  7  7  17  15 | -  -  0.002  <0.001  0.002  0.003 | I_A_  I_B_  I_C_  S_A_  S_B_  S_C_ |
| **H_sp_idb_12174_c0_g1_i1^3^** | Uncharacterized; TM (aa12-31); 20.0% Q,11.5%S, pI 10.6; IDP; shares several peptides with CLC_1949 |  |  |  | -  -  6  7  8  14 | -  -  11  42  76  97 | -  -  0.007  0.003  0.008  0.032 | I_A_  I_B_  I_C_  S_A_  S_B_  S_C_ |
| **H_sp_Tri_107535_c0_g1_i1^3^**  (aa8-371)  **H_sp_CLC_21_c0_g1_i1**  GAAP_HALAI | Similar to glycine-, alanine- and asparagine-rich protein; 31.8% A, 17.8% G, 21.3% S; pI 12.0; IDR (20.2%; aa1-34,331-371); repeats (Fig. S2C) | GAAP_HALAI**^4^**  (aa30-443) | 77.5 | 3.0e-44 | 7  4  10  9  10  4 | 43  37  339  115  123  32 | 0.780  0.473  2.723  0.076  0.071  0.026 | I_A_  I_B_  I_C_  S_A_  S_B_  S_C_ |
| **H_sp_CLC_2296_c0_g1_i1^3^**  (aa6-674)  Comp292963_c0_seq1_2 | Similar to chitinase-3; domains: chitinase_II (aa34-408); chitin-bd_II (aa468-531,619-675); SSP (aa1-31)/TM (aa7-29); 9.8% T; pI 8.5; IDR (23.7; aa412-475,523-618); if SSP: 10.2% T; pI 8.5; IDR: 24.5% | J7FIC1_HYRCU  (aa2-651) | 45.7 | 9.8e-86 | 13  15  16  18  13  5 | 38  51  84  103  56  18 | 0.083  0.054  0.071  0.020  0.009  0.004 | I_A_  I_B_  I_C_  S_A_  S_B_  S_C_ |
| **H_sp_CLC_2347_c0_g1_i1**  (aa233-613)  **H_sp_idb_28940_c0_g1_i1**  Comp238950_c0_seq1_2 | Uncharacterized; domains: ARM_like (aa15-41), chitin-bd_II (aa257-326,323-372), ConA-like (aa438-611); 10.0% A, 9.2% D, 10.2% S, 16.0% T; pI 4.3; IDR (43.2%; aa1-268); [D]_28_ (aa13-40) | V4ARZ6_LOTGI**^4^**  Lotgidraft_228264  (aa223-606) | 36.6 | 3.0e-16 | 2  7  5  9  8  - | 4  14  16  23  22  - | 0.021  0.039  0.028  0.010  0.010  - | I_A_  I_B_  I_C_  S_A_  S_B_  S_C_ |
| **H_sp_CLC_253_c0_g1_i1**  (aa128-218) | Similar to glutamine-rich protein (QRP); 28.0% Q, 9.2% T; pI 11.2; peptides from aa157 to end; IDR (53.5%; aa99-205); repeats (Fig. S2D) | QRP_HALAI**^4^**  (aa6-117) | 57.1 | 4.3e-5 | 3  3  4  -  2  - | 20  22  34  -  5  - | 0.795  0.461  0.283  -  0.003  - | I_A_  I_B_  I_C_  S_A_  S_B_  S_C_ |
| **H_sp_idb_2023_c0_g1_i1^3^**  (aa13-397)  **H_sp_CLC_2607_c0_g1_i1**  **H_sp_idb_2021_c0_g1_i1** | Uncharacterized; domains: IG (120-235), chitin-bd_II (aa347-404); pI 5.5; 10.1% S, 9.4% T; IDR (4.5%; aa39-56) | V3ZHM8_LOTGI  Lotgidraft_236408  (aa136-503) | 22.3 | 2.6e-13 | -  -  -  2  2  - | -  -  -  5  4  - | -  -  -  0.001  0.003  - | I_A_  I_B_  I_C_  S_A_  S_B_  S_C_ |
| **H_sp_CLC_303_c0_g1_i1^3^** | Uncharacterized; aa615-745 almost identical to hasininaP0013F12_631**^4^**; domains: chitin_bd_II (aa350-410,411-475), ConA-like (aa584-731); 21.3% A, 9.8% Q; pI 6.7; IDR (48.7%; aa1-363); repeats (Fig. S2E) |  |  |  | 23  27  34  24  22  10 | 83  124  218  336  221  104 | 0.635  0.619  1.136  0.086  0.066  0.043 | I_A_  I_B_  I_C_  S_A_  S_B_  S_C_ |
| **H_sp_CLC_3466_c0_g1_i1^3^**  (aa156-679)  Comp16849_c0_seq2_2 | Similar to metalloendopeptidase; domains: CUB (aa403-518), TSP1 (aa582-632); SSP (aa1-19); pI 8.6; IDR (1.5%; aa117-127) | A0A077YYY8  _TRITR  (aa92-556) | 31.2 | 1.5e-42 | 3  2  5  3  -  - | 9  3  14  7  -  - | 0.031  0.005  0.009  0.001  -  - | I_A_  I_B_  I_C_  S_A_  S_B_  S_C_ |
| **H_sp_CLC_3878_c0_g1_i1^3^**  (aa8-440)  (aa38-804) | Uncharacterized/IgGFc-binding protein; domains: chitin-bd_II (aa32-90,96-163,236-300,325-374), Sushi_SCR_CCP (aa475-534,733-792,946-1004), galectin_CRD (aa1134-1289), FA58C_3 (aa1134-1289); SSP (aa1-20); 9.3% S, 10.2% T; pI 6.1; shares several peptides with Tri_120379 and idb_26484/idb_2772; IDR (11.8%; aa393-433,660-742,779-800) | K1QJK2_CRAGI  (aa13-458)  K1PRD3_CRAGI  (aa1235-2010) | 28.2  24.2 | 6.3e-16  1.7e-10 | 1  3  4  2  2  2 | 3  7  10  12  9  9 | 0.002  0.004  0.006  0.001  0.001  0.001 | I_A_  I_B_  I_C_  S_A_  S_B_  S_C_ |
| **H_sp_idb_2768_c0_g1_i1^3^**  (aa40-1286) | Uncharacterized; domains: fucolectin/tachylectin-4/pentraxin-1 (FTP1; aa57-205,860-1007), Sushi_SCR_CCP (aa284-346,659-719,1012-1072), galectin (aa353-480),  Gal_bd (aa492-647); pI 5.7; shares most peptides with Tri_120379; IDR (3.5%; aa492-501,710-725,1231-1249) | C3XSY6_BRAFL  (aa17-1131) | 24.7 | 1.9e-6 | 4  4  10  -  -  - | 8  10  30  -  -  - | 0.002  0.002  0.014  -  -  - | I_A_  I_B_  I_C_  S_A_  S_B_  S_C_ |
| **H_sp_idb_2772_c0_g1_i1^3^**  (aa2-440)  (aa38-804) | Uncharacterized; shares several peptides with Tri_120379, CLC_3878, Tri_120377 and idb_2768; domains: chitin-bd_II (aa32-90,96-163,236-300,325-374), Sushi_SCR_CCP (aa475-534,733-792,946-1004,1299-1359,1669-1727), galectin (aa802-928,1341-1493,1942-2089), FA58C_3 (aa1134-1289,1493-1655,2091-2244); SSP (aa1-20); pI 6.0; IDR (6.8%; aa392-432,661-738,1509-1533,1672-1680) | K1QJK2_CRAGI  (aa1-458)  K1PRD3_CRAGI  (aa1235-2010) | 28.9  24.2 | 1.9e-16  3.8e-11 | 17  9  13  4  6  2 | 26  20  48  15  12  11 | 0.010  0.004  0.018  <0.001  <0.001  0.001 | I_A_  I_B_  I_C_  S_A_  S_B_  S_C_ |
| **H_sp_Tri_120377_c0_g1_i1^3^** | Uncharacterized; domains: Sushi_SCR_CCP (aa30-88,235-295), Con_A_like (aa311-436), FA58C (aa453-607); shares several peptides with Tri_120379, idb_13824 and idb_2772; 10.3% V; pI 5.6 |  |  |  | -  -  4  -  -  - | -  -  6  -  -  - | -  -  0.006  -  -  - | I_A_  I_B_  I_C_  S_A_  S_B_  S_C_ |
| **H_sp_Tri_120379_c0_g1_i1^3^** | Uncharacterized; shares many peptides with idb_2772, idb_2768, CLC_3466, Tri_120377, and 1 with idb_13824; domains: multiple Sushi_SCR_CCP, Galactose-bd, FA58C, galectin_CRD, FTP1; 10.2% T; pI 5.6; IDR (8.1%; aa1-8,77-164,373-422,792-800,1666-1693,1753-1760) |  |  |  | 5  26  39  -  -  1 | 9  66  139  -  -  3 | 0.003  0.018  0.051  -  -  <0.001 | I_A_  I_B_  I_C_  S_A_  S_B_  S_C_ |
| **H_sp_CLC_39_c0_g1_i1^3^**  (aa1-186) | Similar to uncharacterized protein 3; SSP (aa1-21); 11.9% A, 11.3% L, 13.7% P; pI 10.7; IDR (32.7%; aa22-77); aa26-52 repeats [GPXPXAXLR]_3_ | UP3_HALAI**^4^**  (aa1-169) | 74.3 | 3.1e-34 | 12  13  13  21  19  18 | 115  148  165  1117  731  647 | 10.191  6.625  4.780  21.976  17.960  20.859 | I_A_  I_B_  I_C_  S_A_  S_B_  S_C_ |
| **UP3_HALAI^3^**  (P86737) | Uncharacterized protein 3; SSP (aa1-21), 10.1% A, 11.5% L, 11.5% P; pI 10.0; shares 1 peptide with CLC_39 |  |  |  | 2  2  2  4  4  4 | 8  14  15  170  118  114 | 0.099  0.013  0.052  0.900  1.277  1.256 | I_A_  I_B_  I_C_  S_A_  S_B_  S_C_ |
| **H_sp_CLC_4_c0_g1_i1^3^**  (aa20-67)  **H_sp_Tri_11338_c0_g1_i1** | Similar to cartilage matrix protein; aa1-165 similar to ML7A11**^4^**; SSP (aa1-18); 20.4% N, 11.2% D, 23.7% G; pI 5.3; (peptides in aa35-68; the rest is similar to DGRP; IDR (84.2%; aa26-46,62-170); repeats (Fig. S2F) | K1Q2S1_CRAGI  (aa344-391) | 52.1 | 1.3e-6 | 3  3  4  6  4  4 | 26  27  56  166  147  9 | 5.377  5.715  10.330  5.536  6.253  8.675 | I_A_  I_B_  I_C_  S_A_  S_B_  S_C_ |
| **H_sp_CLC_4517_c0_g1_i1^3^**  (aa12-210)  Comp94020_c0_seq1_3 | Similar to peptidyl-prolyl cis-trans isomerase FKBP14; domains: PPIase_FKBP (aa43-132), EF-hand-dom_pair (aa142-202); SSP (aa1-22); 13.3% D, 10.1% K; pI 4.6; IDR (40.4%; aa134-210) | V4B2Q8_LOTGI**^4^**  Lotgidraft_238515  (aa9-208) | 62.7 | 2.6e-47 | -  -  2  5  2  8 | -  -  4  8  7  23 | -  -  0.003  0.003  0.004  0.022 | I_A_  I_B_  I_C_  S_A_  S_B_  S_C_ |
| **H_sp_CLC_554_c0_g1_i1^3^** | Uncharacterized; domains: C-type_lectin-like (aa29-135,623-753), Ig-like (aa764-861); 12.5%S, 15.6% T; pI 6.7; IDR (58.5%; aa168-640,751-776,886-901) |  |  |  | 9  11  10  7  4  - | 22  30  31  20  6  - | 0.051  0.039  0.021  0.003  0.001  - | I_A_  I_B_  I_C_  S_A_  S_B_  S_C_ |
| **H_sp_Tri_33510_c0_g1_i1^3^**  **H_sp_CLC_62_c0_g1_i1** | Uncharacterized; SSP (aa1-21); 18.4% Q, 13.1% G, 15.0% P, pI 11.8; IDP; repeats (Fig. S2G) |  |  |  | 12  11  11  10  8  9 | 133  128  169  132  81  57 | 4.509  5.634  6.133  0.854  0.873  1.239 | I_A_  I_B_  I_C_  S_A_  S_B_  S_C_ |
| **H_sp_CLC_620_c0_g1_i1^3^**  (aa43-301)  **H_sp_idb_44242_c0_g1_i1**  Comp427051_c0_seq1_4 | Uncharacterized; SSP (aa1-22); TM (aa271-293); 10.2% S; pI 4.7; IDR (43.1%; aa66-84,311-350,362-520) | V4A4K4_LOTGI  Lotgidraft_234029  (aa32-300) | 26.1 | 7.2e-8 | -  -  -  4  3  - | -  -  -  22  21  - | -  -  -  0.021  0.061  - | I_A_  I_B_  I_C_  S_A_  S_B_  S_C_ |
| **H_sp_CLC_73_c0_g1_i1^3^**  (aa1-655) | Similar to collagen alpha 1 (V) chain;domain: collagen_triple_helix (aa13-247); 15.9% G, 15.7% P, 16.6% S; pI 11.4; IDR (62.3%; aa248-655); shares several peptides with idb_17035; Fig. S2H | A0A068WQE8_  ECHGR  (aa541-1195) | 30.7 | 2.8e-16 | 23  23  27  40  40  33 | 99  110  169  926  827  575 | 0.132  0.167  0.286  1.390  1.862  2.484 | I_A_  I_B_  I_C_  S_A_  S_B_  S_C_ |
| **H_sp_idb_17035_c0_g1_i1^3^** | Uncharacterized; shares several peptides with Tri_121458 and CLC_73; 10.5% A, 9.3% R, 11.0% P, 32.5% S; pI 13.0; IDP; Fig. S2H |  |  |  | 19  18  14  31  31  25 | 73  111  97  1196  1046  648 | 0.505  0.693  0.658  6.214  7.002  6.333 | I_A_  I_B_  I_C_  S_A_  S_B_  S_C_ |
| **H_sp_Tri_121458_c0_g1_i1^3^** | Uncharacterized; 14.6% A, 9.9% R, 11.8% G, 18.9% S; pI 12.3; IDP; shares several peptides with idb_17035; Fig. S2H |  |  |  | 3  5  3  8  8  4 | 23  28  20  352  256  183 | 0.524  0.740  0.541  4.925  6.762  5.987 | I_A_  I_B_  I_C_  S_A_  S_B_  S_C_ |
| **H_sp_CLC_866_c0_g1_i1^3^**  (aa169-300) | Uncharacterized/similar to Col17A1; domain: collagen_triple_helix (aa170-303); TM (aa16-38); 18.8% G; pI 5.5 | G1KDN1_  ANOCA  (aa582-718) | 56.5 | 6.6e-16 | 2  3  3  2  2  - | 12  18  19  49  30  - | 0.223  0.152  0.193  0.017  0.006  - | I_A_  I_B_  I_C_  S_A_  S_B_  S_C_ |
| **H_sp_idb_10705_c0_g1_i1^3^**  (aa358-714)  **H_sp_Tri_36495_c0_g1_i1** | Uncharacterized; domains: C-type_lectin (aa49-147,181-287); TM (aa21-43); pI 5.7; IDR (4.5%; aa308-339) | M4AQ88_XIPMA  (aa651-928)  V3ZN30_LOTGI  Lotgidraft_216450  (aa21-377) | 30.8  54.6 | 7.8e-19  1.2e-81 | 10  10  15  7  8  5 | 29  22  46  56  85  44 | 0.079  0.020  0.063  0.005  0.020  0.016 | I_A_  I_B_  I_C_  S_A_  S_B_  S_C_ |
| **H_sp_idb_12126_c0_g1_i1** | Uncharacterized; domain: TGF_b_C (aa194-324); SSP (aa1-23); pI 7.4; IDR (12.9%; aa182-221) |  |  |  | 2  -  -  3  2  - | 3  -  -  15  5  - | 0.007  -  -  0.002  0.001  - | I_A_  I_B_  I_C_  S_A_  S_B_  S_C_ |
| **H_sp_idb_12176_c0_g1_i1^3^** | Uncharacterized; 10.9% E, 34.3% T; pI 4.4; IDP |  |  |  | -  -  -  -  5  4 | -  -  -  -  14  22 | -  -  -  -  0.009  0.055 | I_A_  I_B_  I_C_  S_A_  S_B_  S_C_ |
| **H_sp_idb_13357_c0_g1_i1^3^**  (aa561-780)  **H_sp_idb_13358_c0_g1_i1** | Similar to shell matrix protein; domains: chitin-bd_II (aa473-526,535-592); 13.3% S, 26.4% T; pI 7.3; IDP; peptides from aa544-780! | PSM_MYTCA**^4^**  (aa71-290) | 30.2 | 2.8e-14 | 4  5  4  7  7  2 | 6  15  11  24  26  2 | 0.021  0.059  0.030  0.007  0.007  <0.001 | I_A_  I_B_  I_C_  S_A_  S_B_  S_C_ |
| **H_sp_idb_13824_c0_g1_i1^3^** | Uncharacterized; domains: Sushi_SCR_CCP (aa52-110,257-317,603-664,801-859), galectin_CRD (aa329-458,674-798,872-997); 10.0% T; pI 6.9; compare Tri_120377 |  |  |  | 5  10  20  5  5  4 | 11  30  63  14  12  11 | 0.021  0.030  0.069  0.001  0.001  0.002 | I_A_  I_B_  I_C_  S_A_  S_B_  S_C_ |
| **H_sp_idb_16318_c0_g1_i1^3^** | Uncharacterized; 11.2% P, 10.0% V, pI 9.5 |  |  |  | 7  3  8  10  7  9 | 19  5  22  212  72  114 | 0.166  0.042  0.081  0.448  0.094  0.283 | I_A_  I_B_  I_C_  S_A_  S_B_  S_C_ |
| **H_sp_idb_17014_c0_g1_i1^3^** | Uncharacterized; SSP (aa1-20); 11.7% A, 10.1% Q, 11.4% P, 17.8% T, pI 5.4; IDP |  |  |  | 2  5  7  11  10  11 | 3  9  23  94  68  79 | 0.010  0.018  0.028  0.045  0.035  0.072 | I_A_  I_B_  I_C_  S_A_  S_B_  S_C_ |
| **H_sp_idb_1745_c0_g1_i1^3^**  (aa35-1929)  Comp170175_c0_seq1_6  Comp189149_c0_seq1_5 | Similar to IgGFc-binding protein; domains: multiple (23) chitin-bd_II; SSP (aa1-19); pI 5.6; IDR (9.7%; aa661-693,762-773,836-846,930-940,961-1001,1064-1078,1142-1186,1334-1343,170-1711); compare CLC_2232! | K1PRD3_CRAGI  (aa280-2199) | 28.1 | 8.7e-55 | 27  48  61  87  74  54 | 64  161  269  613  485  235 | 0.025  0.058  0.128  0.065  0.060  0.038 | I_A_  I_B_  I_C_  S_A_  S_B_  S_C_ |
| **H_sp_idb_18575_c0_g1_i1^3^**  (aa9-461) | Similar to EGF-like domain-containing protein 2; domains: EGF_3 (aa58-93), ZP_2 (100-359); SPP (aa1-19), TM (aa410-432); 10.3% T; pI 5.2; IDR (14.7%; aa350-404,439-451) | ELDP2_LOTGI**^4^**  (aa10-485) | 33.1 | 2.6e-43 | 3  8  5  11  10  9 | 4  37  20  99  89  57 | 0.006  0.075  0.022  0.053  0.040  0.041 | I_A_  I_B_  I_C_  S_A_  S_B_  S_C_ |
| **H_sp_idb_18725_c0_g1_i1^3^**  (aa2-503) | Uncharacterized/similar to mucin; 10.2% A, 10.2% Q, 12.3% S, 15.0% T; pI 7.5; IDP; repeats (Fig. S2I) | MUC22_HUMAN  (aa1155-1655) | 28.4 | 2.4e-12 | -  -  2  2  4  2 | -  -  11  54  69  42 | -  -  0.028  0.033  0.074  0.124 | I_A_  I_B_  I_C_  S_A_  S_B_  S_C_ |
| **H_sp_idb_18771_c0_g1_i1^3^**  (aa1-173)  **H_sp_idb_18767_c0_g1_i1** | Similar to UP5; domain: Methyltransf_FA (aa71-165); SSP (aa1-22); pI 8.9; shares several peptides with idb_50884 | UP5_HALAI**^4^**  (aa1-173) | 89.0 | 4.2e-76 | 10  -  -  8  -  - | 39  -  -  165  -  - | 1.826  -  -  1.126  -  - | I_A_  I_B_  I_C_  S_A_  S_B_  S_C_ |
| **H_sp_idb_50884_c0_g1_i1^3^**  (aa1-173)  UP5_HALAI | Uncharacterized protein 5; domain: Methyltransf_FA (aa71-165); SSP (aa1-18); pI 8.8 | UP5_HALAI**^4^**  (aa1-173) | 90.8 | 4.5e-78 | 10  10  10  4  8  5 | 40  68  78  43  108  35 | 0.860  3.791  2.690  0.566  0.613  0.119 | I_A_  I_B_  I_C_  S_A_  S_B_  S_C_ |
| **H_sp_idb_19681_c0_g1_i1^3^**  (aa19-216) | Similar to ependymin-related protein 1; domain: ependymin (aa89-216); SSP (aa1-35); pI 8.2; 10.5% T | EPDR1_HALAI**^4^**  (aa1-198) | 66.7 | 7.7e-63 | -  -  -  4  3  - | -  -  -  13  8  - | -  -  -  0.011  0.014  - | I_A_  I_B_  I_C_  S_A_  S_B_  S_C_ |
| **H_sp_idb_19812_c0_g1_i1^3^**  (aa170-812)  **H_sp_idb_19814_c0_g1_i1**  Comp87710_c0_seq1_1  Comp71075_c0_seq4_5  Comp103815_c0_seq4_6 | Uncharacterized; domains: haem_peroxidase (aa164-744), TSP1 (aa747-789); SSP (aa1-23); 10.4% S; pI 9.1; IDR (8.8%; aa45-64,85-108,205-211,268-274,376-384,808-814); peptides from aa109-8590 | V4C5T3_LOTGI  Lotgidraft_115464  (aa2-658) | 44.5 | 1.1e-86 | -  7  7  -  -  - | -  13  18  -  -  - | -  0.008  0.009  -  -  - | I_A_  I_B_  I_C_  S_A_  S_B_  S_C_ |
| **H_sp_idb_19915_c0_g1_i1^3^**  **H_sp_CLC_8815_c0_g1_i1**  Comp103351_c1_seq9_2  Comp97720_c0_seq5_2 | Uncharacterized; domains: Pan_app (aa26-97,185-251,252-326); SSP (aa1-27); 12.6% S, 20.6% T; pI 5.8; IDR (37.7%; aa28-54,85-200) |  |  |  | 5  4  5  7  6  6 | 7  10  14  42  17  14 | 0.028  0.038  0.023  0.033  0.014  0.013 | I_A_  I_B_  I_C_  S_A_  S_B_  S_C_ |
| **H_sp_idb_20008_c0_g1_i1^3^**  (aa2-593)  Comp45250_c0_seq1_3  Comp401928_c0_seq1_5 | Uncharacterized; SSP (aa1-20); 16.3% Q, 12.5% G, 17.2% P; pI 10.4; IDP; repeats (Fig. S2J) | B4L3L7_DROMO  (aa1105-1801) | 30.5 | 2.7e-05 | 6  6  11  13  13  12 | 20  19  46  225  149  151 | 0.041  0.049  0.145  0.164  0.165  0.451 | I_A_  I_B_  I_C_  S_A_  S_B_  S_C_ |
| **H_sp_idb_20988_c0_g1_i1^3^**  (aa1-467) | Similar to shell protein 4/aplysianin-A; domain: amine_oxidase (aa2-447)¸ pI 9.3; see also Tri_25106 | A0A0G2YN89  _MYTCO**^4^**  (aa72-537) | 32.9 | 1.5e-50 | 17  25  24  8  10  7 | 61  127  144  46  55  24 | 0.224  0.323  0.418  0.010  0.010  0.008 | I_A_  I_B_  I_C_  S_A_  S_B_  S_C_ |
| **H_sp_idb_22001_c0_g1_i1^3^**  (aa32-229)  **H_sp_idb_22004_c0_g1_i1** | Similar to ependymin-related protein 1; domain: ependymin (aa102-228); 9.1% G, 10.4% T; pI 7.5; shares most peptides with other ependymin-related protein(s) | EPDR1_HALAI**^4^**  (aa1-199) | 75.9 | 4.9e-71 | 1  1  4  5  5  6 | 3  2  24  46  28  44 | 0.024  0.011  0.155  0.099  0.059  0.098 | I_A_  I_B_  I_C_  S_A_  S_B_  S_C_ |
| **H_sp_idb_22086_c0_g1_i1^3^**  (aa124-557) | Uncharacterized; TM? (aa20-42); shares several peptides with idb_22087; 31.6% D; pI 3.3; IDP; repeats (Fig. S2K) |  |  |  | 10  12  12  7  -  5 | 58  86  107  86  -  42 | 1.118  1.135  1.676  0.056  -  0.038 | I_A_  I_B_  I_C_  S_A_  S_B_  S_C_ |
| **H_sp_idb_22087_c0_g1_i1^3^** | Uncharacterized; TM? (aa20-42); shares most peptides with idb_22086; 20.4% D, 19.1% G, 10.7% M; pI 3.3; IDP; repeats (Fig. S2K) |  |  |  | 1  1  2  1  4  1 | 2  7  12  1  38  1 | 0.003  0.012  0.013  <0.001  0.025  <0.001 | I_A_  I_B_  I_C_  S_A_  S_B_  S_C_ |
| **H_sp_idb_42421_c0_g1_i1^3^**  (aa2-230) | Uncharacterized; shares 2 peptides with idb_22086; 28.1% D; pI 3.3; IDP; repeats (Fig. S2K) |  |  |  | 4  5  5  1  3  - | 26  22  60  7  13  - | 0.296  0.193  0.444  0.001  0.008  - | I_A_  I_B_  I_C_  S_A_  S_B_  S_C_ |
| **H_sp_idb_23329_c0_g1_i1^3^**  (aa140-649) | Uncharacterized; SSP (aa1-16); 24.0%A, 10.0% P, 20.2%T, pI 3.9; IDP; unique peptides in aa18-100 | H3E3Y1_PRIPA  (aa100-614) | 43.3 | 5.8e-20 | -  -  -  2  2  3 | -  -  -  4  5  10 | -  -  -  0.002  0.005  0.001 | I_A_  I_B_  I_C_  S_A_  S_B_  S_C_ |
| **H_sp_idb_23519_c0_g1_i1^3^**  (aa1-1668)  H_sp_Tri_4114_c0_g1_i1  Comp94729_c0_seq3_2  Comp110183_c0_seq1_4 | Uncharacterized; 12.5% G, 14.1% S, 40.6% T; pI 4.4; IDP; peptides in aa951-1339 | A0A090CG02  _PODAN  (aa1529-3167) | 40.7 | 7.3e-76 | -  3  6  -  -  - | -  5  10  -  -  - | -  0.004  0.003  -  -  - | I_A_  I_B_  I_C_  S_A_  S_B_  S_C_ |
| **H_sp_Tri_31898_c0_g1_i1^3^**  (aa1-198)  **H_sp_idb_24481_c0_g1_i1** | Similar to to ependymin-related protein 1; domain: ependymin (aa71-198); SSP (aa1-17); 9.6% T, 11.1% V; pI 7.5; shares several peptides with Tri_31892 and Tri_31897 | EPDR1_HALAI**^4^**  (aa1-198) | 75.3 | 8.5e-72 | -  -  2  8  6  7 | -  -  3  41  28  19 | -  -  0.002  0.049  0.062  0.054 | I_A_  I_B_  I_C_  S_A_  S_B_  S_C_ |
| **H_sp_idb_25730_c0_g1_i1**  Comp68740_c0_seq1_1 | Uncharacterized; domains: VWA (aa4-95), TSP1 (aa93-157,156-219,218-281,280-343,342-405), chitin-bd_II (aa416-488,480-532), ConA_like (aa624-769); 12.3% G, 9.8% T; pI 6.7; repeats (Fig. S2M) |  |  |  | 14  19  18  16  15  13 | 56  123  85  246  273  92 | 0.211  0.413  0.130  0.054  0.099  0.024 | I_A_  I_B_  I_C_  S_A_  S_B_  S_C_ |
| **H_sp_idb_25746_c0_g1_i1^3^**  (aa88-662)  Comp44198_c0_seq1_4  Comp39177_c0_seq1_3 | Uncharacterized/peroxidasin-like; domain: peroxidase_3 (aa79-664); SSP (aa1-22); pI 9.0; IDR (3.1%; aa1-11,25-34); peptides from aa35-659! | V4C5T3_LOTGI  Lotgidraft_115464  (aa2-563) | 47.2 | 1.7e-110 | 31  42  48  28  31  21 | 177  330  437  595  448  314 | 0.841  1.595  2.072  0.107  0.188  0.141 | I_A_  I_B_  I_C_  S_A_  S_B_  S_C_ |
| **H_sp_idb_26030_c0_g1_i1^3^**  (aa21-693)  Comp104856_c0_seq3_5 | Similar to zinc transporter; domain: zinc/iron_permease (ZIP; aa395-687); SSP (aa1-23); multiple TM (6; in C-term half); pI 5.3; IDR (18.0%; aa20-38,171-201,292-332,504-540) | E6ZJ32_DICLA  (aa21-680) | 31.4 | 5.5e-48 | 5  5  5  6  5  4 | 32  44  34  153  125  43 | 1.103  0.603  0.324  0.349  0.245  0.053 | I_A_  I_B_  I_C_  S_A_  S_B_  S_C_ |
| **H_sp_idb_26568_c0_g1_i1^3^**  (aa226-1023)  **H_sp_idb_26567_c0_g1_i1**  Comp3500_c0_seq1_5 | Uncharacterized; SSP (aa1-20); 12.8% N, 14.2% Q, 10.3% P, 13.4% S; pI 9.7; IDP; repeats (Fig. S2N) | V4CGH1_LOTGI  Lotgidraft_172708  (aa9-816) | 26.1 | 7.3e-11 | -  2  7  13  17  19 | -  2  17  88  133  188 | -  0.002  0.007  0.008  0.027  0.073 | I_A_  I_B_  I_C_  S_A_  S_B_  S_C_ |
| **H_sp_idb_27355_c0_g1_i1^3^**  (aa13-560)  Comp30184_c0_seq1_4 | Uncharacterized; SSP (aa1-41); 9.8% A, 10.7% Q, 10.9% S, 10.2% T; pI 6.9; IDP; repeats (Fig. S2P) | Q2UQ79_ASPOR  (aa455-1013) | 26.2 | 2.0e-5 | 5  6  9  12  13  15 | 9  10  39  80  113  126 | 0.008  0.013  0.067  0.047  0.112  0.385 | I_A_  I_B_  I_C_  S_A_  S_B_  S_C_ |
| **H_sp_idb_27866_c0_g1_i1^3^**  (aa23-358) | Uncharacterized; 13.4% A, 9.9% G, 16.5% S, 19.6% T; pI 4.5; IDP; repeats (Fig. S2R) | A0A0G2KUY1  _DANRE  (aa317-659) | 38.3 | 3.1e-9 | -  -  -  2  3  3 | -  -  -  11  17  13 | -  -  -  0.018  0.098  0.114 | I_A_  I_B_  I_C_  S_A_  S_B_  S_C_ |
| **H_sp_idb_2814_c0_g1_i1^3^**  (aa233-624) | Uncharacterized; domains: [Lectin_gal-bd](http://www.ebi.ac.uk/interpro/entry/IPR000922) (aa237-310), CUB (aa320-444); SSP (aa1-19), TM (aa528-550); pI 5.1; IDR (62.7%; aa1-238,458-516,550-674) | V4A930_LOTGI**^4^**  Lotgidraft_232714  (aa377-794) | 27.4 | 6.9e-14 | -  -  4  3  6  7 | -  -  5  7  16  27 | -  -  0.002  0.001  0.007  0.006 | I_A_  I_B_  I_C_  S_A_  S_B_  S_C_ |
| **H_sp_idb_28907_c0_g1_i1^3^** | Uncharacterized; SSP (aa1-19); 17.2% Q, 9.8% G, 11.1% P; pI 9.8; IDP; shares 1 peptide with idb_36084 |  |  |  | 2  4  4  5  3  3 | 8  12  25  71  54  29 | 0.077  0.064  0.179  0.103  0.106  0.057 | I_A_  I_B_  I_C_  S_A_  S_B_  S_C_ |
| **H_sp_idb_36084_c0_g1_i1^3^** | Uncharacterized; SSP (aa1-19); 17.2% Q, 10.3% G; pI 9.7; IDR (63.6%; aa1-129) |  |  |  | -  1  1  -  1  - | -  1  1  -  1  - | -  0.023  0.011  -  <0.001  - | I_A_  I_B_  I_C_  S_A_  S_B_  S_C_ |
| **H_sp_idb_3074_c0_g1_i1^3^** | Uncharacterized; 14.4% S, 14.0% T; pI 7.2; domain: EGF_3 (aa53-90); SSP (aa1-21); IDP; peptides in aa1067-1199 |  |  |  | 2  4  6  12  11  10 | 6  9  15  151  115  101 | 0.007  0.004  0.004  0.018  0.017  0.021 | I_A_  I_B_  I_C_  S_A_  S_B_  S_C_ |
| **H_sp_idb_32310_c0_g1_i1^3^**  (aa24-613)  Comp56242_c1_seq1_1 | Similar to chitinase-3; domains: chitinase_II (aa21-371), chitin-bd_II (aa730-786,884-941); SSP (aa1-17); pI 8.7; IDR (31.4%; aa406-474,520-627,633-657,800-891); peptides in aa49-275 | J7F1C1_HYRCU  (aa28-611) | 43.6 | 3.6e-54 | 3  3  7  4  5  - | 4  9  16  10  7  - | 0.002  0.005  0.006  0.001  0.001  - | I_A_  I_B_  I_C_  S_A_  S_B_  S_C_ |
| **H_sp_idb_32603_c0_g1_i1^3^**  **H_sp_idb_32602_c0_g1_i1**  Comp118790_c0_seq1_1  Comp184680_c0_seq1_2  Comp205609_c0_seq1_4 | Uncharacterized; SSP (aa1-21); 14.0% Q, 13.7% P, 10.7% S; pI 6.4; IDP; repeats (Fig. S2S) |  |  |  | 2  9  12  11  10  12 | 3  33  56  84  105  132 | 0.001  0.040  0.058  0.017  0.075  0.136 | I_A_  I_B_  I_C_  S_A_  S_B_  S_C_ |
| **H_sp_idb_34528_c0_g1_i1^3^**  (aa213-386) | Uncharacterized protein 2; SSP (aa1-25)/TM (aa12-34); 10.6% A, 11.7% L, 18.9% P; pI 10.0; IDP; repeats: [APLAXXAAPVN]_2_ in aa 94-119, 2 [LPPGAAXX]_2_ in aa186-201 | UP2_HALAI**^4^**  (aa5-179) | 77.7 | 1.3e-23 | 5  7  8  11  11  11 | 16  21  60  221  167  136 | 0.090  0.214  0.400  0.735  0.819  1.179 | I_A_  I_B_  I_C_  S_A_  S_B_  S_C_ |
| **H_sp_idb_34907_c0_g1_i1^3^**  (aa49-546)  Comp96713_c3_seq4_2  Comp96713_c3_seq1_1  B6RB63_HALDI | Protein disulfide-isomerase; domains: Thioredoxin-like_fold (aa55-180,182-279,281-531,409-531); 11.4% E, 9.2% K; pI 4.5; IDR (15.8%; aa1-34,495-506) | B6RB63_HALDI  (aa1-499) | 92.6 | 2.1e-167 | 3  5  9  -  -  - | 4  8  23  -  -  - | 0.003  0.079  0.092  -  -  - | I_A_  I_B_  I_C_  S_A_  S_B_  S_C_ |
| **H_sp_idb_35012_c0_g1_i1**  (aa76-111) | Uncharacterized; peptides from aa64-105; domain: lustrin_cystein (aa65-111); 12.5% L; pI 8.5; IDR (26.8%; aa1-30) | K1QWL9_CRAGI  (aa86-123) | 68.4 | 1.4e-6 | -  -  -  3  3  2 | -  -  -  9  6  5 | -  -  -  0.002  0.002  0.002 | I_A_  I_B_  I_C_  S_A_  S_B_  S_C_ |
| **H_sp_idb_35506_c0_g1_i1^3^**  (aa1-215)  Comp97330_c0_seq1_5  Q19BK2_HALDI | Glutathione-S-transferase (mu); domains: Thioredoxin-like_fold (aa1-84), GST_C (aa99-190); 10.7% L; pI 6.2 | B3TK24_HALDV  (aa1-215) | 82.3 | 2.0e-79 | 5  3  5  7  2  3 | 6  7  14  30  6  8 | 0.027  0.012  0.031  0.010  0.001  0.002 | I_A_  I_B_  I_C_  S_A_  S_B_  S_C_ |
| **H_sp_idb_3591_c0_g1_i1^3^** | Uncharacterized; SSP (aa1-16); 14.8% G, 11.9% S; pI 6.6; IDP |  |  |  | 29  38  43  50  46  47 | 104  179  205  1210  818  845 | 0.231  0.316  0.351  0.590  0.616  0.899 | I_A_  I_B_  I_C_  S_A_  S_B_  S_C_ |
| **H_sp_idb_36583_c0_g1_i1^3^** | Uncharacterized; 14.7% G, 32.5% S, 16.4% T, pI 6.3; IDP; repeats (Fig. S2T) |  |  |  | -  2  4  4  4  5 | -  5  15  36  46  66 | -  0.013  0.045  0.014  0.043  0.174 | I_A_  I_B_  I_C_  S_A_  S_B_  S_C_ |
| **H_sp_idb_39663_c0_g1_i1^3^** | Uncharacterized; 22.4% S, 17.4% T; pI 3.8; IDR (74.3%; aa103-397) |  |  |  | 4  3  5  5  5  4 | 9  13  20  30  36  32 | 0.042  0.094  0.321  0.042  0.073  0.108 | I_A_  I_B_  I_C_  S_A_  S_B_  S_C_ |
| **H_sp_idb_40080_c0_g1_i1^3^**  (aa1-159) | Similar to ependymin-1/2; domain: ependymin (aa60-156); shares 6 peptides with CLC_1876; 12.5% T, 10.0% V; pI 7.8; IDR (3.8%; aa155-160) | EPDR2_HALAI**^4^**  (aa14-171)  EPDR1_HALAI**^4^**  (aa11-170) | 65.4  66.2 | 1.8e-49  1.1e-47 | -  -  -  -  2  - | -  -  -  -  4  - | -  -  -  -  0.003  - | I_A_  I_B_  I_C_  S_A_  S_B_  S_C_ |
| **H_sp_idb_4071_c0_g1_i1^3^**  (aa43-608)  (aa618-713) | Similar to basic proline-rich protein; Q/P-rich (15.4% Q, 9.8% G, 34.6% P), pI 5.1 and  Methionine-rich protein (9.5% C, 2.1% M); pI 6.1;  IDP; repeats (Fig. S2U) | PRP_PIG  (aa91-647)  MRP_LOTGI**^4^**  (aa162-270) | 40.2  40.9 | 3.7e-24  5.7e-7 | 11  10  12  22  18  18 | 21  22  47  364  244  184 | 0.085  0.173  0.178  0.603  0.439  0.498 | I_A_  I_B_  I_C_  S_A_  S_B_  S_C_ |
| **H_sp_idb_42414_c0_g1_i1^3^**  (aa66-387)  Comp188351_c0_seq1_6 | Uncharacterized; domain: ConA_like (aa221-382); SSP (aa1-24); 12.0% T; pI 4.9; IDR (28.6%; aa1-111) | V4B726_LOTGI  Lotgidraft_236297  (aa45-379) | 25.4 | 2.3e-8 | -  3  2  6  7  4 | -  5  4  25  26  16 | -  0.017  0.010  0.009  0.013  0.010 | I_A_  I_B_  I_C_  S_A_  S_B_  S_C_ |
| **H_sp_idb_43368_c0_g1_i1^3^**  (aa1-462) | Uncharacterized; 28.7% A, 11.9% Q, 12.3% G, 11.2% S; pI 3.3; only one measurable peptide predicted, the second peptide is from a miss-cleavage (K-P)! IDP; repeats (Fig. S2V) | A0A0C6FK35 _9RHIZ  (aa52-546) | 29.7 | 4.4e-13 | 1  1  1  2  2  - | 3  5  5  4  2  - | 0.400  0.438  1.526  0.013  0.022  - | I_A_  I_B_  I_C_  S_A_  S_B_  S_C_ |
| **H_sp_idb_44191_c0_g1_i1^3^**  (aa38-712)  Comp96813_c0_seq1_5  Comp105290_c0_seq2_6 | Similar to cathepsin F; domains: cystatin (aa23-111,114-225), peptidase_I29 (aa411-468), peptidase_C1A (aa499-710); SSP (aa1-17); pI 6.0; IDR (23.9%; aa115-129,217-290,356-403,477-508) | K1QYP7_CRAGI  (aa17-715) | 46.6 | 3.4e-96 | 2  9  6  6  10  5 | 5  27  17  68  91  45 | 0.012  0.030  0.018  0.013  0.022  0.025 | I_A_  I_B_  I_C_  S_A_  S_B_  S_C_ |
| **H_sp_idb_44571_c0_g1_i1^3^**  (66-836)  Comp252421_c0_seq1_4 | Uncharacterized; domains: chitin-bd_II (aa550-612,608-676,721-779,780-838); TM (aa347-369); 11.8% S; pI 8.1; IDR (41.0%; aa1-38,49-87,114-332,670-718) | V4A7L2_LOTGI  Lotgidraft_232881  (aa14-860) | 32.8 | 2.6e-15 | -  -  -  6  6  5 | -  -  -  13  9  14 | -  -  -  0.001  0.001  0.002 | I_A_  I_B_  I_C_  S_A_  S_B_  S_C_ |
| **H_sp_idb_44689_c0_g1_i1^3^**  (aa2-899)  **H_sp_idb_44690_c0_g1_i1** | Uncharacterized; 10.5% G, 28.3% S, 14.0% T; pI 5.1; IDP; repeats (Fig. S2W) | A0A0N0DN50  _STRSU  (aa2355-3252) | 32.2 | 4.8e-21 | 3  3  4  5  5  7 | 5  11  26  33  57  81 | 0.008  0.026  0.087  0.009  0.032  0.118 | I_A_  I_B_  I_C_  S_A_  S_B_  S_C_ |
| **H_sp_idb_45304_c0_g1_i1^3^**  (aa27-313)  **H_sp_idb_45305_c0_g1_i1** | Similar to lustrin A; domains: [WAP/ Cys_repeat_1](http://www.ebi.ac.uk/interpro/entry/IPR008197) (aa67-110,158-204,253-298,353-406); SSP (aa1-19); 13.3% C, 9.9% P, 9.9% T; pI 7.4; IDR (1.5%; aa355-360) | J7QAX0_PATVU  (aa143-438) | 30.0 | 1.2e-13 | -  3  1  7  7  5 | -  4  1  25  35  25 | -  0.005  0.001  0.004  0.027  0.013 | I_A_  I_B_  I_C_  S_A_  S_B_  S_C_ |
| **H_sp_Tri_38314_c0_g1_i1^3^**  (aa5-164)  Comp24992_c0_seq1_5  Comp24992_c0_seq1_1 | Similar to lustrin A (fragment); domains: WAP (aa57-103,104-158); SSP (aa1-19); 14.0% C, 9.1% G, 14.6% P; pI 8.7 | J7QAX0_PATVU  (aa23-192) | 31.5 | 1.1e-9 | -  2  4  3  2  2 | -  4  6  17  6  2 | -  0.004  0.033  0.011  0.009  0.004 | I_A_  I_B_  I_C_  S_A_  S_B_  S_C_ |
| **H_sp_idb_47306_c0_g1_i1^3^** | Uncharacterized; SSP (aa1-19); 24.1% A, 18.1% Q, 11.4% G; pI 4.8; IDP; repeats (Fig. S2X) |  |  |  | 2  3  3  2  2  2 | 20  23  28  119  125  101 | 1.533  2.366  3.245  0.706  0.862  1.219 | I_A_  I_B_  I_C_  S_A_  S_B_  S_C_ |
| **H_sp_idb_51603_c0_g1_i1^3^** | Uncharacterized; SSP (aa1-21); 10.1% R, 10.1% S, pI 9.4; IDR (36.1%; aa28-36,86-119) |  |  |  | -  -  2  5  -  3 | -  -  7  43  -  10 | -  -  0.020  0.087  -  0.060 | I_A_  I_B_  I_C_  S_A_  S_B_  S_C_ |
| **H_sp_idb_5218_c0_g1_i1^3^** | Uncharacterized; pI 6.9; |  |  |  | 8  9  10  14  14  11 | 27  48  46  245  206  117 | 0.334  0.458  0.319  0.980  1.383  0.966 | I_A_  I_B_  I_C_  S_A_  S_B_  S_C_ |
| **H_sp_idb_52687_c0_g1_i1^3^**  (aa35-227) | Similar to ependymin-related protein 2; domain: ependymin (aa101-227); 10.0% T; pI 8.7 | EPDR2_HALAI**^4^**  (aa11-199) | 57.6 | 9.0e-51 | -  3  -  4  4  2 | -  7  -  12  20  5 | -  0.019  -  0.006  0.020  0.001 | I_A_  I_B_  I_C_  S_A_  S_B_  S_C_ |
| **H_sp_idb_53451_c0_g1_i1**  (aa1-234) | Similar to endochitinase; domains: Glyco_hydro_18, catalytic (aa2-94), chitin-bd_II (aa167-235); 26.9% T; pI 4.1; IDR (40.3%; aa88-178); domains separated by T-rich sequence | A0A067RV51  _ZOONE  (aa313-555) | 37.7 | 8.8e-10 | -  3  -  -  2  - | -  5  -  -  4  - | -  0.036  -  -  0.003  - | I_A_  I_B_  I_C_  S_A_  S_B_  S_C_ |
| **H_sp_idb_54301_c0_g1_i1^3^** | Uncharacterized; 10.3% R, 12.1% G, pI 9.7 |  |  |  | 2  6  3  2  7  6 | 3  20  8  13  92  37 | 0.009  0.140  0.017  0.037  0.653  0.185 | I_A_  I_B_  I_C_  S_A_  S_B_  S_C_ |
| **H_sp_idb_55709_c0_g1_i1^3^** | Uncharacterized; shares several peptides with Tri_138845 and idb_55710; 25.3% D, 12.3% E; pI 3.9; IDP |  |  |  | 4  5  11  3  3  4 | 6  13  46  14  28  26 | 0.011  0.015  0.057  0.001  0.003  0.003 | I_A_  I_B_  I_C_  S_A_  S_B_  S_C_ |
| **H_sp_Tri_138845_c0_g1_i1^3^**  (aa6-388)  **H_sp_CLC_25186_c0_g1_i1** | Similar to shell matrix protein; SSP (aa1-22)/TM (aa7-26); 23.9% D; pI 3.8; if SSP: 25.1% D;  IDP; shares several peptides with idb_55709 | G9MBW9  _PINMA**^4^**  (aa3-305) | 31.1 | 2.0e-14 | 1  2  3  1  1  1 | 3  8  13  8  3  11 | 0.006  0.027  0.142  0.005  0.001  0.011 | I_A_  I_B_  I_C_  S_A_  S_B_  S_C_ |
| **H_sp_idb_5580_c0_g1_i1**  Comp8431_c0_seq1_4 | Uncharacterized; 11.1% Q, pI 9.1; IDP |  |  |  | -  4  8  18  16  15 | -  12  23  108  92  115 | -  0.008  0.013  0.011  0.012  0.028 | I_A_  I_B_  I_C_  S_A_  S_B_  S_C_ |
| **H_sp_idb_5896_c0_g1_i1^3^**  (aa135-1236) | Uncharacterized; domain: stereocilin_rel (aa426-1214); SSP (aa1-20); 10.4% D; pI 4.9; IDR (18.7%; aa21-195,231-256,904-932) | V3ZXW7_LOTGI**^4^**  Lotgidraft_235120  (aa42-1130) | 36.2 | 3.3e-116 | 10  15  18  16  12  3 | 21  30  49  71  61  8 | 0.012  0.019  0.022  0.007  0.005  0.001 | I_A_  I_B_  I_C_  S_A_  S_B_  S_C_ |
| **H_sp_idb_6290_c0_g1_i1^3^**  (aa1-436)  (aa437-974)  (aa975-1602) | Similar to chitin deacetylase; domain: Glyco_hydro/deAcase_b/a-brl/NodB (aa53-323); SSP (aa1-19); pI 7.6;  IDR (16.7%)  Similar to translation initiation factor 2 (IF-2 GTPase); 12.2% P, 10.7% S; pI 9.7; IDP  Uncharacterized; domain: Glyco_hydro/deAcase_b/a-brl (aa1323-1591); 11.6% P; pI 5.2; IDR (39.7%)  IDR_tot_: 53.2%; aa364-802,809-847,853-1224) | J7FHX7_HYRCU  (aa2-443)  RANI13_STRSU  (aa313-789  K1P514_CRAGI  (aa168-801) | 41.4  23.0  27.6 | 1.3e-45  7.6e-7  1.4e-28 | 5/4/6  6/3/9  9/14/11  3/10/8  -/10/4  -/10/6 | 20  32  95  112  68  70 | 0.007  0.011  0.030  0.005  0.003  0.006 | I_A_  I_B_  I_C_  S_A_  S_B_  S_C_ |
| **H_sp_idb_64214_c0_g1_i1**  Comp52612_c0_seq1_4 | Uncharacterized; SSP (aa1-22); 13.4% R; pI 9.0; similar to comp91920_c0_seq3_1 |  |  |  | -  -  -  3  2  4 | -  -  -  8  6  13 | -  -  -  0.004  0.001  0.020 | I_A_  I_B_  I_C_  S_A_  S_B_  S_C_ |
| **H_sp_idb_66139_c0_g1_i1^3^**  (aa6-135) | Uncharacterized; pI 6.6; two HQV[G,K]L repeats in aa56-65 | K1R0J9_CRAGI  (aa460-584) | 40.2 | 4.4e-15 | 4  4  4  4  3  - | 19  15  14  24  11  - | 1.062  0.824  0.336  0.143  0.031  - | I_A_  I_B_  I_C_  S_A_  S_B_  S_C_ |
| **H_sp_idb_67370_c0_g1_i1^3^**  (aa1-106) | Uncharacterized; SSP (aa1-18); 14.9% G, pI 5.3 | R7T5B9_CAPTE  (aa1-104) | 45.8 | 6.5e-16 | 2  2  2  2  2  2 | 11  21  23  40  32  23 | 0.065  0.073  0.143  0.015  0.021  0.026 | I_A_  I_B_  I_C_  S_A_  S_B_  S_C_ |
| **H_sp_Tri_121805_c0_g1_i1**  (aa32-163)  **H_sp_idb_7323_c0_g1_i1** | Uncharacterized; domains: WAP (aa67-112,113-163); SSP (aa1-27); 16.4% C, 14.5% P; pI 8.4; IDR (6.7%; aa1-11) | G3UPF3_MELGA  (aa44-165) | 38.2 | 9.3e-10 | 2  -  3  3  2  - | 7  -  6  14  6  - | 0.011  -  0.027  0.004  0.004  - | I_A_  I_B_  I_C_  S_A_  S_B_  S_C_ |
| **H_sp_idb_7598_c0_g1_i1^3^**  (aa1-1942) | Uncharacterized/similar to proteophosphoglycan; domains: PT_rep (aa119-140,181-211); 12.2% A, 14.7% S, 22.4% T; pI 4.5; IDP | E9AEM9_LEIMA  (aa13878-15805) | 19.8 | 3.6e-37 | -  -  4  5  5  9 | -  -  10  16  17  19 | -  -  0.007  0.003  0.008  0.019 | I_A_  I_B_  I_C_  S_A_  S_B_  S_C_ |
| **H_sp_idb_8012_c0_g1_i1^3^** | Uncharacterized; 15.4% Q, 12.7% G, 9.3% P, 9.8% S; pI 10.3; IDP |  |  |  | 9  10  16  37  33  31 | 24  28  47  370  283  238 | 0.010  0.011  0.021  0.032  0.032  0.056 | I_A_  I_B_  I_C_  S_A_  S_B_  S_C_ |
| **H_sp_Tri_130845_c0_g1_i1^3^**  (aa1-573)  **H_sp_idb_813_c0_g1_i1** | Similar to carbonic anhydrase; domain: αCA_2 (aa30-313); SSP (aa1-16); 9.9% Q, 12.5% G; pI 6.0; IDR (26.7%; aa371-459,522-584) | J7QJT8_PATVU  (aa1-613) | 31.2 | 2.3e-35 | 2  5  5  8  2  - | 2  14  12  25  6  - | 0.019  0.022  0.014  0.004  0.001  - | I_A_  I_B_  I_C_  S_A_  S_B_  S_C_ |
| **H_sp_idb_946_c0_g1_i1**  (aa11-3829)  Comp106850_c0_seq1_5  Comp106850_c0_seq3_4 | Similar to neurotrypsin; domains: SRCR_like (aa154-265, 1022-1130,1855-1958), CUB (aa211-2208), C-type_lectin (aa2492-2602), multiple pectin_lyase_fold; SSP(aa1-27)/TM (aa7-26), TM (aa2614-2636); pI 6.0; IDR (12.8%; aa2401-2405,2706-2779,2792-2110) | K1QUE4_CRAGI  (aa9-2955) | 47.6 | 0e0 | -  4  2  -  -  - | -  5  3  -  -  - | -  <0.001  <0.001  -  -  - | I_A_  I_B_  I_C_  S_A_  S_B_  S_C_ |
| **H_sp_idb_982_c0_g1_i1^3^** | Uncharacterized; pI 5.3; domains multiple Sushi_SCR_CCP, galactose_bd, chitin-bd, fucolectin/tachylectin-4/pentraxin-1, galectin; SSP (aa1-22); IDR (4.4%; aa385-484,707-742) |  |  |  | -  6  46  10  9  9 | -  9  167  39  30  45 | -  0.002  0.044  0.001  0.001  0.002 | I_A_  I_B_  I_C_  S_A_  S_B_  S_C_ |
| **H_sp_Tri_100716_c0_g1_i1**  (aa1-110)  UP7_HALAI | Similar to uncharacterized protein 7/ similar to ML5H8**^4^**; SSP (aa1-19); 10.7% C; pI 9.7 | UP7_HALAI**^4^**  (aa1-110) | 90.0 | 2.1e-47 | -  3  4  5  2  - | -  10  14  8  6  - | -  0.189  0.330  0.046  0.011  - | I_A_  I_B_  I_C_  S_A_  S_B_  S_C_ |
| **H_sp_Tri_105613_c0_g1_i1^3^**  (aa1-1442**)**  Comp32926_c1_seq1_1  Comp508308_c0_seq1_2  Comp32926_c0_seq1_5  Comp59338_c0_seq1_5 | Similar to thioester-containing protein; domains: A2M_N (aa115-199), A2M_N_2 (aa449-582), Macroglobulin_a2 (721-811), Terpenoid_cyclase/PrenylTrfase (aa939-1241), A-macroglobulin_rcpt-bd (aa1309-1424); SSP (aa1-16); pI 8.3; IDR (1.3%; aa693-711) | D5FT50_9BIVA  (aa1-1450) | 57.6 | 0e0 | 2  10  12  6  9  - | 3  20  21  11  22  - | 0.002  0.006  0.006  0.001  0.002  - | I_A_  I_B_  I_C_  S_A_  S_B_  S_C_ |
| **H_sp_Tri_107502_c0_g1_i1^3^**  (aa44-805) | Uncharacterized; domains: NodB_homology (aa27-321,513-794); SSP (aa1-21); 10.1% T; pI 8.2; IDR (21.2%; aa330-495) | V4AMK1_LOTGI**^4^**  Lotgidraft_181237  (aa1-811) | 60.2 | 2.0e-57 | -  3  3  -  -  - | -  4  7  -  -  - | -  0.001  0.001  -  -  - | I_A_  I_B_  I_C_  S_A_  S_B_  S_C_ |
| **H_sp_Tri_108584_c0_g1_i1^3^** | Uncharacterized; SSP (aa1-23); 15.1% P, 12.8% S; pI 10.6; IDP; repeats (Fig. S2Z) |  |  |  | 19  24  25  41  37  29 | 141  155  197  1842  1101  698 | 3.320  2.348  1.996  8.640  7.598  8.236 | I_A_  I_B_  I_C_  S_A_  S_B_  S_C_ |
| **H_sp_Tri_109450_c0_g1_i1^3^**  (aa5-229)  Comp101252_c0_seq3_2 | Uncharacterized; domains: chitin-bd_II (aa19-85,83-152); SSP (aa1-17); 9.1% C; pI 8.0 | K1R0D4_CRAGI  (aa2-228) | 45.4 | 4.6e-41 | -  4  3  9  12  7 | -  12  6  35  57  28 | -  0.025  0.009  0.024  0.067  0.034 | I_A_  I_B_  I_C_  S_A_  S_B_  S_C_ |
| **H_sp_Tri_117880_c0_g1_i1^3^**  H_sp_idb_23862_c0_g1_i1 | Uncharacterized; 19.5% A, 12.1% S, 20.1% T; pI 4.3; domain: PT_repeat (aa651-681); IDP; shares 2 peptides with idb_23329 |  |  |  | -  5  6  6  7  9 | -  9  27  19  25  54 | -  0.027  0.055  0.011  0.026  0.140 | I_A_  I_B_  I_C_  S_A_  S_B_  S_C_ |
| **H_sp_Tri_119193_c0_g1_i1^3^**  (aa44-175)  UP4_HALAI | Uncharacterized protein 4; TM (aa43-62); 12.0% A, 11.4% L; pI 8.7 | UP4_HALAI**^4^**  (aa1-130) | 84.1 | 4.5e-42 | 8  8  10  11  11  11 | 82  98  107  487  301  249 | 9.738  4.278  3.579  5.836  5.433  5.576 | I_A_  I_B_  I_C_  S_A_  S_B_  S_C_ |
| **H_sp_Tri_12080_c0_g1_i1^3^**  (aa57-1528) | Uncharacterized; SSP (aa1-20); 12.8% T, pI 10.0; IDP; peptides start at aa29! | B4L2U0_DROMO  (aa1384-2829) | 21.6 | 5.5e-10 | 9  12  19  50  37  36 | 14  29  59  674  354  336 | 0.010  0.007  0.018  0.084  0.043  0.072 | I_A_  I_B_  I_C_  S_A_  S_B_  S_C_ |
| **H_sp_Tri_12500_c0_g1_i1**  (aa81-539) | Uncharacterized; domain: Ig-like_fold (aa414-474); SSP (aa1-30), pI 8.7; IDR (9.7%; aa124-139,549-586) | V3ZJB4_LOTGI  Lotgidraft_153783  (aa59-487) | 33.5 | 1.0e-10 | -  -  4  5  4  - | -  -  5  11  8  - | -  -  0.005  0.002  0.002  - | I_A_  I_B_  I_C_  S_A_  S_B_  S_C_ |
| **H_sp_Tri_127820_c0_g1_i1^3^** | Uncharacterized; SSP (aa1-31); 19.0% G, 9.8% L, 13.1% P, pI 9.5; IDR (41.8%; aa71-85,134-184) |  |  |  | 4  5  4  6  5  5 | 26  21  23  103  83  72 | 1.997  0.819  0.867  0.451  0.124  0.156 | I_A_  I_B_  I_C_  S_A_  S_B_  S_C_ |
| **H_sp_Tri_129603_c0_g1_i1^3^**  (aa1-945)  Comp105495_c0_seq9_4 | Uncharacterized; domain: Hedgehog_sig/DD-Pept_Zn-bd (aa251-429); SSP (aa1-18); pI 5.3; IDR (7.0%; aa225-253,651-688) | V4B8A6_LOTGI**^4^**  Lotgidraft_235988  (aa1-955) | 50.3 | 1.2e-97 | 8  18  17  10  9  - | 16  43  39  38  30  - | 0.024  0.073  0.029  0.002  0.002  - | I_A_  I_B_  I_C_  S_A_  S_B_  S_C_ |
| **H_sp_Tri_135479_c0_g1_i1**  (aa153-866) | Uncharacterized; domain: MetalloPept_cat (aa188-409); SSP (aa1-20); 12.9% S; pI 9.2; IDR (20.9%; aa164-179,559-651,686-716,792-848) | V3Z0L4_LOTGI  Lotgidraft_210968  (aa51-754) | 28.5 | 8.0e-46 | -  -  2  4  -  - | -  -  3  7  -  - | -  -  0.001  0.001  -  - | I_A_  I_B_  I_C_  S_A_  S_B_  S_C_ |
| **H_sp_Tri_14507_c0_g1_i1^3^**  (aa96-590) | Uncharacterized; SSP (aa1-21); 10.7% P, 10.0% S, pI 8.7; IDR (34.8%; aa187-249,254-391); peptides start at aa44! | V4AT07_LOTGI**^4^**  Lotgidraft_173199  (aa1-610) | 28.5 | 3.7e-20 | -  9  11  17  14  11 | -  23  24  92  101  67 | -  0.023  0.020  0.018  0.045  0.020 | I_A_  I_B_  I_C_  S_A_  S_B_  S_C_ |
| **H_sp_Tri_1743_c0_g1_i1^3^**  (aa1-249)  Comp89145_c0_seq2_3 | Similar to uncharacterized protein 1; SSP (aa1-17); 10.3%A, 11.2% Q, 9.5% G, 9.9% L; pI 10.2; IDR (3.9%; aa45-54) | UP1_HALAI**^4^**  (aa1-244) | 69.2 | 1.4e-61 | 4  6  9  11  10  8 | 7  26  38  144  190  149 | 0.032  0.131  0.325  0.125  0.680  0.920 | I_A_  I_B_  I_C_  S_A_  S_B_  S_C_ |
| **Comp89145_c0_seq1_3**  (aa18-263)  UP1_HALAI | Similar to uncharacterized protein 1; 9.1% G, 10.6% L; pI 9.6; IDR (11.3%; aa1-5,55-86) | UP1_HALAI**^4^**  (aa1-244) | 78.9 | 1.4e-75 | 1  -  -  5  -  - | 2  -  -  62  -  - | 0.032  -  -  0.031  -  - | I_A_  I_B_  I_C_  S_A_  S_B_  S_C_ |
| **H_sp_Tri_17455_c0_g1_i1^3^**  (aa1-362)  A0A0B4VCR4_HALAI | Glycine-rich boundary protein; SSP (aa1-23)/TM (aa5-27); 14.1% A, 14.1% Q, 16.0% G, 10.2% M; pI 6.4; IDR (64.6%; aa26-259); if SSP: pI 7.1; 14.2% A, 15.0% Q, 16.8% G, 10.6% M; IDR 68.7; aa176-203 similar to [G-MGA] _7_, aa96-123 [QQQA]_7_ | A0A0B4VCR4  _HALAI  (aa1-343) | 84.8 | 1.0e-36 | 3  4  4  6  5  2 | 6  19  24  109  76  37 | 0.829  3.513  2.691  3.428  3.417  1.028 | I_A_  I_B_  I_C_  S_A_  S_B_  S_C_ |
| **H_sp_Tri_18120_c0_g1_i1^3^**  (aa1-413) | Uncharacterized; 12.7% G, 10.6% P, 16.1% T; pI 5.0; IDP; shares some peptides with Tri_18123; peptides to aa493 | X8J778_9HOMO  (aa96-522) | 27.9 | 1.7e-5 | -  -  -  4  -  - | -  -  -  15  -  - | -  -  -  0.002  -  - | I_A_  I_B_  I_C_  S_A_  S_B_  S_C_ |
| **H_sp_Tri_18123_c0_g1_i1**  (aa13-535) | Uncharacterized; 19.3% T; pI 5.0; IDP; shares some peptides with Tri_18120 | A0A0P5PPH7  _9CRUS  (aa73-599) | 26.1 | 2.8e-10 | -  -  -  1  5  - | -  -  -  7  15  - | -  -  -  <0.001  0.009  - | I_A_  I_B_  I_C_  S_A_  S_B_  S_C_ |
| **H_sp_idb_26836_c0_g1_i1^3^** | Uncharacterized; 13.2% S, 12.5% T; pI 5.1; IDP; shares several peptides with Tri_18123 |  |  |  | -  -  -  -  -  3 | -  -  -  -  -  26 | -  -  -  -  -  0.103 | I_A_  I_B_  I_C_  S_A_  S_B_  S_C_ |
| **H_sp_Tri_18127_c0_g1_i1^3^** | Uncharacterized; 10.0% A, 12.5% S, 11.0% T; pI 6.2; IDP |  |  |  | -  -  4  4  5  6 | -  -  9  24  37  29 | -  -  0.016  0.006  0.014  0.043 | I_A_  I_B_  I_C_  S_A_  S_B_  S_C_ |
| **H_sp_Tri_24151_c0_g1_i1^3^**  (aa1-79)  Comp73368_c0_seq1_5 | Uncharacterized/similar to AP7; SSP (aa1-19); 9.9% L; pI 8.3 | Q9BP37_HALRU**^4^**  (aa1-85) | 43.5 | 0.0005 | 3  4  3  6  6  3 | 22  23  22  173  158  18 | 1.513  1.333  0.873  4.025  2.225  0.241 | I_A_  I_B_  I_C_  S_A_  S_B_  S_C_ |
| **H_sp_Tri_25106_c0_g1_i1^3^**  (aa33-537)  Comp246916_c0_seq1_3 | Similar to shell protein 4¸ SSP (aa1-26)/TM (aa12-31); pI 9.4; compare idb_20988; aa12-21 (IFLL[F,P])_2_ | A0A0G2YN89  _MYTCO**^4^**  (aa39-540) | 35.4 | 8.4e-56 | 20  30  33  18  22  14 | 113  216  238  253  225  115 | 1.191  3.155  2.436  0.074  0.072  0.042 | I_A_  I_B_  I_C_  S_A_  S_B_  S_C_ |
| **H_sp_Tri_28744_c0_g1_i1^3^** | Uncharacterized; domain: CAP (aa209-376); SSP (aa1-25); 11.1% G; pI 9.7; IDR (6.5%; aa147-170) |  |  |  | -  3  6  7  2  4 | -  5  13  69  20  19 | -  0.006  0.018  0.020  0.007  0.006 | I_A_  I_B_  I_C_  S_A_  S_B_  S_C_ |
| **H_sp_Tri_29101_c0_g1_i1^3^** | Uncharacterized; 10.4% A, 12.3% S, 16.7% T, pI 6.8; domain: RmlC-like_jellyroll (aa146-268); IDR (aa1-140); repeats (Fig. S2Za) |  |  |  | 9  11  11  8  8  6 | 46  80  91  128  83  44 | 0.699  1.079  1.593  0.072  0.053  0.047 | I_A_  I_B_  I_C_  S_A_  S_B_  S_C_ |
| **H_sp_Tri_31892_c0_g1_i1^3^**  (aa1-198)  Comp22593_c0_seq1_3 | Similar to ependymin-related protein 1; domain: ependymin (aa71-198); SSP (aa1-17); pI 5.0;shares several peptides with Tri_31898, Tri_31897 and CLC_1876 | EPDR1_HALAI**^4^**  (aa1-198) | 84.3 | 3.1e-198 | 2  3  3  4  5  5 | 9  18  44  116  105  70 | 0.042  0.106  0.422  0.499  1.101  2.113 | I_A_  I_B_  I_C_  S_A_  S_B_  S_C_ |
| **H_sp_Tri_31897_c0_g1_i1^3^**  (aa1-198) | Similar to ependymin-related protein 1; domain: ependymin (aa70-198); SSP (aa1-17); pI 5.1; shares peptide with Tri_31898 and Tri_31892 | EPDR1_HALAI**^4^**  (aa1-198) | 78.3 | 44.3e-74 | -  -  -  4  3  - | -  -  -  19  8  - | -  -  -  0.029  0.005  - | I_A_  I_B_  I_C_  S_A_  S_B_  S_C_ |
| **H_sp_Tri_3408_c0_g1_i1**  (aa11-482)  **H_sp_idb_2652_c0_g1_i1**  Comp100969_c0_seq1_2 | Uncharacterized; domains: EGF (aa26-61), ZP (aa106-368); SSP (aa1-26)/TM (aa10-28), TM (aa430-452); 10.3% T; pI 5.6; IDR (19.0%; aa357-430,470-484); if SSP: pI 5.4; 10.0% T; IDR: 19.0% | V3Z5Y8_LOTGI  Lotgidraft_235547  (aa57-528)  (aa41-528) | 26.4 | 1.2e-35 | -  2  -  6  3  4 | -  3  -  17  8  6 | -  0.002  -  0.002  0.002  0.002 | I_A_  I_B_  I_C_  S_A_  S_B_  S_C_ |
| **H_sp_Tri_35519_c0_g1_i1^3^**  (aa51-376) | Uncharacterized; pI 7.5; domain: ConA_like (aa216-373); TM (aa20-42) | K1QF85_CRAGI  (aa421-782) | 25.8 | 2.5e-10 | 7  8  10  11  12  10 | 26  45  67  177  147  77 | 0.129  0.144  0.166  0.184  0.145  0.090 | I_A_  I_B_  I_C_  S_A_  S_B_  S_C_ |
| **H_sp_Tri_45070_c0_g1_i1^3^**  (aa100-1404)  Comp104254_c0_seq2_4 | Uncharacterized; shares 1 peptide with Tri_127820; not all peptides in alignment; 11.8% P, 12.2% S, 12.3% T, pI 11.0; IDP; short tandem repeats in aa334-343 (STTXP)_2_ , aa500-521 (PSXASXT[S,P])_2_, aa873-883 (TSQ[P,S]T)_2_ | A0A068Y7U9  _ECHMU  (aa9-1297) | 22.1 | 1.7e-13 | 21  25  33  48  45  36 | 79  119  150  855  712  556 | 0.078  0.124  0.192  0.337  0.398  0.580 | I_A_  I_B_  I_C_  S_A_  S_B_  S_C_ |
| **H_sp_Tri_50969_c0_g1_i1^3^**  (aa34-373)  Comp105719_c0_seq1_2  Comp96030_c1_seq6_5 | Uncharacterized; domains: EGF_like (aa59-94,96-131,146-181,183-231,292-334,341-373); 13.7% C,12.8% G, 9.7% S; pI 6.3; IDR (6.5%; aa404-431) | V3ZDP8_LOTGI  Lotgidraft_91483  (aa39-374) | 41.9 | 1.9e-42 | -  4  5  -  -  - | -  7  15  -  -  - | -  0.005  0.015  -  -  - | I_A_  I_B_  I_C_  S_A_  S_B_  S_C_ |
| **H_sp_Tri_57798_c0_g1_i1^3^**  (aa1-528)  *(aa1-220)*  *(aa221-519)* | Similar to molluscan shell protein 1; 12.6% A, 25.4% D, 25.6% G, 9.4% S; pI 3.5; IDP  *Similar to MSI60-related protein/similar to hasinina_P008C13_381***^4^**  *ADG-rich; pI 3.0*  *Similar to Asp- and Gly-rich protein*  *NDG-rich; pI 3.0*  Repeats (Fig. S2Zb) | Q95YF6_MIZYE**^4^**  (aa171-665)  *G9MD31_PINFU*  *(aa185-431)*  *DGRP_HALAI***^4^**  *(aa1-293)* | 32.2  *46.4*  *73.9* | 1.7e-12  *3.0e-8*  *3.8e-35* | 8  8  10  5  6  3 | 76  74  148  41  40  13 | 8.383  4.382  8.644  0.052  0.135  0.130 | I_A_  I_B_  I_C_  S_A_  S_B_  S_C_ |
| **Comp241021_c0_seq1_1^3^**  (aa1-80) | Aspartate and glycine-rich protein (Fragment); 22.5% A, 13.8% R, 28.7% G, 22.5% S; pI 11.8; IDP; shares peptide with Tri_57798 | DGRP_HALAI**^4^**  (aa211-282) | 81.2 | 5.0e-8 | 1  1  1  1  1  1 | 3  2  2  7  5  5 | 0.049  0.005  0.017  0.004  0.007  0.011 | I_A_  I_B_  I_C_  S_A_  S_B_  S_C_ |
| **H_sp_Tri_61496_c0_g1_i1^3^**  (aa23-241) | Similar to ferric-chelate reductase 1; domain: Reeler (47-173); TM (aa21-43); 13.5% S; pI 9.6; IDR (27.4%; aa194-266) | K1Q9X0_CRAGI  (aa1-224) | 29.8 | 4.8e-7 | 9  9  9  9  9  6 | 30  41  60  142  124  74 | 0.462  0.291  0.628  0.119  0.118  0.089 | I_A_  I_B_  I_C_  S_A_  S_B_  S_C_ |
| **H_sp_Tri_62946_c0_g1_i1^3^**  (aa15-735)  Comp99065_c0_seq1_4 | Putative prosaposin; domains: multiple saposin_like/saposinB, saposinA (aa705-738); SSP (aa1-18); 10.6% L, 11.2% V; pI 5.0; IDR (0.8%; aa411-417) | A0A0KJRE48  _IXORI  (aa621-1340) | 31.3 | 4.4e-43 | -  -  2  10  11  3 | -  -  2  79  93  26 | -  -  0.001  0.012  0.021  0.010 | I_A_  I_B_  I_C_  S_A_  S_B_  S_C_ |
| **H_sp_Tri_63049_c0_g1_i1^3^**  (aa64-203) | Similar to putative ferric-chelate reductase 1-like protein; aa54-306 similar to ML7B12**^4^;** domain: Reeler (aa66-226); 10.0% T; pI 10.0; IDR (33.8%; aa30-54,217-286,301-311) | A0A087TMH4  _9ARAC  (aa10-152) | 34.5 | 4.9e-9 | 6  6  13  21  18  11 | 30  37  56  269  255  92 | 0.345  0.336  0.549  0.432  0.579  0.244 | I_A_  I_B_  I_C_  S_A_  S_B_  S_C_ |
| **H_sp_Tri_63812_c0_g1_i1** | Uncharacterized; domains: VWFA (aa284-471, TSP1 (aa470-533,535-587); SSP (aa1-18); 14.6% G, 10.2% S, 9.5% V; pI 4.3; IDR (36.9%; aa24-234); shares peptide with idb_25730 |  |  |  | 3  6  4  2  3  2 | 20  29  20  48  48  22 | 0.192  0.355  0.088  0.034  0.070  0.013 | I_A_  I_B_  I_C_  S_A_  S_B_  S_C_ |
| **H_sp_Tri_64952_c0_g1_i1^3^** | Uncharacterized; SSP (aa1-19); 10.8% R, 10.8% G, 18.5% S, pI 9.7; IDR (43.6%; aa55-140) |  |  |  | 9  8  10  9  6  7 | 48  55  56  125  68  71 | 1.472  0.609  0.684  0.196  0.096  0.219 | I_A_  I_B_  I_C_  S_A_  S_B_  S_C_ |
| **H_sp_Tri_72839_c0_g1_i1^3^**  (aa6-357) | Carbonic anhydrase; domain: Carbonic_anhydrase_a (aa54-352); SSP (aa1-21); pI 8.4; IDR (5.7%; aa239-258) | G0YYQ3_HALTU  (aa1-352) | 78.5 | 8.9e-129 | 9  9  9  7  7  3 | 29  39  56  30  13  4 | 0.396  0.213  0.629  0.010  0.004  0.005 | I_A_  I_B_  I_C_  S_A_  S_B_  S_C_ |
| **H_sp_Tri_73035_c0_g1_i1^3^** | Uncharacterized; TM (aa40-62); 15,8% A, 10.6% G, 14.6% P; pI 6.4; IDP |  |  |  | 7  9  9  12  10  9 | 46  67  73  351  264  170 | 0.767  1.020  1.149  1.381  1.546  1.417 | I_A_  I_B_  I_C_  S_A_  S_B_  S_C_ |
| **H_sp_Tri_7902_c0_g1_i1^3^**  (aa52-258) | Uncharacterized; domains: chitin-bd_II (aa50-113,117-187,209-263); 9.5% S; pI 8.3 | V3ZHU5_LOTGI  Lotgidraft_169029  (aa28-239) | 45.9 | 4.5e-46 | -  -  -  2  4  2 | -  -  -  3  16  2 | -  -  -  0.001  0.015  0.001 | I_A_  I_B_  I_C_  S_A_  S_B_  S_C_ |
| **H_sp_Tri_81308_c0_g1_i1^3^** | Uncharacterized; pI 8.8; shares peptide with comp95780_c0_seq2_4 |  |  |  | 3  2  2  3  3  2 | 13  13  15  69  42  35 | 0.125  0.157  0.116  0.260  0.324  0.171 | I_A_  I_B_  I_C_  S_A_  S_B_  S_C_ |
| **H_sp_Tri_83476_c0_g1_i1^3^**  (aa5-554) | Uncharacterized; SSP (aa1-21); pI 5.4; IDR (12.8%; aa486-554) | V4A4X8_LOTGI**^4^**  Lotgidraft_233348  (aa34-592) | 29.1 | 2.1e-63 | 4  10  10  15  14  13 | 5  30  33  220  189  123 | 0.004  0.047  0.028  0.087  0.127  0.064 | I_A_  I_B_  I_C_  S_A_  S_B_  S_C_ |
| **H_sp_Tri_8786_c0_g1_i1**  (aa28-159) | Similar to complement C1q-like protein 4; domain: C1q/TNF_like (aa28-164);SSP (aa1-30); 10.3% L; pI 9.1 | K1QIM9_CRAGI  (aa61-191) | 38.8 | 4.4e-10 | 3  -  5  3  -  3 | 7  -  25  11  -  8 | 0.030  -  0.149  0.006  -  0.007 | I_A_  I_B_  I_C_  S_A_  S_B_  S_C_ |
| **H_sp_Tri_90659_c0_g1_i1^3^** | Uncharacterized; SSP (aa1-23); 13.9% R; pI 10.6 |  |  |  | -  -  -  7  5  - | -  -  -  17  23  - | -  -  -  0.010  0.040  - | I_A_  I_B_  I_C_  S_A_  S_B_  S_C_ |
| **PLS_HALLA^4^**  (P82595) | Perlustrin; domain: IGFBP_N (aa1-81; 14.3% C; pI 8.0 |  |  |  | -  2  -  2  2  - | -  10  -  19  24  - | -  1.952  -  0.139  0.487  - | I_A_  I_B_  I_C_  S_A_  S_B_  S_C_ |
| **PLC_HALLA^3,4^**  (P82596) | Perlucin; domain: C-type_lectin-like (aa1-141); pI 7.2; shares most peptides with F8J3D2 (perlucin C) and 1 with comp25997_c0_seq1_1; pI 7.2; IDR (9.7%; aa141-155) |  |  |  | 4  18  14  21  19  3 | 43  330  231  916  630  39 | 4.362  18.502  8.568  7.440  8.119  0.054 | I_A_  I_B_  I_C_  S_A_  S_B_  S_C_ |
| **Comp25997_c0_seq1_1^3^**  (aa1-88) | Similar to perlucin; domain: C-type_lectin_like (aa1-88); 11.4% R, 9.1% E, 10.2% G, 10.2% L; pI 7.1; shares several peptides with PLC_HALLA and perlucin C (F8J3D2) | PLC_HALLA**^4^**  (aa42-129) | 65.9 | 1.3e-25 | 1  1  11  1  1  1 | 3  3  8  20  17  1 | 0.103  0.108  0.053  0.090  0.111  0.003 | I_A_  I_B_  I_C_  S_A_  S_B_  S_C_ |
| **F8J3C9_HALLA^3^** | Perlucin A; domain: C-type_lectin-like (aa27-146), SSP (aa1-18); pI 6.5; shares several peptides with F8J3D2_HALLA and PLC_HALLA |  |  |  | -  -  2  -  -  2 | -  -  14  -  -  5 | -  -  0.273  -  -  0.004 | I_A_  I_B_  I_C_  S_A_  S_B_  S_C_ |
| **F8J3D2_HALLA^3^** | Perlucin C; domain: C-type_lectin-like (aa27-146); SSP (aa1-18); 9.5% Q, 13.1% L; pI 6.7; IDR (14.9%; aa207-240); shares most peptides with perlucin (PLC_HALLA) , perlucin A, and 1 with comp25997_c0_seq1_1 |  |  |  | 16  4  3  5  4  11 | 415  404  125  414  281  361 | 6.464  1.983  0.568  0.752  0.500  1.852 | I_A_  I_B_  I_C_  S_A_  S_B_  S_C_ |
| **PWAP_HALLA^3,4^**  (P84811) | Perlwapin; domains: WAP (aa2-43,44-89,90-132); 18.7% C, 11.9% G, 15.7% P; pI 8.6; shares 2 peptides with Comp236269_c0_seq1_4 |  |  |  | 6  8  8  13  12  7 | 28  46  36  221  201  89 | 1.757  2.225  1.838  3.765  4.004  2.853 | I_A_  I_B_  I_C_  S_A_  S_B_  S_C_ |
| **Comp236269_c0_seq1_4^3^**  (aa1-82) | Similar to perlwapin; domains: WAP (aa1-32,35-80); 19.5% C, 11.5% G, 12.6% P; pI 8.6; IDR (5.6%; aa1-5); shares 2 peptides with PWAP_HALLA | PWAP_HALLA**^4^**  (aa71-152) | 92.7 | 1.4e-30 | -  3  4  2  6  5 | -  26  42  11  67  45 | -  0.513  0.803  0.017  0.451  2.340 | I_A_  I_B_  I_C_  S_A_  S_B_  S_C_ |
|  |  |  |  |  |  |  |  |  |

**^1^**, the database entry(s) with the highest number of peptides (majority protein) is shown first, irrespective of the database of origin; the best matching entry of the respective other databases is added in bold print if identified with the same number of peptides, in normal print if identified with fewer peptides; additional entries in the same group but with fewer peptides are available in the respective Additional ProteinGroup files. **^2^**, I, acetic-acid insoluble; S, acetic acid-soluble; A, shell washed with sodium hypochlorite before demineralization; B, hypochlorite washing with short sonication intervals; C, no chemical treatment but sand-blasting of the shell on both sides. **^3^**, also identified in prismatic layer. **^4^**, previously identified in the shell proteome of *Haliotis laevigata* (_HALLA) [14,16,19], of *H. asinina* (_HALAI) [21], of *H. rufescence* (_HALRU) [12,17], of *H. tuberculata* [22], of the limpet *Lottia gigantea* (_LOTGI) [58,69,105], the oyster *Crassostrea gigas* (_CRAGI) [133], the slug *Arion vulgaris* (_9EUPU) [136], the mussel *Mytilus californianus* (_MYTCA) [23], the mussle *Mytilus coruscus* (_MYTCO) [25], the oyster *Pinctada maxima* (_PINMA) [122], and the scallop Mizuhopecten yessoensis (_MIZYE) [76]. Amino acid positions and the data in columns 2 to 6 refer to the majority protein. Predicted domains are abbreviated according to InterPro (<http://www.ebi.ac.uk/interpro/>). SSP, predicted secretion signal peptide; TM, predicted transmembrane helix. IDP, predicted intrinsically disordered protein (predicted disorder <90%); IDR, predicted intrinsically disordered sequence regions. Composition, pI and IDR percentages were calculated without predicted signal peptide sequence. Only
