# Supplementary material for: In-depth proteomic analyses of Haliotis laevigata (greenlip abalone) nacre and prismatic organic shell matrix
Source: Proteome Sci. 2018 Jun 15;16:11. doi: 10.1186/s12953-018-0139-3 (PMC6003135; doi:10.1186/s12953-018-0139-3)
Supplement: Supplementary file 5 — Table S3. Prismatic layer proteins. docx-file listing all accepted identifications of Haliotis laevigata prismatic layer proteins including most similar database matches, number of identified peptides and abundance in different shell fractions. (DOCX 309 kb) [file 12953_2018_139_MOESM5_ESM.docx]

**Table S3**

***Haliotis laevigata* proteins identified in the prismatic shell layer**

| **Accession ^1^** | **Protein** | **Highest scoring**  **FASTP match**  (maximal allowed e-value of 1.0e-4) | **% iden-tity** | **FASTPe-value** | **Unique and razor peptides** | **Total peptides** | **% of total**  **(iBAQ)** | **Frac-tion^2^** |
| --- | --- | --- | --- | --- | --- | --- | --- | --- |
|  |  |  |  |  |  |  |  |  |
| **H_sp_Tri_99928_c0_g1_i1**  (aa6-811)  **comp100001_c0_seq2_1**  **comp100001_c0_seq3_3** | Uncharacterized; domain: SRCR(_like) (aa610-710,710-812); SSP (aa1-22); 9.6% L, 9.5% S; pI 6.4; IDR (5.8%; aa242-253,374-409) | A0A0B7B1Y9  _9EUPU  (aa12-820) | 35.0 | 7.9e-61 | 3  2  -  - | 8  2  -  - | <0.001  <0.001  -  - | I_A_  I_B_  S_A_  S_B_ |
| **Comp100163_c2_seq1_3**  (aa65-1087)  H_sp_idb_3766_c0_g1_i1 | Uncharacterized; pI 8.8; IDR (3.0%; aa1023-1059) | V3ZRI9_LOTGI  Lotgidraft_153519  (aa180-1175) | 23.4 | 1.2e-43 | 17  21  -  - | 70  105  -  - | 0.004  0.008  -  - | I_A_  I_B_  S_A_  S_B_ |
| **H_sp_Tri_2667_c0_g1_i1**  (aa13-465)  **Comp100525_c0_seq1_6** | Uncharacterized/similar to plasma glutamate carboxypeptidase; domain: Peptidase_M28 (aa264-450); SSP (aa1-24); pI 5.5 | C3XQU6_BRAFL  (aa9-457) | 67.1 | 1.9e-129 | -  2  -  - | -  6  -  - | -  0.001  -  - | I_A_  I_B_  S_A_  S_B_ |
| **H_sp_idb_11640_c0_g1_i1**  (aa1-460)  **Comp107184_c2_seq4_5**  **etc** | Uncharacterized/similar to plasma glutamate carboxypeptidase; domain: peptidase_M28 (aa259-439); SSP (aa1-17); pI 5.4; IDR (2.2%; aa459-469) | C3XQU6_BRAFL  (aa1-457) | 60.9 | 1.8e-123 | 2  2  -  - | 6  15  -  - | 0.001  0.002  -  - | I_A_  I_B_  S_A_  S_B_ |
| **H_sp_idb_30234_c0_g1_i1**  (aa77-711)  **H_sp_idb_30235_c0_g1_i1**  **Comp101142_c0_seq1_1**  **Comp101142_c0_seq2_1** | Similar to Xaa-Pro aminopeptidase 1; domains: creatinase_N (aa77-200), Pept_M24_structural (388-652), peptidase_M24_C (aa626-685); SSP (aa1-22); pI 6.3; IDR (3.6%; aa29-54) | K1QMF0_CRAGI  (aa6-641) | 57.5 | 3.6e-159 | 9  6  -  - | 40  50  -  - | 0.005  0.005  -  - | I_A_  I_B_  S_A_  S_B_ |
| **H_sp_idb_10731_c0_g1_i1**  (aa18-693)  Comp101219_c0_seq2_6  Comp101219_c0_seq2_5 | Uncharacterized/similar to Galactocerebrosidase; domain: Glycoside_hydrolase_SF (aa47-344); TM (aa13-35); 9.7% G; pI 5.4; IDR (7.9%; aa1-12,477-488,680-712) | A0A0B7BI09  _9EUPU  (aa10-688) | 58.3 | 8.1e-181 | 3  5  -  - | 10  39  -  - | 0.001  0.006  -  - | I_A_  I_B_  S_A_  S_B_ |
| **H_sp_Tri_109450_c0_g1_i1^3^**  (aa5-229)  Comp101252_c0_seq3_2 | Uncharacterized; domains: chitin-bd_II (aa19-85,83-152); SSP (aa1-17); 9.1% C; pI 8.0 | K1R0D4_CRAGI  (aa2-228) | 45.4 | 4.6e-41 | 9  9  -  - | 70  72  -  - | 0.025  0.028  -  - | I_A_  I_B_  S_A_  S_B_ |
| **H_sp_idb_11270_c0_g1_i1^3^**  (aa1-633)  H_sp_idb_11270_c0_g1_il  Comp101254_c0_seq1_3  **etc** | Similar to HSP70; domains: HSP70_peptide_binding (aa388-544), HSP70_Cterm (aa519-622); pI 5.5; IDR (23.4%; aa490-633); shares several peptides with comp79549_c0_seq1_2 and Tri_2770 | S4U403_9GAST  (aa1-635) | 79.8 | 3.4e-300 | 1  1  -  - | 4  4  -  - | <0.001  <0.001  -  - | I_A_  I_B_  S_A_  S_B_ |
| **H_sp_CLC_351_c0_g1_i1**  (aa18-175)  **Comp101468_c1_seq1_3** | Actin depolymerisation factor/cofilin; domains: ADF-H/Gelsolin-like (aas20-164); 11.4% K, 11.4% S; pI 8.3; IDR (15.3%; aa1-28) | B3SND4_HALDV  (aa1-558) | 81.6 | 1.9e-51 | 2  3  -  - | 6  26  -  - | 0.002  0.008  -  - | I_A_  I_B_  S_A_  S_B_ |
| **H_sp_Tri_88331_c0_g1_i1^3^**  (aa67-452)  **Comp101550_c1_seq1_3** | Similar to endoplasmic reticulum resident protein ERp44; domains: Thioredoxin-like_fold (aa48-177,172-268,265-395); pI 6.3; IDR (19.5%; aa365-452) | K1P6B3_CRAGI  (aa7-394) | 63.8 | 6.1e-110 | 9  10  -  - | 30  46  -  - | 0.006  0.006  -  - | I_A_  I_B_  S_A_  S_B_ |
| **Comp101565_c0_seq1_5^3^**  (aa122-1182) | Similar to hephaestin-like protein; domains: cupredoxin (aa21-208,218-355,364-561,564-709,721-906,918-1064); pI 6.1; shares most peptides with idb_10164; IDR (0.5%; aa961-968) | HEPHL_ACRMI  (aa8-1076) | 45.7 | 0e0 | 22  -  -  - | 204  -  -  - | 0.010  -  -  - | I_A_  I_B_  S_A_  S_B_ |
| **H_sp_idb_10164_c0_g1_i1^3^**  (aa2-1062) | Similar to hephaestin-like protein; domains. cupredoxin (aa21-208,218-355,364-561,564-709,721-906,918-1064); SSP (aa1-22); pI 5.2; ; IDR (1.0%; aa132-143) | HEPHL_ACRMI  (aa8-1076) | 45.7 | 0e0 | 1  21  -  - | 9  208  -  - | 0.001  0.021  -  - | I_A_  I_B_  S_A_  S_B_ |
| **Comp101638_c0_seq5_5^3^**  (aa4-358)  **Comp101638_c0_seq1_5**  **Comp101638_c0_seq4_5**  H_sp_idb_21744_c0_g1_i1 | Uncharacterized; domains: ZP (aa3-291); TM (aa320-342,357-379,392-414,429-461,511-533); pI 8.4; IDR (5.9%; aa1-22,288-297) | C3Z6R0_BRAFL  (aa187-533) | 28.9 | 2.1e-26 | 4  2  -  - | 45  12  -  - | 0.018  0.002  -  - | I_A_  I_B_  S_A_  S_B_ |
| **Comp101644_c0_seq2_4^3^**  (aa35-1158) | Similar to thioester-containing protein/CD109-like; domains: A2M_N_2 (aa135-269), Macroglobln_a2 (aa388-475), A2M_comp (609-950), A-macroglobulin_rcpt-bd (aa1019-1137); pI 6.1; share almost all peptides; IDR (5.2%; aa1-71,779-794) | D4QA02_9CNID  (aa375-1475) | 34.3 | 3.2e-89 | 1  40  1  - | 12  590  1  - | <0.001  0.081  <0.001  - | I_A_  I_B_  S_A_  S_B_ |
| **H_sp_CLC_1485_c0_g1_i1^3^**  (aa3-1513) | Similar to thioester-containing protein(-E); SSP (aa1-17); domains: A2M_N (aa126-207), A2M_N_2 (aa569-630), A2M (aa749-836), A2M_compl (aa966-1311), A-macroglobulin_rcpt-bd (aa1380-1498); IDR (3.6%; aa409-463) | D5FT53_9BIVA  (aa2-1461) | 35.2 | 7.1e-108 | 58  -  -  - | 751  -  -  - | 0.062  -  -  - | I_A_  I_B_  S_A_  S_B_ |
| **Comp101644_c1_seq1_1^3^**  (aa48-430) | Similar to thioester-containing protein(-G); domain: A2M_N (aa171-259); TM (aa36-58); pI 9.0; share almost all peptides | D5FT55_9BIVA  (aa2-363) | 38.9 | 2.5e-38 | 1  15  1  1 | 24  236  1  4 | 0.006  0.120  <0.001  0.005 | I_A_  I_B_  S_A_  S_B_ |
| **H_sp_idb_12235_c0_g1_i1**  (aa24-939) | Uncharacterized; domains: A2M_N (aa126-206), A2M_N_2 (aa522-658), A2M (aa777-864); pI 6.2; IDR (6.9%; (aa396-458,758-762) shares most peptides with comp101644_c0_  seq2_4 and CLC_1485 | A0A0B7BG66  _9EUPU  (aa42-984) | 29.8 | 2.9e-59 | 2  -  1  - | 27  -  1  - | 0.001  -  <0.001  - | I_A_  I_B_  S_A_  S_B_ |
| **H_sp_idb_39269_c0_g1_i1**  (aa8-504)  **Comp101668_c0_seq7_2** | Similar to aldehyde dehydrogenase; domains: Ald_DH_N (aa26-286), Ald_DH_C (aa287-479); pI 5.9; IDR (3.6%; aa1-18) | V4A3P4_LOTGI  Lotgidraft_218707  (aa1-498) | 64.3 | 1.6e-139 | 5  5  -  - | 17  15  -  - | 0.002  0.003  -  - | I_A_  I_B_  S_A_  S_B_ |
| **H_sp_idb_10046_c0_g1_i1^3^**  (aa1-572)  **Comp101858_c3_seq10_6**  **etc** | Similar to pyruvate kinase; domains: Pyrv_Knase_brl (aa70-417, Pyrv_Knase_a/b (aa400-550); pI 6.1; shares 1 peptide with Comp101858_c3_seq18_4 | V4AYD4_LOTGI  Lotgidraft_199626  (aa1-599) | 72.5 | 4.1e-167 | 8  8  -  - | 59  37  -  - | 0.007  0.006  -  - | I_A_  I_B_  S_A_  S_B_ |
| **Comp101858_c3_seq18_4^3^**  (aa1-523) | Pyruvate kinase; domains: Pyrv_Knase_brl (aa7-386), Pyrv_Knase_C (aa364-523); pI 8.1; shares 1 peptide with idb_10046 | A1L3K2_XENLA  (aa9-531) | 100.0 | 0e0 | 2  2  -  - | 2  3  -  - | <0.001  0.001  -  - | I_A_  I_B_  S_A_  S_B_ |
| **H_sp_Tri_110769_c0_g1_i1**  (aa1-125)  **Comp102102_c1_seq1_6** | Histidine triad nucleotide binding protein; domain: HIT-like (aa13-124); 9.6% G, 11.2% V; pI 5.7; IDR (10.4%; aa1-13) | E6Y2Y9_HALDV  (aa1-125) | 87.2 | 8.3e-49 | 2  3  -  - | 2  6  -  - | 0.001  0.002  -  - | I_A_  I_B_  S_A_  S_B_ |
| **H_sp_CLC_2877_c0_g1_i1^3^**  (aa16-850)  **H_sp_idb_3129_c0_g1_i1**  **Comp102281_c0_seq4_1**  **etc** | Similar to dystroglycan; domains: Cadherin-like (aa70-160,420-522), Alpha-dystroglycan_domain_2 (aa182-303), SEA_DG (aa530-638); TM (aa12-34,707-729); pI 6.0; ; IDR (22.6%; aa302-323,640-706,748-850) | V4B4U7_LOTGI  Lotgidraft_224800  (aa1-831) | 45.1 | 3.0e-73 | 2  2  -  - | 11  12  -  - | 0.001  0.002  -  - | I_A_  I_B_  S_A_  S_B_ |
| **H_sp_Tri_133681_c0_g1_i1**  (aa11-410)  **Comp102412_c0_seq2_5**  **Comp102412_c0_seq1_5** | Similar to aspartate aminotransferase; domain: Aminotransferase_I/II (aa11-410); pI 7.2; IDR (2.6%; aa1-11) | V4ASZ2_LOTGI  Lotgidraft_231665  (aa2-402) | 67.3 | 8.3e-121 | 3  5  -  - | 5  18  -  - | 0.001  0.003  -  - | I_A_  I_B_  S_A_  S_B_ |
| **H_sp_idb_8870_c0_g1_i1**  (aa103-1203)  **Comp102820_c2_seq56_4**  **etc** | Similar to Golgi apparatus protein 1; domains: multiple Cys-rich_GLG1_repeats; SSP (aa1-27), TM (aa1170-1192); pI 6.4; IDR (12.7%; aa28-136,601-615,916-922,1034-1051,1199-1203) | A0A151XHS3  _9HYME  (aa26-1129) | 38.8 | 3.8e-184 | 6  5  -  - | 16  8  -  - | 0.001  <0.001  -  - | I_A_  I_B_  S_A_  S_B_ |
| **H_sp_Tri_129753_c0_g1_i1^3^**  (aa1-144)  **Comp102930_c0_seq1_5** | Uncharacterized; domain: UspA (aa1-147); 9.5% G, 10.8% V ; pI 7.8 | A0A0B6ZQD9_  9EUPU  (aa1-145) | 45.5 | 9.6e-23 | 6  7  -  - | 120  126  -  - | 0.136  0.320  -  - | I_A_  I_B_  S_A_  S_B_ |
| **Comp103103_c0_seq1_2**  (aa89-549)  H_sp_Tri_74375_c0_g1_i1 | Uncharacterized/similar to thymus-specific serine protease; domain: peptidase_S28_fam (aa111-541); TM (aa55-77,730-747); pI 6.3; IDR (2.7%; aa83-102) | C3ZCY8_BRAFL  (aa1-462) | 65.7 | 9.2e-147 | 5  6  -  - | 43  52  -  - | 0.005  0.007  -  - | I_A_  I_B_  S_A_  S_B_ |
| **H_sp_idb_29361_c0_g1_i1**  (aa122-443)  (aa7-441)  Comp103134_c0_seq6_1  etc | Purple acid phosphatase; domains: Purple_acid_Pase-like_N (aa25-123), Metallo-depent_PP-like (aa126-431); SSP (aa1-19); pI 5.2 | M4R4H4_HALDV  (aa1-322)  A0A0L8GU12  _OCTBM  (aa9-442) | 93.8  55.6 | 4.8e-143  1.2e-113 | 2  3  -  - | 18  24  -  - | 0.002  0.004  -  - | I_A_  I_B_  S_A_  S_B_ |
| **H_sp_idb_8137_c0_g1_i1**  (aa13-362)  **Comp103344_c0_seq1_6** | Uncharacterized; domain: DNAse_II_fam (aa4-362); TM (aa9-31; SSP?); 11.0% S; pI 8.5; IDR (1.0%; aa380-384) | R7USA3_CAPTE  (aa5-361) | 48.3 | 5.5e-74 | 2  3  -  - | 2  6  -  - | <0.001  0.002  -  - | I_A_  I_B_  S_A_  S_B_ |
| **H_sp_CLC_8815_c0_g1_i1^3^**  **H_sp_idb_19915_c0_g1_i1**  **Comp102251_c1_seq6_2**  **etc** | Uncharacterized; domains: Pan_app (aa26-97,185-251,252-326); SSP (aa1-27); 13.5% S, 22.2% T; pI 5.6; IDR (40.6%; aa28-54,85-200) |  |  |  | 2  2  -  - | 3  2  -  - | 0.001  0.001  -  - | I_A_  I_B_  S_A_  S_B_ |
| **Comp103384_c0_seq2_4^3^**  (aa31-231) | Uncharacterized; domain: ependymin_fam (aa106-229); TM (aa22-40,255-277,314-336); IDR (2.8%; aa1-11); pI 8.7; shares most peptides with Tri_16732 | V4BP88_LOTGI**^4^**  Lotgidraft_233583  (aa10-215) | 33.5 | 1.3e-22 | 6  7  -  - | 13  27  -  - | 0.003  0.004  -  - | I_A_  I_B_  S_A_  S_B_ |
| **H_sp_Tri_16732_c0_g1_i1^3^**  (aa9-209)  Comp100491_c1_seq1_3 | Uncharacterized; shares most peptides with comp103384_c0_seq2_4; domain: ependymin (aa83-207); SSP (aa1-22); pI 9.0 | V4BP88_LOTGI**^4^**  Lotgidraft_233583  (aa10-215) | 33.3 | 4.4e-21 | 1  1  -  - | 1  5  -  - | <0.001  <0.001  -  - | I_A_  I_B_  S_A_  S_B_ |
| **H_sp_idb_4412_c0_g1_i1^3^**  (aa31-229) | Uncharacterized; domain: ependymin (aa88-212); SSP (aa4-26)/TM (aa4-26); TM (aa157-179); pI 9.2; shares 1 peptide with Tri_16732; if SSP: 9.3 | V4BP88_LOTGI**^4^**  Lotgi1\|233583  (aa32-233) | 30.2 | 3.6e-19 | 3  3  -  - | 10  13  -  - | 0.001  0.005  -  - | I_A_  I_B_  S_A_  S_B_ |
| **H_sp_Tri_23898_c0_g1_i1^3^**  (aa21-190)  **Comp103559_c0_seq2_2**  **etc** | Similar to leukocyte cell-derived chemotaxin 1-like protein; domain: BRICHOS (aa75-162); SSP (aa1-30); 11.0% V; pI 5.2 | D5FW85_HALDI  (aa7-173) | 30.1 | 3.6e-7 | 2  6  -  - | 4  33  -  - | 0.001  0.015  -  - | I_A_  I_B_  S_A_  S_B_ |
| **H_sp_CLC_9241_c0_g1_i1**  (aa13-659)  **Comp103563_c0_seq8_1**  **Comp103563_c0_seq7_1** | Similar to beta-galactosidase; domains: Glycoside_hydrolase_SF (aa29-360), Galactose-bd-like (aa479-640); SSP (aa1-28); pI 6.1; IDR (2.8%; aa643-661) | V3ZJV5_LOTGI  Lotgidraft_236200  (aa6-656) | 64.6 | 5.1e-182 | 2  3  -  - | 12  16  -  - | 0.001  0.001  -  - | I_A_  I_B_  S_A_  S_B_ |
| **H_sp_Tri_107222_c0_g1_i1**  (aa7-609)  **Comp103663_c0_seq1_3**  **Comp103663_c0_seq2_3** | Uncharacterized/similar to plastin; domains: EF_hand (aa4-83), CH (aa112-610); pI 5.2; IDR (11.5%; aa1-12,79-113,608-633) | A0A0B6ZVP3  _9EUPU  (aa17-619) | 55.5 | 3.9e-140 | 5  6  -  - | 23  37  -  - | 0.001  0.003  -  - | I_A_  I_B_  S_A_  S_B_ |
| **H_sp_idb_43226_c0_g1_i1**  (aa1-392)  **Comp103713_c1_seq2_5**  Comp88085_c0_seq2_2**^3^** | Alpha-tubulin; domains: Tubulin_FtsZ_GTPase (3-169, Tub_FtsZ_C (202-391); pI 5.5 | V3ZLS0_LOTGI  Lotgidraft_236629  (aa45-436) | 98.2 | 4.9e-172 | 7  2  -  - | 71  8  -  - | 0.008  0.004  -  - | I_A_  I_B_  S_A_  S_B_ |
| **Comp103866_c0_seq6_3**  (aa39-597)  H_sp_Tri_8762_c0_g1_i1  **etc** | Uncharacterized; domain: Haem_peroxidase_SF (aa51-590); SSP (aa1-20); pI 4.7; IDR (5.6%; aa53-64,151-156,177-186,240-244,606-610) | V4B1N2_LOTGI  Lotgidraft_238662  (aa61-628) | 34.1 | 7.7e-66 | 7  5  -  - | 31  29  -  - | 0.002  0.006  -  - | I_A_  I_B_  S_A_  S_B_ |
| **Comp103936_c0_seq2_3**  **Comp103936_c0_seq1_3**  H_sp_Tri_1106_c0_g1_i1 | Uncharacterized; domain: Beta-lactam-related (aa68-383); TM (aa809-831); pI 8.6 | A0A0L8HMP7  _OCTBM  (aa30-686) | 35.4 | 1.7e-90 | 7  8  -  - | 29  28  -  - | 0.002  0.001  -  - | I_A_  I_B_  S_A_  S_B_ |
| **H_sp_idb_7892_c0_g1_i1^3^**  (aa12-365)  **Comp104004_c0_seq4_5**  **Comp104004_c0_seq2_5** | Uncharacterized; domain: Renin_receptor-like (aa9-365); SSP (aa1-29), TM (aa324-346); 11.9% L; pI 6.4; IDR (8.3%; aa286-314) | V4CR93_LOTGI  Lotgidraft_211795  (aa12-365) | 61.1 | 5.2e-86 | 2  2  -  - | 4  3  -  - | 0.001  0.001  -  - | I_A_  I_B_  S_A_  S_B_ |
| **H_sp_idb_5674_c0_g1_i1^3^**  (aa131-626)  **Comp104092_c0_seq1_6** | Polypeptide N-acetylgalactosaminyl-transferase; domains: Glyco_trans_2-like (aa196-380), Ricin_B_lectin (aa500-624); TM (aa12-34); pI 7.6; not all peptides in alignment! IDR (21.7%; aa32-160,627-632) | V3ZHH4_LOTGI**^4^**  Lotgidraft_123129  (aa1-496) | 83.5 | 2.0e-198 | 15  14  -  - | 99  102  -  - | 0.008  0.014  -  - | I_A_  I_B_  S_A_  S_B_ |
| **H_sp_idb_7864_c0_g1_i1**  (aa87-651)  **Comp104220_c0_seq2_1**  **Comp104220_c0_seq3_1** | Similar to L-ascorbate oxidase; domains: multiple cupredoxin; SSP (aa1-36); pI 5.9; IDR (2.6%; aa577-594) | K1QA40_CRAGI  (aa1-571) | 49.3 | 3.5e-116 | 2  4  -  - | 14  25  -  - | 0.001  0.002  -  - | I_A_  I_B_  S_A_  S_B_ |
| **Comp104253_c0_seq4_4**  (aa37-247)  H_sp_CLC_28367_c0_g1_i1 | Similar to X-box binding protein; domain: ependymin_fam (aa1-206); pI 9.0; IDR (1.9%; aa1-7) | B6RB39_HALDI  (aa1-206) | 30.0 | 1.8e-13 | 6  6  -  - | 124  72  -  - | 0.055  0.049  -  - | I_A_  I_B_  S_A_  S_B_ |
| **Comp104383_c0_seq1_2**  (aa1-944) | Uncharacterized; shares most peptides with idb_1397; domains: IG (aa258-342,874-944), FN3 (aa363-544); 9.2% G, 10.3% S, 9.6% T; pI 5.2 | V4B5J1_LOTGI**^4^**  Lotgidraft_168990  (aa2006-2954) | 44.2 | 6.9e-175 | 1  3  -  - | 7  16  -  - | <0.001  0.002  -  - | I_A_  I_B_  S_A_  S_B_ |
| **H_sp_idb_1397_c0_g1_i1**  Comp99390_c0_seq1_2  Comp106765_c0_seq2_5  Comp107330_c0_seq2_4  Comp89292_c0_seq1_4 | Uncharacterized; domains: multiple FN3; SSP (aa1-23); 11.0% S, 9.4% T; 9.5% V; pI 5.9; IDR (0.8%; aa721-724,1437-1440,813-830); shares most peptides with comp104383_c0_seq1_2 and Tri_34683 | V4B5J1_LOTGI**^4^**  Lotgidraft_168990  (aa173-3922) | 36.3 | 0e0 | 36  41  -  - | 127  190  -  - | 0.005  0.007  -  - | I_A_  I_B_  S_A_  S_B_ |
| **H_sp_Tri_34683_c0_g1_i1**  (aa1-2679) | Uncharacterized; domains: multiple Ig-like_fold/FN3; 10.5% V, pI 5.5; IDR (0.6%; aa109-123); shares most peptides with idb_1397 | V4B5J1_LOTGI**^4^**  Lotgidraft_168990  (aa1006-3924) | 37.0 | 0e0 | -  1  -  - | -  2  -  - | -  <0.001  -  - | I_A_  I_B_  S_A_  S_B_ |
| **Comp86036_c0_seq1_4**  (aa2-271) | Uncharacterized; pI 6.0; shares 2 peptides with idb_1397 | V4BIX5_LOTGI  Lotgidraft_165393  (aa720-989) | 53.1 | 5.0e-69 | 1  -  -  - | 2  -  -  - | 0.001  -  -  - | I_A_  I_B_  S_A_  S_B_ |
| **Comp97911_c1_seq1_4**  (aa1-565) | Uncharacterized; 11.2% V; pI 5.7; shares most peptides with idb_1397 | K1Q7J3_CRAGI  (aa29-592) | 38.4 | 4.8e-88 | 2  1  -  - | 8  2  -  - | 0.002  <0.001  -  - | I_A_  I_B_  S_A_  S_B_ |
| **H_sp_idb_6309_c0_g1_i1**  (aa5-820)  **Comp104422_c0_seq2_4**  **Comp104422_c0_seq1_6** | Similar to villin-1; domains: multiple ADF-H/Gelsolin-like; pI 5.5; IDR (6.3%; aa204-248,609-615) | K1RGK4_CRAGI  (aa3-819) | 63.8 | 0e0 | 5  8  -  - | 28  34  -  - | 0.001  0.003  -  - | I_A_  I_B_  S_A_  S_B_ |
| **H_sp_CLC_14282_c0_g1_i1^3^**  (aa136-654)  **Comp104776_c0_seq5_3**  **etc** | Polypeptide N-acetylgalactosaminyl transferase; domains: Nucleotide-diphossugar_trans (aa187-517); ricin_B_lectin (aa523-648); TM (aa45-67); pI 8.1; IDR (21.5%; aa1-35,72-177) | V3ZR56_LOTGI  Lotgidraft_194702  (aa1-519) | 75.3 | 3.7e-196 | 2  2  -  - | 12  11  -  - | 0.001  0.002  -  - | I_A_  I_B_  S_A_  S_B_ |
| **Comp104779_c0_seq1_6**  **Comp104779_c0_seq2_4** | Uncharacterized multi-domain protein domains: HYR (aa574-659,660-743,1877-1955), multiple EGF, Sushi_SCR_CCP (aa744-811,1959-2011), Growth_fac_rcpt (aa921-1079,1483-1613,2148-2315), ConA-like (aa1658-1829); TM (aa2475-2497); pI 4.4; IDR (6.0%; aa1-43,536-599,2511-2559) |  |  |  | 7  7  -  - | 14  14  -  - | 0.001  0.001  -  - | I_A_  I_B_  S_A_  S_B_ |
| **Comp105092_c0_seq2_6**  (aa179-571)  **H_sp_CLC_8052_c0_g1_i1**  **Comp105092_c0_seq1_4** | Uncharacterized; domain: RNase_K (aa203-571); pI 8.0; IDR (4.7%; aa1-28) | V4ANV5_LOTGI  Lotgidraft_158797  (aa25-411) | 50.4 | 1.2e-91 | 2  2  -  - | 3  3  -  - | <0.001  <0.001  -  - | I_A_  I_B_  S_A_  S_B_ |
| **Comp105353_c0_seq8_3**  (aa36-586)  H_sp_idb_13449_c0_g1_i1  **etc** | Uncharacterized/asparaginyl-tRNA synthetase, cytoplasmic-like; domains: nucleic_acid-binding_OB-fold (aa139-250), aa-tRNA-synth_II (aa264-579); TM (aa673-695,930-949); pI 7.9; IDR (9.2%; aa38-98,121-132,820-840) | V4AZ14_LOTGI  Lotgidraft_200884  (aa11-558) | 73.9 | 2.6e-191 | 6  8  -  - | 30  28  -  - | 0.002  0.003  -  - | I_A_  I_B_  S_A_  S_B_ |
| **Comp105805_c2_seq17_3**  (aa36-356)  **H_sp_Tri_10477_c0_g1_i1**  **etc** | Similar to lysosomal aspartic protease; domain: cathepsin_D_fam (aa33-357); 9.8% G, 9.0% S; pI 6.8 | ASPP_AEDAE  (aa22-342) | 63.6 | 7.0e-89 | 4  5  -  - | 28  31  -  - | 0.004  0.004  -  - | I_A_  I_B_  S_A_  S_B_ |
| **H_sp_idb_30241_c0_g1_i1^3^**  **Comp106275_c0_seq2_4** | Uncharacterized; SSP (aa1-20); pI 6.3 |  |  |  | 2  2  -  - | 14  5  -  - | 0.004  0.002  -  - | I_A_  I_B_  S_A_  S_B_ |
| **H_sp_idb_14366_c0_g1_i1^3^**  (aa2-934)  Comp106461_c0_seq3_2  **etc** | Uncharacterized; domains: EMI (aa22-97), multiple EGF-like; SSP (aa1-16), TM (aa809-831); 13.8% C, 11.7% G; pI 6.7; IDR (8.5%; aa782-813,850-900) | T1IUZ7_STRMM  (aa6-934) | 38.3 | 1.8e-116 | 3  2  -  - | 6  3  -  - | 0.001  <0.001  -  - | I_A_  I_B_  S_A_  S_B_ |
| **Comp106499_c0_seq7_3**  (aa38-378)  **H_sp_idb_42907_c0_g1_i1** | Similar to cubilin; domains: CUB (aa44-165,169-286,287-378); 11.3% S, 10.5% T; pI 5.7; shares 2 peptides with Tri_33373 | E5RYG4_TRISP  (aa1101-1437) | 27.6 | 5.5e-17 | 2  1  -  - | 4  4  -  - | <0.001  <0.001  -  - | I_A_  I_B_  S_A_  S_B_ |
| **H_sp_Tri_33373_c0_g1_i1**  (aa2-755)  Comp106499_c0_seq4_3 | Similar to cubilin; domains: 6 CUB; 11.7% S, 9.8% T, 9.6% Y; pI 4.9; shares 2 peptides with comp106499_c0_seq7_3 | G6DEC4_DANPL  (aa857-1287) | 25.0 | 4.2e-23 | 5  3  -  - | 13  6  -  - | 0.002  0.001  -  - | I_A_  I_B_  S_A_  S_B_ |
| **H_sp_idb_2214_c0_g1_i1^3^**  (aa1-430)  **Comp106543_c0_seq12_5**  **etc** | Uncharacterized; domain: HSP70_family (aa33-410); SSP/TM (aa1-22/5-22);, 11.0% L, 9.7% V; pI 7.0; if SSP: 11.0% L, 9.8% V; pI 7.2 | V4AQB2_LOTGI  Lotgidraft_159745  (aa1-430) | 68.1 | 5.0e-117 | 6  10  -  - | 30  49  -  - | 0.005  0.009  -  - | I_A_  I_B_  S_A_  S_B_ |
| **Comp106756_c0_seq2_6^3^**  (aa172-267)  **H_sp_CLC_2126_c0_g1_i1**  **etc** | Similar to cystatin-B; domain: cystatin (aa171-267); pI 8.9; similar to comp81444_c0_seq1_6; CLC_2126 | M4H503_HALDI  (aa1-98) | 70.4 | 5.2e-27 | 3  2  -  - | 15  11  -  - | 0.006  0.038  -  - | I_A_  I_B_  S_A_  S_B_ |
| **Comp106832_c0_seq1_1**  (aa263-4170)  H_sp_CLC_324_c0_g1 | Uncharacterized/similar to signal peptide, CUB and EGF-like domain-containing protein 1;domains: CTDL_fold (aa158-313), CUB (aa350-460,475-579,581-689), multiple Sushi_SCR_CCP, multiple EGF-like, IG (aa1305-1383,1387-1475,2847-2894), multiple Tyr-kin_ephrin_A/B_rcpt-like, multiple TNFR/NGFR_Cys_rich_reg, Pan_app (aa2095-2174), ConA-like (aa2591-2761), pI 7.5; IDR (1.0%; aa2924-2968) | V4BCH6_LOTGI  Lotgidraft_230171  (aa2-3687) | 37.5 | 0e0 | 5  2  -  - | 8  4  -  - | <0.001  <0.001  -  - | I_A_  I_B_  S_A_  S_B_ |
| **Comp106862_c0_seq1_3^3^**  (aa51-1458)  **Comp106862_c0_seq2_3**  H_sp_idb_22196_c0_g1_i1 | Similar to thioester-containing protein; domains: A2M_N (aa49-138), A2M_N2 (368-498), A2M (626-717), A2M_comp (845-1148), A2M_rec_bd (1239-1349); multiple TM; pI 8.3; shares many of its peptides with idb_9842; IDR (2.8%; aa1-30,624-640,1158-1175) | A0A0E4B804  _SCOSU  (aa24-1428) | 30.4 | 5.1e-132 | 30  11  -  - | 196  42  -  - | 0.007  0.001  -  - | I_A_  I_B_  S_A_  S_B_ |
| **H_sp_idb_9842_c0_g1_i1**  (aa12-799) | Similar to thioester-containing protein; domains: A2M (aa37-128), Terpenoid_cyclase/PrenylTrfase (aa258-559); A2M_receptor_binding (aa650-753); 10.4% V; pI 5.6; shares many peptides with comp106862_c0_ seq1_3; IDR (6.3%; aa1-38,471-490) | V3ZK97_LOTGI**^4^**  Lotgidraft_211452  (aa35-825) | 42.4 | 1.9e-134 | 2  1  -  - | 10  2  -  - | 0.001  <0.001  -  - | I_A_  I_B_  S_A_  S_B_ |
| **Comp106997_c0_seq1_2^3^**  (aa76-1443)  **Comp106997_c0_seq3_1** | Similar to protocadherin Fat 4; domains: multiple cadherin; 11.2% T, 9.2% V; pI 4.4; IDR (3.0%; aa1-43) | K1PTY5_CRAGI  (aa36-1403) | 39.4 | 4.7e-101 | 7  8  -  - | 30  42  -  - | 0.002  0.002  -  - | I_A_  I_B_  S_A_  S_B_ |
| **Comp107313_c0_seq1_3^3^**  (aa2231-7451)  H_sp_Tri_26792_c0_g1_i1 | Similar to protocadherin Fat 4; domains: multiple cadherin-like; multiple TM; 9.5% T; pI 6.1 | K1QB61_CRAGI  (aa1505-6530) | 38.8 | 4.3e-165 | 51  59  -  - | 190  243  -  - | 0.001  0.002  -  - | I_A_  I_B_  S_A_  S_B_ |
| **H_sp_idb_1321_c0_g1_i1^3^**  (aa10-2982)  Comp107313_c0_seq1_1  Comp107269_c0_seq9_2 | Similar to protocadherin Fat 4; domains: multiple cadherin; 11.3% T, 9.5% V; pI 4.5 | K1PTY5_CRAGI  (aa1284-4267) | 43.1 | 9.4e-215 | 26  30  -  - | 104  107  -  - | 0.003  0.004  -  - | I_A_  I_B_  S_A_  S_B_ |
| **H_sp_idb_7016_c0_g1_i1**  (aa5-474)  **Comp107084_c0_seq5_1** | Uncharacterized/similar to bactericidal permeability increasing protein; domains: Bactericidal_perm-incr_a/b (aa19-246,232-472); SSP (aa1-17); 10.9% L, 9.7% S; pI 9.7; IDR (4.6%; aa490-512) | V4C5M9_LOTGI  Lotgidraft_231708  (aa13-485) | 47.1 | 5.8e-101 | 6  9  -  - | 42  76  -  - | 0.004  0.006  -  - | I_A_  I_B_  S_A_  S_B_ |
| **H_sp_idb_20_c0_g1_i1**  (aa702-5523)  **Comp107134_c0_seq1_6**  **Comp107134_c0_seq2_6** | Uncharacterized/similar to ATP-binding cassette sub-family A member 1; domains: ABC_transporter-like (aa3720-4736,4808-5524); multiple TM in C-term third; 11.7% L; pI 4.9; IDR (3.9%; aa1201-3205,3722-3748, 3932-3947,4267-4288,4617-4664,  4783-4800,5507-5601) | A0A0L8H2M5  _OCTBM  (aa130-4990) | 26.4 | 1.7e-68 | 13  11  -  - | 24  29  -  - | <0.001  <0.001  -  - | I_A_  I_B_  S_A_  S_B_ |
| **Comp107245_c0_seq1_5^3^**  (aa93-3146)  **Comp107245_c0_seq2_5**  **H_sp_Tri_34963_c0_g1_i1** | Uncharacterized/reeler_Egf_Cub_1; domains: multiple EGF_like, multiple Thyroglobulin_1, mult. WAP, Antistasin_like (aa1832-1857) Kunitz_BPTI (aa2303-2358,2468-2518,2781-2831), SEA (aa966-1071); TM (aa3267-3289); pI 7.0; IDR (6.1%; aa1920-1925,1962-2000,2238-2242,2643-2668,2721-2783,2842-2854,3066-3079,3294-3335) | W4ZEF6_STRPU  (aa1945-5191) | 23.1 | 6.2e-30 | 16  11  -  - | 43  26  -  - | 0.001  0.001  -  - | I_A_  I_B_  S_A_  S_B_ |
| **Comp107325_c0_seq2_5**  (aa86-7547)  **Comp107325_c0_seq1_5** | Uncharacterized/similar to protein jagged-2; shares most peptides with idb_1052; domains: Vitellinogen_b-sht_N (62-256), peptidase_M23 (aa1083-1184), SAP (aa1260-1313,1547-1627), multiple IG, EGF_like, multiple TM; pI 5.3; multiple IDR (5.1%) | V3ZES3_LOTGI**^4^**  Lotgidraft_236952  (aa6-7471) | 49.9 | 0e0 | 12  12  -  - | 50  62  -  - | <0.001  <0.001  -  - | I_A_  I_B_  S_A_  S_B_ |
| **Comp90611_c0_seq1_2**  (aa2-1215) | Uncharacterized; domain: PAN/apple (aa1002-1083); pI 5.6; shares most peptides with idb_1052; IDR (3.0% (aa803-839) | V3ZES3_LOTGI**^4^**  Lotgidraft_236952  (aa3189-4403) | 51.3 | 0.0 | 1  1  -  - | 2  3  -  - | <0.001  0.001  -  - | I_A_  I_B_  S_A_  S_B_ |
| **H_sp_idb_1052_c0_g1_i1**  (aa3-6137)  **Comp90611_c0_seq1_4** | Uncharacterized; domains: SAP (aa190-224), PAN_app (aa2862-2942), FN3 (3510-3707, cadherin (5632-5736, EGF-like (5878-5919); pI 4.9; mult IDR (4.1%); shares most peptides with comp107325_c0_ seq2_5 and/or comp90611_c0_seq1_2 | V3ZES3_LOTGI**^4^**  Lotgidraft_236952  (aa1328-7471) | 49.9 | 0e0 | 47  57  -  - | 124  207  -  - | 0.002  0.003  -  - | I_A_  I_B_  S_A_  S_B_ |
| **H_sp_Tri_7956_c0_g1_i1**  (aa3-6466)  Comp106992_c0_seq1_5 | Uncharacterized; domains: FN3 (aa4299-4389,5486-5585), EGF_like (aa6198-6239,6241-6275); pI 5.1; mult IDR (5.0%); shares several peptides with comp107325_c0_seq2_5 | V3ZES3_LOTGI**^4^**  Lotgidraft_236952  (aa1014-7471) | 48.7 | 0e0 | 4  4  -  - | 14  7  -  - | <0.001  <0.001  -  - | I_A_  I_B_  S_A_  S_B_ |
| **Comp85993_c0_seq3_1^3^**  (aa1-85)  **H_sp_Tri_109898_c0_g1_i1**  **etc** | UBIQP_0/ubiquitin/polyubiquitin-B; domains: Ubiquitin (aa1-27,30-89); 12.2% L; pI 5.8; IDR (54.4%; aa1-16,58-90) | A0A0C9RN62  _9HYME  (aa5-93) | 98.9 | 1.9e-33 | 4  4  2  2 | 68  53  8  14 | 0.032  0.088  0.014  0.154 | I_A_  I_B_  S_A_  S_B_ |
| **Comp109254_c0_seq1_3^3^**  (aa10-348) | Glyceraldehyde-3-phosphate dehydrogenase; domains: GlycerAld_3-P_DH_NAD(P)-bd (aa17-165), GlycerAld_3-P_DH_cat (aa170-327); pI 8.7; IDR (15.1%; aa350-410); see also idb_33826¸ very similar but no shared peptides! | A0A0A0MQF6_  MOUSE  (aa21-359) | 100.0 | 5.8e-143 | 2  3  -  3 | 12  10  -  10 | 0.002  0.003  -  0.033 | I_A_  I_B_  S_A_  S_B_ |
| **H_sp_CLC_597_c0_g1_i1**  **Comp109446_c0_seq1_5** | Uncharacterized; SSP (aa1-19); 14.1% P, 13.1% Y; pI 9.9; IDR (10.1%; aa108-117) |  |  |  | 2  3  -  - | 17  15  -  - | 0.015  0.015  -  - | I_A_  I_B_  S_A_  S_B_ |
| **H_sp_idb_10969_c0_g1_i1**  (aa1-117)  **H_sp_idb_10968_c0_g1_i1^3^**  **Comp112534_c0_seq1_2** | Similar to tyramine beta-hydroxylase/temptin; SSP (aa1-19); 11.2% G; pI 9.0; IDR (26.5%; aa102-127) | K1RV04_CRAGI  (aa1-117) | 49.6 | 8.3e-22 | 5  3  -  - | 10  11  -  - | 0.002  0.002  -  - | I_A_  I_B_  S_A_  S_B_ |
| **Comp114698_c0_seq1_3^3^**  (aa1-121) | Histone 2B; 9.5% A, 13.5% K, 10.1% S; pI 10.5; domain: Histone_H2A/H2B/H3 (aa8-121); IDR (46.0%; aa1-38,118-146) | H2B_PATGR  (aa2-122) | 97.5 | 1.3e-44 | 3  3  -  - | 17  16  -  - | 0.004  <0.001  -  - | I_A_  I_B_  S_A_  S_B_ |
| **Comp128817_c0_seq1_3^3^**  **H_sp_idb_42198_c0_g1_i1** | Uncharacterized; domain: hirudin_antistatin (aa219-257); 12% P, 9.3% S; pI 9.6; IDR (56.6%; aa1-84,190-241) |  |  |  | 18  13  2  - | 367  259  4  - | 0.484  0.303  0.006  - | I_A_  I_B_  S_A_  S_B_ |
| **Comp133740_c0_seq1_1^3^**  (aa1-478)  H_sp_CLC_1079_c0_g1_i1  A7L6B1_HALAI | Catenin beta-1; domain: ARM-type_fold (aa1-480); 14.0% L; pI 9.1 | A0A087QH36_  APTFO  (aa129-606) | 100.0 | 2.2e-209 | 3  3  -  3 | 20  12  -  9 | 0.001  0.001  -  0.004 | I_A_  I_B_  S_A_  S_B_ |
| **Comp135434_c0_seq1_4^3^**  (aa1-105) | Histone H4; domain: TAF_TATA-bd (aa1-104); 12.6% R, 15.3% G, 10.8% K; pI 11.2; IDR (26.1%; aa1-29) | A0A023FTG4_  9ACAR  (aa5-109) | 99.0 | 1.4e-39 | 6  5  -  - | 102  98  -  - | 0.122  0.161  -  - | I_A_  I_B_  S_A_  S_B_ |
| **Comp146963_c0_seq1_4**  (aa3-498) | Similar to mucin-19; domains: VWD (aa14-217), TIL (aa278-333); pI 4.8 | D7RV99_MOUSE  (aa90-589) | 27.3 | 7.7e-25 | 9  6  -  - | 18  18  -  - | 0.003  0.003  -  - | I_A_  I_B_  S_A_  S_B_ |
| **H_sp_Tri_808_c0_g1_i1^3^**  (aa18-132)  **Comp157484_c0_seq1_1** | Similar to zinc metalloproteinase nas-39; domain: CUB (aa18-134); SSP (aa1-19); 11.0% I; pI 4.7 | NAS39_CAEEL  (aa550-646) | 35.0 | 1.8e-5 | 2  2  -  - | 5  3  -  - | 0.001  0.001  -  - | I_A_  I_B_  S_A_  S_B_ |
| **Comp191803_c0_seq1_2** | Uncharacterized; 16.9% T, pI 4.3; IDR (6.9%; aa150-160) |  |  |  | 4  6  2  - | 16  15  2  - | 0.004  0.010  0.005  - | I_A_  I_B_  S_A_  S_B_ |
| **Comp22563_c0_seq1_3**  (aa8-240) | Uncharacterized; 11.5% S, 15.9% T; pI 6.0; domains: chitin-bd_II (aa9-76,101-268,184-241); IDR (21.5%; aa67-95,168-178,270-312); shares 3 peptides with idb_54309 and 1 with idb_57746 | V4A6J8_LOTGI  Lotgidraft_163871  (aa84-309) | 34.5 | 1.6e-13 | 1  4  -  1 | 2  13  -  1 | 0.001  0.008  -  0.003 | I_A_  I_B_  S_A_  S_B_ |
| **H_sp_idb_54309_c0_g1_i1**  (aa36-368) | Uncharacterized; chitin-bd_II (aa22-90,134-201,230-297,313-370); SSP (aa1-19); 13.6% S, 22.0% T; pI 4.6; IDR (19.4%; aa89-138,201-217) | A0A0Q9WIQ0  _DROVI  (aa253-597) | 29.3 | 1.4e-12 | 5  1  3  3 | 23  1  9  8 | 0.013  0.001  0.029  0.016 | I_A_  I_B_  S_A_  S_B_ |
| **H_sp_idb_57746_c0_g1_i1**  (aa38-223) | Similar to mucin-like protein; domains: chitin-bd_II (aa24-92,136-203); SSP (aa1-21); 16.6% S, 23.4% T; pI 4.8; IDR (10.6%; aa203-225) | M1F021_HOLOL  (aa66-256) | 39.1 | 8.8e-12 | 1  1  -  1 | 1  2  -  2 | 0.002  0.006  -  0.015 | I_A_  I_B_  S_A_  S_B_ |
| **Comp23247_c0_seq1_2^3^**  (aa46-424) | Fructose-bisphosphate aldolase; domain: Aldolase_TIM (aa64-407); 10.7% A; pI 8.7; IDR (16.3%; aa1-79) | A6ZI44_MOUSE  (aa40-418) | 98.4 | 8.9e-162 | 2  3  -  - | 4  8  -  - | <0.001  0.002  -  - | I_A_  I_B_  S_A_  S_B_ |
| **Comp23692_c0_seq1_3^3^**  (aa1-250)  Q45Y86_HALRU | Triosephosphate isomerase; Aldolase_TIM (aa2-250); pI 7.9 | TPIS_MOUSE  (aa50-299) | 100.0 | 4.1e-107 | -  5  -  2 | -  7  -  3 | -  0.001  -  0.002 | I_A_  I_B_  S_A_  S_B_ |
| **H_sp_Tri_27896_c0_g1_i1**  (aa23-271)  **Comp90883_c0_seq1_3**  Q45Y86_HALRU**^3^** | Triosephosphate isomerase; domain: Aldolase_TIM (aa23-271); 10.7% A, 11.0% V; pI 6.4; IDR (10.7%; aa1-29) | V4AUY5_LOTGI**^4^**  Lotgidraft_238326  (aa1-250) | 78.4 | 1.0e-83 | 4  4  -  - | 9  17  -  - | 0.003  0.005  -  - | I_A_  I_B_  S_A_  S_B_ |
| **Comp241021_c0_seq1_1^3^**  (aa1-80) | Aspartate and glycine-rich protein (Fragment); 22.5% A, 13.8% R, 28.7% G, 22.5% S; pI 11.8; IDP; shares several peptides with CLC_5 | DGRP_HALAI  (aa211-282) | 81.2 | 5.0e-8 | 1  2  -  - | 8  14  -  - | 0.005  0.005  -  - | I_A_  I_B_  S_A_  S_B_ |
| **H_sp_CLC_5_c0_g1_i1**  (aa1-177)  H_sp_Tri_57798_c0_g1_i1**^3^** | Similar to MSI60-related protein/ similar to hasinina_P008C13_381**^4^**; 20.9% A, 30.8% G, 16.5% S; pI 4.6; IDR (47.8%; aa96-182); shares several peptides with Comp241021_c0_ seq1_1; repeats (Fig. S2Zb) | G9MD31_PINFU  (aa192-371) | 45.9 | 1.5e-7 | 6  4  -  2 | 74  57  -  9 | 1.601  1.548  -  0.833 | I_A_  I_B_  S_A_  S_B_ |
| **H_sp_Tri_78397_c0_g1_i1**  (aa37-315)  **Comp24679_c0_seq1_5** | Uncharacterized/similar to periostin; domains: FAS1 (aa35-173,174-313); SSP (aa1-17); 12.3% L, 9.6% T, 11.6% V; pI 5.6 | V4CG37_LOTGI  Lotgidraft_200505  (aa38-309) | 55.2 | 1.6e-52 | 9  9  -  - | 56  69  -  - | 0.026  0.034  -  - | I_A_  I_B_  S_A_  S_B_ |
| **H_sp_Tri_38314_c0_g1_i1^3^**  (aa5-164)  Comp24992_c0_seq1_5  Comp24992_c0_seq1_1 | Similar to lustrin A (fragment); domains: WAP (aa57-103,104-158); SSP (aa1-19); 14.0% C, 9.1% G, 14.6% P; pI 8.7 | J7QAX0_PATVU  (aa23-192) | 31.5 | 1.5e-9 | 2  3  -  - | 20  26  -  - | 0.006  0.021  -  - | I_A_  I_B_  S_A_  S_B_ |
| **H_sp_idb_45304_c0_g1_i1^3^**  (aa27-313) | Similar to lustrin A; domains: [WAP/ Cys_repeat_1](http://www.ebi.ac.uk/interpro/entry/IPR008197)  (aa67-110,158-204,253-298,353-406); SSP (aa1-19); 13.3% C, 9.9% P, 9.9% T; pI 7.4; IDR (1.5%; aa355-360) | J7QAX0_PATVU  (aa143-438) | 30.0 | 1.2e-13 | 5  6  -  - | 45  49  -  - | 0.010  0.007  -  - | I_A_  I_B_  S_A_  S_B_ |
| **Comp328175_c0_seq1_1**  (aa1-71) | Similar to glutamate carboxypeptidase 2/ N-acetylated-alpha-linked acidic dipeptidase 2 (Fragment); IDR (9.3%; aa68-75); shares 5 peptides with idb_19016; domain: transferrin_ receptor-like_dimerisation (aa2-74) | M7B6A4_CHEMI  (aa595-665) | 50.7 | 3.8e-10 | 1  -  -  - | 9  -  -  - | <0.001  -  -  - | I_A_  I_B_  S_A_  S_B_ |
| **Comp338365_c0_seq1_4**  (aa5-143) | Uncharacterized/similar to glutamate carboxypeptidase 2; domain: protease_associated (PA; aa24-107); IDR (12.5%; aa1-12,139-144); shares 1 peptide with idb_19016 | V4B659_LOTGI  Lotgidraft_185795  (aa176-315) | 51.4 | 8.7e-27 | 2  -  -  - | 8  -  -  - | 0.004  -  -  - | I_A_  I_B_  S_A_  S_B_ |
| **Comp42014_c0_seq1_4**  (aa10-249) | Uncharacterized/similar to N-acetylated-alpha-linked acidic dipeptidase 2; domain: peptidase_M28 (74-249); IDR (4.4%; aa1-11); shares 4 peptides with idb_19016 | WAZL64_STRPU  (aa278-518) |  |  | 2  -  -  - | 10  -  -  - | 0.002  -  -  - | I_A_  I_B_  S_A_  S_B_ |
| **H_sp_idb_19016_c0_g1_i1**  (aa2-505)  Comp42014_c1_Seq1_2 | Uncharacterized; domains: peptidase_M28 (aa123-326), TFR-like_dimer (367-505); pI 7.7; IDR (1.4%; aa44-50); shares several peptides with comp328175_c0_ seq1_1, comp42014_c0_seq1_4, comp338365_c0_seq1_4 | W4ZL64_STRPU  (aa219-726) | 46.3 | 1.1e-95 | 15  7  -  - | 251  83  -  - | 0.054  0.013  -  - | I_A_  I_B_  S_A_  S_B_ |
| **Comp40287_c0_seq1_1** | Uncharacterized; SSP (aa1-14); 11.2% C, 10.1% S; pI 6.7; IDR (4.5%; aa100-103) |  |  |  | 4  4  2  - | 43  23  9  - | 0.048  0.026  0.100  - | I_A_  I_B_  S_A_  S_B_ |
| **Comp44977_c0_seq1_2**  (aa5-298) | Uncharacterized; domain: VWD (aa62-15); 11.1%G; pI 5.3 | A0A0B7A304  _9EUPU  (aa81-383) | 27.3 | 5.1e-17 | 4  6  -  - | 15  23  -  - | 0.004  0.005  -  - | I_A_  I_B_  S_A_  S_B_ |
| **H_sp_Tri_23502_c0_g1_i1^3^**  **Comp46614_c0_seq1_4** | Uncharacterized; 12.8% S, 22.6% T, pI 4.1; IDP |  |  |  | 4  4  2  3 | 29  32  8  8 | 0.022  0.031  0.043  0.054 | I_A_  I_B_  S_A_  S_B_ |
| **Comp46746_c0_seq1_2**  (aa14-447) | Similar to SCO-spondin; domains: VWD (aa1-116), Unchr _Cys-rich (aa102-171), TIL (aa175-235), multiple LDLR; 9.5% C, 9.3% T; pI 4.5; IDR (14.1%; aa36-54,78-88,424-429,463-478,510-520,563-681) | F1QSL8_DANRE  (aa1115-1550) | 38.5 | 1.3e-55 | 4  4  -  - | 11  11  -  - | 0.002  0.002  -  - | I_A_  I_B_  S_A_  S_B_ |
| **Comp48128_c0_seq1_4^3^**  (aa19-541)  Q45Y88_HALRU  H_sp_idb_34797_c0_g1_i1 | Elongation factor 1-alpha; domains: TF_GTP-bd (aa21-260), EFTu-like_2 (aa278-343), Transl_elong_EFTu/EF1A_C (aa354-459); 9.9% K; pI 9.4; IDR (1.2%; aa1-7) | W5PHA3_SHEEP  (aa1-525) | 97.7 | 3.0e-131 | -  2  -  3 | -  7  -  7 | -  0.001  -  0.005 | I_A_  I_B_  S_A_  S_B_ |
| **H_sp_idb_34797_c0_g1_i1**  (aa1-440)  **Comp86258_c0_seq3_4**  **Comp86258_c0_seq2_4** | Elongation factor 1-alpha; domains: P-loop_NTPase (aa1-243, EFTu-like_2 (aa242-307), Transl_elong_EF1A/ Init_IF2_C (aa319-423); 10.9% K; pI 9.1; IDR (4.3%; aa422-440); shares several peptides with comp48128_c0_seq1_4 | D1H0L8_HALTU  (aa19-458) | 95.5 | 3.3e-128 | 4  6  -  - | 33  46  -  - | 0.004  0.005  -  - | I_A_  I_B_  S_A_  S_B_ |
| **Comp49086_c0_seq1_6**  H_sp_idb_68755_c0_g1_i1 | Uncharacterized; 10.1%S, 27.1% T, pI 4.3; IDR (45.0%; aa1-72,193-218) |  |  |  | 6  7  -  - | 21  25  -  - | 0.006  0.001  -  - | I_A_  I_B_  S_A_  S_B_ |
| **Comp49273_c0_seq1_2^3^**  H_sp_Tri_105572_c0_g1_i1 | Uncharacterized; 10.3% N, 14.0% Q, 10.3% S, pI 8.7; shares several peptides with idb_46434; SSP (aa1-27); IDR (61.0%; aa42-220); tandem repeats in aa88-96 ([NAYGT]_2_) |  |  |  | 9  7  6  2 | 265  214  32  15 | 1.026  0.487  0.450  0.086 | I_A_  I_B_  S_A_  S_B_ |
| **H_sp_idb_46434_c0_g1_i1^3^** | Uncharacterized; SSP (aa1-22); 11.7% N, 15.2% Q, 10.3% G, 10.8% S; pI 9.3; IDR (63.7%; aa52-194); shares several peptides with comp49273_c0_seq1_2 |  |  |  | 2  1  -  - | 18  20  -  - | 0.005  <0.001  -  - | I_A_  I_B_  S_A_  S_B_ |
| **Comp49884_c0_seq1_4**  (aa1-78) | Similar to ependymin-related protein; domain: ependymin (aa1-78); pI 9.4 | EPDR1_HALAI**^4^**  (aa118-198) | 45.7 | 2.7e-10 | 2  2  -  - | 11  3  -  - | 0.003  0.002  -  - | I_A_  I_B_  S_A_  S_B_ |
| **H_sp_CLC_1047_c0_g1_i1**  (aa11-133)  **Comp51373_c0_seq1_3** | Similar to BPTI/Kunitz domain-containing protein; domains: Kunitz_BPTI (aa21-79,81-134); SSP (aa1-21); 12.3% C, 12.3% G; pI 8.7 | KCP_HALAI**^4^**  (aa1-120) | 53.7 | 4.9e-27 | 4  5  -  - | 120  90  -  - | 0.129  0.218  -  - | I_A_  I_B_  S_A_  S_B_ |
| **Comp51700_c0_seq3_3^3^**  (aa17-214)  H_sp_idb_57414_c0_g1_i1  B1N694_HALDI | Peroxiredoxin-1;domain: Thioredoxin-like_fold (aa7-201); pI 6.4; IDR (6.5%; aa201-214) | PRDX1_MOUSE  (aa1-198) | 100.0 | 2.4e-88 | 3  2  -  2 | 14  13  -  4 | 0.002  0.002  -  0.012 | I_A_  I_B_  S_A_  S_B_ |
| **H_sp_CLC_18921_c0_g1_i1^3^**  (aa9-603)  **Comp52213_c0_seq1_3** | Uncharacterized; SSP (aa1-20); pI 5.6 | V3ZWU1_LOTGI Lotgidraft_154902  (aa9-600) | 32.7 | 1.2e-81 | 9  10  -  - | 74  96  -  - | 0.011  0.015  -  - | I_A_  I_B_  S_A_  S_B_ |
| **Comp54951_c0_seq1_3**  (aa1-211)  B3TK83_HALDV | Actin; pI 9.0; shares 2 peptides with idb_35737 | A9LGM3_9STRA  (aa84-292) | 95.3 | 2.0e-84 | 1  1  -  - | 33  31  -  - | 0.057  0.068  -  - | I_A_  I_B_  S_A_  S_B_ |
| **H_sp_idb_35737_c0_g1_i1**  (aa1-171) | Actin (A3); pI 4.7; shares 2 peptides with Comp54951_c0_seq1_3 | Q5BQE3_9VEST  (aa120-290) | 99.4 | 4.5e-68 | 2  2  -  - | 23  31  -  - | 0.008  0.027  -  - | I_A_  I_B_  S_A_  S_B_ |
| **Comp59223_c0_seq1_2^3^**  (aa17-123)  **H_sp_Tri_28544_c0_g1_i1** | Similar to putative ferric-chelate reductase 1-like protein; shares most peptides with Tri_28544; 10.3% S; pI 9.5; IDR (35.0%; aa116-176) | K1RDF5_CRAGI  (aa518-626) | 45.5 | 2.0e-10 | 11  10  -  - | 72  35  -  - | 0.025  0.015  -  - | I_A_  I_B_  S_A_  S_B_ |
| **H_sp_Tri_105613_c0_g1_i1^3^**  (aa1-1442**)**  Comp32926_c1_seq1_1  Comp508308_c0_seq1_2  Comp32926_c0_seq1_5  Comp59338_c0_seq1_5  Comp278816_c0_seq1_1 | Similar to thioester-containing protein; domains: A2M_N (aa115-199), A2M_N_2 (aa449-582), Macroglobulin_a2 (721-811), Terpenoid_cyclase/PrenylTrfase (aa939-1241), A-macroglobulin_rcpt-bd (aa1309-1424); SSP (aa1-16); pI 8.3; IDR (1.3%; aa693-711) | D5FT50_9BIVA  (aa1-1450) | 57.6 | 0e0 | 36  37  -  - | 362  313  -  - | 0.027  0.028  -  - | I_A_  I_B_  S_A_  S_B_ |
| **H_sp_idb_38227_c0_g1_i1**  **Comp63434_c0_seq1_2** | Uncharacterized; TM (aa47-69,166-188); 12.1% A, 10.0% S; pI 8.7; IDR (4.2%; aa1-9) |  |  |  | 4  2  -  - | 12  3  -  - | 0.016  0.003  -  - | I_A_  I_B_  S_A_  S_B_ |
| **H_sp_idb_84384_c0_g1_i1**  (aa38-145)  **Comp64150_c0_seq2_1** | Similar to Kunitz-like protease inhibitor; domains: Kunitz_BPTI (aa35-91,92-146); SSP (aa1-28); pI 8.0; 10.0% C | J7HZ94_POMCA  (aa73-178) | 52.8 | 6.0e-24 | 5  2  -  - | 12  7  -  - | 0.002  0.001  -  - | I_A_  I_B_  S_A_  S_B_ |
| **H_sp_Tri_111928_c0_g1_i1^3^**  (aa36-274)  **Comp64272_c0_seq1_3** | Uncharacterized; SSP (aa1-24); pI 9.1 | K1QJ54_CRAGI**^4^**  (aa303-536) | 23.5 | 3.8e-5 | 24  19  3  - | 726  691  5  - | 3.841  4.425  0.038  - | I_A_  I_B_  S_A_  S_B_ |
| **Comp64297_c0_seq2_6**  **Comp64297_c0_seq1_6** | Uncharacterized; SSP (aa1-26); 14.7% G, 18.8% P (aa80-102 multiple GGP-repeats); pI 9.0; IDP |  |  |  | 6  3  -  - | 16  5  -  - | 0.009  0.005  -  - | I_A_  I_B_  S_A_  S_B_ |
| **H_sp_Tri_28744_c0_g1_i1^3^**  Comp64512_c0_seq1_1 | Uncharacterized; domain: CAP (aa209-376); SSP (aa1-25); 11.1% G; pI 9.7; IDR (6.5%; aa147-170) |  |  |  | 5  5  -  - | 57  41  -  - | 0.011  0.008  -  - | I_A_  I_B_  S_A_  S_B_ |
| **Comp68174_c0_seq1_5^3^**  (aa7-155) | Calmodulin; domain: EF-hand-dom_pair (aa4-154); 10.6% D, 13.0% E; pI 4.2; shares peptides with Tri_60708 | CALM_SACJA  (aa1-149) | 99.3 | 2.2e-48 | -  2  -  - | -  5  -  - | -  0.002  -  - | I_A_  I_B_  S_A_  S_B_ |
| **H_sp_Tri_60708_c0_g1_i1^3^**  (aa1-149)  **B3SND3_HALDV**  **Comp31935_c0_seq1_4**  **etc** | Calmodulin; domain: EF-hand-dom_pair (2-149); 11.4% D, 14.1% E; pI 4.1; IDR (46.9%; aa1-59,119-128); shares 1 peptide with comp68174_c0_seq1_5/comp31935_c0_seq1_4; N-term acA_2_ | B3SND3_HALDV  (aa1-149) | 100.0 | 1.9e-49 | -  1  -  2 | -  3  -  8 | -  0.001  -  0.019 | I_A_  I_B_  S_A_  S_B_ |
| **Comp68339_c0_seq2_2** | Uncharacterized; 11.1% C, 9.9% S |  |  |  | 2  2  -  - | 10  6  -  - | 0.008  0.003  -  - | I_A_  I_B_  S_A_  S_B_ |
| **Comp70759_c0_seq1_2^3^**  (aa33-116) | Similar to perlustrin; domain: Growth_fac_rcpt/IGFBP (aa32-98); SSP (aa1-32); 10.3% C; pI 8.0; IDR (28.2%; aa116-149) | PLS_HALLA**^4^**  (aa1-84) | 70.2 | 1.2e-24 | 8  7  5  3 | 283  264  34  17 | 6.042  8.709  1.175  0.525 | I_A_  I_B_  S_A_  S_B_ |
| **H_sp_idb_14514_c0_g1_i1**  (aa11-211)  **H_sp_Tri_65602_c0_g1_i1**  **Comp71326_c0_seq1_2** | Uncharacterized; SSP (aa1-26); pI 4.3; IDR (11.6%; aa181-204) | A0A0B6ZGX7  _9EUPU  (aa4-197) | 35.3 | 4.7e-15 | 2  3  -  - | 14  21  -  - | 0.010  0.009  -  - | I_A_  I_B_  S_A_  S_B_ |
| **Comp73608_c1_seq1_6^3^**  (aa47-180) | Similar to nattectin; domain: C-type_lectin (aa27-181); TM (aa20-37); 10.2% T; pI 9.6; IDR (17.7%; aa1-11,189-215) | G1FKF5_EPIBR  (aa32-161) | 35.1 | 2.1e-15 | 3  2  -  - | 7  6  -  - | 0.001  0.002  -  - | I_A_  I_B_  S_A_  S_B_ |
| **H_sp_Tri_26397_c0_g1_i1**  (aa26-443)  **Comp78504_c0_seq3_1**  **Comp78504_c0_seq2_2** | Eukaryotic initiation factor 4A-II; domain: P-loop_NTPase (aa63-440); pI 5.3; IDR (14.5%; aa1-64) | A0A087TAB2  _9ARAC  (aa7-420) | 79.9 | 3.6e-123 | 3  2  -  - | 10  6  -  - | 0.001  0.001  -  - | I_A_  I_B_  S_A_  S_B_ |
| **Comp79549_c0_seq1_2^3^**  (aa6-663) | Heat shock cognate 71 kDa protein; domains: HSP70_peptide-bd (aa407-538), HSP70_C (aa539-637); pI 6.5; IDR (29.8%; aa1-22,510-693); shares several peptides with idb_22145 and idb_28957 | M0R8M9_RAT  (aa1-658) | 97.8 | 0e0 | -  8  -  4 | -  27  -  5 | -  0.003  -  0.002 | I_A_  I_B_  S_A_  S_B_ |
| **Comp79626_c0_seq1_4^3^**  (aa30-540) | Similar to chitinase-3; domains: chitinase_II (aa46-420), chitin-bd_II (aa476-534); TM (aa20-42); pI 8.7; IDR (14.1%; aa1-10,426-477,537-552); shares most peptides with idb_43266 | J7FIC1_HYRCU  (aa8-515) | 40.4 | 3.9e-70 | 24  1  -  - | 325  22  -  - | 0.114  0.002  -  - | I_A_  I_B_  S_A_  S_B_ |
| **H_sp_idb_43266_c0_g1_i1^3^**  (aa16-520) | Similar to chitinase-3; domains: Chitinase_II (aa33-407); chitin-bd_II (aa463-519); SSP (aa1-28); pI 8.1; IDR (10.0%; aa414-465); shares most peptides with comp79626_c0_seq1_4 | J7FIC1_HYRCU  (aa8-509) | 40.3 | 2.8e-71 | 4  16  -  - | 48  199  -  - | 0.014  0.044  -  - | I_A_  I_B_  S_A_  S_B_ |
| **Comp80185_c0_seq1_5^3^**  (aa34-467) | Alpha-enolase; domains: Enolase_N (aa36-173), Enolase_C (aa176-464); pI 8.4; IDR (10.1%; aa1-4,462-505,563-571) | ENOA_MOUSE  (aa1-434) | 100.0 | 2.9e-176 | 5  6  -  4 | 14  27  -  19 | <0.001  0.002  -  0.014 | I_A_  I_B_  S_A_  S_B_ |
| **H_sp_idb_21028_c0_g1_i1**  (aa64-783)  **H_sp_Tri_128703_c0_g1_i1**  **Comp81189_c0_seq1_4** | Similar to amine oxidase; domain: copper_amine_oxidase (aa154-787); TM (aa69-91; pI 6.3; IDR (8.4%; aa103-153,390-406) | V3ZM09_LOTGI  Lotgidraft_120790  (aa1-686) | 51.9 | 7.5e-173 | 3  7  -  - | 8  36  -  - | 0.001  0.004  -  - | I_A_  I_B_  S_A_  S_B_ |
| **H_sp_idb_33584_c0_g1_i1**  (aa2-140)  **Comp81221_c0_seq1_1** | Uncharacterized/similar to cyclic AMP-regulated protein; domain: ADF-H/Gelsolin-like (aa2-140); 9.9% D; pI 4.8; IDR (7.8%; aa131-141) | C4A035_BRAFL  (aa3-141) | 58.3 | 5.1e-33 | 2  3  -  - | 3  9  -  - | 0.002  0.004  -  - | I_A_  I_B_  S_A_  S_B_ |
| **Comp81444_c0_seq1_6^3^**  (aa1-44) | Similar to cystatin B 4; 9.5% K, 9.5% S; pI 9.7; very similar to comp106756_c0_seq2_6 | M4H503_HALDI  (aa33-77) | 58.1 | 2.1e-7 | 2  2  -  - | 21  20  -  - | 0.018  0.044  -  - | I_A_  I_B_  S_A_  S_B_ |
| **H_sp_idb_24554_c0_g1_i1**  (aa1-127)  **Comp82066_c1_seq1_4** | Similar to heme-binding protein 2; domain: SOUL_haem-bd_fam/ Reg_factor_effector (aa39-216); SSP (aa1-18); 11.1% S; pI 8.9; IDR (7.8%; aa26-42) | K1PGA1_CRAGI  (aa1-214) | 55.9 | 7.5e-49 | 2  2  -  - | 7  6  -  - | 0.015  0.002  -  - | I_A_  I_B_  S_A_  S_B_ |
| **H_sp_idb_12724_c0_g1_i1^3^**  (aa15-307)  **Comp82638_c0_seq1_6** | Uncharacterized; SSP (aa1-30); pI 7.1 | V3ZWU6_LOTGI**^4^**  Lotgidraft_154590  (aa4-293) | 41.2 | 6.3e-50 | 6  6  -  - | 81  63  -  - | 0.028  0.021  -  - | I_A_  I_B_  S_A_  S_B_ |
| **H_sp_idb_54086_c0_g1_i1**  (aa1-133)  Comp82828_c0_seq1_3  **etc** | Transgelin; domain: CH (14-146); 13.2% L, 10.5% K; pI 9.5; IDR (10.5; aa1-16) | B6RB35_HALDI  (aa1-133) | 82.0 | 1.2e-41 | 2  3  -  - | 3  4  -  - | 0.001  0.002  -  - | I_A_  I_B_  S_A_  S_B_ |
| **Comp83799_c2_seq1_1^3^**  (aa53-269)  **H_sp_idb_22374_c0_g1_i1** | Uncharacterized; domain: EF_hand (aa91-199); 14.3% Q, 9.6% P; pI 5.9; C-term: QQPPP- and similar repeats; IDR (67.1%; aa1-13,57-102,128-175,180-219) | V3ZTJ0_LOTGI  Lotgidraft_234943  (aa23-233) | 48.9 | 7.3e-17 | 2  2  -  - | 26  23  -  - | 0.011  0.008  -  - | I_A_  I_B_  S_A_  S_B_ |
| **H_sp_Tri_9274_c0_g1_i1**  (aa12-292)  **Comp84429_c0_seq1_1** | Uncharacterized; domain: DUF4735 (aa10-288); 10.2% E, 10.6% L; pI 5.6; IDR (3.1%; aa83-91) | V4C1F5_LOTGI  Lotgidraft_160424  (aa64-339) | 47.4 | 1.1e-53 | 3  2  -  - | 5  2  -  - | <0.001  <0.001  -  - | I_A_  I_B_  S_A_  S_B_ |
| **Comp84928_c0_seq1_4^3^**  (aa41-166) | Similar to BPTI/Kunitz domain-containing protein; domains: Kunitz_BPTI (aa45-105,108-162); SSP? (aa1-47); shares some peptides with KCP_HALAI | KCP_HALAI**^4^**  (aa1-126) | 83.3 | 9.1e-53 | 9  8  -  - | 144  104  -  - | 0.724  0.598  -  - | I_A_  I_B_  S_A_  S_B_ |
| **H_sp_CLC_77_c0_g1_i1^3^**  (aa13-137) | Similar to BPTI/Kunitz domain-containing protein (fragment); domain: Kunitz_BPTI (aa16-78,80-133); SSP (aa1-20); 11.0% R, 11.0% C, 10.2% G; pI 9.2 | KCP_HALAI**^4^**  (aa1-125) | 83.2 | 5.2e-51 | 8  6  -  - | 198  19  -  - | 0.506  0.093  -  - | I_A_  I_B_  S_A_  S_B_ |
| **H_sp_CLC_148_c0_g1_i1^3^**  (aa13-134)  Comp101644_c0_seq1_4 | Similar to BPTI/Kunitz domain-containing protein (fragment); domain: Kunitz_BPTI (aa16-78,80-135); SSP (aa1-20); 10.2% R, 11.0% C, 11.0% G, 10.1% L; pI 8.8; IDR (14.4%; aa37-54) | KCP_HALAI**^4^**  (aa1-122) | 82.8 | 2.8e-50 | 6  6  -  2 | 130  128  -  2 | 0.282  0.335  -  0.002 | I_A_  I_B_  S_A_  S_B_ |
| **H_sp_Tri_41297_c0_g1_i1**  (aa33-464)  **Comp85374_c0_seq1_2** | Enolase; domains: Enolase_N-like (aa35-166), Enolase_C (aa174-464); pI 7.1 | ENO_DORPE  (aa1-432) | 82.2 | 9.4e-150 | 2  4  -  - | 16  27  -  - | 0.001  0.005  -  - | I_A_  I_B_  S_A_  S_B_ |
| **H_sp_idb_14094_c0_g1_i1**  **H_sp_idb_104082_c0_g1_i1**  **H_sp_idb_14091_c0_g1_i1**  **Comp85564_c0_seq1_5** | Uncharacterized; SSP (aa1-20); 13.6% A, 14.2% L, 13.0% P; pI 5.3; IDR (41.4%; aa21-87; ~58%) |  |  |  | 2  4  -  - | 59  99  -  - | 0.134  0.266  -  - | I_A_  I_B_  S_A_  S_B_ |
| **H_sp_CLC_1642_c0_g1_i1^3^**  (aa31-201)  **Comp85674_c0_seq1_1**  **Comp85674_c0_seq2_1** | Similar to aragonite protein AP24; TM (aa36-55); pI 7.0; IDR (12.4%; aa152-176) | Q9BP38_HALRU**^4^**  (aa1-171) | 77.8 | 3.4e-67 | 11  7  -  - | 115  105  -  - | 0.089  0.102  -  - | I_A_  I_B_  S_A_  S_B_ |
| **H_sp_Tri_119612_c0_g1_i1**  (aa1-165)  **Comp85967_c0_seq6_6**  **Comp85967_c0_seq5_6**  **Comp85967_c0_seq2_6** | Ribosomal protein L12; domains: Ribosomal_L11_N (aa1-80), Ribosomal_L11_C (aa81-144); 11.5% K; pI 9.5; IDR (37.8%; aa1-8,84-106,138-165) | V4AE09_LOTGI**^4^**  Lotgidraft_217320  (aa1-165) | 80.6 | 6.9e-55 | 2  2  -  - | 4  2  -  - | 0.001  0.002  -  - | I_A_  I_B_  S_A_  S_B_ |
| **H_sp_idb_44738_c0_g1_i1**  (aa18-289)  H_sp_CLC_11961_c0_g1_i1  Comp86117_c0_seq5_2 | Uncharacterized; domains: Galactose-bd-like (aa38-186,190-277); 9.2% T, 9.9% V; pI 5.6; IDR (2.0%; aa298-303) | G3PGN1_GASAC  (aa1-263) | 34.1 | 1.1e-21 | 2  2  -  - | 3  5  -  - | 0.002  0.001  -  - | I_A_  I_B_  S_A_  S_B_ |
| **H_sp_idb_47982_c0_g1_i1^3^**  (aa38-303)  **Comp86516_c0_seq2_3** | Uncharacterized; domain: CAP (aa62-234); SSP (aa1-18) or TM (aa13-35); pI 7.1; if SSP: pI 6.7 | V3ZVE9_LOTGI  Lotgidraft_160631  (aa51-314) | 50.0 | 1.0e-47 | 2  5  -  - | 2  25  -  - | 0.001  0.009  -  - | I_A_  I_B_  S_A_  S_B_ |
| **H_sp_Tri_53798_c0_g1_i1^3^**  (aa6-142)  **H_sp_idb_13782_c0_g1_i1**  **Comp87007_c0_seq1_6** | Similar to leukocyte cell-derived chemotaxin 1-like protein; domain: Brichos (aa66-148); SSP (aa1-22) or TM (aa4-26); 9.6% A, 11.5% V; pI 9.3; if SSP: pI 9.4; 10.2% V; (1 peptide not in this region); see also Tri_23898 and comp94109_c0_seq16_5 | D5FW85_HALDI  (aa3-138) | 27.3 | 5.5e-5 | 3  4  -  - | 14  14  -  - | 0.004  0.005  -  - | I_A_  I_B_  S_A_  S_B_ |
| **Comp87087_c0_seq1_3**  (aa70-191) | Uncharacterized; TM (aa12-34,64-86); pI 9.3 | V4AT91_LOTGI  Lotgidraft_157700  (aa18-130) | 46.7 | 2.3e-19 | 5  4  -  - | 37  13  -  - | 0.010  0.004  -  - | I_A_  I_B_  S_A_  S_B_ |
| **Comp87110_c0_seq1_1**  **Comp87110_c0_seq2_1** | Uncharacterized; SSP (aa1-28) or TM (aa13-35); 11.4% G, 11.4% Y; pI 9.1; if SSP: 10.3% Q, 15.4% G, 15.4% Y; pI 7.7 |  |  |  | 4  2  -  - | 34  37  -  - | 0.079  0.112  -  - | I_A_  I_B_  S_A_  S_B_ |
| **Comp87110_c0_seq4_2** | Uncharacterized; SSP (aa1-24) or TM (aa7-29), TM (aa95-117); 9.4% G, 15.0% Y; pI 8.6; IDR (14.0%; aa1-6,38-49); if SSP: 11.4% G, 18.1% Y; pI 8.9; IDR 11.4% |  |  |  | 2  2  -  - | 47  35  -  - | 0.029  0.021  -  - | I_A_  I_B_  S_A_  S_B_ |
| **H_sp_CLC_4146_c0_g1_i1^3^**  (aa4-343)  **Comp87152_c0_seq1_4** | Similar to endochitinase; domains: VWA (aa9-293), chitin-bd_II (aa239-293,294-347); SSP (aa1-17); pI 5.5 | K1R034_CRAGI  (aa7-350) | 36.8 | 1.3e-43 | 19  16  2  - | 632  479  3  - | 1.962  1.452  0.005  - | I_A_  I_B_  S_A_  S_B_ |
| **H_sp_idb_28957_c0_g1_i1^3^**  (aa1-651)  **Comp87346_c0_seq3_4**  **Q17UC1_HALTU** | Heat shock cognate protein 70/71; domains: HSP70_peptide-bd (aa387-518), HSP70_C (aa537-619); pI 5.2; IDR (24.7%; aa491-651); shares most peptides with comp79549_c0_seq1_2 | C1KC83_HALDV  (aa1-651) | 99.7 | 0e0 | 4  1  -  - | 12  6  -  - | 0.001  <0.001  -  - | I_A_  I_B_  S_A_  S_B_ |
| **H_sp_Tri_60505_c0_g1_i1**  (as1-271)  **Comp87374_c0_seq2_6**  **Comp87374_c0_seq4_5** | 14-3-3 zeta; domain: 14-3-3 (aa6-266); 11.0% A, 11.0% E; pI 5.0; IDR (14.7%; aa1-11,78-83,251-272); shares 1 peptide with CLC_3369 | W8E7M6_  HALDV  (aa1-271) | 93.0 | 1.1e-103 | 3  3  -  - | 11  31  -  - | 0.001  0.004  -  - | I_A_  I_B_  S_A_  S_B_ |
| **Comp88250_c0_seq2_2^3^** | Uncharacterized; TM (aa15-34); 9.6% S; pI 7.1; IDR (20.7%; aa1-12,39-57) |  |  |  | 3  2  2  - | 49  20  2  - | 0.025  0.024  0.007  - | I_A_  I_B_  S_A_  S_B_ |
| **H_sp_Tri_97777_c0_g1_i1^3^** (aa1-189)  **Comp88441_c0_seq3_1**  **Comp88441_c0_seq2_1** | Peptidyl-prolyl cis-trans isomerase (Fragment); domain: cyclophilin_like (aa26-194) SSP (aa1-18); 13.7% G, 13.7% K; pI 9.3; IDR (7.4%; aa194-208) | A0A0H4Q136  _HALDH  (aa1-189) | 94.2 | 1.3e-74 | 5  3  -  - | 21  11  -  - | 0.003  0.003  -  - | I_A_  I_B_  S_A_  S_B_ |
| **H_sp_idb_40380_c0_g1_i1**  (aa45-200)  **Comp89520_c0_seq1_4** | Similar to superoxide dismutase [Cu-Zn]; domain: SOD_Cu_Zn (aa45-200); SSP (aa1-21); 12.5% G; pI 8.5 | F6LQK4_HYRCU  (aa25-183) | 47.7 | 2.0e-29 | 3  3  -  - | 17  16  -  - | 0.016  0.011  -  - | I_A_  I_B_  S_A_  S_B_ |
| **H_sp_idb_35832_c0_g1_i1**  **Comp89562_c0_seq3_6**  **Comp89562_c0_seq1_6** | Uncharacterized; SSP (aa1-21); 11.5% D, 10.7% L, 9.9% S; pI 6.5 |  |  |  | 3  3  -  - | 11  12  -  - | 0.004  0.007  -  - | I_A_  I_B_  S_A_  S_B_ |
| **H_sp_idb_16122_c0_g1_i1**  (aa80-1022)  **Comp90174_c0_seq4_3** | Similar to alpha-mannosidase; domains: Glyco_hydro_38_N (aa36-372), Glyco_hydro_38_cen (aa379-471), Glyco_hydro_38_C (aa473-1022); SSP (aa1-22); pI 5.7; IDR (2.3%; aa641-646,986-1002) | K1QIID_CRAGI  (aa36-1022) | 61.4 | 0e0 | 6  7  -  - | 14  29  -  - | 0.001  0.002  -  - | I_A_  I_B_  S_A_  S_B_ |
| **H_sp_Tri_100026_c0_g1_i1**  (aa1-441)  **Comp90707_c0_seq1_2** | Putative GDP dissociation inhibitor; domain: GDI (1-434); pI 5.5; IDR (3.2%; aa429-442) | V5I1A9_IXORI  (aa2-446) | 74.0 | 4.2e-144 | 5  8  -  - | 14  38  -  - | 0.001  0.004  -  - | I_A_  I_B_  S_A_  S_B_ |
| **H_sp_Tri_85863_c0_g1_i1**  **Comp90993_c0_seq1_2** | Uncharacterized; domains: L-domain_like (Leu-rich repeat; aa26-226), LRR5 (aa112-236); SSP (aa1-22) 11.5% S; pI 9.0; IDR (2.2%; aa292-299) |  |  |  | 3  4  -  - | 8  10  -  - | 0.001  0.003  -  - | I_A_  I_B_  S_A_  S_B_ |
| **H_sp_CLC_5354_c0_g1_i1^3^**  (aa1-146)  **Comp91372_c0_seq1_1** | Peptidyl-prolyl cis-trans isomerase; domain: PPI_FKBP_type (aa48-141); SSP (aa1-21); 13.6% G, 14.4% K; pI 8.8 | K1R1I0_CRAGI  (aa1-145) | 76.9 | 1.1e-45 | 3  3  -  - | 5  12  -  - | 0.001  0.007  -  - | I_A_  I_B_  S_A_  S_B_ |
| **H_sp_Tri_102529_c0_g1_i1**  (aa16-98)  **H_sp_idb_15781_c0_g1_i1^3^**  **Comp91382_c0_seq1_6**  **Comp91382_c0_seq3_6** | Uncharacterized; domain: Cyt_B5-like_heme/steroid-bd (aa47-147); TM (aa10-32); 9.0% A, 10.8% L, 9.6% K, 9.6% V; pI 5.6; IDR (12.6%; aa105-119) | A0A0B7AZJ8  _9EUPU  (aa30-112) | 66.3 | 9.4e-25 | 3  2  -  - | 5  3  -  - | 0.001  <0.001  -  - | I_A_  I_B_  S_A_  S_B_ |
| **H_sp_Tri_122828_c0_g1_i1**  (aa44-329)  **Comp91393_c0_seq1_5** | Uncharacterized; domain: Na/K_ATPase_sub_beta (aa36-329). TM (aa77-99); pI 5.1; IDR (13.1%; aa1-43) | V4AVB7_LOTGI**^4^**  Lotgidraft_213719  (aa7-293) | 49.5 | 3.7e-69 | 7  6  -  - | 70  84  -  - | 0.014  0.026  -  - | I_A_  I_B_  S_A_  S_B_ |
| **H_sp_idb_8826_c0_g1_i1**  (aa7-661)  **Comp91733_c0_seq12_2**  **etc** | Protein kinase C¸domains: DAG/PE-bd (aa38-87,102-151), C2 (aa155-282), Kinase-like (aa333-657); pI 6.9, IDR (30.2%; aa1-25,156-331) | R7VI43_CAPTE  (aa1-666) | 76.8 | 1.9e-130 | 2  2  -  - | 14  19  -  - | 0.001  0.001  -  - | I_A_  I_B_  S_A_  S_B_ |
| **H_sp_Tri_129647_c0_g1_i1^3^**  (aa33-828)  **Comp92139_c0_seq1_6**  **Comp92139_c0_seq2_6** | Similar to latrophilin-3; domains: GAIN_dom_N (268-463), GPS (aa491-542), GPCR_2-like (aa552-796); multiple TM in aa555-793); 10.8% S, 12.8% T; pI 6.5; IDR (25.2%; aa1-188,809-840) | A0A210PIR3_  MIZYE  (aa156-943) | 42.1 | 8.3e-75 | 5  4  -  - | 19  10  -  - | 0.001  0.001  -  - | I_A_  I_B_  S_A_  S_B_ |
| **Comp92223_c1_seq1_1^3^**  (aa53-437)  **H_sp_idb_2286_c0_g1_i1** | Uncharacterized; domain: EF_hand_pair (aa250-347); TM (aa30-47); 12.7% Q, 11.4% E; pI 5.1; Q-rich C-term; IDP | A0A0B7AA85_  9EUPU  (aa22-394) | 57.3 | 6.5e-49 | 13  16  -  - | 99  108  -  - | 0.020  0.023  -  - | I_A_  I_B_  S_A_  S_B_ |
| **H_sp_Tri_91048_c0_g1_i1**  (aa30-163)  **Comp92274_c0_seq1_1** | Uncharacterized/similar to ribonuclease; domain: Endoribo_LPSP/chorism_mut-like (aa35-159); pI 9.0 | V4AMR8_LOTGI  Lotgidraft_208507  (aa1-134) | 67.9 | 3.8e-35 | 3  3  -  - | 3  4  -  - | 0.001  0.001  -  - | I_A_  I_B_  S_A_  S_B_ |
| **H_sp_idb_14124_c0_g1_i1**  (aa33-465)  **Comp92385_c0_seq9_2**  **Comp92385_c0_seq6_2** | Similar to carboxypeptidase; domain: AB_hydrolase (aa1-435); SSP (aa1-18); pI 5.6 | V4B2C7_LOTGI  Lotgidraft_134569  (aa1-435) | 68.9 | 1.6e-138 | 4  5  -  - | 49  77  -  - | 0.006  0.010  -  - | I_A_  I_B_  S_A_  S_B_ |
| **H_sp_Tri_61154_c0_g1_i1**  (aa30-424)  **Comp92600_c0_seq1_6** | Uncharacterized; domains: Aldolase_TIM (aa43-331), Glyco_hydro_b (333-425); TM (aa27-49); pI 5.0; IDR (2.8%; aa1-12) | V4AF69_LOTGI**^4^**  Lotgidraft_209340  (aa6-397) | 60.5 | 4.0e-111 | 2  2  -  - | 20  14  -  - | 0.002  0.003  -  - | I_A_  I_B_  S_A_  S_B_ |
| **H_sp_idb_18822_c0_g1_i1^3^**  (aa1-601)  **Comp92750_c0_seq3_1**  **Comp92750_c0_seq5_1** | Similar to ezrin/radixin/moesin; domains: FERM (aa5-295), ERM_C (aa333-601); 14.6% E, 10.5% L, 9.2% K; pI 5.4; IDR (51.4%; aa293-601) | A0A090XC63  _IXORI  (aa1-595) | 59.2 | 1.4e-64 | 16  18  -  - | 73  100  -  - | 0.008  0.015  -  - | I_A_  I_B_  S_A_  S_B_ |
| **Comp92770_c0_seq1_5**  (aa141-1034)  H_sp_Tri_21823_c0_g1_i1 | Uncharacterized/similar to glutamyl aminopeptidase; domains: Peptidase_M1_N (aa155-550), ERAP1-like_C (aa694-1014); TM (aa91-113,1189-1211,1218-1236); pI 8.2; IDR (1.9%; aa120-147) | V3ZTC8_LOTGI  Lotgidraft_130453  (aa4-890) | 63.4 | 0e0 | 7  5  -  - | 29  20  -  - | 0.001  0.001  -  - | I_A_  I_B_  S_A_  S_B_ |
| **H_sp_Tri_47901_c0_g1_i1^3^**  (aa16-363)  **Comp92949_c2_seq16_6**  **etc** | Similar to glycoprotein-N-acetyl-galactosamine 3-beta-galactosyl-transferase 1; domain: C1GALT1 (aa173-297); TM (aa13-30); pI 8.3; IDR (28.9%; aa1-11,33-103,359-391) | K1PSC7_CRAGI  (aa31-370) | 62.0 | 4.5e-98 | 3  3  -  - | 25  11  -  - | 0.004  0.003  -  - | I_A_  I_B_  S_A_  S_B_ |
| **H_sp_idb_48873_c0_g1_i1**  (aa6-403)  **Comp92958_c0_seq2_5**  **etc** | Uncharacterized/similar to alpha-N-acetylgalactosaminidase; domains: Aldolase_TIM (aa17-308), Glyco_hydro_b (310-402); SSP (aa1-16); pI 5.0 | A0A0B7BIS4  _9EUPU  (aa7-406) | 60.8 | 1.8e-120 | 3  3  -  - | 22  36  -  - | 0.002  0.002  -  - | I_A_  I_B_  S_A_  S_B_ |
| **Comp92993_c0_seq1_6**  (aa37-246)  **Comp92993_c0_seq2_6** | Uncharacterized; SSP/TM (aa1-30/12-34); pI 8.4; shares several peptides with Tri_100581 | V3ZDX8_LOTGI**^4^**  Lotgidraft_154424  (aa37-220) | 38.4 | 9.3e-21 | 4  1  -  - | 10  1  -  - | 0.002  <0.001  -  - | I_A_  I_B_  S_A_  S_B_ |
| **H_sp_Tri_100581_c0_g1_i1**  (aa27-236)  **H_sp_idb_41627_c0_g1_i1** | Uncharacterized; SSP (aa1-20); pI 6.1; shares several peptides with comp92993_c0_seq1_6 | V3ZDX8_LOTGI**^4^**  Lotgidraft_154424  (aa37-220) | 38.4 | 1.7e-20 | 1  1  -  - | 1  1  -  - | <0.001  0.004  -  - | I_A_  I_B_  S_A_  S_B_ |
| **H_sp_Tri_3904_c0_g1_i1^3^**  (aa23-647)  **Comp93346_c0_seq1_3**  **Comp93346_c0_seq2_2** | Similar to angiotensin-converting enzyme; domain: peptidase_M2_fam (aa70-647), SSP (aa1-20); pI 4.9; IDR (6.1%; aa38-73) | C3Z9J8_BRAFL  (aa38-666) | 47.2 | 5.7e-126 | 3  2  -  - | 3  2  -  - | <0.001  <0.001  -  - | I_A_  I_B_  S_A_  S_B_ |
| **Comp93747_c0_seq2_4**  (aa26-621**)**  **Comp93747_c0_seq1_4**  H_sp_Tri_83241_c0_g1_i1 | Similar to beta-glucuronidase; domains: Galactose-bd-like (aa21-214), Glyco_hydro_2/20_Ig-like (216-316), Glycoside_hydrolase_SF 317-616); SSP/TM (aa1-20/2-24), TM (aa669-691,745-767,777-799); pI 8.2 | V4A2U2_LOTGI  Lotgidraft_166508  (aa1-598) | 66.6 | 6.0e-195 | 2  4  -  - | 8  14  -  - | <0.001  0.001  -  - | I_A_  I_B_  S_A_  S_B_ |
| **H_sp_Tri_8133_c0_g1_i1^3^**  (aa3-456)  **Comp93947_c0_seq8_1**  **Comp93947_c0_seq11_1** | Similar to intermediate filament protein; domains: Intermed_filament_ifa/ifb (aa1-597); pI 5.6; IDR (37.2%; aa1-85,294-352,426-489,505-512,593-599) | V4CQU1_LOTGI**^4^**  Lotgidraft_109284  (aa6-457) | 51.8 | 7.5e-63 | 20  20  -  - | 182  165  -  - | 0.024  0.025  -  - | I_A_  I_B_  S_A_  S_B_ |
| **H_sp_Tri_91049_c0_g1_i1^3^**  (aa1-363  **Comp93951_c0_seq1_3** | Uncharacterized; domains: villin/gelsolin (aa67-193,194-303,304-395); 11.3% E; pI 4.6; IDR (15.7%; aa1-40,254-270) shares 1 peptide with CLC_5447 | R7U815_CAPTE  (aa1-365) | 58.3 | 2.6e-78 | 7  9  -  - | 69  68  -  - | 0.006  0.012  -  - | I_A_  I_B_  S_A_  S_B_ |
| **H_sp_CLC_4517_c0_g1_i1^3^**  (aa12-210)  **Comp94020_c0_seq1_3** | Similar to peptidyl-prolyl cis-trans isomerase FKBP14; domains: PPIase_FKBP (aa43-132), EF-hand-dom_pair (aa142-202); SSP (aa1-22); 13.3% D, 10.1% K; pI 4.6; IDR (40.4%; aa134-210) | V4B2Q8_LOTGI**^4^**  Lotgidraft_238515  (aa9-208) | 62.7 | 2.6e-47 | 2  2  -  - | 6  17  -  - | 0.009  0.004  -  - | I_A_  I_B_  S_A_  S_B_ |
| **H_sp_idb_10555_c0_g1_i1**  (aa28-8819)  **Comp94035_c1_seq3_6**  **Comp94035_c1_seq7_6**  Comp62063_c0_seq1_1**^3^** | Similar to elongation factor 2; domains: TF_GTP-bd (aa27-382), Transl_B-barrel (aa383-520), EFG_III-V (aa523-598,765-878), Ribosomal_S5_D2-typ_fold (aa600-764); pI 8.2; IDR (0.9%; aa1-8) | A0A094MKW9  _ANTCR  (aa1-857) | 73.6 | 1.6e-193 | 5  10  -  - | 26  27  -  - | 0.001  0.003  -  - | I_A_  I_B_  S_A_  S_B_ |
| **H_sp_Tri_61464_c0_g1_i1**  (aa1-363)  **Comp94177_c0_seq2_3**  **Comp94177_c0_seq1_3** | Uncharacterized; SSP/TM (aa1-19/5-22); 11.1% L, 10.9% S; pI 5.1; if SSP: 10.6% L, 11.0% S; pI 5.0 |  |  |  | 4  4  -  - | 12  11  -  - | 0.001  0.002  -  - | I_A_  I_B_  S_A_  S_B_ |
| **Comp94326_c0_seq1_4**  (aa37-466)  **Comp94326_c0_seq2_5**  **H_sp_Tri_111044_c0_g1_i1** | Similar to beta-hexosaminidase; domains: HEX_eukaryotic_N-term (aa42-181), Glycoside_hydrolase_SF (aa182-456) | V3ZMB9_LOTGI  Lotgidraft_120422  (aa52-488) | 58.3 | 4.0e-114 | 2  3  -  - | 11  9  -  - | 0.002  0.002  -  - | I_A_  I_B_  S_A_  S_B_ |
| **Comp94706_c0_seq1_3^3^**  (aa55-255)  H_sp_idb_32872_c0_g1_i1 | Uncharacterized; TM (aa7-29); pI 8.9 | V3ZY26_LOTGI**^4^**  Lotgidraft_154423  (aa10-212) | 34.1 | 7.3e-24 | 7  5  -  - | 83  110  -  - | 0.018  0.026  -  - | I_A_  I_B_  S_A_  S_B_ |
| **H_sp_CLC_3369_c0_g1_i1^3^**  (aa35-291)  **Comp94743_c0_seq3_1** | Uncharacterized/similar to 14-3-3 epsilon; domain: 14-3-3 (aa36-280); 9.3% A, 11.0% E, 10.0% L, 9.6% S; pI 4.7; IDR (21.7%; aa1-9,22-40,102-110,265-291) | K7J834_NASVI  (aa2-256) | 83.3 | 2.6e-87 | 8  7  -  - | 65  89  -  - | 0.011  0.017  -  - | I_A_  I_B_  S_A_  S_B_ |
| **H_sp_idb_16143_c0_g1_i1**  (aa175-394)  (aa36-294)  **Comp94779_c0_seq1_5**  **etc** | Uncharacterized; domains: PDZ (aa30-124,160-253,292-383); 11.0% V; pI 4.8; IDR (27.6%; aa1-35,133-152,250-293,397-410) | D2HD24_AILME  (aa2-216)  H2ZQ66_CIOSA  (aa6-269) | 42.9  38.7 | 2.0e-25  6.0e-25 | 6  4  -  - | 22  17  -  - | 0.009  0.005  -  - | I_A_  I_B_  S_A_  S_B_ |
| **Comp95150_c0_seq8_1**  (aa1-217) | Arginine kinase; domain: Gln_synth/guanido_kin_cat (aa1-210); pI 9.2; IDR (1.3%; aa224-231); shares 2 peptides with idb_28997 | KARG_TURCO  (aa139-357) | 75.3 | 4.2e-75 | 3  3  -  - | 17  39  -  - | 0.002  0.007  -  - | I_A_  I_B_  S_A_  S_B_ |
| **H_sp_idb_28997_c0_g1_i1**  (aa21-375)  Comp80852_c0_seq1_1  Comp134761_c0_seq1_4 | Arginine kinase; domain: ATP-guanido_PTrfase_N (aa22-112), ATP-guanido_PTrfase_cat (aa124-371); 10.1% L, 9.6% K; pI 6.4; IDR (9.8%; aa1-13,110-126,329-335); shares 2 peptides with comp95150_c0_seq8_1 | A0A0D5BGB7  _HALDV  (aa2-354) | 80.3 | 1.6e-124 | 10  16  -  - | 93  182  -  - | 0.013  0.048  -  - | I_A_  I_B_  S_A_  S_B_ |
| **H_sp_Tri_91277_c0_g1_i1**  (aa1-1032)  **Comp95743_c0_seq1_6**  Q5XPR3_HALTU | Sodium/potassium-transporting ATPase subunit alpha; domains: ATPase_P-typ_transduc_dom_A (aa62-105,144-374), ATPase_P-typ_cyto_domN (aa374-598), ATPase_P-typ_cation-transptr_C (aa808-1017); multiple TM; pI 5.4; IDR (7.5%; aa1-68,234-242) | A0A0B7AV89  _9EUPU  (aa1-1031) | 88.2 | 0e0 | 25  27  -  - | 201  214  -  - | 0.012  0.020  -  - | I_A_  I_B_  S_A_  S_B_ |
| **Comp95780_c0_seq2_4^3^** | Uncharacterized; TM (aa29-51,187-209); pI 10.0 |  |  |  | 2  2  -  - | 58  5  -  - | 0.013  0.001  -  - | I_A_  I_B_  S_A_  S_B_ |
| **H_sp_CLC_3294_c0_g1_i1**  (aa63-1246)  **Comp95784_c0_seq3_3**  **Comp95784_c0_seq1_2**  **Comp95784_c0_seq4_3** | Similar to metalloendopeptidase; domains: Peptidase_Metallo (aa72-212), ApeC (aa327-518), CUB (aa525-637), Kringle-like (646-731), Sushi_SCR_CCP (aa786-839,1029-1083), C-type_lectin_fold (826-944); SSP (aa1-33); IDR (4.3%; aa42-73,792-800, 1100-1112) | V4AWP6_LOTGI  Lotgidraft_172278  (aa184-1388) | 27.5 | 2.5e-76 | 4  11  -  - | 7  29  -  - | 0.001  0.005  -  - | I_A_  I_B_  S_A_  S_B_ |
| **H_sp_Tri_81596_c0_g1_i1**  (aa4-162)  **Comp96576_c0_seq1_4** | Uncharacterized; domain: UspA (aa10-153); 9.3% G, 12.3% V; pI 6.8, IDR (14.8%; aa1-11,150-162) | V4AQH5_LOTGI  Lotgidraft_203658  (aa3-161) | 73.0 | 1.9e-48 | 6  4  -  - | 31  17  -  - | 0.016  0.018  -  - | I_A_  I_B_  S_A_  S_B_ |
| **H_sp_Tri_73887_c0_g1_i1**  (aa16-124)  **Comp96622_c0_seq1_3** | Uncharacterized; SSP (aa1-23); 12.4% T; pI 5.4 | K1PQY0_CRAGI  (aa15-130) | 45.7 | 2.0e-17 | 3  2  -  - | 22  4  -  - | 0.110  0.029  -  - | I_A_  I_B_  S_A_  S_B_ |
| **Comp96813_c0_seq1_5^3^**  (aa40-242)  (aa54-372)  H_sp_CLC_6293_c0_g1_i1 | Similar to cystatin/cathepsin F; domains: cystatin (aa39-126,130-241); pI 8.6; IDR (7.0%; aa237-262)shares most peptides with idb_44191 | I1T027_HALDH  (aa62-269)  K1QYP7_CRAGI  (aa17-352) | 37.5  28.0 | 1.8e-27  1.3e-12 | -  1  -  - | -  2  -  - | -  <0.001  -  - | I_A_  I_B_  S_A_  S_B_ |
| **H_sp_idb_44191_c0_g1_i1^3^**  (aa38-712)  Comp105290_c0_seq2_6 | Similar to cathepsin F; domains: cystatin (aa23-111,114-225), peptidase_I29 (aa411-468), peptidase_C1A (aa499-710); SSP (aa1-17); pI 6.0; IDR (23.9%; aa115-129,217-290,356-403,477-508) | K1QYP7_CRAGI  (aa17-715) | 46.6 | 3.4e-96 | 19  17  -  - | 175  151  -  - | 0.031  0.027  -  - | I_A_  I_B_  S_A_  S_B_ |
| **Comp96833_c0_seq1_1**  (aa189-1590) | Similar to fibropellin-1; domains: C-type_lectin (aa227-382), LDrepeatLR_classA_rpt (aa375-417), CUB (aa423-531,540-651,652-763), EGF_like (aa994-1036,1038-1091), multiple Sushi_SCR_CCP; IDR (8.2%; aa101-118,950-960,1491-1593) | K1RC33_CRAGI  (aa8-1355) | 33.4 | 9.3e-54 | 9  6  -  - | 35  19  -  - | 0.002  0.001  -  - | I_A_  I_B_  S_A_  S_B_ |
| **Comp97057_c2_seq2_1**  (aa31-169)  **H_sp_idb_8482_c0_g1_i1**  **H_sp_Tri_108434_c0_g1_i1** | Uncharacterized; domain: Man6P_isomerase_rcpt-bd (aa36-167); 10.7% T; pI 5.7; IDR (4.7%; aa1-8) | R7VHI3_CAPTE  (aa9-156) | 36.2 | 4.9e-8 | 2  2  -  - | 11  9  -  - | 0.005  0.005  -  - | I_A_  I_B_  S_A_  S_B_ |
| **H_sp_CLC_17798_c0_g1_i1**  (aa75-716)  **H_sp_idb_6762_c0_g1_i1**  **Comp97289_c0_seq1_2**  **Comp97289_c0_seq2_2** | Similar to protein OS-9; domain: Glucosidase_II_beta_ subunit-like (PRKCSH; aa104-223); IDP | K1P3H0_CRAGI  (aa1-647) | 41.4 | 1.9e-58 | 3  3  -  - | 7  9  -  - | 0.001  0.001  -  - | I_A_  I_B_  S_A_  S_B_ |
| **H_sp_idb_19028_c0_g1_i1**  (aa38-642)  **Comp97297_c0_seq2_4** | Uncharacterized; domain: peptidase_M12B (aa34-285); SSP (aa1-19); pI 5.3; IDR (6.7%; aa20-30,656-689) | V4AHG6_LOTGI  Lotgidraft_174864  (aa167-783) | 49.5 | 1.3e-119 | 8  6  -  - | 42  17  -  - | 0.013  0.003  -  - | I_A_  I_B_  S_A_  S_B_ |
| **H_sp_Tri_137490_c0_g1_i1^3^**  (aa18-139)  **Comp97298_c0_seq1_5** | Similar to calcium-binding protein; domains: EF_hand (aa9-44,45-77,84-115); 13.7% D, 9.6% E; pI 4.3; IDR (11.0%; aa1-11,142-146) | B0EE99_ENTDS  (aa6-130) | 30.2 | 2.8e-4 | 5  4  -  - | 37  48  -  - | 0.015  0.038  -  - | I_A_  I_B_  S_A_  S_B_ |
| **H_sp_idb_15018_c0_g1_i1**  (aa56-383)  **Comp97336_c1_seq3_4**  **etc** | Uncharacterized/similar to quinone oxidoreductase; domains: ADH_N (aa61-212), ADH_C (aa214-326); 11.5% G; pI 7.1 | V3ZN01_LOTGI  Lotgidraft_232898  (aa2-331) | 58.9 | 3.8e-68 | 2  2  -  - | 4  2  -  - | <0.001  <0.001  -  - | I_A_  I_B_  S_A_  S_B_ |
| **H_sp_idb_26594_c0_g1_i1**  (aa16-388)  **Comp97362_c0_seq6_5**  **Comp97362_c0_seq3_5** | Similar to deleted in malignant brain tumors 1; domains: CUB (aa22-138,144-255,263-383); SSP (aa1-21); pI 5.7 | E9QPG8_MOUSE  (aa992-1348) | 26.4 | 7.5e-23 | 7  5  -  - | 43  26  -  - | 0.007  0.003  -  - | I_A_  I_B_  S_A_  S_B_ |
| **Comp97413_c0_seq7_1**  (aa107-365)  **Comp97413_c0_seq1_3**  **Comp97413_c0_seq5_2**  H_sp_Tri_119340_c0_g1_i1 | Uncharacterized; domain: Carbonic_anhydrase_a (aa108-365); TM (aa58-80); 9.8% S; pI 6.5; shares peptide with idb_58049 | V4AHB6_LOTGI**^4^**  Lotgidraft_205401  (aa1-260) | 57.7 | 1.5e-69 | -  2  -  - | -  6  -  - | -  0.003  -  - | I_A_  I_B_  S_A_  S_B_ |
| **H_sp_idb_58049_c0_g1_i1**  (aa2-258)  Comp97413_c0_seq2_1 | Uncharacterized; domain: carbonic_anhydrase (aa1-258); IDR (8.9%; aa1-23); shares 1 peptide with comp97413_c0_seq7_1 | V4AHB6_LOTGI**^4^**  Lotgidraft_205401  (aa3-260) | 57.8 | 9.7e-68 | 2  -  -  - | 3  -  -  - | 0.003  -  -  - | I_A_  I_B_  S_A_  S_B_ |
| **H_sp_CLC_5447_c0_g1_i1**  (aa3-310)  **H_sp_idb_15505_c0_g1_i1**  **Comp97886_c0_seq1_6** | Uncharacterized; domains: gelsolin-like (aa30-154,155-268); IDR (27.4%; aa1-39,225-278); shares 1 peptide with Tri_91049 | A0A0B6ZFV9  _9EUPU  (aa28-334) | 73.1 | 4.9e-101 | 2  3  -  - | 5  15  -  - | 0.001  0.003  -  - | I_A_  I_B_  S_A_  S_B_ |
| **H_sp_Tri_52786_c0_g1_i1^3^**  (aa17-224)  **Comp98044_c0_seq2_2**  **Comp98044_c0_seq5_2** | Uncharacterized; domain: PLipase_A2 (aa111-232); SSP (aa1-26); pI 7.9; IDR (4.2%; aa94-103) | T1INX7_STRMM  (aa10-213) | 35.8 | 1.1e-24 | 4  5  -  - | 18  34  -  - | 0.005  0.014  -  - | I_A_  I_B_  S_A_  S_B_ |
| **H_sp_idb_2169_c0_g1_i1**  (aa315-1054)  **Comp98123_c0_seq1_3**  **Comp98123_c0_seq2_1** | Similar to beta-hexosaminidase_ subunit_beta; domains: Chitobiase/Hex_dom_2-like (aa385-550), Glycoside_hydrolase_SF (aa549-927); SSP (aa1-18); TM (aa980-1002,1023-1045); pI 6.1; IDR (26.6%; aa40-62,72-325,1054-1064) | K1PUG5_CRAGI  (aa2-696) | 57.5 | 5.2e-86 | 9  6  -  - | 32  28  -  - | 0.003  0.003  -  - | I_A_  I_B_  S_A_  S_B_ |
| **H_sp_idb_45022_c0_g1_i1**  (aa24-280)  **Comp98351_c4_seq1_5** | Uncharacterized; domain: solute-binding_3/MltF_N (aa41-250); SPP (aa1-22); pI 5.1 | A0A0B6ZXS1  _9EUPU  (aa36-294) | 58.7 | 1.2e-72 | 7  4  -  - | 31  26  -  - | 0.059  0.068  -  - | I_A_  I_B_  S_A_  S_B_ |
| **H_sp_idb_4984_c0_g1_i1**  (aa11-974)  **Comp99132_c0_seq3_2**  **Comp99132_c0_seq2_2** | Uncharacterized/similar to exostosin-3-like; domains: exostosin_fam (aa197-557), EXT_C (aa718-959); TM (aa28-47); pI 6.6; IDR (9.3%; aa1-11,58-66,166-173,,364-428) | A0A0L8GTK9  _OCTBM  (aa7-977) | 51.0 | 7.6e-186 | 4  4  -  - | 8  8  -  - | 0.001  0.001  -  - | I_A_  I_B_  S_A_  S_B_ |
| **H_sp_idb_9026_c0_g1_i1^3^**  (aa28-351)  **Comp99181_c0_seq1_4**  **H_sp_idb_9027_c0_g1_i1** | Uncharacterized; SSP (aa1-22)/TM (aa5-22); pI 4.8; IDR (2.8%; aa468-481); if SSP: pI 4.8; IDR 2.7% | V4BEK2_LOTGI  Lotgidraft_171050  (aa1-332) | 25.2 | 5.1e-10 | 3  4  -  - | 8  6  -  - | 0.001  0.002  -  - | I_A_  I_B_  S_A_  S_B_ |
| **H_sp_idb_22405_c0_g1_i1**  (aa70-471)  **Comp99282_c0_seq1_3** | Similar to serpin-like protein; domain: serpin (aa81-476); SSP (aa1-16); pI 9.4 | I1SY93_HALDH  (aa2-404) | 65.4 | 6.7e-113 | 5  6  -  - | 11  20  -  - | 0.002  0.003  -  - | I_A_  I_B_  S_A_  S_B_ |
| **Comp99376_c0_seq35_5**  (aa193-1085)  **H_sp_CLC_2482_c0_g1_i1** | Similar to alpha-mannosidase; domains Glyco_hydro/deAcase_b/a-brl (187-513), Glyco_hydro_57/38 _cen (aa517-617, Glyco_hydro_ 38_C (aa613-1273); TM (aa139-158); pI 9.0; IDR (3.9%; aa1-13,175-188,719-735,946-960) | V3YVL3_LOTGI  Lotgidraft_135457  (aa8-892) | 50.6 | 4.6e-202 | 3  2  -  - | 6  4  -  - | <0.001  <0.001  -  - | I_A_  I_B_  S_A_  S_B_ |
| **Comp99505_c0_seq1_5**  (aa66-414)  H_sp_CLC_4237_c0_g1_i1 | Uncharacterized; domain: chitinase_II (aa67-415); multiple TM; pI 7.8; IDR (1.4%; aa1-10) | A0A0B6ZXL3  _9EUPU  (aa32-373) | 47.6 | 8.3e-73 | 3  5  -  - | 13  15  -  - | 0.001  0.002  -  - | I_A_  I_B_  S_A_  S_B_ |
| **H_sp_Tri_2770_c0_g1_i1^3^**  (aa1-670)  **Comp99703_c0_seq1_2**  Comp136401_c0_seq1_5 | 78kDa glucose-regulated protein; 9.4% E; pI 4.9; domains: HSP70_peptide-bd (aa426-568,571-653); shares several peptides with comp136401_c0_seq1_5; IDR (27.6%; aa20-41,289-296,489-495,523-670) | Q75W49_CRAGI  (aa1-661) | 84.5 | 8.5e-201 | 8  4  -  - | 27  10  -  - | 0.003  0.001  -  - | I_A_  I_B_  S_A_  S_B_ |
| **H_sp_CLC_11155_c0_g1_i1** | Uncharacterized; 17.5% G, 43.8% S, 17.8% T; pI 4.3; IDP; only 1 identifiable peptide predicted |  |  |  | 1  1  1  1 | 3  4  6  3 | 0.003  0.007  0.703  0.171 | I_A_  I_B_  S_A_  S_B_ |
| **H_sp_CLC_1125_c0_g1_i1^3^**  (aa10-345)  (aa11-850) | Similar to chitin-binding protein; domain: Cellulose/chitin-bd_N (aa28-214); SSP (aa1-27)/TM (aa5-27); aa510-610 T-rich (56%); pI 8.3; IDR (62.8%; aa263-870); if SSP: 9.5% P, 13.7% T; pI 8.5; IDR: 64.3% | A0A075LZK8_  PINMT  (aa5-362)  K1QE07_CRAGI  (aa1-837) | 42.1  23.8 | 1.2e-26  9.5e-19 | 9  7  -  - | 90  26  -  - | 0.008  0.002  -  - | I_A_  I_B_  S_A_  S_B_ |
| **H_sp_CLC_12_c0_g1_i1^3^**  (aa45-461) | Uncharacterized; 9.7% T; pI 6.1; IDR (9.7%; aa31-49,438-463) | V4AKF1_LOTGI  Lotgidraft_228685  (aa30-466) | 29.8 | 1.1e-22 | 10  8  -  - | 107  74  -  - | 0.049  0.034  -  - | I_A_  I_B_  S_A_  S_B_ |
| **H_sp_CLC_12027_c0_g1_i1^3^**  (aa81-180)  **H_sp_idb_54497_c0_g1_i1** | Uncharacterized; SSP (aa1-19)/TM (aa7-29), TM (aa65-87); 18.3% G, 14.1% M, 10.0% P, pI 9.4; IDR (57.8%; aa8-111); tandem repeats (Fig. S2A); if SSP: 19.8 % G, 15.4% M, 9.9% P; pI 9.6; IDR: 63.9% |  |  |  | 9  6  -  - | 99  47  -  - | 0.395  0.087  -  - | I_A_  I_B_  S_A_  S_B_ |
| **H_sp_CLC_123_c0_g1_i1^3^**  (aa2-455)  **H_sp_idb_32947_c0_g1_i1** | Similar to tyrosinase; domains: Tyrosinase_Cu-bd (aa18-271); SSP (aa1-20); 14.6% G; pI 9.0; IDR (24.5%; aa350-457); tandem repeats (Fig. S2B) | V4AN59_LOTGI  Lotgidraft_160808  (aa13-518) | 43.3 | 3.0e-38 | 7  6  2  - | 193  163  6  - | 0.141  0.132  0.026  - | I_A_  I_B_  S_A_  S_B_ |
| **H_sp_CLC_1320_c0_g1_i1^3^**  (aa1-133)  **H_sp_Tri_133893_c0_g1_i1**  **H_sp_Tri_133894_c0_g1_i1** | Lustrin A (fragment); domain: Lustrin_cystein (aa67-108); SSP (aa1-19); 10.3% C, 24.8% P; pI 6.7; C-term IDR (20.6%; aa109-136); tandem repeats aa115-136 (PPA)_7_ | O44341_HALRU**^4^**  (aa1-133) | 76.7 | 7.3e-23 | 4  3  3  - | 37  24  7  - | 0.025  0.023  0.086  - | I_A_  I_B_  S_A_  S_B_ |
| **H_sp_CLC_608_c0_g1_i1^3^**  (aa1-179)  O44341_HALRU | Lustrin A (fragment); O44341_HALRU aa1175-1362; 16.1% G, 26.1% S; pI 4.5; IDR (56.1%; aa1-101) | F6KD05_HALTU  (aa205-380) | 78.3 | 8.1e-28 | 2  1  1  1 | 34  27  1  1 | 0.043  0.019  0.017  0.007 | I_A_  I_B_  S_A_  S_B_ |
| **H_sp_Tri_116352_c0_g1_i1^3^**  (aa16-652) | Similar to lustrin A; domains: multiple Cys_repeat_1;11.0% C, 19.6% P; pI 6.5; IDR (10.1%; aa1-40,123-139); shares several peptides with idb_288, Tri_116349 | O44341_HALRU**^4^**  (aa226-872) | 62.6 | 1.3e-103 | 6  -  5  3 | 73  -  11  6 | 0.009  -  0.019  0.014 | I_A_  I_B_  S_A_  S_B_ |
| **H_sp_CLC_1340_c0_g1_i1**  (aa22-849) | Similar to prestin; domains: SLC26A/SulP (aa104-507), STAS (aa557-820); multiple TM; pI 7.8; IDR (23.6%; aa1-40,608-742,825-849) | K1QK91_CRAGI  (aa3-665) | 56.5 | 7.5e-167 | 5  7  -  - | 22  23  -  - | 0.003  0.004  -  - | I_A_  I_B_  S_A_  S_B_ |
| **H_sp_CLC_146_c0_g1_i1^3^**  H_sp_idb_13068_c0_g1_i1 | Uncharacterized; domains: VWA (aa50-236), ConA_like (aa712-884); 14.0% S, 13.6% T; IDR (18.8%; aa321-421,549-572,595-632) |  |  |  | 22  18  -  - | 246  207  -  - | 0.043  0.041  -  - | I_A_  I_B_  S_A_  S_B_ |
| **H_sp_CLC_15249_c0_g1_i1^3^**  (aa35-337)  Comp82445_c0_seq1_6 | Uncharacterized; SSP (aa1-32)/TM (aa5-27); pI 4.8 | V4AKU8_LOTGI  Lotgidraft_228332  (aa25-334) | 22.1 | 1.9e-5 | 7  8  -  - | 31  28  -  - | 0.007  0.005  -  - | I_A_  I_B_  S_A_  S_B_ |
| **H_sp_CLC_1545_c0_g1_i1**  (aa11-3429)  Comp98763_c0_seq3_3  Comp56050_c0_seq1_1  Comp283473_c0_seq1_1  C7FEG7_HALDV  B4YIJ4_HALDV  Q9U5P3_HALTU | Hemocyanin H1; domains: multiple Unchr_di-copper_centre, multiple Haemocyanin_beta-sandwich; pI 5.8; IDR (1.5%; aa690-698,986-992,2513-2541,2925-2932) | Q9GP18_HALTU  (aa1-3419) | 91.2 | 0e0 | 49  53  -  - | 439  495  -  - | 0.017  0.019  -  - | I_A_  I_B_  S_A_  S_B_ |
| **H_sp_CLC_160_c0_g1_i1**  (aa1-198)  EPDR1_HALAI | Similar to ependymin-related protein 1; domain: ependymin (aa71-198); SSP (aa1-17); shares many peptides with Tri_31892 and Tri_31898 | EPDR1_HALAI**^4^**  (aa1-198) | 77.3 | 2.7e-72 | 9  18  -  - | 355  621  -  - | 0.728  3.133  -  - | I_A_  I_B_  S_A_  S_B_ |
| **H_sp_CLC_18633_c0_g1_i1^3^**  (aa12-222) | Uncharacterized; domain: Chitin-bd_N (aa32-220); TM (aa7-26); 13.3% T; pI 8.5; IDR (31.7%; aa221-364) | V3ZNR4_LOTGI  Lotgidraft_119849  (aa4-211) | 51.2 | 1.6e-27 | 9  6  -  - | 50  37  -  - | 0.012  0.008  -  - | I_A_  I_B_  S_A_  S_B_ |
| **H_sp_CLC_1876_c0_g1_i1^3^**  (aa2-198) | Similar to ependymin-1; domain: ependymin (aa71-198); 11.8% L, 10.6% S; pI 7.7; shares several peptides with Tri_31892 and idb_40080 | EPDR1_HALAI**^4^**  (aa1-198) | 71.2 | 1.6e-65 | 6  6  -  - | 129  167  -  - | 0.328  0.577  -  - | I_A_  I_B_  S_A_  S_B_ |
| **H_sp_CLC_1949_c0_g1_i1^3^** | Uncharacterized; 10.9% R, 17.5% Q, 10.5% S; pI 11.6, IDP; shares several peptides with idb_12174 and CLC_8754 |  |  |  | 3  3  1  - | 68  73  1  - | 0.025  0.052  <0.001  - | I_A_  I_B_  S_A_  S_B_ |
| **H_sp_idb_12174_c0_g1_i1^3^** | Uncharacterized; TM (aa12-31); 20.0% Q,11.5%S, pI 10.6; IDP; share several peptides with CLC_1949 |  |  |  | 24  21  5  4 | 296  332  41  24 | 0.078  0.093  0.053  0.033 | I_A_  I_B_  S_A_  S_B_ |
| **H_sp_Tri_107535_c0_g1_i1^3^**  (aa8-371)  **H_sp_CLC_21_c0_g1_i1**  GAAP_HALAI | Similar to glycine-, alanine- and asparagine-rich protein; 31.8% A, 17.8% G, 21.3% S; pI 12.0; IDR (20.2%; aa1-34,331-371); repeats (Fig. S2C) | GAAP_HALAI**^4^**  (aa30-443) | 77.5 | 3.0e-44 | 18  18  5  3 | 534  473  32  26 | 0.527  0.510  0.739  0.256 | I_A_  I_B_  S_A_  S_B_ |
| **H_sp_CLC_2296_c0_g1_i1^3^**  (aa6-674) | Similar to chitinase-3; domains: chitinase_II (aa34-408); chitin-bd_II (aa468-531,619-675); SSP (aa1-31)/TM (aa7-29); 9.0% P, 9.8% T; pI 8.5; IDR (23.7; aa412-475,523-618); if SSP: 10.2% T; pI 8.5; IDR: 24.5% | J7FIC1_HYRCU  (aa2-651) | 45.7 | 9.8e-86 | 13  12  -  - | 103  65  -  - | 0.011  0.008  -  - | I_A_  I_B_  S_A_  S_B_ |
| **H_sp_idb_2023_c0_g1_i1^3^**  (aa13-397)  **H_sp_CLC_2607_c0_g1_i1**  **H_sp_idb_2021_c0_g1_i1** | Uncharacterized; domains: IG (120-235), chitin-bd_II (aa347-404); 10.1% S, 9.4% T; pI 5.5; IDR (4.5%; aa39-56) | V3ZHM8_LOTGI  Lotgidraft_236408  (aa136-503) | 22.3 | 2.6e-13 | 3  3  -  - | 12  8  -  - | 0.003  0.003  -  - | I_A_  I_B_  S_A_  S_B_ |
| **H_sp_idb_59441_c0_g1_i1**  (aa12-215)  UP6_HALAI | Similar to uncharacterized protein 6; TM (aa10-29); 10.2% A, 10.2% L, 10.2% T; pI 9.2; see also idb_27788! | UP6_HALAI**^4^**  (aa1-204) | 71.1 | 1.3e-64 | 2  6  -  - | 10  86  -  - | 0.006  0.268  -  - | I_A_  I_B_  S_A_  S_B_ |
| **H_sp_CLC_303_c0_g1_i1^3^** | Uncharacterized; aa615-745 almost identical to hasininaP0013F12_631**^4^**; domains: chitin_bd_II (aa350-410,411-475), ConA-like (aa584-731); 21.3% A, 9.8% Q; pI 6.7; IDR (48.7%; aa1-363); repeats (Fig. S2E) |  |  |  | 27  20  5  4 | 415  277  10  6 | 0.093  0.080  0.005  0.003 | I_A_  I_B_  S_A_  S_B_ |
| **H_sp_CLC_3466_c0_g1_i1^3^**  (aa156-679)  Comp16849_c0_seq2_2 | Similar to metalloendopeptidase; domains: CUB (aa403-518), TSP1 (aa582-632); SSP (aa1-19); pI 8.6; IDR (1.5%; aa117-127) | A0A077YYY8  _TRITR  (aa92-556) | 31.2 | 1.5e-42 | 17  9  2  - | 192  51  4  - | 0.050  0.007  0.009  - | I_A_  I_B_  S_A_  S_B_ |
| **H_sp_CLC_3878_c0_g1_i1^3^**  (aa8-440)  (aa38-804) | Uncharacterized/IgGFc-binding protein; domains: chitin-bd_II (aa32-90,96-163,236-300,325-374), Sushi_SCR_CCP (aa475-534,733-792,946-1004), galectin_CRD (aa1134-1289), FA58C_3 (aa1134-1289); SSP (aa1-20); 9.3% S, 10.2% T; pI 6.1; shares several peptides with Tri_120379 and idb_26484/idb_2772; IDR (11.8%; aa393-433,660-742,779-800) | K1QJK2_CRAGI  (aa13-458)  K1PRD3_CRAGI  (aa1235-2010) | 28.2  24.2 | 6.3e-16  1.7e-10 | 7  7  -  - | 83  67  -  - | 0.009  0.008  -  - | I_A_  I_B_  S_A_  S_B_ |
| **H_sp_idb_2768_c0_g1_i1^3^**  (aa40-1286) | Uncharacterized; domains: fucolectin/tachylectin-4/pentraxin-1 (FTP1; aa57-205,860-1007), Sushi_SCR_CCP (aa284-346,659-719,1012-1072), galectin (aa353-480),  Gal_bd (aa492-647); pI 5.7; shares most peptides with Tri_120379; ; IDR (3.5%; aa492-501,710-725,1231-1249) | C3XSY6_BRAFL  (aa17-1131) | 24.7 | 1.9e-6 | 9  4  -  - | 65  34  -  - | 0.004  0.002  -  - | I_A_  I_B_  S_A_  S_B_ |
| **H_sp_idb_2772_c0_g1_i1^3^**  (aa2-440)  (aa38-804) | Uncharacterized; shares several peptides with Tri_120379, CLC_3878, Tri_120377 and idb_2768; domains: chitin-bd_II (aa32-90,96-163,236-300,325-374), Sushi_SCR_CCP (aa475-534,733-792,946-1004,1299-1359,1669-1727), galectin (aa802-928,1341-1493,1942-2089), FA58C_3 (aa1134-1289,1493-1655,2091-2244); SSP (aa1-20); pI 6.0; IDR (6.8%; aa392-432,661-738,1509-1533,1672-1680) | K1QJK2_CRAGI  (aa1-458)  K1PRD3_CRAGI  (aa1235-2010) | 28.9  24.2 | 1.9e-16  3.8e-11 | 52  45  -  - | 547  360  -  - | 0.044  0.031  -  - | I_A_  I_B_  S_A_  S_B_ |
| **H_sp_Tri_120377_c0_g1_i1^3^** | Uncharacterized; domains: Sushi_SCR_CCP (aa30-88,235-295), Con_A_like (aa311-436), FA58C (aa453-607); shares several peptides with Tri_120379, idb_13824 and idb_2772; 10.3% V; pI 5.6 |  |  |  | 3  2  -  - | 12  2  -  - | 0.001  <0.001  -  - | I_A_  I_B_  S_A_  S_B_ |
| **H_sp_Tri_120379_c0_g1_i1^3^** | Uncharacterized; shares many peptides with idb_2772, idb_2768, CLC_3466, Tri_120377, and 1 with idb_13824; domains: multiple Sushi_SCR_CCP, Galactose-bd, FA58C, galectin_CRD, FTP1; 10.2% T; pI 5.6¸ IDR (8.1%; aa1-8,77-164,373-422,792-800,1666-1693,1753-1760) |  |  |  | 18  7  -  - | 102  22  -  - | 0.006  0.001  -  - | I_A_  I_B_  S_A_  S_B_ |
| **H_sp_CLC_39_c0_g1_i1^3^**  (aa1-186) | Similar to uncharacterized protein 3; SSP (aa1-21); 11.9% A, 11.3% L, 13.7% P; pI 10.7; IDR (32.7%; aa22-77); aa26-52 repeats [GPXPXAXLR]_3_ | UP3_HALAI**^4^**  (aa1-169) | 74.3 | 3.1e-34 | 15  12  5  5 | 328  239  19  16 | 0.664  0.749  0.131  0.063 | I_A_  I_B_  S_A_  S_B_ |
| **UP3_HALAI^3^**  (P86737) | Uncharacterized protein 3; SSP (aa1-21), 10.1% A, 11.5% L, 11.5% P; pI 10.0; shares 1 peptide with CLC_39 |  |  |  | 2  2  - | 45  28  -  - | 0.029  0.019  -  - | I_A_  I_B_  S_A_  S_B_ |
| **H_sp_CLC_4_c0_g1_i1^3^**  (aa20-67)  **H_sp_Tri_11338_c0_g1_i1** | Similar to cartilage matrix protein; aa1-165 similar to ML7A11**^4^**; SSP (aa1-18); 20.4% N, 11.2% D, 23.7% G; pI 5.3; (peptides in aa35-68; the rest is similar to DGRP; IDR (84.2%; aa26-46,62-170); repeats (Fig. S2F) | K1Q2S1_CRAGI  (aa344-391) | 52.1 | 1.3e-6 | 3  3  -  - | 95  83  -  - | 1.061  1.033  -  - | I_A_  I_B_  S_A_  S_B_ |
| **H_sp_CLC_413_c0_g1_i1**  (aa51-421)  Comp19203_c0_seq1_1  Comp104237_c0_seq2_2 | Uncharacterized; domain: chitinase_II (aa54-402); pI 8.6; IDR (6.1%; aa1-7,434-455) | V4ANQ5_LOTGI  Lotgidraft_227027  (aa18-381) | 46.2 | 3.1e-73 | 2  7  -  - | 6  31  -  - | 0.001  0.006  -  - | I_A_  I_B_  S_A_  S_B_ |
| **H_sp_CLC_554_c0_g1_i1^3^** | Uncharacterized; domains: C-type_lectin-like (aa29-135,623-753), Ig-like (aa764-861); 12.5%S, 15.6% T; pI 6.7; IDR (58.5%; aa168-640,751-776,886-901) |  |  |  | 10  11  3  - | 77  69  21  - | 0.009  0.012  0.015  - | I_A_  I_B_  S_A_  S_B_ |
| **H_sp_CLC_577_c0_g1_i1**  (aa34-257) | Uncharacterized; TM (aa71-93,103-125,132-154,186-208); pI 8.4; IDR (25.1%; aa1-4,27-58,215-241,282-291) | V3ZP86_LOTGI  Lotgidraft_232674  (aa63-288) | 30.9 | 1.4e-25 | 2  2  -  - | 4  10  -  - | 0.003  0.004  -  - | I_A_  I_B_  S_A_  S_B_ |
| **H_sp_Tri_33510_c0_g1_i1^3^**  **H_sp_CLC_62_c0_g1_i1** | Uncharacterized; SSP (aa1-21); 18.4% Q, 13.1% G, 15.0% P, pI 11.8; IDP; repeats (Fig. S2G) |  |  |  | 5  4  6  8 | 59  30  28  32 | 0.040  0.020  0.156  0.228 | I_A_  I_B_  S_A_  S_B_ |
| **H_sp_CLC_620_c0_g1_i1^3^**  (aa43-301)  **H_sp_idb_44242_c0_g1_i1**  Comp427051_c0_seq1_4 | Uncharacterized; SSP (aa1-22); TM (aa271-293); 10.2% S; pI 4.8; IDR (43.1%; aa66-84,311-350,362-520) | V4A4K4_LOTGI  Lotgidraft_234029  (aa32-300) | 26.1 | 7.2e-8 | 5  5  -  - | 32  39  -  - | 0.019  0.032  -  - | I_A_  I_B_  S_A_  S_B_ |
| **H_sp_CLC_699_c0_g1_i1**  (aa1-363)  **H_sp_idb_17813_c0_g1_i1**  Comp89781_c0_seq1_3 | Fructose-bisphosphate aldolase; domain: Aldolase_TIM (aa3-347); 12.1% A; pI 6.6; IDR (7.4%; aa1-11,348-363); see also comp23247_c0_seq1_2 | K7WKX6  _HALRU  (aa1-363) | 93.4 | 4.7e-147 | 4  4  -  - | 9  20  -  - | 0.001  0.002  -  - | I_A_  I_B_  S_A_  S_B_ |
| **H_sp_CLC_73_c0_g1_i1^3^**  (aa1-655) | Similar to Collagen alpha 1 (V) chain; domain: collagen_triple_helix (aa13-247); 15.9% G, 15.7% P, 16.6% S; pI 11.4; IDR (62.3%; aa248-655); shares several peptides with idb_17035; Fig. S2H | A0A068WQE8_  ECHGR  (aa541-1195) | 30.7 | 2.8e-16 | 26  24  4  2 | 454  291  11  2 | 0.091  0.051  0.010  0.002 | I_A_  I_B_  S_A_  S_B_ |
| **H_sp_idb_17035_c0_g1_i1^3^** | Uncharacterized; shares several peptides with Tri_121458 and CLC_73; 10.5% A, 9.3% R, 11.0% P, 32.5% S; pI 13.0; IDP¸ Fig. S2H |  |  |  | 17  18  8  6 | 506  367  12  11 | 0.405  0.378  0.058  0.024 | I_A_  I_B_  S_A_  S_B_ |
| **H_sp_Tri_121458_c0_g1_i1^3^** | Uncharacterized; 14.6% A, 9.9% R, 11.8% G, 18.9% S; pI 12.3; IDP; shares several peptides with idb_17035; Fig. S2H |  |  |  | 4  5  1  2 | 134  80  1  5 | 0.443  0.260  0.001  0.012 | I_A_  I_B_  S_A_  S_B_ |
| **H_sp_CLC_8154_c0_g1_i1**  (aa1-144) | Uncharacterized; domain: Glu_COpept2_homo (aa1-106); TM (aa7-29); IDR (19.9%; aa34-47,133-146) | W4ZKL5_STRPU  (aa34-185) | 33.6 | 6.9e-9 | 3  2  -  - | 39  7  -  - | 0.036  0.006  -  - | I_A_  I_B_  S_A_  S_B_ |
| **H_sp_CLC_866_c0_g1_i1^3^**  (aa169-300) | Uncharacterized/similar to Col17A1; domain: collagen_triple_helix (aa170-303); TM (aa16-38); 18.8% G; pI 5.5; IDR (3.3%; aa1-10) | G1KDN1_  ANOCA  (aa582-718) | 56.5 | 6.6e-16 | 2  2  -  - | 27  11  -  - | 0.003  0.002  -  - | I_A_  I_B_  S_A_  S_B_ |
| **Comp52297_c0_seq1_2^3^**  (aa10-221)  **H_sp_idb_10314_c0_g1_i1** | Similar to Sushi, von Willebrand factor type A, EGF and pentraxin domain-containing protein 1; domain: ConA-like (aa42-224); 11.1% N; pI 9.1; IDR (12.2%; aa239-270) | K1R3V2_CRAGI**^4^**  (aa2487-2688) | 34.3 | 1.1e-12 | 8  6  -  - | 121  106  -  - | 0.081  0.100  -  - | I_A_  I_B_  S_A_  S_B_ |
| **H_sp_idb_10705_c0_g1_i1^3^**  (aa358-714)  **H_sp_Tri_36495_c0_g1_i1** | Uncharacterized; domains: C-type_lectin (aa49-147,181-287); TM (aa21-43); pI 5.7; IDR (4.5%; aa308-339) | M4AQ88_XIPMA  (aa651-928)  V3ZN30_LOTGI  Lotgidraft_216450  (aa21-377) | 30.8  54.6 | 7.8e-19  1.2e-81 | 26  26  -  - | 352  385  -  - | 0.089  0.190  -  - | I_A_  I_B_  S_A_  S_B_ |
| **H_sp_idb_12172_c0_g1_i1** | Uncharacterized; 16.4% G, 9.1% M, 9.6% P, 13.8% T; IDEP; pI 4.9; shares peptides with idb_12176 |  |  |  | -  6  -  5 | -  73  -  16 | -  0.069  -  0.057 | I_A_  I_B_  S_A_  S_B_ |
| **H_sp_idb_12176_c0_g1_i1^3^** | Uncharacterized; 10.9% E, 34.3% T; pI 4.4; shares several peptides with idb_12172 and Tri_18120; IDP |  |  |  | 1  3  1  3 | 6  33  3  19 | 0.002  0.041  0.005  0.120 | I_A_  I_B_  S_A_  S_B_ |
| **H_sp_Tri_18120_c0_g1_i1^3^**  (aa1-413) | Uncharacterized; 12.7% G, 10.6% P, 16.1% T; pI 5.0; IDP; shares some peptides with Tri_18123; peptides to aa493! mult. GGM-like repeats | X8J778_9HOMO  (aa96-522) | 27.9 | 1.7e-5 | 7  -  7  - | 85  -  37  - | 0.049  -  0.245  - | I_A_  I_B_  S_A_  S_B_ |
| **H_sp_idb_13357_c0_g1_i1^3^**  (aa561-780)  **H_sp_idb_13358_c0_g1_i1** | Similar to shell matrix protein; domains: chitin-bd_II (aa473-526,535-592); 13.3% S, 26.4% T; pI 7.3; IDP (probably not the predicted domains!); peptides from aa544-780! | PSM_MYTCA**^4^**  (aa71-290) | 30.2 | 2.8e-14 | 3  3  -  - | 29  21  -  - | 0.004  0.007  -  - | I_A_  I_B_  S_A_  S_B_ |
| **H_sp_idb_13824_c0_g1_i1^3^**  Comp89292_c0_seq1_4 | Uncharacterized; domains: Sushi_SCR_CCP (aa52-110,257-317,603-664,801-859), galectin_CRD (aa329-458,674-798,872-997); 10.0% T; pI 6.9; shares some peptides with Tri_120377 |  |  |  | 16  12  -  - | 193  75  -  - | 0.018  0.005  -  - | I_A_  I_B_  S_A_  S_B_ |
| **H_sp_idb_16142_c0_g1_i1**  (aa1-298)  (aa315-534)  Comp94779_c0_seq1_5 | Uncharacterized/putative Na+/H+ exchange regulatory cofactor nhe-rf1 (fragment); domains: PDZ (aa4-93,168-264,300-398,432-526); 9.5% G, 11.3% V; pI 4.7; IDR (30.4%; aa96-178,271-295,392-438,539-550); 1 peptide not in match! | A0A023FET9  _9ACAR  (aa26-323)  D2HD25_AILME  (aa2-216) | 35.2  42.9 | 1.4e-26  4.9e-25 | 6  4  -  - | 22  17  -  - | 0.009  0.005  -  - | I_A_  I_B_  S_A_  S_B_ |
| **H_sp_idb_16155_c0_g1_i1**  (aa30-219) | Uncharacterized; SSP (aa1-22); 11.0% N, 12.0% P; pI 8.8; IDP | V4A2P3_LOTGI**^4^**  Lotgidraft_166451  (aa6-165) | 44.2 | 1.4e-16 | 3  3  -  - | 21  13  -  - | 0.007  0.005  -  - | I_A_  I_B_  S_A_  S_B_ |
| **H_sp_idb_16318_c0_g1_i1^3^** | Uncharacterized; 11.2% P, 10.0% V, pI 9.5 |  |  |  | 3  3  -  - | 12  11  -  - | 0.002  0.004  -  - | I_A_  I_B_  S_A_  S_B_ |
| **H_sp_idb_16846_c0_g1_i1**  (aa1-198) | Similar to ependymin-related protein 1; domain: ependymin (aa70-198); SSP (aa1-17); pI 5.6; 9.9% T; shares several peptides with Tri_31897 | EPDR1_HALAI**^4^**  (aa1-198) | 74.7 | 2.9e-70 | 2  2  -  - | 32  20  -  - | 0.048  0.037  -  - | I_A_  I_B_  S_A_  S_B_ |
| **H_sp_idb_17014_c0_g1_i1^3^** | Uncharacterized; SSP (aa1-20); 11.7% A, 10.1% Q, 11.4% P, 17.8% T, pI 5.4; IDP |  |  |  | 14  13  11  7 | 269  208  69  54 | 0.181  0.178  1.109  1.150 | I_A_  I_B_  S_A_  S_B_ |
| **H_sp_idb_17053_c0_g1_i1**  (aa1-420)  **H_sp_Tri_48536_c0_g1_i1** | Similar to zinc transporter ZIP14; domain: zinc/iron_permease (ZIP; aa155-393); SSP (aa1-16), TM (aa157-176,189-211,226-243); 10.8% L, 9.3% S; pI 5.1; IDR (27.5%; aa57-82,136-152,260-336) | A0A0F8CRH9  _LARCR  (aa59-466) | 27.2 | 6.3e-28 | 2  2  -  - | 30  31  -  - | 0.006  0.011  -  - | I_A_  I_B_  S_A_  S_B_ |
| **H_sp_idb_1745_c0_g1_i1^3^**  (aa35-1929)  Comp170175_c0_seq1_6  Comp189149_c0_seq1_5 | Similar to IgGFc-binding protein; domains: multiple (23) chitin_bd; SSP (aa1-19); pI 5.6; IDR (9.7%; aa661-693,762-773,836-846,930-940,961-1001,1064-1078,1142-1186,1334-1343,170-1711) | K1PRD3_CRAGI  (aa280-2199) | 28.1 | 8.7e-55 | 81  66  -  - | 814  566  -  - | 0.068  0.050  -  - | I_A_  I_B_  S_A_  S_B_ |
| **H_sp_idb_17762_c0_g1_i1**  (aa14-556)  **Comp97441_c0_seq3_2** | Uncharacterized; domains: Prot_inhib_I29 (aa253-309), Peptidase_C1A_C (aa3-545); SSP (aa1-32); pI 5.6; IDR (1.9%; aa322-331) | A0A0B7B2Q2  _9EUPU  (aa3-545) | 56.2 | 8.4e-143 | 5  5  -  - | 25  22  -  - | 0.007  0.003  -  - | I_A_  I_B_  S_A_  S_B_ |
| **H_sp_idb_18243_c0_g1_i1**  (aa30-326) | Uncharacterized; domain: VWD (aa69-301); SSP (aa1-29); 9.8% S, 9.8% T; pI 5.3 | V3ZQD5_LOTGI  Lotgidraft_232647  (aa22-318) | 26.1 | 3.3e-15 | 2  3  -  - | 4  6  -  - | 0.001  0.001  -  - | I_A_  I_B_  S_A_  S_B_ |
| **H_sp_idb_18575_c0_g1_i1^3^**  (aa9-461) | Similar to EGF-like domain-containing protein 2; domains: EGF_3 (aa58-93), ZP_2 (100-359); SPP (aa1-19), TM (aa410-432); 10.3% T; pI 5.2; IDR (14.7%; aa350-404,439-451) | ELDP2_LOTGI**^4^**  (aa10-485) | 33.1 | 2.6e-43 | 15  13  3  2 | 199  179  11  5 | 0.110  0.090  0.009  0.003 | I_A_  I_B_  S_A_  S_B_ |
| **H_sp_idb_18707_c0_g1_i1**  (aa289-583) | Similar to metalloendopeptidase; domain: MetalloPept_cat/ peptidase_M12A (aa336-533); SSP (aa1-18); 9.4% R; pI 9.8; IDR (15.1%; aa110-151,265-280,623-657,663-688, 833-839); alignment contains only part of the peptides! | A0A0B6ZEN0  _9EUPU  (aa70-382) | 41.8 | 8.5e-38 | 15  13  -  - | 284  276  -  - | 0.055  0.057  -  - | I_A_  I_B_  S_A_  S_B_ |
| **H_sp_idb_18725_c0_g1_i1^3^**  (aa2-503) | Uncharacterized/similar to mucin; 10.2% A, 10.2% Q, 12.3% S, 15.0% T; pI 7.5; IDP; repeats (Fig. S2I) | MUC22_HUMAN  (aa1155-1655) | 28.4 | 2.4e-12 | 8  7  7  6 | 241  215  66  46 | 0.838  1.379  2.255  0.671 | I_A_  I_B_  S_A_  S_B_ |
| **H_sp_idb_19448_c0_g1_i1**  (aa24-543) | Similar to carboxylic ester hydrolase (BCHE); domain: CarbesteraseB (aa22-544); SSP (aa1-21); pI 5.5; IDR (2.6%; aa551-565) | H0V4F8_CAVPO  (aa34-447) | 43.0 | 1.5e-88 | 4  2  -  - | 10  4  -  - | 0.001  <0.001  -  - | I_A_  I_B_  S_A_  S_B_ |
| **H_sp_idb_19531_c0_g1_i1** | Uncharacterized; 15.4% S, 14.8% T; pI 6.7; IDR (34.8%; aa186-401,626-648) |  |  |  | 3  2  4  - | 14  12  11  - | 0.001  0.002  0.031  - | I_A_  I_B_  S_A_  S_B_ |
| **H_sp_idb_19681_c0_g1_i1^3^**  (aa19-216) | Similar to ependymin-related protein 1; domain: ependymin (aa89-216); SSP (aa1-35); pI 8.2; 10.5% T | EPDR1_HALAI**^4^**  (aa1-198) | 66.7 | 7.7e-63 | 12  12  2  - | 332  298  4  - | 5.301  4.885  0.015  - | I_A_  I_B_  S_A_  S_B_ |
| **H_sp_idb_19738_c0_g1_i1^3^**  (aa1-447)  Comp83487_c0_seq4_4  Comp68763_c0_seq1_6 | β-tubulin; domains: Tubulin_FtsZ_GTPase (aa1-254), Tub_FtsZ_C (aa244-429); pI 4.7; IDR (7.4%; aa415-447); 1 group with comp109271_c0_seq1_3, idb_19739, TBB_HALDI and other tubulins | W4XHC0_STRPU  (aa1-447) | 98.9 | 1.7e-187 | 10  -  -  - | 86  -  -  - | 0.011  -  -  - | I_A_  I_B_  S_A_  S_B_ |
| **H_sp_idb_23118_c0_g1_i1**  (aa1-444)  Comp109271_c0_seq1_3**^3^** | Uncharacterized/beta-tubulin; domains: Tubulin_FtsZ_GTPase (aa1-243), Tub_FtsZ_C (aa244-429); pI 4.8; IDR (7.0%; aa415-445); shares most peptides with other tubulins | V4APA2_LOTGI**^4^**  Lotgidraft_202077  (aa1-444) | 98.2 | 2.3e-191 | -  9  -  - | -  72  -  - | -  0.012  -  - | I_A_  I_B_  S_A_  S_B_ |
| **H_sp_idb_54688_c0_g1_i1**  (aa1-162) | Beta-tubulin; domain: Tubulin/FtsZ_2-layer-sand-dom (aa1-100); IDR (19.6%; aa132-163); shares 1 peptide with idb_19738 | V3ZVA4_LOTGI  Lotgidraft_220547  (aa284-445) | 96.3 | 3.7e-64 | 1  -  -  - | 2  -  -  - | 0.002  -  -  - | I_A_  I_B_  S_A_  S_B_ |
| **H_sp_idb_19812_c0_g1_i1^3^**  (aa170-812)  **H_sp_idb_19814_c0_g1_i1**  Comp87710_c0_seq1_1  Comp71075_c0_seq4_5  Comp103815_c0_seq4_6 | Uncharacterized; domains: haem_peroxidase (aa164-744), TSP1 (aa747-789); SSP (aa1-23); 10.4% S; pI 9.1; IDR (8.8%; aa45-64,85-108,205-211,268-274,376-384,808-814); peptides from aa109-8590 | V4C5T3_LOTGI  Lotgidraft_115464  (aa2-658) | 44.5 | 1.1e-86 | 9  9  -  - | 69  61  -  - | 0.004  0.005  -  - | I_A_  I_B_  S_A_  S_B_ |
| **H_sp_idb_20008_c0_g1_i1^3^**  (aa2-593)  Comp45250_c0_seq1_3  Comp401928_c0_seq1_5 | Uncharacterized; SSP (aa1-20); 15.8% Q, 12.2% G, 16.6% P; pI 10.3; shares several peptides with idb_20009; IDP; repeats (Fig. S2J) | B4L3L7_DROMO  (aa1105-1801) | 30.5 | 2.7e-05 | 19  19  12  7 | 659  500  52  29 | 1.574  1.182  0.629  0.207 | I_A_  I_B_  S_A_  S_B_ |
| **H_sp_idb_20009_c0_g1_i1** | Uncharacterized; SSP (aa1-20); 38.1% Q, 10.5% P; pI 11.8; IDP; shares several peptides with idb_20008 |  |  |  | 1  1  1  1 | 82  60  2  1 | 0.042  0.040  0.002  0.001 | I_A_  I_B_  S_A_  S_B_ |
| **H_sp_idb_20328_c0_g1_i1**  (aa8-655)  Comp315920_c0_seq1_5 | Uncharacterized/similar to metalloprotease; domains: peptidase_M4 (aa299-452), peptidase_M4_C (aa451-595); SSP (aa1-20); pI 5.6; IDR (13.8%; aa31-51,225-298) | A0A0L8GFI5  _OCTBM  (aa14-572) | 33.8 | 6.7e-56 | 8  10  -  - | 65  128  -  - | 0.006  0.024  -  - | I_A_  I_B_  S_A_  S_B_ |
| **H_sp_idb_20544_c0_g1_i1**  (aa15-428)  Comp102123_c2_seq1_1 | Uncharacterized/similar to alpha-N-acetylgalactosaminidase; domains: Glycoside_hydrolase_SF (aa35-327), Glyco_hydro_b (aa228-418);SSP (aa1-35); pI 4.7 | A0A0L8H4W2  _OCTBM  (aa1-411) | 60.8 | 1.3e-118 | 2  2  -  - | 39  40  -  - | 0.007  0.007  -  - | I_A_  I_B_  S_A_  S_B_ |
| **H_sp_idb_20754_c0_g1_i1**  (aa118-1024)  Comp105195_c0_seq12_2  Comp105194_c1_seq1_2 | Uncharacterized/similar to aminopeptidase N; domains: Peptidase_M1_N (aa138-531), ERAP1-like_C (aa685-1003); TM (aa35-57); pI 5.3; IDR (9.7%; aa1-32,64-131) | A0A0B7AVU0  _9EUPU  (aa14-931) | 53.9 | 0e0 | 22  12  -  - | 160  68  -  - | 0.013  0.009  -  - | I_A_  I_B_  S_A_  S_B_ |
| **H_sp_idb_20988_c0_g1_i1^3^**  (aa1-467)  Comp406429_c0_seq1_3 | Similar to shell protein 4/aplysianin-A; domain: amine_oxidase (aa2-447)¸ pI 9.3; see also Tri_25106 | A0A0G2YN89  _MYTCO**^4^**  (aa72-537) | 32.9 | 1.5e-50 | 32  31  -  - | 878  786  -  - | 0.808  0.708  -  - | I_A_  I_B_  S_A_  S_B_ |
| **H_sp_idb_22001_c0_g1_i1^3^**  (aa32-229) | Similar to ependymin-related protein 1; domain: ependymin (aa102-228); 9,1% G, 10.4% T; pI 7.5 | EPDR1_HALAI**^4^**  (aa1-199) | 75.9 | 4.9e-71 | 9  13  -  - | 182  538  -  - | 0.481  2.796  -  - | I_A_  I_B_  S_A_  S_B_ |
| **H_sp_idb_22086_c0_g1_i1^3^**  (aa124-557)  H_sp_idb_22087_c0_g1_i1 | Uncharacterized; TM (aa20-42); 30.6% D; pI 3.3; IDP; similar to other acidic shell proteins; also peptides in aa1-124!  Shares 2 peptides with idb_42421; repeats (Fig S2K) |  |  |  | 7  5  2  2 | 87  63  2  2 | 0.093  0.056  0.006  0.007 | I_A_  I_B_  S_A_  S_B_ |
| **H_sp_idb_42421_c0_g1_i1^3^**  (aa2-230) | Uncharacterized; shares 2 peptides with idb_22086; 28.1% D; pI 3.3; IDP; repeats (Fig S2K) |  |  |  | 3  3  -  - | 11  3  -  - | 0.003  <0.001  -  - | I_A_  I_B_  S_A_  S_B_ |
| **H_sp_idb_23329_c0_g1_i1^3^**  (aa140-649) | Uncharacterized; SSP (aa1-16); 24.0%A, 10.0% P, 20.2%T, pI 3.9; IDP; unique peptides in aa18-100; aa488-649 similar to Tri_117880/idb_23862; 2.3 ~100aa repeats in aa265-482, 8.7 ~10aa repeats in aa528-615) | H3E3Y1_PRIPA  (aa100-614) | 43.3 | 5.8e-20 | 6  6  5  4 | 78  48  114  119 | 0.112  0.093  4.447  5.097 | I_A_  I_B_  S_A_  S_B_ |
| **H_sp_Tri_117880_c0_g1_i1^3^**  (aa1-687) | Uncharacterized; 19.5% A, 12.1% S, 20.1% T; pI 4.3; domain: PT_repeat (aa651-681); N-term (aa1-53): 5 repeats ADTTAAPTT[I,A,E]A; IDP; shares most peptides with idb_23862 | E9AEM9_LEIMA  (aa5712-6408) | 20.5 | 4.9e-13 | -  -  11  10 | -  -  175  141 | -  -  10.153  9.944 | I_A_  I_B_  S_A_  S_B_ |
| **H_sp_idb_23862_c0_g1_i1^3^**  (aa5-658) | Uncharacterized; 17.9% A, 10.8% G, 12.7% S, 19.7% T; pI 4.4; IDP | T1R7Q9_HUMAN  (aa1338-1990) | 28.3 | 1.1e-12 | 10  12  -  - | 145  178  -  - | 0.477  1.190  -  - | I_A_  I_B_  S_A_  S_B_ |
| **H_sp_idb_23519_c0_g1_i1^3^**  (aa1-1668)  Comp94729_c0_seq3_2  Comp110183_c0_seq1_4 | Uncharacterized; 12.5% G, 14.1% S, 40.6% T; pI 4.4; IDP; shares several peptides with Tri_4114 | A0A090CG02  _PODAN  (aa1589-3167) | 40.7 | 8.3e-74 | 16  15  -  - | 43  50  -  - | 0.007  0.012  -  - | I_A_  I_B_  S_A_  S_B_ |
| **H_sp_Tri_4114_c0_g1_i1^3^**  Comp94729_c0_seq1_2 | Uncharacterized; 10% S, 25% T, 10.4% V; pI 4.3; IDP; shares most peptides with idb_23519 |  |  |  | 1  2  -  - | 1  4  -  - | <0.001  0.001  -  - | I_A_  I_B_  S_A_  S_B_ |
| **H_sp_idb_24399_c0_g1_i1**  (aa25-639) | Uncharacterized; domains: multiple IG/FN3; SSP (aa1-20); IDR (8.3%; aa523-548,633-648,738-766,785-800,844-857,1117-1123); pI 9.3; shares most peptides with Tri_6626 | V4AXZ7_LOTGI  Lotgidraft_230852  (aa144-760) | 33.1 | 3.0e-57 | 13  -  -  - | 44  -  -  - | 0.003  -  -  - | I_A_  I_B_  S_A_  S_B_ |
| **H_sp_Tri_6626_c0_g1_i1**  (aa25-656)  Comp101634_c0_seq7_3  Comp101634_c0_seq8_3  Comp101634_c0_seq10_3 | Uncharacterized; domains: multiple IG/FN3; IDR (5.9%; aa528-545,630-653,899-910); shares most peptides with idb_24399 | V4AXZ7_LOTGI  Lotgidraft_230852  (aa144-777) | 32.6 | 1.1e-56 | 1  10  -  - | 1  46  -  - | <0.001  0.003  -  - | I_A_  I_B_  S_A_  S_B_ |
| **H_sp_Tri_31898_c0_g1_i1^3^**  (aa1-198)  **H_sp_idb_24481_c0_g1_i1** | Similar to to ependymin-related protein 1; domain: ependymin (aa71-198); SSP (aa1-17); 9.6% T, 11.1% V; pI 7.5; shares most peptides with CLC_160 | EPDR1_HALAI**^4^**  (aa1-198) | 75.3 | 8.5e-72 | 22  9  7  4 | 1128  553  29  7 | 7.763  3.133  0.197  0.032 | I_A_  I_B_  S_A_  S_B_ |
| **H_sp_idb_25746_c0_g1_i1^3^**  (aa88-662)  Comp44198_c0_seq1_4  Comp39177_c0_seq1_3 | Uncharacterized/peroxidasin-like; domain: peroxidase_3 (aa79-664); SSP (aa1-22); pI 9.0; IDR (3.1%; aa1-11,25-34); peptides from aa35-659! | V4C5T3_LOTGI  Lotgidraft_115464  (aa2-563) | 47.2 | 1.7e-110 | 60  57  12  7 | 2697  2196  25  25 | 2.808  2.929  0.016  0.020 | I_A_  I_B_  S_A_  S_B_ |
| **H_sp_idb_26030_c0_g1_i1^3^**  (aa21-693)  Comp104856_c0_seq3_5 | Similar to zinc transporter; domain: zinc/iron_permease (ZIP; aa395-687); SSP (aa1-23); multiple TM (6; in C-term half); pI 5.3; IDR (18.0%; aa20-38,171-201,292-332,504-540) | E6ZJ32_DICLA  (aa21-680) | 31.4 | 5.5e-48 | 5  4  2  - | 41  30  3  - | 0.004  0.004  0.001  - | I_A_  I_B_  S_A_  S_B_ |
| **H_sp_idb_26064_c0_g1_i1**  (aa56-222)  Comp81814_c0_seq1_2 | Similar to leukocyte cell derived chemotaxin 1-like protein; SSP (aa1-48) or TM (aa26-48); 11.5% A; pI 7.3; IDR (4.9%; aa1-11); if SSP: pI 6.3; no IDR also see Tri_23898 and Tri_53798 | D5FW85_HALDI  (aa16-186) | 28.9 | 2.7e-7 | 7  2  -  - | 66  18  -  - | 0.039  0.010  -  - | I_A_  I_B_  S_A_  S_B_ |
| **H_sp_idb_26322_c0_g1_i1** | Uncharacterized; SSP (aa1-25); 10.3% P, 10.9% S; pI 11.8; IDP |  |  |  | 7  6  -  - | 79  63  -  - | 0.007  0.007  -  - | I_A_  I_B_  S_A_  S_B_ |
| **H_sp_idb_26484_c0_g1_i1^3^**  (aa1-199)  Comp85406_c1_seq2_1  Comp85406_c0_seq1_2 | Similar to peptidyl-prolyl cis-trans isomerase; domain: cyclophilin_type_PPI (aa30-187); SSP (aa1-20); 13.9% G, 10.0% T; pI 4.5; IDR (14.4%; aa174-200) | A0A016S171_  9BILA  (aa1-197) | 67.0 | 3.4e-57 | 6  5  -  - | 89  66  -  - | 0.032  0.032  -  - | I_A_  I_B_  S_A_  S_B_ |
| **H_sp_idb_26568_c0_g1_i1^3^**  (aa226-1023)  **H_sp_idb_26567_c0_g1_i1**  Comp3500_c0_seq1_5 | Uncharacterized; SSP (aa1-20); 12.8% N, 14.2% Q, 10.3% P, 13.4% S; pI 9.7; IDP; repeats (Fig. S2N) | V4CGH1_LOTGI  Lotgidraft_172708  (aa9-816) | 26.1 | 7.3e-11 | 39  37  18  12 | 903  609  91  37 | 0.388  0.252  0.439  0.070 | I_A_  I_B_  S_A_  S_B_ |
| **H_sp_idb_26836_c0_g1_i1^3^** | Uncharacterized; 13.2% S, 12.5% T; pI 5.1; IDP; repeats (Fig. S2O) |  |  |  | 3  3  3  3 | 50  52  19  12 | 0.230  0.333  0.936  0.335 | I_A_  I_B_  S_A_  S_B_ |
| **H_sp_idb_27355_c0_g1_i1^3^**  (aa13-560)  Comp30184_c0_seq1_4 | Uncharacterized; SSP (aa1-41); 9.8% A, 10.7% Q, 10.9% S, 10.2% T; pI 6.9; IDP; repeats (Fig. S2P) | Q2UQ79_ASPOR  (aa455-1013) | 26.2 | 2.0e-5 | 27  23  17  10 | 706  621  126  113 | 2.913  5.541  1.929  1.813 | I_A_  I_B_  S_A_  S_B_ |
| **H_sp_idb_27788_c0_g1_i1**  (aa26-233)  UP6_HALAI | Uncharacterized protein 6; see also CLC_28060; TM (aa17-39); 10.5% A, 10.5% L, 11.8% T; pI 9.4 | UP6_HALAI**^4^**  (aa1-208) | 82.2 | 1.6e-76 | 8  5  2  - | 94  74  7  - | 0.147  0.096  0.028  - | I_A_  I_B_  S_A_  S_B_ |
| **H_sp_idb_27864_c0_g1_i1** | Uncharacterized; 20.8% A, 13.4% Q, 13.4% S, 18.7% T; pI 4.1; IDP; repeats (Fig. S2Q) |  |  |  | 2  2  2  2 | 35  19  62  77 | 0.565  0.264  9.466  16.527 | I_A_  I_B_  S_A_  S_B_ |
| **H_sp_idb_27866_c0_g1_i1^3^**  (aa23-358) | Uncharacterized; 13.4% A, 9.9% G, 16.5% S, 19.6% T; pI 4.5; IDP; repeats (Fig. S2R) | A0A0G2KUY1  _DANRE  (aa317-659) | 38.3 | 3.1e-9 | 5  5  5  5 | 66  54  62  62 | 0.803  0.844  19.703  22.061 | I_A_  I_B_  S_A_  S_B_ |
| **H_sp_idb_27973_c0_g1_i1**  (aa1-225)  Comp99731_c0_seq1_5 | Glutathione-S-transferase; domains: Thioredoxin-like_fold (aa4-90, Glutathione-S-Trfase_C (aa92-217); pI 6.0 | B6RB03_HALDI  (aa1-225) | 85.8 | 1.4e-88 | 3  2  -  - | 4  5  -  - | 0.001  0.001  -  - | I_A_  I_B_  S_A_  S_B_ |
| **H_sp_idb_2814_c0_g1_i1^3^**  (aa233-624) | Uncharacterized; domains: [Lectin_gal-bd](http://www.ebi.ac.uk/interpro/entry/IPR000922) (aa237-310), CUB (aa320-444); SSP (aa1-19), TM (aa528-550); pI 5.1; IDR (62.7%; aa1-238,458-516,550-674) | V4A930_LOTGI**^4^**  Lotgidraft_232714  (aa377-794) | 27.4 | 6.9e-14 | 12  12  4  2 | 279  229  8  6 | 0.270  0.156  0.008  0.010 | I_A_  I_B_  S_A_  S_B_ |
| **H_sp_idb_28907_c0_g1_i1^3^**  H_sp_idb_36084_c0_g1_i1 | Uncharacterized; SSP (aa1-19); 17.2% Q, 9.8% G, 11.1% P; pI 9.8; IDP |  |  |  | 2  -  3  - | 46  -  7  - | 0.020  -  0.021  - | I_A_  I_B_  S_A_  S_B_ |
| **H_sp_idb_3074_c0_g1_i1^3^** | Uncharacterized; 14.4% S, 14.0% T; pI 7.2; domain: EGF_3 (aa53-90); SSP (aa1-21); IDP |  |  |  | 5  7  4  2 | 43  52  8  3 | 0.002  0.003  0.005  0.001 | I_A_  I_B_  S_A_  S_B_ |
| **H_sp_idb_30843_c0_g1_i1** | Uncharacterized; 15.1% G, 12.3% S 17.6% T; pI 4.6; domains: 5 chitin_bd in aa33-374); SSP (aa1-23); aa401-705: 27.9% G, 18.4% S, 30.5% T; IDR (43.7%; aa376-458,483-489,498-705) |  |  |  | 11  10  -  - | 163  81  -  - | 0.032  0.013  -  - | I_A_  I_B_  S_A_  S_B_ |
| **H_sp_idb_32090_c0_g1_i1**  (aa50-286) | Uncharacterized; domains: chitin-bd_ II (aa56-119,142-206,232-287); TM (aa20-39); 11.8% S, 18.8% T; pI 6.7; IDR (3.5%; aa43-52) | V4BP02_LOTGI  Lotgidraft_233660  (aa19-215) | 29.4 | 2.5e-9 | 5  2  4  3 | 17  8  11  10 | 0.007  0.002  0.072  0.021 | I_A_  I_B_  S_A_  S_B_ |
| **H_sp_idb_32310_c0_g1_i1^3^**  (aa24-613)  Comp56242_c0_seq1_1 | Similar to chitinase-3; domains: chitinase_II (aa21-371), chitin-bd_II (aa730-786,884-941); SSP (aa1-17); pI 8.7; IDR (31.4%; aa406-474,520-627,633-657,800-891); peptides in aa49-275 | J7F1C1_HYRCU  (aa28-611) | 43.6 | 3.6e-54 | 17  11  -  - | 202  100  -  - | 0.024  0.007  -  - | I_A_  I_B_  S_A_  S_B_ |
| **H_sp_idb_32603_c0_g1_i1^3^**  **H_sp_idb_32602_c0_g1_i1**  Comp118790_c0_seq1_1  Comp184680_c0_seq1_2  Comp205609_c0_seq1_4 | Uncharacterized; SSP (aa1-21); 14.0% Q, 13.7% P, 10.7% S; pI 6.4; IDP; repeats (Fig. S2S) |  |  |  | 18  17  18  13 | 482  438  277  114 | 1.807  2.049  6.054  3.214 | I_A_  I_B_  S_A_  S_B_ |
| **H_sp_idb_33826_c0_g1_i1**  (aa42-333)  Comp93193_c0_seq1_1 | Glyceraldehyde-3-phosphate dehydrogenase; domains: GlycerAld_3-P_DH_NAD(P)-bd (aa4-151), GlycerAld_3-P_DH_cat (aa156-313); pI 6.1; see also comp109254_ c0_seq1_3; very similar but no shared peptides! | B6RB30_HALDI  (aa1-292) | 96.2 | 4.0e-115 | 9  10  -  - | 114  115  -  - | 0.024  0.042  -  - | I_A_  I_B_  S_A_  S_B_ |
| **H_sp_idb_3423_c0_g1_i1** | Uncharacterized; 10.4% G, 10.8% S, 18.0% T; pI 4.8; IDR (36.3%; aa1-411,801-812) |  |  |  | 22  18  -  - | 193  110  -  - | 0.050  0.036  -  - | I_A_  I_B_  S_A_  S_B_ |
| **H_sp_idb_34528_c0_g1_i1^3^**  (aa213-386) | Uncharacterized protein 2; SSP (aa1-25)/TM (aa12-34); 10.6% A, 11.7% L, 18.9% P; pI 10.0; IDP; repeats: [APLAXXAAPVN]_2_ in aa 94-119, 2 [LPPGAAXX]_2_ in aa186-201 | UP2_HALAI**^4^**  (aa5-179) | 77.7 | 1.3e-23 | 7  7  -  - | 52  34  -  - | 0.070  0.039  -  - | I_A_  I_B_  S_A_  S_B_ |
| **H_sp_idb_34907_c0_g1_i1^3^**  (aa49-546)  Comp96713_c3_seq4_2  Comp96713_c3_seq1_1  B6RB63_HALDI | Protein disulfide-isomerase; domains: Thioredoxin-like_fold (aa55-180,182-279,281-531,409-531); 11.4% E, 9.2% K; pI 4.5¸ IDR (15.8%; aa1-34,495-506) | B6RB63_HALDI  (aa1-499) | 92.6 | 2.1e-167 | 14  10  -  - | 78  63  -  - | 0.138  0.027  -  - | I_A_  I_B_  S_A_  S_B_ |
| **H_sp_idb_35506_c0_g1_i1^3^**  (aa1-215)  Comp97330_c0_seq1_5  B3TK24_HALDV | Glutathione-S-transferase (mu); domains: Thioredoxin-like_fold (aa1-84), GST_C (aa99-190); 10.7% L; pI 6.2 | B3TK24_HALDV  (aa1-215) | 82.3 | 2.0e-79 | 9  6  -  - | 88  82  -  - | 0.024  0.030  -  - | I_A_  I_B_  S_A_  S_B_ |
| **H_sp_idb_3591_c0_g1_i1^3^** | Uncharacterized; SSP (aa1-16); 14.8% G, 11.9% S; pI 6.6; IDP |  |  |  | 22  17  7  3 | 253  158  13  9 | 0.028  0.016  0.014  0.006 | I_A_  I_B_  S_A_  S_B_ |
| **H_sp_idb_35987_c0_g1_i1**  Comp105382_c0_seq2_1 | Uncharacterized; 10% G, 9.7% D, 10.4% S; pI 4.7 |  |  |  | 5  3  -  - | 22  9  -  - | 0.026  0.041  -  - | I_A_  I_B_  S_A_  S_B_ |
| **H_sp_idb_36583_c0_g1_i1^3^** | Uncharacterized; 14.7% G, 32.5% S, 16.4% T, pI 6.3; IDP; repeats (Fig. S2T) |  |  |  | 7  7  7  7 | 193  159  66  63 | 0.986  0.743  4.317  4.034 | I_A_  I_B_  S_A_  S_B_ |
| **H_sp_idb_38838_c0_g1_i1** | Uncharacterized; 10.3% L, 10.3% S, 10.7% T; 9.5; TM (aa15-37) |  |  |  | 4  3  -  - | 21  14  -  - | 0.015  0.010  -  - | I_A_  I_B_  S_A_  S_B_ |
| **H_sp_idb_39663_c0_g1_i1^3^** | Uncharacterized; 22.4% S, 17.4% T; pI 3.8; IDR (74.3%; aa103-397) |  |  |  | 5  4  -  - | 31  14  -  - | 0.073  0.015  -  - | I_A_  I_B_  S_A_  S_B_ |
| **H_sp_idb_40080_c0_g1_i1^3^**  (aa1-159) | Similar to ependymin-1/2; domain: ependymin (aa60-156); shares most peptides with CLC_1876; 12.5% T, 10.0% V; pI 7.8; IDR (3.8%; aa155-160) | EPDR2_HALAI**^4^**  (aa14-171)  EPDR1_HALAI**^4^** (aa11-170) | 65.4  66.2 | 6.2e-50  4.1e-48 | 5  4  -  - | 103  140  -  - | 0.556  0.592  -  - | I_A_  I_B_  S_A_  S_B_ |
| **H_sp_idb_4071_c0_g1_i1^3^**  (aa43-608)  (aa618-713) | Similar to basic proline-rich protein; Q/P-rich (15.4% Q, 9.8% G, 34.6% P), pI 5.1 and  Methionine-rich protein (9.5% C, 2.1% M); pI 6.1;  IDP; repeats (Fig. S2U) | PRP_PIG  (aa91-647)  MRP_LOTGI**^4^**  (aa162-270) | 40.2  40.9 | 3.7e-24  5.7e-7 | 12  12  2  - | 182  149  4  - | 0.091  0.133  0.005  - | I_A_  I_B_  S_A_  S_B_ |
| **H_sp_idb_42414_c0_g1_i1^3^**  (aa66-387)  Comp188351_c0_seq1_6 | Uncharacterized; domain: ConA_like (aa221-382); SSP (aa1-24); 12.0% T; pI 4.9; IDR (28.6%; aa1-111) | V4B726_LOTGI  Lotgidraft_236297  (aa45-379) | 25.4 | 2.3e-8 | 13  11  2  2 | 141  92  6  2 | 0.147  0.091  0.067  0.009 | I_A_  I_B_  S_A_  S_B_ |
| **H_sp_idb_43368_c0_g1_i1^3^**  (aa1-462) | Uncharacterized; 28.7% A, 11.9% Q, 12.3% G, 11.2% S; pI 3.3; 1 measurable peptide predicted, the second peptide is from a miss-cleavage (K-P)! IDP; repeats (Fig. S2V) | A0A0C6FK35 _9RHIZ  (aa52-546) | 29.7 | 4.4e-13 | 1  1  2  1 | 18  20  6  5 | 0.045  0.047  0.511  0.226 | I_A_  I_B_  S_A_  S_B_ |
| **H_sp_idb_44571_c0_g1_i1^3^**  (66-836)  **H_sp_idb_44572_c0_g1_i1** | Uncharacterized; domains: chitin-bd_II (aa550-612,608-676,721-779,780-838); TM (aa347-369); 11.8% S; pI 8.1; IDR (41.0%; aa1-38,49-87,114-332,670-718) | V4A7L2_LOTGI  Lotgidraft_232881  (aa14-860) | 32.8 | 2.6e-15 | 3  2  -  - | 5  4  -  - | <0.001  <0.001  -  - | I_A_  I_B_  S_A_  S_B_ |
| **H_sp_idb_44689_c0_g1_i1^3^**  (aa2-899)  **H_sp_idb_44690_c0_g1_i1** | Uncharacterized; 10.5% G, 28.3% S, 14.0% T; pI 5.1; IDP; repeats (Fig. S2W) | A0A0N0DN50  _STRSU | 32.2 | 4.8e-21 | 15  11  13  12 | 369  294  197  165 | 0.987  1.364  7.572  5.399 | I_A_  I_B_  S_A_  S_B_ |
| **H_sp_idb_47306_c0_g1_i1^3^** | Uncharacterized; SSP (aa1-19); 24.1% A, 18.1% Q, 11.4% G; pI 4.8; IDP; repeats (Fig. S2X) |  |  |  | 2  2  2  2 | 102  85  14  12 | 0.172  0.124  0.235  0.184 | I_A_  I_B_  S_A_  S_B_ |
| **H_sp_idb_50884_c0_g1_i1^3^**  (aa1-173)  UP5_HALAI  H_sp_idb_18771_c0_g1_i1 | Similar to UP5; domain: Methyltransf_FA (aa71-165); SSP (aa1-22); 9.7% V; pI 8.8 | UP5_HALAI**^4^**  (aa1-173) | 90.8 | 4.5e-78 | 6  6  3  - | 204  120  4  - | 0.632  0.342  0.046  - | I_A_  I_B_  S_A_  S_B_ |
| **H_sp_idb_51205_c0_g1_i1**  (aa1-388) | Uncharacterized; 22.2% A, 16.5% Q, 11.1% S, 20.6% T; pI 4.0; IDP; only 1 identifiable peptide predicted! Repeats (Fig. S2Y) | A0A1I8GGN1_  9PLAT  (aa2366-2765) | 35.0 | 1.2e-14 | 1  1  1  1 | 23  19  22  33 | 0.773  0.488  17.704  21.700 | I_A_  I_B_  S_A_  S_B_ |
| **H_sp_idb_51603_c0_g1_i1^3^**  Comp83604_c0_seq2_1 | Uncharacterized; SSP (aa1-21); 10.1% R, 10.1% S, pI 9.4; IDR (36.1%; aa28-36,86-119) |  |  |  | 2  4  -  - | 10  54  -  - | 0.011  0.124  -  - | I_A_  I_B_  S_A_  S_B_ |
| **H_sp_idb_5218_c0_g1_i1^3^** | Uncharacterized; pI 6.9 |  |  |  | 6  7  -  - | 64  43  -  - | 0.021  0.011  -  - | I_A_  I_B_  S_A_  S_B_ |
| **H_sp_idb_52687_c0_g1_i1^3^**  (aa35-227) | Similar to ependymin-related protein 2; domain: ependymin (aa101-227); 10.0% T; pI 8.7 | EPDR2_HALAI**^4^**  (aa11-199) | 57.6 | 9.0e-51 | 16  14  3  2 | 374  299  9  4 | 3.055  3.940  0.052  0.015 | I_A_  I_B_  S_A_  S_B_ |
| **H_sp_idb_53898_c0_g1_i1**  (aa80-341) | Uncharacterized; domains: Methyltransf_FA (aa89-189,237-337); 9.1% G, 10.3% S, 9.1% T; pI 9.8; IDR (6.5%; aa1-22) | R7UVQ9_CAPTE  (aa1-259) | 35.7 | 1.1e-31 | 3  3  -  - | 23  13  -  - | 0.006  0.002  -  - | I_A_  I_B_  S_A_  S_B_ |
| **H_sp_Tri_2952_c0_g1_i1^3^**  (aa43-180)  **Comp48289_c0_seq1_1**  **Comp48289_c0_seq2_1**  **H_sp_idb_54202_c0_g1_i1** | Uncharacterized; domain: MD-2_related_lipid_recognition (ML; aa73-175); SSP (aa1-18); pI 6.3; IDR (3.7%; aa181-187) | K1RHT2_CRAGI  (aa486-625) | 29.8 | 6.1e-10 | 5  5  -  - | 24  23  -  - | 0.005  0.009  -  - | I_A_  I_B_  S_A_  S_B_ |
| **H_sp_idb_54301_c0_g1_i1^3^** | Uncharacterized; 10.3% R, 12.1% G, pI 9.7 |  |  |  | 2  4  -  - | 4  15  -  - | 0.005  0.018  -  - | I_A_  I_B_  S_A_  S_B_ |
| **H_sp_idb_55709_c0_g1_i1^3^**  (aa14-638) | Uncharacterized; shares several peptides with Tri_138845 and idb_55710; 25.3% D, 12.3% E; pI 3.9; IDP | K9FN72_PEND2  (aa424-1048) | 27.4 | 1.1e-19 | 20  17  2  - | 237  220  4  - | 0.111  0.103  0.003  - | I_A_  I_B_  S_A_  S_B_ |
| **H_sp_Tri_138845_c0_g1_i1^3^**  (aa6-388)  **H_sp_CLC_25186_c0_g1_i1** | Similar to shell matrix protein;SSP (aa1-22)/TM (aa7-26); 23.9% D; pI 3.8; if SSP: 25.1% D; IDP; shares several peptides with idb_55709 | G9MBW9  _PINMA**^4^**  (aa3-305) | 31.1 | 2.0e-14 | 4  4  4  3 | 87  53  16  8 | 0.108  0.091  0.095  0.278 | I_A_  I_B_  S_A_  S_B_ |
| **H_sp_idb_55710_c0_g1_i1**  (aa20-190) | Uncharacterized/similar to calsequestrin; 9.9% N, 30.2% D, 15.1% E; pI 3.8; IDP; shares 3 peptides with idb_55709 | Q7T138_DANRE  (aa351-521) | 37.2 | 4.7e-6 | 1  -  2  - | 1  -  2  - | 0.002  -  0.003  - | I_A_  I_B_  S_A_  S_B_ |
| **H_sp_idb_5844_c0_g1_i1**  (aa543-1138)  Comp286881_c0_seq1_1 | Uncharacterized; domains: SEA (aa277-392), chitin-bd_II (aa752-804,1022-1074,1081-1137), Ig-like_fold (aa854-941); TM (aa233-255); 9.2% S, 10.3% T; pI 6.0; IDR (35.9%; aa35-226,400-545,801-805,931-974,1071-1091,1141-1146) | K1Q3C7_CRAGI  (aa33-636) | 32.6 | 2.2e-38 | 6  5  -  - | 14  12  -  - | 0.001  0.001  -  - | I_A_  I_B_  S_A_  S_B_ |
| **H_sp_idb_5896_c0_g1_i1^3^**  (aa135-1236) | Uncharacterized; domain: stereocilin_rel (aa426-1214); SSP (aa1-20); 10.4% D; pI 4.9; IDR (18.7%; aa21-195,231-256,904-932) | V3ZXW7_LOTGI  Lotgidraft_235120**^4^**  (aa42-1130) | 36.2 | 3.3e-116 | 33  28  -  - | 255  225  -  - | 0.038  0.035  -  - | I_A_  I_B_  S_A_  S_B_ |
| **H_sp_idb_6290_c0_g1_i1^3^**  (aa1-436)  (aa437-974)  (aa975-1602) | Similar to chitin deacetylase; domain: Glyco_hydro/deAcase_b/a-brl/NodB (aa53-323); SSP (aa1-19); pI 7.6;  IDR (16.7%)  Similar to translation initiation factor 2 (IF-2 GTPase); 12.2% P, 10.7% S; pI 9.7; IDP  Uncharacterized; domain: Glyco_hydro/deAcase_b/a-brl (aa1323-1591); 11.6% P; pI 5.2; IDR (39.7%)  IDR_tot_: 53.2% (aa364-802,809-847,853-1224) | J7FHX7_HYRCU  (aa2-443)  RANI13_STRSU  (aa313-789  K1P514_CRAGI  (aa168-801) | 41.4  23.0  27.6 | 1.3e-45  7.6e-7  1.4e-28 | 8/21/12  7/11/9  -/-/2  -/-/1 | 306  154  4  2 | 0.015  0.007  0.001  0.001 | I_A_  I_B_  S_A_  S_B_ |
| **H_sp_idb_66139_c0_g1_i1^3^**  (aa6-135) | Uncharacterized; pI 6.6 | K1R0J9_CRAGI  (aa460-584) | 40.2 | 4.4e-15 | 3  2  -  - | 16  14  -  - | 0.018  0.059  -  - | I_A_  I_B_  S_A_  S_B_ |
| **H_sp_idb_666_c0_g1_i1**  (aa18-4105) | Uncharacterized/similar to LDL receptor; domains: multiple LDrepeatLR_ classA_rpt, multiple EGF-like, multiple LDLR_classB_rpt; SSP (aa1-19); pI 5.2 | T1IUN7_STRMM  (aa145-4229) | 44.7 | 0e0 | 4  7  -  - | 7  9  -  - | <0.001  <0.001  -  - | I_A_  I_B_  S_A_  S_B_ |
| **H_sp_idb_67370_c0_g1_i1^3^**  (aa1-106) | Uncharacterized; SSP (aa1-18); 14.9% G, pI 5.3 | R7T5B9_CAPTE  (aa1-104) | 45.8 | 6.5e-16 | 2  2  -  - | 25  11  -  - | <0.001  0.003  -  - | I_A_  I_B_  S_A_  S_B_ |
| **H_sp_idb_7598_c0_g1_i1^3^**  (aa1-1942) | Uncharacterized/similar to proteophosphoglycan; domains: PT_rep (aa119-140,181-211); 12.2% A, 14.7% S, 22.4% T; pI 4.5; IDP | E9AEM9_LEIMA  (aa13878-15805) | 19.8 | 3.6e-37 | 13  16  16  12 | 92  133  114  88 | 0.042  0.149  1.984  0.731 | I_A_  I_B_  S_A_  S_B_ |
| **H_sp_idb_8012_c0_g1_i1^3^** | Uncharacterized; 15.4% Q, 12.7% G, 9.3% P, 9.8% S; pI 10.3; IDP |  |  |  | 6  5  3  - | 38  21  13  - | 0.001  0.001  0.013  - | I_A_  I_B_  S_A_  S_B_ |
| **H_sp_Tri_130845_c0_g1_i1^3^**  (aa1-573)  **H_sp_idb_813_c0_g1_i1** | Similar to carbonic anhydrase; domain: αCA_2 (aa30-313); SSP (aa1-16; 9.9% Q, 12.5% G; pI 6.0; IDR (26.7%; aa371-459,522-584) | J7QJT8_PATVU  (aa1-613) | 31.2 | 2.3e-35 | 16  16  -  - | 426  411  -  - | 0.395  0.497  -  - | I_A_  I_B_  S_A_  S_B_ |
| **H_sp_idb_982_c0_g1_i1^3^** | Uncharacterized; pI 5.3; domains multiple Sushi_SCR_CCP, galactose_bd, chitin-bd, fucolectin/tachylectin-4/pentraxin-1, galectin; SSP (aa1-22); IDR (4.4%; aa385-484,707-742) |  |  |  | 64  50  -  - | 636  356  -  - | 0.032  0.015  -  - | I_A_  I_B_  S_A_  S_B_ |
| **H_sp_Tri_10153_c0_g1_i1**  (aa1-131)  Comp95009_c0_seq1_4 | Uncharacterized; domain: ADF-H/Gelsolin-like (aa1-145); 11.3% K; pI 6.6; IDR (aa15.2%, aa129-151) | R7QGC0_CHOCR  (aa1-126) | 29.9 | 2.1e-5 | 4  5  -  - | 6  24  -  - | 0.001  0.010  -  - | I_A_  I_B_  S_A_  S_B_ |
| **H_sp_Tri_107502_c0_g1_i1^3^**  (aa44-805) | Uncharacterized; domains: NodB_homology (aa27-321,513-794); SSP (aa1-21); 10.1% T; pI 8.2; IDR (21.2%; aa330-495) | V4AMK1_LOTGI**^4^**  Lotgidraft_181237  (aa1-811) | 60.2 | 2.0e-57 | 22  23  -  - | 230  231  -  - | 0.057  0.082  -  - | I_A_  I_B_  S_A_  S_B_ |
| **H_sp_Tri_108584_c0_g1_i1^3^** | Uncharacterized; SSP (aa1-23); 15.1% P, 12.8% S; pI 10.6; IDP; repeats (Fig. S2Z) |  |  |  | 22  20  7  5 | 514  450  22  18 | 0.475  0.454  0.090  0.070 | I_A_  I_B_  S_A_  S_B_ |
| **H_sp_Tri_111351_c0_g1_i1**  (aa6-441)  Comp314509_c0_seq1_3 | Uncharacterized; SSP (aa1-18), TM (aa463-485); pI 4.9 | V4C7H9_LOTGI  Lotgidraft_228270  (aa12-467) | 20.9 | 7.6e-8 | 8  3  -  - | 22  3  -  - | 0.003  <0.001  -  - | I_A_  I_B_  S_A_  S_B_ |
| **H_sp_Tri_119193_c0_g1_i1^3^**  (aa44-175)  UP4_HALAI | Uncharacterized protein 4; TM (aa43-62); 12.0% A, 11.4% L; pI 8.7 | UP4_HALAI**^4^**  (aa1-130) | 84.1 | 4.5e-42 | 8  5  6  4 | 126  104  48  20 | 0.220  0.192  1.621  0.521 | I_A_  I_B_  S_A_  S_B_ |
| **H_sp_Tri_119238_c0_g1_i1**  (aa32-298)  Comp16504_c0_seq1_3  Comp127495_c0_seq1_5 | Similar to alpha-carbonic anhydrase; domain: Carbonic_anhydrase_a (aa40-298); SSP (aa1-31)/TM (aa12-34); pI 6.0; IDR (6.6%; aa1-12,297-304); if SSP: pI 5.4; IDR 2.9% | A0A067ZVM5  _HYRCU  (aa101-370) | 38.7 | 1.2e-33 | 9  9  -  - | 93  121  -  - | 0.023  0.046  -  - | I_A_  I_B_  S_A_  S_B_ |
| **H_sp_Tri_12080_c0_g1_i1^3^**  (aa57-1528) | Uncharacterized; SSP (aa1-20); 12.8% T, pI 10.0; IDP; peptides start at aa29! | B4L2U0_DROMO  (aa1384-2829) | 21.6 | 5.5e-10 | 10  6  3  - | 39  22  6  - | 0.001  0.001  0.001  - | I_A_  I_B_  S_A_  S_B_ |
| **H_sp_Tri_123584_c0_g1_i1**  (aa1-329)  Comp89084_c0_seq1_5 | Similar to malate dehydrogenase: domains: Lactate/malate_DH_N (aa6-152), Lactate/malate_DH_C (aa155-331); 10.2% A, 10,2% V; pI 5.9 | B6R5G9_9GAST  (aa1-328) | 69.9 | 1.1e-98 | 4  4  -  - | 32  45  -  - | 0.003  0.007  -  - | I_A_  I_B_  S_A_  S_B_ |
| **H_sp_Tri_123826_c0_g1_i1**  (aa5-966) | Uncharacterized; domains: VWA (aa313-467); Ig-like_fold (aa755-870); family: calcium-activated chloride channel protein; SSP (aa1-19), TM (aa893-915); pI 6.3; IDR (4.5%; aa708-726,919-936,964-969) | V4CB95_LOTGI**^4^**  Lotgidraft_238844  (aa5-973) | 50.6 | 1.1e-206 | 5  5  -  - | 29  27  -  - | 0.002  0.002  -  - | I_A_  I_B_  S_A_  S_B_ |
| **H_sp_Tri_127820_c0_g1_i1^3^** | Uncharacterized; SSP (aa1-31); 19.0% G, 9.8% L, 13.1% P, pI 9.5; IDR (41.8%; aa71-85,134-184) |  |  |  | 2  -  4  3 | 5  -  21  15 | <0.001  -  1.260  0.324 | I_A_  I_B_  S_A_  S_B_ |
| **H_sp_Tri_129603_c0_g1_i1^3^**  (aa1-945) | Uncharacterized; domain: Hedgehog_sig/DD-Pept_Zn-bd (aa251-429); SSP (aa1-18); pI 5.3; IDR (7.0%; aa225-253,651-688) | V4B8A6_LOTGI**^4^**  Lotgidraft_235988  (aa1-955) | 50.3 | 1.2e-97 | 21  16  -  - | 129  92  -  - | 0.010  0.009  -  - | I_A_  I_B_  S_A_  S_B_ |
| **H_sp_Tri_131427_c0_g1_i1^3^**  (aa1-376)  Comp103470_c1_seq20_6  Q5BQE5_9VEST  etc | Actin; pI 5.2; shares most peptides with other actins | Q6U1K1_HALDH  (aa1-376) | 99.7 | 1.4e-159 | 18  15  4  8 | 333  320  9  30 | 0.166  0.315  0.006  0.060 | I_A_  I_B_  S_A_  S_B_ |
| **H_sp_Tri_14507_c0_g1_i1^3^**  (aa96-590) | Uncharacterized; SSP (aa1-21); 10.7% P, 10.0% S, pI 8.7; IDR (34.8%; aa187-249,254-391); peptides start at aa44! | V4AT07_LOTGI**^4^**  Lotgidraft_173199  (aa1-610) | 28.5 | 3.7e-20 | 15  13  2  3 | 208  152  4  8 | 0.036  0.048  0.005  0.012 | I_A_  I_B_  S_A_  S_B_ |
| **H_sp_Tri_1743_c0_g1_i1^3^**  (aa1-249)  Comp89145_c0_seq2_3 | Similar to uncharacterized protein 1; SSP (aa1-17); 10.3%A, 11.2% Q, 9.5% G, 9.5% L; pI 10.2; IDR (3.9%; aa45-54) | UP1_HALAI**^4^**  (aa1-244) | 69.2 | 1.4e-61 | 27  26  9  6 | 1450  1311  61  59 | 12.267  16.679  1.022  1.784 | I_A_  I_B_  S_A_  S_B_ |
| **H_sp_Tri_2746_c0_g1_i1**  (aa1-362)  **H_sp_Tri_17455_c0_g1_i1^3^**  A0A0B4VCR4_HALAI | Glycine-rich boundary protein; 14.7% A, 15.7% Q, 9.3% G; pI 5.6; IDR (40.7%; aa1-83); [QQQA]_7_ repeats in aa22-45 | A0A0B4VCR4  _HALAI  (aa1-343) | 84.8 | 1.0e-36 | 3  2  3  - | 43  38  4  - | 0.252  0.180  0.010  - | I_A_  I_B_  S_A_  S_B_ |
| **H_sp_Tri_18127_c0_g1_i1^3^** | Uncharacterized; 10.0% A, 12.5% S, 11.0% T; pI 6.2; IDP |  |  |  | 9  10  8  5 | 133  94  25  20 | 0.149  0.102  0.248  0.096 | I_A_  I_B_  S_A_  S_B_ |
| **H_sp_Tri_20350_c0_g1_i1**  **H_sp_Tri_20347_c0_g1_i1**  **H_sp_Tri_20348_c0_g1_i1** | Uncharacterized; 19% P, 23.9% S; pI 7.3; IDP |  |  |  | 4  2  2  - | 7  3  2  - | 0.002  0.001  0.002  - | I_A_  I_B_  S_A_  S_B_ |
| **H_sp_Tri_21565_c0_g1_i1**  (aa45-267) | Similar to Kunitz/bovine pancreatic trypsin inhibitor domain protein; domains: Kunitz_BPTI (aa39-96,99-153,161-263); SSP (aa1-23; 10.0% C, 10.0% G; pI 8.4; IDR (14.3%; aa33-43,257-282) | A0A0B1PLG7  _9BILA  (aa679-919) | 35.4 | 2.1e-26 | 4  5  -  - | 26  49  -  - | 0.006  0.017  -  - | I_A_  I_B_  S_A_  S_B_ |
| **H_sp_Tri_24151_c0_g1_i1^3^** | Uncharacterized/similar to AP7; SSP (aa1-19); 9.9% L; pI 8.3 | Q9BP37_HALRU**^4^**  (aa1-85) | 43.5 | 0.0005 | 3  3  2  2 | 66  64  2  6 | 0.035  0.200  0.001  0.017 | I_A_  I_B_  S_A_  S_B_ |
| **H_sp_Tri_25106_c0_g1_i1^3^**  (aa33-537)  Comp246916_c0_seq1_3 | Similar to shell protein 4¸ SSP (aa1-26)/TM (aa12-31); see also idb_20988; aa12-21 (IFLL[F,P])_2_ | A0A0G2YN89  _MYTCO**^4^**  (aa39-540) | 35.4 | 8.4e-56 | 37  27  7  5 | 1137  810  16  10 | 3.350  1.706  0.021  0.008 | I_A_  I_B_  S_A_  S_B_ |
| **H_sp_Tri_29101_c0_g1_i1^3^** | Uncharacterized; 10.4% A, 12.3% S, 16.7% T, pI 6.8; domain: RmIC-like_jelly_roll_fold (aa146-268); IDR (aa1-140); repeats (Fig. S2Za) |  |  |  | 9  7  -  - | 126  67  -  - | 0.043  0.024  -  - | I_A_  I_B_  S_A_  S_B_ |
| **H_sp_Tri_31892_c0_g1_i1^3^**  (aa1-198)  **Comp22593_c0_seq1_3** | Similar to ependymin-related protein 1; domain: ependymin (aa71-198); SSP (aa1-17); pI 5.0; shares several peptides with CLC_160 | EPDR1_HALAI**^4^**  (aa1-198) | 84.3 | 3.1e-198 | 7  7  2  2 | 1612  1720  22  14 | 13.918  9.714  0.413  0.099 | I_A_  I_B_  S_A_  S_B_ |
| **H_sp_Tri_31897_c0_g1_i1^3^**  (aa1-198) | Similar to ependymin-related protein 1; domain: ependymin (aa70-198); SSP (aa1-17); pI 5.1;shares peptides with idb_16318/16846 and CLC_1876 | EPDR1_HALAI**^4^**  (aa1-198) | 78.3 | 44.3e-74 | 14  11  1  - | 531  321  1  - | 2.763  1.573  0.001  - | I_A_  I_B_  S_A_  S_B_ |
| **H_sp_Tri_35519_c0_g1_i1^3^**  (aa51-376) | Uncharacterized; pI 7.5; domain: ConA_like (aa216-373); TM (aa20-42) | K1QF85_CRAGI  (aa421-782) | 25.8 | 2.5e-10 | 13  12  -  - | 441  376  -  - | 0.614  0.482  -  - | I_A_  I_B_  S_A_  S_B_ |
| **H_sp_Tri_3731_c0_g1_i1^3^**  (aa41-138)  Comp92088_c0_seq1_4 | Uncharacterized; SSP (aa1-30)/TM (aa5-27); 10.6% C; pI 7.3, if SSP: 12.6% C; pI 7.6; share most peptides | V4ALS8_LOTGI  Lotgidraft_239117  (aa118-216) | 41.4 | 1.4e-12 | 8  6  9  3 | 89  13  23  3 | 0.035  0.008  0.138  0.004 | I_A_  I_B_  S_A_  S_B_ |
| **H_sp_Tri_4200_c0_g1_i1**  (aa88-715)  Comp33684_c0_seq1_1  Comp38651_c0_seq2_1 | Uncharacterized/similar to peroxinectin/peroxidasin; domains: haem_peroxidase_SF (aa91-502,606-716); SSP (aa1-21); 10.1% R; pI 9.5 | A0A0P4WN25  _9EUCA | 38.8 | 9.6e-63 | 5  9  -  - | 18  36  -  - | 0.002  0.004  -  - | I_A_  I_B_  S_A_  S_B_ |
| **H_sp_Tri_45070_c0_g1_i1^3^**  (aa100-1404) | Uncharacterized; not all peptides in alignment; 11.8% P, 12.2% S, 12.3% T, pI 11.0; IDP; ; short tandem repeats in aa334-343 (STTXP)_2_ , aa500-521 ((PSXASXT[S,P])_2_, aa873-883 (TSQ[P,S]T)_2_ | A0A068Y7U9  _ECHMU  (aa9-1297) | 22.1 | 1.7e-13 | 16  18  6  6 | 176  161  15  23 | 0.009  0.011  0.007  0.008 | I_A_  I_B_  S_A_  S_B_ |
| **H_sp_Tri_45832_c0_g1_i1**  (aa14-215) | Uncharacterized; domain: CpcT/CpeT (aa32-190); SSP (aa1-23); 9.8% S; pI 5.5 | V4A8D6_LOTGI  Lotgidraft_228780  (aa22-224) | 56.4 | 2.5e-48 | 2  2  -  - | 4  6  -  - | 0.002  0.001  -  - | I_A_  I_B_  S_A_  S_B_ |
| **H_sp_Tri_50040_c0_g1_i1**  (aa12-410) | Uncharacterized; domains: chitin-bd (aa61-105), ConA-like (aa227-373); SSP (aa1-21); pI 5.0; IDR (7.1%; aa37-50,387-400) | V4B726_LOTGI  Lotgidraft_236297  (aa9-412) | 58.2 | 2.4e-115 | 8  6  -  - | 105  37  -  - | 0.024  0.008  -  - | I_A_  I_B_  S_A_  S_B_ |
| **H_sp_Tri_50969_c0_g1_i1^3^**  (aa34-373)  Comp105719_c0_seq1_2  Comp96030_c1_seq6_5 | Uncharacterized; domains: EGF_like (aa59-94,96-131,146-181,183-231,292-334,341-373); 13.7% C,12.8% G, 9.7% S; pI 6.3; IDR (6.5%; aa404-431) | V3ZDP8_LOTGI  Lotgidraft_91483  (aa39-374) | 41.9 | 1.9e-42 | 5  2  -  - | 38  4  -  - | 0.006  0.001  -  - | I_A_  I_B_  S_A_  S_B_ |
| **H_sp_Tri_57542_c0_g1_i1** | Uncharacterized; SSP (aa1-19); 12.3% A, 13.2% P, 13.2% S; pI 8.2; IDP; only 1 measurable peptide predicted |  |  |  | 1  1  1  - | 12  6  2  - | 0.014  0.012  0.008  - | I_A_  I_B_  S_A_  S_B_ |
| **H_sp_Tri_58705_c0_g1_i1**  (aa37-240) | Uncharacterized; domains: Kunitz_BPTI (aa35-88,89-143,146-196); SSP (aa1-19); 12.5% S, 9.8% T; pI 9.4; IDR (36.6%; aa295-302,312-429,454-507,527-537); not all peptides in alignment! | L7LTN2_9ACAR  (aa348-545) | 36.2 | 1.6e-22 | 6  5  -  - | 29  34  -  - | 0.003  0.006  -  - | I_A_  I_B_  S_A_  S_B_ |
| **H_sp_Tri_61496_c0_g1_i1^3^**  (aa23-241) | Similar to ferric-chelate reductase 1; domain: Reeler (47-173); TM (aa21-43); 13.5% S; pI 9.6; IDR (27.4%; aa194-266) | K1Q9X0_CRAGI  (aa1-224) | 29.8 | 4.8e-7 | 9  6  -  - | 206  164  -  - | 0.166  0.118  -  - | I_A_  I_B_  S_A_  S_B_ |
| **H_sp_Tri_62946_c0_g1_i1^3^**  (aa15-735)  Comp99065_c0_seq1_4 | Putative prosaposin; domains: multiple saposin_like/saposinB, saposinA (aa705-738); SSP (aa1-18); 10.6% L, 11.2% V; pI 5.0; IDR (0.8%; aa411-417); shares several peptides with Tri_62947 | A0A0KJRE48  _IXORI  (aa621-1340) | 31.3 | 4.4e-43 | 12  13  -  - | 111  116  -  - | 0.021  0.030  -  - | I_A_  I_B_  S_A_  S_B_ |
| **H_sp_Tri_62947_c0_g1_i1**  (aa1-282) | Uncharacterized; domains: saposinA (aa1-49,67-148,157-238), saposinB (aa247-283); 11.7% L, 10.2% V; pI 7.0; IDR (2.5%; aa56-62); shares several peptides with Tri_62946 | R7VKI2_CAPTE  (aa291-578) | 42.0 | 1.9e-51 | 1  1  -  - | 1  2  -  - | <0.001  <0.001  -  - | I_A_  I_B_  S_A_  S_B_ |
| **H_sp_Tri_63049_c0_g1_i1^3^**  (aa64-203) | Similar to putative ferric-chelate reductase 1-like protein; aa54-306 similar to ML7B12**^4^;** domain: Reeler (aa66-226); 10.0% T; pI 10.0; IDR (33.8%; aa30-54,217-286,301-311) | A0A087TMH4  _9ARAC  (aa10-152) | 34.5 | 4.9e-9 | 6  5  -  - | 132  103  -  - | 0.023  0.015  -  - | I_A_  I_B_  S_A_  S_B_ |
| **H_sp_Tri_64952_c0_g1_i1^3^** | Uncharacterized; SSP (aa1-19); 10.8% R, 10.8% G, 18.5% S, pI 9.7; IDR (43.6%; aa55-140) |  |  |  | 3  2  -  - | 17  4  -  - | 0.006  <0.001  -  - | I_A_  I_B_  S_A_  S_B_ |
| **H_sp_Tri_6552_c0_g1_i1**  (aa3-610) | Similar to carbonic anhydrase; domain: Carbonic_anhydrase_a (aa33-307); 10.1% P, 9.0% S; pI 9.6; IDR (34.5%; aa306-353,380-430,479-568,600-641) | J7QJT8_PATVU  (aa4-612) | 34.7 | 4.7e-43 | 11  10  -  - | 81  51  -  - | 0.010  0.007  -  - | I_A_  I_B_  S_A_  S_B_ |
| **H_sp_Tri_68552_c0_g1_i1**  (aa2-216)  Comp94919_c0_seq1_6 | Uncharacterized; domain: LRR/L_like (aa19-187); SSP (aa1-17); 18.5% S; pI 9.0; IDR (42.4%; aa164-272) | C3YC14_BRAFL  (aa5-224) | 31.7 | 8.8e-13 | 4  2  -  - | 21  4  -  - | 0.004  0.002  -  - | I_A_  I_B_  S_A_  S_B_ |
| **H_sp_Tri_72839_c0_g1_i1^3^**  (aa6-357) | Carbonic anhydrase; domain: Carbonic_anhydrase_a (aa54-352); SSP (aa1-21); pI 8.4; IDR (5.7%; aa239-258) | G0YYQ3_HALTU  (aa1-352) | 78.5 | 8.9e-129 | 9  6  -  - | 69  26  -  - | 0.001  0.010  -  - | I_A_  I_B_  S_A_  S_B_ |
| **H_sp_Tri_73035_c0_g1_i1^3^** | Uncharacterized; TM (aa40-62); 15,8% A, 10.6% G, 14.6% P; pI 6.4; IDP |  |  |  | 7  7  2  - | 112  67  5  - | 0.056  0.041  0.019  - | I_A_  I_B_  S_A_  S_B_ |
| **H_sp_Tri_77019_c0_g1_i1**  (aa9-1438)  Comp106561_c0_seq3_3 | Thioester-containing protein; domains: A2M_N (aa124-201), A2M_N2 (aa499-596), A2M (aa718-809), A2M_thiol_ester-form (aa937-966), A2M_comp (987-1230), A2M_receptor_bind (1320-1420); SSP (aa1-17); 9.4% S, 9.8% V; pI 8.7; IDR (1.5%; aa685-705) | Q3V653_9EUPU  (aa5-1443) | 39.6 | 1.3e-211 | 9  2  -  - | 34  4  -  - | 0.001  <0.001  -  - | I_A_  I_B_  S_A_  S_B_ |
| **H_sp_Tri_7902_c0_g1_i1^3^**  (aa52-258)  Comp80057_c0_seq1_3 | Uncharacterized; domains: chitin-bd_II (aa50-113,117-187,209-263); 9.5% S; pI 8.3 | V3ZHU5_LOTGI  Lotgidraft_169029  (aa28-239) | 45.9 | 4.5e-46 | 7  5  -  - | 51  44  -  - | 0.011  0.013  -  - | I_A_  I_B_  S_A_  S_B_ |
| **H_sp_Tri_79843_c0_g1_i1** | Uncharacterized; domain: Reg_factor_effector (aa61-140), SOUL_haem_bd (aa69-305); SSP (aa1-18); pI 8.7; IDR (7.4%; aa27-48) |  |  |  | 8  7  -  - | 21  39  -  - | 0.006  0.012  -  - | I_A_  I_B_  S_A_  S_B_ |
| **H_sp_Tri_81308_c0_g1_i1^3^** | Uncharacterized; pI 8.8 |  |  |  | 2  2  -  - | 30  15  -  - | 0.021  0.015  -  - | I_A_  I_B_  S_A_  S_B_ |
| **H_sp_Tri_81534_c0_g1_i1**  (aa14-682)  Comp101219_c1_seq3_6 | Uncharacterized; domain: Glycoside_hydrolase_SF (aa48-346); TM (aa16-38); pI 5.8; IDR (1.9%; aa475-487) | A0A0B7AEQ2  _9EUPU  (aa6-671) | 61.9 | 2.6e-191 | 5  2  -  - | 41  16  -  - | 0.003  0.002  -  - | I_A_  I_B_  S_A_  S_B_ |
| **H_sp_Tri_83476_c0_g1_i1^3^**  (aa5-554) | Uncharacterized; SSP (aa1-21); pI 5.4; IDR (12.9%; aa486-554) | V4A4X8_LOTGI**^4^**  Lotgidraft_233348  (aa34-592) | 29.1 | 2.1e-63 | 12  11  -  - | 111  86  -  - | 0.026  0.028  -  - | I_A_  I_B_  S_A_  S_B_ |
| **H_sp_Tri_90659_c0_g1_i1^3^** | Uncharacterized; SSP (aa1-23); 13.9% R; pI 10.6 |  |  |  | 2  2  -  - | 4  3  -  - | 0.002  0.002  -  - | I_A_  I_B_  S_A_  S_B_ |
| **H_sp_Tri_95672_c0_g1_i1**  (aa14-382)  Comp58675_c0_seq1_4 | Uncharacterized; domains: chitin-bd_II (aa34-88,89-141,148-205), ConA-like aa226-385); SSP (aa1-21); 10.0% G, 9.5% K; pI 9.5 | K1PSP7_CRAGI  (aa34-399) | 36.9 | 3.0e-53 | 15  15  -  - | 199  264  -  - | 0.057  0.106  -  - | I_A_  I_B_  S_A_  S_B_ |
| **H_sp_Tri_97599_c0_g1_i1**  (aa16-339)  **H_sp_Tri_97597_c0_g1_i1** | Uncharacterized/placental protein 11; domains: Somatomedin_B (aa24-65), endoribonuclease_XendoU_fam (aa82-339); SSP (1-21); pI 5.7 | V3ZZL2_LOTGI  Lotgidraft_220728  (aa17-342) | 38.4 | 3.3e-46 | 3  2  -  - | 10  4  -  - | 0.003  0.001  -  - | I_A_  I_B_  S_A_  S_B_ |
| **PLC_HALLA^3,4^**  (P82596)  H_sp_Tri_110681_c0_g1_i1  Comp101672_c0_seq9_5 | Perlucin; domain: C-type_lectin-like (aa1-141); pI 7.2;; shares most peptides with F8J3D2 (perlucin C) and 1 with comp25997_c0_seq1_1; pI 7.2; IDR (9.7%; aa141-155) |  |  |  | 16  2  7  4 | 685  7  13  17 | 2.397  0.003  0.026  0.040 | I_A_  I_B_  S_A_  S_B_ |
| **F8J3C9_HALLA^3^** | Perlucin A; domain: C-type_lectin-like (aa27-146), SSP (aa1-18); pI 6.5; shares most peptides with F8J3D2_HALLA and PLC_HALLA |  |  |  | 2  -  -  - | 3  -  -  - | 0.001  -  -  - | I_A_  I_B_  S_A_  S_B_ |
| **F8J3D2_HALLA^3^**  F8J3D1_HALLA  F8J3D0_HALLA  H_sp_Tri_110681_c0_g1_i1  Comp101672_c0_seq1_4 | Perlucin C; domain: C-type_lectin-like (aa27-146); SSP (aa1-18); 9.5% Q, 13.1% L; pI 6.7; IDR (14.9%; aa207-240); shares most peptides with perlucin (PLC_HALLA) , perlucin A, and 1 with comp25997_c0_seq1_1 |  |  |  | 3  8  1  - | 247  212  1  - | 0.127  0.139  0.001  - | I_A_  I_B_  S_A_  S_B_ |
| **Comp25997_c0_seq1_1^3^**  (aa1-88) | Similar to perlucin; domain: C-type_lectin (aa1-88); 11.4% R, 9.1% E, 10.2% G, 10.2% L; pI 7.1 | PLC_HALLA**^4^**  (aa42-129) | 65.9 | 1.3e-25 | 2  1  -  - | 35  3  -  - | 0.037  0.003  -  - | I_A_  I_B_  S_A_  S_B_ |
| **PWAP_HALLA^3,4^**  (P84811) | Perlwapin; domains: WAP (aa2-43,44-89,90-132); 18.7% C, 11.9% G, 15.7% P; pI 8.6; shares 2 peptides with Comp236269_c0_seq1_4 |  |  |  | 7  7  2  - | 175  72  4  - | 0.798  0.231  0.023  - | I_A_  I_B_  S_A_  S_B_ |
| **Comp236269_c0_seq1_4^3^**  (aa1-82) | Perlwapin; domains: WAP (aa1-32,35-80); 19.5% C, 11.5% G, 12.6% P; pI 8.6; IDR (5.6%; aa1-5); shares 1 peptide with PWAP_HALLA; in one group in I_A_ | PWAP_HALLA**^4^**  (aa71-152) | 92.7 | 1.4e-30 | -  3  -  - | -  11  -  - | -  0.014  -  - | I_A_  I_B_  S_A_  S_B_ |
|  |  |  |  |  |  |  |  |  |

**^1^**, the database entry(s) with the highest number of peptides (majority protein) is shown first, irrespective of the database of origin; the best entry of the respective other databases is added in bold print if identified with the same number of peptides, in normal print if identified with fewer peptides; additional entries in the same group but with fewer peptides are available in the respective Additional ProteinGroup files. **^2^**, I, acetic-acid insoluble; S, acetic acid-soluble; A, shell washed with sodium hypochlorite before demineralization; B, hypochlorite washing with short sonication intervals. **^3^**, also identified in nacre (Table Sx). **^4^**, previously identified in the shell proteome of *Haliotis laevigata* (_HALLA) [14,16,19], of *H. asinina* (_HALAI) [21], of *H. rufescence* (_HALRU) [12,17], of *H. tuberculata* [22], of the limpet *Lottia gigantea* (_LOTGI) [58,69,105], the oyster *Crassostrea gigas* (_CRAGI) [133], the slug *Arion vulgaris* (_9EUPU) [136], the mussel *Mytilus californianus* (_MYTCA) [23], the mussle *Mytilus coruscus* (_MYTCO) [25], the oyster *Pinctada maxima* (_PINMA) [122], and the scallop Mizuhopecten yessoensis (_MIZYE) [76]. Amino acid positions and the data in columns 2 to 6 refer to the majority protein. Predicted domains are abbreviated according to InterPro (<http://www.ebi.ac.uk/interpro/>). SSP, predicted secretion signal peptide; TM, predicted transmembrane helix. IDP, predicted intrinsically disordered protein (predicted disorder <90%); IDR, predicted intrinsically disordered sequence regions. Composition, pI and IDR percentages were calculated without predicted signal peptide sequence.
