# Supplementary material for: In-depth proteomic analyses of Haliotis laevigata (greenlip abalone) nacre and prismatic organic shell matrix
Source: Proteome Sci. 2018 Jun 15;16:11. doi: 10.1186/s12953-018-0139-3 (PMC6003135; doi:10.1186/s12953-018-0139-3)
Supplement: Supplementary file 27 — Figure S2. Sequences and repeat structure of uncharacterized major proteins. Sequence regions covered by identified peptides are shown in bold green. Predicted signal sequence peptides are underlined. Collagen triple-helical sequences are in italics. In sequence alignments identical amino acids are shaded yellow. (DOCX 75 kb) [file 12953_2018_139_MOESM27_ESM.docx]

**Figure S2A**

idb_54497 1 MSFFPCLLALTLSPCLVGAQTMGGVSGMSEMGMPGGMPMLTPSGMPGGMPMLTPSGIPGT

CLC_12027 1 MSFFPCLLALTLSPCLVGAQTMGGVSGMSEMGMPGGMPML--------------------

idb_54497 61 GMPGGMPMVTPSGIPGTGMPGGMPMGTPSGMPGMGMPGGMPMGMPGGLMGGMASAIMGRR

CLC_12027 41 ---------TPSGIPGTGMPGGMPMGTPSGMPGMGMPGGMPMGMPGGLMGGMASAIMGRR

idb_54497 121 GSSFR**RSPFYLGAKKLGCSYGIDMESMLR**FLLPRGCSPGFDFCPATFRGR**RCVQAGAIGM**

CLC_12027 92 GSSFR**RSPFYLGAKKLGCSYGIDMESMLR**FLLPRGCSPGFDFCPATFRGR**RCVQAGAIGM**

idb_54497 181 **CCPLYVNKRAIDSTANMILMTKMAEMFAL**

CLC_12027 152 **CCPLYVNKRAIDSTANMILMTKMAEMFAL**

**idb_54497**

**32 GMPGGMPMLTPS-----**

**44 GMPGGMPMLTPSGIPGT**

**61 GMPGGMPMVTPSGIPGT**

**78 GMPGGMPMGTPSGMPGM**

**95 GMPGGMPMGMPGG**

**CLC_12027:**

**31 MGMPGGMPMLTPSGIPG**

**53 TGMPGGMPMGTPSGMPG**

**70 MGMPGGMPMG**

**Figure S2B**

CLC_123 1 MKPLLQTLLFTLLLSVLVDPQAPNFQLATPRVR**QALALANYLMR**RPVGERSIRRDCR**LIP**

idb_32947 1 MKPLLQTLLFTLLLSVLVDPQAPNFQLATPRVR**QALALANYLMR**RPVGERSIRRDCR**LIP**

CLC_123 61 **QSDLDNIFNAIRR**AKADTRLRPNVHDCFAFLHSHPQVXXXXXXXX-------------XX

idb_32947 61 **QSDLDNIFNAIRR**AKADTRLRPNVHDCFAFLHSHPQVNAGAHGGPGFLPWHRVFVFLYEK

CLC_123 108 XXXXXXXXVSMCFWDSTLEPDNFAESSTWTEALFGNGNGPVTSGPGAGWTTPVGRLTRDA

idb_32947 121 MLRMYEPTVSMCFWDSTLEPDNFAESSTWTEALFGNGNGPVTSGPGAGWTTPVGRLTRDA

CLC_123 168 GQRGRPLNMADIENILSQGTLGEVSFPNGHVSSNVEEVHNHVHVHVGGHMGQIETAAYDP

idb_32947 181 GQRGRPLNMADIENILSQGTLGEVSFPNGHVSSNVEEVHNHVHVHVGGHMGQIETAAYDP

CLC_123 228 IFWFHHTFVDCIYEWFRDEQKSRGTVNVQRDWPR**DWGESAHGPFR**AMRLGSLR**NIDGQND**

idb_32947 241 IFWFHHTFVDCIYEWFRDEQKSRGTVNVQRDWPR**DWGESAHGPFR**AMRLGSLR**NIDGQND**

CLC_123 288 **FFSTR**VLQCHRVPRCRTNADCGRHMTCEARSSRCTSTTRVTQSNNFFN*GLMGMLGNIGQV*

idb_32947 301 **FFSTR**VLQCHRVPRCRTNADCGRHMTCEARSSRCTSTTRVTQSNNFFN*GLMGMLGNIGQV*

CLC_123 348 *GNLGNLGNM*FGGNIGGFNQNNGPGAMNGFNTFR**NPNQFGGPQQGNFGGPPGGQRNFGGQP**

idb_32947 361 *GNLGNLGNM*FGGNIGGFNQNNGPGAMNGFNTFR**NPNQFGGPQQGNFGGPPGGQRNFGGQP**

CLC_123 408 **GGPPFGIQGNPQFGPQGNQFGR**PQGGNGFGGMGPMQGGPMQGGPMQGGPM----------

idb_32947 421 **GGPPFGIQGNPQFGPQGNQFGR**PQGGNGFGGMGPMQGGPMQGGPMQGGPMPGGPMPGGQG

CLC_123 ------------------

idb_32947 481 GNFGRGGGGQFGFGQFGK

idb_32947:

**405 NFGGPPGGQR**

**416 NFGGQPGGPP**

**424 P-FG-IQGN**

**431 PQFG-PQGN**

**439 -QFGRPQG**

**453 GPMQG**

**458 GPMQG**

**463 GPMQG**

**468 GPMPG**

**473 GPMPG**

**489 GQFGF**

**494 GQFGK**

**Figure S2C**

Tri_107535 1 -----------------------TRRSSDLGGGGGGGSGSASASASASASAR**ANSINNLA**

CLC_21 1 MLRVPLLVLCLALSVGADYYGYGWGRNGGGGGGGGGGSGSASASASASASAR**ANSINNLA**

Tri_107535 38 **GRLSSLVDASASAR**ASANANAGGFGGSGGGGSGGNGGSGGSGFGGASASASAEALASATA

CLC_21 61 **GRLSSLVDASASAR**ASANANAGGFGGSGGGGSGGNGGSGGSGFGGASASASAEALASATA

Tri_107535 98 ELQSAQDAYDQASAYAEATAQAAANGGSVDSSALATAIASAEASVSARGGSIIARARARA

CLC_21 121 ELQSAQDAYDQASAYAEATAQAAANGGSVDSSALATAIASAEASVSARGGSIIARARARA

Tri_107535 158 EASVRAAR**RAFASAQASAEASVSAVR**SSEGRARSFARAVARAR**ASARAAIAGVR**SSGR**AF**

CLC_21 181 EASVRAAR**RAFASAQASAEASVSAVR**SSEGRARSFARAVARAR**ASARAAIAGVR**SSGR**AF**

Tri_107535 218 **ASATARARASVSAAGRAVASARAQAVAR**ARAGAR**ASASASARASASAAASARASAYARVQ**

CLC_21 241 **ASATARARASVSAAGRAVASARAQAVAR**ARAGAR**ASASASARASASAAASARASAYARVQ**

Tri_107535 278 **VAAAAAAR**AAASAAASASASASSSASGSSFGSGGAGGSGNGGFSSFGASANAVANAFAQA

CLC_21 301 **VAAAAAAR**AAASAAASASASASSSASGSSFGSGGAGGSGNGGFSSFGASANAVANAFAQA

Tri_107535 338 FGSSSGGGSGGGGSGNGGRGSGGSGGSGSGGSGG-----------------

CLC_21 361 FGSSSGGGSGGGGSGNGGRGSGGSGGSGSGGSGGSGGRGGRYYYGSSDYYY

**17 SA**

**19 SA**

**21 SA**

**23 SA**

**25 SA**

**27 SA**

**59 G-GFGGS-**

**65 G-G-GGS-**

**70 G-GNGGSG**

**77 GSGFGG**

**169 ASAQ**

**173 ASAE**

**177 AS**

**182 VRSSEGRARSFARAVARARASARAA-IAG**

**210 VRSS-GRA--FASATARARASVSAAGRA-**

**234 AVASARAQ**

**242 AVARARAG**

**250 ARASA**

**250 ARASASASAR**

**260 ASASAAASAR**

**270 ASAYA**

**345 GSGG-GGSGNGGR**

**357 GSGGSGGSGSGGS**

**Figure S2D**

CLC_253

1 MDTKKWTLSAHGINTKHILIGAVSDFWVNLHIQCFYWTESGPGSCSFGWGNKSATISTIS

61 TTATARTTTATTTPTARTTAAXXXXXXXXXXXXXXXXXQQQQQQLLQQQQQQQQQGLQQQ

121 QQGLQQQQQQQQQGLQQQQQQQQQQVLKQQQQQQQR**LQQQQQQQQFGFR**SQEGR**GQGQNS**

181 **VRPR**AAPQTNNR**AILNMLLWNQIAQSNRMNTLALIMGN**

**64 TARTT-**

**69 TATTTP**

**75 TARTT**

**99 QQQQQQLL-QQQ**

**110 QQQQQQGL----**

**118 -QQQQQGL-QQQ**

**127 QQQQQQGLQQQQ**

**139 QQQQQQVL--KQ**

**149 QQQQQQRL-QQQ**

**160 QQQQQ**

**Figure S2E**

CLC_303

1 LAQAQAEAQAQAQAQAQAVAAAQAQADAQAQAQAQAQAEAAAQAQAQADAQAQAQAQAQA

61 QAQAQAEAQAQAEAHAERDRKALGK**LKAQAEANIRAFSQALAAADAEAQEYTTTLTATVA**

121 **QANSLAQAQANAKSKAEEDEFSLAK**TAADSLVHILTQAQAHATAEAAAFATAMEATTGII

181 QAQSIAHAQSLAQAR**VTAEAQAR**AEAEAK**SLTTVELQK**ALEALAK**AEANAQAVSMANVDA**

241 **K**AAAQVQAKAEAKARAEAQAKAAAQAQAEAQAAAQAQAAAQAQAAAQAQAAAQAQASAQA

301 QAKAKAQAEAR**ASVEASAQSSR**NTTGAPMQPK**RHDVPDYLTLDELRMSFTTGSFDRNTWY**

361 **GINPRFCNQFLEFHMSEKGEFYVANRMCPYGLFWLQDYMRCMAPSSVPCSIDPCR**MNGKT

421 GVVYAR**QDTCGGFYECTRDTSSPYIYAKPMCCPHK**HRFNSK**RGYCVPDDK**CR**EACGVPDD**

481 **LASANGCWK**VAVPFDR**MAYYQVTESQNTYALQVMPCPPGTIYSPKKCHCVTSAILAGPDL**

541 **VNPCLPSMRFNFDMGKVEDVMHSGAAFAQR**EVKIIRKKNRMCARFGKGSFIKFWSFSNRS

601 LGK**KFGLALNFRINYVPNKPQSIISNCNRPSVQLYIVGK**TLYAR**ANNVEIKLPYK**IGKWN

661 SVKYIFDATRGMFELICNDEVQIEGVPSGEGLAATSSCLTIGLCLDGSR**HMEGFDGDLDD**

721 **VEIFECAPSDSTTPDSQNQIVSIFH**

**aa2-72: [AQ]_28_ plus [AX]_7_**

**192 AQARVTAE**

**200 AQARAEAE**

**240 AKAAAQVQ--AKAEAKARAEAQ**

**260 AKAAAQAQAEAQAAAQAQAAAQ**

**282 AQAAAQAQ--A**

**Figure S2E**

CLC_4 1 -----------------------MKCFVPLACLLVAVTGYAIKKPPCDKRPYK**GDNRKFQ**

Tri_11338 1 LAFIHSCPGGQSQREFRWPASFKMKCFVPLACLLVAVTGYAIKKPPCDKRPYK**GDNRKFQ**

CLC_4 39 **QFIPGTGWKTMSCAVGTAYSQATCECSILVKNDDRVKPGYGWK**SKGYGKKRYDNGYNNGY

Tri_11338 61 **QFIPGTGWKTMSCAVGTAYSQATCECSILVKNDDRVKPGYGWK**SKGYGKKRYDNGYNNGY

CLC_4 99 DNGYDNGNGNGNGNGYDNGYDNGNGNGNGNGYDNGYDNGNGNGNGNGNGNGNGNGNGNGN

Tri_11338 121 DNGYDNGNGNGN--------------------------------GNGNGNGNGNGNGNGN

CLC_4 159 GDDNGDDDSWLDR-----------------------------------------------

Tri_11338 149 GDDNGDDDSWLYGGGRPEPYPEVPFPEGFPIGVVGGPLPGLLNFLSGGGGGGGGGGAGSW

CLC_4 ------------------------------------

Tri_11338 209 ELDDDDDDDDDDDDDDDDDDDDDDDDDDDDDDDDDD

CLC_4:

**95 NGYDNGYDNGNGNGNG**

**111 NGYDNGYDNGNGNGNG**

**127 NGYDNGYDNGNGNGNG**

**143 NG--NGNGNGNGNGNG**

**153 NGDDNG**

**Figure S2G**

Tri_33510 1 MWSRPLIAALVILAILASSDAQGRRRRRNRNRSRRRGGSR**NNDRNVIIIVPNNRNRGSTP**

CLC_62 1 MWSRPLIAALVILAILASSDAQGRRRRRNRNRSRRRGGSR**NNDRNVIIIVPNNRNRGSTP**

Tri_33510 61 **NVGNGGGLFDLAARGGESIVPPGFEAVSRSGGLGGPPPPPPDTPPPNIPSPAPAPANNVI**

CLC_62 61 **NVGNGGGLFDLAARGGESIVPPGFEAVSRSGGLGGPPPPPPDTPPPNIPSPAPAPANNVI**

Tri_33510 121 **SGSSGSSR**SSGIIADLFGSPPTANGSPFGSPSQPSTTNIESLLGGSFGNTSPAGLGLSGR

CLC_62 121 SGSSGSSRSSGIIADLFGSPPTANGSPFGSPSQPSTTNIESLLGGSFGNTSPAGLGLSGR

Tri_33510 181 **GPSSTGQNNPPLPPPPPFGRDPPSVGPRVDFPPGGSGPPNVPLDPGNNR**FGGQQQQQQQQ

CLC_62 181 **GPSSTGQNNPPLPPPPPFGRDPPSVGPRVDFPPGGSGPPNVPLDPGNNR**FGGQQQQQQQQ

Tri_33510 241 FQGGPNPVQQQQ-------------------------------QQLQQQQQGPNLLQQQR

CLC_62 241 FQGGPNPLQQQQQQQQFQLVPNPXXXXXXXXXXXXXXXXXXXQQQLQQQQQGPNLLQQQR

Tri_33510 269 **QQQFQAGQDFGQQQQQQQQR**FQGRPDFQQQQQQQQQQTPNNFQQQQQQQQQNQAFNQDLQ

CLC_62 301 **QQQFQAGQDFGQQQQQQQQR**FQGRPDFQQQQQQQQQQTPNNFQQQQQQQQQNQAFNQDLQ

Tri_33510 329 PQPPQQIPSNFLPGLTNALGGGPVAISAPGMNPSNVLQALVPGLGISLPGQTPLRGPSR**Q**

CLC_62 361 PQPPQQIPSNFLPGLTNALGGGPVAISAPGMNPSNVLQALVPGLGISLPGQTPLRGPSRQ

Tri_33510 389 **PTPPPSPGQGQTASQLSDLSAVMNIIQQQQRQNTLMESLLFNSM**

CLC_62 421 **PTPPPSPGQGQTASQLSDLSAVMNIIQQQQRQNTLMESLLFNSM**

CLC_62:

**235 QQQQQQFQGGPNPLQQ**

**251 QQQQQQFQLVPNP**

**327 FQQQQQQQQQ-QTPNN**

**342 FQQQQQQQQQNQ-AFN**

**Figure S2H**

CLC_73 1 PQGRQEARRETK*GLNGPPGPRGLPGPAGGPPGEQGLPGPQGPQGSQGLPGPQGPAGGPKG*

CLC_73 61 *DKGDTGLPGFDGLGGPQGPQGPQGPSGLPGLKGERGLPGADGVQGPVGYDGMDGKPGPPG*

CLC_73 121 *PAGERGLQGYDGLPGPDGLPGLKGQKGEPGAEGLQGPRGYDGYQGPPGVSVKGEAGPPGP*

CLC_73 181 *QGFAGPPGPIGPPGNPGGLGPAGLQGEK****GLSGGEGPQGAR****GWRGFAGLPGPPGKDGLPGP*

CLC_73 241 *PGPPGAQ*SAPRPTGSPTTFR**QGHNHNSIVMTGQPVLR**QPMNSAQTRVNPIYR**TIPPDPPA**

CLC_73 301 **SQQYPRQFAPSHSSTR**AQTVSIRQPRYPPGSFPQSAAAGSGTRTSVAVQRSLDPRFIGSG

CLC_73 361 GSGAAK**NSVSVNRPSPDGSSANTVKIKTGISSLDSTSTGKGGQAGTSSR**DTPGNTK**TNLP**

CLC_73 421 **QAILKALSPSSSSSVASSKPATATVTVKRAPASPATASSSKASVVISRSSKTSAESASPR**

CLC_73 481 **PSLTISRSVVNRPGGSNPR**PAATITISR**PSPTGAIPSK**SR**ALPVTVPAQSEPTARSSIKL**

CLC_73 541 **TRPEQSSSVASPARVSVSRPSSSASITISRQSQSGSNPSPSRPSISVSRPSSSASITISR**

idb_17035 1 --------------------------------QSGSNPSPSR**PSISVSRPSSSASITISR**

CLC_73 601 **QSQSASNPSPSQRSISVSRPASSASITISRQSQSASNPSPSRPSISISR**PASSSS-----

idb_17035 29 **QSQSASNPSPSRPSISISRPASSSSVTISRPFKSASVSSPKRASASVSRPVPSITISRHS**

idb_17035 89 **QSVSKPSQTGFSGTISRSAQSTSIFPLQKSSNSVSGPASSASVRTGRSFVSSAVSSPSRP**

Tri_121458 1 -------------------------------------------------------**PVPSA**

idb_17035 149 **AVSVSRPVPSASVLVSRPVPSASVSVSRPVSSAAVSSSPGASVFLTRPSPNTQSLSTGGS**

Tri_121458 6 **SVLVSRPVPSASVLVSRPVPSASVSVSRPVSSAAVSSSPGASVFLTRPSPNTQSLSTGGS**

idb_17035 209 **ILLR**QR**QRQAAAAASPAAGSQDQAATQRR**-------------------------------

Tri_121458 66 **ILLR**QR**QRQAAAAASPAAGSQDQAATQRRRPSFLSYR**LAATR**RSSSPGTNVASSIVRRPQ**

Tri_121458 126 **PSLGGTGASSLQAR**TPFMRYQSNRNNNALRSSTSSLRIIRHGGQATMPGNPPGHGGGSGA

Tri_121458 186 AGGAGGGAGSGAAGGAALQISAEVETE

CLC_73:

**556 SVSRP-SSSASITISRQSQSGSNPSPS-RPSI**

**586 SVSRP-SSSASITISRQSQSASNPSPSQR-SI**

**616 SVSRP-ASSASITISRQSQSASNPSPS-RPSI**

**646 SISRP-ASSSS**

Tri_121458:

**1 PVPSASVLVSR 180 GG-GSGAAGGAG**

**12 PVPSASVLVSR 191 GGAGSGAAGGA-**

**23 PVPSASVSVSR**

**34 PVSSAAVV**

**Figure S2I**

Idb_18725

1 PTSVPSNTGSYINTAQQTGASTSVPSNTGSYANTAQQTGAPTSAPSNTGSYINTAQQTGS

61 PTSAPSNTGSYINTVQQTGAPTSAPSNTGSYINTVQQPGAPTSAPSNTGSYINTAQQTGA

121 STSAPSNTGSYINTVQQTGAPTSAPSNTGSYANIAQQTGVPTSVPSNTGR**YDNTAQQTGV**

181 **R**VPSISMIGGQTNTAQQTGAPTSAPSNTGSYINTAQQTRAPVPSTSMTGSQTNTAQQIGT

241 IAVAPSMIGSQTNTFLQTGAPASAPSTIGSQTNTAQLLGTIAVNPSMIGSYTNTAQQTGA

301 HVPTASMIGSQSNTAQHTVAPTYGPFSTGNYMNSVHQTGTATQQISASHPNPAQK**ASQSG**

361 **VTGSR**TTQFQFNQQYPTSQYPTNPSASVGQYLAPNSQQGLSVNPLGASGPAQTVQYDRFG

421 QPVHGYNVHAHSQHGLQQTQNSLLAATINK**LVSLYHQVVALGGSRANQLLQSPLADVLSR**

481 NVPVYFTTTQAPTTTTVNPLIPNCPPTR**INLWCQSSSGDPSPPAIYPGEKWEFHNATDLK**

541 **QYCSHSCIGNRCEFTDCTCYCIDDLTK**SVLEPGEALDTTEILEHINTAQPTTKAASRTAA

**1 PT-SVPSNTGSYINTAQQTGA--**

**21 ST-SVPSNTGSYANTAQQTGA--**

**41 PT-SAPSNTGSYINTAQQTGS--**

**61 PT-SAPSNTGSYINTVQQTGA--**

**81 PT-SAPSNTGSYINTVQQPGA--**

**101 PT-SAPSNTGSYINTAQQTGA--**

**121 ST-SAPSNTGSYINTVQQTGA--**

**141 PT-SAPSNTGSYANIAQQTGV--**

**161 PT-SVPSNTGRYDNTAQQTGVRV**

**183 P--S-ISMIGGQTNTAQQTG--A**

**201 PT-SAPSNTGSYINTAQQTR--A**

**221 PVPST-SMTGSQTNTAQQ**

**229 GSQTNTAQQIGTIAVAPSMIGSQTNTFLQTGA--PASAPSTI**

**269 GSQTNTAQLLGTIAVNPSMIGSYTNTAQQTGAHVP-TA-SMI**

**309 GSQSNTAQ**

**374 QYPTS**

**379 QYPTN**

**Figure S2J**

Idb_20008

1 MSRLTYSLTLLAGLFCHVLSQGPNNFR**ILAPGNPMTDAFKPNQGFPQPSNR**FSGFQHPQQ

61 **HQPSNRQGFQPPHHQHQHYQQQQR**QQQQQQQQQQQQQQQQQQQQPNR**QAPMLPSPPQVPA**

121 **SPKAAVVAAAGPNPPVPGPAAGALVPKIPAVVTQFMGMSIHDICAALTLKGTGAQSDALD**

181 TIIK**NRFPNASVVTGFR**NRSNPMEYK**QMFGSGPWACAFDFYAGRNPMEVAGAFSDLVDGA**

241 **GCNIVCDKIR**GPRKIGMMGMLAAMNGGQNPMGPMTNPLAALGNLMSGMGNPMGGMGNPMG

301 GMGNPMGGMGNPMAGMGNPMGGMGNPMAGMGNPMNGMGNPTGGMGNPTGGMGNPTGGMGN

361 PMGGMGNPMNGMGNPMNGNPNNGGNNMQNLMGNIVNLMQNPGPKSGAMGNGRPQGQGPNN

421 GHQFVPPSPQPPRGNR**WGHPGPFPARAPLPFPDPSTQNAALWGK**DPFQITPPQPQPQQPQ

481 HPQQPQQPQHPQQPQQPHHPQQHQPQPTSQPQPTQVPFNQQMPQIPLHQNHFPSQPQNQR

541 PSPFPQPPNVQPQPQPPQQQQPQPQPQQPQQPQGLSANMPNLPQNQDPK**IAPHLSEALQR**

601 **MPEPQRTQFVNDAISAFFRTLGIQLPSPQQQASNHR**GW

**47 QPSNRFSGFQHP-QQH**

**62 QPSNR-QGFQPPHHQH**

**287 GMGNPMG**

**294 GMGNPMG**

**301 GMGNPMG**

**308 GMGNPMA**

**315 GMGNPMG**

**322 GMGNPMA**

**329 GMGNPMN**

**336 GMGNPTG**

**343 GMGNPTG**

**350 GMGNPTG**

**357 GMGNPMG**

**364 GMGNPMN**

**371 GMGNPMN**

**373 GNPMN**

**378 GNPNN**

**471 P--PQ-P-Q**

**477 PQQPQHPQQ**

**485 PQQPQHPQQ**

**494 PQQPHHPQQ**

**504 QPQPTS**

**510 QPQPTQ**

**545 PQP-PNVQ-PQ**

**554 PQP-P-QQ-QQ**

**562 PQPQP-QQPQQ**

**572 PQ**

**Figure S2K**

idb_22086 1 M**GGYDLEAATLKPAAK**STSLLKWLIIGTIMGIVGIGGVLIGLGVSGNLGKGSDENNLITG

idb_22087 1 M**GGYDLEAATLKPAAK**STSLLKWLIIGTIMGIVGIGGVLIGLGVSGNLGKGSDENNLITG

idb_22086 61 GPAQAGMFNGQNRQGSFGQNQNGFGMLGNAVGIPDDTPDNVPDVVENVVENINDVRNDIA

idb_22087 61 GPAQAGMFNGQNRQGSFGQNQNGFGMLGNAVGIPDDTPDNVPDVVENVVENINDVRNDIA

idb_22086 121 DNVADVVDTASDAANNVIDAAADIVDDVNNDVNDVVDDIRDDVAEIRGDVTDTVDDMLDD

idb_22087 121 DNVADVVDTASDAANNVIDAAADIVDDVNNDVNDVVDDIRDDVAEIRGDVTDTVDDMLDD

idb_22086 181 VDDVADGVNDVADDVDDVADDVDDVADDVTDDVDDMADEVNDVADDADDVTDVDDDDSR**E**

idb_22087 181 VDDVADGVNDVADDVDDVADDVDDVADDVTDDVDDMADEVNDVADDADDVTDVDDDDSR**E**

idb_22086 241 **DDDNVSEDIDDALEDIAEAREDLVDQATDALEDRLDGIDDQADDVRDDLEDRLEASLEGA**

idb_22087 241 **DDDNVSEDIDDALEDIAEAREDLVDQATDALEDRLDGIDDQADDVRDDLEDR**LEASLEGA

idb_22086 301 **EADLEDVLEDILDDNESDDDHSDDDSS---LENIR**DAL-----------EDRIEELTDND

idb_22087 301 EADLEDVLEGTVGDIPDTVDGVLGGASAGAFLEVGEGFSDGVSDVGDDIGDVVDEVADGL

idb_22086 347 DTYEGQDDGVDNDDSDDSSLDNIRDALEDR**IEDVAGGDI-GDDDDDDGFEK**QDGIDNDDS

idb_22087 361 DTL---GDGVEDMTLA---MAVAAGLMNPAPAAAMGGELVGDA--GDAFGDLVGDAGDAV

idb_22086 406 DDSPLENMGDALEDGSEDLTGDDDDDDD------STLDNMREALEDRIDDVTDDDDDSDD

idb_22087 413 D-DIGGEVGDAVGDTRDTFGGAAGDLDDMVGDVGDNMGDMYGGIGDAMGDVMGDMGDVMG

idb_42421 1 DDNDDTPLDNMR**EALEDRID-------TITDDR**--------------**TPDD**-

idb_22086 460 GAFEEQNDVDNDDSPLDNMREAFEDRID------DVTGDDNDNNNDDDTVEGQID**TPDD**-

idb_22087 472 GAGDMGDMLGDAG---DTMGDMFGGMSDAMGGMGDMLGDARDGM--GDMVGGMGDTMGDA

idb_42421 31 --------------**QDDLSN**------------------**VNDDNDDTPLDNMR**EALEDR**ID**

idb_22086 513 --------------**QDDLSN**---------------------**DDDDTPWDNMREALEDRID**

idb_22087 527 MGGMGDMLGDARDGMGDMVGGMGDTMGDAMGGMGDMLGDARDGMGDMYGGMGDTMGDAMG

idb_42421 59 **TITDDG—TPDDQDDISDDI**----------------------**DDTPLDNMR**EAVLDKIGDT

idb_22086 538 **TITDDR-TPDDQDDLS----------------------NDNDDTPWDNMR**EALEDR**IDTI**

idb_22087 587 GMGDMLGDARDGMGDMYGGMGDTMGDAMGGMGDMLGDARDTMGDMYGGMGDTMSDAMGGI

idb_42421 96 IDNETPYVVDSSEEADDVMADSVEDTVVDVTDEQNGMIDTMQDIVGDVMDAR**SDAVGNVD**

idb_22086 575 **TDDR**TPD-----------------------------------------------------

idb_22087 647 GDMMGDARDTMGDMFGGLGDAMGDMGDMIGIDGMDNMGGMLAAMEMNRR**MGMGSQPVSQY**

idb_42421 156 **DSSMDIGDNFADAVYNARVSIDDAFSDSVDAAADIR**DTAVDAMLEMDPTIGTINNGGDSM

idb_22086 ------------------------------------------------------------

idb_22087 707 **MGR**RTGPSMGTYMSPFPVNQMGPNVGQGTGQQMGPYMAPRIGQGMGPQTQPQAQSSGFPN

idb_42421 216 DGLMNTDEDGFDVVDE--

idb_22086 ---------------------

idb_22087 767 QLPMMLMDDFEMDFGFDM

idb_22086

**169 DVTDTVD**

**176 DMLDDVD**

**183 DVADGVN**

**190 DVADDVD**

**197 DVADDVD**

**204 DVADDVT**

**317 SDDDH**

**322 SDDDS**

**509 TPDDQDDLSNDDDDTPWDNMREALEDRIDTITDDR**

**544 TPDDQDDLSNDNDDTPWDNMREALEDRIDTITDDR**

idb_22087

**169 DVTDTVD**

**176 DMLDDVD**

**183 DVADGVN**

**190 DVADDVD**

**197 DVADDVD**

**204 DVADDVT**

**393 LVGDAGDAFGD**

**404 LVGDAGDAVDD**

**496 DAMGGMGDMLGDARDGMGDMVGGMGDTMG**

**525 DAMGGMGDMLGDARDGMGDMVGGMGDTMG**

**554 DAMGGMGDMLGDARDGMGDMYGGMGDTMG**

**583 DAMGGMGDMLGDARDGMGDMYGGMGDTMG**

**612 DAMGGMGDMLGDARDTMGDMYGGMGDTMS**

**641 DAMGGIGDMMGDARDTMGDMFGGLGDAMG**

idb_42421

**1 DDNDDTPLDNMREALEDRIDTITDDRTPDDQDDLSNVN**

**39 DDNDDTPLDNMREALEDRIDTITDDGTPDDQDDIS---**

**70 DDIDDTPLDNMREA**

**Figure S2L**

Tri_117880 1 ADTTAAPTTEAADTTAAPTTEAADTTAAPTIAADTTAAPTAAADTTAAR**TEAASSVETSL**

idb_23862 1 -----------------------------------------------------**SSVETSL**

Tri_117880 61 **PRVNSAPLPDPVR**DATAVPSGVADTTATASSSSSSADTTTAPSPAADTTPAPTAAAETTA

idb_23862 8 **PRVNSAPLPDPVR**DATAVPSGVADTTATASSSSSSADTTTAPSPAADTTPAPTAAAETTA

Tri_117880 121 ASTEGQSAVHAPESNMIDK**PSDIAGAQLK**TQK**TVTSAQSVTSLASAGTDGLNHDADKPLN**

idb_23862 68 ASTEGQSAVHAPESNMIDK**PSDIAGAQLK**TQK**TVTSAQSVTSLASAGTDGLNHDADKPLN**

Tri_117880 181 **ADVPHVSK**LTPELAR**LLLAAGLQPCPEMFGHTK**ATSTHTSDASGQMTSSQHTVSESNIHS

idb_23862 128 **ADVPHVSK**LTPELAR**LLLAAGLQPCPEMFGHTK**ATSTHTSDASGQMTSSQHTVSESNIHS

Tri_117880 241 QLHSANLPQAPVPQSASASSVLHAASSHPLPSTVAASGTQTAATTTAGETPKGPSETK**NA**

idb_23862 188 QLHSANLPQAPVPQSASASSVLHAASSHPLPSTVAASGTQTAATTTAGETPKGPSETK**NA**

Tri_117880 301 **VGEGAAVASGISQASGRQTTTGQASGVASAEKEGTTTVQVAGRTSTGTEQTSGATAADLE**

idb_23862 248 **VGEGAAVASGISQASGRQTTTGQASGVASAEKEGTTTVQVAGRTSTGTEQTSGATAADLE**

Tri_117880 361 **GTAVQTAGVSAAGTAHASGASTSEVEGTTIVR**AEGTGQASGSTIPEIEGTTAQGAVSAAA

idb_23862 308 **GTAVQTAGVSAAGTAHASGASTSEVEGTTIVR**AEGTGQASGSTIPEIEGTTAQGAVSAAA

Tri_117880 421 EAGQASGAATVEAEGTSTVHAAGTGSTGTTQTSGSTTSQASGAATMEAEGTSTAQGAAGT

idb_23862 368 EAGQASGAATVEAEGTSTVHAAGTGSTGTTQTSGSTTSQASGAATMEAEGTSTAQGAAGT

Tri_117880 481 LAQTSGPTTTEVEGTNTIKETATEQTSVGTTTETEGMSTVQGTGSGPTGTAPAPALSTAQ

idb_23862 428 LAQTSGPTTTEVEGTNTIKETATEQTSVGTTTETEGMSTVQGTGSGPTGTAPAPALSTAQ

Tri_117880 541 VSGSSINEMEVSGTAGTTQASGTTTTAHASGSTTNEVEGTSTVQAVGTDTAGTAQASGTT

idb_23862 488 VSGSSINEMEVSGTAGTTQASGTTTTAHASGSTTNEVEGTSTVQAVGTGTTGTAQASGTT

Tri_117880 601 TAHAEVTGASTAEPAGAPTVQQTGPSTPEPTDSPTAGPTDASTAQPSGVFTAEPAGAPTV

idb_23862 548 TAHAEVTGASTAEPAGAPTVQPTGASTAEPTDSLTAGPTDASTAQPTGASTAEPAGAPTV

Tri_117880 661 QPTGASTAEPTDSPTAGPTDASMAQPTG------------------------

idb_23862 608 QPTGPFTAEPTNASTAQPTGASTTEPAGAPTVQPTGASTAEPTDSPTAGPTD

Tri_117880

**1 ADTTAAPTTEA**

**12 ADTTAAPTTEA**

**23 ADTTAAP-TIA**

**33 ADTTAAP-TAA**

**43 ADTTAA-RTEA**

**96 ADTTTAPSPA**

**106 ADTTPAPTAA**

**600 TTA-HAEVTGASTAEPAGAPTVQQTGPSTPEPTDSPTAGP**

**639 TDASTAQPSGVFTAEPAGAPTVQPTGASTAEPTDSPTAG**

Idb_23862

**43 ADTTTAPSPA**

**53 ADTTPAPTAA**

**554 TGASTAEPAGAPTVQPTGASTAEPTDSLTAGPTDASTAQP**

**594 TGASTAEPAGAPTVQPTG-----P---FTAEPTNASTAQP**

**626 TGASTTEPAGAPTVQPTGASTAEPTDSPTAGPTD**

**Figure S2M**

idb_25730

1 GISAMNIMLQRYARPGVPK**VAVVITDGMSKNPGATAQAAR**VSKLLGVNMFAVGVGLSTDI

61 MELQSIASGSDHVLTVAQFQQLDGLMSQLFKRVCPVQGGWTHWADSYGACSVFCGGGLQV

121 ITRTRTCTNPSPANGGLYCVGDALVTLTVECNTHGCPVDGGWSLWTDTVAQCSVTCGGGS

181 QLVTRTR**TCTNPAPAYNGQYCVGDDR**TTFTQACNTQGCPVDGGWSLWTDTESECSVSCGG

241 GTLVVTSTR**TCTNPAPAFNGQYCVGDDR**TTSTKACNTHGCPVDGGWSLWTDTVAQCSVSC

301 GGGTQLVTRTRTCTNPAPAYNGQYCVGDDRTTFTQACNTQGCPVDGAWTEWTEAAGACSL

361 TCGGGSQTVVRMR**SCTNPAPSSGGSDCVGEPR**EIVTRACNTQQCPVTTTEKPDLGLCNDC

421 K**MDNGIGYNPHPSDCNKYIQCMFDNNQLIKVEEMECPHGLYWDQDKLTCNRPEEVNCPAE**

481 **R**CNDPMTTTYPSATTCSGYWQCVGGVSKGRCCPAGQAYSIAGYCVPSTTCTTPCKGDGGQ

541 GACDKRPISGDRTRFQQFVKGHGWITMSCAPGSSYNSTECR**CSIQGGVVAPDTCKPDVYL**

601 **SFIDDVGDKSDSGVYIQNDGVK**VKNGVGYFDGKSGLRIPR**FANAELGSTVYIR**IRYRGEG

661 SATRK**QALLSNGDCGKDASISVARGPASTYFGVR**SGAGKSASVSVSSSTNGWNEAFFK**LD**

721 **AGMLSGSVGSSAKQTAFLGNIQRANCALQIGRGTNFANFRGYIDNVTIYLCRP**

**110 CSVFCGGGLQ--VITRTRTCTNPSPA-NGGLYCVGDALVTLTVECNTHGCPVDGGWSLWTDTVAQ-**

**172 CSVTCGGGSQL-V-TRTRTCTNPAPAYN-GQYCVGDDRTTFTQACNTQGCPVDGGWSLWTDTESE-**

**234 CSVSCGGG-TLVV-TSTRTCTNPAPAFN-GQYCVGDDRTTSTKACNTHGCPVDGGWSLWTDTVAQ-**

**295 CSVSCGGGTQL-V-TRTRTCTNPAPAYN-GQYCVGDDRTTFTQACNTQGCPVDGAWTEWT-EAAGA**

**357 CSLTCGGGSQT-V-VRMRSCTNPAP**

**Figure S2N**

idb_26568 1 MAQPGLIGTCLSVFLFTVAAGQICPNLSPNLQRPNMYFEKVHGKLMTR**KCAPGTVYHQSK**

idb_26567 1 MAQPGLIGTCLSVFLFTVAAGQICPNLSPNLQRPNMYFEKVHGKLMTR**KCAPGTVYHQSK**

idb_26568 61 **CTCDHGTSPNVVVPRVSGHASGASQGGYGGSNSQTR**RGGGSQNAQVGHVIEVNQGVQGTQ

idb_26567 61 **CTCDHGTSPNVVVPRVSGHASGASQGGYGGSNSQTR**RGGGSQNAQVGHVIEVNQGVQGTQ

idb_26568 121 RGGGQGYQGGQVAPGTPNSYNQHASNSAPGTWHTIETNPTMNIAGRNVPR**TDTVSSHSHI**

idb_26567 121 RGGGQGYQGGQVAPGTPNSYNQHASNSAPGTWHTIETNPTMNIAGRNVPR**TDTVSSHSHI**

idb_26568 181 **SAESRKSMNMMTALSKFIAQSHGHASTTHDSNPLRKVEQTANVRPVPQTSGQPSYQTRNQ**

idb_26567 181 **SAESRKSMNMMTALSKFIAQSHGHASTTHDSNPLRKVEQTANVRPVPQTSGQPSYQTRNQ**

idb_26568 241 **NPQGDPMHTAFHQHLFRSTLGDSKSEPLSVSPYLMKQQGLNSQPMTRAPLNTYTKKQSQS**

idb_26567 241 **NPQGDPMHTAFHQHLFRSTLGDSKSEPLSVSPYLMKQQGLNSQPMTRAPLNTYTKKQSQS**

idb_26568 301 **SIETQTRIQSHVNPQSTNQKLITAQYVNQPDMNAQPIDK**TPANVYQINQPSTQQFNQPQI

idb_26567 301 **SIETQTRIQSHVNPQSTNQKLITAQYVNQPDMNAQPIDK**TPANVYQINQPSTQQFNQPQI

idb_26568 361 NTLPVNPPPTNAQTMDPSPLNSQVIDK**MSLNEHQVNQPSRNSQSFHQPRINTQPVNQQQQ**

idb_26567 361 NTLPVNPPPTNAQTMDPSPLNSQVIDK**MSLNEHQVNQPSRNSQSFHQPRINTQPVNQQQQ**

idb_26568 421 **INSQSIDLSPINSK**LGHKSATNVYQINQQSSNSQSLLQPQINIQPVNQQQTNSQPINLSP

idb_26567 421 **INSQSIDLSPINSK**LGHKSATNVYQINQQSSNSQSLLQPQINIQPVNQQQTNSQPINLSP

idb_26568 481 MNSQLGHKSSTNVYQINQPSFHQPQINTQPVNQQQTNSQPINLSPMNSQLGHK**SATNVYQ**

idb_26567 481 MNSQLGHKSSTNVYQINQPSFHQPQINTQPVNQQQTNSQPINLSPMNSQLGHK**SATNVYQ**

idb_26568 541 **INQPSSNSQSFHQPQINSLSVNQQQTKSQPADLSPM**N**SQLSGK**-----------------

idb_26567 541 **INQPSSNSQSFHQPQINSLSVNQQQTKSQPADLSPMNSQLSGK**ESMNVYQSNQPSSNSQS

idb_26568 584 ---------------------------------**ESMNVYQSNQPSSNSQPFNQPQINPQP**

idb_26567 601 FHKPQINSLSVNQQQTKSQPADLSPMHSQLSGK**ESMNVYQSNQPSSNSQPFNQPQINPQP**

idb_26568 611 **MKQQQTNSQPVDLSPMNSQLGHKSSTNVYQINQPSSNSQSFHQPQMKSISMNQPDMNSHP**

idb_26567 661 **MKQQQTNSQPVDLSPMNSQLGHKSSTNVYQINQPSSNSQSFHQPQMKSISMNQPDMNSHP**

idb_26568 671 **ENK**STVR**SFSIQHNSLNVQSGNR**QPITSQSMNPTHTNLLPNNNLPMVSFPMNQNPTSPQS

idb_26567 721 ENKSTVRSFSIQHNSLNVQSGNRQPITSQSMNPTHTNLLPNNNLPMVSFPMNQNPTSPQS

idb_26568 731 SNQQPIISQSMNPPHVNLLPNNQPPINTYPR**NQNAMNAQSGNQQPHNSQSVNK**LNMNSQT

idb_26567 781 SNQQPIISQSMNPPHVNLLPNNQPPINTYPR**NQNAMNAQSGNQQPHNSQSVNK**LNMNSQT

idb_26568 791 NNNIPMNSFPINQNSVSSQSANQQTIISQSMNPRHMNFLPNMNSPMSSFPINQDFLNSQS

idb_26567 841 NNNIPMNSFPINQNSVSSQSANQQTIISQSMNPRHMNFLPNMNSPMSSFPINQDFLNSQS

idb_26568 851 GNQQTINSQSINPPHMNLLPNHQSSMHSYPKTQNSINSHSMNHQQMNLQSVNQNPVNSQQ

idb_26567 901 GNQQTINSQSINPPHMNLLPNHQSSMHSYPKTQNSINSHSMNHQQMNLQSVNQNPVNSQQ

idb_26568 911 MNYVQINSLPMNTDPRNQAPMYPQPMNSQSASQPLNHPQLSLPPLHQPPLNLPSHQPPML

idb_26567 961 MNYVQINSLPMNTDPRNQAPMYPQPMNSQSASQPLNHPQLSLPPLHQPPLNLPSHQPPML

idb_26568 971 HGQSSGAPMVISGWTPDNRLSWR**ENLHMADMMDAPDFQR**DAFIR**NVLMNEPSLTPVDRME**

idb_26567 1021 HGQSSGAPMVISGWTPDNRLSWR**ENLHMADMMDAPDFQR**DAFIR**NVLMNEPSLTPVDRME**

idb_26568 1031 **LNQLTI**

idb_26567 1081 **LNQLTI**

idb_26568

**396 NQPSRNSQSFHQPRINTQPVNQQQQINSQSIDLSPINSKL-GHKSATNVYQI**

**447 NQQSSNSQSLLQPQINIQPVN-QQQTNSQPINLSPMNSQL-GHKSSTNVYQI**

**497 NQP-----SFHQPQINTQPVN-QQQTNSQPINLSPMNSQL-GHKSATNVYQI**

**542 NQPSSNSQSFHQPQINSLSVN-QQQTKSQPADLSPMNSQLSG-KESMNVYQS**

**592 NQPSSNSQPFNQPQINPQPMK-QQQTNSQPVDLSPMNSQL-GHKSSTNVYQI**

**642 NQPSSNSQSFHQPQ**

idb_26567

**396 NQPSRNSQSFHQPRINTQPVNQQQQINSQSIDLSPINSKL-GHKSATNVYQI**

**447 NQQSSNSQSLLQPQINIQPVN-QQQTNSQPINLSPMNSQL-GHKSSTNVYQI**

**497 NQP-----SFHQPQINTQPVN-QQQTNSQPINLSPMNSQL-GHKSATNVYQI**

**542 NQPSSNSQSFHQPQINSLSVN-QQQTKSQPADLSPMNSQLSG-KESMNVYQS**

**592 NQPSSNSQSFHKPQINSLSVN-QQQTKSQPADLSPMHSQLSG-KESMNVYQS**

**642 NQPSSNSQPFNQPQINPQPMK-QQQTNSQPVDLSPMNSQL-GHKSSTNVYQI**

**692 NQPSSNSQSFHQPQ**

**Figure S2O**

Idb_26836

1 LNTVPVSAAGSQTSDPVSGNTVHNILPFNEQSGAASQTRTQGAARITSQEIVPVSGINFH

61 VPINGQSYTVGTSAVPVIETSSLDIIPLNRQSYTAGATSQGTAPSSGSSVQDNSHLDGQS

121 HTAGTTSQGAAPTSESSVQDNSHLDRQPNTAGGTTLGAAPTSGSSVQDNSHVDGQSHTVG

181 MTINNSVPVSATTIQDAIPVTGLK**QDVVQAPGSGPSTEVVHFDITGTNMPAQVYKQMPVE**

241 **NIATTKVANSVTGTHTEPGVDTNINK**SLWRIP

**94 TAGATSQGTAPSSGSSVQDNSHLDGQSH**

**122 TAGTTSQGAAPTSESSVQDNSHLDRQPN**

**150 TAGGTTLGAAPTSGSSVQDNSHVDGQSH**

**Figure S2P**

idb_27355

1 LICDFRHFADIMSPRSHAVFGRTLVLILCFSLGQMQQQSQAEVEAQEVAQGQGPIVYHAR

61 IRPGQANQGANGATVVTEAPDVEAPEVAEGQTAPLVVQTGGPNSPGGAVSTYPPNYEYEA

121 PETPEVTSPLPTTGPSVPETPPQTVVSVSR**RQPSYIQQQARGTAGFVSASGQSTPATSAG**

181 **QQHRHKILTQATNAAKSHLMRNLFEQFSKMAEYHQFDMQKMRAAMQQSASPSVSGQRGAP**

241 **IYDTGLGSRQTSTSGQSGASVYDAGQGGMQTSTSGQSGASVYDAGQGSR**QTSTSGQSRTS

301 VYDAGQGSVQTSTSGQSGASVYDAGLGSVQTSTSGQSGASVYDAGQGSLPTSTPSPIWVP

361 IYLAGRWTRQKYNPEVPVYAGGQGNIQTSTPNQIGYESSTGTQPPVTAAPTSSQPLEQTT

421 ATFQTGAQYSYVTGAQTPVNGRTGAQTTTSAPLQVGAQTLIEHLAEVYPISK**THVSGQAT**

481 **AQTAPAVGQDRSPVTDQTPVAIKEIVPVKTVENSVNAKLSHPIEPRLSPVPNTDPYTGTG**

541 **NMLKPITNAEFGAIMQR**MLVQR**LRKYISSQRKFQAYNPEP**

**165 GLGSRQTSTSGQSGASV--YDA**

**185 GQGGMQTSTSGQSGASV--YDA**

**205 GQGSRQTSTSGQSRTSV--YDA**

**225 GQGSVQTSTSGQSGASV--YDA**

**245 GLGSVQTSTSGQSGASV--YDA**

**265 GQGSLPTST--PSPIWVPIYLA**

**Figure S2Q**

idb_27864

1 NVAGTQQASTANVAGTQQASTTNVAGTQQASTANVAGTQQASTANVAGTQQASTTNYAST

61 QQAAAGSNDVTQQASTTNVAGTQQASTTNYASTTQVVAGSNDATQQASTSNVAGTQQTST

121 ANVAGTQQASTTNYASAQQAAASSNDAK**QQVSTTNVAGTQQGSTTNVAGK**QQASTTNNAS

181 TQQAAAGSNDATQQASTTNYASTQQAAAGSNDPTQQGAASSKDATQQASTTNVAATQQTS

241 TTNVAGTQQASTINVAGTQQGPTTNYASAQQAAAGSNDVTQQASTTNVAGTQQASTTNYA

301 STQQAAAGSNDATQQASTTNNAGTQQAATTTYASALSSYDATQQAATTNFASAKPTALSS

361 NDPTALANTVSVSNTLSDTASAATNLDAGTPTGSGANSLYIESTR**GAAANSGSNTLSTFD**

421 **R**LNNANANVDAVSGSTQTTNGASLAAAGTNSVSGTIGSTVSGQYQVPSRATVPSNS

**1 N-VAGTQQASTANVAGTQQASTTNVAGTQQASTA--**

**34 N-VAGTQQASTANVAGTQQASTTNYASTQQA-AAGS**

**68 NDV--TQQASTTNVAGTQQASTTNYASTTQV-VAGS**

**101 N-DA-TQQASTSNVAGTQQTSTANVAGTQQAS**

**71 TQQASTTNVAGTQQASTTNYASTTQVVAGSNDATQQASTSNVAG**

**115 TQQTSTANVAGTQQASTTNYASAQQAAASSNDAKQQVSTTNVAG**

**159 TQQGSTTNVAGKQQASTTNNASTQQAAAGSNDATQQAST**

**224 ATQQASTTNVA**

**235 ATQQTSTTNVA**

**246 GTQQASTINVA**

**257 GTQQ**

**247 TQQASTINVAGTQQGPTTNYASAQQAAAGSNDV**

**280 TQQASTTNVAGTQQASTTNYASTQQAAAGSNDA**

**313 TQQASTTNNAGTQQAATTTYASA**

**Figure S2R**

idb_27866

1 SQQPTSGSNTVLNIGVTATR**DIAPNPEAGVGSNVIANSVSGVRTNTLISYTGKLLASRNN**

61 **DGTSSGTANSIGSPTSLSNTVDNTDTVSATRTTAPTAEPETSSTTSAKATPSVAATANIG**

121 **SGSGSTGPTAVPETASTTSAK**ATPSVAATSTNVGGSGSTGPTAEPETNTAQQTGAPTSAP

181 SNTGSYANTAQQTGAPTSAPSNTGSYANIAQQTGASTSAPSNPGSYINTAQQTGAPTSAP

241 SNTGSYANTAQQTGASTSVPSNTGSYINTAQQTGAPTSVPSNTGSYINTAQQTGASTSVP

301 SNTGSYINTADQTGAPTSAPSNTGSYINTAQQTGAPTSAPSNTGSYINTAQQTGAPTSVP

361 SNT

**95 PTAEPETSSTTSAKATPSVAAT-ANIGSGSGSTG**

**128 PTAVPETASTTSAKATPSVAATSTNVG-GSGSTG**

**161 PTAEPET**

**168 NTAQQTGAPTSAPSNTGSYA**

**188 NTAQQTGAPTSAPSNTGSYA**

**208 NIAQQTGASTSAPSNPGSYI**

**228 NTAQQTGAPTSAPSNTGSYA**

**248 NTAQQTGASTSVPSNTGSYI**

**268 NTAQQTGAPTSVPSNTGSYI**

**288 NTAQQTGASTSVPSNTGSYI**

**308 NTADQTGAPTSAPSNTGSYI**

**328 NTAQQTGAPTSAPSNTGSYI**

**348 NTAQQTGAPTSVPSNT**

**Figure S2S**

idb_32603 1 MTSLFVSTVLLVLASRDVIVGMPTTSWQPGGFFAMSHGTNTGGNAQQSAGQLAQTQAEKT

idb_32602 1 MTSLFVSTVLLVLASRDVIVGMPTTSWQPGGFFAMSHGTNTGGNAQQSAGQLAQTQAEKT

idb_32603 61 TSQPGNGTK**TNDAQPGTMQQSNAAKLAMMQQAAARYEAMMQQAR**SNFNAMNSQIQAHMQG

idb_32602 61 TSQPGNGTK**TNDAQPGTMQQSNAAKLAMMQQAAARYEAMMQQAR**SNFNAMNSQIQAHMQG

idb_32603 121 SMGGPPTQQPGQFNKHTTFSTMSPPAPTPDVTLPAQSTVNTGSSNPRTSASQGSNPAPTA

idb_32602 121 SMGGPPTQQPGQFNKHTTFSTMSPPAPTPDVTLPAQSTVNTGSSNPRTSASQGSNPAPTA

idb_32603 181 SPVRGTSPTVHLPTSQPPNQQEYIPDQPGQQFQSSPSSQPSQPSQSSQPVPPTQPTQPAQ

idb_32602 181 SPVRGTSPTVHLPTSQPPNQQEYIPDQPGQQFQSSPSSQPSQPSQSSQPVPPTQPTQPAQ

idb_32603 241 PTQPPQPVQPVQPVQPVQPVQPVQPVQPVQPSQPVPSAPTAGPAAAISPQEAAMR**AQRMV**

idb_32602 241 PTQPPQPVQPVQPVQPVQPVQPVQPVQPVQPSQPVPSAPTAGPAAAISPQEAAMR**AQRMV**

idb_32603 301 **YQMEAQMMGQRNQQQGFVPDRQVPLPNNPPVQHQWPQQQMTAFGR**QDMPIQAHQSSGQWP

idb_32602 301 **YQMEAQMMGQRNQQQGFVPDRQVPLPNNPPVQHQWPQQQMTAFGR**QDMPIQAHQSSGQWP

idb_32603 361 QSQQSLTTANQGFPDPTNMQNQQPYAMGQMNGR**MHAPAPAMADPAR**MGNMHGMNGRTDTS

idb_32602 361 QSQQSLTTANQGFPDPTNMQNQQPYAMGQMNGR**MHAPAPAMADPAR**MGNMHGMNGRTDTS

idb_32603 421 MFNMNNQQQQQQQPPQMTLEQQYLQMLQQQSQLPPSSPFGQQGQSTASSLFGQQSTSAFS

idb_32602 421 MFNMNNQQQQQQQPPQMTLEQQYLQMLQQQSQLPPSSPFGQQGQSTASSLFGQQSTSAFS

idb_32603 481 HQSPSTHSHFLNQR**QSPSTSPFMPDMSRQQPGAGLGGPASAPTQAQLLQQFAALDSKLSS**

idb_32602 481 HQSPSTHSHFLNQR**QSPSTSPFMPDMSRQQPGAGLGGPASAPTQAQLLQQFAALDSKLSS**

idb_32603 541 **MPNPTPAQLQSVMSQFQQLER**QMEMANNPASPSSALTPDMSGMGGMPGMPGTPDMSGMGG

idb_32602 541 **MPNPTPAQLQSVMSQFQQLER**QMEMANNPASPSSALTPDMSGMGGMPGMPGTPDMSGMGG

idb_32603 601 IPGMPGMPGMPGMPDMSGMGGIPGMPGMPGMPGMPDMSGMAGMPLVPSAAMGPDSIPAIE

idb_32602 601 IPGMPGMPGMPGMPDMSGMGGIPGMPGMPGMPGMPDMSGMAGMPLVPSAAMGPDSIPAIE

idb_32603 661 TQVMALENR**LLTHFKYPNPDPAQTAQMVAEYDVLQKNLK**AAK**RAAAVSSSPMPGGSSSAS**

idb_32602 661 TQVMALENR**LLTHFKYPNPDPAQTAQMVAEYDVLQKNLK**AAK**RAAAVSSSPMPGGSSSAS**

idb_32603 721 **QLSVGNTALNSRK**ALSSMNNPFDFTGLGSSASGNSSPLPSLVSTPQGLTPADINTLPSPG

idb_32602 721 **QLSVGNTALNSRK**ALSSMNNPFDFTGLGSSASGNSSPLPSLVSTPQGLTPADINTLPSPG

idb_32603 781 GLQAAPVLPGLPGTATLEPGAK**DILEPMFER**REDISKYTQQDTLHIPVLTYTRLC-----

idb_32602 781 GLQAAPVLPGLPGTATLEPGAK**DILEPMFERREDINKAR**KLAATMSFIQQITDPSSGSSM

idb_32603 ------------------------------------------------------------

idb_32602 841 GPMEPMNGGNNVFPGTGQSSQPAPSSGGSGASSMSSLGMHSGQLPGGFFPGAASQSLLHS

idb_32603 -------------------------------------

idb_32602 901 PMPDIFPQSSAADPQMQQFLQSFQPPAGMSPFFGGAR

**218 SQP**

**221 SQP**

**224 SQS**

**227 SQP**

**232 PTQ**

**235 PTQ**

**238 PAQ**

**241 PTQ**

**246 QPV**

**249 QPV**

**252 QPV**

**255 QPV**

**258 QPV**

**261 QPV**

**264 QPV**

**267 QPV**

**270 QPS**

**273 QPV**

**578 PDMSGM----G--GMPGMPGT**

**593 PDMSGMGGIPGMPGMPGMPGM**

**614 PDMSGMGGIPGMPGMPGMPGM**

**635 PDMSGM**

**Figure S2T**

idb_36583

1 QSGTGSVHTGTLGSTMNHAVSTDQSSSVQQSGTGSVNTGTLGSTMNHAVSTDQSSSTHQS

61 GTGSVHTGTLGSTMNHAVSTDQSSSVQQSGTDSVHTGTLGSTMNHAVSTDQSSSTTAHRG

121 SATSTGGAIDSTSDPNHVYAVPPNTNSVPDKTSIVK**DITGFNADNTIVVGSLYKPENTKT**

181 **GGDSVVTDLNGQK**HVVWSPNDWHASSSGTVGETHTHSHTHSYIQTDTVGTAQNTGNVFST

241 SAIIPVQLAR**APLPSHLSQNEVSSDKSVVGEK**ASASSGSGAVSGSGSATVSYPTTVSAST

301 SGSSTTTTSKLTK**GSGTTIASSSTIGSVTGSSSTSNK**SSSSSSSGSTNSATGTSSTDSFG

361 STTGSGLTSSSGTSSGSNSETGSITSASPGLSGSVR**GSVSTDKFNMASGSSSIR**NSGATS

421 GSGPETGSSSTSGSSSISGSDTTIASNSATK**SVSTSGSGSTTR**SSTSIGSGSHSGSNSMA

481 GSISTIGSSSTNGSSSTTGSSSNSGSVSATGSSPNSGSVSTTGSSSTSSSASTTRSSSNS

541 GSASTTGFSSNSGSASITGSGYTSGSASTTGFGSTSGSVSITGSSSTSGSDSATGSSSTR

601 GSDSATGFSSNSGSASITGSGSTSGSASTTGSGSTSGSASTTGSSSISGSASTTGTSSNS

661 GSASSSGSSSTTGSGSPTGAGSLSTSGSTAGSGTFSSSGSTTLGSSRSNGPLNG

**1 QSGTGSVHTGTLGSTMNHAVSTDQSSSVQ**

**30 QSGTGSVNTGTLGSTMNHAVSTDQSSSTH**

**59 QSGTGSVHTGTLGSTMNHAVSTDQSSSVQ**

**88 QSGTDSVHTGTLGSTMNHAVSTDQSSST**

**427 GSSSTS**

**433 GSSSIS**

**440 GS**

**454 STS-GSGSTTRS-**

**464 STSIGSGS—HSG**

**496 STTGSSSNSGSVSATGSSPNSGSV**

**520 STTGSSSTSSSASTTRSSSNSGSA**

**544 STTGFSSNSGSASITGSGYTSGSA**

**568 STTGFGSTSGSVSITGSSSTSGSD**

**592 SATGSSSTRGSDSATGFSSNSGSA**

**616 SITGS**

**563 TSGSAS-T-TGFGS**

**575 TSGSVS-I-TGSSS**

**587 TSGSDS-A-TGSSS**

**599 TRGSDS-A-TGFSS**

**611 NSGSAS-I-TGSGS**

**623 TSGSAS-T-TGSGS**

**635 TSGSAS-T-TGSSS**

**647 ISGSAS-T-TGTSS**

**659 NSGSAS-S-SGSSS**

**671 TTGSGSPTGAGSLS**

**686 TSGS**

**680 AGS--LSTSGST**

**690 AGSGTFSSSGST**

**Figure S2U**

Idb_4071

1 LDTIVDTRTMVGIVVWALVSTCLTGMVDAQGGLPSQSMQHQWPQAAPVGGEGHTASPPGQ

61 PSYGAPPPDLPDSLGGLPPNNPQNQGGWQSEQSRAWPK**YPNSPNPGGWDPPSTSTGGGSR**

121 **TGWQPPADAPSQGGGR**WQSPHHHQSPPWQPPPNSPNQVWQPPHDGPPPGWHPSPDKPPQP

181 WQPQPDASSQQLPSGKSPQPWQPPPDKPNQPWQPPPDGAPPGWQPPPDKPPQPWQPPSDG

241 APPGWQPPPDKPPQPWQPPPDGAPSGWQPPPDKPPQPWQPPPDGAPPGWQPPPDKQPQPW

301 QPTPDGAPSGWQPPPDKPPQPWQPPPDGAPSGWQPPPDKPPQPWQPPPDGPPPGWQPPPD

361 QNLQPWQPPPDGPPPGWQPPPDQPPHPWQSPQDGPPPGWQPPADGATSGWQPPPDKPPPG

421 WEPSPNSQTAGANQQPSPYQQPVPAAAQPWGAQNGPAWQQGSPTADPADTPAQPGGGSHW

481 QGGYPDIPNNFFAPPSSNQGGR**NPAPPAGAVRDYPNTYPNQPQPQPQPQPQPQQRPQPRP**

541 **QQPPQQVRPKPQPQPQPKPQPVQR**PQVPRPYQPPQQPYQSQPPNNTFGYNSYNMPPAAPM

601 PR**PPPGPAFVAWQKTQPCVMNPTIPDLCRYNGDGLHVYRLFDVIEITKTLPRVPSCYAGS**

661 **PRADCTQTLQAMHLWNR**DFCRCK**NNPTAECILNDCGGVCSNEFYIGTTR**VLCE

**198 PQP-WQPPPDK-P**

**209 NQP-WQPPPDGAP**

**221 P-G-WQPPPDK-P**

**231 PQP-WQPPSDGAP**

**243 P-G-WQPPPDK-P**

**253 PQP-WQPPPDG-A**

**264 PSG-WQPPPDK-P**

**275 PQP-WQPPPDGAP**

**287 P-G-WQPPPDK-Q**

**297 PQP-WQPTPDG-A**

**308 PSG-WQPPPDK-P**

**319 PQP-WQPPPDG-A**

**330 PSG-WQPPPDK-P**

**341 PQP-WQPPPDG-P**

**352 P-PGWQPPPDQ-N**

**363 LQP-WQPPPDG-P**

**374 P-PGWQPPPDQ-P**

**386 PHP-WQSPQDG-P**

**aa521-532: [QP]_6_**

**532 PQQR**

**536 PQPR**

**540 PQQP**

**545 PQQ**

**aa521-560: [PQ]_4_ and one [PK]**

**Figure S2V**

idb_43368

1 AQAQAQAQSQGGIQVLPGPPGSAMQVNQAGNTNLEAAALAMTEAAAGVETAPSAAEQPNV

61 DGFSNFFNK**PTFLDAQVDPEIKANVNINSVEPVK**GAQAETNLLGSFGIANNSPPWVGFPM

121 MDDAGEGAQSIVDVNAAGGGSAIGSAQAAVGSQAAVNVISQEGAQVLGSAQATGASQAIS

181 NVVAADGGYAVGSSQAEGGSQASANVAAAEGAQAIGSAQAQGGSQSISNTVAAEGGNAVG

241 LAQASAASQALADVVAAQGAQASGSSQAMGGSQSLANVLAAEEAQAAAIAQAIAASQAAA

301 DVVAAQGAQASGSSQAMGGSQSLATVLGAEDAQAMGMAQAIAASQAAADVVAAQGAQASG

361 SSQAMGGSQSLATVLGAEDAQAMGMAQAIAASQAAADVVAAQGAQASGSSLAMGGSQSLA

421 NVLAAEEAQAAAIAQAIAASQAAADVVAAQGAQASGSSLAMGG

**168 GSAQATGASQAISNVVAADGGYAVGSSQAEGGSQASANVAAAEGAQAI**

**216 GSAQAQGGSQSISNTVAAEGGNAVGLAQASAASQALADVVAAQGAQAS**

**264 GSSQAMGGSQS**

**242 AQASAASQALADVVAAQGAQASGSSQAMGGSQSLANVLAAEEAQAAAI**

**290 AQAIAASQAAADVVAAQGAQASGSSQAMGGSQSLATVLGAEDAQAMGM**

**338 AQAIAASQAAADVVAAQGAQASGSSQAMGGSQSLATVLGAEDAQAMGM**

**386 AQAIAASQAAADVVAAQGAQASGSSLAMGGSQSLANVLAAEEAQAAAI**

**434 AQAIAASQAAADVVAAQGAQASGSSLAMGG**

**Figure S2W**

Idb_44689

1 DSATGSSSTSGSASTTGSSSTSGSASTTGSSSTSGSASTTGSSSTSGSASTTGSSSTSGS

61 ASTTGSSSNSGSASTTGSSSTSGSASITGSSSNSGSASTTGSSFNSGSASTTGTSSNSGS

121 ASSSGSSSTTGSGSPTGAGSLSTSGSTAGSGTFSSSGSTTLGSSRSNGPLNGPWSDMNSI

181 FGFMPIIAGR**HDSSGASVFSNDISNAPVPHPQSAANTKVVNTVSIEGLAQLNSMANPSDL**

241 **VASEMSHIPVVPTPDRLSMMPNVNKDPAYNLTPLSSATSSSSSTNQRASIHSTASQISSS**

301 **KQTAHASK**TDTSAVNGDTTSAASGSASSQSSSTSSQGGASSSQSGIVPSQSGTASSQSGS

361 TSQSGIVSSQGELTSHSGLTSQSGSTSQSGMASQSSSTSHSGLTSQSGSTSQSGMASQSA

421 STSQSGMTSQSGMTSQSGSTSQSGMTSQSGSASQSGMTSQSGSTSQSGMTSQSGMTSQSG

481 SASHSGMTSQSGSTSQIGITSSQSSSTSQSGMTSSQSGSASQSDLISTSSQSDLTPSSSP

541 FEINSK**QTSLTDSEQSVYVQPSKTDKNNNLKDVSGANVDNVIHPGIVLKDVKQGKYEQNF**

601 **GTSSGMAGTSSLQNTQTAASHTDLRTSGSNSVLNANQPPTGTAPDTSK**ITLTPTFEADSG

661 SVAVTHTALVGPTAGGSGHTNSPLSSDTTSATTSASEPVLNAQSQGGPEVVYASPSHISS

721 LLYSSAASVLKDGSTAHPSLSTVAPEQALNTASGPSVTAIDVAKPGASPNYDATQQAATG

781 SNDGTQVSTTNVASTQQASTINVAGTQQTSTTNVAGTQQASTTNYASTQQAAAGSNDVTQ

841 QASTTNVAGTQQASTANVAGTQQASTTNVAGTQQASTANVAGTQQASTANVAGTQQAST

**2 S-A-TGSSSTSGSA--**

**14 S-T-TGSSSTSGSA--**

**26 S-T-TGSSSTSGSA--**

**38 S-T-TGSSSTSGSA--**

**50 S-T-TGSSSTSGSA--**

**62 S-T-TGSSSNSGSA--**

**74 S-T-TGSSSTSGSA--**

**86 S-I-TGSSSNSGSA--**

**98 S-T-TGSSFNSGSA--**

**110 S-T-TGTSSNSGSA--**

**122 S-S-SGSSSTTGSG--**

**134 SPTGAGSLSTSGSTAG**

**150 S-G-TFSSSGS**

**341 S-SQSGIV**

**348 P-SQSGTA**

**355 S-SQSG--**

**360 STSQSGIV**

**368 S-SQ**

**404 TSQSGS**

**410 TSQSGM**

**416 ASQSAS**

**422 TSQSGM**

**428 TSQSGM**

**434 TSQSGS**

**440 TSQSGM**

**446 TSQSGS**

**452 ASQSGM**

**458 TSQSGS**

**464 TSQSGM**

**470 TSQSGM**

**476 TSQSG**

**489 SQSGSTSQIGITS**

**503 SQSSSTSQSGMTS**

**516 SQSGS**

**774 TQQAATGSNDGT-QVSTTNVASTQQASTINVAGTQQTSTTNVAGTQQASTTNYAS**

**827 TQQAAAGSNDVTQQASTTNVAGTQQASTANVAGTQQASTTNVAGTQQASTANVAG**

**861 TQQA**

**Figure S2X**

Idb_47306

1 MPLLPAIVVVLCSVCTTSAQTGECCAKNCCTYK**CCQGHGLANK**LAADPASTGNIGVGGSD

61 PTLGTGSTTAFFYDTAVGPNAIGGGYSSHGAGGTPTGLFPYPAEVVNYLDLSATSSLGSA

121 R**ADAVAAAAENAERQAEAINEAATGFR**NKLERAGMSSDLQIAVFGGGLGGHGVGGSSSGS

181 GSAMNTLVQFGGGYQAGVPVQAQPASQNGVAAGAQAETLTTSQAQAQAQAQAQAQAQAQA

241 QAQAQAQAQAQAQAQAQAQAQAQAQAQAQAQAQAQAQVQTQAQAQTQAQAQAQAQAQAQA

301 QAQAQAQVQTQAQAQ

**aa223-315: [QA]_41_ and [QV/T]_5_**

**Figure S2Y**

Idb_51205

1 AGTQQASTANVAGTQQASTANVAGTQQASTTNYASTKQAAAGSNDVTQQASTTNVAGTQQ

61 ASTTNVAGTQQGPTTNYVSAQQAAAGSNDVTQQASTANVAGTQQASTTNYASTK**QAVGGS**

121 **NDVTQQAVATSK**DATEQASTTNVAGTQQASTINVAGTEQASSTNVAGTQQASTANVAGTQ

181 QASTTNYASTQQAAASSNDATQQASTTNVAGTQQASTTNYASTQQAAAGSNDVTQQASTT

241 NVAGTQQASTANVAGTQQASTTNYASTQQAAAGSNDATKQASTTNVAGTQQASTANVAGT

301 QQASTTNVAGTQQASTTNYASTQQAAAGSNDVTQQASTANVAGTEQASTTNFAGTQQAST

361 TNVAGTQQASTANVAGTQQASTTNVAGT

**2 TQQASTANVAG---------------------------------------------**

**14 TQQASTANVAGTQQAS-----------TTNYASTKQAAAGS-ND-----V------**

**47 TQQASTTNVAGTQQASTTNVAGTQQGPTTNYVSAQQAAAGS-NDV-----------**

**91 TQQASTANVAGTQQAS-----------TTNYASTKQAVGGS-NDVTQQAVATSKDA**

**135 TEQASTTNVAGTQQAS-----------TINVAGTEQ--ASSTN------VAG----**

**168 TQQASTANVAGTQQAS-----------TTNYASTQQAAASS-ND-----A------**

**201 TQQASTTNVAGTQQAS-----------TTNYASTQQAAAGS-ND-----V------**

**234 TQQASTTNVAGTQQAS-----------TANVAGTQQA**

**137 QASTTNVAGTQQASTINVAGTEQ--ASSTN-VAGTQQASTANVAGTQQASTTNYASTQQAAASSNDATQ**

**203 QASTTNVAGTQQASTTNYASTQQAAAGS-NDV--T-Q----------QASTTNVAGTQ-----------**

**247 QASTANVAGTQQASTTNYASTQQAAAGS-NDA--TKQASTTNVAGTQQASTANVAGTQ-----------**

**302 QASTTNVAGTQQASTTNYASTQQAAAGS-NDV--TQQASTANVAGTEQASTTNFAGTQ-----------**

**357 QASTTNVAGTQQASTANVAGTQQA**

**Figure S2Z**

Tri_108584

1 MGLLPILIGLLAIQVTWLGFSDGAVPSTVK**YHRPHGPAAGVPASSGIPPVKPLPRNVGPT**

61 **STPATAKALTPAASVTASVTSSAKAKASAAKEDRYLHSLTKITTFMVKLNSFLELTIGSI**

121 **TKQLTDLTSQMAEVR**GSLENLVNNTNTQPSIPSIPPPVPVLPVLNPPTTPQPTPPPPPPP

181 PSAPR**PKPKPIHVQALKPAPRPAPQPAPRPAPRPSRPTAVRQPIQRYMPSYPMQFKPAVV**

241 **PTARKPAITQQRRMDYYHGSGDKSLRWPGNNEFHPWWKNWWSNNFQGFYPETSGDTSSTS**

301 **SFGSRRSKFGSPNQFGSQFGGQSSFGDTSQFGARSQFGSVPQFGGSKFSATSQFGSVPQY**

361 **GGSQFGSFGSYPGGTAAAWMSASR**K**SREPGETEGKGR**SSGSAAEYR

**198 PAPR**

**202 PAPQ**

**206 PAPR**

**210 PAPR**

**335 SQFGSVPQFGGSKF-SAT**

**353 SQFGSVPQYGGSQFGS-F**

**Figure S2Za**

Tri_29101

1 TSSASGTGASTSSATGTSAGTALATGTSASTSSAIGTGANTTSTGTAGAVTATATGTSTS

61 TSSASGTGASTSSATGTGASTSSATGTGASTTSTGTTGAINDSASPASETSEANKGQKVD

121 TK**GVGTTSVTEATNKQLDTVIPEIQNTQLSLEQIK**QLVKIYK**LRPSNYGRFLEALQWQSS**

181 **K**TVRTMDTRRRVITSVAATYQVVLNGGDCVPWNSFPFDR**TLTWQGGRATIHFFNPDGRYA**

241 **ELCIGNPLTEADK**SCR**FQVVIPNDIITAIDTPGFVVYNRIMHIQHQGDVHTIDNMLSR**GA

301 EFVK**RQETIQEFASCYN**

**1 TSSASGTGASTSSATGTSAGTALATGTSASTSSAIGTGANTTSTGTAGAV**

**51 TATATGTSTSTSSASGTGASTSSATGTGASTSSATGTGASTTSTGTTGAI**

**Figure S2Zb**

CLC_5 ------------------------------------------------------------

Tri_57798 1 DDDDDDDDYDYDDDDDDDDDDDDDDDDDDDDDYDYDDDDDDDDDDDDDDDDDDDDGAILN

CLC_5 1 ----------------------------------------------**ASGFGGAGGAAGGF**

Tri_57798 61 LLSTVVSLLTSVLSGGAGGAGGGAGAGGAGGAGGAGGAGGAGGAGGASGFGGAGGAAGGF

CLC_5 15 **GAGGAGGRGGGFGFGSASARASADAAARAFGGGFGGFGSSSASASADASASANIAALLGQ**

Tri_57798 121 GAGGAGGR**GGGFGFGSASARASADAAARAFGGGFGGFGSSSASASADASASANIAALLGQ**

CLC_5 75 **FAEASAR**ASASAAANAGAGGFNGGFGSSSASADASASASASGFGGSGGSGGSGGSRGSR**R**

Tri_57798 181 **FAEASAR**ASASAAANAGAGGFNGGFGSSSASADASASASASGFGGSGGSGGSGGSRGSR**R**

CLC_5 135 **SFGSGGFGR**FGDDNGWGYDNDDDCGDGENGNGNGNGNGNGNGYRSEER------------

Tri_57798 241 **SFGSGGFGR**FGDDNGWGYDNDDDCGDGENGNGNGNGNGNGNGNGNGNGNGNGNGNGNGNG

CLC_5 ------------------------------------------------------------

Tri_57798 301 NGNGYDDDDDWDDFDWDDDDWNDNDNGDNGDDDDWDDWDDDDR**FDDDRFDDDRWDDDRFD**

CLC_5 ------------------------------------------------------------

Tri_57798 361 **DDR**WDDDNDDWDDDDPWGDDDNDDWDNDDLWGDDDNDDLDDDDGFEGADNVYWDDDDPWG

CLC_5 ------------------------------------------------------------

Tri_57798 421 DNGNDDNGNGDDDDDNGGYAFLRRALARASARARAAASAAGRSRGGSGR**SGGSGGSGGSG**

CLC_5 ------------------------------------------------------------

Tri_57798 481 **GSGGSGGSGGSAR**ARASASASARASSGSGGK**GGYYGKGGYYGK**GGYYGKGGYYGKGGYYG

Tri_57798:

**1 DDDDDDDDYDYDDDDDDDDDDDDD**

**25 DDDDDDDDYDYDDDDDDDDDDDDD**

**50 DDDDDDD**

**aa75-131: similar to [G-GA]_18_**

**aa161-172, 209-221: [SA]_12_ and similar**

**aa224-235: [GGS]_4_**

**aa269-304: [NG]_18_**

**307 DDDDW**

**312 DDFDW**

**317 DDDDW**

**340 DDDRF**

**345 DDDRF**

**350 DDDRW**

**355 DDDRF**

**360 DDDRW**

**365 DDD**

**381 DNDDW**

**386 DNDDL**

**421 DNGND**

**426 DNGNG**

**aa461-491: [G-S-G]_10_**

**510 GKGGYY**

**516 GKGGYY**

**522 GKGGYY**

**528 GKGGYY**

**534 GKGGYY**

CLC_5:

**1 ASGF--GGAGGA**

**11 AGGFGAGGAGGR**

**23 GGGF--G**

**23 GGGF-GFGSASARASADA-A-A-RAF**

**45 GGGFGGFGSSSASASADASASANIAA**

**aa103-115:** **[SA]_6_ and similar**

**aa118-129: [GGS]_4_**

**aa163-176: [NG]_7_**
